# Effects of different training on lower limb explosive power in youth soccer players: a systematic review and network meta-analysis

**DOI:** 10.3389/fphys.2026.1769079

**Published:** 2026-03-19

**Authors:** HuaiBin Tan, ZhiGao Yan, Cong Shao, YiFei Lin

**Affiliations:** 1 College of Physical Education, China West Normal University, Nanchong, China; 2 China Football College, Beijing Sport University, Beijing, China; 3 Sports Training College, Tianjin University of Sport, Tianjin, China

**Keywords:** explosive power, network meta analysis, physical fitness training, ranked probability, youth soccer

## Abstract

**Background:**

Football demands high lower-limb explosive power for sprinting and jumping. Traditional youth training often emphasizes aerobic endurance and heavy resistance training, which may not optimally develop such power.

**Purpose:**

To compare the effects of five different training modes, including optimal power load (OPL), resistance training (RT), high-intensity interval training (HIIT), jump training (JT) and combined training (CT), on the lower limb explosive strength of 12–19 years old adolescent soccer players.

**Methods:**

A systematic review and network meta-analysis of randomized controlled trials was conducted, with 29 studies identified from databases such as PubMed, Web of Science, Embase, EBSCO, Cochrane Library, CNKI, WanFang, and VIP. Data were analyzed using Stata 17.0, with effect sizes calculated for 10 m and 30 m sprint times, squat jump (SJ), and countermovement jump (CMJ).

**Results:**

All five training interventions showed statistically significant improvements lower-limb explosive power compared to control training. Network meta-analysis suggested that CT might be the most effective for improving SJ height (MD = 4.86 [95%CI:2.67 to 7.05], P < 0.01, SUCRA = 82.7) and CMJ height (MD = 3.33 [95%CI:2.15 to 4.52], P < 0.01, SUCRA = 65.7), while the conventional meta-analysis suggested that OPL might be the most effective for reducing 10 m sprint time (MD = −1.47 [95%CI: −2.68 to −0.26] and 30 m sprint time (SMD = −2.06 [95%CI: −3.40 to −0.72]). The effects of other interventions such as RT, HIIT, and JT were also significant, but the effects were relatively small. Subgroup analyses suggest that this effect may be most pronounced in younger athletes, with diminishing returns observed in older adolescents.

**Conclusion:**

Combining resistance and plyometric training is likely to be a highly effective strategy to optimize limb explosive power development in youth football players within the training parameters examined. The findings suggest that coaches could consider incorporating CT and OPL into their programs to enhance both sprinting and jumping capabilities. Future research may need to explore the long-term impacts and physiological mechanisms underlying these training effects.

**Systematic Review Registration:**

https://www.crd.york.ac.uk/PROSPERO/view/CRD420251249378, Identifier CRD420251249378.

## 1 Introduction

Football, as a highly competitive sport demanding exceptional physical conditioning, places particular emphasis on the development of explosive power. During matches, athletes must generate high-intensity force within extremely short timeframes to execute critical actions such as rapid acceleration, powerful shooting, and vertical jumping. These explosive movements rely heavily on lower limb explosive strength, making scientifically designed training programs essential for enhancing athletic performance in youth football players. However, in current youth football training practices in some countries—especially those with structured development pathways, physical training is still dominated by aerobic endurance, while explosive force training typically relies on traditional resistance training (Notice of the General Administration, 2024-02; Li, 2020; Yao, 2024). Although such resistance training contributes to gains in maximal strength and provides a foundational basis for explosive power development (Zeng et al., 2023), and although studies have confirmed that both aerobic endurance and resistance training can enhance lower limb strength in adolescents (Wang et al., 2021; Dou et al., 2020), the long-term efficacy of these approaches remains debated. On one hand, substantial evidence supports the positive impact of resistance training on lower limb explosiveness in young athletes. For instance, Lesinski et al. (2016) demonstrated that resistance training significantly improves muscular strength and overall sports performance in youth populations. Slimani et al. (2018) further indicated its effectiveness in enhancing lower limb muscle power, while Harries et al. (2012), McQuilliam et al. (2020) highlighted the benefits of high-load free-weight training for improving explosive metrics such as vertical jump height. On the other hand, concerns persist regarding the suitability and safety of prolonged high-load regimens in adolescent athletes. Assunção et al. (2016) suggested that high-load training may not yield optimal neuromuscular adaptations, with improper loading potentially leading to overtraining. Dahab and McCambridge (2009), Fröberg et al. (2014) emphasized the importance of individualizing training intensity according to maturational status to minimize injury risk. Borkowski et al. (2025) further warned that chronic exposure to high loads may increase the likelihood of musculoskeletal injuries in developing athletes. Moreover, Mehls et al. (2022) found that while high-load training effectively increases maximal strength, improvements in explosive power are often limited by factors such as movement velocity and rate of force development, reflecting its deficiency in the high-speed section of the strength-velocity curve. In these youth football systems, two limitations exist: a reliance on aerobic endurance training and explosive power training relies on high-load resistance training. Firstly, according to the theory of strength-speed curve, such training often ignores the cultivation of movement speed and rapid power generation ability (Maijiu, 2000; Liao et al., 2021); Secondly, the physiological adaptation brought by aerobic endurance training tends to be non-explosive movement mode, which is difficult to match the special movement demand of high frequency and high speed of football (Shui et al., 2016). These limitations highlight systemic deficiencies in training approaches aimed at enhancing the lower-limb explosive power of youth soccer players. A critical question arises: does high-load resistance training represent the optimal approach, and which training method most effectively targets explosive power. To address this, it is essential to systematically evaluate and compare the effects of five popular training methods–optimal power load training (OPL), resistance training (RT), high intensity interval training (HIIT), jump training (JT), and combined training (CT) – on the development of lower-limb explosive strength in adolescent football players. Despite the widespread use of these training modalities, there is a notable gap in the literature: no systematic review or network meta-analysis has yet compared their relative effectiveness in enhancing lower-limb explosive power in adolescent football players. This gap is crucial to address, as coaches and practitioners currently lack clear guidance on the most effective training strategies for this demographic. To fill this void, our study presents the first network meta-analysis of randomized controlled trials to compare the effects of these five prevalent training methods on key performance measures: 10 m and 30 m sprint time, squat jump, and countermovement jump height. By establishing a probabilistic ranking of intervention effectiveness, this research aims to provide evidence-based recommendations for optimizing lower-limb explosive power training in youth football, thereby supporting more targeted and effective training strategies.

## 2 Materials and methods

This Net meta-analysis adhered to the guidelines outlined in the Cochrane Handbook for Systematic Reviews of Interventions and reported following the principles of the Preferred Reporting Items for Systematic Reviews and Meta-Analyses (PRISMA), and the systematic review protocol was registered on the PROSPERO website (www.crd.york.ac.uk/Prospero), the registration ID: CRD420251249378.

### 2.1 Search strategy

We conducted a comprehensive search of the following databases from inception to 25 September 2025: PubMed, Web of Science, Embase, EBSCO, Cochrane Library, CNKI, WanFang, and VIP. The search strategy was developed using a combination of Medical Subject Headings (MeSH) and free-text terms. Key search terms included: (“training” OR “resistance” OR “high-intensity interval training” OR “plyometric training” OR “power training”) AND (“adolescent” OR “youth” OR “juvenile”) AND (“soccer” OR “football”) AND (“explosive strength” OR “lower limb power” OR “sprint” OR “jump”) AND (“randomized controlled trial” OR “RCT”). The complete search strategy for each database is provided in Attachment A1.

### 2.2 Study selection

This systematic review was conducted in accordance with the PRISMA 2020 statement and the Cochrane Handbook for Systematic Reviews of Interventions. Studies were screened independently by two reviewers based on the following predefined PICOS criteria. Population: Adolescent soccer players aged 12–19 years, as defined by the 2014 International Consensus on Youth Resistance Training (Lloyd et al., 2014) or WHO/UN criteria (Lucas, 2023). Intervention: Exercise interventions specifically targeting lower-limb explosive power, including optimal power load (OPL), resistance training (RT), high-intensity interval training (HIIT), jump/plyometric training (JT), and combined training (CT). Combined training was defined in the study as any integration of two or more of the above modalities (e.g., compound, contrast, French contrast, or running + jumping integrations). Comparison: Control group receiving only regular football training, or the same training protocol as the experimental group but with different load, frequency, or volume. Outcomes: At least one objectively measured indicator of lower-limb explosive power: 10 m or 30 m sprint time (s), squat jump (SJ) height (cm), or countermovement jump (CMJ) height (cm). Study design: Randomized controlled trials (RCTs) published from 2015 onward. Exclusion criteria: non-RCT designs, non-adolescent or non-football populations, duplicate publications, and studies with missing or unrecoverable data. Supplementary Table S1 “The table of literature characteristics” is shown in Supplementary Appendix F1.

### 2.3 Data extraction

Two researchers independently screened the eligible articles according to the inclusion and exclusion criteria, and extracted data and information, including the first author, publication year, number of male and female subjects, age of athletes, exercise program, intervention period, and outcome indicators such as 10 m sprint time, 30 m sprint time, static squat jump and their detection methods. We will discuss the main points and refer to third party opinions in case of disagreement. If the required data information is not found in the literature, we will attempt to contact the corresponding author of the literature by mail. If there is no response or no other means of obtaining data information, we will exclude the literature that extracted data information (Higgins et al., 2024). For the 10 m sprint time, 30 m sprint time, SJ and CMJ data of the experimental group and the control group, We extracted changes in their data with reference to “the Cochrane HandBook For Systematic Reviews of Interventions, Part 2,6. In the “imputation of the standard deviation of the baseline change” section of effect indicators, the calculation formula of the effect size of the change between the experimental group and the control group and the standard correlation coefficient (Higgins et al., 2024) are used to obtain the changes of the experimental group and the control group before and after the intervention.

### 2.4 Risk of bias and grade assessment

We assessed all seven domains recommended by the Cochrane ROB tool. For the domain ‘blinding of participants and personnel’, although full blinding is not feasible in exercise training interventions, we retained this domain for all included studies, in accordance with Cochrane guidance when blinding is not possible (Higgins et al., 2011; Chen et al., 2024). Based on these seven domains, each study was rated as low risk, high risk, or unclear risk. Differences of opinion between the two personnel were resolved by discussion, and a third researcher was consulted when necessary. We then assigned a composite rating to each study’s overall risk of bias as follows: studies were classified as having low risk of bias if none of these domains were rated as having high risk and if only three or fewer domains were rated as having unclear risk; Studies were classified as having a moderate risk of bias if one domain was rated as having a high risk of bias or if no domains were rated as having a high risk of bias but four or more domains were rated as having an unclear risk. Whereas all others are assumed to be associated with high ROB (McKinlay et al., 2018) We used the Recommendation Grading evaluation, Development and Evaluation (GRADE) framework to assess the reliability of the proof contributing to the outcome network estimates (Sala et al., 2014).

### 2.5 Statistical analysis

Traditional pairwise meta-analysis and network meta-analysis were performed using Stata 17.0 software (Stata Corp, College Station, TX, United State) to evaluate the difference in effect size between different training interventions or between different training and regular football training in improving lower limb power. For continuous variables, if the units of outcome indicators in each study were consistent, the weighted mean difference (MD) was used for effect size pooling. If the units were inconsistent, standardized mean difference (SMD) was used (Chen et al., 2025). The pre-test and post-test changes of the experimental group and the control group in the study were combined for effect size estimation, and the standard deviation (SD) of the change value was calculated according to the formula recommended by the Cochrane Handbook for Systematic Reviews (Version 6.5) (Higgins et al., 2024): ${SD}_{change}=\sqrt{{SD}_{baseline}^{2}+{SD}_{final}^{2}-\left(2\times Corr\times {SD}_{baseline}\times {SD}_{final}\right)}$ . Because none of the included studies reported correlation coefficients between baseline and final measurements, we assumed a correlation coefficient of r = 0.8 for all SD calculations of change from baseline (Gnambs and Schroeders, 2024). Model selection was based on the results of inter-study heterogeneity test. When I^2^ > 50% and Cochran’s Q test p value ≤ 0.10, heterogeneity was considered to be significant, and the random effect model was used. Otherwise, a fixed effects model (Higgins et al., 2003) was used. In addition to reported network estimates, between-study heterogeneity was also estimated by pairwise meta-analyses (Jackson et al., 2011), supplemented by sensitivity analyses to determine its impact. For specific outcome indicators, static squat jump height, reverse vertical jump height, 10 m sprint time and 30 m sprint time were combined using mean difference (MD) or standard mean difference (SMD), because the measurement tools and units used were the same. Heterogeneity was evaluated by I^2^ statistic and Cochran’s Q test, and the risk of publication bias was evaluated by funnel plot combined with Begg’s test. Under the frequencial framework (Salanti, 2012) and according to the current PRISMA NMA guidelines (Hutton et al., 2015), a random-effect multivariate network meta-analysis was performed. The comparison relationship between training modalities is presented in a network diagram, where node size and line thickness correspond to the number of studies of the intervention and the number of trials directly compared, respectively. The proportion of contributions of each direct comparison to the overall effect estimate was further resolved by constructing a network contribution plot. To ensure the validity of the analysis, the transitivity assumption of network structure was evaluated. Transitivity is a key assumption of NMA (Rücker and Schwarzer, 2015), which refers to the belief that indirect comparisons are valid estimates of unobserved direct comparisons (Salanti, 2012) and that all studies have uniformly distributed effect modifiers (Jansen and Naci, 2013) to ensure that effect modifiers are evenly distributed across studies. Inconsistency factor (IF) and 95% confidence interval (CI) were used to evaluate the consistency of each closed-loop. If the lower limit of 95%CI was greater than 0.40, the consistency was considered to be good. The global inconsistency test adopts the inconsistency model, and if the result is not significant (p > 0.05), the consistency model is used for estimation (Shim et al., 2017). Inconsistency was tested by the node segmentation method, and p > 0.05 was used as the criterion for reliable results. In order to comprehensively evaluate the effects of different training interventions, the area under the cumulative ranking probability curve (SUCRA) was used for ranking (Salanti et al., 2011), which is between 0 and 100. The higher the SUCRA value, the better the effect of the intervention. 100 means the best effect without uncertainty, and 0 means the worst effect without uncertainty (Mbuagbaw et al., 2017). In addition, the possible publication bias caused by small sample size was evaluated by drawing a network funnel plot combined with the symmetry criterion.

In general, all outcome measures in this study were expressed as weighted mean differences and their 95% confidence intervals to systematically compare the difference in effect sizes between different intervention subgroups and regular football training. The specific analysis strategy was to conduct pairwise meta-analysis on the 10 m/30 m sprint time data and network meta-analysis on the static squat jump/reverse vertical jump height data, so as to obtain the corresponding effect size results.

## 3 Results

### 3.1 Study selection

**FIGURE 1:**
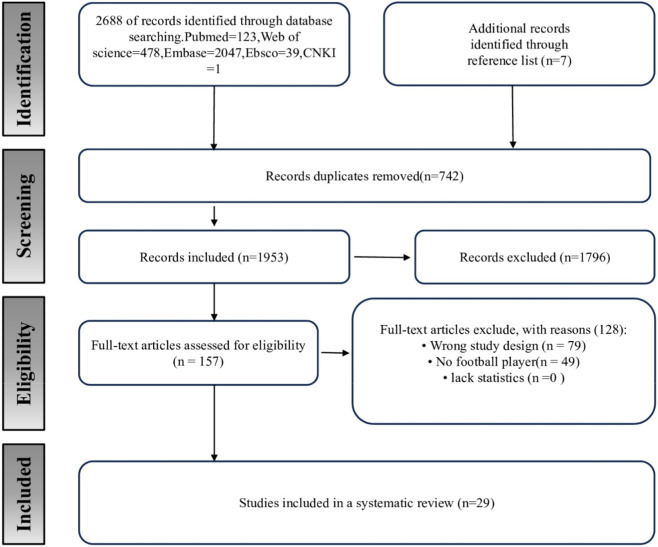
Flow chart of studies screening.

A total of 2,687 articles were retrieved, of which 742 were repeated. A total of 157 studies were initially screened by reading the titles and abstracts of the literature. After intensive reading of the full text, 29 studies were finally included and their research characteristics were summarized. In the final included studies, some control groups only received regular football training, and the intervention methods of the experimental group were all training strategies that could develop the explosive power of the lower limbs. However, exercise protocols varied across studies and involved five training measures, including optimal power, resistance training, high-intensity interval training, rapid stretching and bouncing, and combined-strategy training. Five studies used optimal power training (Derakhti et al., 2021; Loturco et al., 2013; Loturco et al., 2016; Loturco et al., 2020; Ribeiro et al., 2020), five studies used resistance training (Abade et al., 2021; Falces-Prieto et al., 2021; McQuilliam et al., 2023; Gonzalo-Skok et al., 2019; Borges et al., 2016), two studies used high-intensity interval training (Nayıroğlu et al., 2022; Michailidis et al., 2023), and seven studies used plyometric training (Loturco et al., 2015; Jlid et al., 2019; Jlid et al., 2020; Norgeot and Fouré, 2024; Rosas et al., 2016; Kobal et al., 2017; Bianchi et al., 2019). Combined strategy training for 10 study (Zghal et al., 2019; Al et al., 2022; Barra-Moura et al., 2024; Yu et al., 2020; Schneiker et al., 2023; Wallenta et al., 2016; Fischetti et al., 2019; Maio Alves et al., 2010; Zhu, 2024; Vasileiou et al., 2024). The time span of these exercise interventions varied by study design, ranging from 3 weeks to 20 weeks, with most intervention periods of 8 weeks. The frequency of exercise was 2–3 times per week, and 2 times per week in most studies. A total of 869 adolescent players were involved in all studies, and exercise experiments were performed under supervision. All study outcome measures were evaluated using professional equipment such as optical instruments. The study selection process is illustrated in Figure 1.

### 3.2 Risk of bias and GRADE assessment

**FIGURE 2:**
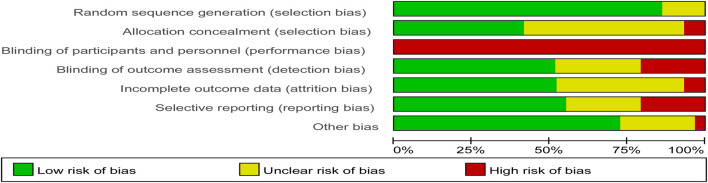
Risk of bias graph.

**FIGURE 3:**
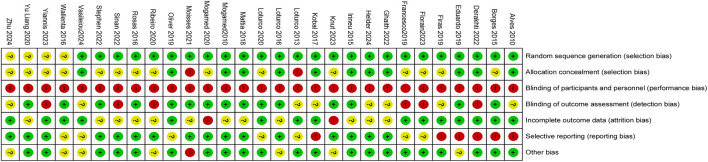
Risk of bias summary.

Review Manager 5.3 software was used to draw Risk assessment charts, such as Risk of Bias (Figure 2) and Risk of bias summary (Figure 3), to summarize the risk results of each study. In addition, we referred to a study (Cipriani et al., 2018) in which the total risk of the literature and the method of pairwise comparison evaluation are attached (Attachment A2). The specific methods are as follows: In the period, we combined the Risk of Bias results with the evidence contribution chart (Attachment A3) to draw a risk evaluation bar chart (Attachment A4), which assigns different weights to Low/Unclear/high, 0/-1/-2, respectively. If the percentage of low-risk ROB is less than 30%, the percentage of low-risk ROB is less than 30%. And if the percentage from intermediate-risk ROB exceeded 70%, a downgrade of one grade was considered to determine Study limitation. The Indirectness was evaluated by clinical and methodological similarity. If there is no subgroup analysis, it will not be downgraded. If the pairwise comparison only comes from indirect comparison in the contribution graph, it will be downgraded by one level. We judge Inconsistency from two domains: heterogeneity and consistency. Specifically, we judge the heterogeneity of pairwise comparison by observing each pairwise comparison forest map (Attachment A5) and judge the consistency according to the inconsistency test results of reference nodes (Attachment A7). At the same time, we evaluated Imprecision by sample size and the gap between ranking values (Attachment A6) (Salanti et al., 2011). Finally, the funnel plot was used to determine Publication bias. The final Grade results are as follows (Table 1).

**TABLE 1:** Grade assessment result.

	Study	Imprecision	Inconsistency	Indirectness	Publication	Grade	Downgrade rationale
	limitation				bias		
CON vs OPL	Downgrade	Downgrade	No downgrade	No downgrade	No downgrade	Low	①②
CON vs RT	No downgrade	Downgrade	No downgrade	No downgrade	No downgrade	Moderate	②
CON vs HIIT	No downgrade	No downgrade	Downgrade	No downgrade	No downgrade	Moderate	③
CON vs JT	No downgrade	No downgrade	No downgrade	Downgrade	No downgrade	Moderate	④
CON vs CT	No downgrade	No downgrade	No downgrade	Downgrade	No downgrade	Moderate	④
OPL vs JT	No downgrad	No downgrade	No downgrade	Downgrade	No downgrade	Moderate	④
RT vs CT	No downgrade	No downgrade	No downgrade	Downgrade	No downgrade	Moderate	④
JT vs CT	No downgrade	Downgrade	No downgrade	No downgrade	No downgrade	Moderate	②
OPL vs RT	Downgrade	Downgrade	Downgrade	Downgrade	No downgrade	Very low	①②③④
OPL vs HIIT	No downgrade	Downgrade	Downgrade	Downgrade	No downgrade	Low	②③④
OPL vs CT	No downgrade	Downgrade	Downgrade	Downgrade	No downgrade	Low	②③④
RT vs HIIT	No downgrade	Downgrade	Downgrade	Downgrade	No downgrade	Low	②③④
RT vs JT	No downgrade	Downgrade	Downgrade	Downgrade	No downgrade	Low	②③④
HIIT vs JT	No downgrade	Downgrade	Downgrade	Downgrade	No downgrade	Low	②③④
HIIT vs CT	No downgrade	Downgrade	Downgrade	Downgrade	No downgrade	Low	②③④

^①^When the contribution of the lower RoB comparison was less than 30% and the contribution of the medium RoB comparison was greater than or equal to 70%, we lowered the rating by one level (Cipriani et al., 2018). ^②^ The imprecision of the ranking results was assessed according to the gap between the ranking values. If the gap between the ranked values of each intervention is small, indicating poor stability, the intervention will be reduced by one level (Salanti et al., 2014). ^③^Based on the p judgment of the node cut method, if the p value of pairwise comparison is greater than 0.5, it is not downgraded, otherwise it is downgraded by 1 level. ^④^Evidence from indirect comparisons alone is downgraded by one level.

### 3.3 Direct pairwise and network meta-analyses

**FIGURE 4:**
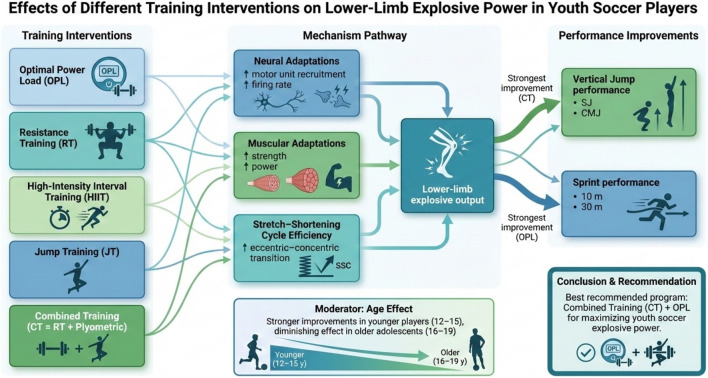
Results summarized.

This meta-analysis shows that different training interventions have different effects on lower limb explosive power in youth soccer players, which is also reflected in various sports performance measures. Summarized in Figure 4.

#### 3.3.1 Direct pairwise meta-analyses

##### 3.3.1.1 10 m sprint time (s)

**FIGURE 5:**
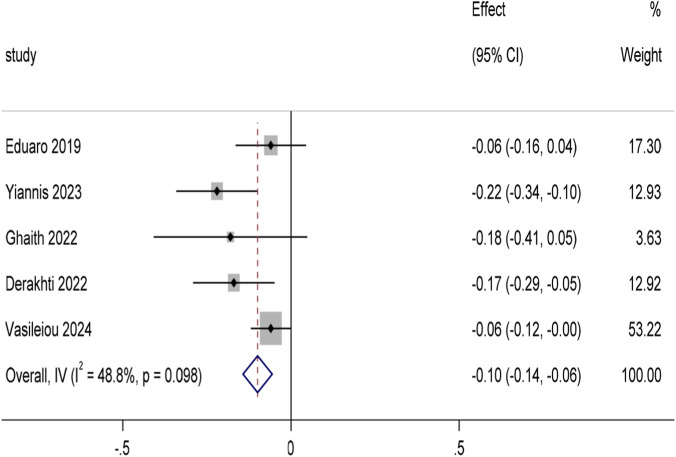
Forest plot of 10 m sprint time for all included studies. In Figure 6, Study 2 used OPL, Study 3 used RT, Study 4 used HIIT, and Study 6 used CT.

**FIGURE 6:**
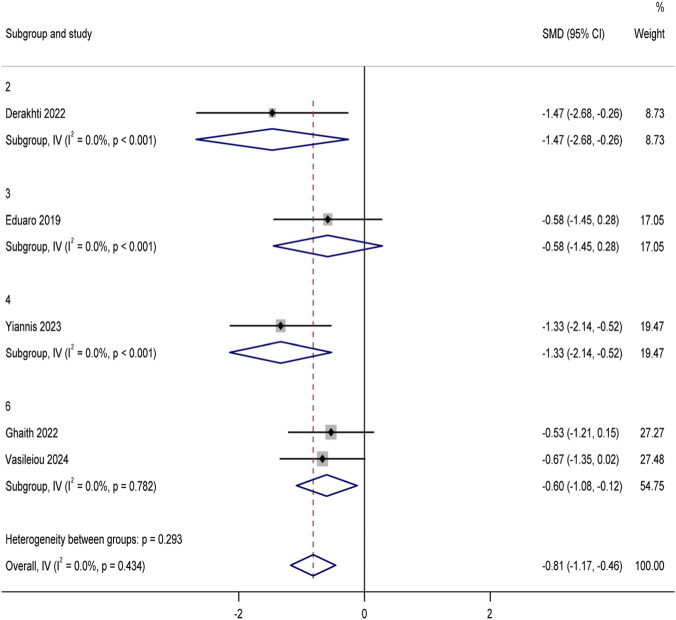
Subgroup analysis of 10 m sprint time by training modality.

**FIGURE 7:**
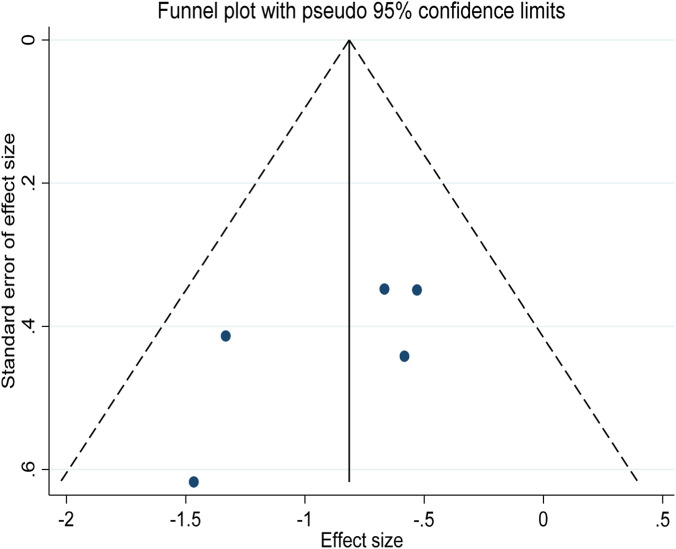
Funnel plot for publication bias assessment of studies reporting 10 m sprint time.

**FIGURE 8:**
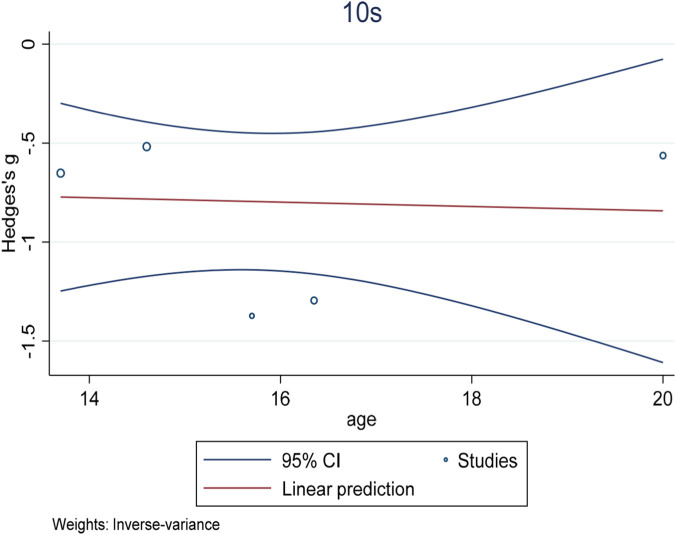
Bubble plot of showing the relationship between mean age (years) and effect size (Hedges’ g) for 10 m sprint time.

Among the 29 included articles, a total of 10 studies reported the 10 m sprint time (unit: s) indicator. After screening and excluding five studies in which the control group did not use traditional football training, data from five studies (Derakhti et al., 2021; Abade et al., 2021; Michailidis et al., 2023; Al et al., 2022; Vasileiou et al., 2024) were finally included in the conventional meta-analysis, with a total of 149 youth football players participating in the trials. The pooled analysis of 10 m sprint time using Stata 17.0 software (Figure 5) showed that the experimental group showed a significant improvement trend compared with the traditional football training control group (MD = −0.1, 95%CI: Although there was moderate heterogeneity among the studies (I^2^ = 48.8%, p = 0.098), we further performed sensitivity analyses to verify the robustness of the results (see Attachment B1). To explore the differences in the effects of different training modes, we conducted a subgroup analysis (Figure 6), and the results showed that all four training modes had a positive effect on 10 m sprint performance (MD = −0.81 [95%CI: −1.17 to −0.46]), with low intra-group heterogeneity (I^2^ = 0%, p = 0.434). Specifically, optimal power training (4 weeks of optimal power loading sprints twice a week) showed the optimal effect size in improving performance in the 10 m sprint (MD = −1.47 [95%CI: −2.68 to −0.26]), superior to resistance training (20 weeks of barbell hip press and half-squat once a week), high-intensity interval training (4 weeks of high-intensity interval sprints twice a week), and combined training (8 weeks of combined training). Slalom and sprint twice weekly). In addition, to further examine the extent to which training methods explained the difference in effect size, we developed a meta-regression model. The results (Attachment B1) showed that the explanatory power of the model was limited (*R* ^2^ = −28.75%), and the training method did not reach statistical significance as an independent variable (experimental group coefficient ES = 1.01 ± 0.02, t = 0.52, p = 0.636; Control group coefficient ES = 0.83 ± 0.09, t = −1.16, p = 0.027), indicating that the type of intervention was not the main factor responsible for the difference in effect size among the studies. In addition, to visualize the relationship between intervention type and age, a bubble plot (Figure 7) was constructed, which showed no clear trend in effect size distribution according to age. In the assessment of publication bias, funnel plot visual tests did not reveal significant asymmetry (Figure 8). The results of Begg’s test (p = 0.624) further confirmed that there was no statistically significant publication bias (Attachment B1), supporting the reliability of the results of this study.

##### 3.3.1.2 30 m sprint time(s)

**FIGURE 9:**
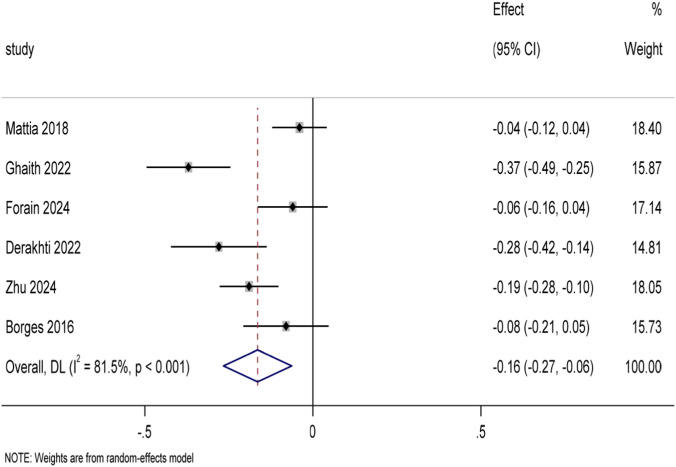
Forest plot of 30 m sprint time for all included studies.

**FIGURE 10:**
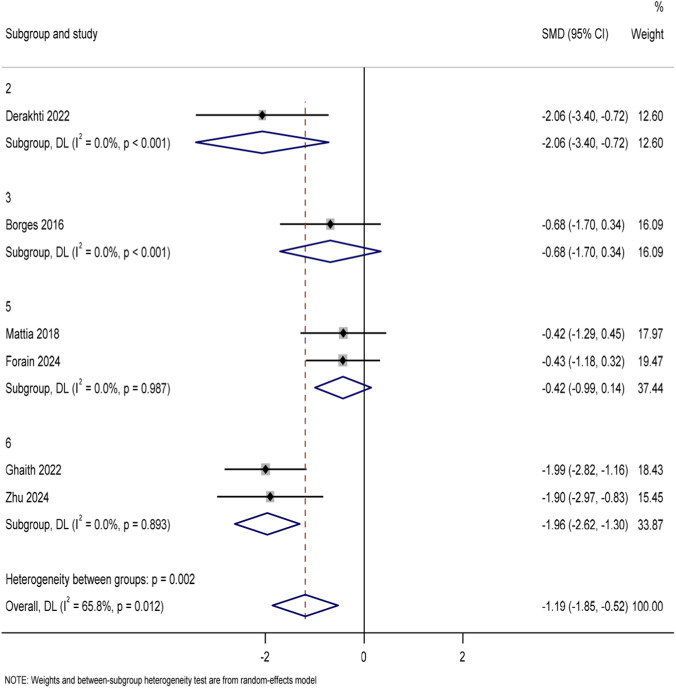
Subgroup analysis of 30 m sprint time by training modality.

**FIGURE 11:**
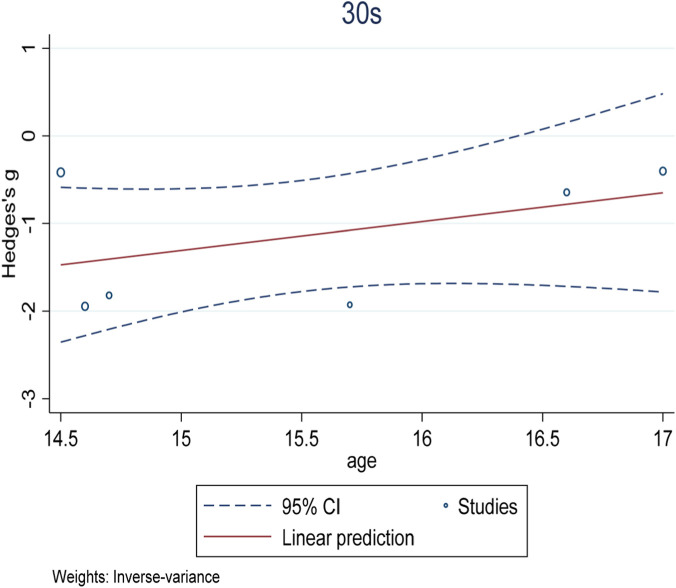
Bubble plot of illustrating the association between mean age (years) and effect size for 30 m sprint time.

A total of 7 studies reported the 30 m sprint time (unit: s) in the 29 included articles. Data from six studies (Derakhti et al., 2021; Norgeot and Fouré, 2024; Bianchi et al., 2019; Al et al., 2022; Borges et al., 2016; Zhu, 2024) involving a total of 133 youth soccer players were included in the conventional meta-analysis after exclusion of one study in which the control group did not use traditional soccer training. The pooled analysis of 30 m sprint time using Stata 17.0 software (Figure 9) showed that the experimental group showed an improvement trend compared with the traditional football training control group (SMD = −0.16, 95%CI: There was a high degree of heterogeneity among the studies (I^2^ = 81.5%, p < 0.001), the Potential effect modifiers include—such as age, intervention duration, or training frequency—may further influence sprint outcomes. Sensitivity analysis was further performed to verify the robustness of the results (see Attachment B2). Similarly, to explore the differences in the effects of different training modes, a subgroup analysis was performed (Figure 10), which showed that all four training modes had a positive effect on 30 m sprint performance (SMD = −1.19 [95%CI: −1.85 to −0.52]), and the intra-group heterogeneity was high (I^2^ = 65.8%, p = 0.012). Specifically, optimal power training (4 weeks of 20 m sprinting at optimal power load twice a week) (SMD = −2.06 [95%CI: −3.40 to −0.72]) and combined training (8 weeks of a integration of obstacle jumps and sprinting twice a week or French contrast training three times a week) showed high effect sizes in improving 30 m sprint performance. The effect was better than that of resistance training (7 weeks, weight sprinting exercise once or twice a week) and rapid stretching and bouncing training (8 weeks, deep jump, obstacle jump, etc., once or twice a week). Similarly, to further examine the extent to which training mode explained the difference in effect size, we developed meta-regression models. The results (Attachment B2) showed that the explanatory power of the model was limited (*R* ^2^ = −29.26%), and the training method did not achieve statistical significance as an independent variable (experimental group coefficient ES = 0.99 ± 0.04, t = −0.14, p = 0.896; Control group coefficient ES = 0.86 ± 0.17, t = −0.71, p = 0.518), which also indicated that the type of intervention was not the main factor responsible for the difference in effect size among the studies. In addition, in order to visually show the relationship between the type of intervention and age, we generated a bubble plot (Figure 11), which revealed a negative association between age and the magnitude of sprint improvement, suggesting that younger adolescents (≈14.5 years) may benefit more than those aged >15.5 years, but this observation is only descriptive.

**FIGURE 12:**
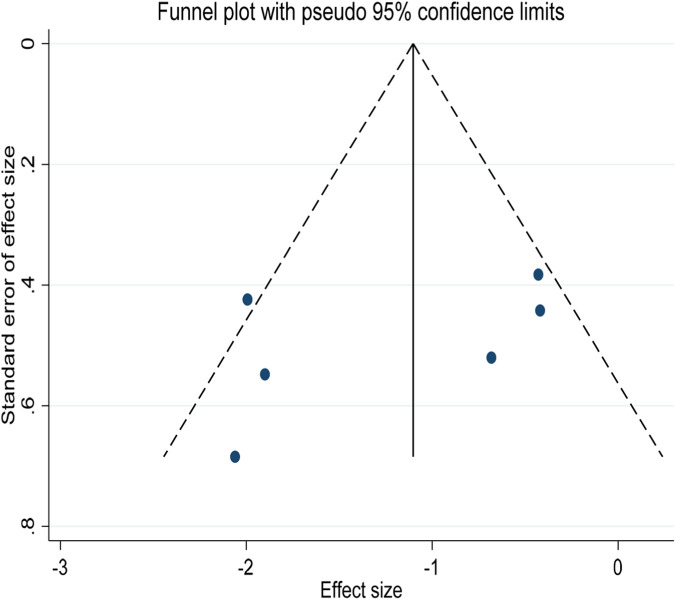
Funnel plot for 30 m sprint time studies.

In the assessment of publication bias, funnel plot visual test did not find significant asymmetry (Figure 12). The results of Begg’s test (p = 0.348) further confirmed that there was no statistically significant publication bias (Attachment B2), supporting the reliability of the results of this study.

#### 3.3.2 Network meta-analysis

##### 3.3.2.1 Network evidence diagram

**FIGURE 13:**
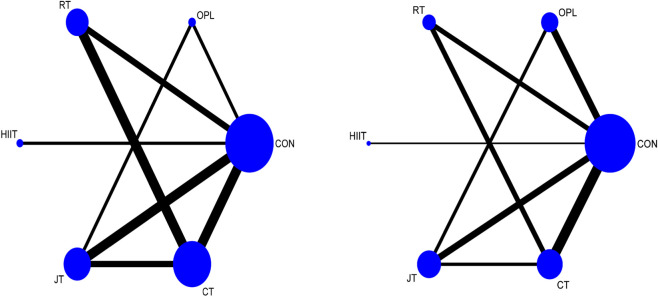
Network evidence. Note: OPL, Optimal power load training; RT, Resistance training; HIIT, High intensity interval training; JT, Jump training (plyometric training); CT, Combined training; Con, Contrast group.

**FIGURE 14:**
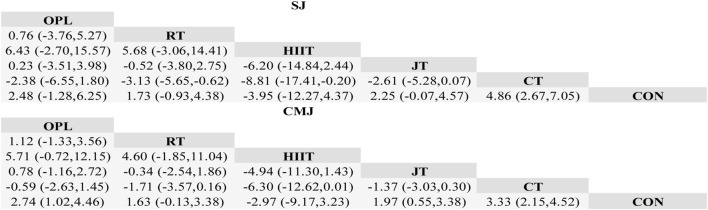
Inverted triangle results of network meta-analysis.

Figures 13, 14 show the network evidence diagram of different training methods on the lower limb explosive power of adolescent soccer players. The most common type of intervention was CT, and the least frequent was HIIT. In the Network evidence map of SJ and CMJ indicators, OPL vs RT, OPL vs HIIT, OPL vs CT, RT vs HIIT, RT vs JT, HIIT vs JT, HIIT vs HIIT vs CT was indirect comparison evidence, and all other groups were mixed comparison evidence.

##### 3.3.2.2 Inconsistency test

The inconsistency of the SJ and CMJ outcome measures was tested by means of the ring inconsistency test, the inconsistency model, and the node segmentation method. The results (Attachment C1) of the loop inconsistency test showed that the inconsistency of all triangular closed loops between SJ and CMJ outcome indicators was not significant (P > 0.05), and all closed loops showed good consistency. The inconsistency model test showed that except for the comparison of CT vs Con in SJ outcome indicators (P < 0.05) and RT vs Con, JT vs Con, and CT vs Con in CMJ indicators (p < 0.05), the other comparisons were all P > 0.05, and the inconsistency was not significant, so the consistency model could be used for analysis. The node segmentation method showed that the direct comparison evidence of each outcome index was consistent with the indirect comparison evidence (all P > 0.05), and the results were highly reliable.

##### 3.3.2.3 Combined effect size analysis and ranking results

**FIGURE 15:**
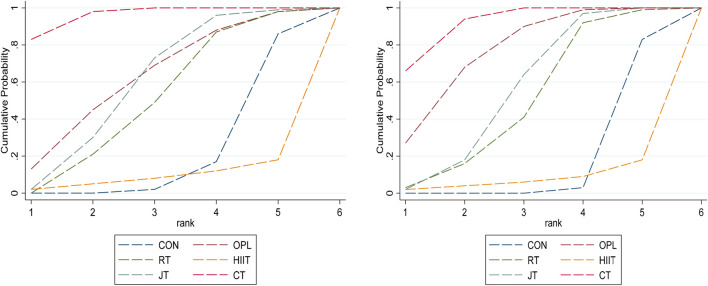
SJ and CMJ Cumulative probability ranking plot.

For the SJ index, compared with the control group, CT (MD = 4.86 [95%CI:2.67 to 7.05], P < 0.01) had statistically significant improvements to the individual SJ index of young football players. The results of SUCRA probability ranking showed that CT had the highest probability as the best intervention method among those compared (SUCRA = 82.7). Followed by OPL (SUCRA = 13.1) and HIIT (SUCRA = 2.0), JT (SUCRA = 1.8), RT (SUCRA = 0.4), Con had the lowest probability (SUCRA = 0). For CMJ index, compared with the control, CT (MD = 3.33 [95%CI:2.15 to 4.52], P < 0.01), an OPL (MD = 2.74 [95% CI:1.02 to 4.46], P < 0.01), JT (MD = 1.97, [95%CI 0.55 to 3.38], P < 0.01) also had statistically significant improvement to the individual CMJ index of adolescent football players. The results of SUCRA showed that CT had the highest probability as the best intervention (SUCRA = 65.7). OPL (SUCRA = 27.3), RT (SUCRA = 2.5), JT (SUCRA = 2.3), HIIT (SUCRA = 2.2), and Con (SUCRA = 0) had the lowest probability. The cumulative probability rankings for SJ and CMJ are shown in Figure 15.

##### 3.3.2.4 Publication bias or small sample effect test

The funnel plot was used to test the publication bias of the outcome indicators, and it was found that the funnel plot of each outcome indicator had good symmetry, and the publication bias or small sample effect had little influence, as shown in the results (Attachment C2).

## 4 Discussion

This study systematically evaluated and compared the effects of five training modalities on lower limb explosive power in adolescent soccer players. Building upon previous research, which has typically focused on only one or two intervention types, we employed a network meta-analytic approach to enable indirect comparisons across all five modalities and generate a probabilistic ranking of their relative effectiveness. The results indicate that Combined Training and Optimal Power Load Training are the most effective interventions for improving vertical jump and sprint performance, respectively. These differential outcomes may be attributed to the principle of training specificity, with distinct physiological adaptations underlying each modality-factors that are further explored in the following sections.

### 4.1 Key findings

The present analysis suggests that several training modalities may enhance lower-limb explosive power in youth soccer players. The most notable improvements in vertical jump height appeared to occur following Combined Training, which integrates plyometric and resistance exercises. This is consistent with previous evidence indicating that such combined approaches could promote muscular strength and jump performance synergistically (McKinlay et al., 2018). Meanwhile, Optimal Power Load Training may be particularly effective for improving sprint performance, especially over short distances such as 10 and 30 m, supporting its potential role in developing explosive acceleration capabilities. These differential outcomes may be explained by distinct adaptive mechanisms.

Firstly, The observed superiority of CT in improving jump performance may be explained by a potential synergistic mechanism that could integrate distinct adaptations from its components. From a theoretical perspective, the plyometric exercises are thought to primarily enhance neurological efficiency by optimizing the stretch-shortening cycle and increasing tendon stiffness (Kubo et al., 2017). Which might contribute to more rapid force production. Simultaneously, the resistance training component is associated with morphological adaptations, such as increases in the cross-sectional area of fast-twitch muscle fibers, thereby raising maximal strength capacity. Therefore, it is plausible that the combination of these adaptations-potential improving neuromuscular coordination and contractile capacity-could work in concert to enhance both the rate of force development and maximal strength, offering one plausible, dual-pathway mechanism underlying the efficacy of CT as suggested by the present analysis.

Secondly, OPL is by movement against a load that maximizes mechanical power output. This specific stimulus is hypothesized to preferentially induce velocity-specific neural adaptations (Sarabia et al., 2017). These may include the optimization of high-threshold motor unit recruitment and the enhancement of intermuscular coordination at movement velocities approximating those required in maximal sprints. In contrast, traditional RT typically employs higher loads, shifting its adaptive emphasis toward the force-end of the force-velocity relationship (Maijiu, 2000). The primary adaptations are thought to be increased maximal force production and morphological changes in muscle, which may have a less direct transfer to the high-speed, lower-force demands of sprinting compared to OPL. Concurrently, HIIT, as operationalized in the included studies through repeated sprints, primarily targets metabolic and cardiovascular systems. Adaptations such as improved anaerobic capacity and between-bout recovery are central. While this provides a sport-specific metabolic challenge, the direct stimulus for neuromuscular structural adaptation or neural efficiency is considered less focal than in OPL or RT, potentially explaining its relatively smaller effect sizes on pure explosive power metrics in this analysis. Furthermore, subgroup analyses indicated that the improvement in sprint performance was more pronounced among younger athletes (approximately 14.5 years). This observation may be attributed to greater neuromuscular plasticity at earlier developmental stages or to greater adaptability in less trained individuals.

### 4.2 Comparison with existing studies

The findings of this study align with prior literature underscoring the value of integrated and strength-oriented training for enhancing lower-limb explosive power in adolescent athletes. Previous research has established that both resistance training and plyometric training can improve strength, albeit through distinct mechanisms. For instance, studies by Lesinski et al. (2016), Slimani et al. (2018) emphasized the role of resistance training in augmenting muscle strength, which serves as a foundation for explosive power. Similarly, plyometric training has been widely documented to improve vertical jump and sprint performance (Loturco et al., 2020; Ribeiro et al., 2020).

The present analysis extends this evidence by directly comparing five distinct training modalities, offering more nuanced insights into their relative effectiveness in youth soccer. A notable contribution is the observed superiority of Combined Training in improving both vertical jump and sprint performance, consistent with earlier suggestions that integrated approaches may promote a more balanced development of explosive qualities (Vasileiou et al., 2024; Falces-Prieto et al., 2021). In contrast, High-Intensity Interval Training (HIIT), as implemented in the included studies—which primarily involved repeated sprint intervals—appeared less impactful on explosive power outcomes compared to CT or OPL. This may reflect a greater emphasis on metabolic and endurance adaptation within such protocols, highlighting that targeted neuromuscular training remains important for optimizing explosive capabilities in young athletes.

### 4.3 Implications for practice

The results of this study have immediate practical implications for football coaches and fitness coaches, particularly in the context of youth football development. Based on our findings, a integration of resistance and plyometrics training (as shown in CT)appears to be a promising strategy for enhancing explosive power. The emphasis on optimal power load training, particularly to improve sprint performance, which is a key component of football matches, may also be beneficial to consider. Furthermore, our study underscores the potential importance of age-specific adaptation in training programs. The results suggest that the effect of training on 30 m sprint performance may diminish with age, especially after the onset of puberty, suggesting that interventions may be most effective in early adolescence, around 14–15 years of age. That is, early adolescents may respond more positively to strength-based training.

### 4.4 Study limitations

Despite the strengths of this study, several limitations must be acknowledged. First, the small number of included studies and the relatively small sample sizes in some of the studies may limit the generalizability of our findings, For instance, the network estimates for HIIT are based on only two studies, which increases the uncertainty around its effect estimates and suggests that its lower ranking in the SUCRA analysis should be interpreted with particular caution. Second, the heterogeneity of intervention protocols across studies, particularly with respect to intervention timing, frequency, and intensity, poses challenges for direct comparisons. Third, while our network meta-analysis considered various training interventions, differences in participant characteristics such as age, soccer skill level may have introduced additional variance into the results. Although we conducted subgroup analyses by training method for the 30 m sprint results, there was still residual heterogeneity in some subgroups. Due to the small number of studies in each subgroup, we were unable to conduct further meta-regression analysis or stratified analysis to examine the effects of continuous or categorical moderator factors such as average age, intervention duration, or training frequency. Fourth, an assessment of training results that is based on a limited number of outcome measures - sprint time and jump height - may not fully capture the broader effects of these programs on overall exercise performance.

## Data Availability

The original contributions presented in the study are included in the article/Supplementary Material, further inquiries can be directed to the corresponding author.
