# Supplementary material for: Effects of different training on lower limb explosive power in youth soccer players: a systematic review and network meta-analysis
Source: Front Physiol. 2026 Mar 19;17:1769079. doi: 10.3389/fphys.2026.1769079 (PMC13043373; doi:10.3389/fphys.2026.1769079)
Supplement: Supplementary file 1 [file DataSheet2.pdf]

Studys	Information of athletes					
	Profession	Number（I/C）	Sex（Male%)	Years	Weeks	Frequency (times/week)
Derakhti (2022)	football	27（19/8）	100%	15.7±0.5	4	2t/w
Loturco (2013)	football	32（16/16）	100%	19.18±0.72	6	2t/w
Ribeiro (2020)	football	16（8/8）	100%	18.4±0.49	7	2t/w
Loturco (2016)	football	17（9/8）	100%	18.4±1.2	6	2t/w
Loturco (2020)	football	23（12/11）	100%	18.3±0.7	4	3t/w
Loturco (2015)	football	24（12/12）	100%	18.2±0.6	3	2~5t/w
ohamed（2019	football	28（14/14）	100%	11.8±0.4	8	2t/w
Firas（2019）	football	31（23/8）	100%	14.5±0.52	7	2t/w
ohamed2（201	football	27（14/13）	100%	19.0±0.9	6	2t/w

Eduaro（2019）	football	24（16/8）	100%	16.56±0.56	20	1t/w
Yiannis(2023)	football	29 (15/14)	100%	16.35±0.35	4	2t/w
Moisses(2021)	football	144 (75/69)	100%	15.83±1.27	15	2t/w
Ghaith（2022）	football	34 (17/17)	100%	14.6±0.4	8	2t/w
Sinan（2022）	football	24（12/12）	0%	18.5±2.1	8	3t/w
Helder（2024）	football	21 (13/8)	100%	15.3±1.1	6	2~3t/w
Forain（2024）	football	28 (14/14)	100%	14.5±0.5	8	2t/w
Rosas(2016)	football	63 (42/21)	100%	12.1±2.2	6	2t/w
Yu (2020)	football	20 (10/10)	100%	15.08±0.76	4	2t/w

[illegible]ephen（2022）	football	22（15/7）	100%	18±1	6	1t/w
Knut（2022）	football	16 (8/8)	100%	19±2	6	4t/w
Borges(2016)	football	20（9/11）	100%	16.6±0.6	7	1~2t/w
Kobal(2017)	football	23(12/11)	100%	15.9±1.2	6	2t/w
Wallenta(2016)	football	18（10/8）	100%	18.1±1.6	6	2t/w
[illegible]attia（2018）	football	21（11/10）	100%	17±0.8	8	1~2t/w
[illegible]rancesco(2019	football	24（12/12）	100%	13.2±0.9	8	2t/w
Alves(2010)	football	23 (17/6)	100%	17.4±0.6	6	1~2t/w
Zhu (2024)	football	20 (10/10)	NR	14.7±0.74	8	3t/w

asileiou（2024	football	35（18/17）	100%	13.7±0.8	8	2t/w
Oliver（2019）	football	35（21/14）	100%	15.6±1.0	10	2t/w

Exercise Protocol					
Practice the movement	Interval between groups	Volume (group × times)	Training Strategies	Intensity	Instruments
20 m resistance sprint	3m	5~8 × 1	OPL	dec, Maximum	1080 Running system
Depth of jump<br>Squat deep	2m	4 × 4~8	OPL	45.2±3.0%1RM	OptoJump<br>Linear sensor<br>Smith's machine
Half squat<br>Hip push<br>Jump	2m	4 × 4~8	OPL	OPL：66~90kg	barbell<br>Beast sensor<br>Timer timer<br>Platform of contact
Jump squats<br>Push up	2m	6 × 4~8	OPL	1.10xOPL	Smith's machine<br>Olympic stick<br>T-Force<br>Smart Jump/Speed
Jump squats	NR	6 × 6	OPL	OPL±20%	Smith's machine<br>Linear sensor<br>Photoelectric timer
Vertical jump<br>Level jump	3m	4~6×8~10	JT	Maximum effort	Force measuring table<br>Photoelectric timer
Alternate feet in the circle<br>Jump with your feet and cross your legs<br>Jump in circles with both feet<br>(Multi-direction jump)	NR	3~4 ×6~12	JT	Self weight	Jump rope<br>An obstacle<br>Optojump<br>Star offset balancing device
Half squat<br>5~20m sprint<br>Hurdle hurdle	3m	2~4×6~8	Resistance + JT	30%~60%1RM<br>30~60cm	Optojump<br>Electronic photocells<br>Timer(Brower Timing System）
Squat Jump (bench)<br>Alternate feet<br>Jump barrier	1m	5~6×4~16	JT	30~60cm	Optojump<br>Photoelectric sensor

(Multi-direction jump)					
Back half squat<br>Barbell hip push	1m30s	3×4~10	RT	4~10RM	Optojump<br>Infrared Timer system (Globus Ergo Timer)<br>Photoelectric sensor、Barbell
High-intensity interval sprint	NR	2~3/4 mins	HIIT	YIR1 Maximum	NR
Self-reorganization: push-ups, TRX training,<br>Loading group: bench press, squat, hip press, etc	NR	4 ×8~12	RT	Self-reorganization: self-weight<br>Overload group: 50 to 65%1RM	Elastic band<br>TRX<br>barbell
Jump over hurdles<br>Jump sideways over hurdles<br>Step of bounce<br>One foot jump + sprint	1m30s	3~6 ×6	JT + run	NR	Hurdle hurdle
High-intensity interval sprint	4m	2~3×6	HIIT	90~95%VO2max	NR
Squat deep<br>High jump<br>Sprint, etc.	NR	Each station /6 times	CCT	80%~90%1RM	Force measuring table<br>Photoelectric cell
Jump over hurdles<br>Reverse squat jump<br>Step of bounce	1m	NR	JT	Self weight	Optojump<br>My Jump app
Side jump<br>One leg/leg level<br>Vertical jump	1m	2×4~8	JT	0~15%weight	Dumbbell<br>Measuring tape<br>Electronic contact pad<br>Radar gun
Squat deep<br>Sprint	NR	1×6	Resistance + JT	at deep：60%1	Vertical jump test system<br>Infrared ray technology<br>EPTS

Parallel back squat	3m	2~3×4~8	RT	80%~90%1RM	Optojump<br>Timing door<br>barbell
Squat<br>Squat jump<br>Stiff pull<br>Gao La	4m	4×6	Resistance + contras	Squat: 85% 1RM<br>Squat jump: 30% 1RM	Plyometric Power System
Sprint (sled load)<br>Jump over hurdles<br>Fall down jump	45~120s	RS：2-7×1<br>PT：2-7×10	RT	10~13%weight	Photoelectric system<br>Weighted sled
Weight bearing jump<br>Dead weight jump	NR	2~6×6	JT	weight/self-wei	Platform of contact<br>Force measuring table<br>Dumbbell
Squat deep<br>Jump over hurdles<br>20 meter Dash	1.5~3m	Squat: 3×5<br>Jump + Sprint: 3×3	JT + run	60~70%1RM<br>Height of hurdle：64~69cm	Linear sensor<br>Force measuring table
Jump deep<br>Level jump<br>Obstacle jump	NR	4×5~6	JT	60cm (jump depth height)<br>15cm (obstacle height)	Photoelectric system
Jump rope<br>Jump over hurdles<br>Barbell squats<br>Barbell lunges,	60~90s	Enhanced type: 1-3 × variable<br>Resistance: 3×10~12	Resistance + JT	Load load<br>(+5%/ week)	Beast sensor<br>Stop watch
Squat + high leg run + sprint<br>Heel raise + vertical jump + head ball<br>Leg extension + sitting jump + deep jump	NR	3 stations ×6	CT	80~90%1RM<br>(+5%/2 weeks)	Ergojump<br>Photoelectric timing system
Barbell squats<br>Self-weight squat jump<br>Dumbbell half squat jump<br>Elastic belt helps jump	3~4m	4×3~8	CT	70-80%1RM (squat)<br>30%1RM (Dumbbell)<br>Self-weight (Other)	barbell<br>Dumbbell<br>Elastic band<br>Force measuring table

					Speed meter
Multi-directional double/unilateral jumps	40s~5m	2~4×1~6	CT	NR	Timing door (WITTY System)
Sprint					Optojump
Jump → run					
Strong and weak side foot resistance	3m	2×6~10	RT	Speed/strength ratio (1-3)	Optojump<br>Force measuring table

Conclusions
5~30m sprint speed (6~8%) ↑<br>Maximum strength ↑<br>SJ height ↑<br>CMJ height ↑<br>10 m sprint time ↓
30m sprint capacity ↑<br>SJ height ↑<br>CMJ height ↑
Jump height ↑<br>Sprint ↑
5~10m sprint speed ↑
VJG: CMJ height and peak ↑10-20m acceleration ↑<br>HJG: jump distance and peak ↑10m acceleration ↑
SJ height ↑<br>CMJ height ↑
COMB: 5m sprint time ↓ 4.7%<br>SJ, CMJ, DJ height ↑<br>PLYO/sprint group: SJ, CMJ, DJ height ↑
SJ height ↑<br>CMJ height↑

Horizontal jump distance ↑<br>10, 20m sprint ability ↑
10m speed ↑
CMJ height ↑ (OG>SG)
SJ height ↑<br>CMJa height ↑<br>10~30m sprint performance ↑
CMJ height↑<br>Long jump ↑
CMJ height ↑
Vertical/horizontal jump performance ↑<br>Sprint time (5m, 10m, 15m, 30m) ↓
Vertical/horizontal jump height ↑
20-meter sprint time ↓<br>CMJ height↑

1RM ↑<br>SJ/CMJ height ↑
1RM half squat ↑<br>Squat jump peak power ↑<br>20m sprint ↑
30m sprint performance ↑
SJ/CMJ height ↑
KT: 1RM↑ CMJ↑<br>BT: 5m, 10m sprint ↑
Standing long jump ↑<br>One leg triple jump ↑<br>10~40m sprint performance ↑
SJ (power/speed/force/height) ↑<br>20m sprint performance ↑
5m sprint time ↓<br>15m sprint time ↓<br>SJ height ↑
30m sprint performance ↑<br>Standing long jump ↑<br>CMJ height ↑

CMJ height↑
10/20m sprint performance ↑
CMJ height ↑
