# Supplementary material for: Effects of different training on lower limb explosive power in youth soccer players: a systematic review and network meta-analysis
Source: Front Physiol. 2026 Mar 19;17:1769079. doi: 10.3389/fphys.2026.1769079 (PMC13043373; doi:10.3389/fphys.2026.1769079)
Supplement: Supplementary file 2 [file Presentation1.zip › 附件/10M/B1.docx]

**1.** **Results of heterogeneity test:**

It can be seen in the figure above that I2 is less than 50% and p is greater than 0.05, so the heterogeneity is small.

**2. Results of sensitivity analysis**

**3.Meta regression analysis to test the source of heterogeneity**

**4. Publication bias test results:**

**5. For age, regression analysis to test the source of heterogeneity results:**
