# Supplementary material for: Effects of different training on lower limb explosive power in youth soccer players: a systematic review and network meta-analysis
Source: Front Physiol. 2026 Mar 19;17:1769079. doi: 10.3389/fphys.2026.1769079 (PMC13043373; doi:10.3389/fphys.2026.1769079)
Supplement: Supplementary file 2 [file Presentation1.zip › 附件/C1.docx]

**1.Inconsistency test of SJ outcome indicators**

**1.1** **Test results of node segmentation method:**

| Side | Direct |  | Indirect |  | Difference |  |  |
| --- | --- | --- | --- | --- | --- | --- | --- |
|  | Coef. | Std. Err. | Coef. | Std. Err. | Coef. | Std. Err. | P>z |
| A B | 2.78 | 2.777613 | 2.144668 | 3.028427 | 0.6353321 | 4.109319 | 0.877 |
| A C | 2.937069 | 1.884069 | 0.2881882 | 2.05151 | 2.648881 | 2.785117 | 0.342 |
| A D | . | . | . | . | . | . | . |
| A E | 1.938672 | 1.486477 | 3.081565 | 2.353486 | -1.142893 | 2.769178 | 0.68 |
| A F | 4.563692 | 1.503499 | 5.418825 | 1.971343 | -0.855133 | 2.475922 | 0.73 |
| B E | 0.0501811 | 2.716597 | -0.5832677 | 3.083835 | 0.6334488 | 4.10979 | 0.878 |
| C F | 4.006677 | 1.595869 | 1.358335 | 2.283677 | 2.648342 | 2.785754 | 0.342 |
| E F | 1.884045 | 1.92094 | 3.562078 | 2.191392 | -1.678033 | 2.912951 | 0.565 |

**1.2 Inconsistency model test results:**

|  | Coefficient | Std. err. | z | P>z | [95% conf. | interval] |
| --- | --- | --- | --- | --- | --- | --- |
|  |  |  |  |  |  |  |
| _y_B |  |  |  |  |  |  |
| _cons | 2.481938 | 1.921092 | 1.29 | 0.196 | -1.283334 | 6.24721 |
|  |  |  |  |  |  |  |
| _y_C |  |  |  |  |  |  |
| _cons | 1.726402 | 1.354869 | 1.27 | 0.203 | -0.9290918 | 4.381897 |
|  |  |  |  |  |  |  |
| _y_D |  |  |  |  |  |  |
| _cons | -3.95 | 4.246133 | -0.93 | 0.352 | -12.27227 | 4.372268 |
|  |  |  |  |  |  |  |
| _y_E |  |  |  |  |  |  |
| _cons | 2.249475 | 1.181516 | 1.9 | 0.057 | -0.0662536 | 4.565203 |
|  |  |  |  |  |  |  |
| _y_F |  |  |  |  |  |  |
| _cons | 4.858484 | 1.116509 | 4.35 | 0 | 2.670167 | 7.046801 |

**1.3 Results of ring inconsistency test:**

| Loop | IF | seIF | z_value | p_value | CI_95 | Loop_Heterog_tau2 | |
| --- | --- | --- | --- | --- | --- | --- | --- |
| Con-RT | CT | 2.453 | 3.941 | 0.622 | 0.534 | (0.00,10.18) | 6.587 |
| Con -OPL | JT | 0.912 | 2.396 | 0.381 | 0.703 | (0.00,5.61) | 0.482 |
| Con -JT | CT | 0.064 | 1.099 | 0.058 | 0.954 | (0.00,2.22) | 0.042 |

**1.4** **Overall inconsistency test results:**

| chi2( 5) | = | 0.84 |
| --- | --- | --- |
| Prob > chi2 | = | 0.9741 |

**2.** **Inconsistency testing of CMJ outcome measures**

**2.1** **Test results of node segmentation method:**

| Side | Direct |  | Indirect |  | Difference |  |  |
| --- | --- | --- | --- | --- | --- | --- | --- |
|  | Coef. | Std. Err. | Coef. | Std. Err. | Coef. | Std. Err. | P>z |
| A B | 2.797158 | 0.998808 | 2.52199 | 1.986267 | 0.2751688 | 2.215979 | 0.901 |
| A C | 1.683148 | 1.098524 | 1.524632 | 1.633557 | 0.1585152 | 1.967246 | 0.936 |
| A D | . | . | . | . | . | . | . |
| A E | 2.426929 | 0.8375178 | 0.7121947 | 1.350166 | 1.714734 | 1.561834 | 0.272 |
| A F | 3.380992 | 0.703642 | 3.16751 | 1.339445 | 0.2134823 | 1.511566 | 0.888 |
| B E | -1.712815 | 1.422661 | 0.1429704 | 1.410799 | -1.855786 | 2.008551 | 0.356 |
| C F | 1.791806 | 1.494013 | 1.634171 | 1.280336 | 0.1576348 | 1.967685 | 0.936 |
| E F | 1.487605 | 1.355051 | 1.283945 | 1.134905 | 0.2036604 | 1.761125 | 0.908 |

**2.2 Inconsistency model test results:**

|  | **Coefficient** | **Std. err.** | **z** | **P>z** | **[95% conf.** | **interval]** |
| --- | --- | --- | --- | --- | --- | --- |
|  |  |  |  |  |  |  |
| **_y_B** |  |  |  |  |  |  |
| **_cons** | 2.741646 | 0.8769697 | 3.13 | 0.002 | 1.022817 | 4.460475 |
|  |  |  |  |  |  |  |
| **_y_C** |  |  |  |  |  |  |
| **_cons** | 1.626344 | 0.893994 | 1.82 | 0.069 | -0.1258522 | 3.37854 |
|  |  |  |  |  |  |  |
| **_y_D** |  |  |  |  |  |  |
| **_cons** | -2.97 | 3.16401 | -0.94 | 0.348 | -9.171346 | 3.231346 |
|  |  |  |  |  |  |  |
| **_y_E** |  |  |  |  |  |  |
| **_cons** | 1.9654 | 0.7223827 | 2.72 | 0.007 | 0.5495558 | 3.381244 |
|  |  |  |  |  |  |  |
| **_y_F** |  |  |  |  |  |  |
| **_cons** | 3.332965 | 0.6045085 | 5.51 | 0 | 2.14815 | 4.51778 |

**2.3 Results of ring inconsistency test:**

| **Loop** | **IF** | **seIF** | **z_value** | **p_value** | **CI_95** | **Loop_Heterog_tau2** |
| --- | --- | --- | --- | --- | --- | --- |
| **Con-OPL-JT** | **0.825** | **2.178** | **0.379** | **0.705** | **(0.00,5.09)** | **1.758** |
| **Con-JT-CT** | **0.593** | **1.871** | **0.317** | **0.751** | **(0.00,4.26)** | **2.269** |
| **Con-RT-CT** | **0.025** | **2.337** | **0.011** | **0.991** | **(0.00,4.60)** | **3.477** |

**2.4** **Overall inconsistency test results:**

| **chi2( 7)** | **=** | 6.81 |
| --- | --- | --- |
| **Prob > chi2** | **=** | 0.4488 |
