# Supplementary material for: Effects of different training on lower limb explosive power in youth soccer players: a systematic review and network meta-analysis
Source: Front Physiol. 2026 Mar 19;17:1769079. doi: 10.3389/fphys.2026.1769079 (PMC13043373; doi:10.3389/fphys.2026.1769079)
Supplement: Supplementary file 2 [file Presentation1.zip › 附件/C2.docx]

Publication bias or small sample effect test

SJ：

CMJ：
