# Supplementary material for: Effects of different training on lower limb explosive power in youth soccer players: a systematic review and network meta-analysis
Source: Front Physiol. 2026 Mar 19;17:1769079. doi: 10.3389/fphys.2026.1769079 (PMC13043373; doi:10.3389/fphys.2026.1769079)
Supplement: Supplementary file 2 [file Presentation1.zip › 附件/Risk of bias and GRADE assessment/A1.pdf]

一、Pubmed：

RT:

Search: ((((((((((((((((((((((((((Resistance Training[MeSH Terms]) OR (Training, Resistance[Title/Abstract])) OR (Weight-Lifting Strengthening Program[Title/Abstract])) OR (Strengthening Programs, Weight-Lifting[Title/Abstract])) OR (Strengthening Program, Weight-Lifting[Title/Abstract])) OR (Weight Lifting Strengthening Program[Title/Abstract])) OR (Weight-Lifting Strengthening Programs[Title/Abstract])) OR (Exercise Programs, Weight-Lifting[Title/Abstract])) OR (Weight Lifting Exercise Program[Title/Abstract])) OR (Weight-Bearing Strengthening Program[Title/Abstract])) OR (Strengthening Programs, Weight-Bearing[Title/Abstract])) OR (Weight Bearing Strengthening Program[Title/Abstract])) OR (Weight-Bearing Exercise Program[Title/Abstract])) OR (Exercise Programs, Weight-Bearing[Title/Abstract])) AND (Athletes[MeSH Terms])) OR (Professional Athletes[Title/Abstract])) OR (Elite Athletes[Title/Abstract])) AND (Adolescent[MeSH Terms])) OR (Adolescents[Title/Abstract])) OR (Adolescence[Title/Abstract])) OR (Youth[Title/Abstract])) OR (Youths[Title/Abstract])) OR (Teens[Title/Abstract])) OR (Teen[Title/Abstract])) OR (Teenager[Title/Abstract])) AND (explosive power[Title/Abstract])) OR (lower limb explosive strength[Title/Abstract])

HIIT:

Search: ((((((((((((((((((((High-Intensity Interval Training[MeSH Terms]) OR (High Intensity Interval Training[Title/Abstract])) OR (High-Intensity Interval Trainings[Title/Abstract])) OR (Interval Training, High-Intensity[Title/Abstract])) OR (Interval Trainings, High-Intensity[Title/Abstract])) OR (Training, High-Intensity Interval[Title/Abstract])) OR (High-Intensity Intermittent Exercise[Title/Abstract])) AND (Adolescent[MeSH Terms])) OR (Adolescence[Title/Abstract])) OR (Youth[Title/Abstract])) OR (Teen[Title/Abstract])) OR (Teenager[Title/Abstract])) AND (Athletes[MeSH Terms])) OR (Athlete[Title/Abstract])) OR (Professional Athletes[Title/Abstract])) OR (Athlete, Professional[Title/Abstract])) OR (Professional Athlete[Title/Abstract])) OR (Elite Athletes[Title/Abstract])) OR (Athlete, Elite[Title/Abstract])) AND (lower limb explosive strength[Title/Abstract])) OR (lower body explosive strength[Title/Abstract])

二、Embase

HIIT:

'lower body explosive strength':ab,ti OR 'lower limb explosive strength':ab,ti OR 'jump':ab,ti AND 'high-intensity intermittent training':ab,ti OR 'high-intensity intermittent exercise':ab,ti OR 'hiit':ab,ti OR 'intermittent high-intensity training':ab,ti OR 'interval high-intensity training':ab,ti OR 'high intensity interval training':ab,ti

FCT：

'french contrast training':ab,ti OR 'french contrast method':ab,ti

三、EBSCO

(AB randomized controlled trial OR AB randomized OR AB placebo) AND (AB Resistance Training OR AB High Intensity Interval Training OR AB Plyometric Exercise OR AB French contrast training OR AB French contrast method OR AB HIIT) AND (AB lower body explosive strength OR AB lower limb explosive strength OR AB jump) AND (AB Adolescents OR AB Adolescence OR AB Teen OR AB Teenagers OR AB Youth OR AB Youths)
