# Supplementary material for: Effects of different training on lower limb explosive power in youth soccer players: a systematic review and network meta-analysis
Source: Front Physiol. 2026 Mar 19;17:1769079. doi: 10.3389/fphys.2026.1769079 (PMC13043373; doi:10.3389/fphys.2026.1769079)
Supplement: Supplementary file 2 [file Presentation1.zip › 附件/Risk of bias and GRADE assessment/A2.pdf]

THE LANCET

Supplementary appendix

This appendix formed part of the original submission and has been peer reviewed. We post it as supplied by the authors.

Supplement to: Cipriani A, Furukawa TA, Salanti G, et al. Comparative efficacy and acceptability of 21 antidepressant drugs for the acute treatment of adults with major depressive disorder: a systematic review and network meta-analysis. *Lancet* 2018; published online Feb 21. http://dx.doi.org/10.1016/S0140-6736(17)32802-7.

COMPARATIVE EFFICACY AND ACCEPTABILITY OF FIRST- AND SECOND- GENERATION ANTIDEPRESSANTS IN THE ACUTE TREATMENT OF MAJOR DEPRESSIVE DISORDER: A NETWORK META-ANALYSIS

Andrea Cipriani, Toshi A Furukawa, Georgia Salanti, Anna Chaimani, Lauren Z Atkinson, Yusuke Ogawa, Stefan Leucht, Henricus G Ruhe, Erick H Turner, Julian PT Higgins, Matthias Egger, Nozomi Takeshima, Yu Hayasaka, Hissei Imai, Kiyomi Shinohara, Aran Tajika, John PA Ioannidis, John R Geddes

Supplementary Appendix

Contents

Supplementary Appendix 1

Contents 2

1 Reference list of included studies 6

2 Characteristics of included studies 65

3 Risk of bias table of included studies 115

4 Definition of covariates 133

4.1 Sponsorship 133

4.2 Baseline severity 133

4.3 Novelty of the agent 133

4.4 Licensed and accepted dose ranges 133

4.5 Unpublished data 134

4.6 Risk of bias in the included studies 136

5 Heterogeneity parameter standard deviations estimated in various models 137

5.1 Primary outcomes 137

5.1.1 Response 137

5.1.2 Dropouts for any reason 137

5.2 Secondary outcomes 137

5.2.1 Efficacy continuous 137

5.2.2 Remission 137

5.2.3 Dropouts due to adverse events 137

6 Estimation of inconsistency: summary of results 138

7 Analyses of the all trials network (including placebo arms) 139

7.1 Results from pairwise meta-analyses 139

7.1.1 Response 140

7.1.2 Dropouts for any reason 141

7.1.3 Efficacy continuous 142

7.1.4 Remission 143

7.1.5 Dropouts due to adverse events 144

7.2 Results from network meta-analyses 145

7.2.1 Response 145

7.2.2 Dropouts for any reason ... 148
7.2.3 Efficacy continuous ... 150
7.2.4 Remission ... 152
7.2.5 Dropouts due to adverse events ... 154
7.3 Assessment of transitivity ... 156
7.4 Evaluation of inconsistency ... 157
7.4.1 Response ... 157
7.4.2 Dropouts for any reason ... 161
7.4.3 Efficacy continuous ... 166
7.4.4 Remission ... 170
7.4.5 Dropouts due to adverse events ... 174
7.5 Comparison-adjusted funnel plots (all drugs vs placebo) ... 179
7.5.1 Response ... 179
7.5.2 Dropouts for any reason ... 180
7.5.3 Efficacy continuous ... 180
7.5.4 Remission ... 181
7.5.5 Dropouts due to adverse events ... 181
7.6 Meta-regressions and sensitivity analyses ... 182
7.6.1 Statistical details for network meta-analysis and meta-regression models ... 182
7.6.2 Changes in heterogeneity after accounting for potential effect modifiers ... 183
7.6.3 Sensitivity analyses ... 184
7.6.4 Summary of significance in the regression coefficients from meta-regressions ... 187
7.6.5 Odds-ratios for response and dropout for any reason adjusted for covariates. ... 188
7.6.6 Summary of the findings from meta-regressions and sugroup analyses ... 195

8 Analyses of the head-to-head trials network ... 197
8.1 Results from network meta-analyses ... 197
8.1.1 Response ... 197
8.1.2 Dropouts for any reason ... 199
8.1.3 Efficacy continuous ... 202
8.1.4 Remission ... 204
8.1.5 Dropouts due to adverse events ... 206
8.2 Assessment of transitivity ... 208
8.3 Inconsistency ... 209
8.3.1 Response ... 209
8.3.2 Dropouts for any reason ... 212
8.3.3 Efficacy continuous ... 215

8.3.4 Remission ... 218
8.3.5 Dropouts due to adverse events ... 221
8.4 Comparison-adjusted funnel plots (active vs fluoxetine) ... **225**
8.4.1 Response ... 225
8.4.2 Dropouts for any reason ... 226
8.4.3 Efficacy continuous ... 226
8.4.4 Remission ... 227
8.4.5 Dropouts due to adverse events ... 227
8.5 Meta-regressions and sensitivity analyses ... **228**
8.5.1 Changes in heterogeneity after accounting for potential effect modifiers ... 228
8.5.2 Odds-ratios for response and dropout for any reason adjusted for covariates ... 228
9 GRADE for the primary outcomes ... **231**
9.1 Response ... **231**
9.1.1 Summary of study limitations of the included studies ... 231
9.1.2 Contribution of low or moderate RoB comparisons to each network estimate ... 231
9.1.3 Table of reasons for downgrading ... 237
9.2 Dropouts for any reason ... **250**
9.2.1 Summary of study limitations of the included studies ... 250
9.2.2 Contribution of low or moderate RoB comparisons to each network estimate ... 250
9.2.3 Table of reasons for downgrading ... 255
10 SUCRA and cumulative probability plots ... **266**
10.1 All trials ... **266**
10.1.1 Response ... 266
10.1.2 Dropouts for any reason ... 268
10.1.3 Efficacy as continuous outcome ... 270
10.1.4 Remission ... 272
10.1.5 Dropouts due to adverse events ... 274
10.2 Head-to-head trials ... **276**
10.2.1 Response ... 276
10.2.2 Dropouts for any reason ... 278
10.2.3 Efficacy as continuous outcome ... 280
10.2.4 Remission ... 282
10.2.5 Dropouts due to adverse eventss ... 284
11 Post hoc sensitivity analyses and meta-regression analyses (as requested by peer reviewers) ... **286**
11.1 Meta-regression using the mean age of participants as covariate ... **286**

11.2 Meta-regression using the sex distribution (percentage of females in each study) as covariate 286
11.3 Sensitivity analysis excluding studies including bipolar patients 287
11.4 Sensitivity analysis for study duration (8 weeks or less versus more than 8 weeks) 288
11.5 Sensitivity analysis for country (North America only vs other) 288
12 Changes to the protocol 289

1 *Reference list of included studies*

1. Aberg-Wistedt 2000

- Aberg-Wistedt A, Agren H, Ekselius L, Bengtsson F, Akerblad AC. Sertraline versus paroxetine in major depression: clinical outcome after six months of continuous therapy. J Clin Psychopharmacol. 2000;20(6):645-52.

2. Aguglia 1993

- Aguglia E, Casacchia M, Cassano GB, Faravelli C, Ferrari G, Giordano P, Pancheri P, Ravizza L, Trabucchi M, Bolino F, Scarpato A, Berardi D, Provenzano G, Brugnoli R, Rozzini R. Double-blind study of the efficacy and safety of sertraline versus fluoxetine in major depression. Int Clin Psychopharmacol. 1993;8(3):197-202.

3. AK1102365

- https://www.gsk-clinicalstudyregister.com/study/AK1102365#ps

4. Alexopoulos 2004 (poster SCT-MD-27)

- www.ncbi.nlm.nih.gov/pubmedhealth/PMH0016624/bin/app17c_et6.pdf

5. Allard 2004

- Allard P, Gram L, Timdahl K, Behnke K, Hanson M, Søgaard J. Efficacy and tolerability of venlafaxine in geriatric outpatients with major depression: a double-blind, randomised 6-month comparative trial with citalopram. Int J Geriatr Psychiatry. 2004;19(12):1123-30.

6. Altamura 1989b

- Altamura AC, Mauri MC, Rudas N, Carpiniello B, Montanini R, Perini M, Scapicchio PL, Hadjchristos C, Carucci G, Minervini M, D'Aloise A, Malinconico A, Aguglia E, De Vanna R, D'Aronco R. Clinical activity and tolerability of trazodone, mianserin, and amitriptyline in elderly subjects with major depression: a controlled multicenter trial. Clin Neuropharmacol. 1989;12 Suppl 1:S25-33; S34-7.

7. Alvarez 2012 (11492A, NCT00839423)

- Alvarez E, Perez V, Dragheim M, Loft H, Artigas F. A double-blind, randomized, placebo-controlled, active reference study of Lu AA21004 in patients with major depressive disorder. Int J Neuropsychopharmacol. 2012;15(5):589-600.
- https://clinicaltrials.gov/ct2/show/NCT00839423

8. Alves 1999

- Alves C, Cachola I, Brandao J. Efficacy and tolerability of venlafaxine and fluoxetine in outpatients with major depression. Primary Care Psychiatry. 1999;5, 57-63.

9. Amin 1984

- Amin MM, Ananth JV, Coleman BS, Darcourt G, Farkas T, Goldstein B, Lapierre YD, Paykel E, Wakelin JS. Fluvoxamine: antidepressant effects confirmed in a placebo-controlled international study. Clinical Neuropharmacology 1984;7(suppl 1):580-581.

10. Amini 2005

- Amini H, Aghayan S, Jalili SA, Akhondzadeh S, Yahyazadeh O, Pakravan-Nejad M. Comparison of mirtazapine and fluoxetine in the treatment of major depressive disorder: a double-blind, randomized trial. J Clin Pharmacy and Therapeutics. 2005;30(2):133-138.

11. Amsterdam 1986

- Amsterdam JD, Case WG, Csanalosi E, Singer M, Rickels K. A double-blind comparative trial of zimelidine, amitriptyline, and placebo in patients with mixed anxiety and depression. Pharmacopsychiatry. 1986 May;19(3):115-9.

12. Andersen 1986

- Danish University Antidepressant Group. Citalopram: clinical effect profile in comparison with clomipramine. A controlled multicenter study. Psychopharmacology. 1986;90(1): 131-138.

13. Ansseau 1989a

- Ansseau M, von Frenckell R, Mertens C, de Wilde J, Botte L, Devoitille JM, Evrard JL, De Nayer A, Darimont P, Dejaiffe G, Mirel J, Meurice E, Parent M, Couzinier JP, Demarez JP, Serre C. Controlled comparison of two doses of milnacipran (F 2207) and amitriptyline in major depressive inpatients. Psychopharmacology. 1989;98(2):163-8.

14. Ansseau 1989b

- Ansseau M, von Frenckell R, Papart P, Mertens C, de Wilde J, Botte L, Devoitille JM, Evrard JL, De Nayer, Couzinier JP, Demarez JP, Serre C. A Controlled comparison of milnacipran (F2207) 200 mg and amitriptyline in endogenous depressive inpatients. Human Psychopharmacology. 1989;4(3);221-227.

15. Ansseau 1991

- Ansseau M, von Frenckell R, Gérard MA, Mertens C, De Wilde J, Botte L, Devoitille JM, Evrard JL, De Nayer A, Darimont P, Mirel J, Troisfontaines B, Toussaint C, Couzinier JP, Demarez JP, Serre C. Interest of a loading dose of milnacipran in endogenous depressive inpatients. Comparison with the standard regimen and with fluvoxamine. Eur Neuropsychopharmacol. 1991;1(2):113-21.

16. Ansseau 1994a (MY-1005/BRL-029060/1/CPMS-112)

- Ansseau M, Gabriëls A, Loyens J, Bartholomé F, Evrard JL, De Nayer A, Linhart R, Wirtz J, Bruynooghe F, Surinx K, Clarysse H, Marganne R, Papart P. Controlled comparison of paroxetine and fluvoxamine in major depression. Human Psychopharmacology: Clinical and Experimental. 1994;9(5):329-336.
- https://www.gsk-clinicalstudyregister.com/study/29060/112?search=study&#rs

17. Ansseau 1994b

- Ansseau M, Papart P, Troisfontaines B, Bartholomé F, Bataille M, Charles G, Schittecatte M, Darimont P, Devoitille JM, De Wilde J, Dufrasne M, Gilson H, Evrard JL, De Nayer A, Krémer P, Mertens C, Serre C. Controlled comparison of milnacipran and fluoxetine in major depression. Psychopharmacology. 1994;114:131-7.

18. Ansseau 1994c

- Ansseau M, Darimont P, Lecoq A, De Nayer A, Evrard JL, Krémer P, Devoitille JM, Dierick M, Mertens C, Mesotten F, Mirel J, Troisfontaines B, Van Moffaert M, Vanbrabant E. Controlled comparison of nefazodone and amitriptyline in major depressive inpatients. Psychopharmacology. 1994;115(1-2):254-60.

19. Armitage 1997

- Armitage R, Yonkers K, Cole DBA, Rush, JA. A Multicenter, Double-Blind Comparison of the Effects of Nefazodone and Fluoxetine on Sleep Architecture and Quality of Sleep in Depressed Outpatients. J Clin Psychopharmacology. 1997;17(3)161-168.

20. Asnis 2013 (LVM-MD-01, NCT00969709)

- Asnis GM, Bose A, Gommoll CP, Chen C, Greenberg WM. Efficacy and safety of levomilnacipran sustained release 40 mg, 80 mg, or 120 mg in major depressive disorder: a phase 3, randomized, double-blind, placebo-controlled study. J Clin Psychiatry. 2013 Mar;74(3):242-8.
- https://clinicaltrials.gov/ct2/show/NCT00969709

21. Bakish 1992

- Bakish D, Bradwejn J, Nair N, McClure J, Remick R, Bulger L. A comparison of moclobemide, amitriptyline and placebo in depression: a Canadian multicentre study. Psychopharmacology (Berl). 1992;106 Suppl:S98-101.

22. Bakish 1997

- Bakish D, Cavazzonia P, Chudzika J, Ravindrana A, Hrdinaa PD. Effects of selective serotonin reuptake inhibitors on platelet serotonin parameters in major depressive disorder. Biological Psychiatry. 1997 Jan;41(2):184-190.

23. Bakish 2014 (LVM-MD-10, NCT01377194)

- Bakish D, Bose A, Gommoll C, Chen C, Nunez R, Greenberg WM, Liebowitz M, Khan A. Levomilnacipran ER 40 mg and 80 mg in patients with major depressive disorder: a phase III, randomized, double-blind, fixed-dose, placebo-controlled study. J Psychiatry Neurosci. 2014 Jan;39(1):40-9.
- https://clinicaltrials.gov/ct2/show/NCT01377194

24. Baldwin 1996

- Baldwin DS, Hawley CJ, Abed RT, Maragakis BP, Cox J, Buckingham SA, Pover GH, Ascher A. A multicenter double-blind comparison of nefazodone and paroxetine in the treatment of outpatients with moderate-to-severe depression. J Clin Psychiatry. 1996;57 Suppl 2:46-52.

25. Baldwin 2006a (97-CRBX-052 - Study 052)

- Baldwin D, Bridgman K, Buis C. Resolution of sexual dysfunction during double-blind treatment of major depression with reboxetine or paroxetine. J Psychopharmacol. 2006 Jan;20(1):91-6. Epub 2005 Oct 4.
- https://www.iqwig.de/download/Studie_052.pdf

26. Baldwin 2006b (Study 99505)

- Baldwin DS, Cooper JA, Huusom AK, Hindmarch I. A double-blind, randomized, parallel-group, flexible-dose study to evaluate the tolerability, efficacy and effects of treatment discontinuation with escitalopram and paroxetine in patients with major depressive disorder. Int Clin Psychopharmacol. 2006 May;21(3):159-69.
- http://www.lundbeck.com/upload/trials/files/pdf/completed/99505%20_14June2006_Final%20for%20upload_CTRS.pdf

27. Baldwin 2012 (11984A, NCT00635219)

- Baldwin DS, Loft H, Dragheim M. A randomised, double-blind, placebo controlled, duloxetine-referenced, fixed-dose study of three dosages of Lu AA21004 in acute treatment of major depressive disorder (MDD). Eur Neuropsychopharmacol. 2012 Jul;22(7):482-91.
- https://clinicaltrials.gov/ct2/show/NCT00635219

28. Ban 1998 (Study 008)

- Ban TA, Gaszner P, Aguglia E, Vergara L. Clinical Efficacy of Reboxetine: a Comparative Study with Desipramine, with Methodological Considerations. Human Psychopharmacology 1998;13: S29-S39.
- https://www.iqwig.de/de/projekte-ergebnisse/studieninformationen-zu-reboxetin.3304.html

29. Barber 2011

- Barber JP, Barrett MS, Gallop R, Rynn MA, Rickels K. Short-term dynamic psychotherapy versus pharmacotherapy for major depressive disorder: a randomized, placebo-controlled trial. J Clin Psychiatry. 2012 Jan;73(1):66-73.

30. Battegay 1985 (PAR 29060.309 HP/81/162A)

- Battegay R, Hager M, Rauchfleisch U. Double-blind comparative study of paroxetine and amitriptyline in depressed patients of a university psychiatric outpatient clinic (pilot study). Neuropsychobiology. 1985;13(1-2):31-7.
- https://www.gsk-clinicalstudyregister.com/study/29060/309?search=compound&compound=paroxetine#rs

31. Beasley 1991

- Beasley CM Jr, Dornseif BE, Pultz JA, Bosomworth JC, Sayler ME. Fluoxetine versus trazodone: efficacy and activating-sedating effects. J Clin Psychiatry. 1991 Jul;52:294-9.

32. Behnke 2003 (E-1690, Kjernisted2002)

- Behnke K, Søgaard J, Martin S, Bäuml J, Ravindran AV, Agren H, Vester-Blokland ED. Mirtazapine orally disintegrating tablet versus sertraline: a prospective onset of action study. J Clin Psychopharmacol. 2003 Aug;23(4):358-64.

33. Benkert 2000 (E-1559)

- Benkert O, Szegedi A, Kohnen R. Mirtazapine compared with paroxetine in major depression. J Clin Psychiatry. 2000 Sep;61(9):656-63.

34. Benkert 2006(C-1763)

- https://www.iqwig.de/download/A05-20C_Abschlussbericht_Bupropion_Mirtazapin_und_Reboxetin_bei_Depressionen.pdf

35. Bennie 1995

- Bennie EH, Mullin JM, Martindale JJ. A double-blind multicentre trial comparing sertraline and fluoxetine in outpatients with major depression. J Clin Psychiatry. 1995 Jun;56(6):229-37.

36. Berlanga 1997

- Berlanga C, Arechavaleta B, Heinze G, Campillo C, Torres M, Caballero A, Apiquián R, Castelli P. A double-blind comparison of nefazodone and fluoxetine in the treatment of depressed outpatients. Salud Mental. 1997;20(3):1-8.

37. Berlanga 2006

- Berlanga C, Flores-Ramos M. Different gender response to serotonergic and noradrenergic antidepressants. A comparative study of the efficacy of citalopram and reboxetine. J Affect Disord. 2006 Oct;95(1-3):119-23.

38. Bersani 1994

- Bersani G, Rapisarda V, Ciani N, Bertolino A, Sorge G. A double-blind comparative study of sertraline and amitriptyline in outpatients with major depressive episodes. Human Psychopharmacology: Clinical and Experimental. 1994;9(1):63-68.

39. Bhatia 1991

- Bhatia SC, Hsieh HH, Theesen KA, Townley RG, Andersen JM, Weiss S, Agrawal DK. Platelet alpha-2 adrenoreceptor activity pre-treatment and post-treatment in major depressive disorder with melancholia. Res Commun Chem Pathol Pharmacol. 1991 Oct;74(1):47-57.

40. Bielski 2004 (SCT-MD-12)

- Bielski RJ, Ventura D, Chang CC. A double-blind comparison of escitalopram and venlafaxine extended release in the treatment of major depressive disorder. J Clin Psychiatry. 2004 Sep;65(9):1190-6.

41. Bignamini 1992 (PAR 29060/078)

- Bignamini A, Rapisarda V. A double-blind multicentre study of paroxetine and amitriptyline in depressed outpatients. Italian Paroxetine Study Group. Int Clin Psychopharmacol. 1992 Jun;6 Suppl 4:37-41.
- https://www.gsk-clinicalstudyregister.com/study/29060/078#rs

42. Binnemann 2008 (NCT00143091)

- Binneman B, Feltner D, Kolluri S, Shi Y, Qiu R, Stiger T. A 6-week randomized, placebo-controlled trial of CP-316,311 (a selective CRH1 antagonist) in the treatment of major depression. Am J Psychiatry. 2008;165(5):617-20.

43. Bjerkenstedt 2005

- Bjerkenstedt L1, Edman GV, Alken RG, Mannel M. Hypericum extract LI 160 and fluoxetine in mild to moderate depression: a randomized, placebo-controlled multi-center study in outpatients. Eur Arch Psychiatry Clin Neurosci. 2005 Feb;255(1):40-7.

44. Blacker 1988

- Blacker R, Shanks NJ, Chapman N, Davey A. The drug treatment of depression in general practice: a comparison of nocte administration of trazodone with mianserin, dothiepin and amitriptyline. Psychopharmacology. 1988;95 Suppl:S18-24.

45. Bosc 1997a (Study 014 – Andreoli 2002)

- Andreoli V, Caillard V, Deo RS, Rybakowski JK, Versiani M. Reboxetine, a new noradrenaline selective antidepressant, is at least as effective as fluoxetine in the treatment of depression. J Clin Psychopharm 2002; 22: 393–99.

46. Bosc 1997b (Study 016 - Massana 1999)

- Massana J, Möller HJ, Burrows GD, Montenegro RM. Reboxetine: a double-blind comparison with fluoxetine in major depressive disorder. Int Clin Psychopharmacol. 1999 Mar;14(2):73-80.
- https://www.iqwig.de/download/Studienbericht_zu_Studie_016.pdf

47. Bose 2008 (SCT-MD-13)

- Bose A, Li D, Gandhi C. Escitalopram in the Acute Treatment of Depressed Patients Aged 60 Years or Older. Am J Geriatric Psychiatry. 2008 Jan;16(1):14-20.

48. Bougerol 1997 (Study 91302 - FDA)

- Bougerol T, Scotto JC, Patris M, Strut N, Lemming O, Petersen HEH. Citalopram and Fluoxetine in Major Depression. Comparison of Two Clinical Trials in a Psychiatrist Setting and in General Practice. Clin. Drug Investig. 1997;14(2):77-89.

49. Boulenger 2006 (Study 10351)

- Boulenger JP, Huusom AK, Florea I, Baekdal T, Sarchiapone M. A comparative study of the efficacy of long-term treatment with escitalopram and paroxetine in severely depressed patients. Curr Med Res Opin. 2006 Jul;22(7):1331-41.
- http://www.lundbeck.com/upload/trials/files/pdf/completed/10351_Final%20for%20upload_28Feb2007_CTRS.pdf

50. Boulenger 2014 (13267A, NCT01140906)

- Boulenger JP, Loft H, Olsen CK. Efficacy and safety of vortioxetine (Lu AA21004), 15 and 20 mg/day: a randomized, double-blind, placebo-controlled, duloxetine-referenced study in the acute treatment of adult patients with major depressive disorder. Int Clin Psychopharmacol. 2014;29(3):138-49.
- https://clinicaltrials.gov/ct2/show/NCT01140906

51. Boyer 2008 (Study 333, NCT00300378, EUCTR2005-005463-28, 3151A1-333-EU)

- Boyer P, Montgomery S, Lepola U, Germain JM, Brisard C, Ganguly R, Padmanabhan SK, Tourian KA. Efficacy, safety, and tolerability of fixed-dose desvenlafaxine 50 and 100 mg/day for major depressive disorder in a placebo-controlled trial. Int Clin Psychopharmacol. 2008;23(5):243-53.
- https://clinicaltrials.gov/ct2/show/NCT00300378

52. Bremner 1995 (MIR 003-022 - FDA)

- Bremner JD. A double-blind comparison of Org 3770, amitriptyline, and placebo in major depression. J Clin Psychiatry. 1995 Nov;56(11):519-25.

53. Brown 1986

- Brown WA, Arato M, Shrivastava R. Pituitary-adrenocortical hyperfunction and intolerance to fluvoxamine, a selective serotonin uptake inhibitor. Am J Psychiatry. 1986 Jan;143(1):88-90.

54. Brunoni 2012

- Brunoni AR, Valiengo L, Baccaro A, Zanão TA, de Oliveira JF, Goulart A, Boggio PS, Lotufo PA, Benseñor IM, Fregni F. The sertraline vs. electrical current therapy for treating depression clinical study: results from a factorial, randomized, controlled trial. JAMA Psychiatry. 2013;70(4):383-91.

55. Buchsbaum 1997

- Buchsbaum MS, Wu J, Siegel BV, Hackett E, Trenary M, Abel L, Reynolds C. Effect of sertraline on regional metabolic rate in patients with affective disorder. Biol Psychiatry. 1997 Jan 1;41(1):15-22.

56. Bueno 1997

- Bueno JR, Mattos P. Estudo duplo-cego comparativo da eficacia e seguranca da nefazodona e amitriptilina. J Bras Psiquiat. 1997;7(46):385-389.

57. Burke 2002 (SCT-MD-01)

- Burke WJ, Gergel I, Bose A. Fixed-dose trial of the single isomer SSRI escitalopram in depressed outpatients. J Clin Psychiatry. 2002 Apr;63(4):331-6

58. Byerley 1988

- Byerley WF, Reimherr FW, Wood DR, Grosser BI. Fluoxetine, a selective serotonin uptake inhibitor, for the treatment of outpatients with major depression. J Clin Psychopharmacol. 1988;8(2):112-5.

59. CAGO178A2303 (NCT00463242)

- https://clinicaltrials.gov/ct2/show/record/NCT00463242

60. Caligiuri 2003

- Caligiuri MP, Gentili V, Eberson S, Kelsoe J, Rapaport M, Gillin JC. A quantitative neuromotor predictor of antidepressant non-response in patients with major depression. J Affect Disord. 2003 Nov;77(2):135-41.

61. Carman 1991

- Carman JS, Ahdieh H, Wyatt-Knowles E, Warga E, Panagides J. A controlled study of mianserin in moderately to severely depressed outpatients. Psychopharmacol Bull. 1991;27(2):135-9.

62. Casabona 2002 (0600B 428)

- Casabona GM, Silenzi V, Guazzelli M. A randomized, double blind, comparison of venlafaxine ER and paroxetine in outpatients with moderate to severe major depression. Eur Neuropsychpharm. 2002;12(Supp3);208.

63. Cassano 1986

- Cassano GB, Conti L, Massimetti G, Mengali F, Waekelin JS, Levine J. Use of a standardized documentation system (BLIPS/BDP) in the conduct of a multicenter international trial comparing fluvoxamine, imipramine, and placebo. Psychopharmacol Bull. 1986;22(1):52-8.

64. Cassano 2002 (29060/421)

- Cassano GB, Puca F, Scapicchio PL, Trabucchi M; Italian Study Group on Depression in Elderly Patients. Paroxetine and fluoxetine effects on mood and cognitive functions in depressed nondemented elderly patients. J Clin Psychiatry. 2002 May;63(5):396-402.
- https://www.gsk-clinicalstudyregister.com/study/29060/421?search=compound&compound=paroxetine#rs

65. Chang 2015

- Chang HH, Chou CH, Yang YK, Lee IH, Chen PS. Association between ABCB1 Polymorphisms and Antidepressant Treatment Response in Taiwanese Major Depressive Patients. Clin Psychopharmacol Neurosci. 2015 Dec 31;13(3):250-5. doi: 10.9758/cpn.2015.13.3.250.

66. Chouinard 1983a

- Chouinard G. Bupropion and amitriptyline in the treatment of depressed patients. J Clin Psychiatry. 1983 May;44(5 Pt 2):121-9.

67. Chouinard 1985

- Chouinard G. A double-blind controlled clinical trial of fluoxetine and amitriptyline in the treatment of outpatients with major depressive disorder. J Clin Psychiatry. 1985 Mar;46(3 Pt 2):32-7.

68. Chouinard 1999 (29060/131)

- Chouinard G1, Saxena B, Bélanger MC, Ravindran A, Bakish D, Beauclair L, Morris P, Vasavan Nair NP, Manchanda R, Reesal R, Remick R, O'Neill MC. A Canadian multicenter, double-blind study of paroxetine and fluoxetine in major depressive disorder. J Affect Disord. 1999 Jul;54(1-2):39-48.
- https://www.gsk-clinicalstudyregister.com/study/29060/131#rs

69. Christiansen 1996

- Christiansen PE1, Behnke K, Black CH, Ohrström JK, Bork-Rasmussen H, Nilsson J. Paroxetine and amitriptyline in the treatment of depression in general practice. Acta Psychiatr Scand. 1996 Mar;93(3):158-63.

70. CL3-20098-022

- http://www.ema.europa.eu/docs/en_GB/document_library/EPAR_-_Public_assessment_report/human/000916/WC500038315.pdf

71. CL3-20098-023

- http://www.ema.europa.eu/docs/en_GB/document_library/EPAR_-_Public_assessment_report/human/000915/WC500046226.pdf

72. CL3-20098-024

- http://www.ema.europa.eu/docs/en_GB/document_library/EPAR_-_Public_assessment_report/human/000916/WC500038315.pdf

73. CL3-20098-026

- http://www.ema.europa.eu/docs/en_GB/document_library/EPAR_-_Public_assessment_report/human/000916/WC500038315.pdf

74. CL3-20098-036

- Unpublished data provided from Servier upon request.

75. CL3-20098-048

- https://www.clinicaltrialsregister.eu/ctr-search/search?query=eudract_number:2005-002388-95

76. CL3-20098-062

- https://www.clinicaltrialsregister.eu/ctr-search/search?query=2008-004642-92

77. CL3-20098-070

- https://www.clinicaltrialsregister.eu/ctr-search/trial/2009-011795-29/FI

78. Claghorn 1983

- Claghorn J, Gershon S, Goldstein BJ. Zimeldine tolerability in comparison to amitriptyline and placebo. Acta Psychiatr Scand Suppl. 1983;308:104-14.

79. Claghorn 1995 (MIR 003-002 (FDA)

- Claghorn JL, Lesem MD. A double-blind placebo-controlled study of Org 3770 in depressed outpatients. J Affect Disord. 1995 Jun 8;34(3):165-71.

80. Claghorn 1996

- Claghorn JL, Earl CQ, Walczak DD, Stoner KA, Wong LF, Kanter D, Houser VP. Fluvoxamine maleate in the treatment of depression: a single-center, double-blind, placebo-controlled comparison with imipramine in outpatients. J Clin Psychopharmacol. 1996 Apr;16(2):113-20.

81. Clayton 2003 (Study 050)

- Clayton AH, Zajecka J, Ferguson JM, Filipiak-Reisner JK, Brown MT, Schwartz GE. Lack of sexual dysfunction with the selective noradrenaline reuptake inhibitor reboxetine during treatment for major depressive disorder. Int Clin Psychopharmacol. 2003;18(3):151-6.
- https://www.iqwig.de/de/projekte-ergebnisse/studieninformationen-zu-reboxetin.3304.html

82. Clayton 2006a (WELL AK130926)

- Clayton AH, Croft H, Horrigan JP, et al. Bupropion XL compared with escitalopram: effects on sexual functioning and antidepressant efficacy in two randomized, double-blind, placebo controlled studies. J Clin Psychiatry 2006; 67: 736–46.
- https://www.gsk-clinicalstudyregister.com/files2/20466.pdf

83. Clayton 2006b (WELL AK130927)

- Clayton AH, Croft H, Horrigan JP, et al. Bupropion XL compared with escitalopram: effects on sexual functioning and antidepressant efficacy in two randomized, double-blind, placebo controlled studies. J Clin Psychiatry 2006; 67: 736–46.
- https://www.gsk-clinicalstudyregister.com/files2/20467.pdf

84. Clayton 2013 (NCT01121484)

- Clayton AH, Kornstein SG, Dunlop BW, Focht K, Musgnung J, Ramey T, Bao W, Ninan PT. Efficacy and safety of desvenlafaxine 50 mg/d in a randomized, placebo-controlled study of perimenopausal and postmenopausal women with major depressive disorder. J Clin Psychiatry. 2013 Oct;74(10):1010-7.
- https://clinicaltrials.gov/ct2/show/NCT01121484

85. Clayton 2015 (NCT01432457)

- Clayton AH, Tourian KA, Focht K, Hwang E, Cheng RF, Thase ME. Desvenlafaxine 50 and 100 mg/d versus placebo for the treatment of major depressive disorder: a phase 4, randomized controlled trial. J Clin Psychiatry. 2015 May;76(5):562-9.
- https://clinicaltrials.gov/show/NCT01432457

86. Clerc 1994

- Clerc GE, Ruimy P, Verdeau-Pallès J. A double-blind comparison of venlafaxine and fluoxetine in patients hospitalized for major depression and melancholia. The Venlafaxine French Inpatient Study Group. Int Clin Psychopharmacol. 1994 Sep;9(3):139-43.

87. Clerc 2001

- Clerc G; Milnacipran/Fluvoxamine Study Group. Antidepressant efficacy and tolerability of milnacipran, a dual serotonin and noradrenaline reuptake inhibitor: a comparison with fluvoxamine. Int Clin Psychopharmacol. 2001 May;16(3):145-51.

88. CN104-002 (FDA)

- http://digitalcommons.ohsu.edu/fdadrug/23/

89. CN104-045 (FDA)

- http://digitalcommons.ohsu.edu/fdadrug/23/

90. CN104-054 (FDA)

- http://digitalcommons.ohsu.edu/fdadrug/23/

91. Cohn 1985a

- Cohn JB, Wilcox C. A comparison of fluoxetine, imipramine, and placebo in patients with major depressive disorder. J Clin Psychiatry. 1985 Mar;46(3 Pt 2):26-31.

92. Cohn 1990

- Cohn CK, Shrivastava R, Mendels J, Cohn JB, Fabre LF, Claghorn JL, Dessain EC, Itil TM, Lautin A. Double-blind, multicenter comparison of sertraline and amitriptyline in elderly depressed patients. J Clin Psychiatry. 1990 Dec;51 Suppl B:28-33.

93. Cohn 1996 (FDA CN104-006-2)

- Cohn CK, Robinson DS, Roberts DL, Schwiderski UE, O'Brien K, Ieni JR. Responders to antidepressant drug treatment: a study comparing nefazodone, imipramine, and placebo in patients with major depression. J Clin Psychiatry. 1996;57 Suppl 2:15-8.
- http://digitalcommons.ohsu.edu/fdadrug/23/

94. Coleman 1999 (AK1A4002)

- Coleman CC, Cunningham LA, Foster VJ, et al. Sexual dysfunction associated with the treatment of depression: a placebo-controlled comparison of bupropion sustained release and sertraline treatment. Ann Clin Psychiatry 1999; 11: 205–15.
- http://www.gsk-clinicalstudyregister.com/study/AK1A4002#rs

95. Coleman 2001 (AK1A4007)

- Coleman CC, King BR, Bolden-Watson C, et al. A placebo-controlled comparison of the eff ects on sexual functioning of bupropion sustained release and fluoxetine. Clin Ther 2001; 23: 1040–58.
- http://www.gsk-clinicalstudyregister.com/study/AK1A4007#rs

96. Colonna 2005 (Study 99022)

- Colonna L, Andersen HF, Reines EH. A randomized, double-blind, 24-week study of escitalopram (10 mg/day) versus citalopram (20 mg/day) in primary care patients with major depressive disorder. Curr Med Res Opin. 2005 Oct;21(10):1659-68.
- http://www.lundbeck.com/upload/trials/files/pdf/completed/99022_CTRS_final_30Dec2005.pdf

97. Corrigan 2000

- Corrigan MH, Denahan AQ, Wright CE, Ragual RJ, Evans DL. Comparison of pramipexole, fluoxetine, and placebo in patients with major depression. Depress Anxiety 2000;11(2):58-65.

98. Corruble 2013 (CL3-20098-063)

- Corruble E, de Bodinat C, Belaïdi C, Goodwin GM; agomelatine study group. Efficacy of agomelatine and escitalopram on depression, subjective sleep and emotional experiences in patients with major depressive disorder: a 24-wk randomized, controlled, double-blind trial. Int J Neuropsychopharmacol. 2013 Nov;16(10):2219-34.
- https://www.clinicaltrialsregister.eu/ctr-search/trial/2006-006540-54/results

99. Costaesilva 1998

- Costa e Silva J. Randomized, double-blind comparison of venlafaxine and fluoxetine in outpatients with major depression. J Clin Psychiatry. 1998 Jul;59(7):352-7.

100. Croft 1999 (AK1A4001)

- Croft H, Settle E Jr, Houser T, Batey SR, Donahue RMJ, Ascher JA. A placebo-controlled comparison of the antidepressant efficacy and effects on sexual functioning of sustained-release bupropion and sertraline. Clin Ther 1999; 21: 643–58.
- http://www.gsk-clinicalstudyregister.com/study/AK1A4001#rs

101. Croft 2014 (NCT01473394)

- Croft HA, Pomara N, Gommoll C, Chen D, Nunez R, Mathews M. Efficacy and safety of vilazodone in major depressive disorder: a randomized, double-blind, placebo-controlled trial. J Clin Psychiatry. 2014 Nov;75(11):e1291-8.
- https://clinicaltrials.gov/ct2/show/NCT01473394

102. Cunningham 1994 (VEN 600A-302 FDA)

- Cunningham LA, Borison RL, Carman JS, Chouinard G, Crowder JE, Diamond BI, Fischer DE, Hearst E. A comparison of venlafaxine, trazodone, and placebo in major depression. J Clin Psychopharmacol. 1994 Apr;14(2):99-106.
- http://digitalcommons.ohsu.edu/fdadrug/24/

103. Cunningham 1997 (VEN XR 208 - FDA)

- Cunningham LA. Once-daily venlafaxine extended release (XR) and venlafaxine immediate release (IR) in outpatients with major depression. Venlafaxine XR 208 Study Group. Ann Clin Psychiatry. 1997 Sep;9(3):157-64.
- http://digitalcommons.ohsu.edu/fdadrug/25/

104. Cutler 2009

- Cutler AJ, Montgomery SA, Feifel D, Lazarus A, Aström M, Brecher M. Extended release quetiapine fumarate monotherapy in major depressive disorder: a placebo- and duloxetine-controlled study. J Clin Psychiatry. 2009 Apr;70(4):526-39.

105. Dalery 2003

- Dalery J, Honig A. Fluvoxamine versus fluoxetine in major depressive episode: a double-blind randomised comparison. Hum Psychopharmacol. 2003 Jul;18(5):379-84.

106. D'Amico 1990 (FDA 030-A2-0007)

- D'Amico MF, Roberts DL, Robinson DS, Schwiderski UE, Copp J. Placebo-controlled dose-ranging trial designs in phase II development of nefazodone. Psychopharmacol Bull. 1990;26(1):147-50.
- http://digitalcommons.ohsu.edu/fdadrug/23/

107. Davidson 2002 (HDTSG, NCT00005013)

- Hypericum Depression Trial Study Group. Effect of Hypericum perforatum (St John's wort) in major depressive disorder: a randomized controlled trial. JAMA. 2002 Apr 10;287(14):1807-14.
- https://www.clinicaltrials.gov/ct2/show/NCT00005013

108. Debonnel 2000 (Blier2009, iQWIG E-1639)

- Blier P, Gobbi G, Turcotte JE, de Montigny C, Boucher N, Hébert C, Debonnel G. Mirtazapine and paroxetine in major depression: a comparison of monotherapy versus their combination from treatment initiation. Eur Neuropsychopharmacol. 2009 Jul;19(7):457-65.
- https://www.iqwig.de/download/A05-20C_Abschlussbericht_1-1_Bupropion_Mirtazapin_und_Reboxetin_bei_Depressionen.pdf

109. Debus 1988

- Debus JR, Rush AJ, Himmel C, et al. Fluoxetine versus trazodone in the treatment of outpatients with major depression. J Clin Psychiatry 1988;49:422-6.

110. DeMartinis 2007 (Study 306, NCT00072774)

- DeMartinis NA, Yeung PP, Entsuah R, Manley AL. A double-blind, placebo-controlled study of the efficacy and safety of desvenlafaxine succinate in the treatment of major depressive disorder. J Clin Psychiatry. 2007 May;68(5):677-88.
- http://www.accessdata.fda.gov/drugsatfda_docs/nda/2008/021992s000TOC.cfm
- https://clinicaltrials.gov/ct2/show/NCT00072774

111. Demyttenaere 1998

- Demyttenaere K, Van Ganse E, Gregoire J, Gaens E, Mesters P. Compliance in depressed patients treated with fluoxetine or amitriptyline. Belgian Compliance Study Group. Int Clin Psychopharmacol. 1998 Jan;13(1):11-7.

112. DeRonchi 1998

- De Ronchi D, Rucci P, Lodi M, Ravaglia G, Forti P, Volterra V. Fluoxetine and amitriptyline in elderly depressed patients. A 10-week, double-blind study on course of neurocognitive adverse events and depressive symptoms. Arch. Gerontology and Geriatrics. 1998;26(Supp 1):125-140.

113. Detke 2002a (HMBH - Study Group A)

- Detke MJ, Lu Y, Goldstein DJ, Hayes JR, Demitrack MA. Duloxetine, 60 mg once daily, for major depressive disorder: a randomized double-blind placebo-controlled trial. J Clin Psychiatry. 2002 Apr;63(4):308-15.

114. Detke 2002b (HMBH - Study Group B)

- Detke MJ, Lu Y, Goldstein D, McNamara R, Demitrack M. Duloxetine 60 mg once daily dosing versus placebo in the acute treatment of major depression. J Psychiatr Res 2002;36:383-90.

115. Detke 2004 (HMAY Study Group A)

- Detke MJ, Wiltse CG, Mallinckrodt CH, McNamara RK, Demitrack MA, Bitter I. Duloxetine in the acute and long-term treatment of major depressive disorder: a placebo- and paroxetine-controlled trial. Eur Neuropsychopharmacol 2004; 14: 457–70.
- http://www.lillytrials.com/results/Cymbalta.pdf

116. Deushle 2003 (NCT01049347)

- Deuschle M, Hamann B, Meichel C, Krumm B, Lederbogen F, Kniest A, Colla M, Heuser I. Antidepressive treatment with amitriptyline and paroxetine: effects on saliva cortisol concentrations. J Clin Psychopharmacol. 2003 Apr;23(2):201-5.
- https://clinicaltrials.gov/ct2/show/study/NCT01049347

117. DeWilde 1983a

- De Wilde JE, Mertens C, Wakelin JS. Clinical trials of fluvoxamine vs chlorimipramine with single and three times daily dosing. Br J Clin Pharmacol. 1983;15 Suppl 3:427S-431S.

118. DeWilde 1993

- De Wilde J, Spiers R, Mertens C, Bartholomé F, Schotte G, Leyman S. A double-blind, comparative, multicentre study comparing paroxetine with fluoxetine in depressed patients. Acta Psychiatr Scand. 1993 Feb;87(2):141-5.
- https://www.gsk-clinicalstudyregister.com/files2/1992.pdf

119. Dierick 1996

- Dierick M, Ravizza L, Realini R, Martin A. A double-blind comparison of venlafaxine and fluoxetine for treatment of major depression in outpatients. Prog Neuropsychopharmacol Biol Psychiatry. 1996 Jan;20(1):57-71.

120. Dimidjian 2006

- Dimidjian S, Hollon SD, Dobson KS, Schmaling KB, Kohlenberg RJ, Addis ME, Gallop R, McGlinchey JB, Markley DK, Gollan JK, Atkins DC, Dunner DL, Jacobson NS. Randomized trial of behavioral activation, cognitive therapy, and antidepressant medication in the acute treatment of adults with major depression. J Consult Clin Psychol. 2006 Aug;74(4):658-70.

121. Dominguez 1985

- Dominguez RA, Goldstein BJ, Jacobson AF, Steinbook RM. A double-blind placebo-controlled study of fluvoxamine and imipramine in depression. J Clin Psychiatry. 1985;46(3):84-7.

122. Doogan 1994

- Doogan D, Langdon C. Double-blind, placebo-controlled comparison of sertraline and dothiepin in treatment of major depression in general practice. Int Clin Psychopharmacol 1994;9:95-100.

123. DUAG 1990

- Danish University Antidepressant Group. Paroxetine: a selective serotonin reuptake inhibitor showing better tolerance, but weaker antidepressant effect than clomipramine in a controlled multicenter study. J Affect Disord. 1990 Apr;18(4):289-99.

124. Dube 2010 (NCT00420004)

- Dubé S, Dellva MA, Jones M, Kielbasa W, Padich R, Saha A, Rao P. A study of the effects of LY2216684, a selective norepinephrine reuptake inhibitor, in the treatment of major depression. J Psychiatr Res. 2010 Apr;44(6):356-63
- https://clinicaltrials.gov/ct2/show/NCT00420004

125. Dunbar 1993a (Claghorn1992, Rickels1989, Rickels1992, PAR 02-001 - FDA)

- Claghorn JL. The safety and efficacy of paroxetine compared with placebo in a double-blind trial of depressed outpatients. J Clin Psychiatry. 1992 Feb;53 Suppl:33-5.
- http://digitalcommons.ohsu.edu/fdadrug/26/

126. Dunbar 1993b (Claghorn1992, PAR 02-002 - FDA)

- Dunbar GC, Claghorn JL, Kiev A, Rickels K, Smith WT. A comparison of paroxetine and placebo in depressed outpatients. Acta Psychiatr Scand. 1993;87(5):302-5
- http://digitalcommons.ohsu.edu/fdadrug/26/

127. Dunbar 1993c (Smith1992, PAR 02-003 - FDA)

- Smith WT, Glaudin V. A placebo-controlled trial of paroxetine in the treatment of major depression. J Clin Psychiatry. 1992;53 Suppl:36-9.
- http://digitalcommons.ohsu.edu/fdadrug/26/

128. Dunbar 1993d (Kiev1992, PAR 02-004 - FDA)

- Kiev A. A double-blind, placebo-controlled study of paroxetine in depressed outpatients. J Clin Psychiatry. 1992;53 Suppl:27-9.
- http://digitalcommons.ohsu.edu/fdadrug/26/

129. Dunlop 2011 (NCT00824291)

- Dunlop BW, Reddy S, Yang L, Lubaczewski S, Focht K, Guico-Pabia CJ. Symptomatic and functional improvement in employed depressed patients: a double-blind clinical trial of desvenlafaxine versus placebo. J Clin Psychopharmacol. 2011 Oct;31(5):569-76.
- https://clinicaltrials.gov/ct2/show/NCT00824291

130. Dunner 1992 (PAR 29060.09)

- Dunner DL, Dunbar GC. Optimal dose regimen for paroxetine. J Clin Psychiatry. 1992;53 Suppl:21-6.
- http://www.gsk-clinicalstudyregister.com/study/29060/009#rs

131. E-1569

- https://www.iqwig.de/download/A05-20C_Abschlussbericht_1-1_Bupropion_Mirtazapin_und_Reboxetin_bei_Depressionen.pdf

132. Edwards 1989 (MD/PAR/009 PAR-276)

- Edwards JG, Goldie A. Placebo-controlled trial of paroxetine in depressive illness. Human Psychopharmacology 1993;8:203-209
- http://www.gsk-clinicalstudyregister.com/study/29060/276?search=compound&compound=paroxetine#rs

133. Ekselius 1997

- Ekselius L, von Knorring L. Personality disorder comorbidity with major depression and response to treatment with sertraline or citalopram. Int Clin Psychopharmacol. 1998 Sep;13(5):205-11.

134. Faber 1995 (SER 103 FDA)

- Fabre L, Abuzzahab F, Amin M, Claghorn J, Mendels J, Dubé S, Small JG. Sertraline in major depression: double-blind comparison with placebo. Biol Psychiatry 1995;38:592-602.
- http://digitalcommons.ohsu.edu/fdadrug/27/

135. Fabre 1979

- Fabre LF, McLendon DM, Gainey A. Trazodone efficacy in depression: a double-blind comparison with imipramine and placebo in day-hospital type patients. Current Therapeutic research 1979;25:827-834

136. Fabre 1996

- Fabre L, Birkhimer L, Zaborny B, Wong L, Kapik B. Fluvoxamine vs imipramine and placebo: a double-blind comparison in depressed patients. Int Clin Psychopharmacol 1996;11:119-27.

137. Falk 1989

- Falk WE, Rosenbaum JF, Otto MW, Zusky PM, Weilburg JB, Nixon RA. Fluoxetine versus trazodone in depressed geriatric patients. J Geriatr Psychiatry Neurol. 1989 Oct-Dec;2(4):208-14.

138. Fang 1997 (Yu 1997)

- Fang Y, Wang Z, Sheng J, Xie B, Da Z, Gao Z, Shi Q. Fluoxetine vs amitriptyline in treating 105 patients with depressive disorder with a double-blind study. New Drugs and Clinical Remedies 1997 July 16(4):254-256. (in Chinese)
- Yu B, Zhou R, Zhang F. Fluoxetine and amitriptyline for treating 22 patients of depressive disorder: a double blind controlled study. New Drugs and Clinical Remedies 1997 16(4):J23-J24.

139. Fava 1998

- Fava M, Amsterdam JD, Deltito JA. A double-blind study of paroxetine, fluoxetine, and placebo in outpatients with major depression. Ann Clin Psychiatry 1998; 10: 145–50.

140. Fava 2002

- Fava M, Hoog SL, Judge RA, Kopp JB, Nilsson ME, Gonzales JS. Acute efficacy of fluoxetine versus sertraline and paroxetine in major depressive disorder including effects of baseline insomnia. J Clin Psychopharmacol. 2002 Apr;22(2):137-47.

141. Fava 2005

- Fava M, Alpert J, Nierenberg AA, Mischoulon D, Otto MW, Zajecka J, Murck H, Rosenbaum JF. A Double-blind, randomized trial of St John's wort, fluoxetine, and placebo in major depressive disorder. J Clin Psychopharmacol. 2005 Oct;25(5):441-7.

142. Fawcett 1989

- Fawcett J, Zajecka JM, Kravitz HM, Edwards J, Jeffriess H, Scorza E. Fluoxetine versus amitriptyline in adult outpatients with major depression. Current Therapeutic Research - Clinical and Experimental. 1989 Jan 1;45(5):821-832.

143. Feiger 1996

- Feiger A, Kiev A, Shrivastava RK, Wisselink PG, Wilcox CS. Nefazodone versus sertraline in outpatients with major depression: focus on efficacy, tolerability, and effects on sexual function and satisfaction. J Clin Psychiatry. 1996;57 Suppl 2:53-62.

144. Feighner 1979

- Feighner JP, Brauzer B, Gelenberg AJ, Gomez E, Kiev A, Kurland ML, Weiss BL. A placebo-controlled multicenter trial of Limbitrol versus its components (amitriptyline and chlordiazepoxide) in the symptomatic treatment of depressive illness. Psychopharmacology (Berl). 1979 Mar 22;61(2):217-25.

145. Feighner 1980

- Feighner JP. Trazodone, a triazolopyridine derivative, in primary depressive disorder. J Clin Psychiatry. 1980;41(7):250-5.

146. Feighner 1984

- Feighner JP, Meredith CH, Stern WC, Hendrickson G, Miller LL. A double-blind study of bupropion and placebo in depression. Am J Psychiatry. 1984;141(4):525-9.

147. Feighner 1989a

- Feighner JP, Boyer WF, Merideth CH, Hendrickson GG. A double-blind comparison of fluoxetine, imipramine and placebo in outpatients with major depression. Int Clin Psychopharmacol. 1989 Apr;4(2):127-34.

148. Feighner 1989b

- Feighner JP, Boyer WF, Meredith CH, Hendrickson GG. A placebo-controlled inpatient comparison of fluvoxamine maleate and imipramine in major depression. Int Clin Psychopharmacol. 1989 Jul;4(3):239-44.

149. Feighner 1991

- Feighner JP, Gardner EA, Johnston JA, Batey SR, Khayrallah MA, Ascher JA, Lineberry CG. Double-blind comparison of bupropion and fluoxetine in depressed outpatients. J Clin Psychiatry. 1991 Aug;52(8):329-35.

150. Feighner 1993a (Feighner 1989c, PAR 03 001 - FDA)

- Feighner JP, Boyer WF. Paroxetine in the treatment of depression: a comparison with imipramine and placebo. Acta Psychiatr Scand Suppl. 1989;350:125-9.
- http://digitalcommons.ohsu.edu/fdadrug/26/

151. Feighner 1993b (Cohn1990, Cohn1992, PAR 03 002 - FDA)

- Cohn JB, Wilcox CS. Paroxetine in major depression: a double-blind trial with imipramine and placebo. J Clin Psychiatry. 1992 Feb;53 Suppl:52-6.
- http://digitalcommons.ohsu.edu/fdadrug/26/

152. Feighner 1993c (PAR 03 003 - FDA)

- Dunbar GC, Cohn JB, Fabre LF, Feighner JP, Fieve RR, Mendels J, Shrivastava RK. A comparison of paroxetine, imipramine and placebo in depressed out-patients. Br J Psychiatry. 1991 Sep;159:394-8
- http://digitalcommons.ohsu.edu/fdadrug/26/

153. Feighner 1993d (Shrivastava 1992, PAR 03 004 - FDA)

- Shrivastava RK, Shrivastava SH, Overweg N, Blumhardt CL. A double-blind comparison of paroxetine, imipramine and placebo in major depression. J Clin Psychiatry 1992;53 Suppl:48-51.
- http://digitalcommons.ohsu.edu/fdadrug/26/

154. Feighner 1993e (PAR 03 005 - FDA)

- Feighner JP. Cohn JB. Fabre LF Jr. Fieve RR. Mendels J. Shrivastava RK. Dunbar GC. A study comparing paroxetine placebo and imipramine in depressed patients. Journal of Affective Disorders. 28(2):71-79, 1993.
- http://digitalcommons.ohsu.edu/fdadrug/26/

155. Feighner 1993f (Fabre 1992, PAR 03 006 - FDA)

- Fabre LF. A 6-week, double-blind trial of paroxetine, imipramine, and placebo in depressed outpatients. J Clin Psychiatry. 1992;53 Suppl:40-3.
- http://digitalcommons.ohsu.edu/fdadrug/26/

156. Feighner 1998

- Feighner J, Targum SD, Bennett ME, Roberts DL, Kensler TT, D'Amico MF, Hardy SA. A double-blind, placebo-controlled trial of nefazodone in the treatment of patients hospitalized for major depression. J Clin Psychiatry. 1998 May;59(5):246-53.

157. Feighner 1999 (Study 91206 FDA)

- Feighner JP1, Overø K. Multicenter, placebo-controlled, fixed-dose study of citalopram in moderate-to-severe depression. J Clin Psychiatry. 1999 Dec;60(12):824-30.

158. Fontaine 1994 (FDA 03A0A-003)

- Fontaine R, Ontiveros A, Elie R, Kensler TT, Roberts DL, Kaplita S, Ecker JA, Faludi G. A double-blind comparison of nefazodone, imipramine, and placebo in major depression. J Clin Psychiatry. 1994;55(6):234-41.
- http://digitalcommons.ohsu.edu/fdadrug/23/

159. Frank 2004

- Frank MG, Hendricks SE, Burke WJ, Johnson DR. Clinical response augments NK cell activity independent of treatment modality: a randomized double-blind placebo controlled antidepressant trial. Psychol Med. 2004 Apr;34(3):491-8.

160. Fudge 1990

- Fudge JL, Perry PJ, Garvey MJ, Kelly MW. A comparison of the effect of fluoxetine and trazodone on the cognitive functioning of depressed outpatients. J Affect Disord. 1990 Apr;18(4):275-80.

161. Gagiano 1993

- Gagiano C. A double-blind comparison of paroxetine and fluoxetine in patients with major depression. Br J Clin Res. 1993;4:145-152.
- https://www.gsk-clinicalstudyregister.com/files2/2783.pdf

162. Gastpar 2006

- Gastpar M, Singer A, Zeller K. Comparative efficacy and safety of a once-daily dosage of hypericum extract STW3-VI and citalopram in patients with moderate depression: a double-blind, randomised, multicentre, placebo-controlled study. Pharmacopsychiatry. 2006 Mar;39(2):66-75.

163. Gelenberg 1990

- Gelenberg AJ, Wojcik JD, Falk WE, Spring B, Brotman AW, Galvin-Nadeau M. Clovoxamine in the treatment of depressed outpatients: a double-blind, parallel-group comparison against amitriptyline and placebo. Compr Psychiatry. 1990 Jul-Aug;31(4):307-14.

164. Gentil 2000

- Gentil V, Kerr-Correa F, Moreno R, D'Arrigo Busnello E, De Campos JA, Juruena MF, Lafer B, Moreno DH, De Cassia Rodrigues RL, Tiosso A, Benedictis E. Double-blind comparison of venlafaxine and amitriptyline in outpatients with major depression with or without melancholia. J Psychopharmacol. 2000 Mar;14(1):61-6.

165. Georgotas 1982

- Georgotas A, Krakowski M, Gershon S. Controlled trial of zimelidine, a 5-HT reuptake inhibitor, for treatment of depression. Am J Psychiatry. 1982 Aug;139(8):1057-8.

166. Geretsegger 1995 (MY-060/BRL-029060/1)

- Geretsegger C, Stuppaeck CH, Mair M, Platz T, Fartacek R, Heim M. Multicenter double blind study of paroxetine and amitriptyline in elderly depressed inpatients. Psychopharmacology (Berl). 1995 Jun;119(3):277-81.
- https://www.gsk-clinicalstudyregister.com/study/29060/073?search=study&#rs

167. Gerner 1980

- Gerner R, Estabrook W, Steuer J, Jarvik L. Treatment of geriatric depression with trazodone, imipramine, and placebo: a double-blind study. J Clin Psychiatry. 1980 Jun;41(6):216-20.

168. Gillin 1997

- Gillin JC, Rapaport M, Erman MK, Winokur A, Albala BJ. A comparison of nefazodone and fluoxetine on mood and on objective, subjective, and clinician-rated measures of sleep in depressed patients: a double-blind, 8-week clinical trial. J Clin Psychiatry. 1997 May;58(5):185-92.

169. Ginestet 1989

- Ginestet D. Fluoxetine in endogenous depression and melancholia versus clomipramine. Int Clin Psychopharmacol. 1989 Jan;4 Suppl 1:37-40.

170. Golden 2002a (29060/448)

- Golden RN, Nemeroff CB, McSorley P, Pitts CD, Dubé EM. Efficacy and tolerability of controlled-release and immediate-release paroxetine in the treatment of depression. J Clin Psychiatry. 2002 Jul;63(7):577-84.
- http://www.gsk-clinicalstudyregister.com/study/29060/448#rs

171. Golden 2002b (29060/449)

- Golden RN, Nemeroff CB, McSorley P, Pitts CD, Dubé EM. Efficacy and tolerability of controlled-release and immediate-release paroxetine in the treatment of depression. J Clin Psychiatry. 2002 Jul;63(7):577-84.
- http://www.gsk-clinicalstudyregister.com/study/29060/449#rs

172. Goldstein 2002 (HMAQ - Study Group A)

- Goldstein DJ, Mallinckrodt C, Lu Y, Demitrack MA. Duloxetine in the treatment of major depressive disorder: a double-blind clinical trial. J Clin Psychiatry. 2002; 63: 225–31.
- http://www.lillytrials.com/results/Cymbalta.pdf

173. Goldstein 2004a (HMAT - Study Group A, ID#4091)

- http://www.lillytrials.com/results/Cymbalta.pdf

174. Goldstein 2004b (HMAT - Study Group B, ID#4091)

- Goldstein DJ, Lu Y, Detke MJ, Wiltse C, Mallinckrodt C, Demitrack MA. Duloxetine in the treatment of depression: a double-blind placebo-controlled comparison with paroxetine. J Clin Psychopharmacol. 2004; 24: 389–99.

175. Gommoll 2014 (LVM-MD-02, NCT00969150)

- Gommoll CP, Greenberg WM, Chen C. A randomized, double-blind, placebo-controlled study of flexible doses of levomilnacipran ER (40–120 mg/day) in patients with major depressive disorder. Journal of Drug Assessment. 2014;3(1):10-19

176. Goodarzi 2015 (IRCT2012101811155N1)

- Goodarzi K, Astaneh N, Mazinani R, Alibeigi N, Mousavi Z. Comparison of therapeutic effects of mirtazaine and citalopram on outpatients with major depressive disorder and anxiety symptoms. World Journal of Pharmaceutical Research. 2015; 4(8):346-357.
- http://www.irct.ir/searchresult.php?id=11155&number=1

177. Gorman 2002 (SCT-MD-02)

- Gorman JM, Korotzer A, Su G. Efficacy comparison of escitalopram and citalopram in the treatment of major depressive disorder: pooled analysis of placebo-controlled trials. CNS Spectr. 2002 Apr;7(4 Suppl 1):40-4.
- www.ncbi.nlm.nih.gov/pubmedhealth/PMH0016624/bin/app17c_et6.pdf

178. Griebel 2012 (Study DF15878, NCT00358631)

- Griebel G, Beeské S, Stahl SM. The vasopressin V(1b) receptor antagonist SSR149415 in the treatment of major depressive and generalized anxiety disorders: results from 4 randomized, double-blind, placebo-controlled studies. J Clin Psychiatry. 2012 Nov;73(11):1403-11.
- https://clinicaltrials.gov/ct2/show/NCT00358631

179. Griebel 2012b (Study DF15879, NCT00361491)

- Griebel G, Beeské S, Stahl SM. The vasopressin V(1b) receptor antagonist SSR149415 in the treatment of major depressive and generalized anxiety disorders: results from 4 randomized, double-blind, placebo-controlled studies. J Clin Psychiatry. 2012 Nov;73(11):1403-11.
- https://clinicaltrials.gov/ct2/show/NCT00361491

180. Grunebaum 2012 (NCT00429169)

- Grunebaum MF, Ellis SP, Duan N, Burke AK, Oquendo MA, Mann JJ. Pilot Randomized Clinical Trial of an SSRI vs Bupropion: Effects on Suicidal Behavior, Ideation, and Mood in Major Depression. Neuropsychopharmacology. 2012 Feb; 37(3): 697–706.
- https://clinicaltrials.gov/ct2/show/NCT00429169

181. GSK14

- http://www.gsk-clinicalstudyregister.com/study/14#rs

182. Guelfi 1995 (VEN 600A-206 FDA)

- Guelfi JD, White C, Hackett D, Guichoux JY, Magni G. Effectiveness of venlafaxine in patients hospitalized for major depression and melancholia. J Clin Psychiatry. 1995 Oct;56(10):450-8.
- http://digitalcommons.ohsu.edu/fdadrug/24/

183. Guelfi 1998

- Guelfi JD, Ansseau M, Corruble E, Samuelian JC, Tonelli I, Tournoux A, Plétan Y. A double-blind comparison of the efficacy and safety of milnacipran and fluoxetine in depressed inpatients. Int Clin Psychopharmacol. 1998 May;13(3):121-8.

184. Guillibert 1989 (PAR 290 MDF 29060 1727 M)

- Guillibert E, Pelicier Y, Archambault JC, Chabannes JP, Clerc G, Desvilles M, Guibert M, Pagot R, Poisat JL, Thobie Y. A double-blind, multicentre study of paroxetine versus clomipramine in depressed elderly patients. Acta Psychiatr Scand Suppl. 1989;350:132-4.
- https://www.gsk-clinicalstudyregister.com/study/29060/290?search=study&#rs

185. Hackett 1998

- Hackett D, Salinas E, Desmet A. Efficacy and safety of venlafaxine vs. fluvoxamine in outpatients with major depression. Eur Neuropsychopharmacol. 1998;8(Supp2):S209

186. Hale 2010 (CL3-20098-045)

- Hale A, Corral RM, Mencacci C, Ruiz JS, Severo CA, Gentil V. Superior antidepressant efficacy results of agomelatine versus fluoxetine in severe MDD patients: a randomized, double-blind study. Int Clin Psychopharmacol. 2010 Nov;25(6):305-14.
- https://www.tga.gov.au/sites/default/files/auspar-valdoxan.pdf

187. Halikas 1995 (MIR 003-023 - FDA)

- Halikas JA. Org 3770 (Mirtazapine) versus Trazodone: A Placebo Controlled Trial in Depressed Elderly Patients. Human Psychopharmacology 1995;10:S125-S133.
- http://digitalcommons.ohsu.edu/fdadrug/20/

188. Hao 2014

- Hao X, Yang F, Kuang H, Shi J, Du B, Zhang K, Zhang H. Duloxetine hydrochloride enteric tablets in the treatment of major depressive disorder: a multicentre, randomised, double-blind and positive drug parallel controlled clinical trial. Chinese Journal of new Drugs. 2014;23:2767-2780.

189. Harris 1991

- Harris B, Szulecka TK, Anstee JA. Fluvoxamine versus amitriptyline in depressed hospital out-patients: A multicentre double-blind comparative trial. Br J Clin Res. 1991;2:89-99.

190. Heiligenstein 1994

- Heiligenstein J, Faries D, Rush A, Andersen J, Pande A, Roffwarg H, Dunner D, Gillin J, James S, Lahmeyer H. Latency to rapid eye movement sleep as a predictor of treatment response to fluoxetine and placebo in nonpsychotic depressed outpatients. Psychiatry Res. 1994;52:327-39.

191. Heller 2009 (NCT00909155)

- Heller AS, Johnstone T, Shackman AJ, Light SN, Peterson MJ, Kolden GG, Kalin NH, Davidson RJ. Reduced capacity to sustain positive emotion in major depression reflects diminished maintenance of fronto-striatal brain activation. Proc Natl Acad Sci U S A. 2009 Dec 29;106(52):22445-50.
- https://clinicaltrials.gov/ct2/show/NCT00909155

192. Henigsberg 2012 (305, NCT00735709)

- Henigsberg N, Mahableshwarkar AR, Jacobsen P, Chen Y, Thase ME. A randomized, double-blind, placebo-controlled 8-week trial of the efficacy and tolerability of multiple doses of Lu AA21004 in adults with major depressive disorder. J Clin Psychiatry 2012;73(7):953-9.
- https://clinicaltrials.gov/ct2/show/NCT00735709

193. Heun 2013 (lSRCTl\l5750736O)

- Heun R, Ahokas A, Boyer P, Giménez-Montesinos N, Pontes-Soares F, Olivier V; Agomelatine Study Group. The efficacy of agomelatine in elderly patients with recurrent major depressive disorder: a placebo-controlled study. J Clin Psychiatry. 2013 Jun;74(6):587-94.

194. Hewett 2009 (WXL101497)

- Hewett K, Chrzanowski W, Schmitz M, Savela A, Milanova V, Gee M, Krishen A, Millen L, Leary MO, Modell J. Eight-week, placebo-controlled, double-blind comparison of the antidepressant efficacy and tolerability of bupropion XR and venlafaxine XR. J Psychopharmacol. 2009;23(5):531-8.
- http://www.gsk-clinicalstudyregister.com/study/101497#rs

195. Hewett 2010a (AK130940, NCT00093288)

- Hewett K, Chrzanowski W, Schmitz M, Savela A, Milanova V, Gee M, Krishen A, Millen L, Leary MO, Modell J. Eight-week, placebo-controlled, double-blind comparison of the antidepressant efficacy and tolerability of bupropion XR and venlafaxine XR. J Psychopharmacol. 2009;23(5):531-8.
- http://www.gsk-clinicalstudyregister.com/study/AK130940#ps
- https://clinicaltrials.gov/ct2/show/NCT00093288

196. Hewett 2010b (AK130939)

- Hewett K, Gee MD, Krishen A, Wunderlich HP, Le Clus A, Evoniuk G, Modell JG. Double-blind, placebo-controlled comparison of the antidepressant efficacy and tolerability of bupropion XR and venlafaxine XR. J Psychopharmacol. 2010;24(8):1209-16.
- http://www.gsk-clinicalstudyregister.com/study/AK130939#rs

197. Hicks 1988

- Hicks F, Robins E, Murphy GE. Comparison of adinazolam, amitriptyline, and placebo in the treatment of melancholic depression. Psychiatry Res. 1988 Feb;23(2):221-7.

198. Hicks 2002

- Hicks JA, Argyropoulos SV, Rich AS, Nash JR, Bell CJ, Edwards C, Nutt DJ, Wilson SJ. Randomised controlled study of sleep after nefazodone or paroxetine treatment in out-patients with depression. Br J Psychiatry. 2002 Jun;180:528-35.

199. Higuchi 2014 (NCT01441440)

- Higuchi T, Kamijima K, Nakagome K, Itamura R, Asami Y, Kuribayashi K, Imaeda T. A randomized, double-blinded, placebo-controlled study to evaluate the efficacy and safety of venlafaxine extended release and a long-term extension study for patients with major depressive disorder in Japan. Int Clin Psychopharmacol. 2016 Jan;31(1):8-19.
- https://clinicaltrials.gov/ct2/show/NCT01441440

200. Higuchi 2009

- Higuchi T, Murasaki M, Kamijima K. Clinical evaluation of duloxetine in the treatment of major depressive disorder. Jpn J Clin Psychopharmacol 2009;12:1613-1634.

201. Higuchi 2011 (PCR112810, NCT00866294)

- Higuchi T, Hong JP, Jung HY, Watanabe Y, Kunitomi T, Kamijima K. Paroxetine controlled-release formulation in the treatment of major depressive disorder: a randomized, double-blind, placebo-controlled study in Japan and Korea. Psychiatry Clin Neurosci 2011;65:655-63.
- http://www.gsk-clinicalstudyregister.com/study/112810#ps
- https://clinicaltrials.gov/ct2/show/NCT00866294

202. Hirayasu 2011a

- Hirayasu Y. A dose-response study of escitalopram in patients with major depressive disorder: a placebo-controlled, double-blind study. Jpn J Clin Psychopharmacol 2011;14:871-882.

203. Hirayasu 2011b

- Hirayasu Y. A dose-response and non-inferiority study evaluating the efficacy and safety of escitalopram in patients with major depressive disorder. Jpn J Clin Psychopharmacol 2011;14:883-899.

204. Hong 2003 (22521)

- Hong CJ, Hu WH, Chen CC, Hsiao CC, Tsai SJ, Ruwe FJ. A double-blind, randomized, group-comparative study of the tolerability and efficacy of 6 weeks' treatment with mirtazapine or fluoxetine in depressed Chinese patients. J Clin Psychiatry. 2003 Aug;64(8):921-6.

205. Hormazabal 1985

- Hormazabal L, Omer LM, Ismail S. Cianopramine and amitriptyline in the treatment of depressed patients--a placebo-controlled study. Psychopharmacology (Berl) 1985;86(1-2):205-8.

206. Hosseini 2015 (IRCT201203048513N1)

- Hosseini F, Amini F, Yassini Ardekani SM, Shariat N, Nadi M. Double-Blind Randomized Clinical Trial of the Efficacy of Venlafaxine Versus Citalopram in the Treatment of the Acute Phase of Major Depressive Disorder. Iran J Psychiatry Behav Sci. 2015 Jun;9(2):e1041.
- http://www.irct.ir/searchresult.php?keyword=&id=8513&field=&number=1&prt=43&total=10&m=1

207. Hoyberg 1996 (Organon 88013, FDA88013)

- Hoyberg OJ, Maragakis B, Mullin J, Norum D, Stordall E, Ekdahl P, Ose E, Moksnes KM, Sennef C. A double-blind multicentre comparison of mirtazapine and amitriptyline in elderly depressed patients. Acta Psychiatr Scand. 1996; 93(3): 184-190.

208. Hsu 2011

- Hsu JW, Su TP, Huang CY, Chen YS, Chou YH. Faster onset of antidepressant effects of citalopram compared with sertraline in drug-naïve first-episode major depressive disorder in a Chinese population: a 6-week double-blind, randomized comparative study. J Clin Psychopharmacol. 2011 Oct;31(5):577-81.

209. Hu 2009

- Hu M, Li L, Lu X, Xun G, Chen J. Escitalopram vs citalopram for depression: a randomized, double-blind, double-dummy, multicentre, parallel controlled study. Central South Pharmacy. 2009; 8(1):1-4.

210. Hunter 2010 (Study 1)

- Hunter AM, Muthén BO, Cook IA, Leuchter AF. Antidepressant response trajectories and quantitative electroencephalography (QEEG) biomarkers in major depressive disorder. J Psychiatr Res. 2010 Jan;44(2):90-8.

211. Hunter 2010 (Study 2)

- Hunter AM, Muthén BO, Cook IA, Leuchter AF. Antidepressant response trajectories and quantitative electroencephalography (QEEG) biomarkers in major depressive disorder. J Psychiatr Res. 2010 Jan;44(2):90-8.

212. Hunter 2010 (Study 3)

- Hunter AM, Muthén BO, Cook IA, Leuchter AF. Antidepressant response trajectories and quantitative electroencephalography (QEEG) biomarkers in major depressive disorder. J Psychiatr Res. 2010 Jan;44(2):90-8.

213. Hutchinson 1992

- Hutchinson DR, Tong S, Moon CA, Vince M, Clarke A. Paroxetine in the treatment of elderly depressed patients in general practice: a double-blind comparison with amitriptyline. Int Clin Psychopharmacol. 1992 Jun;6(Suppl 4):43-51.

214. Itil 1983

- Itil TM, Shrivastava RK, Mukherjee S, Coleman BS, Michael ST. A double-blind placebo-controlled study of fluvoxamine and imipramine in out-patients with primary depression. Br J Clin Pharmacol. 1983;15(Suppl 3):433S-438S.

215. Iwata 2013 (NCT00798707)

- Iwata N1, Tourian KA, Hwang E, Mele L, Vialet C. Efficacy and safety of desvenlafaxine 25 and 50mg/day in a randomized, placebo-controlled study of depressed outpatients. J Psychiatr Pract. 2013 Jan;19(1):5-14.
- https://clinicaltrials.gov/ct2/show/NCT00798707

216. Jacobsen 2015 (316, NCT01163266)

- Jacobsen PL, Mahableshwarkar AR, Serenko M, Chan S, Trivedi MH. A randomized, double-blind, placebo-controlled study of the efficacy and safety of vortioxetine 10 mg and 20 mg in adults with major depressive disorder. J Clin Psychiatry 2015;76(5):575-82.
- https://clinicaltrials.gov/ct2/show/NCT01163266

217. Jain2013 (303, NCT00672958)

- Jain R, Mahableshwarkar AR, Jacobsen PL, Chen Y, Thase ME. A randomized, double-blind, placebo-controlled 6-wk trial of the efficacy and tolerability of 5 mg vortioxetine in adults with major depressive disorder. Int J Neuropsychopharmacol. 2013;16(2):313-21.
- https://clinicaltrials.gov/ct2/show/NCT00672958

218. Jefferson 2000 (29060/785)

- Jefferson J, Griest J. A double-blind, placebo controlled, fixed-dosage study comparing the efficacy and tolerability of paroxetine CR and citalopram to placebo in the treatment of Major Depressive Disorder with anxiety. American College of Neuropsychopharmacology, 39th Annual Meeting 12/11/2000 San Juan, Puerto Rico.
- http://www.gsk-clinicalstudyregister.com/study/29060/785#csr

219. Jefferson 2006

- Jefferson JW, Rush AJ, Nelson JC, VanMeter SA, Krishen A, Hampton KD, Wightman DS, Modell JG. Extended-release bupropion for patients with major depressive disorder presenting with symptoms of reduced energy, pleasure, and interest: findings from a randomized, double-blind, placebo-controlled study. J Clin Psychiatry. 2006 Jun;67(6):865-73.

220. Jiang 2009

- Jiang X, Ren K. A comparative study on escitalopram and citalopram hydrobromide in the treatment of depression. Journal of Qiqihar Medical College, 2009; 30(1):1-2.

221. Judd 1993

- Judd FK, Moore K, Norman TR, Burrows GD, Gupta RK, Parker G. A multicentre double blind trial of fluoxetine versus amitriptyline in the treatment of depressive illness. Aust N Z J Psychiatry. 1993 Mar;27(1):49-55.

222. Kamijima 2013 (japicCTI-090733)

- Kamijima K, Hashimoto S, Nagayoshi E, Koyama T. Double-blind, comparative study of milnacipran and paroxetine in Japanese patients with major depression. Neuropsychiatr Dis Treat. 2013;9:555-65.

223. Kane 1983

- Kane JM, Cole K, Sarantakos S, Howard A, Borenstein M. Safety and efficacy of bupropion in elderly patients: preliminary observations. J Clin Psychiatry. 1983 May;44(5 Pt 2):134-6.

224. Kasper 2005a (Study 99024)

- Kasper S, de Swart H, Friis Andersen H. Escitalopram in the treatment of depressed elderly patients. Am J Geriatr Psychiatry. 2005 Oct;13(10):884-91.

225. Kasper 2005b

- Kasper S, Olivieri L, Di Loreto G, Dionisio P. A comparative, randomised, double-blind study of trazodone prolonged-release and paroxetine in the treatment of patients with major depressive disorder. Curr Med Res Opin. 2005 Aug;21(8):1139-46.

226. Kasper 2012 (NCT00807248)

- Kasper S, Ebert B, Larsen K, Tonnoir B. Combining escitalopram with gaboxadol provides no additional benefit in the treatment of patients with severe major depressive disorder. Int J Neuropsychopharmacol. 2012 Jul;15(6):715-25.
- https://clinicaltrials.gov/ct2/show/NCT00807248

227. Katona 2012 (12541A, NCT00811252)

- Katona C, Hansen T, Olsen CK. A randomized, double-blind, placebo-controlled, duloxetine-referenced, fixed-dose study comparing the efficacy and safety of Lu AA21004 in elderly patients with major depressive disorder. Int Clin Psychopharmacol 2012;27(4):215-23.
- https://clinicaltrials.gov/ct2/show/NCT00811252

228. Katz 1993a

- Katz RJ, Lott M, Landau P, Waldmeier P. A clinical test of noradrenergic involvement in the therapeutic mode of action of an experimental antidepressant. Biol Psychiatry. 1993 Feb 15;33(4):261-6.

229. Katz 1993b

- Katz RJ, Lott M, Landau P, Waldmeier P. A clinical test of noradrenergic involvement in the therapeutic mode of action of an experimental antidepressant. Biol Psychiatry. 1993 Feb 15;33(4):261-6.

230. Katz 2004

- Katz MM, Tekell JL, Bowden CL, Brannan S, Houston JP, Berman N, Frazer A. Onset and early behavioral effects of pharmacologically different antidepressants and placebo in depression. Neuropsychopharmacology. 2004;29(3):566-79.

231. Kavoussi 1997

- Kavoussi RJ, Segraves RT, Hughes AR, Ascher JA, Johnston JA. Double-blind comparison of bupropion sustained release and sertraline in depressed outpatients. J Clin Psychiatry. 1997 Dec;58(12):532-7.

232. Keegan 1991

- Keegan D, Bowen RC, Blackshaw S, Saleh S, Dayal N, Remillard F, Shrikhande S, Cebrian Perez S, Boulton A. A comparison of fluoxetine and amitriptyline in the treatment of major depression. Int Clin Psychopharmacol. 1991 Summer;6(2):117-24.

233. Kellams 1979

- Kellams JJ, Klapper M, Small J. Trazodone, a new antidepressant: efficacy and safety in endogenous depression. J Clin Psychiatry. 1979;40(9):390-5.

234. Keller 2006a (Study059, NCT00035009)

- Keller M, Montgomery S, Ball W, Morrison M, Snavely D, Liu G, Hargreaves R, Hietala J, Lines C, Beebe K, Reines S. Lack of efficacy of the substance p (neurokinin1 receptor) antagonist aprepitant in the treatment of major depressive disorder. Biol Psychiatry. 2006 Feb 1;59(3):216-23.
- https://clinicaltrials.gov/ct2/show/NCT00035009

235. Keller 2006b (Study061, NCT00035295)

- Keller M, Montgomery S, Ball W, Morrison M, Snavely D, Liu G, Hargreaves R, Hietala J, Lines C, Beebe K, Reines S. Lack of efficacy of the substance p (neurokinin1 receptor) antagonist aprepitant in the treatment of major depressive disorder. Biol Psychiatry. 2006 Feb 1;59(3):216-23.
- https://clinicaltrials.gov/ct2/show/NCT00035295?term=NCT00035295&rank=1

236. Keller 2006c (Study062, NCT00048607)

- Keller M, Montgomery S, Ball W, Morrison M, Snavely D, Liu G, Hargreaves R, Hietala J, Lines C, Beebe K, Reines S. Lack of efficacy of the substance p (neurokinin1 receptor) antagonist aprepitant in the treatment of major depressive disorder. Biol Psychiatry. 2006 Feb 1;59(3):216-23.
- https://clinicaltrials.gov/ct2/show/NCT00048607?term=NCT00048607&rank=1

237. Keller 2007 (NCT00046020)

- Keller MB, Trivedi MH, Thase ME, Shelton RC, Kornstein SG, Nemeroff CB, Friedman ES, Gelenberg AJ, Kocsis JH, Dunner DL, Hirschfeld RM, Rothschild AJ, Ferguson JM, Schatzberg AF, Zajecka JM, Pedersen RD, Yan B, Ahmed S, Musgnung J, Ninan PT. The Prevention of Recurrent Episodes of Depression with Venlafaxine for Two Years (PREVENT) Study: Outcomes from the 2-year and combined maintenance phases. J Clin Psychiatry. 2007 Aug;68(8):1246-56.
- https://clinicaltrials.gov/ct2/show/NCT00046020

238. Kennedy 2005 (SCT-MD-16)

- Kennedy SH, Anderson HF, Lam RW. A pooled analysis of selective serotonin reuptake inhibitors and venlafaxine. Poster presented at the American Psychiatric Association, May 2005.

239. Kennedy 2006 (CL3-20098-043)

- Kennedy, SH, Emsley, R Placebo-controlled trial of agomelatine in the treatment of major depressive disorder. Eur Neuropsychopharm 2006; 16: 93-100.
- http://www.ema.europa.eu/docs/en_GB/document_library/EPAR_-_Public_assessment_report/human/000916/WC500038315.pdf

240. Kennedy 2014 (EudraCT 2009-011238-84, CL3-20098-069)

- Kennedy SH, Avedisova A, Giménez-Montesinos N, Belaïdi C, de Bodinat C; Agomelatine Study Group. A placebo-controlled study of three agomelatine dose regimens (10 mg, 25 mg, 25-50 mg) in patients with major depressive disorder. Eur Neuropsychopharmacol. 2014 Apr;24(4):553-63.
- https://www.clinicaltrialsregister.eu/ctr-search/search?query=2009-011238-84
- http://www.ema.europa.eu/docs/en_GB/document_library/EPAR_-_Public_assessment_report/human/000916/WC500038315.pdf

241. Kerkhofs 1990

- Kerkhofs M, Rielaert C, de Maertelaer V, Linkowski P, Czarka M, Mendlewicz J. Fluoxetine in major depression: efficacy, safety and effects on sleep polygraphic variables. Int Clin Psychopharmacol. 1990 Oct;5(4):253-60.

242. Kerr 1984

- Kerr TA, McClelland HA, Stephens DA, Ankier SI. Trazodone. A comparative clinical and predictive study. Acta Psychiatr Scand. 1984 Dec;70(6):573-7.

243. Khan 1998

- Khan A, Upton GV, Rudolph RL, Entsuah R, Leventer SM. The use of venlafaxine in the treatment of major depression and major depression associated with anxiety: a dose-response study. Venlafaxine Investigator Study Group. J Clin Psychopharmacol. 1998 Feb;18(1):19-25.

244. Khan 2007 (SCT-MD-23, NCT00108979)

- https://clinicaltrials.gov/ct2/show/NCT00108979

245. Khan 2011 (CLDA-07-DP-02, NCT00683592)

- Khan A, Cutler AJ, Kajdasz DK, Gallipoli S, Athanasiou M, Robinson DS, Whalen H, Reed CR. A randomized, double-blind, placebo-controlled, 8-week study of vilazodone, a serotonergic agent for the treatment of major depressive disorder. J Clin Psychiatry. 2011;72(4):441-7.
- https://clinicaltrials.gov/ct2/show/NCT00683592

246. Kiev 1997

- Kiev A, Feiger A. A double-blind comparison of fluvoxamine and paroxetine in the treatment of depressed outpatients. J Clin Psychiatry. 1997 Apr;58(4):146-52.

247. Kinoshita 2009

- Kinoshita T. A double-blind, placebo controlled study of a new antidepressant, mirtazapine, in depressed patients. Jpn J Clin Psychopharmacol 2009;12:289-306.

248. Koshino 2013 (NCT01138007)

- Koshino Y, Bahk W, Sakai H, Kobayashi T. Efficacy and safety of bupropion sustained-release formulation for the treatment of major depressive disorder: a multi-center, randomized, double-blind, placebo-controlled study in Asian patients. Neuropsychiatr Dis Treat. 2013;9:1273-80.
- https://clinicaltrials.gov/ct2/show/NCT01138007

249. Kramer 1998

- Kramer MS, Cutler N, Feighner J, Shrivastava R, Carman J, Sramek JJ, Reines SA, Liu G, Snavely D, Wyatt-Knowles E, Hale JJ, Mills SG, MacCoss M, Swain CJ, Harrison T, Hill RG, Hefti F, Scolnick EM, Cascieri MA, Chicchi GG, Sadowski S, Williams AR, Hewson L, Smith D, Carlson EJ, Hargreaves RJ, Rupniak NM. Distinct mechanism for antidepressant activity by blockade of central substance P receptors. Science. 1998 Sep 11;281(5383):1640-5.

250. Kuhs 1989 (MDUK_29060_III_85_043)

- Kuhs H, Rudolf GA. A double-blind study of the comparative antidepressant effect of paroxetine and amitriptyline. Acta Psychiatr Scand Suppl. 1989;350:145-6.
- https://www.gsk-clinicalstudyregister.com/study/29060/043?search=compound&compound= paroxetine#rs

251. Kusalic 1993

- Kusalic M1, Engelsmann F, Bradwejn J. Thyroid functioning during treatment for depression. J Psychiatry Neurosci. 1993;18(5):260-3.

252. Kyle 1998 (Study 92032 - FDA)

- Kyle CJ, Petersen HE, Overø KF. Comparison of the tolerability and efficacy of citalopram and amitriptyline in elderly depressed patients treated in general practice. Depress Anxiety. 1998;8(4):147-53.

253. Laakman 1988

- Laakmann G, Blaschke D, Engel R, Schwarz A. Fluoxetine vs amitriptyline in the treatment of depressed out-patients. Br J Psychiatry Suppl. 1988 Sep;(3):64-8.

254. Lalit 2004

- Lalit V, Appaya PM, Hegde RP, Mital AK, Mittal S, Nagpal R, Palaniappun V, Ramsubramaniam C, Rao GP, Roy K, Trivedi JK, Vankar GK, Karan RS, Shah S, Patel RB. Escitalopram Versus Citalopram and Sertraline: A Double-Blind Controlled, Multi-centric Trial in Indian Patients with Unipolar Major Depression. Indian J Psychiatry. 2004 Oct-Dec; 46(4): 333–341.

255. Lam 1995

- Lam RW, Gorman CP, Michalon M, Steiner M, Levitt AJ, Corral MR, Watson GD, Morehouse RL, Tam W, Joffe RT. Multicenter, placebo-controlled study of fluoxetine in seasonal affective disorder. Am J Psychiatry. 1995;152(12):1765-70.

256. Langlois 1985

- Langlois R, Cournoyer G, de Montigny C, Caillé G. High incidence of multisystemic reactions to zimeldine. Eur J Clin Pharmacol. 1985;28(1):67-71.

257. Lapierre 1980

- Lapierre YD, Sussman P, Ghadirian A. Differential antidepressant properties of trazodone and amitriptyline in agitated and retarded depression. Current Therapeutic Research. 1980; 28:845-856.

258. Lapierre 1987

- Lapierre YD, Browne M, Horn E, Oyewumi LK, Sarantidis D, Roberts N, Badoe K, Tessier P. Treatment of major affective disorder with fluvoxamine. J Clin Psychiatry. 1987 Feb;48(2):65-8.

259. Larsen 1989

- Larsen JK, Holm P, Høyer E, Mejlhede A, Mikkelsen PL, Olesen A, Schaumburg E. Moclobemide and clomipramine in reactive depression. A placebo-controlled randomized clinical trial. Acta Psychiatr Scand. 1989 Jun;79(6):530-6.

260. Learned 2012a Study1 (EUCTR2005-003401-87, SND103285, NCT00448058)

- Learned S, Graff O, Roychowdhury S, Moate R, Krishnan KR, Archer G, Modell JG, Alexander R, Zamuner S, Lavergne A, Evoniuk G, Ratti E. Efficacy, safety, and tolerability of a triple reuptake inhibitor GSK372475 in the treatment of patients with major depressive disorder: two randomized, placebo- and active-controlled clinical trials. J Psychopharmacol. 2012;26:653-62.
- https://www.clinicaltrialsregister.eu/ctr-search/search?query=2005-003401-87
- https://clinicaltrials.gov/ct2/show/NCT00448058

261. Learned 2012b Study2 (NCT00420641)

- Learned S, Graff O, Roychowdhury S, Moate R, Krishnan KR, Archer G, Modell JG, Alexander R, Zamuner S, Lavergne A, Evoniuk G, Ratti E. Efficacy, safety, and tolerability of a triple reuptake inhibitor GSK372475 in the treatment of patients with major depressive disorder: two randomized, placebo- and active-controlled clinical trials. J Psychopharmacol. 2012 May;26(5):653-62.
- https://clinicaltrials.gov/ct2/show/NCT00420641

262. Lecrubier 1997

- Lecrubier Y, Bourin M, Moon CA, Schifano F, Blanchard C, Danjou P, Hackett D. Efficacy of venlafaxine in depressive illness in general practice. Acta Psychiatr Scand. 1997;95(6):485-93.

263. Lee 2007 (F1J-AA-HMCV, NCT00489775)

- Lee P, Shu L, Xu X, Wang CY, Lee MS, Liu CY, Hong JP, Ruschel S, Raskin J, Colman SA, Harrison GA. Once-daily duloxetine 60 mg in the treatment of major depressive disorder: multicenter, double-blind, randomized, paroxetine-controlled, non-inferiority trial in China, Korea, Taiwan and Brazil. Psychiatry Clin Neurosci. 2007 Jun;61(3):295-307.
- https://clinicaltrials.gov/ct2/show/NCT00489775

264. Leinonen 1997

- Leinonen E, Lepola U, Koponen H, Mehtonen OP, Rimon R. Long-term efficacy and safety of milnacipran compared to clomipramine in patients with major depression. Acta Psychiatr Scand. 1997 Dec;96(6):497-504.

265. Leinonen 1999 (Agren1995)

- Leinonen E, Skarstein J, Behnke K, Agren H, Helsdingen JT. Efficacy and tolerability of mirtazapine versus citalopram: a double-blind, randomized study in patients with major depressive disorder. Nordic Antidepressant Study Group. Int Clin Psychopharmacol. 1999 Nov;14(6):329-37.

266. Lemoine 2007 (CL3-20098-035)

- Lemoine P, Guilleminault C, Alvarez E. Improvement in subjective sleep in major depressive disorder with a novel antidepressant, agomelatine: randomized, double-blind comparison with venlafaxine. J Clin Psychiatry. 2007 Nov;68(11):1723-32.
- Servier. Study CL3-20098-035. Efficacy of agomelatine (25 mg with potential adjustment at 50 mg) given orally versus venlafaxine on subjective sleep evaluation of patients with Major Depressive Disorder. A randomised double-blind parallel groups study. 6-week treatment plus optional continuation for 18 weeks. Available from Servier. 2005.

267. Lepine 2000

- Lépine JP, Goger J, Blashko C, Probst C, Moles MF, Kosolowski J, Scharfetter B, Lane RM. A double-blind study of the efficacy and safety of sertraline and clomipramine in outpatients with severe major depression. Int Clin Psychopharmacol. 2000 Sep;15(5):263-71.

268. Lepola 2003 (ESC 99003)

- Lepola UM, Loft H, Reines EH .Escitalopram (10-20 mg/day) is effective and well tolerated in a placebo-controlled study in depression inprimary care. Int Clin Psychopharmacol. 2003 Jul;18(4):211-7.

269. Li 2006

- Li J, Shen W, Liu Y, Xu L, Liu S, Kuang W. The Effectiveness and Safety of Escitalopram in the Treatment of Depression: A Randomized Double-blind Active-drug Controlled Trial. Chin J Evid-based Med. 2006; 6 (8):1-5.

270. Li 2010

- Li X, Tao F, Wang X, Li Ji. Clinical Research on Escitalopram Oxalate Tablets in Treatment of Depression. Pharmaceutical and Clinical Research. 2010; 18(1):1-3.

271. Lieberman 2008a (Study 309, EUCTR2004-000562-13, 3151A1-309-EU, NCT00090649)

- Lieberman DZ, Montgomery SA, Tourian KA, Brisard C, Rosas G, Padmanabhan K, Germain JM, Pitrosky B. A pooled analysis of two placebo-controlled trials of desvenlafaxine in major depressive disorder. Int Clin Psychopharmacol. 2008 Jul;23(4):188-97
- https://www.clinicaltrialsregister.eu/ctr-search/search?query=2004-000562-13
- https://clinicaltrials.gov/ct2/show/NCT00090649

272. Lieberman 2008b (Study 317)

- Lieberman DZ, Montgomery SA, Tourian KA, Brisard C, Rosas G, Padmanabhan K, Germain JM, Pitrosky B. A pooled analysis of two placebo-controlled trials of desvenlafaxine in major depressive disorder. Int Clin Psychopharmacol. 2008 Jul;23(4):188-97

273. Liebowitz 2008 (Study 332, NCT00277823)

- Liebowitz MR, Manley AL, Padmanabhan SK, Ganguly R, Tummala R, Tourian KA. Efficacy, safety, and tolerability of desvenlafaxine 50 mg/day and 100 mg/day in outpatients with major depressive disorder. Curr Med Res Opin. 2008;24(7):1877-90.
- https://clinicaltrials.gov/ct2/show/NCT00277823

274. Liebowitz 2013 (NCT00863798)

- Liebowitz MR1, Tourian KA, Hwang E, Mele L; Study 3362 Investigators. A double-blind, randomized, placebo-controlled study assessing the efficacy and tolerability of desvenlafaxine 10 and 50 mg/day in adult outpatients with major depressive disorder. BMC Psychiatry. 2013 Mar 22;13:94.
- https://www.clinicaltrials.gov/ct2/show/NCT00863798

275. Lineberry 1990 (WELL 84A)

- Lineberry CG, Johnston JA, Raymond RN, Samara B, Feighner JP, Harto NE, Granacher RP Jr, Weisler RH, Carman JS, Boyer WF. A fixed-dose (300 mg) efficacy study of bupropion and placebo in depressed outpatients. J Clin Psychiatry. 1990;51(5):194-9.
- http://www.gsk-clinicalstudyregister.com/study/Well%2084A#rs

276. Loo 2002 (CL2-014)

- Lôo H, Hale A, D'haenen H. Determination of the dose of agomelatine, a melatoninergic agonist and selective 5-HT(2C) antagonist, in the treatment of major depressive disorder: a placebo-controlled dose range study. Int Clin Psychopharmacol. 2002 Sep;17(5):239-47.

277. Lopez-Rodriguez 2004

- Rodriguez JL, Lopez Butron MA, Vargas Terrez BE, Villamil Salcedo V. Estudio doble ciego con antidepressivo, psicoterapia breve y placebo en pacientes con depression leve a moderada. Salud Mental 2004;27:53-61.

278. Lv 2013

- Lv Z, Zhang Y, Qi S. A randomized double-blind study of escitalopram and citalopram in treating major depression. J Clin Psychiatry. 2013;23(1):1-3.

279. Lydiard 1989

- Lydiard RB, Laird LK, Morton WA Jr, Steele TE, Kellner C, Laraia MT, Ballenger JC. Fluvoxamine, imipramine, and placebo in the treatment of depressed outpatients: effects on depression. Psychopharmacol Bull. 1989;25(1):68-70.

280. Lydiard 1997

- Lydiard RB1, Stahl SM, Hertzman M, Harrison WM. A double-blind, placebo-controlled study comparing the effects of sertraline versus amitriptyline in the treatment of major depression. J Clin Psychiatry. 1997 Nov;58(11):484-91.

281. M/2020/0046 (Study 046)

- https://www.iqwig.de/de/projekte-ergebnisse/studieninformationen-zu-reboxetin.3304.html

282. M/2020/0047 (Study 047)

- https://www.iqwig.de/de/projekte-ergebnisse/studieninformationen-zu-reboxetin.3304.html

283. Mahableshwarkar 2013 (304, NCT00672620)

- Mahableshwarkar AR, Jacobsen PL, Chen Y. A randomized, double-blind trial of 2.5 mg and 5 mg vortioxetine (Lu AA21004) versus placebo for 8 weeks in adults with major depressive disorder. Curr Med Res Opin. 2013;29(3):217-26.
- https://clinicaltrials.gov/ct2/show/NCT00672620

284. Mahableshwarkar 2015a (315, NCT01153009)

- Mahableshwarkar AR, Jacobsen PL, Chen Y, Serenko M, Trivedi MH. A randomized, double-blind, duloxetine-referenced study comparing efficacy and tolerability of 2 fixed doses of vortioxetine in the acute treatment of adults with MDD. Psychopharmacology (Berl). 2015;232(12):2061-70.
- https://clinicaltrials.gov/ct2/show/NCT01153009

285. Mahableshwarkar 2015b (317, NCT01179516)

- Mahableshwarkar AR, Jacobsen PL, Serenko M, Chen Y, Trivedi MH. A randomized, double-blind, placebo-controlled study of the efficacy and safety of 2 doses of vortioxetine in adults with major depressive disorder. J Clin Psychiatry 2015;76(5):583-91.
- https://clinicaltrials.gov/ct2/show/NCT01179516

286. Mahableshwarkar 2015c (NCT01564862)

- Mahableshwarkar AR, Zajecka J, Jacobson W, Chen Y, Keefe RS. A Randomized, Placebo-Controlled, Active-Reference, Double-Blind, Flexible-Dose Study of the Efficacy of Vortioxetine on Cognitive Function in Major Depressive Disorder. Neuropsychopharmacology. 2015;40(8):2025-37.
- https://clinicaltrials.gov/ct2/show/NCT01564862

287. Mann 1981

- Mann JJ, Georgotas A, Newton R, Gershon S. A controlled study of trazodone, imipramine, and placebo in outpatients with endogenous depression. J Clin Psychopharmacol. 1981;1(2):75-80.

288. Manna 1989

- Manna V, Martucci N, Agnoli A. Double-blind controlled study on the clinical efficacy and safety of fluoxetine vs clomipramine in the treatment of major depressive disorders. Int Clin Psychopharmacol. 1989 Jan;4 Suppl 1:81-8.

289. Mao 2008

- Mao P, Tang Y, Jiang F, Shu L, Gu X, Li M, Qian M, Ma C, Mitchell PB, Cai Z. Escitalopram in major depressive disorder: a multicenter, randomized, double-blind, fixed-dose, parallel trial in a Chinese population. Depression and Anxiety. 2008; 25(1): 46–54.

290. Mao 2015 (NCT01098318)

- Mao JJ, Xie SX, Zee J, Soeller I, Li QS, Rockwell K, Amsterdam JD. Rhodiola rosea versus sertraline for major depressive disorder: A randomized placebo-controlled trial. Phytomedicine. 2015 Mar 15;22(3):394-9.
- https://clinicaltrials.gov/ct2/show/NCT01098318

291. March 1990

- March JS, Kobak KA, Jefferson JW, Mazza J, Greist JH. A double-blind, placebo-controlled trial of fluvoxamine versus imipramine in outpatients with major depression. J Clin Psychiatry. 1990 May;51(5):200-2.

292. Marchesi 1998

- Marchesi C, Ceccherininelli A, Rossi A, Maggini C. Is Anxious-Agitated Major Depression Responsive to Fluoxetine? A Double-Blind Comparison with Amitriptyline. Pharmacopsychiatry. 1998;31(6):216-21.

293. Masco 1985

- Masco HL, Sheetz MS. Double-blind comparison of Fluoxetine and Amitripyline in the treatment of Major Depressive Illness. Advances in Therapy. 1985; 2(6):275-283.

294. Mathews 2015 (NCT01473381)

- Mathews M, Gommoll C, Chen D, Nunez R, Khan A. Efficacy and safety of vilazodone 20 and 40 mg in major depressive disorder: a randomized, double-blind, placebo-controlled trial. Int Clin Psychopharmacol. 2015;30(2):67-74.
- https://clinicaltrials.gov/ct2/show/NCT01473381

295. McGrath 2000

- McGrath PJ, Stewart JW, Janal MN, Petkova E, Quitkin FM, Klein DF. A placebo-controlled study of fluoxetine versus imipramine in the acute treatment of atypical depression. Am J Psychiatry. 2000 Mar;157(3):344-50.

296. McIntyre 2014 (NCT01422213)

- McIntyre RS, Lophaven S, Olsen CK. A randomized, double-blind, placebo-controlled study of vortioxetine on cognitive function in depressed adults. Int J Neuropsychopharmacol. 2014;17(10):1557-67.
- https://clinicaltrials.gov/ct2/show/NCT01422213

297. McPartlin 1998

- McPartlin GM, Reynolds A, Anderson C, Casoy J. A comparison of once daily venlafaxine xr and paroxetine in depressed outpatients treated in general practice. Primary Care Psychiatry. 1998;4:127-32.

298. MDF/29060/III/070/88/MC

- https://www.gsk-clinicalstudyregister.com/study/29060/070#rs

299. MDUK/26090/III/83/007

- https://www.gsk-clinicalstudyregister.com/study/26090/007#rs

300. Mehtonen 2000

- Mehtonen OP, Sogaard J, Roponen P, Behnke K. Randomized, Double-Blind Comparison of Venlafaxine and Sertraline in Outpatients With Major Depressive Disorder. The Journal of Clinical Psychiatry. 2000;61(2):95-100.

301. Mendels 1995 (FDA 03A0A-004B)

- Mendels J, Reimherr F, Marcus RN, Roberts DL, Francis RJ, Anton SF. A double-blind, placebo-controlled trial of two dose ranges of nefazodone in the treatment of depressed outpatients. J Clin Psychiatry. 1995;56 Suppl 6:30-6.
- http://digitalcommons.ohsu.edu/fdadrug/23/

302. Mendels 1999 (Study 85A - FDA)

- Mendels J, Kiev A, Fabre LF. Double blind comparison of citalopram and placebo in depressed outpatients with melancholia. Depress Anxiety. 1999;9(2):54-60.
- http://www.accessdata.fda.gov/drugsatfda_docs/nda/98/020822a.cfm

303. Miller 1989 (MDUK/29060/III/82/006, PAR-274, PAR UK 06 - FDA)

- Miller SM, Naylor GJ, Murtagh M, Winslow G. A double-blind comparison of paroxetine and placebo in the treatment of depressed patients in a psychiatric outpatient clinic. Acta Psychiatr Scand Suppl. 1989;350:143-4
- http://digitalcommons.ohsu.edu/fdadrug/26/

304. MIR 003-003 (FDA)

- http://digitalcommons.ohsu.edu/fdadrug/20/

305. MIR 003-020 (FDA)

- http://digitalcommons.ohsu.edu/fdadrug/20/

306. MIR 003-021 (FDA)

- http://digitalcommons.ohsu.edu/fdadrug/20/

307. MIR 84062 (FDA)

- http://digitalcommons.ohsu.edu/fdadrug/20/

308. Mischoulon 2014 (NCT00101452)

- A Double-Blind, Randomized, Placebo-Controlled Clinical Trial of S-Adenosyl-l-Methionine (SAMe) Versus Escitalopram in Major Depressive Disorder. The Journal of Clinical Psychiatry. 2014;75(4):370-76.
- https://clinicaltrials.gov/ct2/show/NCT00101452

309. Miura 2000

- Miura S, Koyama T, Asai M, Endo S, Kamijima K, Ushijima S, Murasaki M, Kariya T, Koshino Y, Nakajima T, Nishimura K, Kudo Y, Saito M, Watanabe M, Tashiro N, Nakane Y. Clinical evaluation of paroxetine HCl, a selective serotonin reuptake inhibitor, in the treatment of depression or depressive spisodes: Double-blind, parallel group study with amitriptyline HCl. Yakuri To Chiro (Jpn Pharmacol Ther). 2000; 28 Supplement: 187-210. (in Japanese)

310. Moises 1981

- Moises HW, Kasper S, Beckmann H. Trazodone and amitriptyline in treatment of depressed inpatients. A double-blind study. Pharmacopsychiatria. 1981 Sep;14(5):167-71.

311. Moller1993 (Stuppaeck1994, MY-059.073/29060/1)

- Möller HJ, Berzewski H, Eckmann F, Gonzalves N, Kissling W, Knorr W, Ressler P, Rudolf GA, Steinmeyer EM, Magyar I. Double-blind multicenter study of paroxetine and amitriptyline in depressed inpatients. Pharmacopsychiatry. 1993 May;26(3):75-8.
- https://www.gsk-clinicalstudyregister.com/study/29060/073?search=study&#rs

312. Moller 1998 (Hegerl 1997)

- Möller HJ, Gallinat J, Hegerl U, Arató M, Janka Z, Pflug B, Bauer H. Double-Blind, Multicenter Comparative Study of Sertraline and Amitriptyline in Hospitalized Patients with Major Depression. Pharmacopsychiatry. 1998:31(5):170-7.

313. Moller 2000

- Möller HJ, Glaser K, Leverkus F, Göbel C. Double-blind, multicenter comparative study of sertraline versus amitriptyline in outpatients with major depression. Pharmacopsychiatry. 2000 Nov;33(6):206-12.

314. Montgomery 1992 (Study 89303 FDA)

- http://www.accessdata.fda.gov/drugsatfda_docs/nda/98/020822a.cfm

315. Montgomery 2004a (Study 99067)

- Montgomery SA, Huusom AKT, Bothmer J. A Randomised Study Comparing Escitalopram with Venlafaxine XR in Primary Care Patients with Major Depressive Disorder. Neuropsychobiology. 2004;50(1):57-64.

316. Montgomery 2004b (CL3-20098-030)

- Montgomery SA, Kennedy SH, Burrows GD, Lejoyeux M, Hindmarch I. Absence of discontinuation symptoms with agomelatine and occurrence of discontinuation symptoms with paroxetine: A randomized, double-blind, placebo-controlled discontinuation study. International Clinical Psychopharmacology. 2004;19(5):271-80.

317. Montgomery 2013 (F02695LP202, EudraCT2006-002404-34)

- Montgomery SA, Mansuy L, Ruth A, Bose A, Li H, Li D. Efficacy and safety of levomilnacipran sustained release in moderate to severe major depressive disorder: a randomized, double-blind, placebo-controlled, proof-of-concept study. J Clin Psychiatry 2013;74(4):363-9.
- https://www.clinicaltrialsregister.eu/ctr-search/search?query=2006-002404-34

318. Moon 1994

- Moon CAL, Jago W, Wood K, Doogan DP. A double-blind comparison of sertraline and clomipramine in the treatment of major depressive disorder and associated anxiety in general practice. Journal of Psychopharmacology. 1994;8(3):171-6.

319. Moore 2005

- Moore N, Verdoux H, Fantino B. Prospective, multicentre, randomized, double-blind study of the efficacy of escitalopram versus citalopram in outpatient treatment of major depressive disorder. Int Clin Psychopharmacol. 2005 May;20(3):131-7.

320. Moreno 2005

- Moreno RA, Teng CT, Almeida KM, Tavares Junior H. Hypericum perforatum versus fluoxetine in the treatment of mild to moderate depression: a randomized double-blind trial in a Brazilian sample. Rev Bras Psiquiatr. 2006 Mar;28(1):29-32.

321. Moscovitch 2004

- Moscovitch A, Blashko CA, Eagles JM, Darcourt G, Thompson C, Kasper S, Lane RM; International Collaborative Group on Sertraline in the Treatment of Outpatients with Seasonal Affective Disorders. A placebo-controlled study of sertraline in the treatment of outpatients with seasonal affective disorder. Psychopharmacology (Berl). 2004 Feb;171(4):390-7.

322. Mullin 1996

- Mullin J, Lodge A, Bennie E, McCreadie R, Bhatt GS, Fenton G. A multicentre, double-blind, amitriptyline-controlled study of mirtazapine in patients with major depression. Journal of Psychopharmacology.1996;10(3): 235-240

323. Mundt 2012 (NCT00406952)

- Mundt JC, Vogel AP, Feltner DE, Lenderking WR. Vocal acoustic biomarkers of depression severity and treatment response. Biol Psychiatry. 2012 Oct 1;72(7):580-7.
- https://clinicaltrials.gov/ct2/show/NCT00406952

324. Munizza 2006

- Munizza C, Olivieri L, Di Loreto G, Dionisio P. A comparative, randomized, double-blind study of trazodone prolonged-release and sertraline in the treatment of major depressive disorder. Curr Med Res Opin. 2006 Sep;22(9):1703-13.

325. Murasaki 1998

- Murasaki M, Mori A, Miura S et al. Clinical evaluation of SME3110 (fluvoxamine maleate) in the treatment of depression and depressive state: A double-blind, comparative study with amitriptyline. Rinsho-Iyaku (Journal of Clinical Therapeutics & Medicines) 1998; 14(5): 951-980. (in Japanese)

326. Murasaki 2010a (iQWIG22532, Schoemaker 2002)

- Murasaki M, Schoemaker JH, Miyake K, Gailledreau J, Heukels AJ, Fennema HP, Sitsen JMA. Comparison of efficacy and safety of mirtazapine versus fluvoxamine in Japanese and Caucasian patients with major depressive disorder. Rinsho-Seishin-Yakuri (Japanese Journal of Clinical Psychopharmacology 2010 13(2): 339-355. (in Japanese)

327. Murasaki 2010b (iQWIG9902, Schoemaker 2002)

- Murasaki M, Schoemaker JH, Miyake K, Gailledreau J, Heukels AJ, Fennema HP, Sitsen JMA. Comparison of efficacy and safety of mirtazapine versus fluvoxamine in Japanese and Caucasian patients with major depressive disorder. Rinsho-Seishin-Yakuri (Japanese Journal of Clinical Psychopharmacology 2010 13(2): 339-355. (in Japanese)

328. MY-1008/BRL-029060/2/CPMS-076

- https://www.gsk-clinicalstudyregister.com/study/29060/076#rs

329. MY-1042/BRL-029060/CPMS-251

- http://www.gsk-clinicalstudyregister.com/study/29060/251?search=compound&compound=paroxetine#rs

330. MY-1043/BRL-029060/115

- http://www.gsk-clinicalstudyregister.com/study/29060/115#rs

331. MY-1045/BRL-029060/1 (PAR 128)

- http://www.gsk-clinicalstudyregister.com/study/29060/128#rs

332. Mynors-Wallis 1995

- Mynors-Wallis LM, Gath DH, Lloyd-Thomas AR, Tomlinson D. Randomised controlled trial comparing problem solving treatment with amitriptyline and placebo for major depression in primary care. BMJ 1995 Feb 18;310(6977):441-5.

333. NCT00822744 (EudraCT Number2008-001718-26)

- https://clinicaltrials.gov/ct2/show/NCT00822744
- https://www.clinicaltrialsregister.eu/ctr-search/trial/2008-001718-26/SK

334. NCT01020799

- https://clinicaltrials.gov/ct2/show/NCT01020799

335. NCT01145755

- https://clinicaltrials.gov/ct2/show/NCT01145755

336. NCT01254305

- https://clinicaltrials.gov/ct2/show/NCT01254305

337. NCT01255787 (EUCTR2010-022257-41, JapicCTI-101344, CTRI/2011/08/001963)

- https://clinicaltrials.gov/ct2/show/NCT01255787
- https://www.clinicaltrialsregister.eu/ctr-search/search?query=2010-022257-41

338. NCT01355081 (japicCTI-111492, U1111-1120-9277)

- https://clinicaltrials.gov/ct2/show/NCT01355081

339. NCT01808612

- https://clinicaltrials.gov/ct2/show/NCT01808612

340. Nemeroff 1995

- Nemeroff CB, Ninan P, Ballenger J, Lydiard RB, Feighner J, Patterson WM, Greist JH. Double-blind multicenter comparison of fluvoxamine versus sertraline in the treatment of depressed outpatients. Depression. 1995;3(4):163-69.

341. Nemeroff 2007

- Nemeroff CB, Thase ME; EPIC 014 Study Group. A double-blind, placebo-controlled comparison of venlafaxine and fluoxetine treatment in depressed outpatients. J Psychiatry Res 2007;41: 351–59.

342. Newhouse 2000

- Newhouse PA, Krishnan KR, Doraiswamy PM, Richter EM, Batzar ED, Clary CM. A double-blind comparison of sertraline and fluoxetine in depressed elderly patients. J Clin Psychiatry. 2000;61(8):559-68.

343. Nierenberg 2007 (F1J-MC-HMCR, NCT00073411, Pigott 2007)

- Nierenberg AA, Greist JH, Mallinckrodt CH, et al. Duloxetine versus escitalopram and placebo in the treatment of patients with major depressive disorder: onset of antidepressant action, a non-inferiority study. Curr Med Res Opin 2007; 23: 401–16.
- http://www.lillytrials.com/results/Cymbalta.pdf
- https://clinicaltrials.gov/ct2/show/NCT00073411

344. Ninan 2003 (poster SCT-MD-26)

- www.ncbi.nlm.nih.gov/pubmedhealth/PMH0016624/bin/app17c_et6.pdf

345. NKD20006 (NCT00048204)

- http://www.gsk-clinicalstudyregister.com/study/NKD20006#ps
- https://clinicaltrials.gov/ct2/show/NCT00048204

346. Noguera 1991

- Noguera R, Altuna R. Fluoxetine vs. clomipramine in depressed patients: a controlled multicentre trial. J Affect Disord. 1991;22(3):119-24.

347. Norton 1984

- Norton KR, Sireling LI, Bhat AV, Rao B, Paykel ES. A double-blind comparison of fluvoxamine, imipramine and placebo in depressed patients. J Affect Disord. 1984;7:297-308.

348. Oakes 2012a (NCT00536471)

- Oakes TM, Myers AL, Marangell LB, Ahl J, Prakash A, Thase ME, Kornstein SG. Assessment of depressive symptoms and functional outcomes in patients with major depressive disorder treated with duloxetine versus placebo: primary outcomes from two trials conducted under the same protocol. Hum Psychopharmacol. 2012 Jan;27(1):47-56.
- https://www.clinicaltrials.gov/ct2/show/NCT00536471

349. Oakes 2012b (NCT00536471)

- Oakes TM, Myers AL, Marangell LB, Ahl J, Prakash A, Thase ME, Kornstein SG. Assessment of depressive symptoms and functional outcomes in patients with major depressive disorder treated with duloxetine versus placebo: primary outcomes from two trials conducted under the same protocol. Hum Psychopharmacol. 2012 Jan;27(1):47-56.
- https://www.clinicaltrials.gov/ct2/show/NCT00536471

350. Olie 1997

- Olie J, Gunn K, Katz E. A double-blind placebo-controlled multicentre study of sertraline in the acute and continuation treatment of major depression. Eur Psychiatry. 1997;12(1):34-41.

351. Olie 2007 (CL3-20098-042)

- Olie, JP, Kasper, S Efficacy of agomelatine, a MT1/MT2 receptor agonist with 5-HT2C antagonistic properties, in major depressive disorder. Int J Neuropsychoph 2007; 10: 661-73.
- http://www.ema.europa.eu/docs/en_GB/document_library/EPAR_-_Public_assessment_report/human/000916/WC500038315.pdf

352. Ontiveros 1994 (MY-0144/BRL 29060/1/CPMS-135, PAR135)

- Ontiveros A, Garcia-Barriga C. A double-blind, comparative study of paroxetine and fluoxetine in out-patients with depression. Brit J Clin Research. 1997; 8:23-32.
- https://www.gsk-clinicalstudyregister.com/study/29060/135#rs

353. Ontiveros Sanchez 1998

- Ontiveros Sánchez JA, Brandi F, Brunner E. Estudio doble-ciego sobre fluoxetina vs amitriptilina en los síntomas depresivos y de ansiedad, y calidad de vida de los adultos con depresión mayor. Salud Mental. 1998;21(1):58-63.

354. Ottevanger 1995

- Ottevanger EA. Fluvoxamine and clomipramine in depressed hospitalised patients: Results from a randomised, double-blind study. L Encéphale. 1995;21(4):317-21.

355. Ou 2010

- Ou JJ, Xun GL, Wu RR, Li LH, Fang MS, Zhang HG, Xie SP, Shi JG, Du B, Yuan XQ, Zhao JP. Efficacy and safety of escitalopram versus citalopram in major depressive disorder: a 6-week, multicenter, randomized, double-blind, flexible-dose study. Psychopharmacology. 2011; 213(2): 639–46.

356. PAR 01 001 (GSK & FDA)

- http://digitalcommons.ohsu.edu/fdadrug/26/

357. PAR 279 MDUK

- http://www.gsk-clinicalstudyregister.com/study/29060/012_3?search=compound&compound=paroxetine#rs

358. PAR 29060.308 (HP/81/74A)

- https://www.gsk-clinicalstudyregister.com/study/29060/308#rs

359. PAR 29060.310 (HP 81/85A)

- https://www.gsk-clinicalstudyregister.com/study/29060/310#rs

360. PAR 29060.314 (HP 82/134)

- https://www.gsk-clinicalstudyregister.com/study/29060/314?search=study&#rs

361. PAR 29060.316 (HP/82/47A)

- https://www.gsk-clinicalstudyregister.com/study/29060/316#rs

362. PAR 29060.318 (HP/82/64A)

- https://www.gsk-clinicalstudyregister.com/study/29060/318#rs

363. PAR 29060/281

- https://www.gsk-clinicalstudyregister.com/study/29060/281#rs

364. PAR MDUK 032

- https://www.gsk-clinicalstudyregister.com/study/29060/032#rs

365. Patris 1996 (Study 91301 - FDA)

- Patris M, Bouchard JM, Bougerol T, Charbonnier JF, Chevalier JF, Clerc G, Cyran C, Van Amerongen P, Lemming O, Høpfner-Peterson HE. Citalopram versus fluoxetine: A double-blind, controlled, multicentre, phase III trial in patients with unipolar major depression treated in general practice. International Clinical Psychopharmacology. 1996;11(2):129-36.

366. Paykel 1988

- Paykel ES, Hollyman JA, Freeling P, Sedgwick P. Predictors of therapeutic benefit from amitriptyline in mild depression: a general practice placebo-controlled trial. J Affect Disord. 1988 Jan-Feb;14(1):83-95.

367. Pélicier 1993 (PAR MDED/29060/III/86/1728M291)

- Pélicier Y, Schaeffer P. Multicenter double-blind study comparing the efficacy and tolerance of paroxetine and clomipramine in reactive depression in the elderly patient. L Encéphale. 1993;19(3):257-61.
- https://www.gsk-clinicalstudyregister.com/study/29060/291#rs

368. Perahia 2006 (HMAY - Study Group B)

- Perahia DG, Wang F, Mallinckrodt CH, Walker DJ, Detke MJ. Duloxetine in the treatment of major depressive disorder: a placebo- and paroxetine-controlled trial. Eur Psychiatry 2006; 21: 367–78.
- http://www.lillytrials.com/results/Cymbalta.pdf

369. Perahia 2008a (HMBU, NCT00071695)

- Perahia DGS, Pritchett Y, Kajdasz D, Bauer M, Jain R, Russell JM, Walker DJ, Spencer KA, Froud DM, Raskin J, Thase ME. A randomized, double-blind comparison of duloxetine and venlafaxine in the treatment of patients with major depressive disorder. Journal of Psychiatric Research. 2008;42(1):22-34.
- https://www.clinicaltrials.gov/ct2/show/NCT00071695

370. Perahia 2008b (HMCQ, NCT00067912)

- Perahia DGS, Pritchett Y, Kajdasz D, Bauer M, Jain R, Russell JM, Walker DJ, Spencer KA, Froud DM, Raskin J, Thase ME. A randomized, double-blind comparison of duloxetine and venlafaxine in the treatment of patients with major depressive disorder. Journal of Psychiatric Research. 2008;42(1):22-34.
- https://clinicaltrials.gov/ct2/show/NCT00067912

371. Pomara 2013

- Croft HA, Pomara N, Gommoll C, Chen D, Nunez R, Mathews M. Efficacy and Safety of Vilazodone in Major Depressive Disorder: A Randomized, Double-blind, Placebo-controlled Trial. J Clin Psychiatry. 2014 Nov;75(11):e1291-8.

372. Preskorn 1991

- Preskorn S, Silkey B, Beber J, Dorey C. Antidepressant Response and Plasma Concentrations of Fluoxetine. Annals of Clinical Psychiatry. 1991;3(2):147-151.

373. PZ/109

- Hieronymus F, Emilsson JF, Nilsson S, Eriksson E. Consistent superiority of selective serotonin reuptake inhibitors over placebo in reducing depressed mood in patients with major depression. Mol Psychiatry. 2016 Apr;21(4):523-30.

374. PZ/111

- Hieronymus F, Emilsson JF, Nilsson S, Eriksson E. Consistent superiority of selective serotonin reuptake inhibitors over placebo in reducing depressed mood in patients with major depression. Mol Psychiatry. 2016 Apr;21(4):523-30.

375. Quera-Salva 2010 (CL3-20098-056)

- Quera-Salva MA, Hajak G, Philip P, Montplaisir J, Keufer-Le Gall S, Laredo J, Guilleminault C. Comparison of agomelatine and escitalopram on nighttime sleep and daytime condition and efficacy in major depressive disorder patients. International Clin psychopharmacology. 2011;26(5):252-62.
- https://www.clinicaltrialsregister.eu/ctr-search/trial/2006-004716-48/results

376. Raft 1981

- Raft D, Davidson J, Wasik J, Mattox A. Relationship between response to phenelzine and MAO inhibition in a clinical trial of phenelzine, amitriptyline and placebo. Neuropsychobiology. 1981;7(3):122-6.

377. Rapaport 1996

- Rapaport M, Coccaro EF, Sheline YI, Perse T, Holland P, Fabre L, Bradford D. A Comparison of Fluvoxamine and Fluoxetine in the Treatment of Major Depression. Journal of Clinical Psychopharmacology. 1996;16(5):373-8.

378. Rapaport 2003 (PAR487)

- Rapaport MH, Schneider LS, Dunner DL, Davies JT, Pitts CD. Efficacy of controlled-release paroxetine in the treatment of late-life depression. J Clin Psychiatry 2003;64(9):1065-74.
- http://www.gsk-clinicalstudyregister.com/study/29060/487#rs

379. Rapaport 2009 (BRL-29060/874, NCT00067444)

- Rapaport MH, Lydiard RB, Pitts CD, Schaefer D, Bartolic EI, Iyengar M, Carfagno M, Lipschitz A. Low doses of controlled-release paroxetine in the treatment of late-life depression: a randomized, placebo-controlled trial. J Clin Psychiatry. 2009 Jan;70(1):46-57.
- https://www.clinicaltrials.gov/ct2/show/NCT00067444

380. Raskin 2007 (HMBV, NCT00062673)

- Raskin J, Wiltse CG, Siegal A, Sheikh J, Xu J, Dinkel JJ, Rotz BT, Mohs RC. Efficacy of duloxetine on cognition, depression, and pain in elderly patients with major depressive disorder: an 8-week, double-blind, placebo-controlled trial. Am J Psychiatry 2007;164(6):900-9.
- http://www.lillytrials.com/results/Cymbalta.pdf
- https://clinicaltrials.gov/ct2/show/NCT00062673

381. Ravindran 1995

- Ravindran AV, Teehan MD, Bakish D, Yatham L, O'Reilly R, Fernando ML, Manchanda R, Charbonneau Y, Buttars J. The impact of sertraline, desipramine, and placebo on psychomotor functioning in depression. Human Psychopharmacology. 1995; 10(4):273–281.

382. Ravindran 1997 (MY-1055/BRL-029060/1/CPMS-245 PAR 245)

- Ravindran AV, Judge R, Hunter BN, Bray J, Morten NH. A double-blind, multicenter study in primary care comparing paroxetine and clomipramine in subjects with depression and associated anxiety. J. Clin. Psychiatry. 1997; 58:112-118.
- https://www.gsk-clinicalstudyregister.com/study/29060/245#rs

383. Reimherr 1990 (SER 104 - FDA)

- Reimherr FW, Chouinard G, Cohn CK, Cole JO, Itil TM, LaPierre YD, Masco HL, Mendels J. Antidepressant efficacy of sertraline: a double-blind, placebo- and amitriptyline-controlled, multicenter comparison study in outpatients with major depression. J Clin Psychiatry. 1990 Dec;51 Suppl B:18-27.
- http://digitalcommons.ohsu.edu/fdadrug/27/

384. Reimherr 1998 (WELL203)

- Reimherr FW, Cunningham LA, Batey SR, Johnston JA, Ascher JA. A multicenter evaluation of the efficacy and safety of 150 and 300 mg/d sustained-release bupropion tabletsversus placebo in depressed outpatients. Clin Ther. 1998 May-Jun;20(3):505-16.
- http://digitalcommons.ohsu.edu/fdadrug/21/

385. Remick 1994

- Remick RA, Reesal R, Oakander M, Allen J, Claman J, Ramirez CE, Perry K, Keller FD. Comparison of fluvoxamine and amitriptyline in depressed outpatients. Current Therapeutic Research. 1994;55(3):243-250.

386. Rickels 1982

- Rickels K, Case WG. Trazodone in depressed outpatients. Am J Psychiatry. 1982 Jun;139(6):803-6.

387. Rickels 1985

- Rickels K, Feighner JP, Smith WT. Alprazolam, amitriptyline, doxepin, and placebo in the treatment of depression. Arch Gen Psychiatry. 1985 Feb;42(2):134-41.

388. Rickels 1994 (FDA CN104-005)

- Rickels K, Schweizer E, Clary C, Fox I, Weise C. Nefazodone and imipramine in major depression: a placebo-controlled trial. Br J Psychiatry. 1994;164(6):802-5.
- http://digitalcommons.ohsu.edu/fdadrug/23/

389. Rickels 1995 (FDA CN104-006-1)

- Rickels K, Robinson DS, Schweizer E, Marcus RN, Roberts DL. Nefazodone: aspects of efficacy. J Clin Psychiatry. 1995;56 Suppl 6:43-6.
- http://digitalcommons.ohsu.edu/fdadrug/23/

390. Rickels 2009 (GNSC-04-DP-02, NCT00285376)

- Rickels K, Athanasiou M, Robinson DS, Gibertini M, Whalen H, Reed CR. Evidence for efficacy and tolerability of vilazodone in the treatment of major depressive disorder: a randomized, double-blind, placebo-controlled trial. J Clin Psychiatry. 2009 Mar;70(3):326-33.
- https://clinicaltrials.gov/ct2/show/NCT00285376

391. Robinson 2014 (NCT00406848)

- Robinson M, Oakes TM, Raskin J, Liu P, Shoemaker S, Nelson JC. Acute and long-term treatment of late-life major depressive disorder: duloxetine versus placebo. Am J Geriatr Psychiatry. 2014 Jan;22(1):34-45.
- https://clinicaltrials.gov/ct2/show/NCT00406848

392. Roffman 1982

- Roffman M, Gould EE. A Double-blind comparative study of oxaprotiline with amitriptyline and placebo in moderate depression. Current Therapeutic Research 1982;32:2247-256.

393. Roose 2004 (CIT-MD-03)

- Roose SP, Sackeim HA, Krishnan KR, Pollock BG, Alexopoulos G, Lavretsky H, Katz IR, Hakkarainen H; Old-Old Depression Study Group. Antidepressant pharmacotherapy in the treatment of depression in the very old: a randomized, placebo-controlled trial. Am J Psychiatry. 2004 Nov;161(11):2050-9.

394. Ropert 1989

- Ropert R. Fluoxetine versus clomipramine in major depressive disorders. Int Clin Psychopharmacol. 1989 Jan;4 Suppl 1:89-95.

395. Rossini 2005

- Rossini D, Serretti A, Franchini L, Mandelli L, Smeraldi E, De Ronchi D, Zanardi R. Sertraline Versus Fluvoxamine in the Treatment of Elderly Patients With Major Depression Journal of Clinical Psychopharmacology. 2005;25(5):471-5.

396. Roth 1990

- Roth D, Mattes J, Sheehan KH, Sheehan DV. A double-blind comparison of fluvoxamine, desipramine and placebo in outpatients with depression. Progress in Neuro-Psychopharmacology and Biological Psychiatry. 1990;14(6):929–939.

397. Rouillon 1991 (Limosin 2006, 029060/1/CPMS 069 1991)

- Rouillon F. A double-blind, multicentre study comparing increasing doses of paroxetine (20-50mg) and clomipramine (50-150mg) in elderly patients with major depression. World Congress of Biological Psychiatry 1991:28 (poster presentation)
- Limosin F, Samuelian JC, Rouillon F. Multicenter Double-Blind Study of the Efficacy of Paroxetine versus Clomipramine in Elderly Patients with Major Depression. Journal of Aging and Pharmacotherapy. 2006;13(2):7-19.
- https://www.gsk-clinicalstudyregister.com/study/29060/069#rs

398. Rowan 1980

- Rowan P, Paykel ES, Parker PR, West E. Comparative effects of phenelzine and amitriptyline: a placebo controlled trial. Neuropharmacology. 1980 Dec;19(12):1223-5.

399. Rudolph 1998 (VEN 600A-203 (FDA)

- Rudolph RL, Fabre LF, Feighner JP, Rickels K, Entsuah R, Derivan AT. A randomized, placebo-controlled, dose-response trial of venlafaxine hydrochloride in the treatment of major depression. J Clin Psychiatry. 1998 Mar;59(3):116-22.
- http://digitalcommons.ohsu.edu/fdadrug/24/

400. Rudolph 1999

- Rudolph RL, Feiger AD. A double-blind, randomized, placebo-controlled trial of once-daily venlafaxine extended release (xr) and fluoxetine for the treatment of depression. J Affect Disord 1999; 56: 171–81.

401. Rush 1998 (excluding Armitage 1997 and Gillin 1997)

- Rush AJ, Armitage R, Gillin JC, Yonkers KA, Winokur A, Moldofsky H, Vogel GW, Kaplita SB, Fleming JB, Montplaisir J, Erman MK, Albala BJ, McQuade RD. Comparative effects of nefazodone and fluoxetine on sleep in outpatients with major depressive disorder. Biol Psychiatry. 1998 Jul 1;44(1):3-14.

402. Rush 2001

- Rush AJ, Trivedi MH, Carmody TJ, Donahue RM, Houser TL, Bolden-Watson C, Batey SR, Ascher JA, Metz A. Response in relation to baseline anxiety levels in major depressive disorder treated with bupropion sustained release or sertraline. Neuropsychopharmacology. 2001 Jul;25(1):131-8.

403. Sacchetti 2002 (BRL-29060/109)

- Sacchetti E, Cassano GB, Penati G, Pavan L, Nardini M, Casacchia M, Pancheri P, Muscettola G, Faravelli C, Aguglia E, Nivoli G, Ravizza L, Castrogiovanni P. Paroxetine versus amitriptyline in patients with recurrent major depression: A double-blind trial. Int J Psychiatry Clin Pract. 2002;6(1):23-9.
- https://www.gsk-clinicalstudyregister.com/study/29060/109#rs

404. Sambunaris 2014 (LVM-MD-03, NCT01034462)

- Sambunaris A, Bose A, Gommoll CP, Chen C, Greenberg WM, Sheehan DV. A phase III, double-blind, placebo-controlled, flexible-dose study of levomilnacipran extended-release in patients with major depressive disorder. J Clin Psychopharmacol. 2014 Feb;34(1):47-56.
- https://clinicaltrials.gov/ct2/show/NCT01034462

405. Samuelian 1998

- Samuelian JC, Hackett D. A Randomized, Double-Blind, Parallel-Group Comparison of Venlafaxine and Clomipramine in Outpatients With Major Depression. J Psychopharmacol. 1998;12 (3):273-278.

406. Sauer 2003

- Sauer H, Huppertz-Helmhold S, Dierkes W. Efficacy and safety of venlafaxine ER vs. amitriptyline ER in patients with major depression of moderate severity. Pharmacopsychiatry. 2003 Sep;36(5):169-75.

407. Schatzberg 2002 (003-901)

- Schatzberg AF, Kremer C, Rodrigues HE, Murphy GM Jr: Mirtazapine vs. Paroxetine Study Group. Double-blind, randomized comparison of mirtazapine and paroxetine in elderly depressed patients. Am J Geriatr Psychiatry. 2002 Sep-Oct;10(5):541-50.

408. Schatzberg 2006a

- Schatzberg A1, Roose S. A double-blind, placebo-controlled study of venlafaxine and fluoxetine in geriatric outpatients with major depression. Am J Geriatr Psychiatry. 2006 Apr;14(4):361-70.

409. Schneider 2003

- Schneider LS, Nelson JC, Clary CM, Newhouse P, Krishnan KR, Shiovitz T, Weihs K; Sertraline Elderly Depression Study Group. An 8-week multicenter, parallel-group, double-blind, placebo-controlled study of sertraline in elderly outpatients with major depression. Am J Psychiatry. 2003 Jul;160(7):1277-85.

410. Schoene 1993 (Geretsegger 1994, MY1021/BRC)

- Schöne W, Ludwig M. A double-blind study of paroxetine compared with fluoxetine in geriatric patients with major depression. J Clin Psychopharmacol. 1993 Dec;13(6 Suppl 2):34S-39S.

411. Schwartz 2002 (iQWIG)

- https://www.iqwig.de/download/Studie_Schwartz_et_al_Poster.pdf

412. Schweizer 1994 (VEN 600A-301 FDA)

- Schweizer E, Feighner J, Mandos LA, Rickels K. Comparison of venlafaxine and imipramine in the acute treatment of major depression in outpatients. J Clin Psychiatry. 1994 Mar;55(3):104-8.
- http://digitalcommons.ohsu.edu/fdadrug/24/

413. SCT-MD-09

- Forest Research Institute. Double-blind comparison of the effects of lu 26-054 (escitalopram) and fluoxetine on sleep in depressed patients (SCT-MD-09).

414. SCT-MD-49 (NCT00668525)

- https://clinicaltrials.gov/ct2/show/NCT00668525

415. Sechter 1999

- Sechter D, Troy S, Paternetti S, Boyer P. A double-blind comparison of sertraline and fluoxetine in the treatment of major depressive episode in outpatients. Eur Psychiatry. 1999 Mar;14(1):41-8.

416. Sechter 2004

- Sechter D, Vandel P, Weiller E, Pezous N, Cabanac F, Tournoux A; study co-coordinators. A comparative study of milnacipran and paroxetine in outpatients with major depression. J Affect Disord. 2004 Dec;83(2-3):233-6.

417. SER 101 (FDA)

- http://digitalcommons.ohsu.edu/fdadrug/27/

418. SER 310 (FDA)

- http://digitalcommons.ohsu.edu/fdadrug/27/

419. SER 315 (FDA)

- http://digitalcommons.ohsu.edu/fdadrug/27/

420. SER-CHN-1

- https://www.gsk-clinicalstudyregister.com/study/29060/403#rs

421. Settle 1999 (WELL212)

- Settle EC, Stahl SM, Batey SR, Johnston JA, Ascher JA. Safety profile of sustained-release bupropion in depression: results of three clinical trials. Clin Ther. 1999;21(3):454-63.
- http://digitalcommons.ohsu.edu/fdadrug/21/

422. Shaw 1986

- Shaw DM, Thomas DR, Briscoe MH, Watkins SE, Crimmins R, Harris B, Lovett J, Raj M, Lloyd AT, Osborne C. A comparison of the antidepressant action of citalopram and amitriptyline. Br J Psychiatry. 1986 Oct;149:515-7.

423. Sheehan 2009a

- Sheehan DV, Nemeroff CB, Thase ME, Entsuah R; EPIC 016 Study Group. Placebo-controlled inpatient comparison of venlafaxine and fluoxetine for the treatment of major depression with melancholic features. Int Clin Psychopharmacol. 2009 Mar;24(2):61-86.

424. Sheehan 2009b (NCT00775203)

- Sheehan DV, Croft HA, Gossen ER, Levitt RJ, Brullé C, Bouchard S, Rozova A. Extended-release Trazodone in Major Depressive Disorder: A Randomized, Double-blind, Placebo-controlled Study. Psychiatry (Edgmont). 2009;6(5):20-33.
- https://clinicaltrials.gov/ct2/show/NCT00775203

425. Shelton 2006 (NCT00179283)

- Shipley JE, Kupfer DJ, Spiker DG, Shaw DH, Coble PA, Neil JF, Cofsky J. Neuropsychological assessment and EEG sleep in affective disorders. Biol Psychiatry. 1981 Oct;16(10):907-18.
- https://clinicaltrials.gov/ct2/show/NCT00179283

426. Shipley 1981

- Shipley JE, Kupfer DJ, Spiker DG, Shaw DH, Coble PA, Neil JF, Cofsky J. Neuropsychological assessment and EEG sleep in affective disorders. Biol Psychiatry. 1981 Oct;16(10):907-18.

427. Shu 2014 (CL3-20098-052, ChiCTR-TRC-11001668)

- Shu L, Sulaiman AH, Huang YS, Fones Soon Leng C, Crutel VS, Kim YS. Comparable efficacy and safety of 8 weeks treatment with agomelatine 25-50mg or fluoxetine 20-40mg in Asian out-patients with major depressive disorder. Asian J Psychiatr. 2014 Apr;8:26-32.

428. Silverstone 1999

- Silverstone PH, Ravindran A. Once-daily venlafaxine extended release (xr) compared with fluoxetine in outpatients with depression and anxiety. Venlafaxine xr 360 study group. J Clin Psychiatry 1999; 60: 22–28.

429. Sir 2005

- Sir A, D'Souza RF, Uguz S, George T, Vahip S, Hopwood M, Martin AJ, Lam W, Burt T. Randomized trial of sertraline versus venlafaxine XR in major depression: efficacy and discontinuation symptoms. J Clin Psychiatry. 2005 Oct;66(10):1312-20.

430. Smeraldi 1998

- Smeraldi E, Aguglia A, Cattaneo M, Cerati C, Covelli C, Del Zompo M, Faravelli C, Gerrari G, Ghirlanda A, Guazzelli G, Mansi M, Ravizza L.Double-blind, randomized study of venlafaxine, clomipramine, and trazodone in geriatric patients with major depression. Eur Neuropychopharm. 1997;7(2):S170.

431. Smith 1990 (MIR 003-024 FDA)

- Smith WT, Glaudin V, Panagides J, Gilvary E. Mirtazapine vs Amitriptyline vs Placebo in the treatment of major depressive disorder. Psychopharmacologi Bulltin 1990;26:191-195.
- http://digitalcommons.ohsu.edu/fdadrug/20/

432. Sramek 1995

- Sramek JJ, Kashkin K, Jasinsky O, Kardatzke D, Kennedy S, Cutler NR. Placebo-controlled study of ABT-200 versus fluoxetine in the treatment of major depressive disorder. Depression 1995;3:199-203.

433. Stahl 2000

- Stahl SM. Placebo-controlled comparison of the selective serotonin reuptake inhibitors citalopram and sertraline. Biol Psychiatry. 2000 Nov 1;48(9):894-901.

434. Stahl 2010 (CAGO178A2302)

- Stahl, SM, Fava, M, Trivedi, MH, Caputo, A, Shah, A, Post, A Agomelatine in the treatment of major depressive disorder: an 8-week, multicenter, randomized, placebo-controlled trial. J Clin Psychiat 2010; 71: 616-26.
- https://clinicaltrials.gov/ct2/show/NCT00411242

435. Staner 1995 (063)

- https://www.gsk-clinicalstudyregister.com/study/29060/063?#rs

436. Stark 1985 (Study 27 - FDA)

- Stark P, Hardison CD. A review of multicenter controlled studies of fluoxetine vs. imipramine and placebo in outpatients with major depressive disorder. J Clin Psychiatry 1985;46:53-8.
- http://digitalcommons.ohsu.edu/fdadrug/22/

437. Stratas 1984

- Stratas NE. A double-blind study of the efficacy and safety of dothiepin hydrochloride in the treatment of major depressive disorder. J Clin Psychiatry. 1984 Nov;45(11):466-9.

438. Studie009 (CTN009-FCE20124)

- https://www.iqwig.de/de/projekte-ergebnisse/studieninformationen-zu-reboxetin.3304.html

439. Studie032 (M2020/0032)

- https://www.iqwig.de/download/Studie_032.pdf

440. Study 015

- https://www.iqwig.de/de/projekte-ergebnisse/studieninformationen-zu-reboxetin.3304.html

441. Study 032a (CTN032-FCE20124)

- https://www.iqwig.de/de/projekte-ergebnisse/studieninformationen-zu-reboxetin.3304.html

442. Study 043

- https://www.iqwig.de/download/Studie_043.pdf

443. Study 045

- https://www.iqwig.de/de/projekte-ergebnisse/studieninformationen-zu-reboxetin.3304.html

444. Study 049

- https://www.iqwig.de/de/projekte-ergebnisse/studieninformationen-zu-reboxetin.3304.html

445. Study 19 (FDA) (Fabre 1985)

- Fabre, LF, Crismon, L. Efficacy of fluoxetine in outpatients with major depression. Current Therapeutic Research. 1985;37(1):115-23.
- http://digitalcommons.ohsu.edu/fdadrug/22/

446. Study 205 (FDA) (WELL 205)

- http://digitalcommons.ohsu.edu/fdadrug/21/

447. Study 25 (FDA) (Rickels 1986)

- Rickels K, Amsterdam JD, Avallone MF. Fluoxetine in major depression: A controlled study. Current Therapeutic Research. 1986;39(4):559-63.
- http://digitalcommons.ohsu.edu/fdadrug/22/

448. Study 62a (FDA) - MILD depression (Dunlop 1990)

- Dunlop SR, Dornseif BE, Wernicke JR, Potvin JH. Pattern analysis shows beneficial effect of fluoxetine treatment in mild depression. Psychopharmacology bulletin. 1990;26(2):173-80.
- http://digitalcommons.ohsu.edu/fdadrug/22/

449. Study 62b (FDA) - MODERATE depression

- http://digitalcommons.ohsu.edu/fdadrug/22/

450. Study 89306 (FDA)

- http://www.accessdata.fda.gov/drugsatfda_docs/nda/98/020822a_medr_P2.pdf

451. Study F1J-MC-HMAQ - Study Group B (starting page in the pdf document: 147)

- http://www.lillytrials.com/results/Cymbalta.pdf

452. Szegedi 2006

- Benkert O, Szegedi A, Philipp M, Kohnen R, Heinrich C, Heukels A, van der Vegte-Senden M, Baker RA, Simmons JH, Schutte AJ. Mirtazapine orally disintegrating tablets versus venlafaxine extended release: a double-blind, randomized multicenter trial comparing the onset of antidepressant response in patients with major depressive disorder. J Clin Psychopharmacol. 2006 Feb;26(1):75-8.

453. Thase 1997 (VEN XR 209 FDA)

- Thase ME. Efficacy and tolerability of once-daily venlafaxine extended release (XR) in outpatients with major depression. J Clin Psychiatry. 1997;58(9):393-8.
- http://digitalcommons.ohsu.edu/fdadrug/25/

454. Thase 2006

- Thase ME, Clayton AH, Haight BR, Thompson AH, Modell JG, Johnston JA. A double-blind comparison between bupropion XL and venlafaxine XR: sexual functioning, antidepressant efficacy, and tolerability. J Clin Psychopharmacol. 2006 Oct;26(5):482-8.

455. Thomson 1982

- Thomson J, Rankin H, Ashcroft GW, Yates CM, McQueen JK, Cummings SW. The treatment of depression in general practice: a comparison of L-tryptophan, amitriptyline, and a combination of L-tryptophan and amitriptyline with placebo. Psychol Med. 1982 Nov;12(4):741-51.

456. Tignol 1993 (29060/087)

- Tignol J. A double-blind, randomized, fluoxetine-controlled, multicenter study of paroxetine in the treatment of depression. J Clin Psychopharmacol. 1993 Dec;13(6 Suppl 2):18S-22S.
- https://www.gsk-clinicalstudyregister.com/study/29060/087

457. Timmerman 1993 (Study 89422 – FDA, Haffmans 1996)

- Haffmans PM, Timmerman L, Hoogduin CA. Efficacy and tolerability of citalopram in comparison with fluvoxamine in depressed outpatients: a double-blind, multicentre study. The LUCIFER Group. Int Clin Psychopharmacol. 1996 Sep;11(3):157-64.

458. Tollefson 1995

- Tollefson GD, Bosomworth JC, Heiligenstein JH, Potvin JH, Holman S. A double-blind, placebo-controlled clinical trial of fluoxetine in geriatric patients with major depression. The Fluoxetine Collaborative Study Group. Int Psychogeriatr. 1995 Spring;7(1):89-104.

459. Tomarken 2004

- Tomarken AJ, Dichter GS, Freid C, Addington S, Shelton RC. Assessing the effects of bupropion SR on mood dimensions of depression. J Affect Disord. 2004 Mar;78(3):235-41.

460. Tourian 2009 (NCT00384033)

- Tourian KA, Padmanabhan SK, Groark J. Desvenlafaxine 50 and 100 mg/d in the Treatment of Major Depressive Disorder: An 8-Week, Phase III, Multicenter, Randomized, Double-Blind, Placebo-Controlled, Parallel Group Trial and a Post Hoc Pooled Analysis of Three Studies. Clinical Therapeutics 2009;31:1405-1423.
- https://clinicaltrials.gov/show/NCT00384033

461. Trivedi 2004 (29060/810)

- Trivedi MH, Pigotti TA, Perera P, Dillingham KE, Carfagno ML, Pitts CD. Effectiveness of low doses of paroxetine controlled release in the treatment of major depressive disorder. J Clin Psychiatry. 2004;65(10):1356-64.
- http://www.gsk-clinicalstudyregister.com/study/29060/810?search=study&#rs

462. Tsutsui 2000

- Tsutsui S, Okuse T, Sasaki D et al. Clinical evaluation of paroxetine HCl, a selective serotonin reuptake inhibitor, in the treatment of depression or depressive episodes: Double-blind, parallel group study with trazodone HCl. Yakuri-To-Chiryo (Jpn Pharmacol Ther) 2000; Supplement: 161-185.

463. Tylee 1997

- Tylee A, Beaumont G, Bowden MW, Reynolds A. A double-blind, randomized, 12-week comparison study of the safety and efficacy of venlafaxine and fluoxetine in moderate to severe major depression in general practice. Primary Care Psychiatry. 1997;3:51-58.

464. Tzanakaki 2000

- Tzanakaki M, Guazzelli M, Nimatoudis I, Zissis NP, Smeraldi E, Rizzo F. Increased remission rates with venlafaxine compared with fluoxetine in hospitalized patients with major depression and melancholia. Int Clin Psychopharmacol. 2000 Jan;15(1):29-34.

465. Upward 1988

- Upward JW, Edwards JG, Goldie A, Waller DG. Comparative effects of fluoxetine and amitriptyline on cardiac function. Br J Clin Pharmacol. 1988 Oct; 26(4): 399–402.

466. Van de Merwe 1984

- Van de Merwe TJ, Silverstone T, Ankier SI, Warrington SJ, Turner P. A double-blind non-crossover placebo-controlled study between group comparison of trazodone and amitriptyline on cardiovascular function in major depressive disorder. Psychopathology. 1984;17 Suppl 2:64-76.

467. Van Moffaert 1995a

- Van Moffaert M, Bartholome F, Cosyns P, De Nayer AR, Mertens C. A controlled comparison of sertraline and fluoxetine in acute and continuation treatment of major depression. Human Psychopharm. 1995;10(5):393–405.

468. Van Moffaert 1995b

- Van Moffaert M, de Wilde J, Vereecken A, Dierick M, Evrard JL, Wilmotte J, Mendlewicz J. Mirtazapine is more effective than trazodone: a double-blind controlled study in hospitalized patients with major depression. Int Clin Psychopharmacol. 1995 Mar;10(1):3-9.

469. Vartiainen 1994 (MIR 84023 FDA)

- Vartiainen H, Leinonen E. Double-blind study of mirtazapine and placebo in hospitalized patients with major depression. Eur Neuropsychopharmacol. 1994 Jun;4(2):145-50.
- http://digitalcommons.ohsu.edu/fdadrug/20/

470. VEN 600A-303 (FDA)

- http://digitalcommons.ohsu.edu/fdadrug/24/

471. VEN 600A-313 (FDA)

- http://digitalcommons.ohsu.edu/fdadrug/24/

472. VEN XR 367 (FDA)

- http://digitalcommons.ohsu.edu/fdadrug/25/

473. Ventura 2007 (SCT-MD-18)

- Ventura D, Armstrong EP, Skrepnek GH, Haim Erder M. Escitalopram versus sertraline in the treatment of major depressive disorder: A randomized clinical trial Current Medical Research and Opinion. 2007;23(2):245-50.

474. Versiani 1999

- Versiani M, Ontiveros JA, Mazzotti G, Ospina J, Dávila J, Mata S, Pacheco A, Plewes JM, Tamura RN, Palacios M. Fluoxetine versus amitriptyline in the treatment of major depression with associated anxiety (anxious depression): A double-blind comparison. International Clinical Psychopharmacology. 1993;8(3):143-9.

475. Versiani 2000 (Study 091)

- Versiani M, Amin M, Chouinard G. Double-blind, placebo-controlled study with reboxetine in inpatients with severe major depressive disorder. J Clin Psychopharmacol. 2000;20(1):28-34
- https://www.iqwig.de/de/projekte-ergebnisse/studieninformationen-zu-reboxetin.3304.html

476. Versiani 2005

- Versiani M, Moreno R, Ramakers-van Moorsel CJ, Schutte AJ; Comparative Efficacy Antidepressants Study Group. Comparison of the effects of mirtazapine and fluoxetine in severely depressed patients. CNS Drugs. 2005;19(2):137-46.

477. Wade 2002 (ESC Study 99001 - FDA)

- Wade A, Michael Lemming O, Bang Hedegaard K. Escitalopram 10 mg/day is effective and well tolerated in a placebo-controlled study in depression in primary care. Int Clin Psychopharmacol. 2002;17(3):95-102.
- http://www.lundbeck.com/upload/trials/files/pdf/completed/99001_CTRS_final_30Dec2005.pdf

478. Wade 2003 (E-1721)

- Wade A, Crawford GM, Angus M, Wilson R, Hamilton L. A randomized, double-blind, 24-week study comparing the efficacy and tolerability of mirtazapine and paroxetine in depressed patients in primary care. Int Clin Psychopharmacol. 2003 May;18(3):133-41.

479. Wade 2007 (Study 10990)

- Wade A, Gembert K, Florea I. A comparative study of the efficacy of acute and continuation treatment with escitalopram versus duloxetine in patients with major depressive disorder. Curr Med Res Opin. 2007 Jul;23(7):1605-14.

480. Walczak 1996

- Walczak DD, Apter JT, Halikas JA, Borison RL, Carman JS, Post GL, Patrick R, Cohn JB, Cunningham LA, Rittberg B, Preskorn SH, Kang JS, Wilcox CS. The oral dose-effect relationship for fluvoxamine: a fixed-dose comparison against placebo in depressed outpatients. Ann Clin Psychiatry. 1996 Sep;8(3):139-51.

481. Wang 2014 (EUCTR2005-005052-40, NCT00351169, D1448C00004)

- Wang G, McIntyre A, Earley WR, Raines SR, Eriksson H. A randomized, double-blind study of the efficacy and tolerability of extended-release quetiapine fumarate (quetiapine XR) monotherapy in patients with major depressive disorder. Neuropsychiatr Dis Treat. 2014 Jan 30;10:201-16.

482. Wang 2015 (NCT01571453, KCT0000432, 13926A)

- Wang G, Gislum M, Filippov G, Montgomery S. Comparison of vortioxetine versus venlafaxine XR in adults in Asia with major depressive disorder: a randomized, double-blind study. Curr Med Res Opin. 2015 Apr;31(4):785-94.
- https://clinicaltrials.gov/ct2/show/NCT01571453

483. Weisler 1994

- Weisler RH, Johnston JA, Lineberry CG, Samara B, Branconnier RJ, Billow AA. Comparison of bupropion and trazodone for the treatment of major depression. J Clin Psychopharmacol. 1994 Jun;14(3):170-9.

484. WELL 029

- http://digitalcommons.ohsu.edu/fdadrug/21/

485. WELL AK140016

- https://www.gsk-clinicalstudyregister.com/study/AK140016#rs

486. WELL AK1A4006

- http://digitalcommons.ohsu.edu/fdadrug/21/

487. Wellbutrin 06

- http://digitalcommons.ohsu.edu/fdadrug/21/

488. Wellbutrin 25

- http://digitalcommons.ohsu.edu/fdadrug/21/

489. Wheatley 1998

- Wheatley DP, van Moffaert M, Timmerman L, Kremer CM. Mirtazapine: efficacy and tolerability in comparison with fluoxetine in patients with moderate to severe major depressive disorder. Mirtazapine-Fluoxetine Study Group. J Clin Psychiatry. 1998 Jun;59(6):306-12.

490. Wilcox 1994

- Wilcox CS, Cohn JB, Katz BB, Mijares CP, Guarino JJ, Panagides J, DeFrancisco DF. A double-blind, placebo-controlled study comparing mianserin and amitriptyline in moderately depressed outpatients. Int Clin Psychopharmacol. 1994 Winter;9(4):271-9.

491. Winokur 2003

- Winokur A, DeMartinis NA 3rd, McNally DP, Gary EM, Cormier JL, Gary KA. Comparative effects of mirtazapine and fluoxetine on sleep physiology measures in patients with major depression and insomnia. J Clin Psychiatry. 2003 Oct;64(10):1224-9.

492. Yang 2003

- Yang JC, Kim, SW, Yu BH. Milnacipran vs Sertraline in Major Depressive Disorder: A double-blind randomised comparative study on the treatment effect and β-adrenergic receptor responsiveness. Korean J Psychopharmacol. 2003;14(4):387-396.

493. Yevtushenko 2007

- Yevtushenko VY, Belous AI, Yevtushenko YG, Gusinin SE, Buzik OJ, Agibalova TV. Efficacy and tolerability of escitalopram versus citalopram in major depressive disorder: a 6-week, multicenter, prospective, randomized, double-blind, active-controlled study in adult outpatients. Clin Ther. 2007 Nov;29(11):2319-32.

494. Young 1987

- Young JP, Coleman A, Lader MH. A controlled comparison of fluoxetine and amitriptyline in depressed out-patients. Br J Psychiatry. 1987 Sep;151:337-40.

495. Zajecka 2010 (CAGO178A2301, NCT00411099)

- Zajecka J, Schatzberg A, Stahl S. Efficacy and safety of agomelatine in the treatment of major depressive disorder. J Clin Psychopharm 2010; 30: 135-44.
- https://clinicaltrials.gov/ct2/show/NCT00411099

496. Zhang 2014

- Zhang L, Xie WW, Li LH,. Efficacy and safety of prolonged-release trazodone in major depressive disorder. Pharmacology. 2014;94(5-6):199-206.

497. Zivkov 1995 (Organon 85146, FDA85146)

- Zivkov M, De Jongh GD. Org 3770 versus amitriptyline: A 6-week randomized double-blind multicentre trial in hospitalized depressed patients. Human Psychpharmacology. 1995;10(3):173-180.

498. 845

- http://digitalcommons.ohsu.edu/fdadrug/21/

499. 003-008 (FDA)

- http://digitalcommons.ohsu.edu/fdadrug/20/

500. 003-042

- http://digitalcommons.ohsu.edu/fdadrug/20/

501. 003-048

- http://digitalcommons.ohsu.edu/fdadrug/20/

502. 030A2-0004/030A2-0005 (FDA)

- http://digitalcommons.ohsu.edu/fdadrug/23/

503. 03A0A-004A (FDA)

- http://digitalcommons.ohsu.edu/fdadrug/23/

504. 0600A1-300

- Unpublished Report-Wyeth Research Philadelphia: a randomized, double-blind comparison of venlafaxine, amitriptyline, and placebo capsules in inpatients with major depression: final report (Protocol: 0600A1-300-US). 19-11-2002.

505. 0600A1-372

- Unpublished Report-Wyeth Research Philadelphia: a double-blnd, placebocontrolled, parallelgroup, comparative study of venlafaxine and fluoxetine in depressed outpatients to measure onset of clinical activity: final report (Protocol 0600A1-372-US). 6-10-1998

506. 0600A-326

- Unpublished Study Report-Wyeth-Ayerst France: a randomised, double-blind, parallel group comparison of venlafaxine and clomipramine in outpatients with major depression (Protocol 600A-326). 1991.

507. 0600A-332

- Unpublished Report-Wyeth-Ayerst USA: a randomized double-blind comparison of venlafaxine and fluoxetine in outpatients with major depression: final report (Protocol 600A-332-US). 1992.

508. 0600A-347

- Unpublished Report-Wyeth Research Philadelphia: a double-blind randomization study of the safety and efficacy of two regimens of venlafaxine compared with one regimen of fluvoxamine: final report (Protocol 0600A1-347-FR/NE). 4-3-2001.

509. 0600A-349

- Unpublished Report-Wyeth Research Philadelphia: a double-blind, randomized 8-week, comparative study of the safety and efficacy of venlafaxine and paroxetine: final report (Protocol 0600A1-349-NE/UK). 28-2-2001.

510. 0600A-626

- Unpublished Report-Wyeth-Ayerst: a randomised double-blind parallel-group comparison of the efficacy and safety of venlaflaxine versus fluoxetine in the treatment of moderately depressed outpatients (Protocol 600A-626-NL). 2007.

511. 0600A-654

- Unpublished Report-Wyeth-Ayerst: a double-blind, randomized 12-week study of the safety and eficacy of oral venlaflaxine up to 75 mg bid compared with oral fluoxetine up to 20 mg bid in patients with moderate and severe major depression (Protocol 600A-654-AU). 2007.

512. 0600B1-367

- Unpublished Study Report-Wyeth-Ayerst France: a randomized, double-blind, placebo controlled, fixed-dose study of the efficacy and safety of venlafaxine extended release and paroxetine in depressed outpatients: final report (Protocol 600B-367-EU). 1996.

513. 0600B1-384

- Unpublished Report-Wyeth-Ayerst USA: a double-blind, placebo-controlled, comparative study of an extended release formulation of venlafaxine and imipramine on the time of onset of anti-depressant response in patients with severe major depression: final report (Protocol 600B1-384-US/EU/CA). 12-9-2002.

514. 0600B1-402

- Unpublished Report-Wyeth Research Philadelphia: a double-blind, placebo-controlled, comparative efficacy study of venlafaxine ER and sertraline in producing remission in outpatients with major depressive disorder: final report (Protocol 0600B1-402-US/CA). 28-5-2003.

515. 244 (EMD 68 843-009)

- http://www.accessdata.fda.gov/drugsatfda_docs/nda/2011/022567Orig1s000MedR.pdf

516. 245 (EMD 68 843-010)

- http://www.accessdata.fda.gov/drugsatfda_docs/nda/2011/022567Orig1s000MedR.pdf

517. 246 (SB 659746-003)

- http://www.accessdata.fda.gov/drugsatfda_docs/nda/2011/022567Orig1s000MedR.pdf

518. 247 (SB 659746-014)

- http://www.accessdata.fda.gov/drugsatfda_docs/nda/2011/022567Orig1s000MedR.pdf

519. 248 (SB 659746-002)

- http://www.accessdata.fda.gov/drugsatfda_docs/nda/2011/022567Orig1s000MedR.pdf

520. 29060 07 001

- http://www.gsk-clinicalstudyregister.com/study/29060/07/001#rs

521. 29060/299

- https://www.gsk-clinicalstudyregister.com/study/29060/299#rs

522. 29060/356

- https://www.gsk-clinicalstudyregister.com/study/29060/356#rs

2 *Characteristics of included studies*

Study Name	Year	No. of arms included in NMA	No of total study arms	Drug	Sponsored	No. randomised	Mean age (SD)	% Female	Dose (min-max, mg daily)	Dosing Schedule	Mean Dose Delivered	Baseline Severity Scale	Mean Baseline Severity	Length of trial (weeks)	Diagnostic Criteria	Recruitment	Multi/ Single Centre	Treatment Setting	Patient Status	Placebo Run in	Rescue Medication
Aberg-Wisted 2000	2000	2	2	paroxetine	No	177	*	*	20-40	Flexible	*	MADRS	30.70	8	DSM-III-R	Europe	Multi-center	Unclear	Outpatients only	No	Unclear
				sertraline	No	176	*	*	50-150	Flexible	*	MADRS	30.30								
Aguglia 1993	1993	2	2	sertraline	No	52	57.5 (15.5)	*	50-150	Flexible	72	HAMD 17	24.80	8	DSM-III-R	Europe	Multi-center	Unclear	Outpatients only	Yes	Unclear
				fluoxetine	No	56	58.9 (17.3)	*	20-60	Flexible	28	HAMD 17	25.10								
AK1102365	unpublished	2	2	bupropion	Yes	166	36.8 (9.3)	0.47	200-300	Flexible	*	HAMD 17	19.40	12	*	Asia	Multi-center	Unclear	Unclear	No	Unclear
				placebo	No	159	36 (8.9)	0.44	0-0	Flexible	0	HAMD 17	19.80								
Alexopoulos 2004 (poster SCT-MD-27)	2004	3	3	escitalopram	Yes	136	39.5 (10.7)	*	10-20	Flexible	*	HAMD 24	26.47	8	DSM-IV	North America	Multi-center	Unclear	Outpatients only	Unclear	Unclear
				sertraline	No	138	40 (11.4)	*	50-200	Flexible	*	HAMD 24	26.15								
				placebo	No	135	40.6 (12.3)	*	0-0	Flexible	0	HAMD 24	27.29								
Allard 2004	2004	2	2	citalopram	No	75	72.5 (5.7)	*	20-30	Flexible	26	MADRS	27.00	8	DSM-IV	Europe	Multi-center	Unclear	Outpatients only	No	Unclear
				venlafaxine	Yes	76	73.6 (5.9)	*	75-150	Flexible	116	MADRS	27.60								
Altamura 1989b	1989	2	3	amitriptyline	Unclear	37	66.43 (5.6)	0.57	75-75	Fixed	75	HAMD 21	29.03	5	DSM-III	Europe	Multi-center	Unclear	Inpatients only	Unclear	Unclear
				trazodone	Unclear	36	64.61 (5.7)	0.61	150-150	Fixed	150	HAMD 21	29.68								
Alvarez 2012 (11492A, NCT00839423)	2012	4	4	placebo	No	105	42 (10.9)	0.66	0-0	Fixed	0	HAMD 24	29.70	6	DSM-IV	Cross-Continental	Multi-center	Secondary /Tertiary care	Outpatients only	Unclear	Yes
				vortioxetine	Yes	109	43.8 (11.6)	0.64	5-5	Fixed	5	HAMD 24	29.90								
				vortioxetine	Yes	101	42.3 (13.1)	0.65	10-10	Fixed	10	HAMD 24	29.30								
				venlafaxine	No	114	45 (10.3)	0.54	225-225	Fixed	225	HAMD24	29.40								
Alves 1999	1999	2	2	fluoxetine	No	47	42.3 (12.2)	*	20-40	Flexible	*	HAMD 21	26.90	12	DSM-IV	Europe	Multi-center	Unclear	Outpatients only	No	Unclear
				venlafaxine	Yes	40	45.4 (12)	*	75-150	Flexible	*	HAMD 21	27.90								
Amin 1984	1984	2	3	fluvoxamine	Yes	161	42 (*)	*	*	Flexible	155	HAMD unspecified	22.60	4	Feighner	Cross-Continental	Multi-center	Secondary /Tertiary care	Both	Unclear	Unclear
				placebo	No	150	42 (*)	*	0-0	Flexible	0	HAMD unspecified	22.70								

Amini 2005	2005	2	2	mirtazapine	No	18	35 (10.6)	*	30-30	Fixed	30	HAMD 17	25.80	6	DSM-IV	Asia	Single center	Secondary /Tertiary care	Both	No	Unclear
				fluoxetine	No	18	37.7 (11.3)	*	20-20	Fixed	20	HAMD 17	24.80								
Amsterdam 1986	1986	2	3	amitriptyline	No	55	41 (12)	*	200-300	Flexible	182	HAMD 21	24.5	4	RDC	North America	Single center	Secondary /Tertiary care	Outpatients only	Yes	Yes
				placebo	No	54	41 (12)	*	0-0	Flexible	0	HAMD 21	23.4								
Andersen 1986	1986	2	2	citalopram	Unclear	57	*	0.70	40-40	Fixed	40	HAMD 17	22.5	5	*	Europe	Multi-center	Secondary /Tertiary care	Inpatients only	Yes	Yes
				clomipramin e	Unclear	57	*	0.69	150-150	Fixed	150	HAMD17	22								
Ansseau 1989a	1989	3	3	milnacipran	Unclear	47	48.6 (10.9)	0.66	50-50	Fixed	50	HAMD 17	25.10	4	RDC	Europe	Multi-center	Secondary /Tertiary care	Inpatients only	Yes	Yes
				milnacipran	Unclear	48	48.6 (10.9)	0.66	100-100	Fixed	100	HAMD 17	24.40								
				amitriptyline	No	49	48.6(1 0.9)	0.66	150-150	Fixed	150	HAMD 17	24.80								
Ansseau 1989b	1989	2	2	milnacipran	Unclear	44	49.6 (11.6)	*	200-200	Fixed	200	HAMD 24	37.30	4	RDC	Europe	Multi-center	Secondary /Tertiary care	Inpatients only	Yes	Yes
				amitriptyline	No	43	49.6 (11.6)	*	150-150	Fixed	150	HAMD 24	37.60								
Ansseau 1991	1991	3	3	fluvoxamine	Unclear	41	46.6 (11.6)	*	200-200	Fixed	200	HAMD 24	32.69	4	RDC	Europe	Multi-center	Secondary /Tertiary care	Inpatients only	Yes	Yes
				milnacipran	Unclear	42	49.9 (12.4)	*	150-150	Fixed	150	HAMD 24	33.94								
				milnacipran	Unclear	43	46.5 (13.3)	*	200-200	Fixed	200	HAMD 24	31.89								
Ansseau 1994a (MY-1005/BRL-029060/1/CPM S-112)	1994	2	2	paroxetine	Yes	55	43.7 (11.5)	0.46	20-30	Flexible	*	HAMD 21	26.00	6	DSM-III-R	Europe	Multi-center	Other/Unc lear	Other/Unclea r	Yes	Unclear
				fluvoxamine	No	65	43.4 (9.9)	0.67	100-200	Flexible	*	HAMD 21	26.50								
Ansseau 1994b	1994	2	2	milnacipran	Yes	97	44.8 (11.2)	*	100-100	Fixed	100	HAMD 24	30.60	6	DSM-III-R	Europe	Multi-center	Secondary /Tertiary care	Outpatients only	Yes	Yes
				fluoxetine	No	93	45 (12.3)	*	20-20	Fixed	20	HAMD 24	32.40								
Ansseau 1994c	1994	2	2	nefazodone	Yes	55	45.8 (12.6)	0.73	100-400	Flexible	242	HAMD 17	27.70	6	DSM-III-R	Europe	Multi-center	Secondary /Tertiary care	Inpatients only	Unclear	Yes
				amitriptyline	No	51	48.6 (13.6)	0.67	50-200	Flexible	124	HAMD 17	28.10								
Armitage 1997	1997	2	2	nefazodone	Yes	22	37.1 (8.4)	0.50	400-500	Flexible	433	HAMD 17	23.50	8	DSM-III-R	North America	Multi-center	Other/Unc lear	Outpatients only	Unclear	Unclear
				fluoxetine	No	21	37.2 (10.5)	0.76	20-40	Flexible	31	HAMD 17	23.60								

Asnis 2013 (LVM-MD-01, NCT00969709)	2013	4	4	placebo	No	179	41.3 (11.3)	0.61	0-0	Fixed	0	HAMD 17	24.60	8	DSM-IV	North America	Multi-center	Unclear	Outpatients only	Yes	Yes
				levomilnacipran	Yes	181	41.6 (13.1)	0.69	40-40	Fixed	40	HAMD 17	24.70								
				levomilnacipran	Yes	181	41 (12.8)	0.62	80-80	Fixed	80	HAMD 17	24.90								
				levomilnacipran	Yes	183	40.3 (11.9)	0.59	120-120	Fixed	120	HAMD 17	25.00								
Bakish 1992	1992	2	3	amitriptyline	No	58	42 (9)	*	50-150	Flexible	112	HAMD 17	22.81	6	DSM-III-R	North America	Multi-center	Unclear	Outpatients only	Yes	Yes
				placebo	No	56	44 (10.7)	*	0-0	Flexible	0	HAMD 17	23.04								
Bakish 1997	1997	2	2	fluoxetine	No	9	39 (9)	0.62	20-80	Flexible	*	HAMD 17	22.30	12	DSM-III-R	North America	Unclear	Unclear	Other/Unclear	Unclear	Yes
				paroxetine	Yes	12	39 (9)	0.62	20-50	Flexible	*	HAMD 17	22.30								
Bakish 2014 (LVM-MD-10, NCT01377194)	2014	3	3	placebo	No	189	42.3 (13.2)	0.62	0-0	Fixed	0	MADRS	31.00	8	DSM-IV	North America	Multi-center	Unclear	Outpatients only	Yes	Unclear
				levomilnacipran	Yes	190	42.9 (13.4)	0.62	40-40	Fixed	40	MADRS	30.80								
				levomilnacipran	Yes	189	43.1 (12.8)	0.66	80-80	Fixed	80	MADRS	31.20								
Baldwin 1996	1996	2	2	nefazodone	Yes	105	38.3 (*)	0.60	200-600	Flexible	472	HAMD 17	24.60	8	DSM-III-R	Europe	Multi-center	Secondary /Tertiary care	Outpatients only	Unclear	Yes
				paroxetine	No	101	37.9 (*)	0.50	20-40	Flexible	33	HAMD 17	24.80								
Baldwin 2006a (97-CRBX-052 - Study 052)	2006	2	2	reboxetine	Yes	159	42.4 (12.1)	0.63	8-10	Flexible	9	HAMD 17	24.20	8	DSM-IV	Europe	Multi-center	Other/Unclear	Other/Unclear	Unclear	Yes
				paroxetine	No	166	45.1 (11)	0.62	20-40	Flexible	27	HAMD 17	24.10								
Baldwin 2006b (Study 99505)	2006	2	2	paroxetine	No	159	45.1 (13.2)	*	20-40	Flexible	26	MADRS	29.70	8	DSM-III-R	Europe	Multi-center	Other/Unclear	Other/Unclear	Yes	Unclear
				escitalopram	Yes	166	44.9 (14.7)	*	10-20	Flexible	14	MADRS	29.60								
Baldwin 2012 (11984A, NCT00635219)	2012	4	5	placebo	No	152	43.3 (12.5)	0.70	0-0	Fixed	0	HAMD 24	29.80	8	DSM-IV	Cross-Continental	Multi-center	Secondary /Tertiary care	Both	Unclear	Yes
				vortioxetine	Yes	159	44.7 (13.1)	0.66	5-5	Fixed	5	HAMD 24	31.30								
				vortioxetine	Yes	153	45.2 (13.1)	0.68	10-10	Fixed	10	HAMD 24	30.40								
				duloxetine	No	157	45.3 (12)	0.68	60-60	Fixed	60	HAMD 24	29.90								
Ban 1998 (Study 008)	1998	2	3	reboxetine	Unclear	84	46.4 (11.4)	0.50	8-8	Fixed	8	HAMD 17	26.89	4	DSM-III-R	Cross-Continental	Multi-center	Secondary /Tertiary care	Inpatients only	No	Yes
				placebo	No	85	46.7 (11.8)	0.64	0-0	Fixed	0	HAMD 17	25.43								

Barber 2011	2011	2	3	sertraline	No	55	38 (12.5)	*	50-200	Fixed	200	HAMD 17	19.00	16	DSM-IV	North America	Multi-center	Secondary /Tertiary care	Outpatients only	No	Unclear
				placebo	No	50	38.3 (12)	*	0-0	Fixed	0	HAMD 17	19.3								
Battegay 1985 (PAR 29060.309 HP/81/162A)	1985	2	2	paroxetine	Yes	11	41.5 (11.3)	0.55	30-30	Fixed	30	*	*	7	*	Europe	Single center	Unclear	Unclear	Unclear	Unclear
				amitriptyline	No	10	36.9 (10.8)	0.60	100-100	Fixed	100	*	*								
Beasley 1991	1991	2	2	trazodone	No	61	40 (10.8)	0.69	50-400	Flexible	244	HAMD 21	24.30	6	DSM-III	North America	Multi-center	Unclear	Outpatients only	Yes	Yes
				fluoxetine	Yes	65	40 (11.3)	0.65	20-60	Flexible	21	HAMD 21	23.40								
Behnke 2003 (E-1690, Kjernisted 2002)	2003	2	2	mirtazapine	Yes	176	42 (11)	*	30-45	Flexible	38	HAMD 17	*	8	DSM-IV	Cross-Continental	Multi-center	Both	Unclear	No	Unclear
				sertraline	No	170	41 (12)	*	50-150	Flexible	93	HAMD 17	*								
Benkert 2000 (E-1559)	2000	2	2	mirtazapine	Yes	139	47.2 (11.1)	*	15-45	Flexible	33	HAMD 17	22.40	6	DSM-IV	Europe	Multi-center	Both	Outpatients only	Yes	Unclear
				paroxetine	No	136	47.3 (10.3)	*	20-40	Flexible	23	HAMD 17	22.40								
Benkert 2006(C-1763)	2006	2	2	mirtazapine	Yes	140	46.4 (11.1)	0.56	45-45	Fixed	45	HAMD 17	24.60	6	DSM-IV	Europe	Multi-center	Unclear	Outpatients only	No	Unclear
				venlafaxine	No	135	45.1 (12.7)	0.59	225-225	Fixed	225	HAMD17	24.90								
Bennie 1995	1995	2	2	sertraline	Yes	142	49.9 (*)	*	50-100	Flexible	62	HAMD 17	23.20	6	DSM-III-R	Europe	Multi-center	Secondary /Tertiary care	Outpatients only	Yes	Yes
				fluoxetine	No	144	49.9 (*)	*	20-40	Flexible	25	HAMD 17	23.40								
Berlanga 1997	1997	2	2	nefazodone	Yes	37	41.5 (12.8)	0.78	400-500	Flexible	400	HAMD 17	25.10	8	DSM-III-R	South America	Multi-center	Unclear	Outpatients only	Unclear	Yes
				fluoxetine	No	37	39.9 (13.4)	0.76	20-40	Flexible	24	HAMD 17	23.70								
Berlanga 2006	2006	2	2	reboxetine	No	46	29.2 (6)	*	4-8	Flexible	*	HAMD 21	26.99	8	DSM-IV	South America	Single center	Secondary /Tertiary care	Outpatients only	No	No
				citalopram	Yes	55	29.65 (7.3)	*	20-40	Flexible	*	HAMD21	27.15								
Bersani 1994	1994	2	2	sertraline	Unclear	34	47.2 (12)	0.63	50-150	Flexible	88	HAMD 21	33	8	DSM-III-R	Europe	Unclear	Unclear	Outpatients only	Unclear	No
				amitriptyline	Unclear	34	46.9 (12)	0.63	50-150	Flexible	84	HAMD 21	32								
Bhatia 1991	1991	2	3	amitriptyline	No	7	38 (15.1)	*	200-300	Flexible	250	HAMD 17	32.92	6	DSM-III	North America	Multi-center	Secondary /Tertiary care	Inpatients only	No	Unclear
				placebo	No	8	45.6 (10.2)	*	0-0	Flexible	0	HAMD 17	32.92								
Bielski 2004 (SCT-MD-12)	2004	2	2	escitalopram	Yes	101	37.3 (12.3)	*	20-20	Fixed	20	HAMD 24	28.60	8	DSM-IV	North America	Multi-center	Secondary /Tertiary care	Outpatients only	Yes	Unclear
				venlafaxine	No	101	37.5 (11.6)	*	225-225	Fixed	225	HAMD 24	27.40								

Bignamini 1992 (PAR 29060/078)	1992	2	2	paroxetine	Yes	156	49.1 (13.8)	*	20-30	Flexible	*	HAMD 21	30.50	6	DSM-III	Europe	Multi-center	Unclear	Outpatients only	Unclear	Unclear
				amitriptyline	No	153	49.8 (11.9)	*	75-150	Flexible	*	HAMD 21	30.90								
Binnemann 2008 (NCT00143091)	2008	2	3	sertraline	Yes	43	49 (*)	*	100-100	Fixed	100	HAMD 17	23.30	6	DSM-IV	Cross-Continental	Multi-center	Unclear	Outpatients only	Yes	Unclear
				placebo	No	39	49 (*)	*	0-0	Fixed	0	HAMD 17	23.55								
Bjerkenstedt 2005	2005	2	3	fluoxetine	No	57	50.4 (11.6)	*	20-20	Fixed	20	HAMD 21	23.80	6	DSM-IV	Europe	Multi-center	Primary care	Outpatients only	Yes	Unclear
				placebo	No	58	51.4 (11.8)	*	0-0	Fixed	0	HAMD 21	25.20								
Blacker 1988	1988	2	4	amitriptyline	No	44	42 (12.5)	*	100-100	Fixed	100	HAMD 21	26.70	6	DSM-III	Europe	Multi-center	Primary care	Outpatients only	Unclear	Unclear
				trazodone	Unclear	112	45 (12.8)	*	150-150	Fixed	150	HAMD 21	26.30								
Bosc 1997a (Study 014 - Andreoli2002)	1997	3	3	reboxetine	Yes	126	40 (12)	0.67	8-10	Flexible	*	HAMD 21	26.80	8	DSM-III-R	Cross-Continental	Multi-center	Unclear	Outpatients only	No	Yes
				placebo	No	128	44.1 (12.1)	0.54	0-0	Flexible	0	HAMD 21	27.40								
				fluoxetine	No	127	40.2 (11.5)	0.65	20-40	Flexible	*	HAMD 21	26.90								
Bosc 1997b (Study 016 - Massana 1999)	1997	2	2	reboxetine	Yes	79	44 (12.6)	0.72	8-10	Flexible	8	HAMD 21	28.60	8	DSM-III-R	Cross-Continental	Multi-center	Unclear	Both	Unclear	Yes
				fluoxetine	No	89	43.6 (11.8)	0.72	20-40	Flexible	23	HAMD 21	27.40								
Bose 2008 (SCT-MD-13)	unpublished	2	2	escitalopram	Yes	132	68.1 (6.7)	*	10-20	Flexible	*	HAMD unspecified	24.90	12	DSM-IV	North America	Multi-center	Unclear	Outpatients only	Unclear	Unclear
				placebo	No	135	68.5 (7.1)	*	0-0	Flexible	0	HAMD unspecified	24.30								
Bougerol 1997 (Study 91302 - FDA)	1997	2	2	fluoxetine	No	158	41 (*)	*	20-20	Fixed	20	MADRS	31.80	8	DSM-III-R	Europe	Multi-center	Secondary /Tertiary care	Other/Unclear	No	Yes
				citalopram	Yes	158	43 (*)	*	40-40	Fixed	40	MADRS	31.20								
Boulenger 2006 (Study 10351)	2006	2	2	escitalopram	Yes	232	43.8 (12.5)	*	20-20	Fixed	20	MADRS	35.20	24	DSM-IV	Europe	Multi-center	Both	Outpatients only	No	Unclear
				paroxetine	Yes	227	44.7 (13)	*	40-40	Fixed	40	MADRS	34.80								
Boulenger 2014 (13267A, NCT01140906)	2014	4	4	placebo	No	158	48.1 (13.1)	0.70	0-0	Fixed	0	MADRS	31.50	8	DSM-IV	Cross-Continental	Multi-center	Secondary /Tertiary care	Both	Unclear	Yes
				vortioxetine	Yes	152	47 (14.6)	0.64	15-15	Fixed	15	MADRS	31.80								
				vortioxetine	Yes	151	46.2 (13.4)	0.60	20-20	Fixed	20	MADRS	31.20								
				duloxetine	No	147	45.6 (13.6)	0.69	60-60	Fixed	60	MADRS	31.20								
Boyer 2008 (Study 333, NCT00300378)(EUCTR2005-	2008	3	3	desvenlafaxine	Yes	166	44 (14)	0.70	50-50	Fixed	50	HAMD 17	24.26	8	DSM-IV	Cross-Continental	Multi-center	Both	Outpatients only	Unclear	Unclear
				desvenlafaxine	Yes	158	46 (13)	0.71	100-100	Fixed	100	HAMD 17	24.44								

005463-28, 3151A1-333-EU)				placebo	No	161	46 (12)	0.68	0-0	Fixed	0	HAMD 17	24.29								
Bremner 1995(MIR 003-022 - FDA)	1995	3	3	amitriptyline	No	50	39 (*)	*	40-280	Flexible	187	HAMD 21	32	6	DSM-III	North America	Single center	Secondary /Tertiary care	Outpatients only	Yes	Yes
				placebo	No	50	37 (*)	*	0-0	Flexible	0	HAMD 21	31.2								
				mirtazapine	Yes	50	39 (*)	*	5-35	Flexible	31	HAMD 21	33								
Brown 1986	1986	2	3	fluvoxamine	Unclear	33	33.4 (*)	*	200-300	Flexible	214	HAMD 21	*	6	DSM-III	North America	Multi-center	Secondary /Tertiary care	Outpatients only	Unclear	Unclear
				placebo	No	31	*	*	0-0	Flexible	0	HAMD 21	*								
Brunoni 2012	2012	2	4	sertraline	No	30	41 (12)	*	50-50	Fixed	50	MADRS	30.5	6	DSM-IV	South America	Single center	Secondary / Tertiary care	Outpatients only	No	Yes
				placebo	No	30	46.4 (14)	*	0-0	Fixed	0	MADRS	30.76								
Buchsbaum 1997	1997	2	2	sertraline	Yes	7	38.5 (12.3)	0.59	*	Other/Unclear	*	HAMD 24	32.00	10	DSM-III-R	North America	Unclear	Secondary /Tertiary care	Unclear	Yes	Unclear
				placebo	No	10	38.5 (12.3)	0.59	0-0	Other/Unclear	0	HAMD 24	29.45								
Bueno 1997	1997	2	2	nefazodone	Unclear	19	46.7 (10.5)	0.79	200-500	Flexible	*	HAMD unspecified	*	8	DSM-III-R	South America	Unclear	Unclear	Outpatients only	Yes	Unclear
				amitriptyline	Unclear	18	45.2 (12.2)	0.89	50-125	Flexible	*	HAMD unspecified	*								
Burke 2002 (SCT-MD-01)	2002	4	4	escitalopram	Yes	124	40.6 (12.3)	*	10-10	Fixed	10	HAMD unspecified	24.30	8	DSM-IV	North America	Multi-center	Unclear	Outpatients only	Yes	Unclear
				escitalopram	Yes	128	39.6 (12.1)	*	20-20	Fixed	20	HAMD unspecified	25.80								
				citalopram	Yes	127	40 (11.5)	*	40-40	Fixed	40	HAMD unspecified	25.90								
				placebo	No	127	40.3 (10.6)	*	0-0	Fixed	0	HAMD unspecified	25.80								
Byerley 1988	1988	2	3	placebo	No	29	37.5 (9.1)	*	0-0	Flexible	0	HAMD 21	27.40	6	DSM-III	North America	Multi-center	Secondary /Tertiary care	Outpatients only	Yes	Yes
				fluoxetine	Yes	32	38.9 (10.7)	*	40-80	Flexible	*	HAMD 21	27.10								
CAGO178A2303 (NCT00463242)	unpublished	3	3	agomelatine	Yes	169	42.1 (13.1)	0.62	25-50	Flexible	*	HAMD 17	27.20	8	DSM-IV	North America	Multi-center	Unclear	Unclear	Unclear	Unclear
				paroxetine	No	168	43.7 (12.7)	0.59	20-40	Flexible	*	HAMD 17	27.00								
				placebo	No	166	42.9 (11.8)	0.67	0-0	Flexible	0	HAMD 17	26.90								
Caligiuri 2003	2003	2	3	bupropion	No	7	42 (9.8)	*	*	Flexible	400	HAMD 17	18.40	9	DSM-IV	North America	Single center	Secondary /Tertiary care	Outpatients only	Unclear	Unclear
				sertraline	No	9	42 (9.8)	*	50-*	Flexible	165	HAMD 17	18.40	8							

Carman 1991	1991	2	3	amitriptyline	No	50	*	*	60-300	Flexible	*	HAMD 17	27.6	6	DSM-III	North America	Unclear	Unclear	Outpatients only	Yes	Unclear
				placebo	No	50	*	*	0-0	Flexible	0	HAMD 17	26.7								
Casabona 2002 (0600B 428)	2002	2	2	paroxetine	No	56	*	*	20-20	Fixed	20	*	*	8	DSM-IV	Europe	Multi-center	Unclear	Outpatients only	Unclear	Unclear
				venlafaxine	Yes	58	*	*	75-75	Fixed	75	*	*								
Cassano 1986	1986	2	3	fluvoxamine	Yes	161	42.7 (13.7)	*	50-300	Flexible	145	HAMD 17	25.61	4	DSM-III	Cross-Continental	Multi-center	Unclear	Both	Yes	Yes
				placebo	No	149	42.1 (11.8)	*	0-0	Flexible	0	HAMD 17	25.60								
Cassano 2002 (29060/421)	2002	2	2	paroxetine	Yes	123	75.61 (6.99)	*	20-40	Flexible	*	HAMD unspecified	22.95	6	ICD-10	Europe	Multi-center	Unclear	Outpatients only	Yes	Unclear
				fluoxetine	No	119	74.85 (6.67)	*	20-60	Flexible	*	HAMD unspecified	23.41								
Chang 2015	2015	2	2	fluoxetine	No	58	41.7 (12)	0.76	20-80	Flexible	21	HAMD 21	24.30	6	DSM-IV	Asia	Unclear	Other/Unclear	Outpatients only	Unclear	Yes
				venlafaxine	No	54	37.5 (12.5)	0.70	75-225	Flexible	80	HAMD 21	25.90								
Chouinard 1983a	1983	2	2	amitriptyline	Unclear	41	35.1 (*)	*	75-150	Flexible	119	HAMD 21	26.10	13	Other operationalized	North America	Multi-center	Unclear	Outpatients only	Yes	Yes
				bupropion	Unclear	77	38.9 (*)	*	300-450	Flexible	374	HAMD 21	26.50								
Chouinard 1985	1985	2	2	fluoxetine	Unclear	25	41 (*)	*	40-80	Flexible	69	HAMD 21	27.60	5	RDC	North America	Multi-center	Secondary /Tertiary care	Outpatients only	Yes	Yes
				amitriptyline	Unclear	28	39 (*)	*	150-300	Flexible	163	HAMD 21	25.90								
Chouinard 1999 (29060/131)	1999	2	2	paroxetine	Yes	102	40.6 (10.7)	*	20-50	Flexible	26	HAMD 21	25.91	12	DSM-III-R	North America	Multi-center	Unclear	Unclear	Yes	Unclear
				fluoxetine	No	101	41.2 (10.7)	*	20-80	Flexible	28	HAMD 21	25.45								
Christiansen 1996	1996	2	2	paroxetine	Unclear	71	*	*	20-40	Flexible	*	HAMD 17	23.8	8	*	Europe	Multi-center	Unclear	Outpatients only	Unclear	Unclear
				amitriptyline	Unclear	73	*	*	100-150	Flexible	*	HAMD 17	24.2								
CL3-20098-022	2001	3	3	agomelatine	Yes	133	41 (9.7)	0.67	25-25	Fixed	25	HAMD 17	27.60	24	DSM-IV	Europe	Multi-center	Secondary /Tertiary care	Both	Yes	No
				fluoxetine	no	137	42.9 (11.3)	0.67	20-20	Fixed	20	HAMD 17	27.50								
				placebo	No	149	43 (9.5)	0.67	0-0	Fixed	0	HAMD 17	28.00								
CL3-20098-023	2001	3	3	agomelatine	Yes	142	40.6 (10.8)	0.75	25-25	Fixed	25	HAMD 17	25.70	24	DSM-IV	Cross-Continental	Multi-center	Secondary /Tertiary care	Both	No	No
				paroxetine	No	138	40.9 (11)	0.75	20-20	Fixed	20	HAMD 17	26.10								
				placebo	No	137	41.2 (10.1)	0.75	0-0	Fixed	0	HAMD 17	26.00								

CL3-20098-024	2002	3	4	agomelatine	Yes	301	40.4 (11.1)	0.73	25-50	Fixed	38	HAMD 17	26.40	24	DSM-IV	Cross-Continental	Multi-center	Secondary /Tertiary care	Both	No	Unclear
				fluoxetine	No	148	41.3 (10)	0.73	20-20	Fixed	20	HAMD 17	26.50								
				placebo	No	158	41.6 (11.2)	0.73	0-0	Fixed	0	HAMD 17	26.90								
CL3-20098-026	2001	2	2	agomelatine	Yes	109	66.8 (5.3)	*	25-25	Fixed	25	HAMD 17	23.40	24	DSM-IV	Cross-Continental	Multi-center	Secondary /Tertiary care	Both	No	No
				placebo	No	109	67.8 (5.1)	*	0-0	Fixed	0	HAMD 17	23.00								
CL3-20098-036	2004	2	2	agomelatine	Yes	137	39.6 (10.3)	0.72	50-50	Fixed	50	MADRS	27.90	24	DSM-IV	Cross-Continental	Multi-center	Primary care	Outpatients only	No	No
				venlafaxine	No	140	42.1 (10)	0.72	75-150	Flexible	*	MADRS	27.90								
CL3-20098-048	2008	2	2	agomelatine	Yes	213	68.6 (6.2)	*	25-50	Flexible	*	HAMD 17	26.30	24	DSM-IV	Cross-Continental	Multi-center	Primary care	Outpatients only	No	No
				paroxetine	No	199	68.3 (6.2)	*	20-30	Flexible	*	HAMD 17	26.20								
CL3-20098-062	2010	2	2	agomelatine	Yes	202	43 (12.3)	*	25-50	Flexible	*	HAMD 17	26.20	25	DSM-IV	Cross-Continental	Multi-center	Primary care	Outpatients only	No	No
				duloxetine	No	216	42.5 (11.9)	*	60-60	Fixed	60	HAMD 17	26.30								
CL3-20098-070	2011	2	2	agomelatine	Yes	151	71.9 (5.1)	*	25-50	Flexible	*	HAMD 17	26.80	24	DSM-IV	Cross-Continental	Multi-center	Primary care	Outpatients only	No	No
				placebo	No	71	71.7 (4.8)	*	0-0	Flexible	0	HAMD 17	26.70								
Claghorn 1983	1983	2	3	amitriptyline	No	85	39 (12.7)	*	75-300	Flexible	180	HAMD 21	26.51	4	RDC	North America	Multi-center	Secondary /Tertiary care	Outpatients only	Yes	Yes
				placebo	No	87	39.1 (12.5)	*	0-0	Flexible	0	HAMD 21	27.11								
Claghorn 1995 (MIR 003-002 (FDA)	1995	2	2	mirtazapine	Yes	45	39 (*)	*	*-35	Flexible	17	HAMD 21	24.20	6	DSM-III	North America	Single center	Secondary /Tertiary care	Outpatients only	Yes	Yes
				placebo	No	45	40 (*)	*	0-0	Flexible	0	HAMD 21	24.70								
Claghorn 1996	1996	2	3	fluvoxamine	Yes	50	39 (10.9)	*	50-150	Flexible	93	HAMD 21	26.09	6	DSM-III-R	North America	Single center	Unclear	Outpatients only	Yes	Unclear
				placebo	No	50	39 (10.9)	*	0-0	Flexible	0	HAMD 21	26.42	7							
Clayton 2003 (Study 050)	2003	3	3	reboxetine	Yes	150	39.8 (11.4)	0.63	8-10	Flexible	10	HAMD 21	25.60	8	DSM-IV	North America	Multi-center	Unclear	Outpatients only	Yes	Yes
				placebo	No	150	39.8 (11.1)	0.60	0-0	Flexible	0	HAMD 21	25.50								
				fluoxetine	No	150	40.7 (10.6)	0.66	20-40	Flexible	36	HAMD 21	26.00								
Clayton 2006a (WELL AK130926)	2006	3	3	bupropion	Yes	138	37 (12.5)	*	300-450	Flexible	309	HAMD 17	*	8	DSM-IV	North America	Multi-center	Unclear	Outpatients only	Unclear	Yes
				escitalopram	Unclear	149	35.7 (12.1)	*	10-20	Flexible	13	HAMD 17	*								
				placebo	No	137	37 (10.7)	*	0-0	Flexible	0	HAMD 17	*								

Clayton 2006b (WELL AK130927)	2006	3	3	escitalopram	Unclear	138	35.4 (10.4)	*	10-20	Flexible	13	HAMD 17	23.80	8	DSM-IV	North America	Multi-center	Unclear	Outpatients only	Unclear	Yes
				bupropion	Yes	141	36.5 (12.2)	*	300-450	Flexible	323	HAMD 17	24.50								
				placebo	No	141	35.1 (11.8)	*	0-0	Flexible	0	HAMD 17	24.40								
Clayton 2013 (NCT01121484)	2013	2	2	desvenlafaxine	Yes	218	53.2 (6.8)	1.00	50-50	Fixed	50	HAMD 17	22.8	8	DSM-IV	North America	Multi-center	Secondary /Tertiary care	Outpatients only	No	Yes
				placebo	No	221	52.8 (6.6)	1.00	0-0	Fixed	0	HAMD 17	22.4								
Clayton 2015	2015	3	3	placebo	No	306	41.7 (12.4)	0.58	0-0	Fixed	0	HAMD 17	23.6	8	DSM-IV	North America	Multi-center	Unclear	Outpatients only	Unclear	Unclear
				desvenlafaxine	Yes	306	41.8 (13.6)	0.57	50-50	Fixed	50	HAMD 17	23.4								
				desvenlafaxine	Yes	312	41.3 (12.8)	0.53	100-100	Fixed	100	HAMD 17	23.5								
Clerc 1994	1994	2	2	fluoxetine	No	34	53.6 (*)	*	40-40	Fixed	40	HAMD 21	29.70	6	DSM-III-R	Europe	Multi-center	Secondary /Tertiary care	Inpatients only	Yes	Unclear
				venlafaxine	Yes	34	49 (*)	*	200-200	Fixed	200	HAMD 21	29.10								
Clerc 2001	2001	2	2	fluvoxamine	No	56	52.2 (12.6)	*	200-200	Fixed	200	HAMD 24	31.40	6	DSM-III-R	Europe	Multi-center	Secondary /Tertiary care	Both	Yes	Yes
				milnacipran	Yes	57	48.7 (15.1)	*	100-100	Fixed	100	HAMD 24	32.80								
CN104-002 (FDA)	unpublished	2	3	nefazodone	Yes	60	38.2 (*)	0.57	50-300	Flexible	263	HAMD 17	23.30	6	DSM-III-R	North America	Multi-center	Secondary /Tertiary care	Outpatients only	Unclear	Yes
				placebo	No	60	38.6 (*)	0.65	0-0	Flexible	0	HAMD 17	23.10								
CN104-045 (FDA)	unpublished	2	2	nefazodone	Yes	*	*	0.63	100-400	Other/Unclear	*	*	*	6	Unclear	North America	Multi-center	Unclear	Outpatients	Unclear	Unclear
				placebo	No	*	*	0.63	0-0	Other/Unclear	0	*	*								
CN104-054 (FDA)	unpublished	3	3	nefazodone	Yes	*	*	0.68	100-600	Fixed	*	*	*	8	Unclear	North America	Multi-center	Unclear	Outpatients	Unclear	Unclear
				fluoxetine	No	*	*	0.68	20-20	Fixed	20	*	*								
				placebo	No	*	*	0.68	0-0	Fixed	0	*	*								
Cohn 1985a	1985	2	3	fluoxetine	Yes	54	41 (*)	0.72	20-80	Flexible	*	HAMD unspecified	25.75	6	DSM-III	North America	Single center	Unclear	Outpatients only	Yes	Unclear
				placebo	No	58	44 (*)	0.48	0-0	Flexible	0	HAMD unspecified	25.14								
Cohn 1990	1990	2	2	sertraline	Unclear	161	70.2 (*)	0.50	50-200	Flexible	116	HAMD 17	23.7	8	DSM-III	North America	Multi-center	Secondary /Tertiary care	Outpatients only	Yes	Yes
				amitriptyline	No	80	70.6 (*)	0.46	50-150	Flexible	88	HAMD 17	25.2								
Cohn 1996 (FDA CN104-006-2)	1996	2	3	nefazodone	Yes	43	39.4 (13.1)	0.77	200-600	Flexible	321	HAMD 17	22.80	8	DSM-III-R	North America	Single center	Unclear	Outpatients only	Unclear	No
				placebo	No	43	38.8 (12.5)	0.64	0-0	Flexible	0	HAMD 17	23.40								

Coleman 1999 (AK1A4002)	1999	3	3	sertraline	No	118	38.3 (11.7)	*	50-200	Flexible	106	HAMD 31	34.80	8	DSM-IV	North America	Multi-center	Secondary /Tertiary care	Outpatients only	Unclear	No
				placebo	No	124	38.5 (10.6)	*	0-0	Flexible	0	HAMD 31	34.00								
				bupropion	Yes	122	38.1 (10.4)	*	150-400	Flexible	290	HAMD 31	34.50								
Coleman 2001 (AK1A4007)	2001	3	3	fluoxetine	No	154	37.1 (10.6)	*	20-60	Flexible	26	HAMD 21	24.50	8	DSM-IV	North America	Multi-center	Unclear	Outpatients only	No	Unclear
				bupropion	Yes	150	36.6 (10.5)	*	150-400	Flexible	319	HAMD 21	24.60								
				placebo	No	152	36.7 (10.7)	*	0-0	Flexible	0	HAMD 21	24.40								
Colonna 2005 (Study 99022)	2005	2	2	escitalopram	Yes	175	46 (12)	*	10-10	Fixed	10	MADRS	29.50	8	DSM-IV	Europe	Multi-center	Primary care	Outpatients only	Yes	Unclear
				citalopram	No	182	46 (11)	*	20-20	Fixed	20	MADRS	30.20								
Corrigan 2000	2000	2	5	fluoxetine	No	35	42 (*)	*	20-20	Fixed	20	HAMD 17	22.00	8	DSM-III-R	North America	Multi-center	Secondary /Tertiary care	Unclear	Yes	Unclear
				placebo	No	35	42 (*)	*	0-0	Fixed	0	HAMD 17	20.80								
Corruble 2013 (CL3-20098-063)	2008	2	2	agomelatine	Yes	164	43.6 (12.9)	0.73	25-50	Flexible	*	HAMD 17	26.80	25	DSM-IV	Cross-Continental	Multi-center	Primary care	Outpatients only	No	No
				escitalopram	No	160	42.8 (11.8)	0.69	10-20	Flexible	*	HAMD 17	26.60								
Costaesilva 1998	1998	2	2	fluoxetine	No	186	39.8 (10.3)	*	20-40	Flexible	*	HAMD 17	29.70	8	DSM-III-R	South America	Multi-center	Unclear	Outpatients only	No	Unclear
				venlafaxine	Yes	196	40.5 (10.7)	*	75-150	Flexible	*	HAMD 17	30.40								
Croft 1999 (AK1A4001)	1999	3	3	sertraline	No	119	36 (9.8)	*	50-200	Flexible	121	HAMD 29	33.10	8	DSM-IV	North America	Multi-center	Unclear	Unclear	No	No
				bupropion	Yes	120	35.9 (10.8)	*	150-400	Flexible	293	HAMD 29	32.90								
				placebo	No	121	37.4 (10.9)	*	0-0	Flexible	0	HAMD 29	32.20								
Croft 2014 (NCT01473394)	2014	2	2	vilazodone	Yes	260	39.3 (12.8)	0.51	40-40	Fixed	40	MADRS	30.60	8	DSM-IV	North America	Multi-center	Unclear	Outpatients only	No	Yes
				placebo	No	258	41.1 (13.2)	0.56	0-0	Fixed	0	MADRS	30.90								
Cunningham 1994 (VEN 600A-302 FDA)	1994	3	3	venlafaxine	Yes	76	42 (*)	0.63	75-200	Flexible	160	HAMD 21	25.02	6	DSM-III-R	North America	Multi-center	Unclear	Outpatients only	Yes	Yes
				trazodone	No	77	39 (*)	0.69	150-400	Flexible	300	HAMD 21	24.66								
				placebo	No	76	41 (*)	0.67	0-0	Flexible	0	HAMD 21	24.41								
Cunningham 1997 (VEN XR 208 - FDA)	1997	3	3	venlafaxine	Yes	97	39.7 (11)	0.63	75-150	Flexible	139	HAMD 21	24.40	12	DSM-III-R	North America	Multi-center	Unclear	Outpatients only	Yes	Yes
				venlafaxine	Yes	96	42.8 (11.4)	0.67	75-150	Flexible	123	HAMD 21	23.98								
				placebo	No	100	39.9 (10.1)	0.59	0-0	Flexible	0	HAMD 21	24.56								

Cutler 2009	2009	2	3	duloxetine	No	151	40.2 (12.5)	*	60-60	Fixed	60	HAMD 17	25.20	8	DSM-IV	North America	Multi-center	Unclear	Outpatients only	Unclear	Yes
				placebo	No	157	42.3 (11.5)	*	0-0	Fixed	0	HAMD 17	25.20								
Dalery 2003	2003	2	2	fluvoxamine	Yes	90	42 (*)	*	100-100	Fixed	100	HAMD 17	22.30	6	DSM-III-R	Europe	Multi-center	Secondary /Tertiary care	Outpatients only	Yes	Yes
				fluoxetine	No	94	42.1 (*)	*	20-20	Fixed	20	HAMD 17	22.20								
D'Amico 1990 (FDA 030-A2-0007)	1990	3	5	nefazodone	Yes	47	*	*	200-200	Fixed	200	HAMD 17	26.10	6	RDC	North America	Multi-center	Unclear	Outpatients only	Unclear	Unclear
				nefazodone	Yes	47	*	*	300-300	Fixed	300	HAMD 17	25.40								
				placebo	No	47	*	*	0-0	Fixed	0	HAMD 17	26.40								
Davidson 2002 (HDTSG) (NCT00005013)	2002	2	3	sertraline	Unclear	111	43.9 (13.9)	0.67	50-100	Flexible	75	HAMD 17	22.50	8	DSM-IV	North America	Multi-center	Secondary /Tertiary care	Outpatients only	Yes	Yes
				placebo	No	116	40.1 (12.2)	0.66	0-0	Flexible	0	HAMD 17	22.70								
Debonnel 2000 (Blier 2009, iQWIG E-1639)	2009	2	3	mirtazapine	Yes	21	45.9 (9.1)	0.29	30-45	Flexible	*	HAMD 17	24.00	6	DSM-IV	North America	Single center	Unclear	Unclear	Yes	Yes
				paroxetine	No	21	39.7 (11.8)	0.48	20-30	Flexible	*	HAMD 17	24.20								
Debus 1988	1988	2	2	fluoxetine	Yes	22	35.4 (8.6)	*	20-60	Flexible	31	HAMD 21	23.40	6	DSM-III	North America	Multi-center	Both	Outpatients only	Yes	No
				trazodone	No	21	39.3 (8.3)	*	50-400	Flexible	305	HAMD 21	25.20								
DeMartinis 2007 (Study 306, NCT00072774)	2007	2	4	placebo	No	120	40 (12.8)	0.68	0-0	Fixed	0	HAMD 17	23.1	8	DSM-IV	North America	Multi-center	Unclear	Outpatients only	Unclear	Unclear
				desvenlafaxine	Yes	118	40.4 (12.1)	0.65	100-100	Fixed	100	HAMD 17	23.2								
Demyttenaere 1998	1998	2	2	amitriptyline	No	31	42.1 (8.1)	*	150-150	Fixed	150	HAMD unspecified	23.90	9	DSM-III-R	Europe	Multi-center	Secondary /Tertiary care	Outpatients only	Unclear	Unclear
				fluoxetine	Yes	35	41.4 (8.9)	*	20-20	Fixed	20	HAMD unspecified	24.90								
DeRonchi 1998	1988	2	2	fluoxetine	Unclear	32	69.1 (5.92)	*	20-20	Fixed	20	HAMD 17	25.60	10	DSM-III-R	Europe	Multi-center	Secondary /Tertiary care	Outpatients only	Yes	Yes
				amitriptyline	Unclear	33	68.6 (7.64)	*	50-100	Fixed	100	HAMD 17	26.50								
Detke 2002a (HMBH - Study Group A)	2002	2	2	duloxetine	Yes	123	42.44 (13.7)	*	60-60	Fixed	60	HAMD 17	21.50	8	DSM-IV	North America	Multi-center	Unclear	Outpatients only	Yes	Unclear
				placebo	No	122	42.34 (12.6)	*	0-0	Fixed	0	HAMD 17	21.09								
Detke 2002b (HMBH - Study Group B)	2002	2	2	duloxetine	Yes	128	40.83 (12.6)	*	60-60	Fixed	60	HAMD 17	20.28	8	DSM-IV	North America	Multi-center	Unclear	Outpatients only	Yes	Unclear
				placebo	No	139	41.04 (14.7)	*	0-0	Fixed	0	HAMD 17	20.49								
Detke 2004 (HMAY Study Group A)	2004	4	4	duloxetine	Yes	95	43.09 (11.1)	*	80-80	Fixed	80	HAMD 17	19.88	8	DSM-IV	Unspecified	Multi-center	Unclear	Outpatients only	Yes	Unclear
				duloxetine	Yes	93	44.72 (10.7)	*	120-120	Fixed	120	HAMD 17	20.17								

				paroxetine	No	86	42 (10.6)	*	20-20	Fixed	20	HAMD 17	20.26								
				placebo	No	93	43.67 (12.2)	*	0-0	Fixed	0	HAMD 17	19.86								
Deushle 2003 (NCT01049347)	2003	2	2	amitriptyline	No	14	49.5 (17.5)	0.64	150-150	Fixed	150	HAMD unspecified	21.64	5	DSM-IV	Europe	Unclear	nclear	Inpatients only	Unclear	Yes
				paroxetine	No	21	61.2 (13)	0.67	40-40	Fixed	40	HAMD unspecified	23.62								
DeWilde 1983a	1983	2	2	fluvoxamine	Yes	22	*	*	100-300	Flexible	300	HAMD 17	23.40	6	Feighner	Europe	Single center	Secondary /Tertiary care	Outpatients only	Unclear	Yes
				clomipramine	No	21	*	*	50-150	Flexible	144	HAMD 17	24.20								
DeWilde 1993	1993	2	2	paroxetine	Yes	49	43.9 (13.2)	*	20-40	Flexible	*	HAMD 21	27.00	6	DSM-III	Europe	Multi-center	Other/Unclear	Other/Unclear	Yes	Unclear
				fluoxetine	No	50	44.3 (12)	*	20-60	Flexible	*	HAMD 21	28.20								
Dierick 1996	1996	2	2	fluoxetine	No	161	43.2 (13)	0.64	20-20	Fixed	20	HAMD 17	26.60	8	DSM-III-R	Europe	Multi-center	Unclear	Outpatients only	Unclear	Unclear
				venlafaxine	Yes	153	43.7 (12.5)	0.65	75-150	Flexible	112	HAMD 17	27.00								
Dimidjian 2006	2006	2	4	paroxetine	No	100	39.9 (*)	0.68	*-50	Flexible	32	HAMD 17	20.87	16	DSM-IV	North America	Unclear	Secondary /Tertiary care	Outpatients only	No	Unclear
				placebo	No	53	39.9 (*)	0.72	0-0	Flexible	0	HAMD 17	21.15								
Dominguez 1985	1985	2	3	fluvoxamine	Unclear	35	*	*	100-300	Flexible	*	HAMD 17	20.40	4	DSM-III	North America	Single center	Secondary /Tertiary care	Outpatients only	Yes	Unclear
				placebo	No	31	*	*	0-0	Flexible	0	HAMD 17	20.90								
Doogan 1994	1994	2	3	sertraline	Yes	99	46 (*)	0.71	50-100	Flexible	62	MADRS	27.9	6	DSM-III-R	Europe	Multi-center	Primary care	Outpatients only	Yes	Yes
				placebo	No	101	45.4 (*)	0.64	0-0	Flexible	0	MADRS	27.4								
DUAG 1990	1990	2	2	paroxetine	Unclear	62	*	*	30-30	Fixed	30	HAMD 17	23.12	6	DSM-III	Europe	Multi-center	Secondary /Tertiary care	Inpatients only	Yes	Yes
				clomipramine	Unclear	58	*	*	150-150	Fixed	150	HAMD 17	23.36								
Dube 2010 (NCT00420004)	2010	2	3	escitalopram	No	62	34.3 (9.7)	0.36	10-20	Flexible	15	HAMD21	*	8	DSM-IV	Cross-Continental	Multi-center	Secondary /Tertiary care	Outpatients only	Unclear	Yes
				placebo	No	138	37.5 (11)	0.47	0-0	Flexible	0	HAMD 21	*								
Dunbar 1993a (Claghorn 1992, Rickels 1989, Rickels 1992, PAR 02-001 - FDA)	1993	2	2	paroxetine	Yes	55	43.4 (*)	0.69	20-50	Flexible	*	HAMD 21	26.80	6	DSM-III	North America	Single center	Secondary /Tertiary care	Outpatients only	Yes	Unclear
				placebo	No	56	46 (*)	0.59	0-0	Flexible	0	HAMD 21	25.90								
Dunbar 1993b (Claghorn 1992,	1993	2	2	paroxetine	Yes	36	34.5 (*)	0.41	20-50	Flexible	*	HAMD 21	25.00	6	DSM-III	North America	Single center	Secondary /Tertiary care	Outpatients only	Yes	Unclear

PAR 02-002 - FDA)				placebo	No	36	35.7 (*)	0.39	0-0	Flexible	0	HAMD 21	24.60								
Dunbar 1993c (Smith 1992, PAR 02-003 - FDA)	1993	2	2	paroxetine	Yes	39	44.9 (*)	0.46	20-50	Flexible	*	HAMD 21	28.60	6	DSM-III	North America	Single center	Secondary /Tertiary care	Outpatients only	Yes	Unclear
				placebo	No	38	44.6 (*)	0.55	0-0	Flexible	0	HAMD 21	28.90								
Dunbar 1993d (Kiev 1992, PAR 02-004 - FDA)	1993	2	2	paroxetine	Yes	40	34.9 (*)	0.41	20-50	Flexible	*	HAMD 21	28.80	6	DSM-III	North America	Single center	Secondary /Tertiary care	Outpatients only	Yes	Unclear
				placebo	No	41	40.3 (*)	0.50	0-0	Flexible	0	HAMD 21	27.50								
Dunlop 2011 (NCT00824291)	2011	2	2	desvenlafaxine	Yes	291	43.2 (11.7)	0.66	50-50	Fixed	50	HAMD 17	22	12	DSM-IV	North America	Multi-center	Secondary /Tertiary care	Outpatients only	Unclear	Yes
				placebo	No	146	41.6 (12.6)	0.66	0-0	Fixed	0	HAMD 17	21.8								
Dunner 1992 (PAR 29060.09)	1992	4	5	paroxetine	Yes	106	41.6 (*)	*	20-20	Fixed	20	HAMD 21	25.19	6	DSM-III	North America	Multi-center	Other/Unclear	Outpatients only	Unclear	Unclear
				paroxetine	Yes	104	41.6 (*)	*	30-30	Fixed	30	HAMD 21	25.18								
				paroxetine	Yes	106	41.4 (*)	*	40-40	Fixed	40	HAMD 21	25.03								
				placebo	No	53	40.9 (*)	*	0-0	Fixed	0	HAMD 21	24.50								
E-1569	unpublished	2	2	mirtazapine	Yes	73	46.5 (12.5)	0.68	30-45	Flexible	*	HAMD 17	24.60	6	DSM-IV	Europe	Multi-center	Other/Unclear	Outpatients only	Yes	Unclear
				paroxetine	No	75	43 (12.5)	0.69	20-40	Flexible	*	HAMD 17	25.70								
Edwards 1989 (MD/PAR/009 PAR-276)	1989	2	2	paroxetine	Yes	21	45.1 (*)	0.55	30-30	Fixed	30	HAMD 17	26.80	6	DSM-III	Europe	Single center	Secondary /Tertiary care	Outpatients only	Unclear	Yes
				placebo	No	21	43.3 (*)	0.57	0-0	Fixed	0	HAMD 17	25.50								
Ekselius 1997	1997	2	2	citalopram	No	200	48.1 (12)	*	20-60	Flexible	34	MADRS	28.30	12	DSM-III-R	Europe	Multi-center	Primary care	Outpatients only	Yes	No
				sertraline	Yes	200	47.3 (13.3)	*	50-150	Flexible	82	MADRS	28.30								
Faber 1995 (SER 103 FDA)	1995	4	4	sertraline	Yes	95	37 (*)	0.51	50-50	Fixed	50	HAMD17	24.80	6	DSM-III	North America	Multi-center	Other/Unclear	Outpatients only	Yes	Yes
				sertraline	Yes	92	37 (*)	0.57	100-100	Fixed	100	HAMD 17	24.90								
				sertraline	Yes	91	38 (*)	0.63	200-200	Fixed	200	HAMD 17	25.70								
				placebo	No	91	37 (*)	0.44	0-0	Fixed	0	HAMD 17	25.30								
Fabre 1979	1979	2	3	trazodone	Yes	13	*	*	200-300	Flexible	287	HAMD unspecified	*	4	Other operationalized	North America	Unclear	Secondary /Tertiary care	Unclear	Yes	Yes
				placebo	No	11	*	*	0-0	Flexible	0	HAMD unspecified	*								

Fabre 1996	1996	2	3	fluvoxamine	Yes	50	45 (*)	*	50-150	Flexible	119	HAMD 21	27.70	6	DSM-III-R	North America	Single center	Secondary /Tertiary care	Outpatients only	Yes	Yes
				placebo	No	50	41 (*)	*	0-0	Flexible	0	HAMD 21	26.00								
Falk 1989	1989	2	2	trazodone	No	13	67.5 (4)	*	50-400	Flexible	350	HAMD 21	26.17	6	DSM-III	North America	Single center	Secondary /Tertiary care	Outpatients only	Yes	Unclear
				fluoxetine	Yes	14	69.1 (6.2)	*	20-60	Flexible	48	HAMD 21	23.77								
Fang 1997 (Yu 1997)	1997	2	2	fluoxetine	Unclear	11	36 (8)	0.55	20-20	Fixed	20	HAMD17	33	6	Other operati onalize d	Asia	Single center	Secondary /Tertiary care	Inpatients only	No	Unclear
				amitriptyline	Unclear	12	34 (13)	0.58	25-75	Other	*	HAMD 17	34								
Fava 1998	1998	3	3	paroxetine	Yes	55	41.3 (12.6)	*	20-50	Flexible	*	HAMD 21	23.10	12	DSM-III-R	North America	Multi-center	Unclear	Outpatients only	Yes	Unclear
				fluoxetine	No	54	41.3 (12.6)	*	20-80	Flexible	*	HAMD 21	23.90								
				placebo	No	19	41.3 (12.6)	*	0-0	Flexible	0	HAMD 21	23.70								
Fava 2002	2002	3	3	sertraline	No	96	44 (14.7)	*	50-200	Flexible	108	HAMD 17	21.00	10	DSM-IV	North America	Multi-center	Secondary /Tertiary care	Outpatients only	Yes	Yes
				fluoxetine	Yes	92	42.1 (13.5)	*	20-60	Flexible	42	HAMD 17	20.50								
				paroxetine	No	96	42.5 (14.7)	*	20-60	Flexible	37	HAMD 17	20.60								
Fava 2005	2005	2	3	placebo	No	43	37.8 (12)	*	0-0	Fixed	0	HAMD 17	19.90	12	DSM-IV	North America	Multi-center	Secondary /Tertiary care	Outpatients only	Yes	Unclear
				fluoxetine	No	47	36.7 (9.6)	*	20-20	Fixed	20	HAMD 17	19.60								
Fawcett 1989	1989	2	2	amitriptyline	No	20	44.5 (10.3)	*	50-200	Flexible	*	HAMD 21	23.50	6	DSM-III	North America	Multi-center	Secondary /Tertiary care	Outpatients only	Yes	Yes
				fluoxetine	Yes	20	39.9 (10.6)	*	20-60	Flexible	*	HAMD 21	23.60								
Feiger 1996	1996	2	2	nefazodone	Yes	78	44.5 (11.5)	0.55	100-600	Flexible	456	HAMD 17	23.30	6	DSM-III-R	North America	Multi-center	Other/Unc lear	Outpatients only	Unclear	Yes
				sertraline	No	82	43.1 (10)	0.48	50-200	Flexible	148	HAMD 17	23.30								
Feighner 1979	1979	2	4	amitriptyline	No	93	40.9 (*)	*	100-150	Flexible	115	HAMD 24	36	4	Feighne r	North America	Multi-center	Secondary /Tertiary care	Outpatients only	Yes	Unclear
				placebo	No	50	40.9 (*)	*	0-0	Flexible	0	HAMD 24	34.7								
Feighner 1980	1980	2	3	trazodone	Yes	19	*	0.65	200-600	Flexible	313	HAMD unspecified	35.40	4	Feighne r	North America	Unclear	Secondary /Tertiary care	Inpatients only	Yes	Unclear
				placebo	No	10	*	0.60	0-0	Flexible	0	HAMD unspecified	36.00								

Feighner 1984	1984	2	2	bupropion	Yes	45	43.9 (*)	*	300-600	Flexible	392	HAMD 21	31.00	4	Feighner	North America	Unclear	Secondary /Tertiary care	Inpatients only	No	Yes
				placebo	No	22	49 (*)	*	0-0	Flexible	0	HAMD 21	28.00								
Feighner 1989a	1989	2	3	placebo	No	59	39.7 (*)	*	0-0	Flexible	0	HAMD 21	25.90	6	DSM-III	North America	Single center	Unclear	Outpatients only	Yes	Unclear
				fluoxetine	Unclear	62	45 (*)	*	*-80	Flexible	*	HAMD 21	25.60								
Feighner 1989b	1989	2	3	fluvoxamine	Unclear	31	41 (*)	*	150-300	Flexible	145	HAMD unspecified	25.00	6	DSM-III	North America	Unclear	Secondary /Tertiary care	Inpatients only	No	Unclear
				placebo	No	19	41 (*)	*	0-0	Flexible	0	HAMD unspecified	25.00								
Feighner 1991	1991	2	2	fluoxetine	No	62	42.9 (*)	*	20-80	Flexible	38	HAMD 17	26.10	6	DSM-III-R	North America	Multi-center	Unclear	Outpatients only	Yes	Unclear
				bupropion	Yes	61	40.9 (*)	*	225-450	Flexible	382	HAMD 17	25.30								
Feighner 1993a (Feighner 1989c PAR 03 001 - FDA)	1993	2	3	paroxetine	Yes	40	42.6 (*)	0.56	10-50	Flexible	26	HAMD 21	24.80	6	DSM-III	North America	Single center	Secondary /Tertiary care	Outpatients only	Yes	Unclear
				placebo	No	40	42.3 (*)	0.55	0-0	Flexible	0	HAMD 21	24.70								
Feighner 1993b (Cohn 1990 Cohn 1992 PAR 03 002 - FDA)	1993	2	3	paroxetine	Yes	40	42.3 (*)	0.69	10-50	Flexible	31	HAMD 21	25.60	6	DSM-III	North America	Single center	Secondary /Tertiary care	Outpatients only	Yes	Unclear
				placebo	No	40	41.3 (*)	0.47	0-0	Flexible	0	HAMD 21	26.70								
Feighner 1993c (PAR 03 003 - FDA)	1993	2	3	paroxetine	Yes	41	39.8 (*)	0.61	10-50	Flexible	*	HAMD 21	25.60	6	DSM-III	North America	Single center	Secondary /Tertiary care	Outpatients only	Yes	Unclear
				placebo	No	42	41.3 (*)	0.65	0-0	Flexible	0	HAMD 21	26.70								
Feighner 1993d (Shrivastava 1992 PAR 03 004 - FDA)	1993	2	3	paroxetine	Yes	40	38 (*)	0.33	10-50	Flexible	40	HAMD 21	27.60	6	DSM-III	North America	Single center	Unclear	Outpatients only	Yes	Unclear
				placebo	No	40	34.6 (*)	0.39	0-0	Flexible	0	HAMD 21	26.70								
Feighner 1993e (Peselow 1989 PAR 03 005 - FDA)	1993	2	3	paroxetine	Yes	40	43.5 (*)	0.41	10-50	Flexible	38	HAMD 21	25.60	6	DSM-III	North America	Single center	Unclear	Outpatients only	Yes	Unclear
				placebo	No	42	44.7 (*)	0.24	0-0	Flexible	0	HAMD 21	26.70								
Feighner 1993f (Fabre 1992 PAR 03 006 - FDA)	1993	2	3	paroxetine	Yes	40	35.6 (*)	0.55	10-50	Flexible	35	HAMD 21	29.70	6	DSM-III	North America	Single center	Unclear	Outpatients only	Yes	Unclear
				placebo	No	40	35.9 (*)	0.64	0-0	Flexible	0	HAMD 21	28.80								
Feighner 1998	1998	2	2	nefazodone	Yes	61	37.2 (11.7)	0.61	400-600	Flexible	503	HAMD 17	27.50	6	DSM-III-R	North America	Multi-center	Secondary /Tertiary care	Inpatients only	Unclear	Yes
				placebo	No	59	39.5 (12.3)	0.63	0-0	Flexible	0	HAMD 17	27.90								
Feighner 1999 (Study 91206 FDA)	1999	4	5	citalopram	Yes	130	39 (*)	0.66	20-20	Fixed	20	HAMD 21	24.24	6	DSM-III-R	North America	Multi-center	Unclear	Outpatients only	Yes	Yes
				citalopram	Yes	131	39 (*)	0.60	40-40	Fixed	40	HAMD 21	24.44								
				citalopram	Yes	129	38 (*)	0.53	60-60	Fixed	60	HAMD 21	24.52								

				placebo	No	129	38 (*)	0.55	0-0	Fixed	0	HAMD 21	24.62							
Fontaine 1994 (FDA 03A0A-003)	1994	3	4	nefazodone	Yes	46	39.9 (10.9)	0.61	50-250	Flexible	242	HAMD 17	25.20	6	RDC	North America	Single center	Secondary /Tertiary care	Outpatients only	Yes	Yes
				nefazodone	Yes	44	43.3 (10.6)	0.73	100-500	Flexible	460	HAMD 17	25.60							
				placebo	No	45	42.2 (10.7)	0.67	0-0	Flexible	0	HAMD 17	25.90							
Frank 2004	2004	2	2	citalopram	Yes	8	39.12 (6.7)	0.25	20-20	Fixed	20	*	*	4	DSM-IV	North America	Single center	Secondary /Tertiary care	Outpatients only	No	Unclear
				placebo	No	8	41.5 (7.1)	0.75	0-0	Fixed	0	*	*							
Fudge 1990	1990	2	2	fluoxetine	Unclear	21	*	*	20-60	Flexible	*	HAMD unspecified	23.20	6	DSM-III	North America	Single center	Secondary /Tertiary care	Outpatients only	Yes	Unclear
				trazodone	Unclear	17	*	*	50-400	Flexible	*	HAMD unspecified	23.60							
Gagiano 1993	1993	2	2	paroxetine	Yes	45	37.8 (12.5)	*	20-40	Flexible	*	HAMD 21	25.00	6	DSM-III	Africa	Single center	Unclear	Outpatients only	Yes	Unclear
				fluoxetine	No	45	39.6 (11.7)	*	20-60	Flexible	*	HAMD 21	24.50							
Gastpar 2006	2006	2	3	citalopram	No	127	49.3 (10.7)	0.65	20-20	Fixed	20	HAMD unspecified	21.80	6	DSM-IV	Europe	Multi-center	Primary care	Outpatients only	Unclear	Unclear
				placebo	No	130	49.4 (12.7)	0.73	0-0	Fixed	0	HAMD unspecified	22.00							
Gelenberg 1990	1990	2	3	amitriptyline	No	19	*	*	50-350	Flexible	114	HAMD 21	24.84	6	DSM-III	North America	Single center	Secondary /Tertiary care	Outpatients only	Yes	Yes
				placebo	No	22	*	*	0-0	Flexible	0	HAMD 21	23.6							
Gentil 2000	2000	2	2	venlafaxine	Yes	57	37.9 (10.1)	*	75-150	Flexible	103	HAMD 21	23.90	8	DSM-IV	South America	Multi-center	Unclear	Outpatients only	Yes	Yes
				amitriptyline	No	59	39.1 (8.7)	*	75-150	Flexible	103	HAMD 21	24.50							
Georgotas 1982	1981	2	3	amitriptyline	No	15	36.1 (2.84)	*	150-300	Flexible	206	HAMD 21	28.5	4	RDC	North America	Unclear	Unclear	Other/Unclea r	Unclear	Unclear
				placebo	No	18	39.5 (3.11)	*	0-0	Flexible	0	HAMD 21	28.6							
Geretsegger 1995 (MY-060/BRL-029060/1)	1995	2	2	paroxetine	Yes	44	71 (*)	*	20-30	Flexible	*	HAMD 21	26.80	6	DSM-III	Europe	Multi-center	Unclear	Inpatients only	No	Unclear
				amitriptyline	No	47	71.3 (*)	*	100-150	Flexible	*	HAMD 21	28.30							
Gerner 1980	1980	2	3	trazodone	Yes	19	68.4 (*)	0.62	100-400	Flexible	305	HAMD unspecified	27.38	4	RDC	North America	Unclear	Unclear	Outpatients only	Unclear	Unclear
				placebo	No	20	68.4 (*)	0.62	0-0	Flexible	0	HAMD unspecified	25.00							
Gillin 1997	1997	2	2	nefazodone	Yes	24	35.3 (8.8)	0.67	400-500	Flexible	435	HAMD 17	22.90	8	DSM-III-R	North America	Multi-center	Unclear	Outpatients only	Unclear	Unclear
				fluoxetine	No	20	36.7 (8.5)	0.70	20-40	Flexible	34	HAMD 17	23.20							
Ginestet 1989	1989	2	2	fluoxetine	Unclear	28	46.5 (10.8)	0.71	20-80	Flexible	58	HAMD 21	33.10	8	DSM-III	Europe	Multi-center	Secondary /Tertiary care	Inpatients only	Unclear	Yes

				clomipramine	No	26	51.6 (11)	0.73	50-200	Flexible	148	HAMD 21	33.70								
Golden 2002a (29060/448)	2002	3	3	paroxetine	Yes	106	38.9 (10.6)	*	25-62.5	Flexible	*	HAMD 17	23.00	12	DSM-IV	North America	Multi-center	Unclear	Outpatients only	Yes	Unclear
				paroxetine	Yes	106	39.4 (10.7)	*	20-50	Flexible	*	HAMD 17	23.30								
				placebo	No	103	38.7 (9.91)	*	0-0	Flexible	0	HAMD 17	23.40								
Golden 2002b (29060/449)	2002	3	3	paroxetine	Yes	108	42.38 (10.8)	*	25-62.5	Flexible	*	HAMD 17	23.80	12	DSM-IV	North America	Multi-center	Unclear	Outpatients only	Yes	Unclear
				paroxetine	Yes	112	40.55 (12.1)	*	20-50	Flexible	*	HAMD 17	23.70								
				placebo	No	110	40.71 (11.6)	*	0-0	Flexible	0	HAMD 17	23.50								
Goldstein 2002 (HMAQ - Study Group A)	2002	3	3	duloxetine	Yes	70	42.33 (10.8)	*	40-120	Flexible	*	HAMD 17	18.47	10	DSM-IV	North America	Multi-center	Unclear	Outpatients only	Yes	Unclear
				placebo	No	70	41.35 (13.2)	*	0-0	Flexible	0	HAMD 17	19.44								
				fluoxetine	No	33	39.69 (10.5)	*	20-20	Flexible	*	HAMD 17	17.88								
Goldstein 2004a (HMAT - Study Group A, ID#4091)	2004	4	4	duloxetine	Yes	91	43.36 (15.3)	*	40-40	Fixed	40	HAMD 17	17.47	8	DSM-IV	North America	Multi-center	Unclear	Outpatients only	Yes	Unclear
				duloxetine	Yes	84	43.68 (14.1)	*	80-80	Fixed	80	HAMD 17	17.44								
				placebo	No	90	43.18 (14.5)	*	0-0	Fixed	0	HAMD 17	17.79								
				paroxetine	Yes	89	44.43 (14.7)	*	20-20	Fixed	20	HAMD 17	17.97								
Goldstein 2004b (HMAT - Study Group B, ID#4091)	2004	4	4	duloxetine	Yes	86	40.69 (10.0)	*	40-40	Fixed	40	HAMD 17	18.63	8	DSM-IV	North America	Multi-center	Unclear	Outpatients only	Yes	Unclear
				duloxetine	Yes	91	40.89 (11.9)	*	80-80	Fixed	80	HAMD 17	18.06								
				placebo	No	89	40.14 (12.9)	*	0-0	Fixed	0	HAMD 17	17.19								
				paroxetine	No	87	40.25 (11.0)	*	20-20	Fixed	20	HAMD 17	17.65								
Gommoll 2014 (LVM-MD-02, NCT00969150)	2014	2	2	placebo	No	182	43.7 (13.3)	0.64	0-0	Flexible	0	HAMD 17	24.40	8	DSM-IV	North America	Multi-center	Unclear	Outpatients only	Yes	Unclear
				levomilnacipran	Yes	175	42.8 (12.9)	0.57	40-120	Flexible	93	HAMD 17	24.90								
Goodarzi 2015(IRCT2012101811155N1)	2015	2	2	mirtazapine	Unclear	24	38 (5.47)	0.79	30-30	Fixed	30	HAMD unspecified	30.58	8	DSM-IV	Asia	Multi-center	Secondary /Tertiary care	Both	Unclear	Unclear
				citalopram	Unclear	28	40.35 (10.2)	0.89	40-40	Fixed	40	HAMD unspecified	28.85								
Gorman 2002 (SCT-MD-02)	2002	3	3	escitalopram	Yes	129	41.4 (11.9)	*	10-20	Flexible	*	HAMD unspecified	24.80	8	DSM-IV	North America	Multi-center	Unclear	Outpatients only	Yes	Unclear
				citalopram	Yes	128	42.1 (12.7)	*	20-40	Flexible	*	HAMD unspecified	25.00								
				placebo	No	129	42.2 (12.5)	*	0-0	Flexible	0	HAMD unspecified	25.00								
Griebel 2012 (Study DF15878) (NCT00358631)	2012	2	4	escitalopram	No	84	40.8 (10.7)	0.62	10-10	Fixed	10	HAMD 17	23.40	8	DSM-IV	North America	Multi-center	Unclear	Outpatients only	Yes	Unclear
				placebo	No	76	41.2 (12.4)	0.63	0-0	Fixed	0	HAMD 17	23.00								

Griebel 2012b (Study DF15879) (NCT00361491)	2012	2	4	paroxetine	No	80	40 (13.2)	0.70	20-20	Fixed	20	HAMD 17	24.90	8	DSM-IV	Cross-Continental	Multi-center	Unclear	Outpatients only	Yes	Unclear
				placebo	No	79	40.1 (10.6)	0.73	0-0	Fixed	0	HAMD 17	24.80								
Grunebaum 2011 (NCT00429169)	2011	2	2	bupropion	Yes	40	37.9 (11.9)	*	300-450	Flexible	275	HAMD 17	17.60	24	DSM-IV	North America	Single center	Secondary /Tertiary care	Both	No	Yes
				paroxetine	Yes	38	35.2 (12.8)	*	37.5-50	Flexible	34	HAMD 17	16.90								
GSK14	unpublished	2	2	bupropion	Yes	77	45.9 (*)	*	300-600	Flexible	*	HAMD 21	31.20	4	DSM-III	North America	Multi-center	Unclear	Inpatients only	No	Unclear
				placebo	No	40	48.2 (*)	*	0-0	Flexible	0	HAMD 21	30.30								
Guelfi 1995 (VEN 600A-206 FDA)	1995	2	2	venlafaxine	Yes	46	56 (14)	*	150-375	Flexible	350	HAMD 21	28.20	4	DSM-III-R	Europe	Multi-center	Secondary /Tertiary care	Inpatients only	Yes	Yes
				placebo	No	47	56 (13)	*	0-0	Flexible	0	HAMD 21	28.60								
Guelfi 1998	1998	3	3	milnacipran	Yes	100	45.6 (12.4)	*	100-100	Fixed	100	HAMD 17	27.90	12	DSM-III-R	Europe	Multi-center	Secondary /Tertiary care	Inpatients only	Yes	Yes
				milnacipran	Yes	100	45.2 (12.5)	*	200-200	Fixed	200	HAMD 17	27.70								
				fluoxetine	No	100	45.8 (12.8)	*	20-20	Fixed	20	HAMD 17	27.40								
Guillibert 1989 (PAR 290 MDF 29060 1727 M)	1989	2	2	clomipramine	No	39	68.1 (6.1)	*	75-75	Fixed	75	HAMD 21	27.50	6	DSM-III	Europe	Multi-center	Unclear	Unclear	Yes	Yes
				paroxetine	Yes	40	69.3 (5.3)	*	30-30	Fixed	30	HAMD 21	27.90								
Hackett 1998	1998	2	2	fluvoxamine	No	34	*	*	200-200	Fixed	200	MADRS	31.60	6	DSM-III-R	Europe	Multi-center	Unclear	Outpatients only	No	Unclear
				venlafaxine	Yes	77	*	*	150-150	Fixed	150	MADRS	32.20								
Hale 2010 (CL3-20098-045)	2008	2	2	agomelatine	Yes	252	41.8 (11.2)	0.77	25-50	Flexible	*	HAMD 17	28.50	24	DSM-IV	Cross-Continental	Multi-center	Primary care	Outpatients only	No	No
				fluoxetine	No	263	42.7 (11.9)	0.78	20-40	Flexible	*	HAMD 17	28.70								
Halikas 1995 (MIR 003-023 - FDA)	1995	3	3	mirtazapine	Yes	50	63 (*)	0.43	5-35	Flexible	29	HAMD 21	24.60	6	DSM-III	North America	Unclear	Unclear	Outpatients only	Yes	Yes
				trazodone	No	50	61 (*)	0.60	40-280	Flexible	220	HAMD 21	24.60								
				placebo	No	50	62 (*)	0.59	0-0	Flexible	0	HAMD 21	23.50								
Hao 2014	2014	2	2	duloxetine	Yes	140	38.4 (13)	0.56	60-60	Fixed	60	HAMD 17	23.70	6	DSM-IV	Asia	Multi-center	Both	Both	No	Yes
				paroxetine	No	141	38.5 (1.6)	0.62	20-20	Fixed	20	HAMD 17	23.70								
Harris 1991	1991	2	2	fluvoxamine	Unclear	35	38.6 (12.1)	0.63	50-150	Flexible	115	HAMD 17	22.70	6	DSM-III	Europe	Multi-center	Secondary /Tertiary care	Outpatients only	Yes	Yes
				amitriptyline	Unclear	34	48.1 (12.3)	0.82	50-150	Flexible	99	HAMD 17	22.80								
Heiligenstein 1994	1994	2	2	fluoxetine	Yes	46	41.6 (10.9)	*	20-20	Fixed	20	HAMD 17	21.10	8	DSM-III-R	North America	Multi-center	Secondary /Tertiary care	Outpatients only	Yes	Yes

				placebo	No	43	39.2 (9.8)	*	0-0	Fixed	0	HAMD 17	21.60								
Heller 2009 (NCT00909155)	2009	2	2	venlafaxine	Yes	15	30.74 (11.5)	0.53	75-300	Flexible	118	HAMD 21	20.07	24	DSM-IV	North America	Unclear	/Unclear	Other/Unclear	Unclear	Unclear
				fluoxetine	No	14	33.14 (10.7)	0.57	20-80	Flexible	37	HAMD 21	21.36								
Henigsberg 2012 (305, NCT00735709)	2012	3	4	placebo	No	140	46.4 (12.3)	0.61	0-0	Fixed	0	HAMD 24	32.70	8	DSM-IV	Cross-Continental	Multi-center	Unclear	Unclear	Unclear	Unclear
				vortioxetine	Yes	140	47.3 (11.9)	0.62	5-5	Fixed	5	HAMD 24	32.10								
				vortioxetine	Yes	140	46.4 (12.3)	0.61	10-10	Fixed	10	HAMD 24	33.10								
Heun 2013 (ISRCTI\l5750736O)	2013	2	2	agomelatine	Yes	151	71.9 (5.1)	0.70	25-50	Flexible	*	HAMD 17	26.80	8	DSM-IV	Cross-Continental	Multi-center	Both	Outpatients only	Unclear	Yes
				placebo	No	71	71.7 (4.8)	0.65	0-0	Flexible	0	HAMD 17	26.70								
Hewett 2009 (WXL101497)	2009	3	3	bupropion	Yes	188	41.8 (11.7)	*	150-300	Flexible	*	MADRS	30.40	12	DSM-IV	Cross-Continental	Multi-center	Secondary /Tertiary care	Outpatients only	Yes	Unclear
				venlafaxine	No	189	42.7 (11.48)	*	75-150	Flexible	*	MADRS	30.00								
				placebo	No	199	41.8 (11.6)	*	0-0	Flexible	0	MADRS	30.40								
Hewett 2010a (AK130940) (NCT00093288)	2010	2	2	bupropion	Yes	212	70.9 (5.55)	*	150-300	Flexible	*	MADRS	29.50	10	DSM-IV	Cross-Continental	Multi-center	Unclear	Outpatients only	Unclear	Unclear
				placebo	No	208	71.3 (5.88)	*	0-0	Flexible	0	MADRS	29.80								
Hewett 2010b (AK130939)	2010	2	3	venlafaxine	No	198	44.1 (11.5)	*	75-150	Flexible	85	MADRS	30.10	8	DSM-IV	Cross-Continental	Multi-center	Unclear	Outpatients only	No	Unclear
				placebo	No	189	44.5 (10.8)	*	0-0	Flexible	0	MADRS	30.60								
Hicks 1988	1988	2	3	amitriptyline	No	16	42.2 (*)	*	25-300	Flexible	142	HAMD 17	30.8	6	DSM-III	North America	Single center	Secondary /Tertiary care	Inpatients only	Yes	Unclear
				placebo	No	15	40.8 (*)	*	0-0	Flexible	0	HAMD 17	29.4								
Hicks 2002	2002	2	2	nefazodone	Yes	20	42.8 (11.9)	0.40	400-600	Flexible	495	HAMD unspecified	21.60	24	DSM-IV	Europe	Single center	Secondary /Tertiary care	Outpatients only	Unclear	No
				paroxetine	No	20	43 (10.1)	0.45	20-40	Flexible	30	HAMD unspecified	22.30								
Higuchi 2014 (NCT01441440)	2016	3	3	venlafaxine	Yes	174	38.4 (11.9)	0.52	75-75	Fixed	75	HAMD 17	*	8	DSM-IV	Asia	Multi-center	Unclear	Outpatients only	Unclear	Unclear
				venlafaxine	Yes	180	38.3 (10.2)	0.48	75-225	Flexible	*	HAMD 17	*								
				placebo	No	184	38.6 (11.1)	0.51	0-0	Flexible	0	HAMD 17	*								
Higuchi 2009	2009	4	4	duloxetine	Yes	75	37.9 (9.3)	*	40-40	Flexible	40	HAMD 17	20.60	6	DSM-IV	Asia	Multi-center	Both	Both	Yes	Yes
				duloxetine	Yes	75	39 (10.1)	*	60-60	Fixed	60	HAMD 17	20.40								
				paroxetine	No	148	37.9 (9.6)	*	20-40	Flexible	*	HAMD 17	20.40								

				placebo	No	146	38.7 (10.5)	*	0-0	Fixed	0	HAMD 17	20.40								
Higuchi 2011 (PCR112810, NCT00866294)	2011	3	3	paroxetine	Yes	83	35.5 (10.4)	*	20-40	Flexible	*	HAMD 17	22.70	8	DSM-IV	Asia	Multi-center	Unclear	Unclear	Yes	Unclear
				paroxetine	Yes	161	36.4 (11.5)	*	25-50	Flexible	*	HAMD 17	22.70								
				placebo	No	172	36.8 (10.1)	*	0-0	Flexible	0	HAMD 17	22.60								
Hirayasu 2011a	2011	3	3	escitalopram	Yes	105	35.9 (11.2)	*	20-20	Fixed	20	HAMD 17	22.20	8	DSM-IV	Asia	Multi-center	Both	Outpatients only	No	Yes
				escitalopram	Yes	100	33.1 (9)	*	10-10	Fixed	10	HAMD 17	22.30								
				placebo	No	105	34.7 (8.6)	*	0-0	Fixed	0	HAMD 17	22.50								
Hirayasu 2011b	2011	4	4	placebo	No	124	36.4 (10.8)	*	0-0	Fixed	0	MADRS	29.00	8	DSM-IV	Asia	Multi-center	Both	Outpatients only	Yes	Yes
				escitalopram	Yes	121	36.3 (9.7)	*	10-10	Fixed	10	MADRS	29.40								
				escitalopram	Yes	119	35.7 (9.8)	*	20-20	Fixed	20	MADRS	29.80								
				paroxetine	No	121	36.3 (8.4)	*	20-40	Flexible	*	MADRS	29.80								
Hong 2003 (22521)	2003	2	2	mirtazapine	Yes	66	47.2 (14.7)	*	30-45	Flexible	34	HAMD 17	23.10	6	DSM-IV	Asia	Multi-center	Secondary /Tertiary care	Outpatients only	Yes	Unclear
				fluoxetine	No	66	47.1 (15.5)	*	20-40	Flexible	31	HAMD 17	24.30								
Hormazabal 1985	1985	2	3	amitriptyline	No	20	43.9 (15.3)	*	50-150	Flexible	86	HAMD 21	36.7	4	DSM-III	South America	Unclear	Unclear	Both	Unclear	Unclear
				placebo	No	20	42.3 (14.5)	*	0-0	Flexible	0	HAMD 21	35.8								
Hosseini 2015 (IRCT20120304 8513N1.)	2015	2	2	venlafaxine	No	28	37.12 (10.9)	0.75	37.5-150	Other/U nclear	*	HAMD unspecified	19.88	8	DSM-IV	Asia	Single center	Secondary /Tertiary care	Outpatients only	Unclear	Unclear
				citalopram	No	25	35.6 (8.72)	0.91	10-40	Other/U nclear	*	HAMD unspecified	17.63								
Hoyberg 1996 (Organon 88013, FDA88013)	1996	2	2	mirtazapine	Yes	56	70.3 (5.4)	*	15-45	Flexible	37	HAMD 17	26.70	6	DSM-III	Europe	Multi-center	Secondary /Tertiary care	Outpatients only	No	Yes
				amitriptyline	No	59	71.3 (5.6)	*	30-90	Flexible	74	HAMD 17	25.70								
Hsu 2011	2011	2	2	citalopram	No	25	43.4 (11.4)	*	20-*	Flexible	23	MADRS	36.60	6	DSM-IV	Asia	Single center	Secondary /Tertiary care	Other/Unclea r	No	Yes
				sertraline	No	26	38 (13.8)	*	50-*	Flexible	57	MADRS	38.20								
Hu 2009	2009	2	2	escitalopram	Unclear	25	30.92 (8.04)	0.52	10-20	Flexible	*	HAMD 17	21.30	6	Other operati onalize d	Asia	Single center	Secondary /Tertiary care	Outpatients only	Yes	Yes
				citalopram	Unclear	23	30.61 (8.61)	0.57	20-40	Flexible	*	HAMD 17	20.78								

Hunter 2010 (Study 1)	2010	2	2	fluoxetine	Yes	14	42.4 (12.6)	0.68	20-20	Fixed	20	HAMD 17	22.10	8	DSM-IV	North America	Multi-center	Unclear	Outpatients only	Yes	No
				placebo	No	14	42.4 (12.6)	0.68	0-0	Fixed	0	HAMD 17	22.10								
Hunter 2010 (Study 2)	2010	2	2	venlafaxine	Yes	17	44.7 (14)	0.64	150-150	Fixed	150	HAMD 17	22.30	8	DSM-IV	North America	Multi-center	Unclear	Outpatients only	Yes	No
				placebo	No	16	44.7 (14)	0.64	0-0	Fixed	0	HAMD 17	22.30								
Hunter 2010 (Study 3)	2010	2	2	venlafaxine	Yes	18	38.1 (12.5)	0.55	150-150	Fixed	150	HAMD 17	21.50	8	DSM-IV	North America	Multi-center	Unclear	Outpatients only	Yes	No
				placebo	No	15	38.1 (12.5)	0.55	0-0	Fixed	0	HAMD 17	21.50								
Hutchinson 1992	1992	2	2	amitriptyline	No	32	71.5 (9.5)	*	100-100	Fixed	100	HAMD 21	20.28	6	DSM-III	Europe	Multi-center	Primary care	Outpatients only	Yes	Yes
				paroxetine	Yes	58	72 (5.6)	*	30-30	Fixed	30	HAMD 21	21.56								
Itil 1983	1983	2	3	fluvoxamine	Yes	22	41.3 (*)	0.43	50-300	Flexible	101	HAMD 17	20.30	4	RDC	North America	Unclear	Unclear	Outpatients only	Yes	Yes
				placebo	No	22	41.3 (*)	0.43	0-0	Flexible	0	HAMD 17	19.70								
Iwata 2013 (NCT00798707)	2013	2	3	desvenlafaxine	Yes	237	39 (12)	0.56	50-50	Fixed	50	HAMD 17	23	8	DSM-IV	Cross-Continental	Multi-center	Unclear	Outpatients only	Yes	Unclear
				placebo	No	235	40 (12)	0.55	0-0	Fixed	0	HAMD 17	23								
Jacobsen 2015 (316, NCT01163266)	2015	3	3	placebo	No	157	42.3 (11.6)	0.70	0-0	Fixed	0	MADRS	32.00	8	DSM-IV	North America	Multi-center	Unclear	Unclear	Unclear	Unclear
				vortioxetine	Yes	155	43.1 (12.0)	0.76	10-10	Fixed	10	MADRS	32.30								
				vortioxetine	Yes	150	43.1 (13.1)	0.71	20-20	Fixed	20	MADRS	32.40								
Jain 2013 (303, NCT00672958)	2013	2	2	placebo	No	300	42.4 (12.7)	0.55	0-0	Fixed	0	HAMD 24	32.20	6	DSM-IV	North America	Multi-center	Both	Outpatients only	Unclear	No
				vortioxetine	Yes	300	42.5 (13.0)	0.62	5-5	Fixed	5	HAMD 24	32.70								
Jefferson 2000 (29060/785)	2000	4	5	placebo	No	105	40.2 (11.5)	*	0-0	Fixed	0	MADRS	31.70	6	DSM-IV	North America	Multi-center	Unclear	Outpatients only	No	Unclear
				paroxetine	Yes	103	41.5 (12.1)	*	25-25	Fixed	25	MADRS	31.40								
				citalopram	Yes	107	38.4 (11.3)	*	20-20	Fixed	20	MADRS	31.40								
				citalopram	Yes	100	39.6 (11.1)	*	40-40	Fixed	40	MADRS	31.00								
Jefferson 2006	2006	2	2	placebo	No	139	39.8 (*)	*	0-0	Flexible	0	IDS-IVR-30	46.00	8	DSM-IV	North America	Multi-center	Secondary /Tertiary care	Outpatients only	Unclear	Yes
				bupropion	Yes	135	40 (*)	*	300-450	Flexible	384	IDS-IVR-30	45.90								
Jiang 2009	2009	2	2	escitalopram	Unclear	32	38.62 (11.54)	0.56	10-20	Flexible	*	HAMD 17	23.82	6	Other operationalized	Asia	Single center	Secondary /Tertiary care	Both	Unclear	Yes
				citalopram	Unclear	32	39.23 (11.9)	0.53	20-60	Flexible	*	HAMD 17	23.46								

Judd 1993	1993	2	2	fluoxetine	Yes	30	41.7 (9.8)	0.66	20-20	Fixed	20	HAMD 17	24.40	6	DSM-III-R	Australia	Multi-center	Unclear	Unclear	Yes	Yes
				amitriptyline	No	28	41.7 (9.8)	0.66	150-200	Flexible	171	HAMD 17	23.70								
Kamijima 2013 (japicCTI-090733)	2013	3	3	milnacipran	Yes	303	35.9 (10.4)	0.53	100-100	Fixed	100	HAMD 17	22.10	9	DSM-IV	Asia	Multi-center	Unclear	Both	Yes	Yes
				milnacipran	Yes	301	36.7 (10.5)	0.51	200-200	Fixed	200	HAMD 17	22.30								
				paroxetine	No	301	36.6 (11.1)	0.55	30-40	Flexible	*	HAMD 17	22.10								
Kane 1983	1983	2	4	bupropion	Unclear	13	63.9 (6.98)	*	100-450	Flexible	323	HAMD unspecified	24.10	4	Other operati onalize d	North America	Multi-center	Unclear	Unclear	Yes	Unclear
				placebo	No	7	63.9 (6.98)	*	0-0	Flexible	0	HAMD unspecified	25.60								
Kasper 2005a (Study 99024)	2005	3	3	escitalopram	Yes	174	75 (7)	*	10-10	Fixed	10	MADRS	28.20	8	DSM-IV	Europe	Multi-center	Both	Both	Yes	Unclear
				fluoxetine	No	164	75 (7)	*	20-20	Fixed	20	MADRS	28.50								
				placebo	No	180	75 (7)	*	0-0	Fixed	0	MADRS	28.60								
Kasper 2005b	2005	2	2	paroxetine	Unclear	53	44.3 (11.3)	0.68	20-40	Flexible	22	HAMD 17	20.90	6	DSM-IV	Europe	Multi-center	Unclear	Outpatients only	Yes	Yes
				trazodone	Unclear	55	43.5 (12.2)	0.58	150-450	Flexible	305	HAMD 17	21.00								
Kasper 2012 (NCT00807248)	2012	2	4	escitalopram	Yes	140	41.6 (12.6)	0.69	20-20	Fixed	20	MADRS	35.40	8	DSM-IV	Europe	Multi-center	Secondary /Tertiary care	Both	Unclear	No
				placebo	No	71	42.6 (11.6)	0.75	0-0	Fixed	0	MADRS	34.70								
Katona 2012 (12541A, NCT00811252)	2012	3	3	placebo	No	145	70.3 (4.4)	0.62	0-0	Fixed	0	HAMD 24	29.40	8	DSM-IV	Cross-Continental	Multi-center	Secondary /Tertiary care	Both	Unclear	Unclear
				vortioxetine	Yes	156	70.5 (4.8)	0.69	5-5	Fixed	5	HAMD 24	29.20								
				duloxetine	No	151	70.9 (5.5)	0.66	60-60	Fixed	60	HAMD 24	28.50								
Katz 1993a	1993	2	3	amitriptyline	No	95	42.6 (*)	*	75-150	Flexible	*	HAMD 17	24.3	4	DSM-III	Cross-Continental	Unclear	Unclear	Unclear	Yes	Yes
				placebo	No	94	44.7 (*)	*	0-0	Flexible	0	HAMD 17	24.5								
Katz 1993b	1993	2	3	amitriptyline	No	93	43.2 (*)	*	75-225	Flexible	*	HAMD 17	23.6	4	DSM-III-R	Cross-Continental	Unclear	Unclear	Unclear	Yes	Yes
				placebo	No	104	44 (*)	*	0-0	Flexible	0	HAMD 17	22.2								
Katz 2004	2004	2	3	paroxetine	Unclear	28	46 (*)	*	20-60	Flexible	*	HAMD 21	24.00	6	DSM-III-R	North America	Multi-center	Secondary /Tertiary care	Inpatients only	Yes	Unclear
				placebo	No	25	46 (*)	*	0-0	Flexible	0	HAMD 21	25.90								
Kavoussi 1997	1997	2	2	bupropion	Yes	122	39.2 (10.3)	0.48	100-300	Flexible	238	HAMD unspecified	*	16	DSM-IV	North America	Multi-center	Unclear	Outpatients only	No	Yes
				sertraline	No	126	40 (10.5)	0.48	50-200	Flexible	114	HAMD unspecified	*								

Keegan 1991	1991	2	2	fluoxetine	Yes	20	39.5 (13.6)	0.67	20-80	Flexible	53	HAMD 21	*	6	DSM-III	North America	Multi-center	Unclear	Unclear	Yes	Yes
				amitriptyline	No	22	47.8 (14.2)	0.67	100-250	Flexible	171	HAMD 21	*								
Kellams 1979	1979	2	3	trazodone	Unclear	9	*	*	200-600	Flexible	515	HAMD unspecified	23.50	4	Other operationalized	North America	Single center	Secondary /Tertiary care	Inpatients only	Yes	Yes
				placebo	No	9	*	*	0-0	Flexible	0	HAMD unspecified	26.90								
Keller 2006a (Study 059, NCT00035009)	2006	2	3	paroxetine	No	154	39 (*)	0.68	20-20	Fixed	20	HAMD 17	27.50	8	DSM-IV	North America	Multi-center	Unclear	Outpatients only	Unclear	Unclear
				placebo	No	155	41.4 (*)	0.61	0-0	Fixed	0	HAMD 17	27.70								
Keller 2006b (Study 061) (NCT00035295)	2006	2	4	paroxetine	No	143	42.2 (*)	0.75	20-20	Fixed	20	HAMD 17	27.60	8	DSM-IV	North America	Multi-center	Unclear	Outpatients only	Unclear	Unclear
				placebo	No	145	41.1 (*)	0.74	0-0	Fixed	0	HAMD 17	27.30								
Keller 2006c (Study 062) (NCT00048607)	2006	2	4	paroxetine	No	164	40.6 (*)	0.64	20-20	Fixed	20	HAMD 17	28.30	8	DSM-IV	Cross-Continental	Multi-center	Unclear	Outpatients only	Unclear	Unclear
				placebo	No	161	41.3 (*)	0.71	0-0	Fixed	0	HAMD 17	28.30								
Keller 2007 (NCT00046020)	2007	2	2	fluoxetine	Unclear	275	40 (11.6)	*	20-60	Flexible	50	HAMD 17	23.00	10	DSM-IV	North America	Multi-center	Secondary /Tertiary care	Outpatients only	Unclear	No
				venlafaxine	Yes	821	39.6 (11.2)	*	75-300	Flexible	208	HAMD 17	22.60								
Kennedy 2005 (SCT-MD-16)	unpublished	2	2	escitalopram	Yes	102	37.1 (11.4)	*	10-20	Flexible	*	HAMD unspecified	25.90	8	DSM-IV	North America	Multi-center	Unclear	Outpatients only	Unclear	Unclear
				fluoxetine	No	103	37 (11.8)	*	20-40	Flexible	*	HAMD unspecified	26.50								
Kennedy 2008 (CL3-20098-043)	2003	2	2	agomelatine	Yes	107	42.3 (12.7)	0.65	25-50	Flexible	*	HAMD 17	26.50	6	DSM-IV	Cross-Continental	Multi-center	Secondary /Tertiary care	Both	No	No
				placebo	No	105	42.7 (12)	0.55	0-0	Flexible	0	HAMD 17	26.70								
Kennedy 2014 (EudraCT 2009-011238-84, CL3-20098-069)	2014	3	4	agomelatine	Yes	138	46.5 (11.9)	0.65	25-25	Fixed	25	HAMD 17	26.70	6	DSM-IV	Cross-Continental	Multi-center	Unclear	Outpatients only	Unclear	No
				agomelatine	Yes	137	43.1 (12.6)	0.72	25-50	Flexible	*	HAMD 17	26.70								
				placebo	No	141	45 (13.1)	0.77	0-0	Flexible	0	HAMD 17	26.60								
Kerkhofs 1990	1990	2	2	fluoxetine	Yes	16	45.3 (12.7)	0.69	60-60	Fixed	60	HAMD unspecified	21.90	6	RDC	Europe	Unclear	Unclear	Other/Unclear	Yes	Yes
				amitriptyline	No	18	46.2 (11.4)	0.61	150-150	Fixed	150	HAMD unspecified	21.80								
Kerr 1984	1984	2	2	amitriptyline	No	38	62.5(median) (*)	0.62	75-225	Flexible	*	HAMD 17	19.00	6	Other operationalized	Europe	Multi-center	Secondary /Tertiary care	Both	Unclear	Unclear
				trazodone	Yes	36	59 (median) (*)	0.76	150-300	Flexible	*	HAMD 17	20.00								
Khan 1998	1998	4	4	venlafaxine	Yes	85	43.3 (*)	0.68	75-75	Fixed	75	HAMD 21	24.30	12	DSM-III-R	North America	Multi-center	Unclear	Outpatients only	yes	unclear

				venlafaxine	Yes	90	40 (*)	0.64	150-150	Fixed	150	HAMD 21	24.50								
				venlafaxine	Yes	83	43.6 (*)	0.60	200-200	Fixed	200	HAMD 21	24.80								
				placebo	No	95	40.2 (*)	0.61	0-0	Fixed	0	HAMD 21	25.10								
Khan 2007 (SCT-MD-23, NCT00108979)	2007	2	2	escitalopram	Yes	140	41.8 (12.7)	*	10-20	Flexible	*	HAMD 24	26.60	8	DSM-IV	North America	Multi-center	Unclear	Outpatients only	Unclear	Unclear
				duloxetine	No	138	43 (13.4)	*	60-60	Fixed	60	HAMD 24	26.90								
Khan 2011 (CLDA-07-DP-02, NCT00683592)	2011	2	2	vilazodone	Yes	240	41.1 (12.2)	*	40-40	Fixed	40	HAMD 17	25.00	8	DSM-IV	North America	Multi-center	Other/Unclear	Other/Unclear	Unclear	Unclear
				placebo	No	241	42.4 (12.5)	*	0-0	Fixed	0	HAMD 17	25.30								
Kiev 1997	1997	2	2	fluvoxamine	Yes	30	42.7 (*)	*	50-150	Flexible	102	HAMD 21	24.40	7	DSM-III-R	North America	Multi-center	Secondary /Tertiary care	Outpatients only	Yes	Yes
				paroxetine	No	30	39.9 (*)	*	20-50	Flexible	36	HAMD 21	24.40								
Kinoshita 2009	2009	4	4	mirtazapine	Yes	70	39.4 (10.9)	*	15-15	Fixed	15	HAMD 17	23.20	6	DSM-IV	Asia	Multi-center	Both	Both	No	No
				mirtazapine	Yes	70	37.7 (10.8)	*	30-30	Fixed	30	HAMD 17	22.50								
				mirtazapine	Yes	71	41.6 (12.5)	*	45-45	Fixed	45	HAMD 17	22.10								
				placebo	No	70	39.9 (12.8)	*	0-0	Fixed	0	HAMD 17	22.50								
Koshino 2013 (NCT01138007)	2013	2	3	bupropion	Yes	192	37.5 (10.9)	0.56	300-300	Fixed	300	MADRS	32.10	8	DSM-IV	Asia	Multi-center	Secondary /Tertiary care	Other/Unclear	Unclear	Unclear
				placebo	No	187	37.9 (11.1)	0.54	0-0	Fixed	0	MADRS	31.90								
Kramer 1998	1998	2	3	paroxetine	Unclear	72	*	*	20-20	Fixed	20	HAMD 21	*	6	DSM-IV	North America	Multi-center	Other/Unclear	Outpatients only	Unclear	Unclear
				placebo	No	70	*	*	0-0	Fixed	0	HAMD 24	*								
Kuhs 1989 (MDUK_29060_III_85_043)	1989	2	2	paroxetine	Yes	12	39.2 (11.8)	0.9	30-30	Fixed	30	HAMD 21	24.60	6	DSM-III	Europe	Single center	Other/Unclear	Other/Unclear	Yes	Unclear
				amitriptyline	No	12	39.1 (11.5)	1.0	150-150	Fixed	150	HAMD 21	25.00								
Kusalic 1993	1993	2	3	amitriptyline	No	13	41.3 (10.1)	*	*-*	Flexible	110	HAMD 17	*	6	DSM-III-R	North America	Unclear	Secondary /Tertiary care	Outpatients only	Yes	Unclear
				placebo	No	15	41.3 (10.1)	*	0-0	Flexible	0	HAMD 17	*								
Kyle 1998 (Study 92032 - FDA)	1998	2	2	citalopram	Yes	179	73.4 (*)	0.73	20-40	Flexible	24	MADRS	27.8	8	DSM-III-R	Europe	Multi-center	Primary care	Other/Unclear	Yes	Unclear
				amitriptyline	No	186	74.1 (*)	0.74	50-100	Flexible	57	MADRS	29.4								
Laakman 1988	1988	2	2	fluoxetine	Unclear	63	*	*	20-60	Flexible	*	HAMD unspecified	26.7	5	Other operationalized	Europe	Unclear	Other/Unclear	Outpatients only	Yes	Yes

				amitriptyline	Unclear	65	*	*	50-150	Flexible	*	HAMD unspecified	23.8								
Lalit 2004	2004	3	3	escitalopram	Yes	69	33 (8)	*	10-20	Flexible	*	HAMD 17	26.00	4	ICD-10	Asia	Multi-center	Other/Unclear	Outpatients only	No	Yes
				citalopram	Yes	74	33 (9)	*	20-40	Flexible	*	HAMD 17	25.00								
				sertraline	No	71	37 (11)	*	100-150	Flexible	*	HAMD 17	25.00								
Lam 1995	1995	2	2	fluoxetine	Yes	36	40.9 (8.6)	*	20-20	Fixed	20	HAMD 21	18.70	5	DSM-III-R	North America	Multi-center	Secondary /Tertiary care	Outpatients only	Yes	No
				placebo	No	32	38.1 (9.7)	*	0-0	Fixed	0	HAMD 21	18.30								
Langlois 1985	1985	2	3	amitriptyline	No	15	*	*	150-225	Flexible	*	HAMD 17	*	4	DSM-III	North America	Single center	Secondary /Tertiary care	Outpatients only	No	Unclear
				placebo	No	15	*	*	0-0	Flexible	0	HAMD 17	*								
Lapierre 1980	1980	2	2	amitriptyline	Unclear	20	*	*	150-150	Fixed	150	*	*	4	Feighner	North America	Unclear	Secondary /Tertiary care	Both	Yes	Unclear
				trazodone	Unclear	20	*	*	150-150	Fixed	150	*	*								
Lapierre 1987	1987	2	3	fluvoxamine	Yes	22	43.3 (*)	*	150-300	Flexible	207	HAMD 17	24.84	6	DSM-III	North America	Multi-center	Secondary /Tertiary care	Inpatients only	Yes	Unclear
				placebo	No	20	45.8 (*)	*	0-0	Flexible	0	HAMD 17	21.98								
Larsen 1989	1989	2	3	clomipramine	Unclear	20	48 (*)	0.65	75-150	Fixed	150	HAMD 17	17.80	6	DSM-III	Europe	Unclear	Other/Unclear	Both	Unclear	Yes
				placebo	No	18	57 (*)	0.67	0-0	Fixed	0	HAMD 17	18.30								
Learned 2012a Study1 (EUCTR2005-003401-87, SND103285, NCT00448058)	2012	2	3	venlafaxine	No	134	43 (11.19)	0.61	150-225	Flexible	*	HAMD 17	*	10	DSM-IV	Cross-Continental	Multi-center	Other/Unclear	Outpatients only	Unclear	Unclear
				Placebo	No	126	41.9 (11.8)	0.64	0-0	Flexible	0	HAMD 17	*								
Learned 2012b Study2 (NCT00420641)	2012	2	3	paroxetine	Yes	166	44.4 (10.9)	0.33	30-30	Fixed	30	MADRS	30.30	10	DSM-IV	Cross-Continental	Multi-center	Other/Unclear	Outpatients only	Unclear	Unclear
				placebo	No	156	41.8 (10.9)	0.25	0-0	Fixed	0	MADRS	31.80								
Lecrubier 1997	1997	2	3	venlafaxine	Unclear	78	38.3 (*)	0.71	75-150	Flexible	128	MADRS	24.90	13	RDC	Europe	Multi-center	Other/Unclear	Outpatients only	Yes	Yes
				placebo	No	76	40.5 (*)	0.63	0-0	Flexible	0	MADRS	24.20								
Lee 2007 (F1J-AA-HMCV) (NCT00489775)	2007	2	2	duloxetine	Yes	238	39 (13.9)	*	60-60	Fixed	60	HAMD 17	21.10	8	DSM-IV	*	Multi-center	Secondary /Tertiary care	Both	No	Unclear
				paroxetine	No	240	38 (15.3)	*	20-20	Fixed	20	HAMD 17	21.20								
Leinonen 1997	1997	2	2	milnacipran	Yes	52	49.2 (9.8)	*	100-200	Flexible	*	HAMD 17	23.70	26	DSM-III-R	Europe	Multi-center	Secondary /Tertiary care	Both	Unclear	Yes
				clomipramine	No	55	47.1 (10.6)	*	75-150	Flexible	*	HAMD 17	23.10								

Leinonen 1999 (Agren1995)	1999	2	2	mirtazapine	Yes	137	42.1 (12.3)	*	15-60	Flexible	35	MADRS	29.60	8	DSM-IV	Europe	Multi-center	Secondary /Tertiary care	Both	Yes	Yes
				citalopram	No	133	41.1 (10.8)	*	20-60	Flexible	37	MADRS	29.10								
Lemoine 2007 (CL3-20098-035)	2004	2	4	agomelatine	Yes	165	40.7 (10.7)	0.75	25-50	Fixed	50	HAMD 17	25.90	24	DSM-IV	Europe	Multi-center	Primary care	Outpatients only	No	No
				venlafaxine	No	167	39.6 (10.3)	0.67	75-150	Fixed	150	HAMD 17	26.00								
Lepine 2000	2000	2	2	sertraline	Yes	82	42.3 (*)	0.71	50-200	Flexible	114	HAMD 17	29.8	8	DSM-III-R	Cross-Continental	Multi-center	Secondary /Tertiary care	Outpatients only	Yes	Yes
				clomipramine	No	84	41.8 (*)	0.69	50-150	Flexible	98	HAMD 17	29.6								
Lepola 2003 (ESC 99003)	2001	3	3	escitalopram	Yes	156	43 (11)	*	10-20	Flexible	*	MADRS	29.00	8	DSM-IV	Cross-Continental	Multi-center	Primary care	Outpatients only	Yes	Unclear
				citalopram	No	161	44 (11)	*	20-40	Flexible	*	MADRS	29.20								
				placebo	No	154	43 (12)	*	0-0	Flexible	0	MADRS	28.70								
Li 2006	2006	2	2	escitalopram	Unclear	28	36.5 (12.8)	0.55	10-20	Flexible	*	HAMD 17	23.70	6	Other operationalized	Asia	Single center	Secondary /Tertiary care	Outpatients only	Unclear	Yes
				citalopram	Unclear	28	34.4 (11.9)	0.53	20-40	Flexible	*	HAMD 17	23.80								
Li 2010	2010	2	2	escitalopram	Yes	24	*	*	10-20	Flexible	*	HAMD unspecified	*	6	Other operationalized	Asia	Multi-center	Other/Unclear	Other/Unclear	Unclear	Unclear
				citalopram	Yes	24	*	*	20-40	Flexible	*	HAMD unspecified	*								
Lieberman 2008a (Study 309, EUCTR2004-000562-13, 3151A1-309-EU, NCT00090649)	2008	2	3	placebo	No	120	45.4 (12)	0.67	0-0	Flexible	0	HAMD 17	*	8	DSM-IV	Europe	Multi-center	Other/Unclear	Outpatients only	Unclear	Unclear
				venlafaxine	Yes	127	45.9 (12)	0.72	75-150	Flexible	*	HAMD 17	25.8								
Lieberman 2008b (Study 317)	2008	2	3	placebo	No	125	39.5 (11.4)	0.64	0-0	Flexible	0	HAMD 17	*	8	DSM-IV	North America	Multi-center	Other/Unclear	Outpatients only	Unclear	Unclear
				venlafaxine	Yes	117	41.5 (11.6)	0.70	150-225	Flexible	*	HAMD 17	25.1								
Liebowitz 2008 (Study 332, NCT00277823)	2008	3	3	desvenlafaxine	Yes	151	43 (15)	0.62	50-50	Fixed	50	HAMD 17	23.37	8	DSM-IV	North America	Multi-center	Other/Unclear	Outpatients only	Unclear	Yes
				desvenlafaxine	Yes	148	43 (13)	0.53	100-100	Fixed	100	HAMD 17	23.35								
				placebo	No	152	42 (14)	0.63	0-0	Fixed	0	HAMD 17	23.02								
Liebowitz 2013 (NCT00863798)	2013	2	3	desvenlafaxine	Yes	227	43 (14)	0.60	50-50	Fixed	50	HAMD 17	23	8	DSM-IV	North America	Multi-center	Both	Outpatients only	Yes	Unclear

				placebo	No	227	42 (13)	0.62	0-0	Fixed	0	HAMD 17	23								
Lineberry 1990 (WELL 84A)	1990	2	2	bupropion	Yes	112	41.9 (*)	*	300-300	Fixed	300	HAMD 21	26.54	6	DSM-III-R	North America	Multi-center	Secondary /Tertiary care	Outpatients only	Yes	Yes
				placebo	No	112	40.8 (*)	*	0-0	Fixed	0	HAMD 21	27.02								
Loo 2002 (CL2-014)	2002	3	5	agomelatine	Yes	137	42.3 (*)	0.67	25-25	Fixed	25	HAMD 17	27.40	8	DSM-IV	Europe	Multi-center	Both	Other/Unclear	Yes	Yes
				placebo	No	139	42.3 (*)	0.67	0-0	Fixed	0	HAMD 17	27.40								
				paroxetine	No	147	42.3 (*)	0.67	20-20	Fixed	20	HAMD 17	27.30								
LopezRodriguez 2004	2004	2	4	fluoxetine	Unclear	10	31.9 (*)	*	20-20	Fixed	20	HAMD 21	17.27	20	DSM-IV	South America	Single center	Primary care	Outpatients only	No	Unclear
				placebo	No	10	31.9 (*)	*	0-0	Fixed	0	HAMD 21	17.27								
Lv 2013	2013	2	2	escitalopram	Unclear	20	32.1 (7.64)	0.50	10-20	Flexible	*	HAMD 17	23.06	6	Other operationalized	Asia	Single center	Secondary /Tertiary care	Outpatients only	Unclear	Yes
				citalopram	Unclear	22	31.61 (8.61)	0.50	20-40	Flexible	*	HAMD 17	22.78								
Lydiard 1989	1989	2	3	fluvoxamine	Yes	18	47.2 (*)	*	100-300	Flexible	240	HAMD unspecified	24.50	6	DSM-III	North America	Single center	Secondary /Tertiary care	Outpatients only	Yes	Unclear
				placebo	No	18	47.2 (*)	*	0-0	Flexible	0	HAMD unspecified	26.00								
Lydiard 1997	1997	3	3	amitriptyline	No	131	39 (*)	*	50-150	Flexible	103	HAMD 17	22.1	8	DSM-III-R	North America	Multi-center	Other/Unclear	Outpatients only	Yes	Yes
				placebo	No	129	40.2 (*)	*	0-0	Flexible	0	HAMD 17	22.1								
				sertraline	No	132	41.2 (*)	*	50-200	Flexible	139	HAMD 17	21.5								
M/2020/0046 (Study 046)	unpublished	3	3	reboxetine	Yes	265	39.9 (11.1)	0.71	8-10	Flexible	9	HAMD 21	23.00	8	DSM-IV	North America	Multi-center	Other/Unclear	Other/Unclear	No	Yes
				placebo	No	257	39 (11.6)	0.70	0-0	Flexible	0	HAMD 21	23.00								
				paroxetine	No	265	39.8 (11.8)	0.69	20-40	Flexible	29	HAMD 21	22.80								
M/2020/0047 (Study 047)	unpublished	3	3	reboxetine	Yes	258	39.3 (11.6)	0.74	8-10	Flexible	9	HAMD 21	24.20	8	DSM-IV	North America	Multi-center	Other/Unclear	Other/Unclear	No	Yes
				placebo	No	254	37.1 (11)	0.82	0-0	Flexible	0	HAMD 21	23.70								
				paroxetine	No	262	39.8 (10.8)	0.72	20-40	Flexible	33	HAMD 21	23.90								
Mahableshwarkar 2013 (304, NCT00672620)	2013	3	4	placebo	No	153	42.6 (13.7)	0.61	0-0	Fixed	0	HAMD 24	29.50	8	DSM-IV	North America	Multi-center	Other/Unclear	Other/Unclear	Unclear	Unclear
				vortioxetine	Yes	153	43.1 (13.9)	0.69	5-5	Fixed	5	HAMD 24	29.80								
				duloxetine	No	152	42.7 (14.4)	0.60	60-60	Fixed	60	HAMD 24	28.70								
	2015	4	4	placebo	No	161	42.4 (12.6)	0.72	0-0	Fixed	0	MADRS	31.60	8	DSM-IV	North America	Multi-center	Other/Unclear	Other/Unclear	Unclear	No

Mahableshwarkar 2015a (315, NCT01153009)				vortioxetine	Yes	147	43.1 (12.3)	0.71	15-15	Fixed	15	MADRS	31.90								
				vortioxetine	Yes	154	42.8 (12.4)	0.74	20-20	Fixed	20	MADRS	32.00								
				duloxetine	No	152	43.4 (12.2)	0.78	60-60	Fixed	60	MADRS	32.90								
Mahableshwarkar 2015b (317, NCT01179516)	2015	3	3	placebo	No	160	46.2 (11.8)	0.68	0-0	Fixed	0	MADRS	33.40	8	DSM-IV	North America	Multi-center	Other/Unclear	Other/Unclear	Unclear	Unclear
				vortioxetine	Yes	157	45.2 (11.9)	0.72	10-10	Fixed	10	MADRS	34.10								
				vortioxetine	Yes	152	43.8 (13.5)	0.71	15-15	Fixed	15	MADRS	33.70								
Mahableshwarkar 2015c (NCT01564862)	2015	3	3	vortioxetine	Yes	198	44.2 (12.2)	0.68	10-20	Flexible	16	MADRS	31.40	8	DSM-IV	Cross-Continental	Multi-center	Secondary /Tertiary care	Both	Unclear	Unclear
				duloxetine	No	210	45.7 (11.5)	0.66	60-60	Fixed	60	MADRS	31.70								
				placebo	No	194	45 (12.1)	0.61	0-0	Fixed	0	MADRS	31.90								
Mann 1981	1981	2	3	trazodone	Yes	10	44.5 (3.9)	0.10	200-600	Flexible	418	HAMD unspecified	26.30	4	Feighner	North America	Single center	Secondary /Tertiary care	Outpatients only	Yes	Unclear
				placebo	No	10	47.2 (4.1)	0.11	0-0	Flexible	0	HAMD unspecified	22.90								
Manna 1989	1989	2	2	fluoxetine	Unclear	15	47.7 (10)	0.63	20-20	Fixed	20	HAMD 17	25.7	5	DSM-III	Europe	Unclear	Secondary /Tertiary care	Inpatients only	Unclear	Unclear
				clomipramine	Unclear	15	48.5 (8.36)	0.63	75-75	Fixed	75	HAMD 17	25								
Mao 2008	2008	2	2	fluoxetine	No	117	40.7 (13.9)	0.74	20-20	Fixed	20	HAMD 17	24.10	8	DSM-IV	Asia	Multi-center	Secondary /Tertiary care	Both	No	Yes
				escitalopram	Yes	123	37.1 (14.1)	0.61	10-10	Fixed	10	HAMD 17	24.70								
Mao 2015 (NCT01098318)	2015	2	3	sertraline	No	19	41.4 (14.6)	0.53	50-200	Flexible	*	HAMD 17	15.40	12	DSM-IV	North America	Single center	Primary care	Outpatients only	Unclear	Unclear
				placebo	No	18	46.7 (15.2)	0.44	0-0	Fixed	0	HAMD 17	14.40								
March 1990	1990	2	3	fluvoxamine	Yes	18	39.4 (*)	*	100-300	Flexible	*	HAMD unspecified	25.10	6	DSM-III	North America	Single center	Secondary /Tertiary care	Outpatients only	Yes	Unclear
				placebo	No	18	39.4 (*)	*	0-0	Flexible	0	HAMD unspecified	27.30								
Marchesi 1998	1998	2	2	fluoxetine	Yes	67	43.6 (11.9)	0.73	20-20	Fixed	20	HAMD 17	25.50	10	DSM-III-R	Europe	Multi-center	Secondary /Tertiary care	Outpatients only	Yes	Yes
				amitriptyline	No	75	43.6 (11.9)	0.75	75-225	Flexible	115	HAMD 17	25.30								
Masco 1985	1985	2	2	fluoxetine	Yes	21	51 (*)	0.65	40-80	Flexible	*	HAMD unspecified	*	6	*	North America	Unclear	Secondary /Tertiary care	Outpatients only	Unclear	Unclear
				amitriptyline	No	21	51 (*)	0.81	150-300	Flexible	*	HAMD unspecified	*								
Mathews 2015 (NCT01473381)	2015	4	4	vilazodone	Yes	292	41.7 (12.7)	0.58	20-20	Fixed	17	MADRS	31.00	10	DSM-IV	North America	Multi-center	Other/Unclear	Outpatients only	Unclear	Yes

				vilazodone	Yes	291	40.8 (13.2)	0.57	40-40	Fixed	30	MADRS	30.80								
				placebo	No	290	42 (13)	0.56	0-0	Fixed	0	MADRS	31.30								
				citalopram	No	289	42.6 (12.6)	0.59	40-40	Fixed	40	MADRS	31.10								
McGrath 2000	2000	2	2	fluoxetine	Yes	49	41.6 (11)	*	20-60	Flexible	51	HAMD 17	*	10	DSM-IV	North America	Single center	Secondary /Tertiary care	Outpatients only	Yes	Unclear
				placebo	No	52	41.6 (11)	*	0-0	Flexible	0	HAMD 17	*								
McIntyre 2014 (NCT01422213)	2014	3	3	placebo	No	198	45.6 (12.1)	0.66	0-0	Fixed	0	MADRS	31.34	8	DSM-IV	Cross-Continental	Multi-center	Secondary /Tertiary care	Both	Unclear	No
				vortioxetine	Yes	197	45.4 (12.2)	0.69	10-10	Fixed	10	MADRS	31.62								
				vortioxetine	Yes	207	46.1 (11.8)	0.64	20-20	Fixed	20	MADRS	31.74								
McPartlin 1998	1998	2	2	paroxetine	No	178	44 (14)	*	20-20	Fixed	20	HAMD 17	23.00	12	DSM-IV	Europe	Multi-center	Primary care	Outpatients only	Unclear	Yes
				venlafaxine	Yes	183	45 (15)	*	75-75	Fixed	75	HAMD 17	23.00								
MDF/29060/III/070/88/MC	unpublished	2	2	paroxetine	Yes	32	73 (*)		20-30	Flexible	*	MADRS	30.88	5	Feighner	Europe	Multi-center	Other/Unclear	Outpatients only	Yes	Yes
				clomipramine	No	30	73 (*)		60-75	Flexible	*	MADRS	29.00								
MDUK/26090/III/83/007	unpublished	2	3	paroxetine	Yes	4	*	0.50	15-30	Flexible	*	*	*	4	Feighner	Europe	Single center	Secondary /Tertiary care	Inpatients only	Unclear	Unclear
				placebo	No	3	*	0.67	0-0	Flexible	0	*	*								
Mehtonen 2000	2000	2	2	sertraline	No	72	41 (10.7)	*	50-100	Flexible	*	HAMD 21	25.80	8	DSM-IV	Europe	Multi-center	Other/Unclear	Outpatients only	No	Unclear
				venlafaxine	Yes	75	44.1 (11.4)	*	75-150	Flexible	*	HAMD 21	25.50								
Mendels 1995 (FDA 03A0A-004B)	1995	3	3	nefazodone	Yes	80	38.4 (*)	0.64	50-300	Flexible	247	HAMD 17	25.10	6	DSM-III	North America	Multi-center	Other/Unclear	Other/Unclear	Unclear	Unclear
				nefazodone	Yes	80	39.1 (*)	0.60	100-600	Flexible	397	HAMD 17	25.40								
				placebo	No	80	40.1 (*)	0.61	0-0	Flexible	0	HAMD 17	25.00								
Mendels 1999 (Study 85A - FDA)	1999	2	2	citalopram	No	89	43.3 (*)	*	20-80	Flexible	52	HAMD 24	33.52	4	DSM-III	North America	Multi-center	Secondary /Tertiary care	Outpatients only	Yes	Yes
				placebo	No	91	42.7 (*)	*	0-0	Flexible	0	HAMD 24	33.72								
Miller 1989 (MDUK/29060/III/82/006 (PAR-274) PAR UK 06 - FDA)	1989	2	2	paroxetine	Yes	22	41.6 (13.2)	*	30-30	Fixed	30	HAMD 21	23.70	4	Feighner	Europe	Single center	Other/Unclear	Outpatients only	Unclear	Unclear
				placebo	No	25	43.3 (11.8)	*	0-0	Fixed	0	HAMD 21	24.20								
MIR 003-003 (FDA)	unpublished	2	2	mirtazapine	Yes	45	43 (*)	0.47	*-35	Flexible	27	HAMD 21	25.40	6	*	North America	Unclear	Other/Unclear	Other/Unclear	Yes	Yes
				placebo	No	45	43 (*)	0.67	0-0	Flexible	0	HAMD 21	25.50								

MIR 003-020 (FDA)	unpublished	3	3	mirtazapine	Yes	44	44 (*)	0.46	5-35	Flexible	24	HAMD 21	27.80	6	*	North America	Unclear	Other/Unclear	Outpatients only	Yes	Yes
				amitriptyline	No	43	43 (*)	0.45	40-280	Flexible	137	HAMD 21	29.20								
				placebo	No	43	45 (*)	0.62	0-0	Flexible	0	HAMD 21	29.50								
MIR 003-021 (FDA)	unpublished	3	3	mirtazapine	Yes	50	45 (*)	0.56	5-35	Flexible	21	HAMD 21	24.20	6	*	North America	Unclear	Other/Unclear	Outpatients only	Yes	Yes
				amitriptyline	No	50	44 (*)	0.54	40-280	Flexible	116	HAMD 21	25.00								
				placebo	No	50	48 (*)	0.56	0-0	Flexible	0	HAMD 21	24.40								
MIR 84062 (FDA)	unpublished	3	3	mirtazapine	Yes	15	*	*	*	Flexible	*	HAMD 17	*	6	DSM-III	Europe	Single center	Other/Unclear	Outpatients only	Unclear	Unclear
				amitriptyline	No	15	*	*	*	Flexible	*	HAMD 17	*								
				placebo	No	15	*	*	0-0	Flexible	0	HAMD 17	*								
Mischoulon 2014 (NCT00101452)	2014	2	3	escitalopram	No	69	45 (15)	0.50	10-20	Flexible	*	HAMD 17	19.25	12	DSM-IV	North America	Multi-center	Other/Unclear	Outpatients only	Unclear	Unclear
				placebo	No	65	45 (15)	0.50	*	Flexible	*	HAMD 17	19.43								
Miura 2000	2000	2	2	paroxetine	Yes	109	45.9 (13.9)	*	20-40	Flexible	32	HAMD 17	23.80	6	DSM-IV	Asia	Multi-center	Secondary /Tertiary care	Both	No	Yes
				amitriptyline	No	119	47.1 (13.8)	*	50-150	Flexible	109	HAMD 17	23.90								
Moises 1981	1981	2	2	amitriptyline	Unclear	22	48.7 (*)	0.86	150-200	Flexible	164	HAMD unspecified	24.9	4	Other operationalized	Europe	Single center	Secondary /Tertiary care	Inpatients only	Unclear	Yes
				trazodone	Unclear	21	48.7 (*)	0.86	450-600	Flexible	512	HAMD unspecified	25.3								
Moller 1993 (Stuppaeck 1994, MY-059.073/29060/1)	1993	2	2	paroxetine	Yes	78	47.5 (11.6)	*	30-50	Flexible	33	HAMD 21	28.60	6	DSM-III	Europe	Multi-center	Secondary /Tertiary care	Inpatients only	Yes	Yes
				amitriptyline	No	75	46.6 (11.4)	*	150-250	Flexible	166	HAMD 21	28.90								
Moller 1998 (Hegerl 1997)	1998	2	2	amitriptyline	No	79	49.5 (12.9)	0.71	75-225	Flexible	141	HAMD 21	29.1	6	DSM-III-R	Europe	Multi-center	Secondary /Tertiary care	Inpatients only	No	Yes
				sertraline	Yes	81	47.7 (13)	0.69	50-150	Flexible	105	HAMD 21	28.3								
Moller 2000	2000	2	2	sertraline	Yes	116	47.7 (13.2)	*	50-100	Flexible	55	HAMD 21	27.10	6	DSM-III-R	Europe	Multi-center	Both	Outpatients only	No	Yes
				amitriptyline	No	124	48.1 (14)	*	75-150	Flexible	87	HAMD 21	27.50								
Montgomery 1992 (Study 89303 FDA)	unpublished	3	3	citalopram	Yes	70	*	*	20-20	Fixed	20	HAMD 17	24.29	6	DSM-III-R	*	Multi-center	Other/Unclear	Both	Yes	Yes
				citalopram	Yes	64	*	*	40-40	Fixed	40	HAMD 17	22.98								
				placebo	No	66	*	*	0-0	Fixed	0	HAMD 17	23.72								
Montgomery 2004a (Study 99067)	2004	2	2	escitalopram	Yes	148	49 (15)	*	10-20	Flexible	12	HAMD 17	19.99	8	DSM-IV	Europe	Multi-center	Primary care	Other/Unclear	No	Unclear
				venlafaxine	No	145	47 (14)	*	75-150	Flexible	95	HAMD 17	20.44								

Montgomery 2004b (CL3-20098-030)	2001	2	2	agomelatine	Yes	167	42.8 (13.5)	*	25-25	Fixed	25	MADRS	23.10	12	DSM-IV	Cross-Continental	Multi-center	Primary care	Outpatients only	No	No
				paroxetine	No	168	42.1 (13.4)	*	20-20	Fixed	20	MADRS	23.00								
Montgomery 2013 (F02695LP202, EudraCT2006-002404-34)	2013	2	2	placebo	No	281	45 (*)	0.66	0-0	Flexible	0	HAMD 17	25.80	10	DSM-IV	Cross-Continental	Multi-center	Other/Unclear	Outpatients only	Unclear	Yes
				levomilnacipran	Yes	282	44 (*)	0.67	75-100	Flexible	65	HAMD 17	26.20								
Moon 1994	1994	2	2	sertraline	Yes	51	42 (*)	0.41	50-150	Flexible	64	HAMD 17	23.9	6	DSM-III	Europe	Multi-center	Primary care	Outpatients only	Yes	Yes
				clomipramine	No	55	45 (*)	0.60	50-150	Flexible	61	HAMD 17	22.7								
Moore 2005	2005	2	2	escitalopram	Yes	142	44.1 (10.9)	*	20-20	Fixed	20	MADRS	36.30	8	DSM-IV	Europe	Multi-center	Both	Outpatients only	No	Unclear
				citalopram	No	152	46.2 (11.1)	*	40-40	Fixed	40	MADRS	35.70								
Moreno 2005	2005	2	3	fluoxetine	No	20	37.7 (9.9)	0.83	20-20	Fixed	20	HAMD 21	15.41	8	DSM-IV	South America	Single center	Secondary /Tertiary care	Outpatients only	Yes	Yes
				placebo	No	26	45.9 (10.8)	0.83	0-0	Fixed	0	HAMD 21	16.49								
Moscovitch 2004	2004	2	2	sertraline	Yes	93	39.6 (11.6)	*	50-200	Flexible	111	HAMD 17	18.62	8	DSM-III-R	Cross-Continental	Multi-center	Secondary /Tertiary care	Outpatients only	Yes	Yes
				placebo	No	94	40 (11.2)	*	0-0	Flexible	0	HAMD 17	17.76								
Mullin 1996	1996	2	2	mirtazapine	Yes	79	45.4 (11.8)	*	20-60	Flexible	*	HAMD 17	22.50	5	DSM-III	Europe	Multi-center	Secondary /Tertiary care	Outpatients only	Yes	Yes
				amitriptyline	No	77	44.2 (10.3)	*	75-225	Flexible	*	HAMD 17	22.60								
Mundt 2012 (NCT00406952)	2012	2	2	sertraline	Yes	80	37.8 (12.5)	0.63	50-100	Flexible	*	HAMD 17	24.90	4	DSM-IV	North America	Multi-center	Other/Unclear	Outpatients only	Unclear	Unclear
				placebo	No	85	37.8 (12.5)	0.63	0-0	Flexible	0	HAMD 17	24.60								
Munizza 2006	2006	2	2	sertraline	No	60	46.9 (10.6)	0.70	50-100	Flexible	59	HAMD 17	21.9	6	DSM-IV	Europe	Multi-center	Other/Unclear	Outpatients only	Unclear	Yes
				trazodone	Yes	62	45 (11.5)	0.60	150-450	Flexible	297	HAMD 17	21.7								
Murasaki 1998	1998	2	2	fluvoxamine	Yes	113	40.5 (*)	*	50-150	Flexible	87	HAMD 21	22.90	4	DSM-III-R	Asia	Multi-center	Both	Both	No	Yes
				amitriptyline	No	122	45.3 (*)	*	50-150	Flexible	76	HAMD 21	23.60								
Murasaki 2010a (iQWIG22532, Schoemaker 2002)	2002	2	2	mirtazapine	Yes	104	42 (12.1)	0.68	15-45	Flexible	23	HAMD 17	25.10	6	DSM-IV	Europe	Multi-center	Both	Outpatients only	No	Yes
				fluvoxamine	No	105	39.4 (12.7)	0.64	50-150	Flexible	78	HAMD 17	25.10								
Murasaki 2010b (iQWIG9902, Schoemaker 2002)	2002	2	2	mirtazapine	Yes	101	40.7 (11.8)	0.50	15-45	Flexible	*	HAMD 17	22.90	6	DSM-IV	Asia	Multi-center	Both	Outpatients only	No	Yes
				fluvoxamine	No	102	42.2 (12.9)	0.39	50-150	Flexible	*	HAMD 17	22.50								
	unpublished	2	3	paroxetine	Yes	4	49.3 (*)	1.00	30-30	Fixed	30	HAMD 21	*	4	DSM-IV	Europe	Multi-center	Other/Unclear	Other/Unclear	Unclear	Unclear

MY-1008/BRL-029060/2/CPMS-076				placebo	No	4	46.5 (*)	0.50	0-0	Fixed	0	HAMD 21	*								
MY-1042/BRL-029060/CPMS-251	unpublished	2	2	paroxetine	Yes	125	41.7 (11.3)	*	20-50	Flexible	*	HAMD unspecified	24.47	8	DSM-III-R	North America	Multi-center	Other/Unclear	Outpatients only	Yes	Unclear
				placebo	No	129	42 (10.5)	*	0-0	Flexible	0	HAMD unspecified	24.35								
MY-1043/BRL-029060/115	unpublished	3	3	paroxetine	Yes	284	42.3 (12.5)	*	20-50	Flexible	*	HAMD 21	*	12	DSM-III-R	North America	Multi-center	Other/Unclear	Outpatients only	Yes	Unclear
				fluoxetine	No	289	41.7 (11.3)	*	20-80	Flexible	*	HAMD 21	*								
				placebo	No	118	42.1 (11.6)	*	0-0	Flexible	0	HAMD 21	*								
MY-1045/BRL-029060/1 (PAR 128)	unpublished	3	3	paroxetine	Yes	357	42.3 (12.6)	*	20-50	Flexible	*	HAMD 21	25.60	12	DSM-III-R	North America	Multi-center	Other/Unclear	Outpatients only	Yes	Unclear
				fluoxetine	No	351	40.6 (11.6)	*	20-80	Flexible	*	HAMD 21	25.60								
				placebo	No	140	43.3 (12.2)	*	0-0	Flexible	0	HAMD 21	25.70								
Mynors-Wallis 1995	1995	2	3	amitriptyline	No	31	37.2 (11.4)	*	150-150	Fixed	139	HAMD 17	19.1	12	RDC	Europe	Single center	Primary care	Outpatients only	Unclear	Unclear
				placebo	No	30	37 (10.4)	*	0-0	Fixed	0	HAMD 17	18.4								
NCT00822744 (EudraCT Number2008-001718-26)	unpublished	2	5	escitalopram	Unclear	103	*	*	10-10	Fixed	10	HAMD 17	*	8	DSM-IV	Cross-Continental	Multi-center	Other/Unclear	Other/Unclear	Unclear	Unclear
				placebo	No	106	*	*	0-0	Fixed	0	HAMD 17	*								
NCT01020799	unpublished	2	3	escitalopram	No	50	36.22 (11.3)	0.44	20-20	Fixed	20	HAMD unspecified	24.40	4	DSM-IV	North America	Multi-center	Other/Unclear	Other/Unclear	Unclear	Unclear
				placebo	No	99	41.77 (10.0)	0.49	0-0	Fixed	0	HAMD unspecified	24.60								
NCT01145755	unpublished	2	3	duloxetine	No	47	39.9 (*)	0.51	60-60	Fixed	60	MADRS	*	6	*	North America	Multi-center	Other/Unclear	Other/Unclear	Unclear	Unclear
				placebo	No	44	38.4 (*)	0.68	0-0	Fixed	0	MADRS	*								
NCT01254305	Unpublished	2	3	placebo	No	93	41.4 (12)	0.65	0-0	Flexible	0	*	*	8	DSM-IV	North America	Multi-center	Other/Unclear	Other/Unclear	Yes	Unclear
				levomilnacipran	Yes	90	42.9 (12.6)	0.58	40-120	Flexible	*	*	*								
NCT01255787 (EUCTR2010-022257-41, Lu AA21004/CCT-002, U1111-1117-6595, JapicCTI-101344, CTRI/2011/08/001963)	Unpublished	4	4	placebo	No	152	43.6 (11.6)	0.60	0-0	Fixed	0	MADRS	31.60	8	DSM-IV	Cross-Continental	Multi-center	Other/Unclear	Other/Unclear	Unclear	Unclear
				vortioxetine	Yes	144	44.2 (11.9)	0.68	5-5	Fixed	5	MADRS	31.60								
				vortioxetine	Yes	150	45.7 (10.9)	0.62	10-10	Fixed	10	MADRS	31.80								
				vortioxetine	Yes	154	44 (11.8)	0.60	20-20	Fixed	20	MADRS	31.70								
NCT01355081(japicCTI-111492, U1111-1120-9277)	Unpublished	3	3	vortioxetine	Yes	119	38.8 (10.8)	0.42	5-5	Fixed	5	HAMD 17	20.90	8	DSM-IV	Asia	Multi-center	Other/Unclear	Other/Unclear	Unclear	Unclear
				vortioxetine	Yes	123	38.8 (11)	0.44	10-10	Fixed	10	HAMD 17	21.20								
				placebo	No	124	37.6 (10.7)	0.54	0-0	Fixed	0	HAMD 17	21.50								

NCT01808612	unpublished	3	3	fluoxetine	Yes	169	40.5 (11.2)	0.49	20-20	Fixed	20	HAMD 21	*	6	*	Asia	Multi-center	Other/Unclear	Outpatients only	Unclear	Unclear
				fluoxetine	Yes	84	39.58 (10.6)	0.40	40-40	Fixed	40	HAMD 21	*								
				placebo	No	260	38.54 (11.6)	0.50	0-0	Fixed	0	HAMD 21	*								
Nemeroff 1995	1995	2	2	fluvoxamine	Yes	49	38.5 (*)	*	50-150	Flexible	124	HAMD 21	24.57	7	DSM-III-R	North America	Multi-center	Secondary /Tertiary care	Outpatients only	Yes	Yes
				sertraline	No	48	41.2 (*)	*	50-200	Flexible	137	HAMD 21	23.15								
Nemeroff 2007	2007	3	3	fluoxetine	Unclear	104	37.9 (11.5)	*	20-60	Flexible	41	HAMD 21	23.70	6	DSM-IV	North America	Multi-center	Secondary /Tertiary care	Outpatients only	Yes	Unclear
				venlafaxine	Yes	102	40.1 (11.1)	*	75-225	Flexible	142	HAMD 21	23.50								
				placebo	No	102	40.4 (11.7)	*	0-0	Flexible	0	HAMD 21	23.70								
Newhouse 2000	2000	2	2	sertraline	Yes	117	68 (5.3)	*	50-100	Flexible	72	HAMD 24	25.10	12	DSM-III-R	North America	Multi-center	Secondary /Tertiary care	Outpatients only	Yes	Yes
				fluoxetine	No	119	67 (5.9)	*	20-40	Flexible	29	HAMD 24	25.00								
Nierenberg 2007 (F1J-MC-HMCR, NCT00073411, Pigott 2007)	2007	3	3	escitalopram	No	274	43.3 (13)	*	10-10	Fixed	10	HAMD 17	17.80	8	DSM-IV	North America	Multi-center	Secondary /Tertiary care	Outpatients only	Yes	Unclear
				duloxetine	Yes	273	41.1 (11.6)	*	60-60	Fixed	60	HAMD 17	17.60								
				placebo	No	137	42.5 (12.3)	*	0-0	Fixed	0	HAMD 17	17.70								
Ninan 2003 (poster SCT-MD-26)	unpublished	2	2	escitalopram	Yes	154	37.8 (11.6)	*	10-20	Flexible	*	MADRS	30.40	8	DSM-IV	North America	Multi-center	Other/Unclear	Outpatients only	Unclear	Unclear
				placebo	No	155	39 (11.6)	*	0-0	Flexible	0	MADRS	30.50								
NKD20006 (NCT00048204)	unpublished	2	3	paroxetine	Yes	125	37.9 (11.9)	0.63	20-20	Fixed	20	HAMD 17	24.70	8	DSM-IV	North America	Multi-center	Other/Unclear	Outpatients only	Yes	No
				placebo	No	125	38 (11.3)	0.56	0-0	Fixed	0	HAMD 17	24.50								
Noguera 1991	1991	2	2	clomipramine	No	60	46 (*)	*	100-100	Fixed	100	HAMD unspecified	24.60	6	DSM-III	Europe	Multi-center	Secondary /Tertiary care	Other/Unclear	Yes	Yes
				fluoxetine	Yes	60	46.3 (*)	*	20-40	Flexible	*	HAMD unspecified	24.30								
Norton 1984	1984	2	3	fluvoxamine	Yes	35	39.2 (*)	*	100-*	Flexible	133	HAMD 17	19.50	4	RDC	Europe	Multi-center	Secondary /Tertiary care	Outpatients only	No	Yes
				placebo	No	25	38.8 (*)	*	0-0	Flexible	0	HAMD 17	19.90								
Oakes 2012a (NCT00536471)	2012	2	2	duloxetine	Yes	257	42.2 (12.2)	0.60	60-60	Fixed	60	HAMD 17	22.90	36	DSM-IV	North America	Multi-center	Other/Unclear	Other/Unclear	Unclear	Unclear
				placebo	No	127	43.7 (12.5)	0.61	0-0	Fixed	0	HAMD 17	22.80								
Oakes 2012b (NCT00536471)	2012	2	2	duloxetine	Yes	261	44.7 (12.2)	0.65	60-60	Fixed	60	HAMD 17	22.80	36	DSM-IV	North America	Multi-center	Other/Unclear	Outpatients only	Unclear	Unclear

				placebo	No	131	43.9 (11.9)	0.66	0-0	Fixed	0	HAMD 17	22.90								
Olie 1997	1997	2	2	sertraline	Yes	129	43.6 (11.4)	0.61	50-200	Flexible	*	HAMD 17	25.4	6	DSM-III-R	Europe	Unclear	Other/Unclear	Outpatients only	Yes	Yes
				placebo	No	129	43.9 (12.1)	0.65	0-0	Flexible	0	HAMD 17	25.5								
Olie 2007 (CL3-20098-042)	2004	2	2	agomelatine	Yes	118	44.3 (11.5)	0.72	25-50	Flexible	*	HAMD 17	27.40	6	DSM-IV	Europe	Multi-center	Secondary /Tertiary care	Both	No	No
				placebo	No	120	45.6 (11.1)	0.75	0-0	Flexible	0	HAMD 17	27.20								
Ontiveros 1994 (MY-0144/BRL 29060/1/CPMS-135, PAR135)	1994	2	2	paroxetine	No	60	43.1 (11)	*	20-20	Fixed	20	HAMD 21	26.20	6	DSM-III-R	North America	Multi-center	Other/Unclear	Outpatients only	Yes	No
				fluoxetine	No	62	38.7 (10.7)	*	20-20	Fixed	20	HAMD 21	26.40								
OntiverosSanchez 1998	1998	2	2	fluoxetine	Yes	21	38.6 (11.5)	0.43	20-20	Fixed	20	HAMD 17	23.50	6	DSM-III-R	South America	Single center	Secondary /Tertiary care	Outpatients only	Yes	Unclear
				amitriptyline	No	21	36.5 (9.34)	0.62	150-250	Flexible	160	HAMD 17	23.10								
Ottevanger 1995	1995	2	2	fluvoxamine	Yes	20	51.3 (15.39)	*	100-300	Flexible	204	HAMD 17	26.40	4	Feighner	Europe	Multi-center	Other/Unclear	Inpatients only	Unclear	Yes
				clomipramine	No	20	47.05 (12.4)	*	50-150	Flexible	106	HAMD 17	25.70								
Ou 2010	2010	2	2	citalopram	Unclear	120	36.4 (12.3)	0.54	20-40	Flexible	28	HAMD 17	22.9	6	DSM-IV	Asia	Multi-center	Secondary /Tertiary care	Both	Unclear	Yes
				escitalopram	Unclear	120	36.7 (12.5)	0.58	10-20	Flexible	14	HAMD 17	23.4								
PAR 01 001 (GSK & FDA)	1989	2	2	paroxetine	Yes	25	42.1 (*)	0.35	10-50	Flexible	27	HAMD 21	27.90	6	DSM-III	North America	Single center	Other/Unclear	Outpatients only	Yes	Unclear
				placebo	No	25	44.1 (*)	0.35	0-0	Flexible	0	HAMD 21	27.50								
PAR 279 MDUK	unpublished	2	3	paroxetine	Yes	19	39.7 (18.2)	0.68	30-30	Fixed	30	HAMD unspecified	22.80	6	*	Europe	Multi-center	Other/Unclear	Other/Unclear	Yes	Unclear
				placebo	No	10	51.6 (17.3)	0.70	0-0	Fixed	0	HAMD unspecified	22.30								
PAR 29060.308 (HP/81/74A)	unpublished	2	2	paroxetine	Yes	10	43.3 (12.6)	0.60	30-30	Fixed	30	*	*	6	*	Europe	Single center	Secondary /Tertiary care	Inpatients only	Yes	Unclear
				amitriptyline	No	12	45.3 (12.5)	0.58	150-150	Fixed	150	*	*								
PAR 29060.310 (HP 81/85A)	unpublished	2	2	paroxetine	Yes	10	39.1 (11.9)	0.67	30-30	Fixed	30	*	*	7	*	Europe	Single center	Other/Unclear	Other/Unclear	Yes	Unclear
				amitriptyline	No	13	43.7 (10.2)	0.50	150-150	Fixed	150	*	*								
PAR 29060.314 (HP 82/134)	unpublished	2	2	paroxetine	Yes	10	46.8 (13.1)	0.30	30-30	Fixed	30	*	*	6	*	Europe	Single center	Other/Unclear	Other/Unclear	Yes	Unclear
				amitriptyline	No	8	41.8 (11.7)	0.75	150-150	Fixed	150	*	*								
PAR 29060.316 (HP/82/47A)	unpublished	2	2	paroxetine	Yes	9	47.2 (14.8)	0.78	30-30	Fixed	30	*	*	7	RDC	Europe	Multi-center	Secondary /Tertiary care	Inpatients only	Yes	Unclear

				amitriptyline	No	8	41 (11.7)	1.00	150-150	Fixed	150	*	*								
PAR 29060.318 (HP/82/64A)	unpublished	2	2	paroxetine	Yes	9	47.3 (10.1)	1.00	30-30	Fixed	30	*	*	7	*	Europe	Single center	Secondary Tertiary care	Inpatients only	Yes	Unclear
				amitriptyline	No	12	50.1 (8.2)	1.00	150-150	Fixed	150	*	*								
PAR 29060/281	unpublished	2	2	paroxetine	Yes	82	39.5 (13.1)	0.78	30-30	Fixed	30	HAMD 21	27.80	6	Feighner	Europe	Single center	Primary care	Other/Unclear	Yes	Unclear
				amitriptyline	No	80	38.1 (13.1)	0.75	75-150	Flexible	*	HAMD 21	26.60								
PAR MDUK 032	unpublished	2	2	paroxetine	Yes	29	42.6 (11.7)	*	20-30	Flexible	*	HAMD 17	24.00	6	DSM-III	*	Multi-center	Primary care	Outpatients only	Unclear	Unclear
				amitriptyline	No	30	46.2 (11.8)	*	100-150	Flexible	*	HAMD 17	23.20								
Patris 1996 (Study 91301 - FDA)	1996	2	2	fluoxetine	No	184	43 (*)	*	20-20	Fixed	20	MADRS	29.40	8	DSM-III-R	Europe	Multi-center	Primary care	Outpatients only	Yes	Unclear
				citalopram	Yes	173	44 (*)	*	20-20	Fixed	20	MADRS	29.70								
Paykel 1988	1988	2	2	amitriptyline	No	45	*	*	125-175	Flexible	111	HAMD 17	16.2	6	RDC	Europe	Multi-center	Primary care	Outpatients only	Unclear	Yes
				placebo	No	55	*	*	0-0	Flexible	0	HAMD 17	15.8								
Pelicier 1993 (PAR MDED/29060/III/86/1728M291)	1993	2	2	clomipramine	No	42	70.2 (8.9)		60-60	Fixed	60	MADRS	30.00	5	Feighner	Europe	Multi-center	Other/Unclear	Outpatients only	Unclear	Unclear
				paroxetine	Yes	41	71.1 (8)		20-20	Fixed	20	MADRS	29.50								
Perahia 2006 (HMAY - Study Group B)	2006	4	4	duloxetine	Yes	93	46.47 (12.7)	*	80-80	Fixed	80	HAMD 17	21.30	8	DSM-IV	Unspecified	Multi-center	Other/Unclear	Outpatients only	Yes	Unclear
				duloxetine	Yes	103	43.99 (10.8)	*	120-120	Fixed	120	HAMD 17	21.38								
				paroxetine	No	97	45.81 (10.6)	*	20-20	Fixed	20	HAMD 17	21.03								
				placebo	No	99	44.67 (10.1)	*	0-0	Fixed	0	HAMD 17	20.58								
Perahia 2008a (HMBU) (NCT00071695)	2008	2	2	duloxetine	Yes	166	45.42 (13)	0.72	60-60	Fixed	60	HAMD 17	23.10	6	DSM-IV	Cross-Continental	Multi-center	Other/Unclear	Outpatients only	Unclear	Unclear
				venlafaxine	No	166	42.62 (13.1)	0.69	150-150	Fixed	150	HAMD 17	23.10								
Perahia 2008b (HMCQ, NCT00067912)	2008	3	3	duloxetine	Yes	164	43.07 (12.6)	0.67	60-60	Fixed	60	HAMD 17	22.21	6	DSM-IV	Cross-Continental	Multi-center	Other/Unclear	Outpatients only	Unclear	Unclear
				venlafaxine	No	169	42.46 (12.4)	0.68	75-75	Fixed	75	HAMD 17	22.51								
				venlafaxine	No	171	40.64 (11.4)	0.62	150-150	Fixed	150	HAMD 17	22.24								
Pomara 2013	2013	2	2	vilazodone	Unclear	255	*	*	40-40	Fixed	40	MADRS	*	9	DSM-IV	North America	Multi-center	Other/Unclear	Outpatients only	Unclear	Unclear
				placebo	No	253	*	*	0-0	Fixed	0	MADRS	*								
Preskorn 1991	1991	2	2	fluoxetine	Yes	30	*	*	20-60	Flexible	*	HAMD unspecified	23.8	6	DSM-III	North America	Multi-center	Other/Unclear	Outpatients only	Yes	Yes
				amitriptyline	No	31	*	*	50-200	Flexible	*	HAMD unspecified	23.5								

PZ/109	unpublis hed	2	2	sertraline	Yes	104	*	0.60	*	Flexible	*	HAMD 17	22.00	8	*	*	*	*	*	*	*
				placebo	No	103	*	0.66	*	Flexible	*	HAMD 17	21.50								
PZ/111	unpublis hed	3	3	fluoxetine	No	108	*	0.67	*	Flexible	*	HAMD 17	24.40	8	*	*	*	*	*	*	*
				sertraline	Yes	106	*	0.75	*	Flexible	*	HAMD 17	24.1								
				placebo	No	105	*	0.65	*	Flexible	*	HAMD 17	24.2								
Quera-Salva 2010 (CL3-20098-056)	2010	2	2	agomelatine	Yes	71	41.3 (12.4)	*	25-50	Flexible	*	HAMD 17	26.10	25	DSM-IV	Cross-Continental	Multi-center	Primary care	Outpatients only	No	No
				escitalopram	No	67	41.4 (10.7)	*	10-20	Flexible	*	HAMD 17	26.10								
Raft 1981	1981	2	3	amitriptyline	No	12	*	*	100-300	Flexible	235	HAMD17	29.66	5	Feighne r	North America	Single center	Secondary /Tertiary care	Inpatients only	Unclear	Unclear
				placebo	No	7	*	*	0-0	Flexible	0	HAMD 17	27.2								
Rapaport 1996	1996	2	2	fluvoxamine	Yes	51	40 (*)	*	100-150	Flexible	102	HAMD 21	25.20	7	DSM-III-R	North America	Multi-center	Other/Unc lear	Outpatients only	Yes	Yes
				fluoxetine	No	49	38.6 (*)	*	20-80	Flexible	34	HAMD 21	25.60								
Rapaport 2003 (PAR487)	2003	3	3	paroxetine	Yes	104	70.39 (5.93)	*	12.5-50	Flexible	30	HAMD 17	22.10	12	DSM-IV	North America	Multi-center	Other/Unc lear	Outpatients only	Yes	Unclear
				paroxetine	Yes	106	70.05 (6.59)	*	10-40	Flexible	26	HAMD 17	22.30								
				placebo	No	109	69.39 (5.4)	*	0-0	Flexible	0	HAMD 17	22.10								
Rapaport 2009 (BRL-29060/874) (NCT00067444)	2009	2	3	paroxetine	Yes	177	67 (6.56)	*	25-25	Fixed	25	HAMD 17	22.74	10	DSM-IV	North America	Multi-center	Other/Unc lear	Outpatients only	Yes	Unclear
				placebo	No	180	68 (6.7)	*	0-0	Fixed	0	HAMD 17	22.41								
Raskin 2007 (HMBV, NCT00062673)	2007	2	2	duloxetine	Yes	207	72.63 (5.65)	0.60	60-60	Fixed	60	HAMD 17	18.76	8	DSM-IV	North America	Multi-center	Other/Unc lear	Other/Unclea r	Yes	Unclear
				placebo	No	104	73.33 (5.73)	0.58	0-0	Fixed	0	HAMD 17	18.94								
Ravindran 1995	1995	2	3	sertraline	Yes	40	38.8 (10.9)	0.65	50-200	Flexible	*	HAMD 17	20.7	8	DSM-III-R	North America	Multi-center	Secondary /Tertiary care	Outpatients only	Yes	Unclear
				placebo	No	26	39 (9.7)	0.58	0-0	Flexible	0	HAMD 17	21								
Ravindran 1997 (MY-1055/BRL-029060/1/CPM S-245 PAR 245)	1997	2	2	paroxetine	Yes	513	43.3 (13)	0.73	20-40	Flexible	*	MADRS	29.7	12	Other operati onalize d	Cross-Continental	Multi-center	Primary care	Other/Unclea r	Yes	Yes
				clomipramin e	No	506	42 (12.5)	0.73	75-150	Flexible	*	MADRS	29.1								
Reimherr 1990 (SER 104 - FDA)	1990	3	3	amitriptyline	No	149	37.9 (*)	*	50-150	Flexible	104	HAMD 17	23.18	8	DSM-III	North America	Multi-center	Secondary /Tertiary care	Outpatients only	Yes	No
				placebo	No	150	40.1 (*)	*	0-0	Flexible	0	HAMD 17	23.43								
				sertraline	Unclear	149	39 (*)	*	50-200	Flexible	145	HAMD 17	23.28								

Reimherr 1998 (WELL203, FDA203)	1998	2	3	placebo	No	121	40.2 (12.2)	*	0-0	Fixed	0	HAMD 17	23.21	8	DSM-III-R	North America	Multi-center	Other/Unclear	Outpatients only	Yes	Unclear
				bupropion	Yes	120	38.6 (10.7)	*	300-300	Fixed	300	HAMD 17	23.41								
Remick 1994	1994	2	2	amitriptyline	No	17	41 (*)	*	50-300	Flexible	135	HAMD 17	23.50	7	DSM-III	North America	Multi-center	Secondary /Tertiary care	Outpatients only	Yes	Unclear
				fluvoxamine	Yes	16	41.7 (*)	*	50-300	Flexible	175	HAMD 17	23.80								
Rickels 1982	1982	3	3	amitriptyline	No	68	40 (13)	*	75-200	Flexible	140	HAMD 21	26.46	6	DSM-III	North America	Single center	Both	Outpatients only	Unclear	Unclear
				placebo	No	68	40 (13)	*	0-0	Flexible	0	HAMD 21	26.46								
				trazodone	Unclear	66	40 (13)	*	150-400	Flexible	275	HAMD 21	26.46								
Rickels 1985	1985	2	4	amitriptyline	No	124	39 (11.7)	*	50-225	Flexible	148	HAMD 21	25.48	6	DSM-III	North America	Multi-center	Secondary /Tertiary care	Outpatients only	Yes	No
				placebo	No	130	39 (11.7)	*	0-0	Flexible	0	HAMD 21	26.38								
Rickels 1994 (FDA CN104-005)	1994	2	3	nefazodone	Yes	96	44.7 (13.5)	0.68	100-600	Flexible	375	HAMD 17	24.40	8	DSM-III-R	North America	Multi-center	Both	Outpatients only	Unclear	Yes
				placebo	No	95	42.6 (13.5)	0.60	0-0	Flexible	0	HAMD 17	23.50								
Rickels 1995 (FDA CN104-006-1)	1995	2	3	nefazodone	Yes	45	*	*	200-600	Flexible	419	HAMD 17	24.20	8	DSM-III-R	North America	Single center	Other/Unclear	Outpatients only	Unclear	No
				placebo	No	44	*	*	0-0	Flexible	0	HAMD 17	24.20								
Rickels 2009 (GNSC-04-DP-02, NCT00285376)	2009	2	2	vilazodone	Yes	205	40 (12.1)	*	40-40	Fixed	40	HAMD 17	24.80	8	DSM-IV	North America	Multi-center	Other/Unclear	Other/Unclear	Unclear	Unclear
				placebo	No	205	39.8 (12.7)	*	0-0	Fixed	0	HAMD 17	24.90								
Robinson 2014 (NCT00406848)	2014	2	2	duloxetine	Yes	249	73.01 (6.26)	0.66	60-60	Fixed	60	HAMD 17	19.40	24	DSM-IV	Cross-Continental	Multi-center	Other/Unclear	Other/Unclear	Yes	Unclear
				placebo	No	121	73.1 (5.64)	0.59	0-0	Fixed	0	HAMD 17	19.30								
Roffman 1982	1982	2	3	amitriptyline	No	95	42.55 (12.3)	*	75-225	Fixed	225	HAMD 21	24.2	4	DSM-III	North America	Multi-center	Other/Unclear	Outpatients only	Yes	Yes
				placebo	No	94	44.66 (12.1)	*	0-0	Fixed	0	HAMD 21	24.5								
Roose 2004 (CIT-MD-03)	2004	2	2	citalopram	Yes	87	79.9 (4)	0.54	10-40	Flexible	*	HAMD 24	24.40	8	DSM-IV	North America	Multi-center	Other/Unclear	Outpatients only	Yes	Unclear
				placebo	No	91	79.3 (4.7)	0.62	0-0	Flexible	0	HAMD 24	24.20								
Ropert 1989	1989	2	2	fluoxetine	Unclear	71	42.7 (11)	0.60	20-20	Fixed	20	HAMD 21	23.00	6	DSM-III	Europe	Multi-center	Other/Unclear	Outpatients only	Unclear	Unclear
				clomipramine	No	72	44.8 (12.6)	0.69	75-75	Fixed	75	HAMD 21	23.00								
Rossini 2005	2005	2	2	fluvoxamine	Unclear	40	67.8 (*)	*	200-200	Fixed	200	HAMD 21	31.23	7	DSM-IV	Europe	Single center	Secondary /Tertiary care	Inpatients only	Unclear	Yes
				sertraline	Unclear	48	68.2 (*)	*	150-150	Fixed	150	HAMD 21	29.23								

Roth 1990	1990	2	3	fluvoxamine	Yes	30	42.8 (11.1)	0.67	100-300	Flexible	218	HAMD unspecified	28.3	6	DSM-III	North America	Multi-center	Secondary /Tertiary care	Outpatients only	Yes	Yes
				placebo	No	30	43.9 (11.3)	0.52	0-0	Flexible	0	HAMD unspecified	28.9								
Rouillon 1991 (Limosin 2006, 029060/1/CPM S 069 1991)	1991	2	2	paroxetine	Yes	45	71.8 (7.4)	0.87	20-50	Other/U nclear	*	HAMD 21	27	6	DSM-III	Europe	Multi-center	Secondary /Tertiary care	Inpatients only	Unclear	Unclear
				clomipramin e	No	47	70.8 (6.5)	0.91	50-150	Other/U nclear	*	HAMD 21	27.6								
Rowan 1980	1980	2	3	amitriptyline	No	44	*	*	75-187.5	Flexible	*	HAMD 17	*	6	RDC	Europe	Single center	Secondary /Tertiary care	Outpatients only	Yes	Unclear
				placebo	No	45	*	*	0-0	Flexible	0	HAMD 17	*								
Rudolph 1998 (VEN 600A-203 (FDA)	1998	4	4	venlafaxine	Yes	89	43.6 (10.9)	0.40	75-75	Fixed	75	HAMD 21	26.00	6	DSM-III	North America	Multi-center	Secondary /Tertiary care	Outpatients only	Yes	Yes
				venlafaxine	Yes	89	43.1 (9.61)	0.43	225-225	Fixed	225	HAMD 21	26.00								
				venlafaxine	Yes	88	43.8 (10.3)	0.33	375-375	Fixed	375	HAMD 21	24.90								
				placebo	No	92	41.5 (11.3)	0.32	0-0	Fixed	0	HAMD 21	25.30								
Rudolph 1999	1999	3	3	fluoxetine	No	103	40 (*)	*	20-60	Flexible	47	HAMD 21	26.00	8	DSM-IV	North America	Multi-center	Secondary /Tertiary care	Outpatients only	Yes	Unclear
				venlafaxine	Yes	100	40 (*)	*	75-225	Flexible	175	HAMD 21	25.00								
				placebo	No	98	40 (*)	*	0-0	Flexible	0	HAMD 21	25.00								
Rush 1998 (excluding Armitage 1997 and Gillin 1997)	1998	2	2	nefazodone	Yes	*	*	*	200-500	Flexible	*	HAMD 17	*	8	DSM-III-R	North America	Multi-center	Other/Unc lear	Outpatients only	Unclear	Unclear
				fluoxetine	No	*	*	*	20-40	Flexible	*	HAMD 17	*								
Rush 2001	2001	2	2	bupropion	Yes	122	39 (*)	0.48	100-300	Flexible	259	HAMD 21	24.8	16	DSM-IV	North America	Multi-center	Other/Unc lear	Outpatients only	Unclear	Unclear
				sertraline	No	126	40 (*)	0.48	50-200	Flexible	123	HAMD 21	24.8								
Sacchetti 2002 (BRL-29060/109)	2002	2	2	paroxetine	Yes	64	48.7 (*)	0.64	20-50	Flexible	*	HAMD 21	25.4	12	DSM-III-R	Europe	Multi-center	Secondary /Tertiary care	Outpatients only	Yes	Yes
				amitriptyline	No	65	50.5 (*)	0.66	100-250	Flexible	*	HAMD 21	26.2								
Sambunaris 2014 (LVM-MD-03, NCT01034462)	2014	2	2	placebo	No	220	44.6 (13.9)	0.66	0-0	Flexible	0	HAMD 17	22.90	8	DSM-IV	North America	Multi-center	Other/Unc lear	Outpatients only	Yes	Yes
				levomilnacip ran	Yes	222	45 (13.2)	0.65	40-120	Flexible	73	HAMD 17	23.30								
Samuelian 1998	1998	2	2	venlafaxine	Yes	52	47 (12)	*	100-150	Flexible	105	HAMD 21	27.80	6	DSM-III-R	Europe	Multi-center	Other/Unc lear	Outpatients only	Yes	Yes
				clomipramin e	No	50	47 (11)	*	100-150	Flexible	105	HAMD 21	27.80								
Sauer 2003	2003	2	2	venlafaxine	Yes	79	48.5 (10.8)	*	75-150	Flexible	85	HAMD 21	23.60	6	ICD-10	Europe	Multi-center	Both	Outpatients only	Unclear	No
				amitriptyline	No	77	46 (10.9)	*	75-150	Flexible	84	HAMD 21	23.64								

Schatzberg 2002 (003-901)	2002	2	2	mirtazapine	Yes	128	71.7 (5.7)	*	15-45	Flexible	34	HAMD 17	22.20	8	DSM-IV	North America	Multi-center	Other/Unclear	Outpatients only	Unclear	Yes
				paroxetine	No	126	72 (5.1)	*	20-40	Flexible	34	HAMD 17	22.40								
Schatzberg 2006a	2006	3	3	fluoxetine	No	100	71 (*)	*	20-60	Flexible	*	HAMD 21	24.00	8	DSM-IV	North America	Multi-center	Secondary /Tertiary care	Outpatients only	Yes	Yes
				venlafaxine	Yes	104	71 (*)	*	75-225	Flexible	*	HAMD 21	24.00								
			3	placebo	No	96	71 (*)	*	0-0	Flexible	0	HAMD 21	23.00								
Schneider 2003	2003	2	2	sertraline	Yes	371	70 (6.8)	0.54	50-100	Flexible	82	HAMD 17	21.40	8	DSM-IV	North America	Multi-center	Both	Outpatients only	Yes	Yes
				placebo	No	376	69.6 (6.5)	0.58	0-0	Flexible	0	HAMD 17	21.40								
Schoene 1993 (Geretsegger 1994 MY1021/BRC)	1993	2	2	paroxetine	Yes	54	74.3 (6)	*	20-50	Flexible	*	HAMD 21	29.00	6	DSM-III-R	Europe	Multi-center	Other/Unclear	Outpatients only	Yes	Unclear
				fluoxetine	No	52	73.7 (6.4)	*	20-80	Flexible	*	HAMD 21	27.90								
Schwartz 2002 (iQWIG)	2002	2	2	reboxetine	Unclear	80	41.9 (*)	0.71	8-10	Flexible	*	HAMD 17	28.60	8	DSM-IV	Cross-Continental	Multi-center	Other/Unclear	Other/Unclear	Unclear	Unclear
				venlafaxine	Unclear	87	42.2 (*)	0.67	225-375	Flexible	*	HAMD 17	28.80								
Schweizer 1994 (VEN 600A-301 FDA)	1994	2	3	venlafaxine	Yes	76	41 (13)	0.71	150-225	Flexible	179	HAMD 21	25.39	6	DSM-III-R	North America	Multi-center	Other/Unclear	Outpatients only	Yes	Unclear
				placebo	No	78	42 (12)	0.67	0-0	Flexible	0	HAMD 21	24.56								
SCT-MD-09	unpublished	2	2	escitalopram	Yes	16	35.9 (10.6)	*	10-20	Fixed	*	*	*	5	DSM-IV	North America	Single center	Other/Unclear	Outpatients only	Unclear	Unclear
				fluoxetine	No	14	41.4 (9.8)	*	20-40	Fixed	*	*	*								
SCT-MD-49 (NCT00668525)	unpublished	3	3	escitalopram	Yes	325	41.4 (12.3)	0.64	10-10	Fixed	10	HAMD 24	29.30	8	DSM-IV	North America	Multi-center	Other/Unclear	Other/Unclear	Yes	Unclear
				escitalopram	Yes	332	40.4 (11.9)	0.69	28-28	Fixed	28	HAMD 24	29.30								
				placebo	No	220	42.3 (12.7)	0.63	*	Fixed	*	HAMD 24	28.90								
Sechter 1999	1999	2	2	sertraline	Yes	118	43.4 (12.3)	*	50-150	Flexible	77	HAMD 17	24.50	8	DSM-III-R	Europe	Multi-center	Secondary /Tertiary care	Outpatients only	No	Unclear
				fluoxetine	No	120	42.5 (11.1)	*	20-60	Flexible	34	HAMD 17	24.90								
Sechter 2004	2004	2	2	milnacipran	Yes	149	44.8 (11.6)	*	100-100	Fixed	100	HAMD 17	23.70	6	DSM-IV	Europe	Multi-center	Secondary /Tertiary care	Outpatients only	Unclear	Unclear
				paroxetine	No	153	42.8 (11.2)	*	20-20	Fixed	20	HAMD 17	23.40								
SER 101 (FDA)	unpublished	4	5	sertraline	Yes	26	*	*	50-50	Fixed	50	HAMD 17	*	4	DSM-III	North America	Multi-center	Secondary /Tertiary care	Inpatients only	Yes	Unclear
				sertraline	Yes	24	*	*	100-100	Fixed	100	HAMD 17	*								
				sertraline	Yes	23	*	*	200-200	Fixed	200	HAMD 17	*								

				placebo	No	26	*	*	0-0	Fixed	0	HAMD 17	*								
SER 310 (FDA)	unpublished	4	4	sertraline	Yes	33	*	*	50-50	Fixed	50	*	*	4	DSM-III	Europe	Multi-center	Secondary /Tertiary care	Inpatients only	Yes	Yes
				sertraline	Yes	35	*	*	100-100	Fixed	100	*	*								
				sertraline	Yes	37	*	*	200-200	Fixed	200	*	*								
				placebo	No	34	*	*	0-0	Fixed	0	*	*								
SER 315 (FDA)	unpublished	3	3	sertraline	Yes	85	41 (*)	0.66	50-200	Flexible	105	HAMD 17	23.10	8	DSM-III	Europe	Multi-center	Other/Unclear	Outpatients only	Yes	Yes
				amitriptyline	No	77	44 (*)	0.71	50-200	Flexible	82	HAMD 17	23.50								
				placebo	No	80	43 (*)	0.78	0-0	Flexible	0	HAMD 17	22.20								
SER-CHN-1	unpublished	2	2	paroxetine	Yes	113	40.14 (13.2)	0.55	20-30	Flexible	*	HAMD unspecified	27.10	6	DSM-III-R	Asia	Multi-center	Other/Unclear	Other/Unclear	Unclear	Unclear
				amitriptyline	No	118	40.54 (13.45)	0.53	150-150	Fixed	150	HAMD unspecified	26.60								
Settle 1999 (WELL212)	1999	2	3	bupropion	Yes	150	37.2 (11.3)	*	100-300	Flexible	*	HAMD 21	24.40	8	DSM-III-R	North America	Multi-center	Other/Unclear	Outpatients only	Yes	No
				placebo	No	154	38.2 (11.4)	*	0-0	Flexible	0	HAMD 21	23.88								
Shaw 1986	1986	2	2	citalopram	Unclear	24	*	*	30-60	Flexible	46	HAMD 17	25.67	6	DSM-III	Europe	Multi-center	Secondary /Tertiary care	Both	Unclear	Yes
				amitriptyline	Unclear	20	*	*	112.5-225	Flexible	148	HAMD 17	26.48								
Sheehan 2009a	2009	3	3	fluoxetine	No	99	37.8 (11.1)	0.69	60-80	Flexible	61	HAMD 21	29.50	6	DSM-IV	North America	Multi-center	Secondary /Tertiary care	Inpatients only	Yes	Yes
				venlafaxine	Yes	95	41.7 (12.8)	0.48	225-375	Flexible	298	HAMD 21	29.90								
				placebo	No	95	39.9 (13)	0.64	0-0	Flexible	0	HAMD 21	29.40								
Sheehan 2009b (NCT00775203)	2009	2	2	trazodone	Yes	206	43.8 (12.8)	0.64	150-375	Flexible	310	HAMD 17	23.20	8	DSM-IV	North America	Multi-center	Other/Unclear	Other/Unclear	No	No
				placebo	No	206	44 (13.5)	0.64	0-0	Flexible	0	HAMD 17	22.40								
Shelton 2006 (NCT00179283)	2006	2	2	venlafaxine	Unclear	78	37.2 (11.6)	*	75-225	Flexible	*	HAMD 17	22.40	8	DSM-IV	North America	Multi-center	Secondary /Tertiary care	Outpatients only	Unclear	No
				sertraline	Yes	82	41.2 (12)	*	50-150	Flexible	*	HAMD 17	22.10								
Shipley 1981	1981	2	2	amitriptyline	No	53	39 (13.1)	*	200-200	Fixed	200	HAMD 17	38.5	4	RDC	North America	Single center	Secondary /Tertiary care	Inpatients only	Yes	Unclear
				placebo	No	23	40.3 (13.9)	*	0-0	Fixed	0	HAMD 17	44.2								
Shu 2014 (CL3-20098-052, ChiCTR-TRC-11001668)	2014	2	2	agomelatine	Yes	314	39.2 (12.8)	0.68	25-50	Flexible	*	HAMD 17	26.80	8	DSM-IV	Asia	Multi-center	Primary care	Outpatients only	No	No
				fluoxetine	No	314	38.9 (12.6)	0.71	20-40	Flexible	*	HAMD 17	26.80								

Silverstone 1999	1999	3	3	fluoxetine	No	121	43.2 (10.9)	*	20-60	Flexible	40	HAMD 21	27.20	12	DSM-IV	*	Multi-center	Other/Unclear	Outpatients only	Yes	Unclear
				venlafaxine	Yes	128	41.1 (12)	*	75-225	Flexible	141	HAMD 21	27.20								
				placebo	No	119	41.6 (10.8)	*	0-0	Flexible	0	HAMD 21	27.20								
Sir 2005	2005	2	2	venlafaxine	No	84	36.8 (12.4)	*	75-225	Flexible	161	HAMD 17	23.50	8	DSM-IV	Cross-Continental	Multi-center	Secondary /Tertiary care	Outpatients only	Unclear	Unclear
				sertraline	Yes	79	37.3 (13.5)	*	50-150	Flexible	105	HAMD 17	23.40								
Smeraldi 1998	1998	3	3	venlafaxine	Unclear	55	71 (6)	*	75-150	Flexible	*	*	*	6	DSM-III-R	Europe	Multi-center	Other/Unclear	Both	Yes	Unclear
				clomipramine	Unclear	58	71 (6)	*	50-100	Flexible	*	*	*								
				trazodone	Unclear	57	71 (6)	*	150-300	Flexible	*	*	*								
Smith 1990 (MIR 003-024 FDA)	1990	3	3	mirtazapine	Yes	50	*	*	10-35	Flexible	18	HAMD 21	27.50	6	DSM-III	North America	Unclear	Secondary /Tertiary care	Outpatients only	Yes	Unclear
				amitriptyline	No	50	*	*	80-280	Flexible	111	HAMD 21	27.60								
				placebo	No	50	*	*	0-0	Flexible	0	HAMD 21	27.70								
Sramek 1995	1995	2	3	fluoxetine	No	72	33.9 (*)	0.60	20-20	Fixed	20	HAMD 24	28.20	9	DSM-III-R	North America	Single center	Other/Unclear	Other/Unclear	Yes	Unclear
				placebo	No	72	33.9 (*)	0.60	0-0	Fixed	0	HAMD 24	27.50								
Stahl 2000	2000	3	3	citalopram	Yes	107	39.1 (*)	*	20-60	Flexible	57	HAMD 21	26.50	8	DSM-IV	North America	Multi-center	Other/Unclear	Outpatients only	Yes	Unclear
				sertraline	Unclear	108	39.5 (*)	*	50-150	Flexible	143	HAMD 21	26.60								
				placebo	No	108	37.3 (*)	*	0-0	Flexible	0	HAMD 21	26.40								
Stahl 2010 (CAGO178A2302)	2010	3	3	agomelatine	Yes	168	43.2 (11.82)	0.68	25-25	Fixed	25	HAMD 17	26.80	8	DSM-IV	North America	Multi-center	Other/Unclear	Outpatients only	Unclear	No
				agomelatine	Yes	169	43.8 (11.96)	0.64	50-50	Fixed	50	HAMD17	26.80								
				placebo	No	166	43 (13.11)	0.65	0-0	Fixed	0	HAMD 17	26.40								
Staner 1995 (063)	1995	2	2	paroxetine	Yes	21	41.7 (10.8)	*	30-30	Fixed	30	HAMD 21	26.00	5	RDC	Europe	Single center	Secondary /Tertiary care	Inpatients only	Yes	Yes
				amitriptyline	No	19	42.5 (11.7)	*	150-150	Fixed	150	HAMD 21	24.00								
Stark 1985 (Study 27 - FDA)	1985	2	3	fluoxetine	Yes	185	40 (*)	0.68	20-80	Flexible	69	HAMD 21	27.50	6	DSM-III	North America	Multi-center	Other/Unclear	Outpatients only	Yes	Unclear
				placebo	No	169	41 (*)	0.67	0-0	Flexible	0	HAMD 21	28.10								

Stratas 1984	1984	2	3	amitriptyline	No	12	*	*	50-300	Flexible	179	HAMD 21	*	6	RDC	North America	Single center	Secondary /Tertiary care	Outpatients only	Yes	Unclear
				placebo	No	10	*	*	0-0	Flexible	0	HAMD 21	*								
Studie009 (CTN009-FCE20124)	unpublished	2	2	reboxetine	Yes	26	45.81 (11.3)	0.65	8-8	Fixed	8	HAMD 17	25.33	4	DSM-III	Europe	Multi-center	Secondary /Tertiary care	Inpatients only	Unclear	Yes
				placebo	No	24	47.04 (13.5)	0.67	0-0	Fixed	0	HAMD 17	25.27								
Studie032 (M2020/0032)	unpublished	2	2	reboxetine	Yes	43	40.65 (14.6)	0.63	8-10	Flexible	8	HAMD 21	27.23	8	DSM-IV	Asia	Multi-center	Secondary /Tertiary care	Both	Yes	Yes
				fluoxetine	No	42	35.98 (12.7)	0.62	20-40	Flexible	27	HAMD 21	28.33								
Study 015	unpublished	2	3	reboxetine	Yes	112	45.9 (12.7)	0.63	8-10	Flexible	9	HAMD 21	27.50	6	DSM-III-R	Cross-Continental	Multi-center	Secondary /Tertiary care	Both	Unclear	Yes
				placebo	No	112	43.3 (11.7)	0.48	0-0	Flexible	0	HAMD 21	27.10								
Study 032a (CTN032-FCE20124)	unpublished	2	2	reboxetine	Yes	24	79.75 (7.11)	0.79	4-6	Flexible	*	HAMD 21	23.20	8	DSM-III-R	Europe	Multi-center	Secondary /Tertiary care	Inpatients only	Unclear	Yes
				placebo	No	26	80.15 (4.51)	0.77	0-0	Flexible	0	HAMD 21	23.10								
Study 043	2006	2	2	reboxetine	Yes	183	42.8 (13.3)	0.69	8-10	Flexible	9	HAMD 21	27.40	24	DSM-IV	Europe	Multi-center	Other/Unclear	Outpatients only	Unclear	Yes
				citalopram	No	176	41.5 (12)	0.60	20-40	Flexible	28	HAMD 21	27.40								
Study 045	unpublished	3	4	reboxetine	Yes	87	40.8 (10)	0.63	4-4	Fixed	4	HAMD 21	26.20	6	DSM-IV	Cross-Continental	Multi-center	Both	Both	No	Yes
				reboxetine	Yes	89	41.6 (10.6)	0.63	8-8	Fixed	8	HAMD 21	26.40								
				placebo	No	87	40.5 (11.2)	0.70	0-0	Fixed	0	HAMD 21	26.40								
Study 049	unpublished	2	2	reboxetine	Yes	107	39.9 (11.6)	0.55	8-10	Flexible	10	HAMD 21	25.10	6	DSM-IV	North America	Multi-center	Other/Unclear	Outpatients only	Yes	No
				placebo	No	105	39.7 (11.1)	0.58	0-0	Flexible	0	HAMD 21	25.30								
Study 19 (FDA, Fabre 1985)	unpublished	2	2	fluoxetine	Yes	22	34.1 (*)	0.75	40-80	Flexible	*	HAMD 21	28.90	5	RDC	*	Single center	Other/Unclear	Outpatients only	Yes	Unclear
				placebo	No	25	32 (*)	0.48	0-0	Flexible	0	HAMD 21	27.40								
Study 205 (FDA, WELL 205)	unpublished	4	5	bupropion	Yes	120	39.6 (10.4)	0.57	200-200	Fixed	200	HAMD 17	23.20	8	Other operationalized	North America	Multi-center	Other/Unclear	Outpatients only	Yes	Unclear
				bupropion	Yes	120	39.9 (11.4)	0.62	300-300	Fixed	300	HAMD 17	23.60								
				bupropion	Yes	119	38.8 (11.7)	0.58	400-400	Fixed	400	HAMD 17	24.20								
				placebo	No	124	40.7 (11.6)	0.68	0-0	Fixed	0	HAMD 17	23.40								
Study 25 (FDA, Rickels 1986)	unpublished	2	2	fluoxetine	Yes	18	49.4 (*)	0.72	*-80	Other/Unclear	*	HAMD 21	25.20	5	RDC	*	Unclear	Other/Unclear	Outpatients only	Yes	Unclear

				placebo	No	24	45.7 (*)	0.83	0-0	Other/Unclear	0	HAMD 21	25.80								
Study 62a (FDA) - MILD depression (Dunlop 1990)	unpublished	4	4	fluoxetine	Yes	104	38.31 (*)	0.59	60-60	Fixed	60	HAMD 21	17.20	6	DSM-III	*	Multi-center	Other/Unclear	Outpatients only	Yes	Unclear
				fluoxetine	Yes	105	39.8 (*)	0.56	40-40	Fixed	40	HAMD 21	16.83								
				fluoxetine	Yes	107	39.97 (*)	0.62	20-20	Fixed	20	HAMD 21	16.99								
				placebo	No	56	38.96 (*)	0.50	0-0	Fixed	0	HAMD 21	17.41								
Study 62b (FDA) - MODERATE depression	unpublished	4	4	fluoxetine	Yes	105	39.93 (*)	0.58	60-60	Fixed	60	HAMD 21	24.20	6	DSM-III	*	Multi-center	Other/Unclear	Outpatients only	Yes	Unclear
				fluoxetine	Yes	103	41.15 (*)	0.56	40-40	Fixed	40	HAMD 21	24.19								
				fluoxetine	Yes	100	39.4 (*)	0.60	20-20	Fixed	20	HAMD 21	24.72								
				placebo	No	48	38.56 (*)	0.50	0-0	Fixed	0	HAMD 21	24.25								
Study 89306 (FDA)	unpublished	3	3	citalopram	Yes	88	*	*	20-20	Fixed	20	MADRS	32.70	6	DSM-III-R	*	Multi-center	Both	Both	Yes	Yes
				citalopram	Yes	97	*	*	40-40	Fixed	40	MADRS	31.30								
				placebo	No	89	*	*	0-0	Fixed	0	MADRS	33.14								
Study F1J-MC-HMAQ - Study Group B	unpublished	3	3	duloxetine	Yes	82	39.86 (9.8)	*	40-120	Flexible	*	HAMD 17	17.88	10	DSM-IV	North America	Multi-center	Other/Unclear	Outpatients only	Yes	Unclear
				placebo	No	75	41.39 (11.8)	*	0-0	Flexible	0	HAMD 17	18.32								
				fluoxetine	No	37	39.65 (12.7)	*	20-20	Fixed	20	HAMD 17	20.03								
Szegedi 2006	2006	2	2	mirtazapine	Yes	130	*	*	45-45	Fixed	45	HAMD 17	24.60	6	DSM-IV	Europe	Multi-center	Other/Unclear	Outpatients only	No	Unclear
				venlafaxine	No	128	*	*	225-225	Fixed	225	HAMD 17	24.90								
Thase 1997 (VEN XR 209 FDA)	1997	2	2	venlafaxine	Yes	95	40 (11)	0.63	75-225	Flexible	177	HAMD 21	24.53	8	DSM-IV	North America	Multi-center	Other/Unclear	Outpatients only	Yes	Yes
				placebo	No	102	42 (12)	0.60	0-0	Flexible	0	HAMD 21	23.63								
Thase 2006 (WELL 100368)	2006	2	2	bupropion	Yes	171	37.1 (12.3)	*	150-450	Flexible	300	HAMD 17	24.90	12	DSM-IV	North America	Multi-center	Secondary/Tertiary care	Outpatients only	Unclear	Unclear
				venlafaxine	No	177	37.4 (11.6)	*	75-225	Flexible	150	HAMD 17	24.10								
Thomson 1982	1982	2	4	amitriptyline	No	31	*	*	150-150	Fixed	150	HAMD unspecified	17.35	12	RDC	Europe	Multi-center	Primary care	Outpatients only	Yes	Yes
				placebo	No	28	*	*	0-0	Fixed	0	HAMD unspecified	19.43								
Tignol 1993 (29060/087)	1993	2	2	paroxetine	Yes	89	43 (11.6)	*	20-20	Fixed	20	MADRS	30.74	6	DSM-III-R	Europe	Multi-center	Secondary/Tertiary care	Inpatients only	Yes	Unclear
				fluoxetine	No	87	45 (13.2)	*	20-20	Fixed	20	MADRS	31.60								
Timmerman 1993 (Study 89422 - FDA	1993	2	2	fluvoxamine	No	109	*	*	100-200	Flexible	*	HAMD 17	24.50	6	DSM-III-R	Europe	Multi-center	Secondary/Tertiary care	Outpatients only	Unclear	Yes

and Haffmans 1996)				citalopram	Yes	108	*	*	20-40	Flexible	*	HAMD 17	24.70								
Tollefson 1995	1995	2	2	fluoxetine	Yes	335	67.4 (5.4)	0.54	*-20	Flexible	*	HAMD 17	22.20	6	DSM-III-R	North America	Multi-center	Other/Unclear	Outpatients only	Yes	Unclear
				placebo	No	336	68.1 (5.9)	0.55	0-0	Flexible	0	HAMD 17	22.10								
Tomarken 2004	2004	2	2	bupropion	Yes	10	39.4 (9.8)	*	200-300	Flexible	*	HAMD 17	21.70	6	DSM-IV	North America	Single center	Secondary /Tertiary care	Outpatients only	No	Unclear
				placebo	No	9	37.5 (7.8)	*	0-0	Flexible	0	HAMD 17	21.70								
Tourian 2009 (NCT00384033)	2009	4	4	desvenlafaxine	Yes	155	41 (13)		50-50	Fixed	50	HAMD 17	23	8	DSM-IV	North America	Multi-center	Other/Unclear	Outpatients only	No	Unclear
				desvenlafaxine	Yes	160	39 (12)		100-100	Fixed	100	HAMD 17	23								
				duloxetine	No	159	39 (12)		60-60	Fixed	60	HAMD 17	23								
				placebo	No	164	39 (13)		0-0	Fixed	0	HAMD 17	24								
Trivedi 2004 (29060/810)	2004	2	3	placebo	No	149	38.4 (11.7)	*	0-0	Fixed	0	HAMD 17	23.80	8	DSM-IV	North America	Multi-center	Other/Unclear	Other/Unclear	Yes	Unclear
				paroxetine	Yes	154	39.4 (10.8)	*	25-25	Fixed	25	HAMD 17	23.50								
Tsutsui 2000	2000	2	2	paroxetine	Yes	116	39.1 (13.9)	*	10-40	Flexible	32	HAMD 17	23.60	6	DSM-IV	Asia	Multi-center	Both	Both	No	Yes
				trazodone	No	109	38.5 (12.7)	*	75-200	Flexible	154	HAMD 17	24.40								
Tylee 1997	1997	2	2	fluoxetine	No	170	45.5 (14.3)	0.75	20-20	Fixed	20	HAMD 17	22.50	12	DSM-IV	Europe	Multi-center	Primary care	Outpatients only	Unclear	Unclear
				venlafaxine	Yes	171	43.5 (14)	0.68	75-75	Fixed	75	HAMD 17	22.40								
Tzanakaki 2000	2000	2	2	fluoxetine	No	54	49 (10)	*	20-60	Flexible	*	HAMD 21	27.10	6	DSM-IV	Europe	Multi-center	Secondary /Tertiary care	Both	Yes	Unclear
				venlafaxine	Yes	55	47 (11)	*	75-225	Flexible	*	HAMD 21	27.80								
Upward 1988	1988	2	2	fluoxetine	Yes	13	40 (10.6)	0.55	60-80	Flexible	75	*	*	4	*	Europe	Unclear	Other/Unclear	Outpatients only	Yes	Yes
				amitriptyline	No	14	45 (13.2)	0.58	150-200	Flexible	183	*	*								
Van de Merwe 1984	1984	3	3	amitriptyline	No	5	*	*	100-150	Flexible	*	HAMD 17	*	4	RDC	Europe	Single center	Secondary /Tertiary care	Outpatients only	Yes	Yes
				placebo	No	3	*	*	0-0	Flexible	0	HAMD 17	*								
				trazodone	Yes	6	*	*	50-300	Flexible	*	HAMD 17	*								
VanMoffaert 1995a	1995	2	2	sertraline	Yes	83	46.1 (*)	*	50-100	Flexible	*	HAMD 17	24.50	8	DSM-III-R	Europe	Multi-center	Secondary /Tertiary care	Other/Unclear	Yes	Yes
				fluoxetine	No	82	48.4 (*)	*	20-40	Flexible	*	HAMD 17	23.20								
VanMoffaert 1995b	1995	2	2	mirtazapine	Yes	100	46.1 (10.8)	0.69	24-72	Flexible	*	HAMD 17	29.2	6	DSM-III	Europe	Multi-center	Secondary /Tertiary care	Inpatients only	Unclear	Yes

				trazodone	No	100	46.3 (12.6)	0.71	150-450	Flexible	*	HAMD 17	27.5								
Vartiainen 1994 (MIR 84023 FDA)	1994	2	2	mirtazapine	Yes	60	46 (*)	0.58	15-50	Flexible	33	HAMD 21	25.60	6	*	Europe	Multi-center	Secondary /Tertiary care	Inpatients only	Yes	Yes
				placebo	No	57	46 (*)	0.48	0-0	Flexible	0	HAMD 21	26.00								
VEN 600A-303 (FDA)	unpublished	2	3	venlafaxine	Yes	83	39 (*)	0.70	150-225	Flexible	185	HAMD 21	23.63	6	DSM-III-R	North America	Single center	Other/Unclear	Outpatients only	Yes	Yes
				placebo	No	82	38 (*)	0.68	0-0	Flexible	0	HAMD 21	24.59								
VEN 600A-313 (FDA)	unpublished	3	4	venlafaxine	Yes	76	38 (*)	0.70	75-75	Fixed	75	HAMD 21	25.57	6	DSM-III-R	North America	Unclear	Other/Unclear	Outpatients only	Yes	Yes
				venlafaxine	Yes	82	39 (*)	0.61	200-200	Fixed	200	HAMD 21	25.57								
				placebo	No	79	38 (*)	0.69	0-0	Fixed	0	HAMD 21	25.39								
VEN XR 367 (FDA)	unpublished	4	4	venlafaxine	Yes	83	*	0.70	75-75	Fixed	75	HAMD 21	26.49	8	DSM-III-R	Europe	Multi-center	Other/Unclear	Outpatients only	Yes	Yes
				venlafaxine	Yes	82	*	0.61	150-150	Fixed	150	HAMD 21	27.11								
				paroxetine	No	81	*	0.54	20-20	Fixed	20	HAMD 21	26.13								
				placebo	No	83	*	0.67	0-0	Fixed	0	HAMD 21	26.60								
Ventura 2007 (SCT-MD-18)	2007	2	2	escitalopram	Yes	107	40.6 (13.7)	*	10-10	Fixed	10	HAMD 24	26.80	8	DSM-IV	North America	Multi-center	Other/Unclear	Outpatients only	Unclear	Unclear
				sertraline	No	108	38.1 (11.5)	*	50-200	Flexible	*	HAMD 24	26.80								
Versiani 1999	1999	2	2	fluoxetine	Yes	77	41.3 (*)	0.76	20-20	Fixed	20	HAMD 21	28.40	8	DSM-IV	South America	Multi-center	Other/Unclear	Other/Unclear	Yes	No
				amitriptyline	No	80	41.3 (*)	0.76	50-250	Flexible	138	HAMD 21	27.80								
Versiani 2000 (Study 091)	2000	2	2	reboxetine	Yes	28	41.4 (*)	0.46	10-10	Fixed	10	HAMD 21	35.70	6	DSM-III-R	Cross-Continental	Multi-center	Secondary /Tertiary care	Inpatients only	Yes	No
				placebo	No	28	39.9 (*)	0.50	0-0	Fixed	0	HAMD 21	35.10								
Versiani 2005	2005	2	2	mirtazapine	Yes	145	43 (*)	*	15-60	Flexible	*	HAMD 17	29.00	8	DSM-IV	Cross-Continental	Multi-center	Other/Unclear	Other/Unclear	Yes	No
				fluoxetine	No	149	47 (*)	*	20-40	Flexible	*	HAMD 17	28.00								
Wade 2002 (ESC Study 99001 - FDA)	2002	2	2	placebo	No	189	40 (12)	0.78	0-0	Fixed	0	MADRS	28.70	8	DSM-IV	Cross-Continental	Multi-center	Primary care	Outpatients only	Yes	Unclear
				escitalopram	Yes	191	41 (11)	0.74	10-10	Fixed	10	MADRS	29.20								
Wade 2003 (E-1721)	2003	2	2	mirtazapine	Yes	99	40 (14.3)	*	15-45	Flexible	35	HAMD 17	23.80	12	DSM-IV	Europe	Multi-center	Primary care	Outpatients only	No	No
				paroxetine	No	98	40 (11.7)	*	10-30	Flexible	24	HAMD 17	24.40								
Wade 2007 (Study 10990)	2007	2	2	escitalopram	Yes	144	43.3 (11.6)	*	20-20	Fixed	20	HAMD 17	22.70	8	DSM-IV	Cross-Continental	Multi-center	Primary care	Outpatients only	No	Unclear
				duloxetine	No	151	44.5 (11)	*	60-60	Fixed	60	HAMD 17	22.70								
Walczak 1996	1996	4	5	fluvoxamine	Yes	101	*	*	50-50	Fixed	50	HAMD 21	*	6	DSM-III-R	North America	Multi-center	Secondary /Tertiary care	Outpatients only	Yes	No

				fluvoxamine	Yes	100	*	*	100-100	Fixed	100	HAMD 21	*								
				fluvoxamine	Yes	99	*	*	150-150	Fixed	150	HAMD 21	*								
				placebo	No	200	*	*	0-0	Fixed	0	HAMD 21	*								
Wang 2014 (EUCTR2005-005052-40, NCT00351169, D1448C00004)	2014	2	3	escitalopram	No	157	40.3 (12.5)	0.75	10-20	Flexible	11	HAMD unspecified	27.20	8	DSM-IV	Cross-Continental	Multi-center	Other/Unclear	Other/Unclear	Unclear	Yes
				placebo	No	157	39.7 (11.1)	0.67	0-0	Flexible	0	HAMD unspecified	26.60								
Wang 2015 (NCT01571453, KCT0000432, 13926A)	2015	2	2	vortioxetine	Yes	213	39.6 (12.4)	0.58	10-10	Fixed	10	MADRS	32.30	8	DSM-IV	Asia	Multi-center	Secondary /Tertiary care	Both	Unclear	Yes
				venlafaxine	No	230	40.7 (12.3)	0.62	150-150	Fixed	150	MADRS	32.30								
Weisler 1994	1994	2	2	bupropion	Yes	63	40.2 (*)	*	225-450	Flexible	345	HAMD 21	25.80	6	DSM-III-R	North America	Multi-center	Secondary /Tertiary care	Outpatients only	Yes	Unclear
				trazodone	No	61	40.8 (*)	*	150-400	Flexible	207	HAMD 21	24.90								
WELL 029	unpublished	2	3	bupropion	Yes	25	37.8 (*)	0.44	300-300	Fixed	300	HAMD 21	26.60	4	*	North America	Multi-center	Secondary /Tertiary care	Inpatients only	Unclear	Unclear
				placebo	No	24	41.5 (*)	0.50	0-0	Fixed	0	HAMD 21	26.20								
WELL AK140016	unpublished	2	2	bupropion	Yes	69	37.6 (10.6)	*	300-300	Fixed	300	HAMD 17	21.90	8	DSM-IV	North America	Multi-center	Other/Unclear	Other/Unclear	Unclear	Unclear
				paroxetine	No	71	37 (10.8)	*	20-20	Fixed	20	HAMD 17	22.30								
WELL AK1A4006	unpublished	3	3	fluoxetine	No	155	38.6 (12)	*	20-60	Flexible	*	HAMD 21	25.30	8	DSM-IV	North America	Multi-center	Other/Unclear	Outpatients only	Unclear	Unclear
				bupropion	Yes	158	37.1 (10.7)	*	150-400	Flexible	*	HAMD 21	25.60								
				placebo	No	154	37.2 (11.2)	*	0-0	Flexible	0	HAMD 21	25.20								
Wellbutrin 06	unpublished	2	2	bupropion	Yes	56	42 (*)	0.19	300-600	Flexible	*	HAMD 21	28.50	4	*	North America	Multi-center	Secondary /Tertiary care	Inpatients only	Unclear	Unclear
				placebo	No	29	40.7 (*)	0.26	0-0	Flexible	0	HAMD 21	29.30								
Wellbutrin 25	unpublished	3	3	bupropion	Yes	45	52.4 (*)	0.49	300-300	Fixed	300	HAMD 21	32.40	4	*	North America	Multi-center	Secondary /Tertiary care	Inpatients only	Unclear	Unclear
				bupropion	Yes	40	47.5 (*)	0.50	450-450	Fixed	450	HAMD 21	34.80								
				placebo	No	43	51.9 (*)	0.51	0-0	Fixed	0	HAMD 21	33.60								
Wheatley 1998	1998	2	2	mirtazapine	Yes	66	47.2 (15.3)	*	15-60	Flexible	40	HAMD 17	26.00	6	DSM-III	Europe	Multi-center	Other/Unclear	Both	Yes	No
				fluoxetine	No	67	47.5 (14.8)	*	20-40	Flexible	24	HAMD 17	26.10								
Wilcox 1994	1994	2	3	amitriptyline	No	50	40 (*)	*	60-300	Flexible	122	HAMD 17	23.8	6	DSM-III	North America	Single center	Secondary /Tertiary care	Outpatients only	Yes	Yes

				placebo	No	49	40 (*)	*	0-0	Flexible	0	HAMD 17	23.5								
Winokur 2003	2003	2	2	mirtazapine	Yes	8	40.9 (*)	*	15-45	Flexible	*	HAMD 21	25.60	8	DSM-IV	North America	Unclear	Other/Unc lear	Other/Uncle ar	No	No
				fluoxetine	No	12	43.5 (*)	*	20-40	Flexible	*	HAMD 21	26.70								
Yang 2003	2003	2	2	milnacipran	Yes	27	43.5 (10.4)	*	50-100	Flexible	*	HAMD 17	25.20	6	DSM-IV	Asia	Multi-center	Secondary /Tertiary care	Outpatients only	Unclear	Unclear
				sertraline	No	26	42 (8.9)	*	50-100	Flexible	*	HAMD 17	23.20								
Yevtushenko 2007	2007	2	3	escitalopram	Yes	109	35.2 (6.5)	0.61	10-10	Fixed	10	MADRS	25.67	6	DSM-IV	Europe	Multi-center	Secondary /Tertiary care	Outpatients only	Unclear	Yes
				citalopram	No	110	35.1 (6.5)	0.56	20-20	Fixed	20	MADRS	35.70								
Young 1987	1987	2	2	fluoxetine	Unclear	32	46.1 (*)	0.68	40-80	Flexible	73	HAMD unspecified	44.00	6	RDC	Europe	Unclear	Secondary /Tertiary care	Outpatients only	Unclear	Yes
				amitriptyline	Unclear	32	46.6 (*)	0.68	50-150	Flexible	122	HAMD unspecified	48.10								
Zajecka 2010 (CAGO178A2301, NCT00411099)	2010	3	3	agomelatine	Yes	170	44.2 (12.3)	0.69	25-25	Fixed	25	HAMD 17	26.70	8	DSM-IV	North America	Multi-center	Primary care	Outpatients only	No	No
				agomelatine	Yes	168	44.2 (12.3)	0.63	50-50	Fixed	50	HAMD 17	27.10								
				placebo	No	173	43.1 (12)	0.68	0-0	Fixed	0	HAMD 17	27.10								
Zhang 2014	2013	2	2	trazodone	No	192	39.5 (12.7)	0.62	150-450	Flexible	273	HAMD 17	21.60	6	DSM-IV	Asia	Multi-center	Secondary /Tertiary care	Both	Yes	Unclear
				placebo	No	190	38.3 (12.2)	0.61	0-0	Flexible	0	HAMD 17	21.90								
Zivkov 1995 (Organon 85146, FDA85146)	1997	2	2	mirtazapine	Yes	103	48.5 (10.6)	*	*	Other/U nclear	*	HAMD 17	27.30	6	DSM-III	North America	Multi-center	Other/Unc lear	Other/Uncle ar	Unclear	Unclear
				amitriptyline	No	104	47.7 (12.4)	*	*	Other/U nclear	*	HAMD 17	26.20								
845	unpublis hed	2	3	bupropion	Yes	56	36.4 (*)	0.67	150-450	Flexible	*	HAMD 21	24.60	6	*	North America	Multi-center	Other/Unc lear	Outpatients only	Yes	Unclear
				placebo	No	52	37.4 (*)	0.70	0-0	Flexible	0	HAMD 21	26.80								
003-008 (FDA)	unpublis hed	3	5	mirtazapine	Yes	30	38 (*)	0.63	15-15	Fixed	15	HAMD 21	26.00	6	DSM-III	North America	Single center	Other/Unc lear	Outpatients only	Yes	Unclear
				mirtazapine	Yes	30	40 (*)	0.71	30-30	Fixed	30	HAMD 21	25.50								
				placebo	No	30	39 (*)	0.61	0-0	Fixed	0	HAMD 21	25.80								
003-042	unpublis hed	4	4	mirtazapine	Yes	70	41 (10.8)	0.71	15-15	Fixed	15	HAMD 21	25.30	8	DSM-IV	North America	Multi-center	Other/Unc lear	Outpatients only	Yes	Unclear
				mirtazapine	Yes	70	39.8 (10.2)	0.56	30-30	Fixed	30	HAMD 21	24.70								
				mirtazapine	Yes	70	42.2 (12.9)	0.60	45-45	Fixed	45	HAMD 21	25.00								
				placebo	No	70	40.8 (10.8)	0.66	0-0	Fixed	0	HAMD 21	25.90								

003-048	unpublished	3	3	mirtazapine	Yes	210	41.4 (12.1)	0.60	45-45	Fixed	45	HAMD 17	22.30	8	DSM-IV	North America	Multi-center	Other/Unclear	Other/Unclear	Yes	Unclear
				fluoxetine	No	210	40.8 (11.6)	0.58	60-60	Fixed	60	HAMD 17	22.80								
				placebo	No	72	40.2 (11.5)	0.61	0-0	Fixed	0	HAMD 17	23.00								
030A2-0004/030A2-0005 (FDA)	unpublished	2	3	nefazodone	Yes	76	44.9 (*)	0.53	50-250	Flexible	175	*	*	6	DSM-III	North America	Multi-center	Other/Unclear	Other/Unclear	Unclear	Yes
				placebo	No	74	41.8 (*)	0.51	0-0	Flexible	0	*	*								
03A0A-004A (FDA)	unpublished	3	3	nefazodone	Yes	80	40.1 (*)	0.54	150-300	Flexible	276	HAMD 17	23.20	6	DSM-III	North America	Multi-center	Other/Unclear	Outpatients only	Unclear	Yes
				nefazodone	Yes	80	39.6 (*)	0.63	300-600	Flexible	514	HAMD 17	23.60								
				placebo	No	80	36.8 (*)	0.64	0-0	Flexible	0	HAMD 17	23.50								
0600A1-300	unpublished	3	*	venlafaxine	Yes	58	*	*	*	Other/Unclear	*	*	*	*	*	*	Unclear	Other/Unclear	Other/Unclear	Unclear	Unclear
				amitriptyline	No	52	*	*	*	Other/Unclear	*	*	*								
				placebo	No	*	*	*	0-0	Other/Unclear	0	*	*								
0600A1-372	unpublished	2	2	venlafaxine	Yes	156	*	*	*	Other/Unclear	*	*	*	6	DSM-IV	North America	Unclear	Other/Unclear	Outpatients only	Unclear	Unclear
				fluoxetine	No	152	*	*	*	Other/Unclear	*	*	*								
0600A-326	unpublished	2	2	venlafaxine	Unclear	60	*	*	*	Other/Unclear	*	*	*	6	DSM-III-R	*	*	Other/Unclear	Other/Unclear	Unclear	Unclear
				clomipramine	Unclear	61	*	*	*	Other/Unclear	*	*	*								
0600A-332	unpublished	2	2	venlafaxine	Yes	27	*	*	*	Other/Unclear	*	*	*	6	DSM-III-R	North America	Unclear	Other/Unclear	Outpatients only	Unclear	Unclear
				fluoxetine	No	24	*	*	*	Other/Unclear	*	*	*								
0600A-347	unpublished	2	2	venlafaxine	Yes	63	*	*	*	Other/Unclear	*	*	*	*	*	*	Unclear	Other/Unclear	Other/Unclear	Unclear	Unclear
				fluvoxamine	No	29	*	*	*	Other/Unclear	*	*	*								
0600A-349	unpublished	2	2	paroxetine	No	85	*	*	*	Other/Unclear	*	*	*	8	DSM-III-R	*	Unclear	Other/Unclear	Other/Unclear	Unclear	Unclear
				venlafaxine	Yes	82	*	*	*	Other/Unclear	*	*	*								
0600A-626	unpublished	2	2	venlafaxine	Yes	79	*	*	*	Other/Unclear	*	*	*	8	DSM-IV	North America	Unclear	Other/Unclear	Outpatients only	Unclear	Unclear
				fluoxetine	No	77	*	*	*	Other/Unclear	*	*	*								
0600A-654	unpublished	2	2	venlafaxine	Yes	131	*	*	*-150	Flexible	*	MADRS	*	12	DSM-IV	*	Unclear	Other/Unclear	Other/Unclear	Unclear	Unclear
				fluoxetine	No	135	*	*	*-40	Flexible	*	MADRS	*								
0600B1-367	unpublished	2	2	paroxetine	No	81	*	*	*	Other/Unclear	*	HAMD unspecified	*	8	DSM-III-R	*	Unclear	Other/Unclear	Other/Unclear	Unclear	Unclear
				venlafaxine	Yes	165	*	*	*	Other/Unclear	*	HAMD unspecified	*								

0600B1-384	unpublished	2	2	venlafaxine	Yes	176	*	*	*	Other/Unclear	*	MADRS	*	6	DSM-III-R	*	Unclear	Other/Unclear	Other/Unclear	Unclear	Unclear
				placebo	No	183	*	*	0-0	Other/Unclear	0	MADRS	*								
0600B1-402	unpublished	2	2	venlafaxine	Yes	295	*	*	*	Other/Unclear	*	*	*	8	DSM-IV	North America	Unclear	Other/Unclear	Outpatients only	Unclear	Unclear
				sertraline	No	293	*	*	*	Other/Unclear	*	*	*								
244 (EMD 68 843-009)	unpublished	2	3	fluoxetine	Unclear	89	*	*	20-20	Flexible	20	HAMD unspecified	24.40	8	DSM-IV	North America	Multi-center	Other/Unclear	Other/Unclear	Yes	Unclear
				placebo	No	95	*	*	0-0	Flexible	0	HAMD unspecified	24.00								
245 (EMD 68 843-010)	unpublished	3	5	vilazodone	Yes	104	*	*	10-20	Flexible	*	HAMD 17	23.80	8	DSM-IV	North America	Multi-center	Other/Unclear	Outpatients only	Yes	Unclear
				fluoxetine	No	92	*	*	20-20	Fixed	20	HAMD 17	23.50								
				placebo	No	99	*	*	0-0	Fixed	0	HAMD 17	23.40								
246 (SB 659746-003)	unpublished	4	4	vilazodone	Yes	120	*	*	10-10	Fixed	10	HAMD 17	23.80	8	DSM-IV	North America	Multi-center	Other/Unclear	Outpatients only	Yes	Unclear
				vilazodone	Yes	123	*	*	20-20	Fixed	20	HAMD 17	23.70								
				citalopram	No	117	*	*	20-20	Fixed	20	HAMD 17	23.10								
				placebo	No	129	*	*	0-0	Fixed	0	HAMD 17	23.30								
247 (SB 659746-014)	unpublished	2	2	vilazodone	Yes	109	*	*	5-20	Flexible	*	HAMD 17	23.30	8	DSM-IV	North America	Multi-center	Other/Unclear	Outpatients only	Yes	Unclear
				placebo	No	111	*	*	0-0	Flexible	0	HAMD 17	23.50								
248 (SB 659746-002)	unpublished	3	4	vilazodone	Yes	133	*	*	10-10	Fixed	10	HAMD 17	24.50	8	DSM-IV	North America	Multi-center	Other/Unclear	Outpatients only	Yes	Unclear
				vilazodone	Yes	132	*	*	20-20	Fixed	20	HAMD 17	24.30								
				placebo	No	128	*	*	0-0	Fixed	0	HAMD 17	23.70								
29060 07 001	unpublished	3	3	paroxetine	Yes	13	40 (*)	0.69	10-60	Flexible	*	HAMD 21	30.50	6	DSM-III	North America	Multi-center	Secondary /Tertiary care	Inpatients only	Yes	Unclear
				placebo	No	12	45.1 (*)	0.42	0-0	Flexible	0	HAMD 21	28.30								
				amitriptyline	No	13	44.6 (*)	0.62	*	Flexible	*	HAMD 21	30.40								
29060/299	unpublished	2	2	paroxetine	Yes	109	40.6 (12.6)	*	20-50	Flexible	*	HAMD 17	27.52	8	ICD-10	Europe	Multi-center	Secondary /Tertiary care	Inpatients only	Unclear	Unclear
				amitriptyline	No	108	40.27 (12.7)	*	100-250	Flexible	*	HAMD 17	28.16								
29060/356	unpublished	2	2	paroxetine	Yes	68	42.2 (1.5)	*	20-20	Fixed	20	HAMD 17	24.60	8	DSM-III-R	Australia	Multi-center	Other/Unclear	Both	Unclear	Unclear
				fluoxetine	No	70	40.6 (1.5)	*	20-20	Fixed	20	HAMD 17	23.80								

3 *Risk of bias table of included studies*

For details about the rating criteria, see published protocol (Furukawa et al., BMJ Open 2016). ‘Stated not tested’ refers to the mention of double-blind procedures used in the methods section of a paper, but maintenance of blinding is not tested.

StudyID		Sequence_generation	Allocation_concealment	Blinding_participant	Blinding_therapist	Blinding_assessor	Selective_reporting_bias	Attrition_bias
**1.**	**Aberg-Wisted 2000**	Unclear risk of bias	Unclear risk of bias	Stated but not tested	Stated but not tested	Stated but not tested	Unclear risk of bias	Low risk of bias
**2.**	**Aguglia 1993**	Unclear risk of bias	Unclear risk of bias	Stated but not tested	Stated but not tested	Stated but not tested	Low risk of bias	High risk of bias
**3.**	**AK1102365**	Unclear risk of bias	Unclear risk of bias	Stated but not tested	Stated but not tested	Stated but not tested	Low risk of bias	High risk of bias
**4.**	**Alexopoulos 2004**	Unclear risk of bias	Unclear risk of bias	Stated but not tested	Stated but not tested	Unclear risk of bias	Low risk of bias	Unclear risk of bias
**5.**	**Allard 2004**	Unclear risk of bias	Unclear risk of bias	Stated but not tested	Stated but not tested	Stated but not tested	Unclear risk of bias	Unclear risk of bias
**6.**	**Altamura 1989b**	Unclear risk of bias	Unclear risk of bias	Stated but not tested	Stated but not tested	Unclear risk of bias	Low risk of bias	Low risk of bias
**7.**	**Alvarez 2012**	Low risk of bias	Low risk of bias	Stated but not tested	Stated but not tested	Stated but not tested	Low risk of bias	High risk of bias
**8.**	**Alves 1999**	Low risk of bias	Low risk of bias	Stated but not tested	Stated but not tested	Unclear risk of bias	Low risk of bias	Unclear risk of bias
**9.**	**Amin 1984**	Unclear risk of bias	Unclear risk of bias	Unclear risk of bias	Unclear risk of bias	Unclear risk of bias	Unclear risk of bias	Unclear risk of bias
**10.**	**Amini 2005**	Unclear risk of bias	Unclear risk of bias	Stated but not tested	Stated but not tested	Unclear risk of bias	Low risk of bias	Low risk of bias
**11.**	**Amsterdam 1986**	Unclear risk of bias	Unclear risk of bias	Stated but not tested	Stated but not tested	Unclear risk of bias	Low risk of bias	High risk of bias
**12.**	**Andersen 1986**	Unclear risk of bias	Unclear risk of bias	Stated but not tested	Stated but not tested	Unclear risk of bias	Low risk of bias	Low risk of bias
**13.**	**Ansseau 1989a**	Unclear risk of bias	Unclear risk of bias	Stated but not tested	Stated but not tested	Unclear risk of bias	Unclear risk of bias	High risk of bias
**14.**	**Ansseau 1989b**	Unclear risk of bias	Unclear risk of bias	Stated but not tested	Stated but not tested	Unclear risk of bias	Low risk of bias	Low risk of bias
**15.**	**Ansseau 1991**	Unclear risk of bias	Unclear risk of bias	Stated but not tested	Stated but not tested	Unclear risk of bias	Low risk of bias	High risk of bias
**16.**	**Ansseau 1994a**	Unclear risk of bias	Unclear risk of bias	Stated but not tested	Stated but not tested	Unclear risk of bias	Low risk of bias	Unclear risk of bias
**17.**	**Ansseau 1994b**	Low risk of bias	Unclear risk of bias	Stated but not tested	Stated but not tested	Unclear risk of bias	Low risk of bias	Unclear risk of bias
**18.**	**Ansseau 1994c**	Unclear risk of bias	Unclear risk of bias	Stated but not tested	Stated but not tested	Unclear risk of bias	Low risk of bias	High risk of bias
**19.**	**Armitage 1997**	Unclear risk of bias	Unclear risk of bias	Stated but not tested	Stated but not tested	Unclear risk of bias	Low risk of bias	Unclear risk of bias
**20.**	**Asnis 2013**	Low risk of bias	Unclear risk of bias	Stated but not tested	Stated but not tested	Stated but not tested	Low risk of bias	High risk of bias
**21.**	**Bakish 1992**	Unclear risk of bias	Unclear risk of bias	Stated but not tested	Stated but not tested	Unclear risk of bias	Low risk of bias	High risk of bias
**22.**	**Bakish 1997**	Unclear risk of bias	Unclear risk of bias	Stated but not tested	Stated but not tested	Unclear risk of bias	High risk of bias	Unclear risk of bias
**23.**	**Bakish 2014**	Low risk of bias	Low risk of bias	Stated but not tested	Stated but not tested	Stated but not tested	Low risk of bias	Unclear risk of bias

**24.**	**Baldwin 1996**	Unclear risk of bias	Unclear risk of bias	Stated but not tested	Stated but not tested	Unclear risk of bias	Low risk of bias	Unclear risk of bias
**25.**	**Baldwin 2006a**	Unclear risk of bias	Unclear risk of bias	Stated but not tested	Stated but not tested	Unclear risk of bias	Low risk of bias	High risk of bias
**26.**	**Baldwin 2006b**	Low risk of bias	Low risk of bias	Stated but not tested	Stated but not tested	Unclear risk of bias	Low risk of bias	Low risk of bias
**27.**	**Baldwin 2012**	Low risk of bias	Low risk of bias	Stated but not tested	Stated but not tested	Stated but not tested	Low risk of bias	Unclear risk of bias
**28.**	**Ban 1998**	Unclear risk of bias	Unclear risk of bias	Stated but not tested	Stated but not tested	Unclear risk of bias	Low risk of bias	Low risk of bias
**29.**	**Barber 2011**	Unclear risk of bias	Unclear risk of bias	Stated but not tested	Stated but not tested	Unclear risk of bias	Unclear risk of bias	Unclear risk of bias
**30.**	**Battegay 1985**	Unclear risk of bias	Unclear risk of bias	Stated but not tested	Stated but not tested	Unclear risk of bias	Unclear risk of bias	High risk of bias
**31.**	**Beasley 1991**	Unclear risk of bias	Unclear risk of bias	Stated but not tested	Stated but not tested	Unclear risk of bias	Low risk of bias	Unclear risk of bias
**32.**	**Behnke 2003**	Unclear risk of bias	Unclear risk of bias	Stated but not tested	Stated but not tested	Unclear risk of bias	Low risk of bias	Unclear risk of bias
**33.**	**Benkert 2000**	Unclear risk of bias	Unclear risk of bias	Stated but not tested	Stated but not tested	Unclear risk of bias	Low risk of bias	Unclear risk of bias
**34.**	**Benkert 2006**	Unclear risk of bias	Unclear risk of bias	Stated but not tested	Stated but not tested	Stated but not tested	Low risk of bias	Unclear risk of bias
**35.**	**Bennie1995**	Low risk of bias	Unclear risk of bias	Stated but not tested	Stated but not tested	Unclear risk of bias	Unclear risk of bias	Low risk of bias
**36.**	**Berlanga 1997**	Unclear risk of bias	Unclear risk of bias	Stated but not tested	Stated but not tested	Unclear risk of bias	Low risk of bias	High risk of bias
**37.**	**Berlanga 2006**	Unclear risk of bias	Unclear risk of bias	Stated but not tested	Stated but not tested	Unclear risk of bias	Low risk of bias	Unclear risk of bias
**38.**	**Bersani 1994**	Unclear risk of bias	Unclear risk of bias	Stated but not tested	Stated but not tested	Unclear risk of bias	Low risk of bias	Low risk of bias
**39.**	**Bhatia 1991**	Unclear risk of bias	Unclear risk of bias	Stated but not tested	Stated but not tested	Unclear risk of bias	Unclear risk of bias	Unclear risk of bias
**40.**	**Bielski 2004**	Low risk of bias	Unclear risk of bias	Stated but not tested	Stated but not tested	Unclear risk of bias	Low risk of bias	Unclear risk of bias
**41.**	**Bignamini 1992**	Unclear risk of bias	Unclear risk of bias	Stated but not tested	Stated but not tested	Unclear risk of bias	Low risk of bias	Low risk of bias
**42.**	**Binnemann 2008**	Unclear risk of bias	Unclear risk of bias	Stated but not tested	Stated but not tested	Unclear risk of bias	Low risk of bias	Unclear risk of bias
**43.**	**Bjerkenstedt 2005**	Unclear risk of bias	Unclear risk of bias	Stated but not tested	Stated but not tested	Unclear risk of bias	Low risk of bias	Low risk of bias
**44.**	**Blacker 1988**	Unclear risk of bias	Unclear risk of bias	Stated but not tested	Stated but not tested	Unclear risk of bias	Low risk of bias	Unclear risk of bias
**45.**	**Bosc 1997a**	Unclear risk of bias	Unclear risk of bias	Stated but not tested	Stated but not tested	Unclear risk of bias	Low risk of bias	High risk of bias
**46.**	**Bosc 1997b**	Unclear risk of bias	Unclear risk of bias	Stated but not tested	Stated but not tested	Unclear risk of bias	Low risk of bias	Unclear risk of bias
**47.**	**Bose 2008**	Unclear risk of bias	Unclear risk of bias	Stated but not tested	Stated but not tested	Unclear risk of bias	Low risk of bias	Unclear risk of bias
**48.**	**Bougerol 1997**	Unclear risk of bias	Unclear risk of bias	Stated but not tested	Stated but not tested	Unclear risk of bias	Low risk of bias	Unclear risk of bias
**49.**	**Boulenger 2006**	Low risk of bias	Unclear risk of bias	Stated but not tested	Stated but not tested	Unclear risk of bias	Unclear risk of bias	Low risk of bias
**50.**	**Boulenger 2014**	Low risk of bias	Low risk of bias	Stated but not tested	Stated but not tested	Unclear risk of bias	Low risk of bias	High risk of bias
**51.**	**Boyer 2008**	Unclear risk of bias	Unclear risk of bias	Stated but not tested	Stated but not tested	Unclear risk of bias	Low risk of bias	Low risk of bias
**52.**	**Bremner 1995**	Unclear risk of bias	Unclear risk of bias	Stated but not tested	Stated but not tested	Unclear risk of bias	Low risk of bias	Unclear risk of bias
**53.**	**Brown 1986**	Unclear risk of bias	Unclear risk of bias	Stated but not tested	Stated but not tested	Unclear risk of bias	Unclear risk of bias	Unclear risk of bias
**54.**	**Brunoni 2012**	Low risk of bias	Low risk of bias	Low risk of bias	Stated but not tested	Unclear risk of bias	Unclear risk of bias	Low risk of bias

55.	Buchsbaum 1997	Unclear risk of bias	Unclear risk of bias	Unclear risk of bias	Unclear risk of bias	Unclear risk of bias	Unclear risk of bias	Unclear risk of bias
56.	Bueno 1997	Unclear risk of bias	Unclear risk of bias	Stated but not tested	Stated but not tested	Unclear risk of bias	High risk of bias	Unclear risk of bias
57.	Burke 2002	Unclear risk of bias	Unclear risk of bias	Stated but not tested	Stated but not tested	Unclear risk of bias	Low risk of bias	Unclear risk of bias
58.	Byerley1988	Low risk of bias	Unclear risk of bias	Stated but not tested	Stated but not tested	Unclear risk of bias	Low risk of bias	Unclear risk of bias
59.	CAGO178A2303	Unclear risk of bias	Unclear risk of bias	Stated but not tested	Stated but not tested	Unclear risk of bias	Low risk of bias	Unclear risk of bias
60.	Caligiuri 2003	Unclear risk of bias	Unclear risk of bias	Stated but not tested	Stated but not tested	Unclear risk of bias	Unclear risk of bias	Unclear risk of bias
61.	Carman 1991	Unclear risk of bias	Unclear risk of bias	Stated but not tested	Stated but not tested	Unclear risk of bias	Unclear risk of bias	Unclear risk of bias
62.	Casabona 2002	Unclear risk of bias	Unclear risk of bias	Stated but not tested	Stated but not tested	Unclear risk of bias	Low risk of bias	Low risk of bias
63.	Cassano 1986	Unclear risk of bias	Unclear risk of bias	Stated but not tested	Stated but not tested	Unclear risk of bias	Low risk of bias	Unclear risk of bias
64.	Cassano 2002	Unclear risk of bias	Unclear risk of bias	Stated but not tested	Stated but not tested	Stated but not tested	Unclear risk of bias	Unclear risk of bias
65.	Chang 2015	Unclear risk of bias	Unclear risk of bias	Stated but not tested	Stated but not tested	Stated but not tested	Unclear risk of bias	Unclear risk of bias
66.	Chouinard 1983a	Unclear risk of bias	Unclear risk of bias	Stated but not tested	Stated but not tested	Unclear risk of bias	Unclear risk of bias	High risk of bias
67.	Chouinard 1985	Unclear risk of bias	Unclear risk of bias	Stated but not tested	Stated but not tested	Unclear risk of bias	Low risk of bias	Low risk of bias
68.	Chouinard 1999	Unclear risk of bias	Unclear risk of bias	Stated but not tested	Stated but not tested	Stated but not tested	Low risk of bias	Unclear risk of bias
69.	Christiansen 1996	Low risk of bias	Unclear risk of bias	Stated but not tested	Stated but not tested	Unclear risk of bias	Low risk of bias	Unclear risk of bias
70.	CL3-20098-022	Low risk of bias	Low risk of bias	Stated but not tested	Stated but not tested	Unclear risk of bias	Low risk of bias	High risk of bias
71.	CL3-20098-023	Low risk of bias	Low risk of bias	Stated but not tested	Stated but not tested	Unclear risk of bias	Low risk of bias	Low risk of bias
72.	CL3-20098-024	Low risk of bias	Low risk of bias	Stated but not tested	Stated but not tested	Unclear risk of bias	Low risk of bias	Low risk of bias
73.	CL3-20098-026	Low risk of bias	Low risk of bias	Stated but not tested	Stated but not tested	Unclear risk of bias	Low risk of bias	Unclear risk of bias
74.	CL3-20098-036	Low risk of bias	Low risk of bias	Stated but not tested	Stated but not tested	Unclear risk of bias	Low risk of bias	Low risk of bias
75.	CL3-20098-048	Low risk of bias	Low risk of bias	Stated but not tested	Stated but not tested	Unclear risk of bias	Low risk of bias	Unclear risk of bias
76.	CL3-20098-062	Low risk of bias	Low risk of bias	Stated but not tested	Stated but not tested	Unclear risk of bias	Low risk of bias	Unclear risk of bias
77.	CL3-20098-070	Low risk of bias	Low risk of bias	Stated but not tested	Stated but not tested	Unclear risk of bias	Low risk of bias	Unclear risk of bias
78.	Claghorn 1983	Low risk of bias	Unclear risk of bias	Stated but not tested	Stated but not tested	Unclear risk of bias	Low risk of bias	Unclear risk of bias
79.	Claghorn 1995	Unclear risk of bias	Unclear risk of bias	Stated but not tested	Stated but not tested	Unclear risk of bias	Low risk of bias	High risk of bias
80.	Claghorn 1996	Unclear risk of bias	Unclear risk of bias	Stated but not tested	Stated but not tested	Unclear risk of bias	Low risk of bias	Unclear risk of bias
81.	Clayton 2003	Unclear risk of bias	Unclear risk of bias	Stated but not tested	Stated but not tested	Unclear risk of bias	Low risk of bias	High risk of bias
82.	Clayton 2006a	Unclear risk of bias	Unclear risk of bias	Stated but not tested	Stated but not tested	Unclear risk of bias	Low risk of bias	Unclear risk of bias
83.	Clayton 2006b	Unclear risk of bias	Unclear risk of bias	Stated but not tested	Stated but not tested	Unclear risk of bias	Low risk of bias	Unclear risk of bias
84.	Clayton 2013	Unclear risk of bias	Unclear risk of bias	Stated but not tested	Stated but not tested	Unclear risk of bias	Low risk of bias	Low risk of bias
85.	Clayton 2015	Unclear risk of bias	Unclear risk of bias	Stated but not tested	Stated but not tested	Stated but not tested	Low risk of bias	Unclear risk of bias

**86.**	**Clerc 1994**	Unclear risk of bias	Unclear risk of bias	Stated but not tested	Stated but not tested	Unclear risk of bias	Low risk of bias	High risk of bias
**87.**	**Clerc 2001**	Unclear risk of bias	Unclear risk of bias	Stated but not tested	Stated but not tested	Unclear risk of bias	Low risk of bias	Unclear risk of bias
**88.**	**CN104-002**	Unclear risk of bias	Unclear risk of bias	Stated but not tested	Stated but not tested	Unclear risk of bias	Low risk of bias	High risk of bias
**89.**	**CN104-045**	Unclear risk of bias	Unclear risk of bias	Stated but not tested	Stated but not tested	Unclear risk of bias	High risk of bias	Unclear risk of bias
**90.**	**CN104-054**	Unclear risk of bias	Unclear risk of bias	Stated but not tested	Stated but not tested	Unclear risk of bias	High risk of bias	Unclear risk of bias
**91.**	**Cohn 1985a**	Unclear risk of bias	Unclear risk of bias	Stated but not tested	Stated but not tested	Unclear risk of bias	Low risk of bias	High risk of bias
**92.**	**Cohn1990**	Unclear risk of bias	Unclear risk of bias	Stated but not tested	Stated but not tested	Unclear risk of bias	Low risk of bias	Unclear risk of bias
**93.**	**Cohn 1996**	Unclear risk of bias	Unclear risk of bias	Stated but not tested	Stated but not tested	Unclear risk of bias	Low risk of bias	Unclear risk of bias
**94.**	**Coleman 1999**	Low risk of bias	Unclear risk of bias	Stated but not tested	Stated but not tested	Unclear risk of bias	Low risk of bias	High risk of bias
**95.**	**Coleman 2001**	Unclear risk of bias	Unclear risk of bias	Stated but not tested	Stated but not tested	Unclear risk of bias	Low risk of bias	Unclear risk of bias
**96.**	**Colonna 2005**	Low risk of bias	Low risk of bias	Stated but not tested	Stated but not tested	Unclear risk of bias	Unclear risk of bias	Low risk of bias
**97.**	**Corrigan 2000**	Unclear risk of bias	Unclear risk of bias	Stated but not tested	Stated but not tested	Unclear risk of bias	Low risk of bias	High risk of bias
**98.**	**Corruble 2013**	Low risk of bias	Low risk of bias	Stated but not tested	Stated but not tested	Unclear risk of bias	Low risk of bias	Low risk of bias
**99.**	**Costaesilva 1998**	Unclear risk of bias	Unclear risk of bias	Stated but not tested	Stated but not tested	Unclear risk of bias	Low risk of bias	Low risk of bias
**100.**	**Croft 1999**	Unclear risk of bias	Unclear risk of bias	Stated but not tested	Stated but not tested	Stated but not tested	Low risk of bias	Unclear risk of bias
**101.**	**Croft 2014**	Low risk of bias	Unclear risk of bias	Stated but not tested	Stated but not tested	Unclear risk of bias	Low risk of bias	Low risk of bias
**102.**	**Cunningham 1994**	Unclear risk of bias	Unclear risk of bias	Stated but not tested	Stated but not tested	Unclear risk of bias	Low risk of bias	Unclear risk of bias
**103.**	**Cunningham 1997**	Unclear risk of bias	Unclear risk of bias	Stated but not tested	Stated but not tested	Unclear risk of bias	Low risk of bias	High risk of bias
**104.**	**Cutler 2009**	Low risk of bias	Unclear risk of bias	Stated but not tested	Stated but not tested	Unclear risk of bias	Low risk of bias	Unclear risk of bias
**105.**	**Dalery 2003**	Unclear risk of bias	Unclear risk of bias	Stated but not tested	Stated but not tested	Unclear risk of bias	Unclear risk of bias	Unclear risk of bias
**106.**	**D'Amico 1990**	Unclear risk of bias	Unclear risk of bias	Stated but not tested	Stated but not tested	Unclear risk of bias	Low risk of bias	Unclear risk of bias
**107.**	**Davidson 2002**	Low risk of bias	Low risk of bias	Stated but not tested	Stated but not tested	Unclear risk of bias	Low risk of bias	Unclear risk of bias
**108.**	**Debonnel 2000**	Unclear risk of bias	Unclear risk of bias	Stated but not tested	Stated but not tested	Stated but not tested	Low risk of bias	High risk of bias
**109.**	**Debus 1988**	Unclear risk of bias	Unclear risk of bias	Stated but not tested	Stated but not tested	Unclear risk of bias	Low risk of bias	High risk of bias
**110.**	**DeMartinis 2007**	Unclear risk of bias	Unclear risk of bias	Stated but not tested	Stated but not tested	Unclear risk of bias	Low risk of bias	Unclear risk of bias
**111.**	**Demyttenaere 1998**	Unclear risk of bias	Unclear risk of bias	Stated but not tested	Stated but not tested	Unclear risk of bias	Low risk of bias	High risk of bias
**112.**	**DeRonchi 1998**	Unclear risk of bias	Unclear risk of bias	Stated but not tested	Stated but not tested	Unclear risk of bias	Low risk of bias	Unclear risk of bias
**113.**	**Detke 2002a**	Unclear risk of bias	Unclear risk of bias	Stated but not tested	Stated but not tested	Unclear risk of bias	Low risk of bias	Unclear risk of bias
**114.**	**Detke 2002b**	Unclear risk of bias	Unclear risk of bias	Stated but not tested	Stated but not tested	Unclear risk of bias	Low risk of bias	Unclear risk of bias
**115.**	**Detke 2004**	Unclear risk of bias	Unclear risk of bias	Stated but not tested	Stated but not tested	Unclear risk of bias	Low risk of bias	Low risk of bias
**116.**	**Deushle 2003**	Unclear risk of bias	Unclear risk of bias	Stated but not tested	Stated but not tested	Unclear risk of bias	High risk of bias	High risk of bias

**117.**	**DeWilde 1983a**	Unclear risk of bias	Unclear risk of bias	Stated but not tested	Stated but not tested	Unclear risk of bias	Low risk of bias	Low risk of bias
**118.**	**DeWilde 1993**	Unclear risk of bias	Unclear risk of bias	Stated but not tested	Stated but not tested	Stated but not tested	Low risk of bias	Unclear risk of bias
**119.**	**Dierick 1996**	Unclear risk of bias	Unclear risk of bias	Stated but not tested	Stated but not tested	Unclear risk of bias	Low risk of bias	Unclear risk of bias
**120.**	**Dimidjian 2006**	Low risk of bias	Unclear risk of bias	Stated but not tested	Stated but not tested	Stated but not tested	Low risk of bias	Unclear risk of bias
**121.**	**Dominguez 1985**	Unclear risk of bias	Unclear risk of bias	Stated but not tested	Stated but not tested	Stated but not tested	Low risk of bias	High risk of bias
**122.**	**Doogan 1994**	Low risk of bias	Low risk of bias	Stated but not tested	Stated but not tested	Unclear risk of bias	Unclear risk of bias	Unclear risk of bias
**123.**	**DUAG 1990**	Unclear risk of bias	Unclear risk of bias	Stated but not tested	Stated but not tested	Unclear risk of bias	Low risk of bias	High risk of bias
**124.**	**Dube 2010**	Unclear risk of bias	Unclear risk of bias	Stated but not tested	Stated but not tested	Unclear risk of bias	Low risk of bias	Unclear risk of bias
**125.**	**Dunbar 1993a**	Unclear risk of bias	Unclear risk of bias	Stated but not tested	Stated but not tested	Unclear risk of bias	Low risk of bias	Unclear risk of bias
**126.**	**Dunbar 1993b**	Unclear risk of bias	Unclear risk of bias	Stated but not tested	Stated but not tested	Unclear risk of bias	Low risk of bias	Unclear risk of bias
**127.**	**Dunbar 1993c**	Unclear risk of bias	Unclear risk of bias	Stated but not tested	Stated but not tested	Unclear risk of bias	Low risk of bias	High risk of bias
**128.**	**Dunbar 1993d**	Unclear risk of bias	Unclear risk of bias	Stated but not tested	Stated but not tested	Unclear risk of bias	Low risk of bias	Unclear risk of bias
**129.**	**Dunlop 2011**	Unclear risk of bias	Unclear risk of bias	Stated but not tested	Stated but not tested	Unclear risk of bias	Low risk of bias	Unclear risk of bias
**130.**	**Dunner 1992**	Unclear risk of bias	Unclear risk of bias	Stated but not tested	Stated but not tested	Unclear risk of bias	Low risk of bias	Unclear risk of bias
**131.**	**E-1569**	Unclear risk of bias	Unclear risk of bias	Stated but not tested	Stated but not tested	Stated but not tested	Low risk of bias	Low risk of bias
**132.**	**Edwards 1989**	Unclear risk of bias	Unclear risk of bias	Low risk of bias	Stated but not tested	Unclear risk of bias	Low risk of bias	High risk of bias
**133.**	**Ekselius 1997**	Unclear risk of bias	Unclear risk of bias	Stated but not tested	Stated but not tested	Stated but not tested	Unclear risk of bias	Low risk of bias
**134.**	**Faber 1995**	Low risk of bias	Unclear risk of bias	Stated but not tested	Stated but not tested	Unclear risk of bias	Low risk of bias	High risk of bias
**135.**	**Fabre 1979**	Unclear risk of bias	Unclear risk of bias	Stated but not tested	Stated but not tested	Unclear risk of bias	Unclear risk of bias	Unclear risk of bias
**136.**	**Fabre 1996**	Unclear risk of bias	Unclear risk of bias	Stated but not tested	Stated but not tested	Stated but not tested	Low risk of bias	Unclear risk of bias
**137.**	**Falk 1989**	Unclear risk of bias	Unclear risk of bias	Stated but not tested	Stated but not tested	Unclear risk of bias	Low risk of bias	High risk of bias
**138.**	**Fang 1997**	Unclear risk of bias	Unclear risk of bias	Stated but not tested	Stated but not tested	Unclear risk of bias	High risk of bias	Unclear risk of bias
**139.**	**Fava 1998**	Unclear risk of bias	Unclear risk of bias	Stated but not tested	Stated but not tested	Unclear risk of bias	Low risk of bias	Unclear risk of bias
**140.**	**Fava 2002**	Unclear risk of bias	Unclear risk of bias	Stated but not tested	Stated but not tested	Unclear risk of bias	Low risk of bias	Unclear risk of bias
**141.**	**Fava 2005**	Unclear risk of bias	Unclear risk of bias	Stated but not tested	Stated but not tested	Unclear risk of bias	Low risk of bias	Unclear risk of bias
**142.**	**Fawcett 1989**	Unclear risk of bias	Unclear risk of bias	Stated but not tested	Stated but not tested	Unclear risk of bias	Low risk of bias	High risk of bias
**143.**	**Feiger 1996**	Unclear risk of bias	Unclear risk of bias	Stated but not tested	Stated but not tested	Unclear risk of bias	Low risk of bias	Unclear risk of bias
**144.**	**Feighner 1979**	Low risk of bias	Unclear risk of bias	Stated but not tested	Stated but not tested	Unclear risk of bias	Low risk of bias	Unclear risk of bias
**145.**	**Feighner 1980**	Unclear risk of bias	Unclear risk of bias	Stated but not tested	Stated but not tested	Unclear risk of bias	Unclear risk of bias	High risk of bias
**146.**	**Feighner 1984**	Unclear risk of bias	Unclear risk of bias	Stated but not tested	Stated but not tested	Unclear risk of bias	Low risk of bias	Unclear risk of bias
**147.**	**Feighner 1989a**	Unclear risk of bias	Unclear risk of bias	Stated but not tested	Stated but not tested	Unclear risk of bias	Low risk of bias	High risk of bias

148.	Feighner 1989b	Unclear risk of bias	Unclear risk of bias	Stated but not tested	Stated but not tested	Stated but not tested	Low risk of bias	Unclear risk of bias
149.	Feighner 1991	Unclear risk of bias	Unclear risk of bias	Stated but not tested	Stated but not tested	Unclear risk of bias	Unclear risk of bias	Unclear risk of bias
150.	Feighner 1993a	Unclear risk of bias	Unclear risk of bias	Stated but not tested	Stated but not tested	Unclear risk of bias	Low risk of bias	Unclear risk of bias
151.	Feighner 1993b	Unclear risk of bias	Unclear risk of bias	Stated but not tested	Stated but not tested	Unclear risk of bias	Low risk of bias	High risk of bias
152.	Feighner 1993c	Unclear risk of bias	Unclear risk of bias	Stated but not tested	Stated but not tested	Unclear risk of bias	Low risk of bias	Unclear risk of bias
153.	Feighner 1993d	Unclear risk of bias	Unclear risk of bias	Stated but not tested	Stated but not tested	Unclear risk of bias	Low risk of bias	High risk of bias
154.	Feighner 1993e	Unclear risk of bias	Unclear risk of bias	Stated but not tested	Stated but not tested	Unclear risk of bias	Low risk of bias	Unclear risk of bias
155.	Feighner 1993f	Unclear risk of bias	Unclear risk of bias	Stated but not tested	Stated but not tested	Unclear risk of bias	Low risk of bias	High risk of bias
156.	Feighner 1998	Unclear risk of bias	Unclear risk of bias	Stated but not tested	Stated but not tested	Unclear risk of bias	Low risk of bias	Unclear risk of bias
157.	Feighner 1999	Unclear risk of bias	Unclear risk of bias	Stated but not tested	Stated but not tested	Unclear risk of bias	Low risk of bias	High risk of bias
158.	Fontaine 1994	Unclear risk of bias	Unclear risk of bias	Stated but not tested	Stated but not tested	Unclear risk of bias	Low risk of bias	High risk of bias
159.	Frank 2004	Unclear risk of bias	Unclear risk of bias	Stated but not tested	Stated but not tested	Unclear risk of bias	High risk of bias	Unclear risk of bias
160.	Fudge 1990	Unclear risk of bias	Unclear risk of bias	Stated but not tested	Stated but not tested	Unclear risk of bias	Unclear risk of bias	High risk of bias
161.	Gagiano 1993	Unclear risk of bias	Unclear risk of bias	Stated but not tested	Stated but not tested	Stated but not tested	Low risk of bias	Low risk of bias
162.	Gastpar 2006	Low risk of bias	Unclear risk of bias	Stated but not tested	Stated but not tested	Unclear risk of bias	Low risk of bias	Low risk of bias
163.	Gelenberg 1990	Unclear risk of bias	Unclear risk of bias	Stated but not tested	Stated but not tested	Unclear risk of bias	Low risk of bias	High risk of bias
164.	Gentil 2000	Unclear risk of bias	Unclear risk of bias	Stated but not tested	Stated but not tested	Unclear risk of bias	Low risk of bias	Low risk of bias
165.	Georgotas 1982	Unclear risk of bias	Unclear risk of bias	Stated but not tested	Stated but not tested	Unclear risk of bias	Unclear risk of bias	Unclear risk of bias
166.	Geretsegger 1995	Unclear risk of bias	Unclear risk of bias	Stated but not tested	Stated but not tested	Unclear risk of bias	Low risk of bias	Unclear risk of bias
167.	Gerner 1980	Unclear risk of bias	Unclear risk of bias	Stated but not tested	Stated but not tested	Unclear risk of bias	Unclear risk of bias	Unclear risk of bias
168.	Gillin 1997	Unclear risk of bias	Unclear risk of bias	Stated but not tested	Stated but not tested	Unclear risk of bias	Low risk of bias	Low risk of bias
169.	Ginestet 1989	Unclear risk of bias	Unclear risk of bias	Stated but not tested	Stated but not tested	Unclear risk of bias	Unclear risk of bias	Unclear risk of bias
170.	Golden 2002a	Unclear risk of bias	Unclear risk of bias	Stated but not tested	Stated but not tested	Unclear risk of bias	Low risk of bias	Unclear risk of bias
171.	Golden 2002b	Unclear risk of bias	Unclear risk of bias	Stated but not tested	Stated but not tested	Unclear risk of bias	Low risk of bias	Unclear risk of bias
172.	Goldstein 2002	Unclear risk of bias	Unclear risk of bias	Stated but not tested	Stated but not tested	Unclear risk of bias	Low risk of bias	Unclear risk of bias
173.	Goldstein 2004a	Unclear risk of bias	Unclear risk of bias	Stated but not tested	Stated but not tested	Unclear risk of bias	Low risk of bias	Unclear risk of bias
174.	Goldstein 2004b	Unclear risk of bias	Unclear risk of bias	Stated but not tested	Stated but not tested	Unclear risk of bias	Low risk of bias	Unclear risk of bias
175.	Gommoll 2014	Low risk of bias	Unclear risk of bias	Stated but not tested	Stated but not tested	Stated but not tested	Low risk of bias	Unclear risk of bias
176.	Goodarzi 2015	Unclear risk of bias	Unclear risk of bias	Stated but not tested	Stated but not tested	Unclear risk of bias	High risk of bias	Unclear risk of bias
177.	Gorman 2002	Unclear risk of bias	Unclear risk of bias	Stated but not tested	Stated but not tested	Unclear risk of bias	Low risk of bias	Unclear risk of bias
178.	Griebel 2012	Unclear risk of bias	Unclear risk of bias	Stated but not tested	Stated but not tested	Unclear risk of bias	Low risk of bias	Unclear risk of bias

**179. Griebel 2012b**	Unclear risk of bias	Unclear risk of bias	Stated but not tested	Stated but not tested	Unclear risk of bias	Low risk of bias	Unclear risk of bias
**180. Grunebaum 2011**	Low risk of bias	Low risk of bias	Stated but not tested	Stated but not tested	Stated but not tested	Low risk of bias	High risk of bias
**181. GSK14**	Unclear risk of bias	Unclear risk of bias	Stated but not tested	Stated but not tested	Unclear risk of bias	Low risk of bias	High risk of bias
**182. Guelfi 1995**	Unclear risk of bias	Unclear risk of bias	Stated but not tested	Stated but not tested	Stated but not tested	Low risk of bias	High risk of bias
**183. Guelfi 1998**	Low risk of bias	Unclear risk of bias	Stated but not tested	Stated but not tested	Stated but not tested	Low risk of bias	Unclear risk of bias
**184. Guillibert 1989**	Unclear risk of bias	Unclear risk of bias	Stated but not tested	Stated but not tested	Unclear risk of bias	Low risk of bias	Unclear risk of bias
**185. Hackett 1998**	Unclear risk of bias	Unclear risk of bias	Stated but not tested	Stated but not tested	Unclear risk of bias	Unclear risk of bias	High risk of bias
**186. Hale 2010**	Low risk of bias	Low risk of bias	Stated but not tested	Stated but not tested	Unclear risk of bias	Low risk of bias	Low risk of bias
**187. Halikas 1995**	Unclear risk of bias	Unclear risk of bias	Stated but not tested	Stated but not tested	Unclear risk of bias	Low risk of bias	High risk of bias
**188. Hao 2014**	Unclear risk of bias	Unclear risk of bias	Stated but not tested	Stated but not tested	Stated but not tested	Unclear risk of bias	Unclear risk of bias
**189. Harris 1991**	Unclear risk of bias	Unclear risk of bias	Stated but not tested	Stated but not tested	Unclear risk of bias	Low risk of bias	Unclear risk of bias
**190. Heiligenstein 1994**	Low risk of bias	Unclear risk of bias	Stated but not tested	Stated but not tested	Unclear risk of bias	Low risk of bias	Unclear risk of bias
**191. Heller 2009**	Unclear risk of bias	Unclear risk of bias	Stated but not tested	Stated but not tested	Unclear risk of bias	Unclear risk of bias	High risk of bias
**192. Henigsberg 2012**	Unclear risk of bias	Unclear risk of bias	Stated but not tested	Stated but not tested	Unclear risk of bias	Low risk of bias	Low risk of bias
**193. Heun 2013**	Unclear risk of bias	Low risk of bias	Stated but not tested	Stated but not tested	Unclear risk of bias	Low risk of bias	Unclear risk of bias
**194. Hewett 2009**	Unclear risk of bias	Unclear risk of bias	Stated but not tested	Stated but not tested	Unclear risk of bias	Low risk of bias	Low risk of bias
**195. Hewett 2010a**	Unclear risk of bias	Unclear risk of bias	Stated but not tested	Stated but not tested	Unclear risk of bias	Low risk of bias	Unclear risk of bias
**196. Hewett 2010b**	Unclear risk of bias	Unclear risk of bias	Stated but not tested	Stated but not tested	Unclear risk of bias	Low risk of bias	Unclear risk of bias
**197. Hicks 1988**	Low risk of bias	Unclear risk of bias	Stated but not tested	Stated but not tested	Unclear risk of bias	Low risk of bias	High risk of bias
**198. Hicks 2002**	Unclear risk of bias	Unclear risk of bias	Stated but not tested	Stated but not tested	Unclear risk of bias	Low risk of bias	Low risk of bias
**199. Higuchi 2014**	Unclear risk of bias	Unclear risk of bias	Stated but not tested	Stated but not tested	Stated but not tested	Unclear risk of bias	Low risk of bias
**200. Higuchi 2009**	Low risk of bias	Low risk of bias	Stated but not tested	Stated but not tested	Stated but not tested	Low risk of bias	Low risk of bias
**201. Higuchi 2011**	Unclear risk of bias	Unclear risk of bias	Stated but not tested	Stated but not tested	Unclear risk of bias	Low risk of bias	Low risk of bias
**202. Hirayasu 2011a**	Unclear risk of bias	Unclear risk of bias	Stated but not tested	Stated but not tested	Stated but not tested	Low risk of bias	Low risk of bias
**203. Hirayasu 2011b**	Unclear risk of bias	Unclear risk of bias	Stated but not tested	Stated but not tested	Stated but not tested	Low risk of bias	Low risk of bias
**204. Hong 2003**	Unclear risk of bias	Unclear risk of bias	Stated but not tested	Stated but not tested	Unclear risk of bias	Low risk of bias	High risk of bias
**205. Hormazabal 1985**	Unclear risk of bias	Unclear risk of bias	Stated but not tested	Stated but not tested	Unclear risk of bias	Low risk of bias	Low risk of bias
**206. Hosseini 2015**	Unclear risk of bias	Unclear risk of bias	Stated but not tested	Stated but not tested	Unclear risk of bias	Low risk of bias	High risk of bias
**207. Hoyberg 1996**	Low risk of bias	Unclear risk of bias	Stated but not tested	Stated but not tested	Unclear risk of bias	Low risk of bias	Unclear risk of bias
**208. Hsu 2011**	Unclear risk of bias	Unclear risk of bias	Stated but not tested	Stated but not tested	Unclear risk of bias	Unclear risk of bias	Unclear risk of bias
**209. Hu 2009**	Unclear risk of bias	Unclear risk of bias	Stated but not tested	Stated but not tested	Unclear risk of bias	Unclear risk of bias	Unclear risk of bias

210.	Hunter 2010 (Study 1)	Unclear risk of bias	Unclear risk of bias	Stated but not tested	Stated but not tested	Unclear risk of bias	High risk of bias	Unclear risk of bias
211.	Hunter 2010 (Study 2)	Unclear risk of bias	Unclear risk of bias	Stated but not tested	Stated but not tested	Unclear risk of bias	High risk of bias	Unclear risk of bias
212.	Hunter 2010 (Study 3)	Unclear risk of bias	Unclear risk of bias	Stated but not tested	Stated but not tested	Unclear risk of bias	High risk of bias	Unclear risk of bias
213.	Hutchinson 1992	Unclear risk of bias	Unclear risk of bias	Stated but not tested	Stated but not tested	Unclear risk of bias	Low risk of bias	High risk of bias
214.	Itil 1983	Unclear risk of bias	Unclear risk of bias	Stated but not tested	Stated but not tested	Unclear risk of bias	Unclear risk of bias	Unclear risk of bias
215.	Iwata 2013	Unclear risk of bias	Low risk of bias	Stated but not tested	Stated but not tested	Unclear risk of bias	Low risk of bias	Low risk of bias
216.	Jacobsen 2015	Unclear risk of bias	Unclear risk of bias	Stated but not tested	Stated but not tested	Unclear risk of bias	Low risk of bias	Low risk of bias
217.	Jain 2013	Low risk of bias	Low risk of bias	Stated but not tested	Stated but not tested	Unclear risk of bias	Low risk of bias	Low risk of bias
218.	Jefferson 2000	Unclear risk of bias	Unclear risk of bias	Stated but not tested	Stated but not tested	Unclear risk of bias	Low risk of bias	High risk of bias
219.	Jefferson 2006	Unclear risk of bias	Unclear risk of bias	Stated but not tested	Stated but not tested	Unclear risk of bias	Low risk of bias	Unclear risk of bias
220.	Jiang 2009	Unclear risk of bias	Unclear risk of bias	Stated but not tested	Stated but not tested	Stated but not tested	Low risk of bias	Low risk of bias
221.	Judd 1993	Unclear risk of bias	Unclear risk of bias	Stated but not tested	Stated but not tested	Unclear risk of bias	Low risk of bias	Unclear risk of bias
222.	Kamijima 2013	Unclear risk of bias	Unclear risk of bias	Stated but not tested	Stated but not tested	Stated but not tested	Low risk of bias	Low risk of bias
223.	Kane 1983	Unclear risk of bias	Unclear risk of bias	Stated but not tested	Stated but not tested	Unclear risk of bias	Low risk of bias	Low risk of bias
224.	Kasper 2005a	Unclear risk of bias	Unclear risk of bias	Stated but not tested	Stated but not tested	Unclear risk of bias	Low risk of bias	High risk of bias
225.	Kasper 2005b	Unclear risk of bias	Unclear risk of bias	Stated but not tested	Stated but not tested	Unclear risk of bias	Low risk of bias	Low risk of bias
226.	Kasper 2012	Low risk of bias	Unclear risk of bias	Stated but not tested	Stated but not tested	Stated but not tested	Low risk of bias	High risk of bias
227.	Katona 2012	Low risk of bias	Low risk of bias	Stated but not tested	Stated but not tested	Unclear risk of bias	Low risk of bias	Low risk of bias
228.	Katz 1993a	Unclear risk of bias	Unclear risk of bias	Stated but not tested	Stated but not tested	Unclear risk of bias	Unclear risk of bias	Unclear risk of bias
229.	Katz 1993b	Unclear risk of bias	Unclear risk of bias	Stated but not tested	Stated but not tested	Unclear risk of bias	Unclear risk of bias	Unclear risk of bias
230.	Katz 2004	Unclear risk of bias	Unclear risk of bias	Stated but not tested	Stated but not tested	Unclear risk of bias	Low risk of bias	Low risk of bias
231.	Kavoussi 1997	Unclear risk of bias	Unclear risk of bias	Stated but not tested	Stated but not tested	Stated but not tested	Unclear risk of bias	Low risk of bias
232.	Keegan 1991	Unclear risk of bias	Unclear risk of bias	Stated but not tested	Stated but not tested	Unclear risk of bias	Low risk of bias	Low risk of bias
233.	Kellams 1979	Unclear risk of bias	Unclear risk of bias	Stated but not tested	Stated but not tested	Unclear risk of bias	Low risk of bias	High risk of bias
234.	Keller 2006a	Unclear risk of bias	Unclear risk of bias	Stated but not tested	Stated but not tested	Unclear risk of bias	Unclear risk of bias	Unclear risk of bias
235.	Keller 2006b	Unclear risk of bias	Unclear risk of bias	Stated but not tested	Stated but not tested	Unclear risk of bias	Unclear risk of bias	Unclear risk of bias
236.	Keller 2006c	Unclear risk of bias	Unclear risk of bias	Stated but not tested	Stated but not tested	Unclear risk of bias	Low risk of bias	Unclear risk of bias
237.	Keller 2007	Unclear risk of bias	Unclear risk of bias	Stated but not tested	Stated but not tested	Stated but not tested	Unclear risk of bias	Unclear risk of bias
238.	Kennedy 2005	Unclear risk of bias	Unclear risk of bias	Stated but not tested	Stated but not tested	Unclear risk of bias	Low risk of bias	High risk of bias
239.	Kennedy 2008	Low risk of bias	Low risk of bias	Stated but not tested	Stated but not tested	Unclear risk of bias	Low risk of bias	Low risk of bias
240.	Kennedy 2014	Unclear risk of bias	Low risk of bias	Stated but not tested	Stated but not tested	Unclear risk of bias	Low risk of bias	Low risk of bias

**241.**	**Kerkhofs 1990**	Unclear risk of bias	Unclear risk of bias	Stated but not tested	Stated but not tested	Unclear risk of bias	Unclear risk of bias	Unclear risk of bias
**242.**	**Kerr 1984**	Unclear risk of bias	Unclear risk of bias	Stated but not tested	Stated but not tested	Unclear risk of bias	Unclear risk of bias	Unclear risk of bias
**243.**	**Khan 1998**	Unclear risk of bias	Unclear risk of bias	Stated but not tested	Stated but not tested	Unclear risk of bias	Low risk of bias	Unclear risk of bias
**244.**	**Khan 2007**	Unclear risk of bias	Unclear risk of bias	Stated but not tested	Stated but not tested	Unclear risk of bias	Low risk of bias	High risk of bias
**245.**	**Khan 2011**	Unclear risk of bias	Unclear risk of bias	Stated but not tested	Stated but not tested	Unclear risk of bias	Low risk of bias	Low risk of bias
**246.**	**Kiev 1997**	Unclear risk of bias	Unclear risk of bias	Stated but not tested	Stated but not tested	Unclear risk of bias	Low risk of bias	Unclear risk of bias
**247.**	**Kinoshita 2009**	Unclear risk of bias	Unclear risk of bias	Stated but not tested	Stated but not tested	Stated but not tested	Low risk of bias	Unclear risk of bias
**248.**	**Koshino 2013**	Unclear risk of bias	Low risk of bias	Stated but not tested	Stated but not tested	Stated but not tested	Low risk of bias	Unclear risk of bias
**249.**	**Kramer 1998**	Unclear risk of bias	Unclear risk of bias	Stated but not tested	Stated but not tested	Unclear risk of bias	Low risk of bias	Unclear risk of bias
**250.**	**Kuhs 1989**	Unclear risk of bias	Unclear risk of bias	Stated but not tested	Stated but not tested	Unclear risk of bias	Unclear risk of bias	Unclear risk of bias
**251.**	**Kusalic 1993**	Unclear risk of bias	Unclear risk of bias	Stated but not tested	Stated but not tested	Unclear risk of bias	Unclear risk of bias	Unclear risk of bias
**252.**	**Kyle 1998**	Unclear risk of bias	Unclear risk of bias	Stated but not tested	Stated but not tested	Unclear risk of bias	Unclear risk of bias	Unclear risk of bias
**253.**	**Laakman 1988**	Unclear risk of bias	Unclear risk of bias	Stated but not tested	Stated but not tested	Unclear risk of bias	Unclear risk of bias	Unclear risk of bias
**254.**	**Lalit 2004**	Unclear risk of bias	Unclear risk of bias	Stated but not tested	Stated but not tested	Unclear risk of bias	Low risk of bias	High risk of bias
**255.**	**Lam 1995**	Unclear risk of bias	Unclear risk of bias	Stated but not tested	Stated but not tested	Unclear risk of bias	Low risk of bias	Unclear risk of bias
**256.**	**Langlois 1985**	Unclear risk of bias	Unclear risk of bias	Stated but not tested	Stated but not tested	Unclear risk of bias	High risk of bias	Unclear risk of bias
**257.**	**Lapierre 1980**	Unclear risk of bias	Unclear risk of bias	Stated but not tested	Stated but not tested	Unclear risk of bias	High risk of bias	Low risk of bias
**258.**	**Lapierre 1987**	Unclear risk of bias	Unclear risk of bias	Stated but not tested	Stated but not tested	Unclear risk of bias	Low risk of bias	High risk of bias
**259.**	**Larsen 1989**	Unclear risk of bias	Unclear risk of bias	Stated but not tested	Stated but not tested	Unclear risk of bias	Unclear risk of bias	Unclear risk of bias
**260.**	**Learned 2012a Study1**	Unclear risk of bias	Unclear risk of bias	Stated but not tested	Stated but not tested	Unclear risk of bias	Low risk of bias	Unclear risk of bias
**261.**	**Learned 2012b Study2**	Unclear risk of bias	Unclear risk of bias	Stated but not tested	Stated but not tested	Unclear risk of bias	Low risk of bias	Unclear risk of bias
**262.**	**Lecrubier 1997**	Unclear risk of bias	Unclear risk of bias	Stated but not tested	Stated but not tested	Unclear risk of bias	Low risk of bias	Unclear risk of bias
**263.**	**Lee 2007**	Unclear risk of bias	Unclear risk of bias	Stated but not tested	Stated but not tested	Unclear risk of bias	Low risk of bias	Unclear risk of bias
**264.**	**Leinonen 1997**	Low risk of bias	Unclear risk of bias	Stated but not tested	Stated but not tested	Unclear risk of bias	Low risk of bias	Unclear risk of bias
**265.**	**Leinonen 1999**	Low risk of bias	Low risk of bias	Stated but not tested	Stated but not tested	Unclear risk of bias	Low risk of bias	Low risk of bias
**266.**	**Lemoine 2007**	Low risk of bias	Low risk of bias	Stated but not tested	Stated but not tested	Unclear risk of bias	Low risk of bias	High risk of bias
**267.**	**Lepine 2000**	Unclear risk of bias	Unclear risk of bias	Stated but not tested	Stated but not tested	Unclear risk of bias	Low risk of bias	Low risk of bias
**268.**	**Lepola 2003**	Unclear risk of bias	Unclear risk of bias	Stated but not tested	Stated but not tested	Stated but not tested	Low risk of bias	Low risk of bias
**269.**	**Li 2006**	Low risk of bias	Unclear risk of bias	Stated but not tested	Stated but not tested	Unclear risk of bias	Low risk of bias	Unclear risk of bias
**270.**	**Li 2010**	Unclear risk of bias	Unclear risk of bias	Stated but not tested	Stated but not tested	Unclear risk of bias	Unclear risk of bias	Low risk of bias
**271.**	**Lieberman 2008a**	Unclear risk of bias	Unclear risk of bias	Stated but not tested	Stated but not tested	Unclear risk of bias	Low risk of bias	Low risk of bias

272.	**Lieberman 2008b**	Unclear risk of bias	Unclear risk of bias	Stated but not tested	Stated but not tested	Unclear risk of bias	Low risk of bias	Low risk of bias
273.	**Liebowitz 2008**	Unclear risk of bias	Unclear risk of bias	Stated but not tested	Stated but not tested	Unclear risk of bias	Low risk of bias	Low risk of bias
274.	**Liebowitz 2013**	Low risk of bias	Low risk of bias	Stated but not tested	Stated but not tested	Unclear risk of bias	Low risk of bias	Low risk of bias
275.	**Lineberry 1990**	Unclear risk of bias	Unclear risk of bias	Stated but not tested	Stated but not tested	Unclear risk of bias	Low risk of bias	Unclear risk of bias
276.	**Loo 2002**	Unclear risk of bias	Unclear risk of bias	Stated but not tested	Stated but not tested	Unclear risk of bias	Low risk of bias	Unclear risk of bias
277.	**LopezRodriguez 2004**	Unclear risk of bias	Unclear risk of bias	Stated but not tested	Stated but not tested	Stated but not tested	Unclear risk of bias	Unclear risk of bias
278.	**Lv 2013**	Unclear risk of bias	Unclear risk of bias	Stated but not tested	Stated but not tested	Unclear risk of bias	Unclear risk of bias	Unclear risk of bias
279.	**Lydiard 1989**	Unclear risk of bias	Unclear risk of bias	Stated but not tested	Stated but not tested	Unclear risk of bias	Low risk of bias	Unclear risk of bias
280.	**Lydiard 1997**	Unclear risk of bias	Unclear risk of bias	Stated but not tested	Stated but not tested	Unclear risk of bias	Low risk of bias	High risk of bias
281.	**M/2020/0046**	Unclear risk of bias	Unclear risk of bias	Stated but not tested	Stated but not tested	Unclear risk of bias	Low risk of bias	Unclear risk of bias
282.	**M/2020/0047**	Unclear risk of bias	Unclear risk of bias	Stated but not tested	Stated but not tested	Unclear risk of bias	Low risk of bias	Unclear risk of bias
283.	**Mahableshwarkar 2013**	Low risk of bias	Low risk of bias	Stated but not tested	Stated but not tested	Unclear risk of bias	Low risk of bias	Unclear risk of bias
284.	**Mahableshwarkar 2015a**	Unclear risk of bias	Low risk of bias	Stated but not tested	Stated but not tested	Unclear risk of bias	Low risk of bias	Unclear risk of bias
285.	**Mahableshwarkar 2015b**	Unclear risk of bias	Low risk of bias	Stated but not tested	Stated but not tested	Stated but not tested	Low risk of bias	Low risk of bias
286.	**Mahableshwarkar 2015c**	Unclear risk of bias	Low risk of bias	Stated but not tested	Stated but not tested	Stated but not tested	Low risk of bias	Unclear risk of bias
287.	**Mann 1981**	Unclear risk of bias	Unclear risk of bias	Stated but not tested	Stated but not tested	Unclear risk of bias	Unclear risk of bias	Unclear risk of bias
288.	**Manna 1989**	Unclear risk of bias	Unclear risk of bias	Stated but not tested	Stated but not tested	Unclear risk of bias	Unclear risk of bias	Unclear risk of bias
289.	**Mao 2008**	Unclear risk of bias	Unclear risk of bias	Stated but not tested	Stated but not tested	Unclear risk of bias	Low risk of bias	Low risk of bias
290.	**Mao 2015**	Low risk of bias	Unclear risk of bias	Stated but not tested	Stated but not tested	Stated but not tested	Low risk of bias	High risk of bias
291.	**March 1990**	Low risk of bias	Unclear risk of bias	Stated but not tested	Stated but not tested	Unclear risk of bias	Low risk of bias	Unclear risk of bias
292.	**Marchesi 1998**	Unclear risk of bias	Unclear risk of bias	Stated but not tested	Stated but not tested	Unclear risk of bias	Low risk of bias	Low risk of bias
293.	**Masco 1985**	Unclear risk of bias	Unclear risk of bias	Stated but not tested	Stated but not tested	Unclear risk of bias	Low risk of bias	High risk of bias
294.	**Mathews 2015**	Low risk of bias	Unclear risk of bias	Stated but not tested	Stated but not tested	Unclear risk of bias	Low risk of bias	Unclear risk of bias
295.	**McGrath 2000**	Unclear risk of bias	Unclear risk of bias	Stated but not tested	Stated but not tested	Unclear risk of bias	Unclear risk of bias	High risk of bias
296.	**McIntyre 2014**	Low risk of bias	Low risk of bias	Stated but not tested	Stated but not tested	Unclear risk of bias	Low risk of bias	Low risk of bias
297.	**McPartlin 1998**	Unclear risk of bias	Unclear risk of bias	Stated but not tested	Stated but not tested	Unclear risk of bias	Low risk of bias	Unclear risk of bias
298.	**MDF/29060/III/070/88/MC**	Unclear risk of bias	Unclear risk of bias	Stated but not tested	Stated but not tested	Unclear risk of bias	Low risk of bias	Unclear risk of bias
299.	**MDUK/26090/III/83/007**	Unclear risk of bias	Unclear risk of bias	Stated but not tested	Stated but not tested	Unclear risk of bias	Unclear risk of bias	Unclear risk of bias
300.	**Mehtonen 2000**	Unclear risk of bias	Unclear risk of bias	Stated but not tested	Stated but not tested	Stated but not tested	Low risk of bias	Low risk of bias
301.	**Mendels 1995**	Unclear risk of bias	Unclear risk of bias	Stated but not tested	Stated but not tested	Unclear risk of bias	Low risk of bias	Unclear risk of bias

302.	Mendels 1999	Unclear risk of bias	Unclear risk of bias	Stated but not tested	Stated but not tested	Unclear risk of bias	Low risk of bias	Unclear risk of bias
303.	Miller 1989	Unclear risk of bias	Unclear risk of bias	Stated but not tested	Stated but not tested	Unclear risk of bias	Unclear risk of bias	High risk of bias
304.	MIR 003-003	Unclear risk of bias	Unclear risk of bias	Unclear risk of bias	Unclear risk of bias	Unclear risk of bias	Low risk of bias	Unclear risk of bias
305.	MIR 003-020	Unclear risk of bias	Unclear risk of bias	Stated but not tested	Stated but not tested	Unclear risk of bias	Low risk of bias	Unclear risk of bias
306.	MIR 003-021	Unclear risk of bias	Unclear risk of bias	Stated but not tested	Stated but not tested	Unclear risk of bias	Low risk of bias	High risk of bias
307.	MIR 84062	Unclear risk of bias	Unclear risk of bias	Stated but not tested	Stated but not tested	Unclear risk of bias	Unclear risk of bias	Unclear risk of bias
308.	Mischoulon 2014	Unclear risk of bias	Unclear risk of bias	Stated but not tested	Stated but not tested	Unclear risk of bias	Low risk of bias	Unclear risk of bias
309.	Miura 2000	Unclear risk of bias	Unclear risk of bias	Stated but not tested	Stated but not tested	Stated but not tested	Low risk of bias	Unclear risk of bias
310.	Moises 1981	Unclear risk of bias	Unclear risk of bias	Stated but not tested	Stated but not tested	Unclear risk of bias	Unclear risk of bias	High risk of bias
311.	Moller 1993	Unclear risk of bias	Unclear risk of bias	Stated but not tested	Stated but not tested	Unclear risk of bias	Low risk of bias	Unclear risk of bias
312.	Moller 1998	Unclear risk of bias	Unclear risk of bias	Stated but not tested	Stated but not tested	Unclear risk of bias	Low risk of bias	Low risk of bias
313.	Moller 2000	Unclear risk of bias	Unclear risk of bias	Stated but not tested	Stated but not tested	Unclear risk of bias	Low risk of bias	Low risk of bias
314.	Montgomery 1992	Unclear risk of bias	Unclear risk of bias	Stated but not tested	Stated but not tested	Unclear risk of bias	Low risk of bias	Unclear risk of bias
315.	Montgomery 2004a	Unclear risk of bias	Unclear risk of bias	Stated but not tested	Stated but not tested	Stated but not tested	Low risk of bias	Low risk of bias
316.	Montgomery 2004b	Low risk of bias	Low risk of bias	Stated but not tested	Stated but not tested	Unclear risk of bias	Low risk of bias	Unclear risk of bias
317.	Montgomery 2013	Low risk of bias	Low risk of bias	Stated but not tested	Stated but not tested	Unclear risk of bias	Low risk of bias	Unclear risk of bias
318.	Moon 1994	Unclear risk of bias	Unclear risk of bias	Stated but not tested	Stated but not tested	Unclear risk of bias	Low risk of bias	Unclear risk of bias
319.	Moore 2005	Low risk of bias	Unclear risk of bias	Stated but not tested	Stated but not tested	Stated but not tested	Low risk of bias	Low risk of bias
320.	Moreno 2005	Unclear risk of bias	Unclear risk of bias	Stated but not tested	Stated but not tested	Unclear risk of bias	Unclear risk of bias	Unclear risk of bias
321.	Moscovitch 2004	Low risk of bias	Low risk of bias	Stated but not tested	Stated but not tested	Unclear risk of bias	Low risk of bias	Unclear risk of bias
322.	Mullin 1996	Unclear risk of bias	Unclear risk of bias	Stated but not tested	Stated but not tested	Unclear risk of bias	Low risk of bias	Unclear risk of bias
323.	Mundt 2012	Unclear risk of bias	Unclear risk of bias	Stated but not tested	Stated but not tested	Unclear risk of bias	Low risk of bias	Unclear risk of bias
324.	Munizza 2006	Low risk of bias	Unclear risk of bias	Stated but not tested	Stated but not tested	Unclear risk of bias	Low risk of bias	Low risk of bias
325.	Murasaki 1998	Unclear risk of bias	Unclear risk of bias	Stated but not tested	Stated but not tested	Stated but not tested	Low risk of bias	Unclear risk of bias
326.	Murasaki 2010a	Unclear risk of bias	Unclear risk of bias	Stated but not tested	Stated but not tested	Stated but not tested	Low risk of bias	Low risk of bias
327.	Murasaki 2010b	Unclear risk of bias	Unclear risk of bias	Stated but not tested	Stated but not tested	Stated but not tested	Low risk of bias	Unclear risk of bias
328.	MY-1008/CPMS-076	Unclear risk of bias	Unclear risk of bias	Stated but not tested	Stated but not tested	Unclear risk of bias	Unclear risk of bias	Unclear risk of bias
329.	MY-1042/CPMS-251	Unclear risk of bias	Unclear risk of bias	Stated but not tested	Stated but not tested	Unclear risk of bias	Low risk of bias	Unclear risk of bias
330.	MY-1043/BRL-029060/115	Unclear risk of bias	Unclear risk of bias	Stated but not tested	Stated but not tested	Unclear risk of bias	Low risk of bias	Unclear risk of bias
331.	MY-1045/BRL-029060/1	Unclear risk of bias	Unclear risk of bias	Stated but not tested	Stated but not tested	Unclear risk of bias	Low risk of bias	Unclear risk of bias

332.	Mynors-Wallis 1995	Low risk of bias	Low risk of bias	Stated but not tested	Stated but not tested	Unclear risk of bias	Low risk of bias	High risk of bias
333.	NCT00822744	Unclear risk of bias	Unclear risk of bias	Stated but not tested	Stated but not tested	Unclear risk of bias	Unclear risk of bias	Unclear risk of bias
334.	NCT01020799	Unclear risk of bias	Unclear risk of bias	Stated but not tested	Stated but not tested	Stated but not tested	Low risk of bias	Low risk of bias
335.	NCT01145755	Unclear risk of bias	Unclear risk of bias	Stated but not tested	Stated but not tested	Stated but not tested	Unclear risk of bias	Unclear risk of bias
336.	NCT01254305	Unclear risk of bias	Unclear risk of bias	Stated but not tested	Stated but not tested	Stated but not tested	Unclear risk of bias	Unclear risk of bias
337.	NCT01255787	Unclear risk of bias	Unclear risk of bias	Stated but not tested	Stated but not tested	Stated but not tested	Low risk of bias	Low risk of bias
338.	NCT01355081	Unclear risk of bias	Unclear risk of bias	Stated but not tested	Stated but not tested	Stated but not tested	Low risk of bias	Low risk of bias
339.	NCT01808612	Unclear risk of bias	Unclear risk of bias	Stated but not tested	Stated but not tested	Stated but not tested	Low risk of bias	Low risk of bias
340.	Nemeroff 1995	Unclear risk of bias	Unclear risk of bias	Stated but not tested	Stated but not tested	Unclear risk of bias	Low risk of bias	High risk of bias
341.	Nemeroff 2007	Unclear risk of bias	Unclear risk of bias	Stated but not tested	Stated but not tested	Unclear risk of bias	Low risk of bias	Unclear risk of bias
342.	Newhouse 2000	Unclear risk of bias	Unclear risk of bias	Stated but not tested	Stated but not tested	Unclear risk of bias	Low risk of bias	Unclear risk of bias
343.	Nierenberg 2007	Low risk of bias	Low risk of bias	Stated but not tested	Stated but not tested	Unclear risk of bias	Low risk of bias	Unclear risk of bias
344.	Ninan 2003	Unclear risk of bias	Unclear risk of bias	Stated but not tested	Stated but not tested	Unclear risk of bias	Low risk of bias	Unclear risk of bias
345.	NKD20006	Unclear risk of bias	Low risk of bias	Stated but not tested	Stated but not tested	Unclear risk of bias	Low risk of bias	High risk of bias
346.	Noguera 1991	Unclear risk of bias	Unclear risk of bias	Stated but not tested	Stated but not tested	Unclear risk of bias	Low risk of bias	Unclear risk of bias
347.	Norton 1984	Unclear risk of bias	Unclear risk of bias	Stated but not tested	Stated but not tested	Unclear risk of bias	Low risk of bias	Low risk of bias
348.	Oakes 2012a	Low risk of bias	Low risk of bias	Stated but not tested	Stated but not tested	Stated but not tested	Unclear risk of bias	Unclear risk of bias
349.	Oakes 2012b	Low risk of bias	Low risk of bias	Stated but not tested	Stated but not tested	Stated but not tested	Unclear risk of bias	Unclear risk of bias
350.	Olie 1997	Unclear risk of bias	Unclear risk of bias	Stated but not tested	Stated but not tested	Unclear risk of bias	Low risk of bias	Unclear risk of bias
351.	Olie 2007	Low risk of bias	Low risk of bias	Stated but not tested	Stated but not tested	Unclear risk of bias	Low risk of bias	Low risk of bias
352.	Ontiveros 1994	Unclear risk of bias	Unclear risk of bias	Stated but not tested	Stated but not tested	Unclear risk of bias	Low risk of bias	Low risk of bias
353.	OntiverosSanchez 1998	Unclear risk of bias	Unclear risk of bias	Stated but not tested	Stated but not tested	Unclear risk of bias	Low risk of bias	High risk of bias
354.	Ottevanger 1995	Unclear risk of bias	Unclear risk of bias	Stated but not tested	Stated but not tested	Unclear risk of bias	Low risk of bias	Low risk of bias
355.	Ou 2010	Low risk of bias	Low risk of bias	Stated but not tested	Stated but not tested	Unclear risk of bias	Low risk of bias	Low risk of bias
356.	PAR 01 001	Unclear risk of bias	Unclear risk of bias	Stated but not tested	Stated but not tested	Unclear risk of bias	Low risk of bias	Unclear risk of bias
357.	PAR 279 MDUK	Unclear risk of bias	Unclear risk of bias	Stated but not tested	Stated but not tested	Unclear risk of bias	Low risk of bias	Unclear risk of bias
358.	PAR 29060.308	Unclear risk of bias	Unclear risk of bias	Stated but not tested	Stated but not tested	Unclear risk of bias	Unclear risk of bias	Low risk of bias
359.	PAR 29060.310	Unclear risk of bias	Unclear risk of bias	Stated but not tested	Stated but not tested	Unclear risk of bias	Low risk of bias	Unclear risk of bias
360.	PAR 29060.314	Unclear risk of bias	Unclear risk of bias	Stated but not tested	Stated but not tested	Unclear risk of bias	Unclear risk of bias	High risk of bias
361.	PAR 29060.316	Unclear risk of bias	Unclear risk of bias	Stated but not tested	Stated but not tested	Unclear risk of bias	Unclear risk of bias	Unclear risk of bias
362.	PAR 29060.318	Unclear risk of bias	Unclear risk of bias	Stated but not tested	Stated but not tested	Unclear risk of bias	Low risk of bias	Low risk of bias

363.	PAR 29060/281	Unclear risk of bias	Unclear risk of bias	Stated but not tested	Stated but not tested	Unclear risk of bias	Low risk of bias	High risk of bias
364.	PAR MDUK 032	Unclear risk of bias	Unclear risk of bias	Stated but not tested	Stated but not tested	Unclear risk of bias	Low risk of bias	Unclear risk of bias
365.	Patris 1996	Unclear risk of bias	Unclear risk of bias	Stated but not tested	Stated but not tested	Unclear risk of bias	Low risk of bias	Low risk of bias
366.	Paykel 1988	Low risk of bias	Unclear risk of bias	Stated but not tested	Stated but not tested	Unclear risk of bias	Unclear risk of bias	Unclear risk of bias
367.	Pelicier 1993	Unclear risk of bias	Unclear risk of bias	Stated but not tested	Stated but not tested	Unclear risk of bias	Low risk of bias	Unclear risk of bias
368.	Perahia 2006	Unclear risk of bias	Unclear risk of bias	Stated but not tested	Stated but not tested	Unclear risk of bias	Low risk of bias	Low risk of bias
369.	Perahia 2008a	Unclear risk of bias	Unclear risk of bias	Stated but not tested	Stated but not tested	Unclear risk of bias	Low risk of bias	Low risk of bias
370.	Perahia 2008b	Unclear risk of bias	Unclear risk of bias	Stated but not tested	Stated but not tested	Unclear risk of bias	Low risk of bias	Unclear risk of bias
371.	Pomara 2013	Unclear risk of bias	Unclear risk of bias	Stated but not tested	Stated but not tested	Unclear risk of bias	High risk of bias	Unclear risk of bias
372.	Preskorn 1991	Unclear risk of bias	Unclear risk of bias	Stated but not tested	Stated but not tested	Unclear risk of bias	Low risk of bias	High risk of bias
373.	PZ/109	Unclear risk of bias	Unclear risk of bias	Unclear risk of bias	Unclear risk of bias	Unclear risk of bias	Unclear risk of bias	High risk of bias
374.	PZ/111	Unclear risk of bias	Unclear risk of bias	Unclear risk of bias	Unclear risk of bias	Unclear risk of bias	Unclear risk of bias	High risk of bias
375.	Quera-Salva 2010	Low risk of bias	Low risk of bias	Stated but not tested	Stated but not tested	Unclear risk of bias	Low risk of bias	Low risk of bias
376.	Raft 1981	Unclear risk of bias	Unclear risk of bias	Stated but not tested	Stated but not tested	Unclear risk of bias	Low risk of bias	High risk of bias
377.	Rapaport 1996	Unclear risk of bias	Unclear risk of bias	Stated but not tested	Stated but not tested	Unclear risk of bias	Low risk of bias	Low risk of bias
378.	Rapaport 2003	Unclear risk of bias	Unclear risk of bias	Stated but not tested	Stated but not tested	Unclear risk of bias	Low risk of bias	Unclear risk of bias
379.	Rapaport 2009	Unclear risk of bias	Unclear risk of bias	Stated but not tested	Stated but not tested	Unclear risk of bias	Low risk of bias	Unclear risk of bias
380.	Raskin 2007	Unclear risk of bias	Unclear risk of bias	Stated but not tested	Stated but not tested	Unclear risk of bias	Low risk of bias	Unclear risk of bias
381.	Ravindran 1995	Unclear risk of bias	Unclear risk of bias	Stated but not tested	Stated but not tested	Unclear risk of bias	Unclear risk of bias	High risk of bias
382.	Ravindran 1997	Unclear risk of bias	Unclear risk of bias	Stated but not tested	Stated but not tested	Unclear risk of bias	Low risk of bias	Unclear risk of bias
383.	Reimherr 1990	Unclear risk of bias	Unclear risk of bias	Stated but not tested	Stated but not tested	Unclear risk of bias	Low risk of bias	Unclear risk of bias
384.	Reimherr 1998	Unclear risk of bias	Unclear risk of bias	Stated but not tested	Stated but not tested	Unclear risk of bias	Low risk of bias	Unclear risk of bias
385.	Remick 1994	Unclear risk of bias	Unclear risk of bias	Stated but not tested	Stated but not tested	Unclear risk of bias	Low risk of bias	High risk of bias
386.	Rickels 1982	Unclear risk of bias	Unclear risk of bias	Stated but not tested	Stated but not tested	Unclear risk of bias	Unclear risk of bias	Unclear risk of bias
387.	Rickels 1985	Unclear risk of bias	Unclear risk of bias	Stated but not tested	Stated but not tested	Unclear risk of bias	Low risk of bias	High risk of bias
388.	Rickels 1994	Unclear risk of bias	Unclear risk of bias	Stated but not tested	Stated but not tested	Unclear risk of bias	Low risk of bias	Unclear risk of bias
389.	Rickels 1995	Unclear risk of bias	Unclear risk of bias	Stated but not tested	Stated but not tested	Unclear risk of bias	Low risk of bias	Unclear risk of bias
390.	Rickels 2009	Unclear risk of bias	Unclear risk of bias	Stated but not tested	Stated but not tested	Unclear risk of bias	Low risk of bias	Unclear risk of bias
391.	Robinson 2014	Low risk of bias	Low risk of bias	Stated but not tested	Stated but not tested	Unclear risk of bias	Unclear risk of bias	Unclear risk of bias
392.	Roffman 1982	Unclear risk of bias	Unclear risk of bias	Stated but not tested	Stated but not tested	Unclear risk of bias	Low risk of bias	Unclear risk of bias
393.	Roose 2004	Low risk of bias	Unclear risk of bias	Stated but not tested	Stated but not tested	Unclear risk of bias	Low risk of bias	High risk of bias

394.	Ropert 1989	Unclear risk of bias	Unclear risk of bias	Stated but not tested	Stated but not tested	Unclear risk of bias	Low risk of bias	High risk of bias
395.	Rossini 2005	Low risk of bias	Unclear risk of bias	Stated but not tested	Stated but not tested	Stated but not tested	Low risk of bias	Low risk of bias
396.	Roth 1990	Unclear risk of bias	Unclear risk of bias	Stated but not tested	Stated but not tested	Unclear risk of bias	Unclear risk of bias	Unclear risk of bias
397.	Rouillon 1991	Unclear risk of bias	Unclear risk of bias	Stated but not tested	Stated but not tested	Unclear risk of bias	Low risk of bias	Unclear risk of bias
398.	Rowan 1980	Unclear risk of bias	Unclear risk of bias	Stated but not tested	Stated but not tested	Unclear risk of bias	Low risk of bias	Unclear risk of bias
399.	Rudolph 1998	Low risk of bias	Unclear risk of bias	Stated but not tested	Stated but not tested	Unclear risk of bias	Low risk of bias	Unclear risk of bias
400.	Rudolph 1999	Low risk of bias	Unclear risk of bias	Stated but not tested	Stated but not tested	Unclear risk of bias	Low risk of bias	Unclear risk of bias
401.	Rush 1998	Unclear risk of bias	Unclear risk of bias	Stated but not tested	Stated but not tested	Unclear risk of bias	High risk of bias	Unclear risk of bias
402.	Rush 2001	Unclear risk of bias	Unclear risk of bias	Stated but not tested	Stated but not tested	Unclear risk of bias	Unclear risk of bias	Unclear risk of bias
403.	Sacchetti 2002	Unclear risk of bias	Unclear risk of bias	Stated but not tested	Stated but not tested	Unclear risk of bias	Low risk of bias	Unclear risk of bias
404.	Sambunaris 2014	Low risk of bias	Unclear risk of bias	Stated but not tested	Stated but not tested	Stated but not tested	Low risk of bias	Unclear risk of bias
405.	Samuelian 1998	Unclear risk of bias	Unclear risk of bias	Stated but not tested	Stated but not tested	Unclear risk of bias	Low risk of bias	Unclear risk of bias
406.	Sauer 2003	Unclear risk of bias	Unclear risk of bias	Stated but not tested	Stated but not tested	Unclear risk of bias	Low risk of bias	Unclear risk of bias
407.	Schatzberg 2002	Low risk of bias	Unclear risk of bias	Low risk of bias	Stated but not tested	Unclear risk of bias	Low risk of bias	Unclear risk of bias
408.	Schatzberg 2006a	Unclear risk of bias	Unclear risk of bias	Stated but not tested	Stated but not tested	Unclear risk of bias	Low risk of bias	High risk of bias
409.	Schneider 2003	Low risk of bias	Low risk of bias	Stated but not tested	Stated but not tested	Unclear risk of bias	Low risk of bias	Unclear risk of bias
410.	Schoene 1993	Unclear risk of bias	Unclear risk of bias	Stated but not tested	Stated but not tested	Stated but not tested	Low risk of bias	Low risk of bias
411.	Schwartz 2002	Unclear risk of bias	Unclear risk of bias	Stated but not tested	Stated but not tested	Unclear risk of bias	Low risk of bias	Unclear risk of bias
412.	Schweizer 1994	Unclear risk of bias	Unclear risk of bias	Stated but not tested	Stated but not tested	Unclear risk of bias	Low risk of bias	High risk of bias
413.	SCT-MD-09	Unclear risk of bias	Unclear risk of bias	Stated but not tested	Stated but not tested	Unclear risk of bias	Unclear risk of bias	Low risk of bias
414.	SCT-MD-49	Unclear risk of bias	Unclear risk of bias	Stated but not tested	Stated but not tested	Stated but not tested	Low risk of bias	Unclear risk of bias
415.	Sechter 1999	Unclear risk of bias	Unclear risk of bias	Stated but not tested	Stated but not tested	Unclear risk of bias	Unclear risk of bias	Unclear risk of bias
416.	Sechter 2004	Unclear risk of bias	Unclear risk of bias	Stated but not tested	Stated but not tested	Unclear risk of bias	Low risk of bias	Unclear risk of bias
417.	SER 101	Unclear risk of bias	Unclear risk of bias	Stated but not tested	Stated but not tested	Unclear risk of bias	High risk of bias	High risk of bias
418.	SER 310	Unclear risk of bias	Unclear risk of bias	Stated but not tested	Stated but not tested	Unclear risk of bias	High risk of bias	Unclear risk of bias
419.	SER 315	Unclear risk of bias	Unclear risk of bias	Stated but not tested	Stated but not tested	Unclear risk of bias	Low risk of bias	High risk of bias
420.	SER-CHN-1	Unclear risk of bias	Unclear risk of bias	Stated but not tested	Stated but not tested	Unclear risk of bias	Low risk of bias	Low risk of bias
421.	Settle 1999	Unclear risk of bias	Unclear risk of bias	Stated but not tested	Stated but not tested	Unclear risk of bias	Low risk of bias	Unclear risk of bias
422.	Shaw 1986	Unclear risk of bias	Unclear risk of bias	Stated but not tested	Stated but not tested	Unclear risk of bias	Low risk of bias	High risk of bias
423.	Sheehan 2009a	Low risk of bias	Unclear risk of bias	Stated but not tested	Stated but not tested	Stated but not tested	Low risk of bias	Unclear risk of bias
424.	Sheehan 2009b	Unclear risk of bias	Unclear risk of bias	Stated but not tested	Stated but not tested	Unclear risk of bias	Low risk of bias	Unclear risk of bias

**425.**	**Shelton 2006**	Unclear risk of bias	Unclear risk of bias	Stated but not tested	Stated but not tested	Unclear risk of bias	Low risk of bias	Low risk of bias
**426.**	**Shipley 1981**	Unclear risk of bias	Unclear risk of bias	Stated but not tested	Stated but not tested	Unclear risk of bias	Unclear risk of bias	Unclear risk of bias
**427.**	**Shu 2014**	Low risk of bias	Low risk of bias	Stated but not tested	Stated but not tested	Unclear risk of bias	Low risk of bias	Unclear risk of bias
**428.**	**Silverstone 1999**	Unclear risk of bias	Unclear risk of bias	Stated but not tested	Stated but not tested	Unclear risk of bias	Low risk of bias	High risk of bias
**429.**	**Sir 2005**	Unclear risk of bias	Unclear risk of bias	Stated but not tested	Stated but not tested	Unclear risk of bias	Low risk of bias	High risk of bias
**430.**	**Smeraldi 1998**	Unclear risk of bias	Unclear risk of bias	Stated but not tested	Stated but not tested	Unclear risk of bias	Unclear risk of bias	Unclear risk of bias
**431.**	**Smith 1990**	Unclear risk of bias	Unclear risk of bias	Unclear risk of bias	Unclear risk of bias	Unclear risk of bias	Low risk of bias	High risk of bias
**432.**	**Sramek 1995**	Unclear risk of bias	Unclear risk of bias	Stated but not tested	Stated but not tested	Unclear risk of bias	Low risk of bias	Low risk of bias
**433.**	**Stahl 2000**	Unclear risk of bias	Unclear risk of bias	Stated but not tested	Stated but not tested	Stated but not tested	Low risk of bias	High risk of bias
**434.**	**Stahl 2010**	Unclear risk of bias	Unclear risk of bias	Stated but not tested	Stated but not tested	Unclear risk of bias	Low risk of bias	Unclear risk of bias
**435.**	**Staner 1995**	Unclear risk of bias	Unclear risk of bias	Stated but not tested	Stated but not tested	Unclear risk of bias	Low risk of bias	Unclear risk of bias
**436.**	**Stark 1985**	Unclear risk of bias	Unclear risk of bias	Stated but not tested	Stated but not tested	Unclear risk of bias	Low risk of bias	Unclear risk of bias
**437.**	**Stratas 1984**	Unclear risk of bias	Unclear risk of bias	Stated but not tested	Stated but not tested	Unclear risk of bias	High risk of bias	High risk of bias
**438.**	**Studie009**	Low risk of bias	Unclear risk of bias	Stated but not tested	Stated but not tested	Unclear risk of bias	Low risk of bias	Unclear risk of bias
**439.**	**Studie032**	Low risk of bias	Unclear risk of bias	Stated but not tested	Stated but not tested	Unclear risk of bias	Low risk of bias	Unclear risk of bias
**440.**	**Study 015**	Low risk of bias	Low risk of bias	Stated but not tested	Stated but not tested	Unclear risk of bias	Low risk of bias	Unclear risk of bias
**441.**	**Study 032a**	Low risk of bias	Unclear risk of bias	Stated but not tested	Stated but not tested	Unclear risk of bias	Low risk of bias	High risk of bias
**442.**	**Study 043**	Unclear risk of bias	Unclear risk of bias	Stated but not tested	Stated but not tested	Unclear risk of bias	Unclear risk of bias	Unclear risk of bias
**443.**	**Study 045**	Low risk of bias	Low risk of bias	Stated but not tested	Stated but not tested	Unclear risk of bias	Low risk of bias	High risk of bias
**444.**	**Study 049**	Unclear risk of bias	Low risk of bias	Stated but not tested	Stated but not tested	Unclear risk of bias	Low risk of bias	High risk of bias
**445.**	**Study 19**	Unclear risk of bias	Unclear risk of bias	Stated but not tested	Stated but not tested	Unclear risk of bias	Low risk of bias	Unclear risk of bias
**446.**	**Study 205**	Unclear risk of bias	Unclear risk of bias	Stated but not tested	Stated but not tested	Unclear risk of bias	Low risk of bias	High risk of bias
**447.**	**Study 25**	Unclear risk of bias	Unclear risk of bias	Stated but not tested	Stated but not tested	Unclear risk of bias	Low risk of bias	Unclear risk of bias
**448.**	**Study 62a**	Unclear risk of bias	Unclear risk of bias	Stated but not tested	Stated but not tested	Unclear risk of bias	Low risk of bias	High risk of bias
**449.**	**Study 62b**	Unclear risk of bias	Unclear risk of bias	Stated but not tested	Stated but not tested	Unclear risk of bias	Low risk of bias	High risk of bias
**450.**	**Study 89306**	Unclear risk of bias	Unclear risk of bias	Stated but not tested	Stated but not tested	Unclear risk of bias	Unclear risk of bias	Unclear risk of bias
**451.**	**Study HMAQ - Group B**	Unclear risk of bias	Unclear risk of bias	Stated but not tested	Stated but not tested	Unclear risk of bias	Unclear risk of bias	High risk of bias
**452.**	**Szegedi 2006**	Low risk of bias	Unclear risk of bias	Stated but not tested	Stated but not tested	Unclear risk of bias	Low risk of bias	Unclear risk of bias
**453.**	**Thase 1997**	Unclear risk of bias	Unclear risk of bias	Stated but not tested	Stated but not tested	Unclear risk of bias	Low risk of bias	High risk of bias
**454.**	**Thase 2006**	Unclear risk of bias	Unclear risk of bias	Stated but not tested	Stated but not tested	Unclear risk of bias	Low risk of bias	Unclear risk of bias
**455.**	**Thomson 1982**	Unclear risk of bias	Unclear risk of bias	Stated but not tested	Stated but not tested	Unclear risk of bias	Unclear risk of bias	High risk of bias

456.	Tignol 1993	Unclear risk of bias	Unclear risk of bias	Stated but not tested	Stated but not tested	Stated but not tested	Low risk of bias	Unclear risk of bias
457.	Timmerman 1993	Low risk of bias	Unclear risk of bias	Stated but not tested	Stated but not tested	Unclear risk of bias	Low risk of bias	Unclear risk of bias
458.	Tollefson 1995	Unclear risk of bias	Unclear risk of bias	Stated but not tested	Stated but not tested	Unclear risk of bias	Low risk of bias	Unclear risk of bias
459.	Tomarken 2004	Unclear risk of bias	Unclear risk of bias	Stated but not tested	Stated but not tested	Unclear risk of bias	Unclear risk of bias	Low risk of bias
460.	Tourian 2009	Low risk of bias	Low risk of bias	Stated but not tested	Stated but not tested	Unclear risk of bias	Low risk of bias	Unclear risk of bias
461.	Trivedi 2004	Unclear risk of bias	Unclear risk of bias	Stated but not tested	Stated but not tested	Unclear risk of bias	Low risk of bias	Unclear risk of bias
462.	Tsutsui 2000	Unclear risk of bias	Unclear risk of bias	Stated but not tested	Stated but not tested	Stated but not tested	Low risk of bias	Unclear risk of bias
463.	Tylee 1997	Unclear risk of bias	Unclear risk of bias	Stated but not tested	Stated but not tested	Unclear risk of bias	Low risk of bias	Unclear risk of bias
464.	Tzanakaki 2000	Unclear risk of bias	Unclear risk of bias	Stated but not tested	Stated but not tested	Unclear risk of bias	Low risk of bias	Unclear risk of bias
465.	Upward 1988	Unclear risk of bias	Unclear risk of bias	Stated but not tested	Stated but not tested	Unclear risk of bias	Unclear risk of bias	Low risk of bias
466.	Van de Merwe 1984	Low risk of bias	Low risk of bias	Stated but not tested	Stated but not tested	Unclear risk of bias	High risk of bias	Unclear risk of bias
467.	vanMoffaert 1995a	Unclear risk of bias	Unclear risk of bias	Stated but not tested	Stated but not tested	Unclear risk of bias	Unclear risk of bias	Low risk of bias
468.	vanMoffaert 1995b	Unclear risk of bias	Unclear risk of bias	Stated but not tested	Stated but not tested	Unclear risk of bias	Low risk of bias	Unclear risk of bias
469.	Vartiainen 1994	Unclear risk of bias	Unclear risk of bias	Stated but not tested	Stated but not tested	Unclear risk of bias	Low risk of bias	Unclear risk of bias
470.	VEN 600A-303	Unclear risk of bias	Unclear risk of bias	Stated but not tested	Stated but not tested	Unclear risk of bias	Low risk of bias	High risk of bias
471.	VEN 600A-313	Unclear risk of bias	Unclear risk of bias	Stated but not tested	Stated but not tested	Unclear risk of bias	Low risk of bias	Unclear risk of bias
472.	VEN XR 367	Unclear risk of bias	Unclear risk of bias	Stated but not tested	Stated but not tested	Unclear risk of bias	Low risk of bias	High risk of bias
473.	Ventura 2007	Unclear risk of bias	Unclear risk of bias	Stated but not tested	Stated but not tested	Unclear risk of bias	Low risk of bias	Low risk of bias
474.	Versiani 1999	Unclear risk of bias	Unclear risk of bias	Stated but not tested	Stated but not tested	Unclear risk of bias	Low risk of bias	Low risk of bias
475.	Versiani 2000	Unclear risk of bias	Unclear risk of bias	Stated but not tested	Stated but not tested	Unclear risk of bias	Low risk of bias	High risk of bias
476.	Versiani 2005	Unclear risk of bias	Unclear risk of bias	Stated but not tested	Stated but not tested	Stated but not tested	Low risk of bias	Low risk of bias
477.	Wade 2002	Unclear risk of bias	Unclear risk of bias	Stated but not tested	Stated but not tested	Unclear risk of bias	Low risk of bias	Low risk of bias
478.	Wade 2003	Unclear risk of bias	Low risk of bias	Stated but not tested	Stated but not tested	Stated but not tested	Unclear risk of bias	Unclear risk of bias
479.	Wade 2007	Low risk of bias	Low risk of bias	Stated but not tested	Stated but not tested	Unclear risk of bias	Low risk of bias	Unclear risk of bias
480.	Walczak 1996	Unclear risk of bias	Unclear risk of bias	Stated but not tested	Stated but not tested	Unclear risk of bias	Unclear risk of bias	High risk of bias
481.	Wang 2014	Low risk of bias	Unclear risk of bias	Stated but not tested	Stated but not tested	Unclear risk of bias	Low risk of bias	Unclear risk of bias
482.	Wang 2015	Low risk of bias	Low risk of bias	Stated but not tested	Stated but not tested	Stated but not tested	Low risk of bias	Unclear risk of bias
483.	Weisler 1994	Unclear risk of bias	Unclear risk of bias	Stated but not tested	Stated but not tested	Unclear risk of bias	Low risk of bias	Unclear risk of bias
484.	WELL 029	Unclear risk of bias	Unclear risk of bias	Stated but not tested	Stated but not tested	Unclear risk of bias	Unclear risk of bias	Unclear risk of bias
485.	WELL AK140016	Unclear risk of bias	Unclear risk of bias	Stated but not tested	Stated but not tested	Unclear risk of bias	Low risk of bias	Unclear risk of bias
486.	WELL AK1A4006	Unclear risk of bias	Unclear risk of bias	Stated but not tested	Stated but not tested	Unclear risk of bias	Low risk of bias	Unclear risk of bias

487.	Wellbutrin 06	Unclear risk of bias	Unclear risk of bias	Stated but not tested	Stated but not tested	Unclear risk of bias	Low risk of bias	Low risk of bias
488.	Wellbutrin 25	Unclear risk of bias	Unclear risk of bias	Stated but not tested	Stated but not tested	Unclear risk of bias	Low risk of bias	High risk of bias
489.	Wheatley 1998	Low risk of bias	Low risk of bias	Stated but not tested	Stated but not tested	Stated but not tested	Low risk of bias	Unclear risk of bias
490.	Wilcox 1994	Unclear risk of bias	Unclear risk of bias	Stated but not tested	Stated but not tested	Unclear risk of bias	Low risk of bias	High risk of bias
491.	Winokur 2003	Unclear risk of bias	Unclear risk of bias	Stated but not tested	Stated but not tested	Stated but not tested	Low risk of bias	Low risk of bias
492.	Yang 2003	Unclear risk of bias	Unclear risk of bias	Stated but not tested	Stated but not tested	Unclear risk of bias	Unclear risk of bias	High risk of bias
493.	Yevtushenko 2007	Unclear risk of bias	Unclear risk of bias	Stated but not tested	Stated but not tested	Stated but not tested	Unclear risk of bias	Low risk of bias
494.	Young 1987	Unclear risk of bias	Unclear risk of bias	Stated but not tested	Stated but not tested	Unclear risk of bias	Unclear risk of bias	Unclear risk of bias
495.	Zajecka 2010	Low risk of bias	Low risk of bias	Stated but not tested	Stated but not tested	Unclear risk of bias	Low risk of bias	Unclear risk of bias
496.	Zhang 2014	Low risk of bias	Low risk of bias	Stated but not tested	Stated but not tested	Unclear risk of bias	Low risk of bias	Unclear risk of bias
497.	Zivkov 1995	Low risk of bias	Unclear risk of bias	Stated but not tested	Stated but not tested	Unclear risk of bias	Low risk of bias	Unclear risk of bias
498.	845	Unclear risk of bias	Unclear risk of bias	Stated but not tested	Stated but not tested	Unclear risk of bias	Low risk of bias	Unclear risk of bias
499.	003-008	Unclear risk of bias	Unclear risk of bias	Stated but not tested	Stated but not tested	Stated but not tested	Low risk of bias	High risk of bias
500.	003-042	Unclear risk of bias	Unclear risk of bias	Stated but not tested	Stated but not tested	Stated but not tested	Low risk of bias	High risk of bias
501.	003-048	Unclear risk of bias	Unclear risk of bias	Stated but not tested	Stated but not tested	Stated but not tested	Low risk of bias	High risk of bias
502.	030A2-0004/030A2-0005	Unclear risk of bias	Unclear risk of bias	Stated but not tested	Stated but not tested	Unclear risk of bias	High risk of bias	Unclear risk of bias
503.	03A0A-004A	Unclear risk of bias	Unclear risk of bias	Stated but not tested	Stated but not tested	Unclear risk of bias	Low risk of bias	Unclear risk of bias
504.	0600A1-300	Unclear risk of bias	Unclear risk of bias	Unclear risk of bias	Unclear risk of bias	Unclear risk of bias	Unclear risk of bias	Unclear risk of bias
505.	0600A1-372	Unclear risk of bias	Unclear risk of bias	Stated but not tested	Stated but not tested	Unclear risk of bias	Low risk of bias	Unclear risk of bias
506.	0600A-326	Low risk of bias	Unclear risk of bias	Stated but not tested	Unclear risk of bias	Unclear risk of bias	Low risk of bias	High risk of bias
507.	0600A-332	Unclear risk of bias	Unclear risk of bias	Stated but not tested	Stated but not tested	Unclear risk of bias	Low risk of bias	Unclear risk of bias
508.	0600A-347	Unclear risk of bias	Unclear risk of bias	Stated but not tested	Stated but not tested	Unclear risk of bias	Unclear risk of bias	High risk of bias
509.	0600A-349	Unclear risk of bias	Unclear risk of bias	Stated but not tested	Stated but not tested	Unclear risk of bias	Unclear risk of bias	Unclear risk of bias
510.	0600A-626	Unclear risk of bias	Unclear risk of bias	Stated but not tested	Stated but not tested	Unclear risk of bias	Low risk of bias	High risk of bias
511.	0600A-654	Unclear risk of bias	Unclear risk of bias	Stated but not tested	Stated but not tested	Unclear risk of bias	Unclear risk of bias	High risk of bias
512.	0600B1-367	Unclear risk of bias	Unclear risk of bias	Stated but not tested	Stated but not tested	Unclear risk of bias	Low risk of bias	Unclear risk of bias
513.	0600B1-384	Unclear risk of bias	Unclear risk of bias	Stated but not tested	Stated but not tested	Unclear risk of bias	Low risk of bias	Unclear risk of bias
514.	0600B1-402	Unclear risk of bias	Unclear risk of bias	Stated but not tested	Stated but not tested	Unclear risk of bias	Low risk of bias	Unclear risk of bias
515.	244 (EMD 68 843-009)	Unclear risk of bias	Unclear risk of bias	Stated but not tested	Stated but not tested	Unclear risk of bias	Unclear risk of bias	Unclear risk of bias
516.	245 (EMD 68 843-010)	Unclear risk of bias	Unclear risk of bias	Stated but not tested	Stated but not tested	Unclear risk of bias	Unclear risk of bias	Unclear risk of bias
517.	246 (SB 659746-003)	Unclear risk of bias	Unclear risk of bias	Stated but not tested	Stated but not tested	Unclear risk of bias	Unclear risk of bias	Unclear risk of bias

**518.**	**247 (SB 659746-014)**	Unclear risk of bias	Unclear risk of bias	Stated but not tested	Stated but not tested	Unclear risk of bias	Low risk of bias	Unclear risk of bias
**519.**	**248 (SB 659746-002)**	Unclear risk of bias	Unclear risk of bias	Stated but not tested	Stated but not tested	Unclear risk of bias	Low risk of bias	High risk of bias
**520.**	**29060 07 001**	Unclear risk of bias	Unclear risk of bias	Stated but not tested	Stated but not tested	Unclear risk of bias	Low risk of bias	High risk of bias
**521.**	**29060/299**	Unclear risk of bias	Unclear risk of bias	Stated but not tested	Stated but not tested	Unclear risk of bias	Low risk of bias	High risk of bias
**522.**	**29060/356**	Unclear risk of bias	Unclear risk of bias	Stated but not tested	Stated but not tested	Unclear risk of bias	Low risk of bias	Unclear risk of bias

4 *Definition of covariates*

4.1 *Sponsorship*

We rated each arm of a study as being "sponsored" when it was indicated anywhere in the text that the study was funded/sponsored by the company which manufactured or marketed the drug in question, or if one or more of the authors were affiliated with the company in question, or if the data came from the documents provided by or obtained from the company website. We rated the sponsorship as "Unclear" if the authors only listed the names of the companies in question in their declaration of conflicts of interest.
When one of the arms was "sponsored" thus defined, we regarded that the study was sponsored.
Sponsorship bias was considered at the arm level. Arms in trials sponsored by an academic, governmental or non-for-profit organization were considered to be at low risk of bias. In trials that received finding by industry we considered the arm examining the drug manufactured by the company to be at high risk of bias whereas the comparator arm not associated with the company to be at low risk of bias. Arms in studies with unclear sponsoring were considered at high risk of bias.

4.2 *Baseline severity*

To make the mean baseline severity scores of participants comparable across studies that used different rating scales we used the transformation tables provided online at http://www.ids-qids.org/index2.html - table1 (tables 3, 4). We converted all values into HAMD17 scale. We excluded studies that used HAMD29, HAMD31 or other scales that we could not transform into HAMD17 using this online tool. We included studies that reported the use of a HAMD scale without specifying which HAMD scale was used by assuming that such studies used the HAMD17 scale.

4.3 *Novelty of the agent*

In each study we rate the drug of interest as "novel" in comparison with placebo or with an older standard drug. The "oldness" of the drug was judged according to the region where the study was conducted because when and whether a drug was marked would be different from region to region in the world. When such information was unclear, we rated novelty of the agent as "Unclear."

4.4 *Licensed and accepted dose ranges*

	Licensed dose range: dosage approved by FDA and EMA (if the drug was not licensed in USA or Europe, we used BNF) (mg/day)	Accepted dose range: Range accepted/ recommended in main clinical guidelines, including starting dose by FDA (mg/day)		
		Accepted range	*Accepted but lower than licensed range*	*Accepted but higher than licensed range*
Agomelatine	25-50	25-50	-	-
Amitriptyline	75-200	50a-300	50-74	201-300

Bupropion	300–450	200a-450	200-299	-
Citalopram	20–40	20-60	-	41-60
Clomipramine	30-250	10-300	10-29	251-300
Desvenlafaxine	50-100	50-100	-	-
Duloxetine	40-120	30-120	*30-39*	-
Escitalopram	10-20	10-30	-	*21-30*
Fluoxetine	20-80	10-80	*10-19*	-
Fluvoxamine	50-300	50-300	-	-
Levomilnacipran	40-120	20a-120	*20-39*	-
Milnacipran	50-200	50-200	-	-
Mirtazapine	15-45	15-45	-	-
Nefazodone	300-600	150-600	*150-299*	-
Paroxetine	20-50	20-60	-	*51-60*
Reboxetine	8-12	4-12	*4-7*	-
Sertraline	50-200	50-200	-	-
Trazodone	150-400	150-600	-	*401-600*
Venlafaxine	75-375	75-375	-	-
Vilazodone	20-40	10a-40	*10-19*	-
Vortioxetine	5-20	5-20	-	-

a: Starting dose in FDA, which should usually be increased

4.5 Unpublished data

For all studies we sought information about the response to treatment from published and unpublished data. The flowchart below presents the followed hierarchy when extracting response rates from published and unpublished reports of trials.

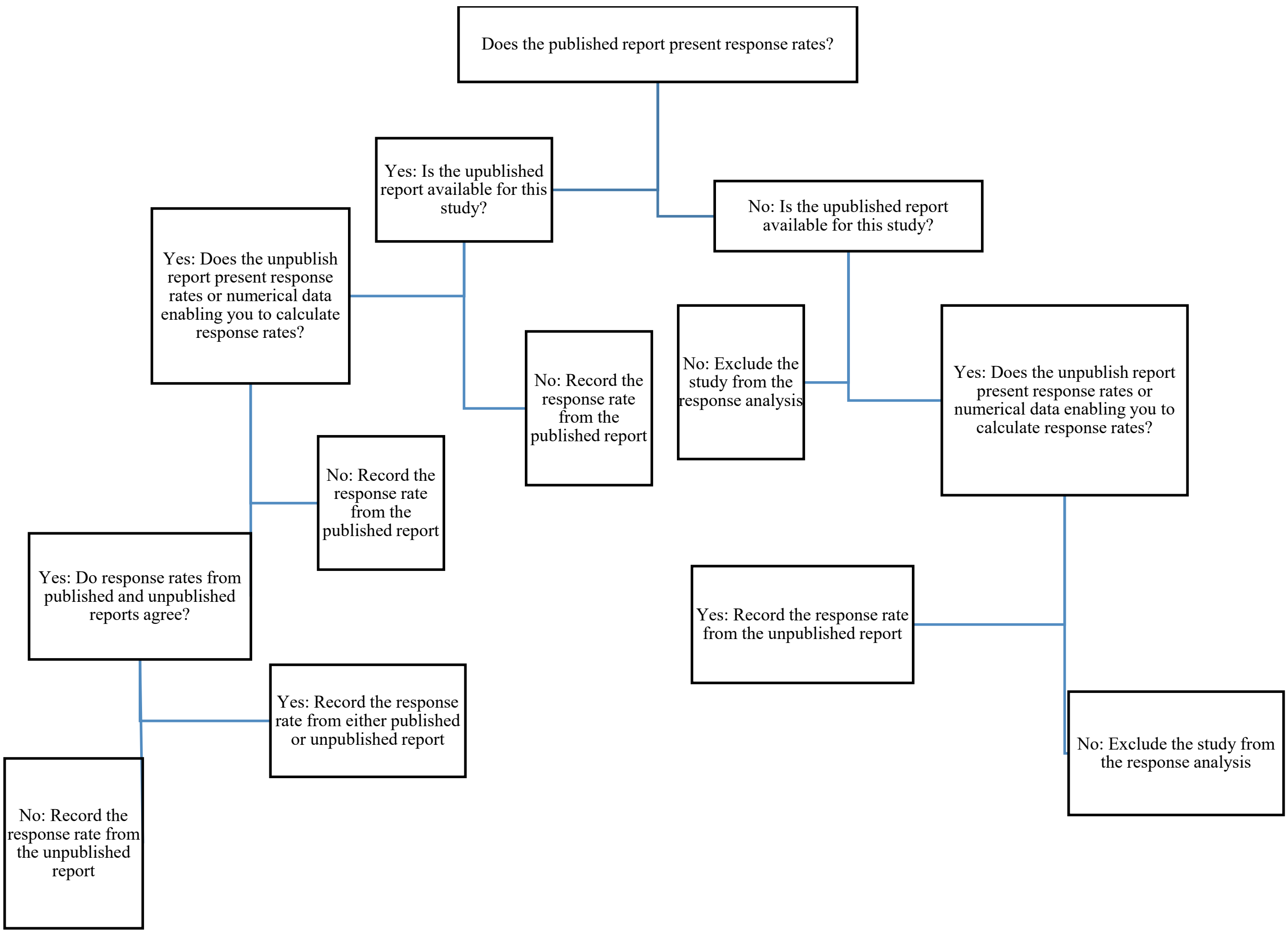

Studies can be classified into the following 6 categories. These categories are grouped into forming the variables "Response rate presented in published report" and "Unpublished report available and presents adequate response data" as shown in the following table.

**Trial category**	Number of trials (arms)	Variable "Response rate presented in published report"	Variable "Unpublished report available and presents adequate response data"
No response rates were reported in in the published or the unpublished study reports	35 (56)	*Excluded from the analysis of response*	NO
No response data presented in the published report. Response rate is presented in the unpublished report (and recorded).	73 (123)	NO	YES

<table>
<tr><td>The published report presents some information about response but the numerical data reported is not sufficient to estimate response rates. Response rate is recorded from unpublished reports.</td><td>22 (43)</td><td></td><td rowspan="2"></td></tr>
<tr><td>Response rates are presented both in published and unpublished reports but figures do not match. We recorded response rates from the unpublished reports.</td><td>26 (36)</td><td rowspan="4">YES</td></tr>
<tr><td>Response rates imputed from other relevant information presented in the published reports (e.g. from continuous outcomes). There is no unpublished study report or the unpublished report presents insufficient data to calculate response rates.</td><td>51 (78)</td><td rowspan="2">NO</td></tr>
<tr><td>Response rates are presented in published report. There is no unpublished study report or the unpublished report presents insufficient data to calculate response rates.</td><td>164 (277)</td></tr>
<tr><td>Response rates are presented both in published and unpublished reports and figures do match.</td><td>50 (93)</td><td>YES</td></tr>
</table>

4.6 Risk of bias in the included studies

Two independent reviewers assessed RoB of each study for the primary outcome of efficacy in the following domains: sequence generation, allocation concealment, blinding of participant, blinding of therapist, blinding of assessor, selective reporting and attrition.

Then we summatively rated the overall RoB of each study as follows : Studies were classified as having low risk of bias if none of the domains above was rated as high risk of bias and three or less were rated as unclear risk; moderate if one was rated as high risk of bias or none was rated as high risk of bias but four or more were rated as unclear risk, and all other cases were assumed to pertain to high risk of bias.

5 *Heterogeneity parameter standard deviations estimated in various models*

We present heterogeneity τ with 95% credible intervals (CrI).

5.1 Primary outcomes

5.1.1 Response

Network τ (from NMA in all trials, n=433)= 0.21 (0.17,0.25)

Network τ (from NMA in head-to-head trials, n=180)= 0.16 (0.04,0.25)

Placebo-controlled trials common τ (from pairwise meta-analyses sharing the same τ)= 0.22 (0.17,0.27)

Head-to-head trials common τ (from pairwise meta-analyses sharing the same τ)= 0.17 (0.08,0.25)

5.1.2 Dropouts for any reason

Network τ (from NMA in all trials, n=423)= 0.20 (0.15,0.25)

Network τ (from NMA in in head-to-head trials, n=174)= 0.11 (0.00,0.23)

Placebo-controlled trials common τ (from pairwise meta-analyses sharing the same τ)= 0.22 (0.17,0.28)

Head-to-head trials common τ (from pairwise meta-analyses sharing the same τ)= 0.09 (0.01,0.20)

5.2 Secondary outcomes

5.2.1 Efficacy continuous

Network τ (from NMA in all trials, n=391)= 0.12 (0.10,0.14)

Network τ (from NMA in head-to-head trials, n=155)= 0.08 (0.03,0.13)

Placebo-controlled trials common τ (from pairwise meta-analyses sharing the same τ)= 0.14 (0.11,0.16)

Head-to-head trials common τ (from pairwise meta-analyses sharing the same τ)= 0.08 (0.04,0.12)

5.2.2 Remission

Network τ (from NMA in all trials, n=417)= 0.19 (0.15,0.24)

Network τ (from NMA in head-to-head trials, n=172)=0.18 (0.06,0.28)

Placebo-controlled trials common τ (from pairwise meta-analyses sharing the same τ)= 0.19 (0.13,0.25)

Head-to-head trials common τ (from pairwise meta-analyses sharing the same τ)= 0.20 (0.12,0.28)

5.2.3 Dropouts due to adverse events

Network τ (from NMA in all trials, n=395)= 0.32 (0.23,0.40)

Network τ (from NMA in in head-to-head trials, n=154)= 0.31 (0.10,0.48)

Placebo-controlled trials common τ (from pairwise meta-analyses sharing the same τ)= 0.28 (0.14,0.39)

Head-to-head trials common τ (from pairwise meta-analyses sharing the same τ)= 0.19 (0.03,0.35)

6 *Estimation of inconsistency: summary of results*

Outcome and Data	Number of studies	Number of inconsistent loops out of total (loop-specific method)	Percentage of the inconsistent loops	Number of inconsistent comparisons out of total (SIDE splitting)	Percentage of the inconsistent comparisons	p-value of the Design-by-Treatment test
**Response all trials**	432	17/219	8%	8/93	9%	0.0631
**Response head-to-head**	179	11/140	8%	5/69	7%	0.3182
**Dropout for any reason all trials**	422	16/210	8%	4/91	4%	0.2190
**Dropout for any reason head-to-head trials**	173	11/122	9%	4/66	6%	0.0544
**Efficacy continuous all trials**	390	13/200	7%	5/90	6%	0.7872
**Efficacy continuous head-to-head trials**	154	6/115	5%	3/64	5%	0.8145
**Remission all trials**	416	13/206	6%	9/91	10%	0.4655
**Remission head-to-head trials**	171	9/119	8%	6/65	9%	0.0652
**Dropout due to AE all trials**	394	16/204	8%	7/90	8%	0.0008
**Dropout due to AE head-to-head**	155	10/113	9%	9/63	14%	0.0034

7 *Analyses of the all trials network (including placebo arms)*

7.1 *Results from pairwise meta-analyses*

In the following pairwise meta-analyses, we assumed two heterogeneity variance parameters: one for placebo-controlled trials and one for head-to-head trials.

Drugs are reported in alphabetical order. Results are the ORs (with 95% CrI) in the column-defining treatment compared with the row-defining treatment. For efficacy, ORs higher than 1 favour the column-defining treatment (ie, the first in alphabetical order). For acceptability, ORs lower than 1 favour the first drug in alphabetical order. To obtain ORs for comparisons in the opposite direction, reciprocals should be taken.

7.1.1 Response

**Agom**	-	-	-	-	-	1.32 (0.77, 2.25)	0.81 (0.49, 1.33)	0.98 (0.76, 1.28)	-	-	-	-	-	1.17 (0.91, 1.51)	-	-	-	0.73 (0.47, 1.15)	-	-	0.64 (0.53, 0.76)
-	**Amit**	-	-	-	-	-	-	0.93 (0.68, 1.28)	0.92 (0.56, 1.50)	-	0.85 (0.46, 1.55)	0.95 (0.61, 1.49)	-	0.93 (0.74, 1.18)	-	0.77 (0.60, 0.99)	0.78 (0.49, 1.27)	0.84 (0.48, 1.49)	-	-	0.37 (0.31, 0.45)
-	-	**Bupr**	-	-	-	-	1.07 (0.70, 1.62)	1.20 (0.83, 1.71)	-	-	-	-	-	1.00 (0.47, 2.12)	-	1.13 (0.12, 9.74)	0.47 (0.21, 1.05)	0.85 (0.49, 1.47)	-	-	0.67 (0.56, 0.81)
-	-	-	**Cita**	1.78 (0.79, 4.08)	-	-	1.35 (1.09, 1.67)	1.04 (0.69, 1.56)	0.91 (0.46, 1.78)	-	-	0.74 (0.34, 1.63)	-	0.74 (0.41, 1.33)	0.58 (0.34, 0.99)	1.18 (0.74, 1.89)	-	0.55 (0.29, 1.06)	1.11 (0.76, 1.62)	-	0.66 (0.54, 0.81)
-	-	-	0.56 (0.25, 1.26)	**Clom**	-	-	-	1.65 (1.01, 2.70)	0.53 (0.18, 1.49)	-	1.17 (0.50, 2.75)	-	-	1.10 (0.82, 1.48)	-	1.05 (0.61, 1.82)	0.62 (0.26, 1.43)	1.68 (0.90, 3.12)	-	-	-
-	-	-	-	-	**Desv**	1.21 (0.72, 2.02)	-	-	-	-	-	-	-	-	-	-	-	-	-	-	0.68 (0.56, 0.82)
0.76 (0.44, 1.30)	-	-	-	-	0.83 (0.49, 1.39)	**Dulo**	1.18 (0.86, 1.62)	0.81 (0.43, 1.51)	-	-	-	-	-	1.02 (0.82, 1.28)	-	-	-	1.11 (0.77, 1.61)	-	0.72 (0.58, 0.90)	0.52 (0.45, 0.60)
1.24 (0.75, 2.03)	-	0.94 (0.62, 1.42)	0.74 (0.60, 0.92)	-	-	0.85 (0.62, 1.16)	**Esci**	0.88 (0.61, 1.27)	-	-	-	-	-	1.00 (0.73, 1.38)	-	1.17 (0.77, 1.80)	-	0.83 (0.52, 1.31)	-	-	0.69 (0.60, 0.80)
1.02 (0.78, 1.32)	1.07 (0.78, 1.46)	0.84 (0.59, 1.20)	0.97 (0.64, 1.45)	0.60 (0.37, 0.99)	-	1.24 (0.66, 2.31)	1.13 (0.79, 1.63)	**Fluo**	1.03 (0.61, 1.76)	-	0.85 (0.54, 1.32)	1.34 (0.99, 1.84)	1.04 (0.53, 2.01)	1.05 (0.86, 1.28)	0.81 (0.58, 1.15)	1.43 (1.08, 1.89)	1.22 (0.69, 2.11)	1.24 (1.04, 1.50)	-	-	0.70 (0.62, 0.79)
-	1.09 (0.67, 1.79)	-	1.10 (0.56, 2.18)	1.90 (0.67, 5.69)	-	-	-	0.97 (0.57, 1.65)	**Fluv**	-	1.72 (0.96, 3.15)	1.14 (0.71, 1.82)	-	1.14 (0.60, 2.17)	-	0.63 (0.33, 1.20)	-	2.40 (0.98, 5.97)	-	-	0.48 (0.36, 0.65)
-	-	-	-	-	-	-	-	-	-	**Levo**	-	-	-	-	-	-	-	-	-	-	0.63 (0.49, 0.81)
-	1.18 (0.65, 2.15)	-	-	0.86 (0.36, 2.02)	-	-	-	1.18 (0.76, 1.84)	0.58 (0.32, 1.04)	-	**Miln**	-	-	1.05 (0.74, 1.49)	-	0.46 (0.05, 2.80)	-	-	-	-	-
-	1.05 (0.67, 1.64)	-	1.34 (0.61, 2.97)	-	-	-	-	0.75 (0.54, 1.01)	0.88 (0.55, 1.41)	-	-	**Mirt**	-	1.03 (0.76, 1.41)	-	1.03 (0.58, 1.81)	0.66 (0.39, 1.12)	0.73 (0.48, 1.12)	-	-	0.55 (0.43, 0.71)
-	-	-	-	-	-	-	-	0.96 (0.50, 1.88)	-	-	-	-	**Nefa**	1.33 (0.75, 2.43)	-	0.86 (0.42, 1.74)	-	-	-	-	0.57 (0.42, 0.77)
0.85 (0.66, 1.10)	1.07 (0.84, 1.35)	1.00 (0.47, 2.12)	1.35 (0.75, 2.45)	0.91 (0.68, 1.22)	-	0.98 (0.78, 1.22)	1.00 (0.73, 1.36)	0.96 (0.78, 1.16)	0.88 (0.46, 1.68)	-	0.95 (0.67, 1.35)	0.97 (0.71, 1.32)	0.75 (0.41, 1.34)	**Paro**	0.81 (0.60, 1.08)	1.11 (0.72, 1.72)	0.58 (0.32, 1.02)	1.58 (1.09, 2.31)	-	-	0.60 (0.53, 0.67)
-	-	-	1.74 (1.01, 2.98)	-	-	-	-	1.23 (0.87, 1.72)	-	-	-	-	-	1.24 (0.92, 1.66)	**Rebo**	-	-	0.95 (0.47, 1.92)	-	-	0.70 (0.56, 0.87)
-	1.29 (1.01, 1.66)	0.88 (0.10, 8.13)	0.84 (0.53, 1.35)	0.95 (0.55, 1.65)	-	-	0.85 (0.56, 1.31)	0.70 (0.53, 0.92)	1.58 (0.84, 3.01)	-	2.18 (0.36, 19.73)	0.98 (0.55, 1.73)	1.16 (0.58, 2.37)	0.90 (0.58, 1.38)	-	**Sert**	1.82 (0.79, 4.27)	1.18 (0.77, 1.81)	-	-	0.62 (0.53, 0.74)
-	1.28 (0.79, 2.06)	2.11 (0.95, 4.73)	-	1.62 (0.70, 3.79)	-	-	-	0.82 (0.47, 1.44)	-	-	-	1.51 (0.89, 2.57)	-	1.73 (0.98, 3.08)	-	0.55 (0.23, 1.27)	**Traz**	1.58 (0.91, 2.79)	-	-	0.56 (0.41, 0.76)
1.36 (0.87, 2.15)	1.19 (0.67, 2.09)	1.17 (0.68, 2.02)	1.81 (0.94, 3.48)	0.60 (0.32, 1.11)	-	0.90 (0.62, 1.30)	1.21 (0.77, 1.92)	0.81 (0.67, 0.97)	0.42 (0.17, 1.02)	-	-	1.37 (0.89, 2.09)	-	0.63 (0.43, 0.92)	1.05 (0.52, 2.12)	0.84 (0.55, 1.30)	0.63 (0.36, 1.10)	**Venl**	-	1.19 (0.80, 1.76)	0.56 (0.48, 0.65)
-	-	-	0.90 (0.62, 1.31)	-	-	-	-	-	-	-	-	-	-	-	-	-	-	-	**Vila**	-	0.64 (0.50, 0.81)
-	-	-	-	-	-	1.38 (1.11, 1.72)	-	-	-	-	-	-	-	-	-	-	-	0.84 (0.57, 1.25)	-	**Vort**	0.57 (0.49, 0.67)
1.57 (1.31, 1.87)	2.69 (2.22, 3.26)	1.49 (1.23, 1.80)	1.51 (1.24, 1.84)	-	1.48 (1.21, 1.80)	1.93 (1.68, 2.23)	1.44 (1.25, 1.67)	1.43 (1.26, 1.61)	2.07 (1.53, 2.78)	1.59 (1.23, 2.06)	-	1.82 (1.41, 2.35)	1.75 (1.29, 2.37)	1.67 (1.49, 1.87)	1.43 (1.15, 1.79)	1.60 (1.35, 1.90)	1.79 (1.32, 2.41)	1.79 (1.54, 2.08)	1.57 (1.23, 2.00)	1.75 (1.49, 2.05)	**Plac**

7.1.2 Dropouts for any reason

**Agom**	-	-	-	-	-	0.86 (0.49, 1.50)	1.29 (0.71, 2.35)	1.04 (0.80, 1.35)	-	-	-	-	-	1.01 (0.77, 1.31)	-	-	-	2.06 (1.30, 3.42)	-	-	1.16 (0.94, 1.43)
-	**Amit**	1.88 (0.71, 6.25)	-	-	-	-	-	0.63 (0.45, 0.87)	0.74 (0.47, 1.19)	-	0.99 (0.48, 1.98)	1.17 (0.76, 1.84)	-	1.00 (0.78, 1.26)	-	0.93 (0.73, 1.20)	1.01 (0.58, 1.74)	1.00 (0.54, 1.88)	-	-	1.33 (1.08, 1.63)
-	0.53 (0.16, 1.40)	**Bupr**	-	-	-	-	1.07 (0.71, 1.62)	0.99 (0.72, 1.38)	-	-	-	-	-	1.37 (0.60, 3.05)	-	-	0.92 (0.44, 1.92)	1.15 (0.72, 1.87)	-	-	0.99 (0.81, 1.23)
-	-	-	**Cita**	0.62 (0.21, 1.61)	-	-	0.88 (0.67, 1.16)	0.87 (0.58, 1.30)	1.45 (0.74, 2.89)	-	-	2.44 (1.01, 6.12)	-	1.10 (0.59, 2.01)	1.60 (0.92, 2.83)	1.61 (0.97, 2.68)	-	10.11 (2.19, 88.50)	1.21 (0.80, 1.82)	-	0.95 (0.75, 1.21)
-	-	-	1.61 (0.62, 4.77)	**Clom**	-	-	-	0.65 (0.36, 1.17)	2.04 (0.40, 11.26)	-	-	-	-	0.65 (0.49, 0.85)	-	0.54 (0.28, 1.07)	-	0.96 (0.41, 2.35)	-	-	2.31 (0.42, 14.70)
-	-	-	-	-	**Desv**	0.87 (0.53, 1.36)	-	-	-	-	-	-	-	-	-	-	-	-	-	-	0.94 (0.75, 1.17)
1.16 (0.67, 2.05)	-	-	-	-	1.15 (0.74, 1.87)	**Dulo**	0.62 (0.46, 0.84)	1.23 (0.66, 2.25)	-	-	-	-	-	0.99 (0.79, 1.24)	-	-	-	0.64 (0.44, 0.94)	-	0.96 (0.76, 1.20)	0.93 (0.80, 1.10)
0.78 (0.43, 1.40)	-	0.93 (0.62, 1.40)	1.13 (0.86, 1.49)	-	-	1.60 (1.19, 2.17)	**Esci**	1.13 (0.78, 1.61)	-	-	-	-	-	1.16 (0.79, 1.71)	-	0.92 (0.61, 1.45)	-	1.12 (0.71, 1.76)	-	-	1.01 (0.85, 1.19)
0.97 (0.74, 1.26)	1.59 (1.15, 2.23)	1.01 (0.73, 1.38)	1.15 (0.77, 1.74)	1.55 (0.85, 2.74)	-	0.81 (0.44, 1.50)	0.89 (0.62, 1.28)	**Fluo**	1.18 (0.63, 2.12)	-	1.08 (0.71, 1.65)	1.39 (1.01, 1.91)	2.00 (0.81, 5.03)	1.05 (0.87, 1.26)	1.39 (0.99, 1.92)	0.86 (0.61, 1.16)	1.55 (0.88, 2.74)	1.14 (0.94, 1.38)	-	-	1.16 (1.01, 1.33)
-	1.36 (0.84, 2.12)	-	0.69 (0.35, 1.34)	0.49 (0.09, 2.49)	-	-	-	0.85 (0.47, 1.59)	**Fluv**	-	0.77 (0.36, 1.63)	1.17 (0.71, 1.94)	-	0.74 (0.39, 1.40)	-	0.44 (0.17, 1.05)	-	0.48 (0.20, 1.19)	-	-	0.92 (0.68, 1.24)
-	-	-	-	-	-	-	-	-	-	**Levo**	-	-	-	-	-	-	-	-	-	-	0.84 (0.65, 1.09)
-	1.01 (0.50, 2.10)	-	-	-	-	-	-	0.93 (0.61, 1.41)	1.30 (0.61, 2.81)	-	**Miln**	-	-	1.01 (0.72, 1.40)	-	0.60 (0.19, 1.71)	-	-	-	-	-
-	0.85 (0.54, 1.31)	-	0.41 (0.16, 0.99)	-	-	-	-	0.72 (0.52, 0.99)	0.86 (0.51, 1.40)	-	-	**Mirt**	-	1.20 (0.86, 1.68)	-	0.77 (0.43, 1.34)	1.10 (0.64, 1.95)	1.37 (0.93, 2.05)	-	-	1.10 (0.85, 1.41)
-	-	-	-	-	-	-	-	0.50 (0.20, 1.23)	-	-	-	-	**Nefa**	0.95 (0.52, 1.74)	-	0.98 (0.48, 2.10)	-	-	-	-	1.19 (0.87, 1.61)
0.99 (0.76, 1.29)	1.00 (0.80, 1.29)	0.73 (0.33, 1.67)	0.91 (0.50, 1.71)	1.54 (1.17, 2.04)	-	1.01 (0.81, 1.27)	0.86 (0.59, 1.26)	0.95 (0.79, 1.15)	1.35 (0.71, 2.59)	-	0.99 (0.71, 1.38)	0.84 (0.59, 1.17)	1.05 (0.58, 1.92)	**Paro**	1.24 (0.95, 1.63)	1.15 (0.72, 1.80)	1.43 (0.82, 2.48)	0.82 (0.57, 1.17)	-	-	1.03 (0.91, 1.16)
-	-	-	0.62 (0.35, 1.09)	-	-	-	-	0.72 (0.52, 1.01)	-	-	-	-	-	0.81 (0.61, 1.06)	**Rebo**	-	-	-	-	-	0.94 (0.74, 1.20)
-	1.08 (0.83, 1.38)	-	0.62 (0.37, 1.03)	1.85 (0.94, 3.59)	-	-	1.08 (0.69, 1.64)	1.16 (0.86, 1.64)	2.27 (0.95, 6.05)	-	1.68 (0.58, 5.25)	1.31 (0.75, 2.31)	1.02 (0.48, 2.10)	0.87 (0.56, 1.38)	-	**Sert**	0.57 (0.17, 1.85)	1.21 (0.76, 1.93)	-	-	0.94 (0.78, 1.12)
-	0.99 (0.57, 1.72)	1.08 (0.52, 2.27)	-	-	-	-	-	0.65 (0.36, 1.14)	-	-	-	0.91 (0.51, 1.57)	-	0.70 (0.40, 1.23)	-	1.74 (0.54, 5.91)	**Traz**	0.86 (0.42, 1.72)	-	-	0.84 (0.61, 1.16)
0.49 (0.29, 0.77)	1.00 (0.53, 1.84)	0.87 (0.53, 1.40)	0.10 (0.01, 0.46)	1.05 (0.43, 2.42)	-	1.56 (1.07, 2.28)	0.89 (0.57, 1.41)	0.88 (0.73, 1.06)	2.08 (0.84, 5.12)	-	-	0.73 (0.49, 1.08)	-	1.22 (0.85, 1.77)	-	0.82 (0.52, 1.32)	1.16 (0.58, 2.40)	**Venl**	-	0.65 (0.44, 0.97)	1.00 (0.86, 1.18)
-	-	-	0.83 (0.55, 1.25)	-	-	-	-	-	-	-	-	-	-	-	-	-	-	-	**Vila**	-	0.86 (0.65, 1.14)
-	-	-	-	-	-	1.04 (0.83, 1.31)	-	-	-	-	-	-	-	-	-	-	-	1.54 (1.03, 2.27)	-	**Vort**	0.93 (0.77, 1.12)
0.86 (0.70, 1.06)	0.75 (0.61, 0.93)	1.01 (0.82, 1.24)	1.05 (0.83, 1.33)	0.43 (0.07, 2.40)	1.07 (0.85, 1.34)	1.07 (0.91, 1.26)	0.99 (0.84, 1.17)	0.86 (0.75, 0.99)	1.09 (0.81, 1.47)	1.19 (0.92, 1.55)	-	0.91 (0.71, 1.18)	0.84 (0.62, 1.15)	0.97 (0.86, 1.09)	1.07 (0.84, 1.36)	1.07 (0.89, 1.27)	1.19 (0.86, 1.65)	1.00 (0.85, 1.17)	1.16 (0.88, 1.54)	1.08 (0.89, 1.30)	**Plac**

7.1.3 Efficacy continuous

**Agom**	-	-	-	-	-	-0.24 (-0.49, 0.02)	0.11 (-0.12, 0.33)	0.00 (-0.12, 0.13)	-	-	-	-	-	-0.16 (-0.29,-0.04)	-	-	-	0.06 (-0.14, 0.26)	-	-	0.25 (0.15, 0.35)
-	**Amit**	-	-	-	-	-	-	0.18 (0.01, 0.35)	-0.02 (-0.35, 0.31)	-	0.11 (-0.18, 0.40)	0.09 (-0.13, 0.31)	-	0.07 (-0.05, 0.20)	-	0.19 (0.06, 0.32)	0.08 (-0.25, 0.41)	0.11 (-0.16, 0.37)	-	-	0.62 (0.51, 0.73)
-	-	**Bupr**	-	-	-	-	-0.06 (-0.26, 0.15)	-0.02 (-0.21, 0.16)	-	-	-	-	-	-0.01 (-0.39, 0.37)	-	-0.04 (-1.06, 0.93)	0.13 (-0.28, 0.53)	-0.00 (-0.27, 0.27)	-	-	0.22 (0.12, 0.33)
-	-	-	**Cita**	-0.38 (-0.81, 0.05)	-	-	-0.13 (-0.23,-0.03)	0.05 (-0.14, 0.25)	0.19 (-0.13, 0.50)	-	-	0.02 (-0.27, 0.31)	-	0.13 (-0.17, 0.43)	0.26 (-0.02, 0.53)	0.03 (-0.16, 0.23)	-	0.07 (-0.30, 0.44)	-0.04 (-0.22, 0.15)	-	0.21 (0.10, 0.32)
-	-	-	0.38 (-0.05, 0.81)	**Clom**	-	-	-	-0.16 (-0.46, 0.14)	0.16 (-0.30, 0.61)	-	0.69 (0.26, 1.13)	-	-	-0.00 (-0.15, 0.15)	-	-0.08 (-0.36, 0.19)	-	-0.13 (-0.57, 0.29)	-	-	-
-	-	-	-	-	**Desv**	-0.02 (-0.28, 0.24)	-	-	-	-	-	-	-	-	-	-	-	-	-	-	0.24 (0.13, 0.35)
0.24 (-0.02, 0.49)	-	-	-	-	0.02 (-0.24, 0.28)	**Dulo**	-0.09 (-0.24, 0.07)	0.17 (-0.14, 0.48)	-	-	-	-	-	0.09 (-0.01, 0.21)	-	-	-	-0.06 (-0.24, 0.13)	-	0.21 (0.10, 0.32)	0.38 (0.30, 0.46)
-0.11 (-0.33, 0.12)	-	0.06 (-0.15, 0.26)	0.13 (0.03, 0.23)	-	-	0.09 (-0.07, 0.24)	**Esci**	0.03 (-0.15, 0.21)	-	-	-	-	-	0.02 (-0.14, 0.17)	-	0.01 (-0.17, 0.20)	-	0.07 (-0.15, 0.28)	-	-	0.21 (0.13, 0.30)
-0.00 (-0.13, 0.12)	-0.18 (-0.35,-0.01)	0.02 (-0.16, 0.21)	-0.05 (-0.25, 0.14)	0.16 (-0.14, 0.46)	-	-0.17 (-0.48, 0.14)	-0.03 (-0.21, 0.15)	**Fluo**	-0.12 (-0.40, 0.16)	-	-0.17 (-0.46, 0.12)	-0.21 (-0.40,-0.02)	-0.05 (-0.37, 0.28)	-0.03 (-0.13, 0.06)	0.07 (-0.10, 0.23)	-0.08 (-0.24, 0.07)	-0.04 (-0.39, 0.32)	-0.12 (-0.21,-0.03)	-	-	0.22 (0.15, 0.28)
-	0.02 (-0.31, 0.35)	-	-0.19 (-0.50, 0.13)	-0.16 (-0.61, 0.30)	-	-	-	0.12 (-0.16, 0.40)	**Fluv**	-	-0.20 (-0.50, 0.10)	-0.07 (-0.31, 0.16)	-	-0.02 (-0.35, 0.32)	-	0.20 (-0.12, 0.52)	-	-0.17 (-0.76, 0.42)	-	-	0.44 (0.24, 0.64)
-	-	-	-	-	-	-	-	-	-	**Levo**	-	-	-	-	-	-	-	-	-	-	0.27 (0.12, 0.41)
-	-0.11 (-0.40, 0.18)	-	-	-0.69 (-1.13,-0.26)	-	-	-	0.17 (-0.12, 0.46)	0.20 (-0.10, 0.50)	-	**Miln**	-	-	-0.07 (-0.24, 0.11)	-	-	-	-	-	-	-
-	-0.09 (-0.31, 0.13)	-	-0.02 (-0.31, 0.27)	-	-	-	-	0.21 (0.02, 0.40)	0.07 (-0.16, 0.31)	-	-	**Mirt**	-	0.05 (-0.13, 0.23)	-	-	0.31 (-0.12, 0.73)	0.01 (-0.21, 0.22)	-	-	0.37 (0.23, 0.51)
-	-	-	-	-	-	-	-	0.05 (-0.28, 0.37)	-	-	-	-	**Nefa**	-0.15 (-0.44, 0.14)	-	0.00 (-0.37, 0.36)	-	-	-	-	0.31 (0.14, 0.47)
0.16 (0.04, 0.29)	-0.07 (-0.20, 0.05)	0.01 (-0.37, 0.39)	-0.13 (-0.43, 0.17)	0.00 (-0.15, 0.15)	-	-0.09 (-0.21, 0.01)	-0.02 (-0.17, 0.14)	0.03 (-0.06, 0.13)	0.02 (-0.32, 0.35)	-	0.07 (-0.11, 0.24)	-0.05 (-0.23, 0.13)	0.15 (-0.14, 0.44)	**Paro**	0.15 (0.00, 0.30)	0.07 (-0.14, 0.28)	0.19 (-0.06, 0.45)	-0.20 (-0.41, 0.00)	-	-	0.30 (0.24, 0.36)
-	-	-	-0.26 (-0.53, 0.02)	-	-	-	-	-0.07 (-0.23, 0.10)	-	-	-	-	-	-0.15 (-0.30,-0.00)	**Rebo**	-	-	-0.15 (-0.50, 0.20)	-	-	0.19 (0.06, 0.32)
-	-0.19 (-0.32,-0.06)	0.04 (-0.93, 1.06)	-0.03 (-0.23, 0.16)	0.08 (-0.19, 0.36)	-	-	-0.01 (-0.20, 0.17)	0.08 (-0.07, 0.24)	-0.20 (-0.52, 0.12)	-	-	-	-0.00 (-0.36, 0.37)	-0.07 (-0.28, 0.14)	-	**Sert**	-0.13 (-0.52, 0.26)	0.01 (-0.24, 0.26)	-	-	0.28 (0.18, 0.37)
-	-0.08 (-0.41, 0.25)	-0.13 (-0.53, 0.28)	-	-	-	-	-	0.04 (-0.32, 0.39)	-	-	-	-0.31 (-0.73, 0.12)	-	-0.19 (-0.45, 0.06)	-	0.13 (-0.26, 0.52)	**Traz**	-0.23 (-0.62, 0.15)	-	-	0.38 (0.21, 0.56)
-0.06 (-0.26, 0.14)	-0.11 (-0.37, 0.16)	0.00 (-0.27, 0.27)	-0.07 (-0.44, 0.30)	0.13 (-0.29, 0.57)	-	0.06 (-0.13, 0.24)	-0.07 (-0.28, 0.15)	0.12 (0.03, 0.21)	0.17 (-0.42, 0.76)	-	-	-0.01 (-0.22, 0.21)	-	0.20 (-0.00, 0.41)	0.15 (-0.20, 0.50)	-0.01 (-0.26, 0.24)	0.23 (-0.15, 0.62)	**Venl**	-	-0.08 (-0.27, 0.11)	0.34 (0.26, 0.42)
-	-	-	0.04 (-0.15, 0.22)	-	-	-	-	-	-	-	-	-	-	-	-	-	-	-	**Vila**	-	0.26 (0.13, 0.39)
-	-	-	-	-	-	-0.21 (-0.32,-0.10)	-	-	-	-	-	-	-	-	-	-	-	0.08 (-0.11, 0.27)	-	**Vort**	0.31 (0.21, 0.40)
-0.25 (-0.35,-0.15)	-0.62 (-0.73,-0.51)	-0.22 (-0.33,-0.12)	-0.21 (-0.32,-0.10)	-	-0.24 (-0.35,-0.13)	-0.38 (-0.46,-0.30)	-0.21 (-0.30,-0.13)	-0.22 (-0.28,-0.15)	-0.44 (-0.64,-0.24)	-0.27 (-0.41,-0.12)	-	-0.37 (-0.51,-0.23)	-0.31 (-0.47,-0.14)	-0.30 (-0.36,-0.24)	-0.19 (-0.32,-0.06)	-0.28 (-0.37,-0.18)	-0.38 (-0.56,-0.21)	-0.34 (-0.42,-0.26)	-0.26 (-0.39,-0.13)	-0.31 (-0.40,-0.21)	**Plac**

7.1.4 Remission

**Ago m**	-	-	-	-	-	1.88 (1.03, 3.46)	0.80 (0.48, 1.33)	1.01 (0.75, 1.37)	-	-	-	-	-	1.35 (1.01, 1.82)	-	-	-	0.77 (0.49, 1.21)	-	-	0.77 (0.63, 0.94)
-	**Amit**	-	-	-	-	-	-	0.87 (0.63, 1.21)	0.93 (0.53, 1.63)	-	0.66 (0.29, 1.53)	0.91 (0.53, 1.57)	1.12 (0.28, 4.60)	0.99 (0.77, 1.28)	-	0.75 (0.56, 1.00)	0.74 (0.43, 1.28)	1.07 (0.58, 1.97)	-	-	0.37 (0.29, 0.46)
-	-	**Bupr**	-	-	-	-	1.07 (0.69, 1.67)	0.87 (0.59, 1.30)	-	-	-	-	-	1.03 (0.44, 2.41)	-	1.30 (0.12, 16.56)	0.47 (0.20, 1.11)	0.64 (0.35, 1.20)	-	-	0.67 (0.54, 0.82)
-	-	-	**Cita**	3.72 (1.54, 9.43)	-	-	1.51 (1.22, 1.87)	0.94 (0.62, 1.44)	0.54 (0.20, 1.39)	-	-	0.91 (0.48, 1.75)	-	0.77 (0.38, 1.54)	0.64 (0.35, 1.16)	1.06 (0.68, 1.66)	-	0.44 (0.21, 0.90)	1.06 (0.69, 1.63)	-	0.73 (0.60, 0.90)
-	-	-	0.27 (0.11, 0.65)	**Clom**	-	-	-	1.41 (0.86, 2.34)	0.64 (0.22, 1.80)	-	0.61 (0.25, 1.45)	-	-	0.97 (0.70, 1.33)	-	1.09 (0.60, 2.01)	-	1.34 (0.48, 3.85)	-	-	-
-	-	-	-	-	**Desv**	1.37 (0.75, 2.49)	-	-	-	-	-	-	-	-	-	-	-	-	-	-	0.71 (0.59, 0.87)
0.53 (0.29, 0.97)	-	-	-	-	0.73 (0.40, 1.33)	**Dulo**	1.03 (0.73, 1.46)	0.63 (0.32, 1.23)	-	-	-	-	-	0.97 (0.76, 1.23)	-	-	-	1.14 (0.75, 1.73)	-	0.78 (0.61, 0.99)	0.56 (0.49, 0.65)
1.25 (0.75, 2.08)	-	0.93 (0.60, 1.45)	0.66 (0.53, 0.82)	-	-	0.98 (0.69, 1.38)	**Esci**	1.00 (0.69, 1.46)	-	-	-	-	-	0.96 (0.68, 1.35)	-	1.22 (0.71, 2.10)	-	1.00 (0.62, 1.63)	-	-	0.69 (0.59, 0.81)
0.99 (0.73, 1.34)	1.14 (0.82, 1.58)	1.14 (0.77, 1.70)	1.06 (0.70, 1.62)	0.71 (0.43, 1.17)	-	1.58 (0.81, 3.13)	1.00 (0.69, 1.46)	**Fluo**	1.18 (0.66, 2.11)	-	1.31 (0.65, 2.72)	1.14 (0.82, 1.62)	0.94 (0.44, 2.02)	1.10 (0.89, 1.36)	0.84 (0.58, 1.20)	1.17 (0.86, 1.60)	1.05 (0.57, 1.91)	1.18 (0.97, 1.44)	-	-	0.73 (0.64, 0.83)
-	1.07 (0.61, 1.88)	-	1.84 (0.72, 5.00)	1.57 (0.56, 4.57)	-	-	-	0.85 (0.47, 1.51)	**Fluv**	-	-	0.84 (0.52, 1.36)	-	1.13 (0.50, 2.46)	-	0.68 (0.34, 1.36)	-	1.93 (0.76, 5.01)	-	-	0.58 (0.39, 0.86)
-	-	-	-	-	-	-	-	-	-	**Levo**	-	-	-	-	-	-	-	-	-	-	0.75 (0.58, 0.97)
-	1.52 (0.66, 3.49)	-	-	1.65 (0.69, 3.99)	-	-	-	0.76 (0.37, 1.55)	-	-	**Miln**	-	-	1.04 (0.70, 1.53)	-	-	-	-	-	-	-
-	1.10 (0.64, 1.87)	-	1.09 (0.57, 2.09)	-	-	-	-	0.87 (0.62, 1.23)	1.19 (0.73, 1.94)	-	-	**Mirta**	-	0.89 (0.63, 1.27)	-	0.99 (0.55, 1.79)	0.67 (0.17, 2.46)	0.97 (0.58, 1.63)	-	-	0.59 (0.43, 0.80)
-	0.89 (0.22, 3.63)	-	-	-	-	-	-	1.06 (0.49, 2.29)	-	-	-	-	**Nefa**	1.33 (0.63, 2.82)	-	0.94 (0.40, 2.19)	-	-	-	-	0.50 (0.35, 0.72)
0.74 (0.55, 0.99)	1.01 (0.78, 1.31)	0.97 (0.41, 2.26)	1.30 (0.65, 2.63)	1.04 (0.75, 1.43)	-	1.03 (0.81, 1.31)	1.04 (0.74, 1.47)	0.91 (0.73, 1.12)	0.89 (0.41, 2.00)	-	0.97 (0.65, 1.43)	1.13 (0.79, 1.59)	0.75 (0.35, 1.59)	**Paro**	0.80 (0.58, 1.11)	0.98 (0.62, 1.54)	0.75 (0.42, 1.33)	1.55 (1.04, 2.34)	-	-	0.61 (0.54, 0.68)
-	-	-	1.57 (0.86, 2.86)	-	-	-	-	1.19 (0.83, 1.71)	-	-	-	-	-	1.24 (0.90, 1.72)	**Rebo**	-	-	0.95 (0.42, 2.18)	-	-	0.82 (0.65, 1.03)
-	1.34 (1.00, 1.80)	0.77 (0.06, 8.17)	0.94 (0.60, 1.47)	0.92 (0.50, 1.67)	-	-	0.82 (0.48, 1.40)	0.85 (0.63, 1.16)	1.47 (0.74, 2.96)	-	-	1.01 (0.56, 1.83)	1.07 (0.46, 2.48)	1.02 (0.65, 1.60)	-	**Sert**	1.59 (0.71, 3.67)	1.25 (0.81, 1.94)	-	-	0.67 (0.55, 0.83)
-	1.35 (0.78, 2.34)	2.13 (0.90, 5.10)	-	-	-	-	-	0.95 (0.52, 1.75)	-	-	-	1.50 (0.41, 5.97)	-	1.34 (0.75, 2.37)	-	0.63 (0.27, 1.41)	**Traz**	2.53 (0.92, 7.69)	-	-	0.72 (0.52, 1.02)
1.31 (0.83, 2.05)	0.94 (0.51, 1.72)	1.55 (0.84, 2.90)	2.28 (1.12, 4.82)	0.74 (0.26, 2.07)	-	0.88 (0.58, 1.33)	1.00 (0.61, 1.61)	0.85 (0.70, 1.03)	0.52 (0.20, 1.31)	-	-	1.03 (0.62, 1.71)	-	0.64 (0.43, 0.96)	1.05 (0.46, 2.37)	0.80 (0.52, 1.23)	0.40 (0.13, 1.09)	**Venl**	-	1.09 (0.72, 1.64)	0.56 (0.48, 0.65)
-	-	-	0.95 (0.61, 1.46)	-	-	-	-	-	-	-	-	-	-	-	-	-	-	-	**Vila**	-	0.69 (0.54, 0.86)
-	-	-	-	-	-	1.28 (1.01, 1.64)	-	-	-	-	-	-	-	-	-	-	-	0.92 (0.61, 1.39)	-	**Vort**	0.67 (0.57, 0.79)
1.30 (1.06, 1.59)	2.71 (2.16, 3.44)	1.50 (1.23, 1.84)	1.36 (1.11, 1.67)	-	1.40 (1.15, 1.71)	1.77 (1.54, 2.04)	1.45 (1.24, 1.70)	1.37 (1.20, 1.56)	1.73 (1.16, 2.59)	1.33 (1.03, 1.73)	-	1.70 (1.26, 2.31)	2.00 (1.40, 2.89)	1.65 (1.47, 1.86)	1.22 (0.97, 1.53)	1.48 (1.20, 1.83)	1.38 (0.98, 1.94)	1.79 (1.53, 2.09)	1.46 (1.16, 1.84)	1.48 (1.26, 1.75)	**Plac**

7.1.5 Dropouts due to adverse events

**Agom**	-	-	-	-	-	1.09 (0.46, 2.52)	3.46 (1.32, 10.23)	1.41 (0.89, 2.23)	-	-	-	-	-	1.31 (0.84, 2.05)	-	-	-	3.85 (1.78, 8.79)	-	-	0.94 (0.66, 1.33)
-	**Amit**	1.97 (0.21, 52.51)	-	-	-	-	-	0.35 (0.21, 0.58)	0.55 (0.28, 1.08)	-	0.54 (0.10, 2.70)	0.71 (0.39, 1.26)	-	0.97 (0.68, 1.35)	-	0.86 (0.60, 1.23)	0.94 (0.38, 2.42)	0.99 (0.37, 2.68)	-	-	0.31 (0.22, 0.43)
-	0.51 (0.02, 4.70)	**Bupr**	-	-	-	-	0.68 (0.29, 1.66)	0.73 (0.34, 1.54)	-	-	-	-	-	0.57 (0.15, 1.96)	-	-	1.37 (0.53, 3.65)	1.85 (0.78, 4.61)	-	-	0.49 (0.34, 0.70)
-	-	-	**Cita**	7.35 (0.90, 223.18)	-	-	0.88 (0.54, 1.42)	0.60 (0.29, 1.21)	1.84 (0.82, 4.29)	-	-	2.07 (0.59, 8.59)	-	1.94 (0.66, 5.37)	-	2.33 (0.40, 22.07)	-	7.03 (1.47, 58.79)	1.50 (0.70, 3.29)	-	0.41 (0.26, 0.64)
-	-	-	0.14 (0.00, 1.11)	**Clom**	-	-	-	0.29 (0.09, 0.76)	2.75 (0.19, 192.10)	-	0.51 (0.15, 1.62)	-	-	0.52 (0.34, 0.80)	-	0.41 (0.18, 0.92)	-	0.61 (0.19, 1.91)	-	-	1.09 (0.09, 11.46)
-	-	-	-	-	**Desv**	2.22 (1.03, 4.96)	-	-	-	-	-	-	-	-	-	-	-	-	-	-	0.58 (0.39, 0.85)
0.91 (0.40, 2.17)	-	-	-	-	0.45 (0.20, 0.98)	**Dulo**	0.44 (0.25, 0.73)	0.18 (0.00, 1.48)	-	-	-	-	-	0.98 (0.64, 1.48)	-	-	-	0.50 (0.28, 0.88)	-	0.93 (0.66, 1.33)	0.48 (0.37, 0.62)
0.29 (0.10, 0.76)	-	1.48 (0.60, 3.45)	1.13 (0.70, 1.84)	-	-	2.28 (1.37, 3.94)	**Esci**	1.36 (0.76, 2.48)	-	-	-	-	-	0.77 (0.32, 1.75)	-	0.82 (0.34, 1.97)	-	2.42 (1.21, 5.03)	-	-	0.53 (0.40, 0.71)
0.71 (0.45, 1.13)	2.87 (1.72, 4.87)	1.37 (0.65, 2.97)	1.65 (0.83, 3.42)	3.51 (1.32, 10.62)	-	5.44 (0.68, 298.27)	0.73 (0.40, 1.32)	**Fluo**	0.97 (0.08, 10.00)	-	0.63 (0.30, 1.35)	1.34 (0.82, 2.23)	1.30 (0.40, 5.45)	1.28 (0.93, 1.73)	1.88 (1.14, 3.14)	0.70 (0.38, 1.23)	1.11 (0.42, 2.81)	1.57 (1.17, 2.09)	-	-	0.54 (0.43, 0.68)
-	1.82 (0.93, 3.60)	-	0.54 (0.23, 1.22)	0.36 (0.01, 5.23)	-	-	-	1.03 (0.10, 12.68)	**Fluv**	-	0.41 (0.14, 1.25)	1.72 (0.83, 3.84)	-	0.65 (0.22, 1.75)	-	0.35 (0.09, 1.14)	-	-	-	-	0.31 (0.20, 0.46)
-	-	-	-	-	-	-	-	-	-	**Levo**	-	-	-	-	-	-	-	-	-	-	0.39 (0.25, 0.60)
-	1.86 (0.37, 10.39)	-	-	1.96 (0.62, 6.72)	-	-	-	1.59 (0.74, 3.38)	2.41 (0.80, 7.19)	-	**Miln**	-	-	1.10 (0.66, 1.83)	-	-	-	-	-	-	-
-	1.41 (0.79, 2.54)	-	0.48 (0.12, 1.69)	-	-	-	-	0.74 (0.45, 1.23)	0.58 (0.26, 1.20)	-	-	**Mirt**	-	1.44 (0.85, 2.44)	-	0.21 (0.06, 0.60)	1.80 (0.68, 5.06)	1.46 (0.85, 2.52)	-	-	0.61 (0.41, 0.88)
-	-	-	-	-	-	-	-	0.77 (0.18, 2.52)	-	-	-	-	**Nefa**	0.69 (0.29, 1.58)	-	0.56 (0.21, 1.53)	-	-	-	-	0.58 (0.37, 0.91)
0.76 (0.49, 1.18)	1.04 (0.74, 1.46)	1.74 (0.51, 6.59)	0.52 (0.19, 1.53)	1.93 (1.26, 2.97)	-	1.02 (0.68, 1.55)	1.30 (0.57, 3.12)	0.78 (0.58, 1.07)	1.55 (0.57, 4.52)	-	0.91 (0.55, 1.52)	0.70 (0.41, 1.18)	1.44 (0.63, 3.46)	**Paro**	1.40 (0.91, 2.15)	0.50 (0.15, 1.52)	1.49 (0.69, 3.27)	0.79 (0.45, 1.38)	-	-	0.48 (0.40, 0.58)
-	-	-	-	-	-	-	-	0.53 (0.32, 0.88)	-	-	-	-	-	0.72 (0.46, 1.10)	**Rebo**	-	-	1.11 (0.32, 3.97)	-	-	0.45 (0.31, 0.65)
-	1.17 (0.81, 1.67)	-	0.43 (0.05, 2.53)	2.44 (1.09, 5.64)	-	-	1.22 (0.51, 2.94)	1.44 (0.81, 2.62)	2.86 (0.87, 11.68)	-	-	4.85 (1.68, 15.69)	1.78 (0.65, 4.69)	2.00 (0.66, 6.46)	-	**Sertr**	0.26 (0.03, 1.33)	2.36 (1.02, 6.05)	-	-	0.36 (0.26, 0.50)
-	1.06 (0.41, 2.60)	0.73 (0.27, 1.87)	-	-	-	-	-	0.90 (0.36, 2.37)	-	-	-	0.56 (0.20, 1.47)	-	0.67 (0.31, 1.45)	-	3.85 (0.75, 31.94)	**Trazo**	0.68 (0.27, 1.65)	-	-	0.27 (0.15, 0.50)
0.26 (0.11, 0.56)	1.01 (0.37, 2.72)	0.54 (0.22, 1.28)	0.14 (0.02, 0.68)	1.63 (0.52, 5.40)	-	2.02 (1.13, 3.59)	0.41 (0.20, 0.82)	0.64 (0.48, 0.85)	-	-	-	0.68 (0.40, 1.17)	-	1.27 (0.72, 2.20)	0.90 (0.25, 3.16)	0.42 (0.17, 0.98)	1.48 (0.60, 3.71)	**Venl**	-	0.37 (0.20, 0.67)	0.33 (0.26, 0.43)
-	-	-	0.67 (0.30, 1.42)	-	-	-	-	-	-	-	-	-	-	-	-	-	-	-	**Vila**	-	0.45 (0.27, 0.72)
-	-	-	-	-	-	1.07 (0.75, 1.52)	-	-	-	-	-	-	-	-	-	-	-	2.67 (1.49, 4.95)	-	**Vort**	0.66 (0.49, 0.89)
1.06 (0.75, 1.52)	3.23 (2.34, 4.49)	2.05 (1.43, 2.97)	2.44 (1.57, 3.84)	0.92 (0.09, 10.70)	1.72 (1.18, 2.53)	2.09 (1.62, 2.72)	1.88 (1.41, 2.52)	1.85 (1.48, 2.33)	3.27 (2.17, 5.07)	2.55 (1.66, 4.01)	-	1.64 (1.14, 2.41)	1.72 (1.09, 2.74)	2.09 (1.73, 2.52)	2.22 (1.54, 3.22)	2.75 (1.98, 3.83)	3.65 (1.98, 6.80)	3.02 (2.34, 3.92)	2.24 (1.38, 3.67)	1.51 (1.12, 2.06)	**Plac**

7.2 Results from network meta-analyses

Below we present the network diagram, the forest plot of the effect estimate for each active intervention versus placebo, and the league table of all comparisons for each of the two primary and three secondary outcomes.

7.2.1 Response

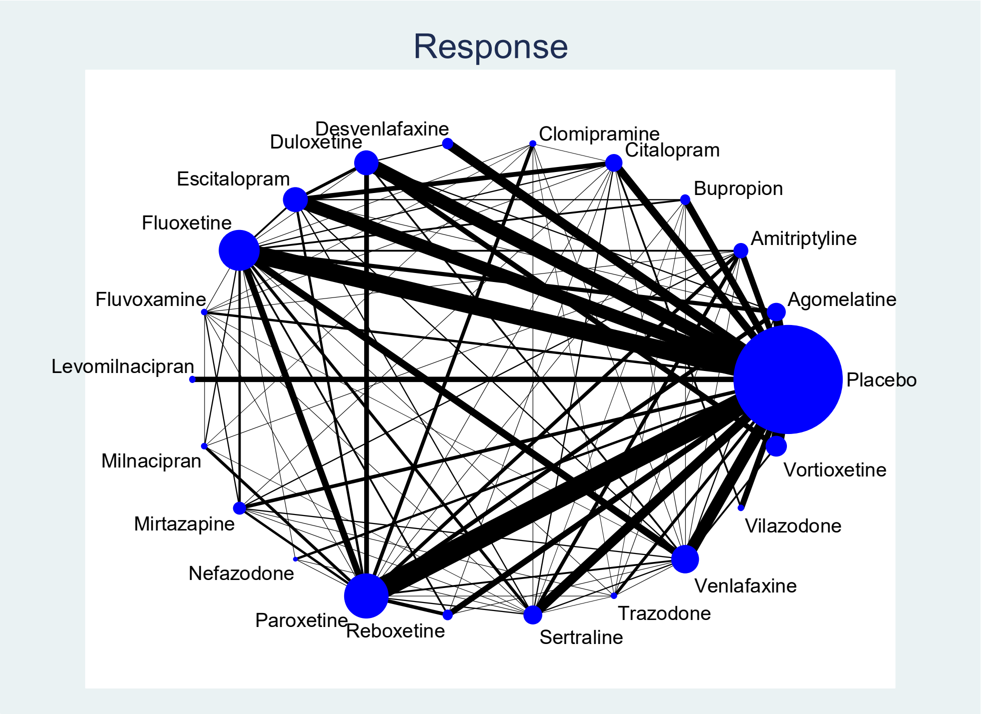

Response

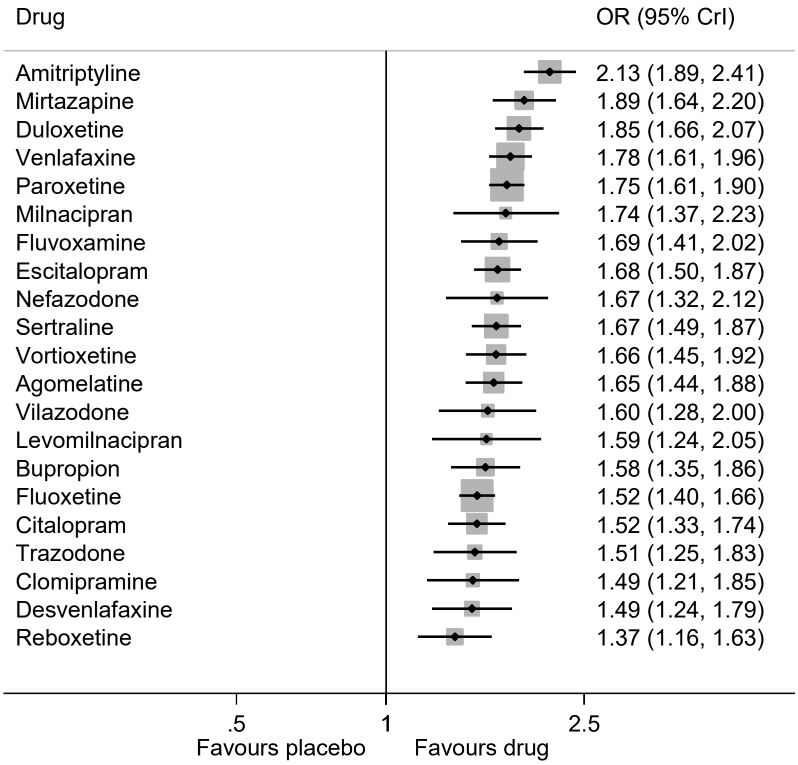
Drug
OR (95% CrI)
Amitriptyline
2.13 (1.89, 2.41)
Mirtazapine
1.89 (1.64, 2.20)
Duloxetine
1.85 (1.66, 2.07)
Venlafaxine
1.78 (1.61, 1.96)
Paroxetine
1.75 (1.61, 1.90)
Milnacipran
1.74 (1.37, 2.23)
Fluvoxamine
1.69 (1.41, 2.02)
Escitalopram
1.68 (1.50, 1.87)
Nefazodone
1.67 (1.32, 2.12)
Sertraline
1.67 (1.49, 1.87)
Vortioxetine
1.66 (1.45, 1.92)
Agomelatine
1.65 (1.44, 1.88)
Vilazodone
1.60 (1.28, 2.00)
Levomilnacipran
1.59 (1.24, 2.05)
Bupropion
1.58 (1.35, 1.86)
Fluoxetine
1.52 (1.40, 1.66)
Citalopram
1.52 (1.33, 1.74)
Trazodone
1.51 (1.25, 1.83)
Clomipramine
1.49 (1.21, 1.85)
Desvenlafaxine
1.49 (1.24, 1.79)
Reboxetine
1.37 (1.16, 1.63)
.5
1
2.5
Favours placebo
Favours drug

**Agom**																					
0.77 (0.65, 0.92)	**Amit**																				
1.04 (0.85, 1.27)	1.35 (1.10, 1.64)	**Bupr**																			
1.08 (0.90, 1.30)	1.40 (1.18, 1.67)	1.04 (0.85, 1.28)	**Cita**																		
1.10 (0.86, 1.41)	1.43 (1.13, 1.81)	1.06 (0.81, 1.38)	1.02 (0.80, 1.30)	**Clom**																	
1.11 (0.88, 1.39)	1.43 (1.15, 1.79)	1.06 (0.83, 1.36)	1.02 (0.81, 1.28)	1.00 (0.76, 1.34)	**Desv**																
0.89 (0.75, 1.05)	1.15 (0.98, 1.35)	0.86 (0.71, 1.04)	0.82 (0.69, 0.97)	0.81 (0.64, 1.02)	0.80 (0.65, 0.99)	**Dulo**															
0.98 (0.84, 1.16)	1.27 (1.09, 1.49)	0.95 (0.78, 1.14)	0.91 (0.78, 1.05)	0.89 (0.71, 1.13)	0.89 (0.72, 1.10)	1.11 (0.95, 1.28)	**Esci**														
1.08 (0.94, 1.25)	1.40 (1.22, 1.60)	1.04 (0.87, 1.24)	1.00 (0.86, 1.16)	0.98 (0.79, 1.22)	0.98 (0.80, 1.19)	1.21 (1.06, 1.39)	1.10 (0.97, 1.25)	**Fluo**													
0.98 (0.78, 1.21)	1.27 (1.03, 1.55)	0.94 (0.74, 1.19)	0.90 (0.73, 1.12)	0.89 (0.68, 1.16)	0.88 (0.68, 1.14)	1.10 (0.89, 1.35)	0.99 (0.81, 1.22)	0.90 (0.75, 1.09)	**Fluvo**												
1.03 (0.78, 1.37)	1.34 (1.01, 1.77)	0.99 (0.74, 1.34)	0.96 (0.72, 1.27)	0.94 (0.67, 1.31)	0.93 (0.68, 1.28)	1.16 (0.88, 1.53)	1.05 (0.80, 1.39)	0.96 (0.73, 1.25)	1.06 (0.78, 1.44)	**Levo**											
0.94 (0.72, 1.24)	1.23 (0.95, 1.58)	0.91 (0.68, 1.21)	0.87 (0.66, 1.15)	0.86 (0.63, 1.16)	0.85 (0.63, 1.16)	1.06 (0.81, 1.39)	0.96 (0.74, 1.25)	0.88 (0.68, 1.12)	0.97 (0.73, 1.28)	0.91 (0.64, 1.30)	**Miln**										
0.87 (0.72, 1.05)	1.13 (0.94, 1.34)	0.84 (0.68, 1.04)	0.80 (0.66, 0.97)	0.79 (0.61, 1.01)	0.79 (0.62, 0.99)	0.98 (0.81, 1.17)	0.88 (0.74, 1.06)	0.80 (0.69, 0.94)	0.89 (0.72, 1.10)	0.84 (0.63, 1.13)	0.92 (0.70, 1.21)	**Mirt**									
0.98 (0.75, 1.29)	1.28 (0.98, 1.66)	0.95 (0.71, 1.26)	0.91 (0.70, 1.19)	0.89 (0.65, 1.23)	0.89 (0.66, 1.20)	1.11 (0.85, 1.44)	1.00 (0.77, 1.30)	0.91 (0.71, 1.17)	1.01 (0.75, 1.36)	0.95 (0.67, 1.35)	1.04 (0.74, 1.47)	1.13 (0.86, 1.50)	**Nefa**								
0.94 (0.82, 1.08)	1.22 (1.07, 1.39)	0.91 (0.76, 1.08)	0.87 (0.75, 1.01)	0.85 (0.69, 1.06)	0.85 (0.69, 1.04)	1.06 (0.93, 1.20)	0.96 (0.84, 1.09)	0.87 (0.79, 0.96)	0.96 (0.80, 1.16)	0.91 (0.70, 1.19)	1.00 (0.78, 1.27)	1.08 (0.93, 1.26)	0.96 (0.75, 1.22)	**Paro**							
1.20 (0.97, 1.48)	1.55 (1.27, 1.91)	1.15 (0.92, 1.45)	1.11 (0.90, 1.36)	1.09 (0.83, 1.42)	1.08 (0.84, 1.39)	1.35 (1.10, 1.65)	1.22 (1.00, 1.49)	1.11 (0.93, 1.33)	1.23 (0.96, 1.57)	1.16 (0.85, 1.57)	1.27 (0.95, 1.70)	1.38 (1.11, 1.72)	1.22 (0.91, 1.63)	1.27 (1.06, 1.52)	**Rebo**						
0.99 (0.83, 1.17)	1.28 (1.10, 1.48)	0.95 (0.78, 1.15)	0.91 (0.78, 1.08)	0.90 (0.71, 1.13)	0.89 (0.72, 1.11)	1.11 (0.95, 1.30)	1.01 (0.87, 1.17)	0.92 (0.80, 1.04)	1.01 (0.83, 1.24)	0.96 (0.72, 1.26)	1.05 (0.80, 1.36)	1.14 (0.95, 1.36)	1.00 (0.78, 1.30)	1.05 (0.92, 1.20)	0.82 (0.67, 1.01)	**Sert**					
1.09 (0.87, 1.37)	1.41 (1.14, 1.75)	1.05 (0.82, 1.34)	1.01 (0.80, 1.27)	0.99 (0.75, 1.31)	0.99 (0.75, 1.29)	1.23 (0.98, 1.53)	1.11 (0.89, 1.38)	1.01 (0.83, 1.24)	1.12 (0.86, 1.44)	1.05 (0.77, 1.45)	1.15 (0.85, 1.57)	1.26 (1.00, 1.58)	1.11 (0.82, 1.50)	1.16 (0.95, 1.42)	0.91 (0.71, 1.18)	1.10 (0.89, 1.37)	**Trazo**				
0.93 (0.79, 1.08)	1.20 (1.04, 1.39)	0.89 (0.74, 1.07)	0.86 (0.73, 1.00)	0.84 (0.67, 1.05)	0.84 (0.68, 1.03)	1.04 (0.90, 1.20)	0.94 (0.82, 1.08)	0.86 (0.77, 0.96)	0.95 (0.78, 1.16)	0.90 (0.68, 1.18)	0.98 (0.76, 1.27)	1.07 (0.90, 1.26)	0.94 (0.73, 1.21)	0.98 (0.87, 1.11)	0.77 (0.64, 0.93)	0.94 (0.81, 1.08)	0.85 (0.69, 1.05)	**Venl**			
1.03 (0.79, 1.34)	1.33 (1.03, 1.72)	0.99 (0.75, 1.30)	0.95 (0.74, 1.22)	0.93 (0.69, 1.27)	0.93 (0.70, 1.25)	1.16 (0.90, 1.49)	1.05 (0.82, 1.34)	0.95 (0.75, 1.21)	1.05 (0.79, 1.41)	1.00 (0.71, 1.40)	1.09 (0.78, 1.52)	1.18 (0.91, 1.55)	1.04 (0.75, 1.45)	1.09 (0.86, 1.39)	0.86 (0.65, 1.14)	1.04 (0.81, 1.34)	0.94 (0.70, 1.27)	1.11 (0.87, 1.42)	**Vila**		
0.99 (0.82, 1.20)	1.28 (1.07, 1.54)	0.95 (0.77, 1.18)	0.92 (0.75, 1.11)	0.90 (0.70, 1.16)	0.89 (0.71, 1.13)	1.11 (0.95, 1.31)	1.01 (0.84, 1.20)	0.92 (0.78, 1.08)	1.01 (0.81, 1.27)	0.96 (0.72, 1.28)	1.05 (0.79, 1.39)	1.14 (0.93, 1.40)	1.00 (0.76, 1.32)	1.05 (0.90, 1.24)	0.83 (0.66, 1.03)	1.00 (0.84, 1.20)	0.91 (0.72, 1.15)	1.07 (0.91, 1.26)	0.96 (0.73, 1.25)	**Vort**	
1.65 (1.44, 1.88)	2.13 (1.89, 2.41)	1.58 (1.35, 1.86)	1.52 (1.33, 1.74)	1.49 (1.21, 1.85)	1.49 (1.24, 1.79)	1.85 (1.66, 2.07)	1.68 (1.50, 1.87)	1.52 (1.40, 1.66)	1.69 (1.41, 2.02)	1.59 (1.24, 2.05)	1.74 (1.37, 2.23)	1.89 (1.64, 2.20)	1.67 (1.32, 2.12)	1.75 (1.61, 1.90)	1.37 (1.16, 1.63)	1.67 (1.49, 1.87)	1.51 (1.25, 1.83)	1.78 (1.61, 1.96)	1.60 (1.28, 2.00)	1.66 (1.45, 1.92)	**Plac**

OR>1 favour the treatment in the column

7.2.2 Dropouts for any reason

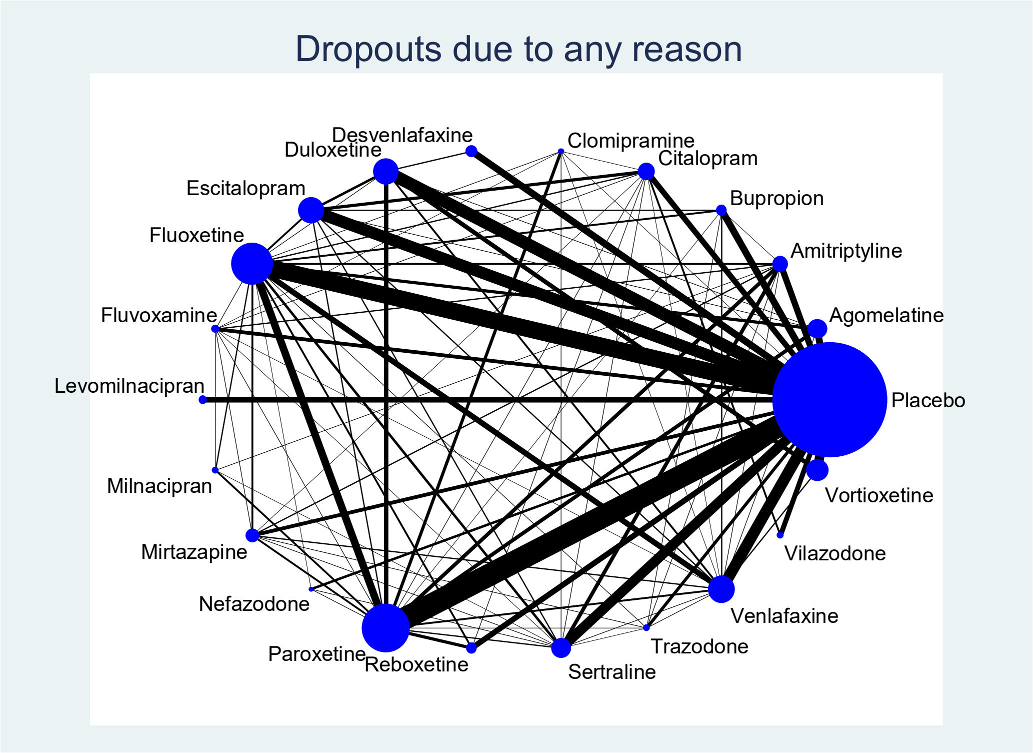

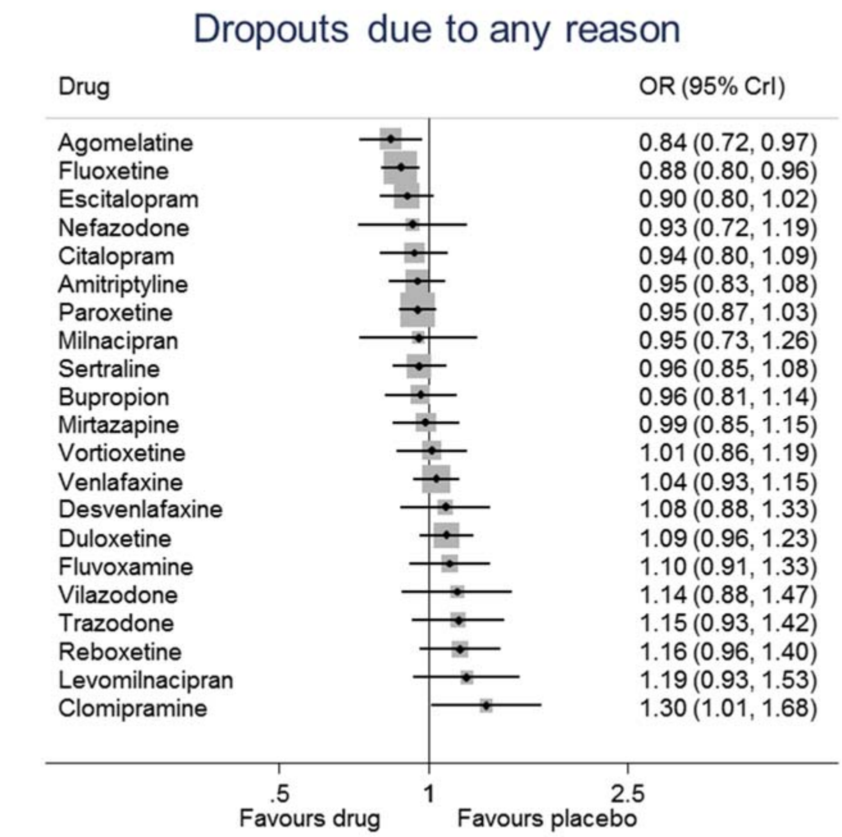

**Agom**																					
0.89 (0.73, 1.07)	**Amit**																				
0.87 (0.70, 1.09)	0.98 (0.80, 1.21)	**Bupr**																			
0.90 (0.73, 1.11)	1.01 (0.83, 1.24)	1.03 (0.82, 1.30)	**Cita**																		
0.64 (0.48, 0.86)	0.73 (0.55, 0.96)	0.74 (0.55, 1.00)	0.72 (0.54, 0.96)	**Clom**																	
0.78 (0.60, 1.00)	0.88 (0.69, 1.12)	0.89 (0.68, 1.17)	0.87 (0.67, 1.12)	1.21 (0.87, 1.68)	**Desv**																
0.77 (0.64, 0.93)	0.87 (0.73, 1.04)	0.89 (0.72, 1.09)	0.86 (0.71, 1.05)	1.20 (0.91, 1.59)	0.99 (0.79, 1.26)	**Dulo**															
0.93 (0.77, 1.12)	1.05 (0.88, 1.25)	1.07 (0.87, 1.30)	1.03 (0.86, 1.24)	1.44 (1.09, 1.90)	1.19 (0.94, 1.52)	1.20 (1.02, 1.41)	**Esci**														
0.96 (0.81, 1.12)	1.08 (0.94, 1.25)	1.10 (0.92, 1.32)	1.07 (0.90, 1.27)	1.49 (1.15, 1.93)	1.23 (0.98, 1.54)	1.24 (1.07, 1.43)	1.03 (0.89, 1.19)	**Fluo**													
0.76 (0.60, 0.96)	0.86 (0.70, 1.07)	0.88 (0.68, 1.12)	0.85 (0.67, 1.08)	1.19 (0.87, 1.62)	0.98 (0.74, 1.30)	0.99 (0.79, 1.23)	0.82 (0.66, 1.02)	0.80 (0.65, 0.97)	**Fluv**												
0.70 (0.53, 0.94)	0.80 (0.60, 1.06)	0.81 (0.60, 1.09)	0.78 (0.58, 1.05)	1.09 (0.77, 1.56)	0.91 (0.66, 1.25)	0.91 (0.69, 1.20)	0.76 (0.57, 1.00)	0.74 (0.57, 0.96)	0.92 (0.68, 1.26)	**Levo**											
0.88 (0.65, 1.20)	0.99 (0.74, 1.33)	1.01 (0.73, 1.39)	0.98 (0.72, 1.34)	1.37 (0.95, 1.97)	1.13 (0.80, 1.60)	1.14 (0.84, 1.53)	0.95 (0.70, 1.27)	0.92 (0.70, 1.21)	1.15 (0.85, 1.58)	1.25 (0.86, 1.81)	**Miln**										
0.85 (0.69, 1.05)	0.96 (0.80, 1.16)	0.98 (0.78, 1.22)	0.95 (0.77, 1.17)	1.32 (0.99, 1.76)	1.09 (0.84, 1.42)	1.10 (0.90, 1.34)	0.92 (0.76, 1.11)	0.89 (0.75, 1.05)	1.11 (0.89, 1.40)	1.21 (0.90, 1.62)	0.97 (0.71, 1.32)	**Mirt**									
0.91 (0.68, 1.21)	1.02 (0.78, 1.35)	1.04 (0.77, 1.41)	1.01 (0.75, 1.35)	1.41 (0.99, 2.01)	1.17 (0.84, 1.61)	1.17 (0.89, 1.55)	0.98 (0.74, 1.29)	0.95 (0.73, 1.23)	1.19 (0.87, 1.62)	1.29 (0.90, 1.83)	1.03 (0.71, 1.49)	1.07 (0.80, 1.43)	**Nefa**								
0.88 (0.75, 1.04)	1.00 (0.87, 1.15)	1.02 (0.84, 1.22)	0.99 (0.83, 1.17)	1.37 (1.07, 1.77)	1.14 (0.91, 1.42)	1.14 (0.99, 1.31)	0.95 (0.83, 1.10)	0.92 (0.83, 1.03)	1.16 (0.95, 1.41)	1.26 (0.97, 1.63)	1.00 (0.77, 1.32)	1.04 (0.88, 1.23)	0.98 (0.75, 1.27)	**Paro**							
0.73 (0.57, 0.92)	0.82 (0.66, 1.02)	0.83 (0.65, 1.07)	0.81 (0.64, 1.02)	1.13 (0.83, 1.53)	0.93 (0.71, 1.23)	0.94 (0.75, 1.17)	0.78 (0.63, 0.97)	0.76 (0.62, 0.92)	0.95 (0.73, 1.23)	1.03 (0.76, 1.40)	0.82 (0.60, 1.14)	0.85 (0.67, 1.08)	0.80 (0.59, 1.09)	0.82 (0.68, 1.00)	**Rebo**						
0.88 (0.73, 1.06)	0.99 (0.84, 1.16)	1.01 (0.82, 1.24)	0.98 (0.81, 1.18)	1.36 (1.03, 1.79)	1.13 (0.88, 1.44)	1.13 (0.96, 1.35)	0.94 (0.80, 1.11)	0.92 (0.80, 1.06)	1.15 (0.92, 1.43)	1.25 (0.94, 1.64)	1.00 (0.74, 1.34)	1.03 (0.85, 1.25)	0.97 (0.74, 1.27)	0.99 (0.86, 1.14)	1.21 (0.97, 1.51)	**Sert**					
0.73 (0.57, 0.94)	0.83 (0.65, 1.05)	0.84 (0.65, 1.09)	0.82 (0.63, 1.06)	1.14 (0.82, 1.58)	0.94 (0.70, 1.27)	0.95 (0.74, 1.21)	0.79 (0.62, 1.00)	0.77 (0.61, 0.95)	0.96 (0.72, 1.27)	1.04 (0.75, 1.44)	0.83 (0.59, 1.17)	0.86 (0.67, 1.10)	0.81 (0.58, 1.12)	0.83 (0.66, 1.03)	1.01 (0.76, 1.33)	0.83 (0.66, 1.06)	**Traz**				
0.81 (0.68, 0.96)	0.92 (0.78, 1.07)	0.93 (0.77, 1.13)	0.90 (0.75, 1.09)	1.26 (0.96, 1.65)	1.04 (0.82, 1.32)	1.05 (0.90, 1.22)	0.87 (0.75, 1.02)	0.85 (0.75, 0.96)	1.06 (0.86, 1.31)	1.15 (0.88, 1.51)	0.92 (0.69, 1.23)	0.95 (0.80, 1.14)	0.89 (0.68, 1.17)	0.92 (0.81, 1.04)	1.12 (0.91, 1.38)	0.92 (0.79, 1.08)	1.11 (0.88, 1.40)	**Venl**			
0.74 (0.55, 0.99)	0.83 (0.63, 1.11)	0.84 (0.62, 1.15)	0.82 (0.62, 1.09)	1.14 (0.80, 1.64)	0.95 (0.68, 1.31)	0.95 (0.72, 1.26)	0.79 (0.60, 1.05)	0.77 (0.59, 1.01)	0.96 (0.70, 1.32)	1.04 (0.73, 1.49)	0.84 (0.58, 1.22)	0.87 (0.64, 1.17)	0.81 (0.57, 1.16)	0.83 (0.64, 1.09)	1.01 (0.74, 1.39)	0.84 (0.63, 1.11)	1.00 (0.72, 1.40)	0.91 (0.69, 1.20)	**Vila**		
0.83 (0.67, 1.03)	0.93 (0.76, 1.15)	0.95 (0.75, 1.20)	0.92 (0.74, 1.15)	1.29 (0.95, 1.74)	1.06 (0.82, 1.38)	1.07 (0.89, 1.28)	0.89 (0.73, 1.09)	0.87 (0.72, 1.04)	1.08 (0.85, 1.39)	1.18 (0.87, 1.58)	0.94 (0.68, 1.29)	0.97 (0.78, 1.21)	0.91 (0.68, 1.23)	0.94 (0.78, 1.12)	1.14 (0.89, 1.46)	0.94 (0.77, 1.16)	1.13 (0.87, 1.47)	1.02 (0.85, 1.23)	1.13 (0.83, 1.52)	**Vort**	
0.84 (0.72, 0.97)	0.95 (0.83, 1.08)	0.96 (0.81, 1.14)	0.94 (0.80, 1.09)	1.30 (1.01, 1.68)	1.08 (0.88, 1.33)	1.09 (0.96, 1.23)	0.90 (0.80, 1.02)	0.88 (0.80, 0.96)	1.10 (0.91, 1.33)	1.19 (0.93, 1.53)	0.95 (0.73, 1.26)	0.99 (0.85, 1.15)	0.93 (0.72, 1.19)	0.95 (0.87, 1.03)	1.16 (0.96, 1.40)	0.96 (0.85, 1.08)	1.15 (0.93, 1.42)	1.04 (0.93, 1.15)	1.14 (0.88, 1.47)	1.01 (0.86, 1.19)	**Plac**

OR<1 favour the treatment in the column.

7.2.3 Efficacy continuous

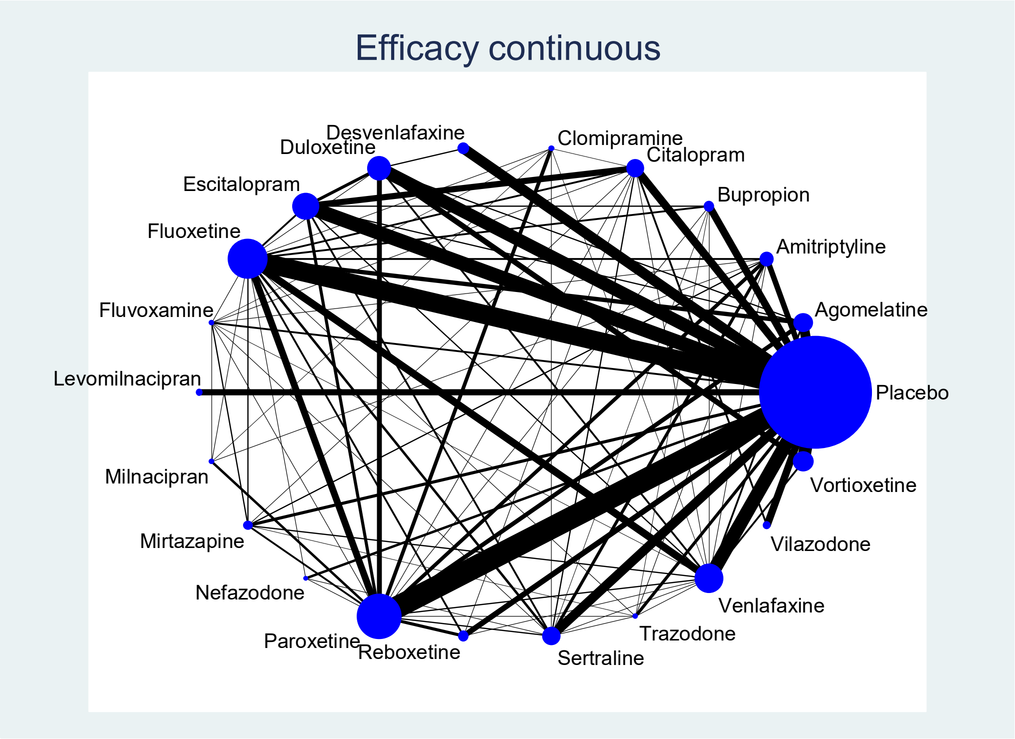

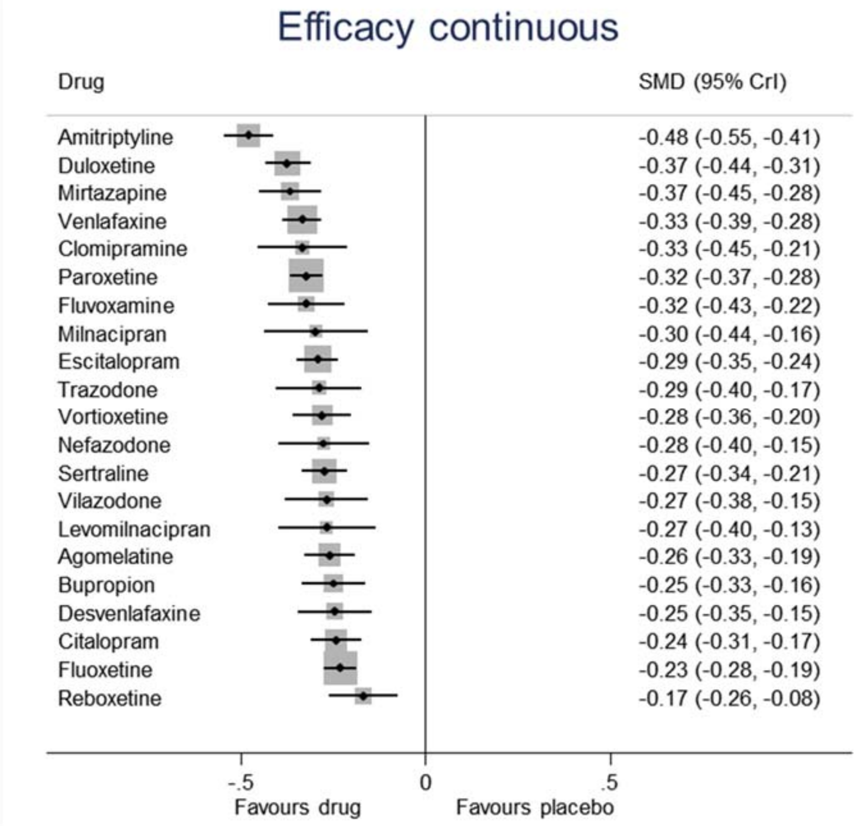

**Agom**																					
0.22 (0.13, 0.31)	**Amit**																				
-0.01 (-0.12, 0.10)	-0.23 (-0.34,-0.12)	**Bupr**																			
-0.02 (-0.11, 0.08)	-0.24 (-0.33,-0.14)	-0.01 (-0.12, 0.10)	**Cita**																		
0.07 (-0.06, 0.21)	-0.15 (-0.28,-0.01)	0.08 (-0.06, 0.23)	0.09 (-0.04, 0.23)	**Clom**																	
-0.01 (-0.14, 0.11)	-0.23 (-0.35,-0.11)	-0.00 (-0.13, 0.13)	0.00 (-0.12, 0.13)	-0.09 (-0.24, 0.07)	**Desv**																
0.11 (0.03, 0.20)	-0.10 (-0.19,-0.02)	0.13 (0.02, 0.23)	0.13 (0.04, 0.22)	0.04 (-0.09, 0.17)	0.13 (0.01, 0.24)	**Dulo**															
0.03 (-0.05, 0.12)	-0.19 (-0.27,-0.10)	0.04 (-0.06, 0.14)	0.05 (-0.03, 0.13)	-0.04 (-0.17, 0.09)	0.05 (-0.07, 0.16)	-0.08 (-0.16,-0.00)	**Esci**														
-0.03 (-0.11, 0.05)	-0.25 (-0.32,-0.18)	-0.02 (-0.11, 0.07)	-0.01 (-0.09, 0.07)	-0.10 (-0.23, 0.02)	-0.02 (-0.13, 0.09)	-0.14 (-0.22,-0.07)	-0.06 (-0.13, 0.01)	**Fluo**													
0.06 (-0.06, 0.19)	-0.16 (-0.27,-0.04)	0.07 (-0.06, 0.21)	0.08 (-0.04, 0.20)	-0.01 (-0.16, 0.14)	0.08 (-0.07, 0.22)	-0.05 (-0.17, 0.07)	0.03 (-0.08, 0.15)	0.09 (-0.02, 0.20)	**Fluv**												
0.01 (-0.14, 0.16)	-0.21 (-0.36,-0.06)	0.02 (-0.14, 0.18)	0.02 (-0.12, 0.18)	-0.07 (-0.25, 0.11)	0.02 (-0.15, 0.19)	-0.11 (-0.25, 0.04)	-0.03 (-0.17, 0.12)	0.04 (-0.10, 0.18)	-0.06 (-0.23, 0.11)	**Levo**											
0.04 (-0.12, 0.19)	-0.18 (-0.33,-0.04)	0.05 (-0.11, 0.21)	0.06 (-0.10, 0.21)	-0.03 (-0.21, 0.14)	0.05 (-0.12, 0.22)	-0.08 (-0.23, 0.07)	0.01 (-0.14, 0.15)	0.07 (-0.07, 0.21)	-0.03 (-0.18, 0.14)	0.03 (-0.16, 0.22)	**Miln**										
0.11 (-0.00, 0.21)	-0.11 (-0.21,-0.01)	0.12 (-0.00, 0.24)	0.12 (0.02, 0.23)	0.03 (-0.11, 0.18)	0.12 (-0.01, 0.25)	-0.01 (-0.11, 0.10)	0.07 (-0.02, 0.17)	0.14 (0.05, 0.23)	0.04 (-0.08, 0.17)	0.10 (-0.06, 0.26)	0.07 (-0.09, 0.23)	**Mirt**									
0.02 (-0.12, 0.16)	-0.20 (-0.34,-0.06)	0.03 (-0.12, 0.18)	0.03 (-0.11, 0.17)	-0.06 (-0.23, 0.11)	0.03 (-0.13, 0.19)	-0.10 (-0.24, 0.04)	-0.02 (-0.15, 0.12)	0.05 (-0.08, 0.17)	-0.05 (-0.21, 0.11)	0.01 (-0.17, 0.19)	-0.02 (-0.21, 0.17)	-0.09 (-0.24, 0.06)	**Nefa**								
0.06 (-0.01, 0.14)	-0.16 (-0.23,-0.08)	0.07 (-0.02, 0.17)	0.08 (0.00, 0.16)	-0.01 (-0.13, 0.11)	0.08 (-0.03, 0.19)	-0.05 (-0.12, 0.02)	0.03 (-0.04, 0.10)	0.09 (0.04, 0.15)	0.00 (-0.11, 0.11)	0.06 (-0.08, 0.20)	0.03 (-0.11, 0.16)	-0.04 (-0.13, 0.05)	0.05 (-0.08, 0.17)	**Paro**							
-0.09 (-0.20, 0.02)	-0.31 (-0.42,-0.20)	-0.08 (-0.20, 0.05)	-0.07 (-0.19, 0.04)	-0.16 (-0.31,-0.01)	-0.08 (-0.21, 0.06)	-0.21 (-0.32,-0.09)	-0.12 (-0.23,-0.02)	-0.06 (-0.16, 0.04)	-0.15 (-0.29,-0.02)	-0.10 (-0.26, 0.07)	-0.13 (-0.29, 0.04)	-0.20 (-0.32,-0.07)	-0.11 (-0.26, 0.05)	-0.15 (-0.25,-0.06)	**Rebo**						
0.01 (-0.08, 0.10)	-0.21 (-0.29,-0.12)	0.03 (-0.08, 0.13)	0.03 (-0.06, 0.12)	-0.06 (-0.19, 0.07)	0.03 (-0.09, 0.14)	-0.10 (-0.19,-0.01)	-0.02 (-0.10, 0.06)	0.04 (-0.03, 0.11)	-0.05 (-0.17, 0.06)	0.01 (-0.14, 0.15)	-0.02 (-0.17, 0.13)	-0.09 (-0.19, 0.01)	-0.00 (-0.14, 0.13)	-0.05 (-0.12, 0.02)	0.10 (-0.01, 0.21)	**Sert**					
0.03 (-0.10, 0.16)	-0.19 (-0.32,-0.06)	0.04 (-0.10, 0.18)	0.05 (-0.09, 0.18)	-0.04 (-0.21, 0.12)	0.04 (-0.11, 0.20)	-0.09 (-0.21, 0.04)	-0.00 (-0.13, 0.12)	0.06 (-0.06, 0.18)	-0.03 (-0.19, 0.12)	0.02 (-0.15, 0.20)	-0.01 (-0.19, 0.17)	-0.08 (-0.22, 0.06)	0.01 (-0.15, 0.18)	-0.03 (-0.15, 0.08)	0.12 (-0.03, 0.27)	0.02 (-0.11, 0.14)	**Traz**				
0.07 (-0.01, 0.16)	-0.15 (-0.23,-0.06)	0.09 (-0.01, 0.18)	0.09 (0.01, 0.18)	0.00 (-0.13, 0.13)	0.09 (-0.03, 0.20)	-0.04 (-0.12, 0.04)	0.04 (-0.03, 0.11)	0.10 (0.04, 0.16)	0.01 (-0.10, 0.12)	0.07 (-0.08, 0.21)	0.04 (-0.11, 0.18)	-0.03 (-0.13, 0.06)	0.06 (-0.08, 0.19)	0.01 (-0.05, 0.07)	0.16 (0.06, 0.27)	0.06 (-0.02, 0.14)	0.04 (-0.08, 0.17)	**Venl**			
0.01 (-0.12, 0.14)	-0.21 (-0.34,-0.08)	0.02 (-0.12, 0.16)	0.03 (-0.10, 0.15)	-0.06 (-0.23, 0.10)	0.02 (-0.13, 0.17)	-0.11 (-0.23, 0.02)	-0.02 (-0.15, 0.10)	0.04 (-0.08, 0.16)	-0.06 (-0.21, 0.10)	0.00 (-0.17, 0.18)	-0.03 (-0.21, 0.15)	-0.10 (-0.24, 0.04)	-0.01 (-0.18, 0.16)	-0.06 (-0.18, 0.06)	0.10 (-0.05, 0.24)	-0.01 (-0.13, 0.12)	-0.02 (-0.18, 0.14)	-0.07 (-0.19, 0.06)	**Vila**		
0.02 (-0.08, 0.12)	-0.20 (-0.30,-0.10)	0.03 (-0.08, 0.15)	0.04 (-0.06, 0.14)	-0.05 (-0.20, 0.09)	0.03 (-0.09, 0.16)	-0.09 (-0.18,-0.00)	-0.01 (-0.11, 0.08)	0.05 (-0.04, 0.14)	-0.04 (-0.17, 0.08)	0.01 (-0.14, 0.17)	-0.02 (-0.18, 0.14)	-0.09 (-0.20, 0.03)	0.00 (-0.14, 0.15)	-0.04 (-0.13, 0.04)	0.11 (-0.01, 0.23)	0.01 (-0.09, 0.11)	-0.01 (-0.15, 0.13)	-0.05 (-0.14, 0.04)	0.01 (-0.12, 0.15)	**Vort**	
-0.26 (-0.33,-0.19)	-0.48 (-0.55,-0.41)	-0.25 (-0.33,-0.16)	-0.24 (-0.31,-0.17)	-0.33 (-0.45,-0.21)	-0.25 (-0.35,-0.15)	-0.37 (-0.44,-0.31)	-0.29 (-0.35,-0.24)	-0.23 (-0.28,-0.19)	-0.32 (-0.43,-0.22)	-0.27 (-0.40,-0.13)	-0.30 (-0.44,-0.16)	-0.37 (-0.45,-0.28)	-0.28 (-0.40,-0.15)	-0.32 (-0.37,-0.28)	-0.17 (-0.26,-0.08)	-0.27 (-0.34,-0.21)	-0.29 (-0.40,-0.17)	-0.33 (-0.39,-0.28)	-0.27 (-0.38,-0.15)	-0.28 (-0.36,-0.20)	**Placebo**

SMD<0 favours the treatment in the column

7.2.4 Remission

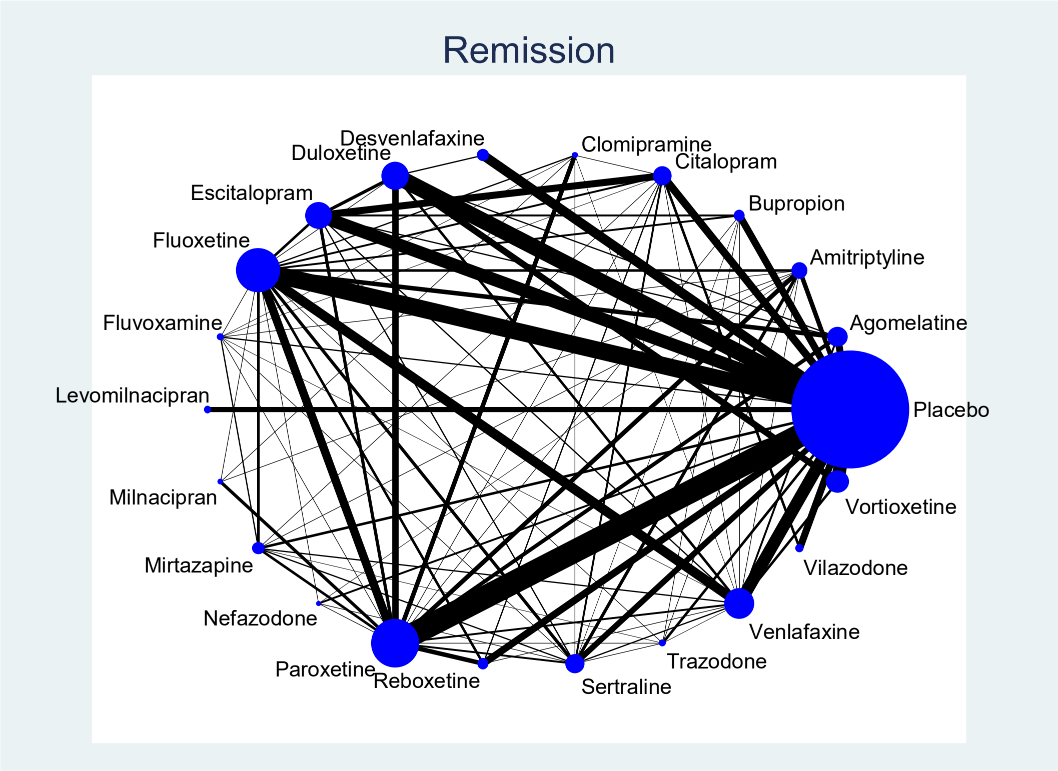

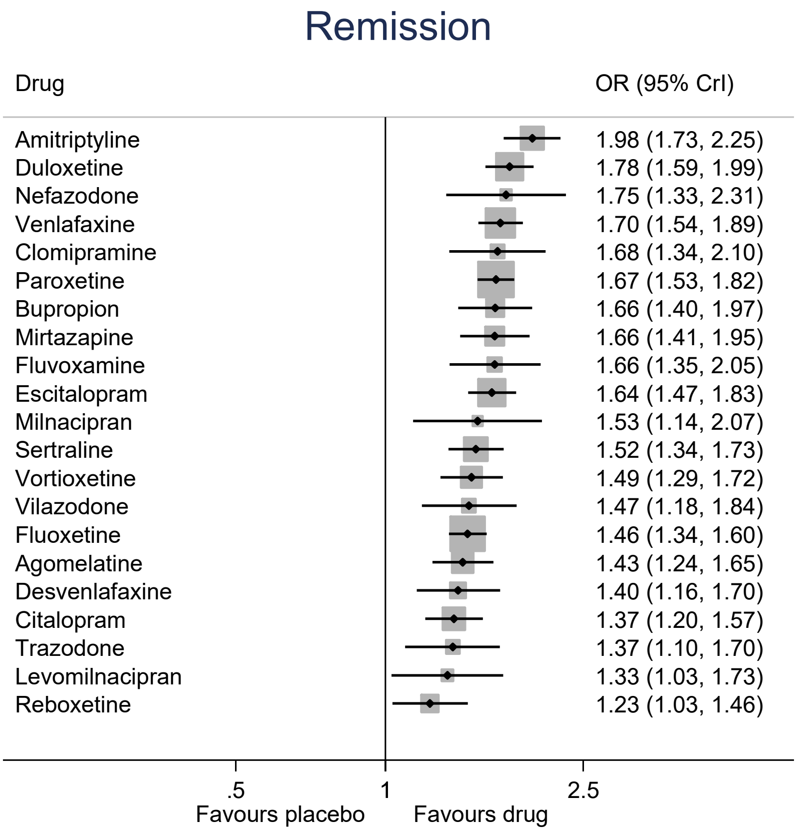

**Agom**																					
0.72 (0.60, 0.87)	**Amit**																				
0.86 (0.69, 1.07)	1.19 (0.96, 1.47)	**Bupr**																			
1.04 (0.86, 1.26)	1.44 (1.20, 1.72)	1.21 (0.98, 1.50)	**Cita**																		
0.85 (0.66, 1.10)	1.17 (0.92, 1.51)	0.99 (0.75, 1.31)	0.82 (0.64, 1.05)	**Clom**																	
1.02 (0.81, 1.30)	1.41 (1.12, 1.78)	1.19 (0.92, 1.54)	0.98 (0.77, 1.24)	1.20 (0.89, 1.61)	**Desv**																
0.80 (0.68, 0.96)	1.11 (0.94, 1.31)	0.94 (0.76, 1.14)	0.77 (0.65, 0.91)	0.95 (0.74, 1.21)	0.79 (0.63, 0.98)	**Dulo**															
0.87 (0.74, 1.03)	1.21 (1.02, 1.42)	1.02 (0.84, 1.23)	0.84 (0.73, 0.97)	1.03 (0.80, 1.31)	0.86 (0.68, 1.07)	1.09 (0.94, 1.26)	**Esci**														
0.98 (0.84, 1.14)	1.35 (1.17, 1.55)	1.14 (0.94, 1.37)	0.94 (0.81, 1.09)	1.15 (0.92, 1.44)	0.96 (0.77, 1.18)	1.21 (1.06, 1.39)	1.12 (0.98, 1.27)	**Fluo**													
0.86 (0.67, 1.10)	1.19 (0.95, 1.50)	1.00 (0.77, 1.31)	0.83 (0.65, 1.05)	1.01 (0.75, 1.36)	0.84 (0.63, 1.12)	1.07 (0.85, 1.36)	0.99 (0.78, 1.24)	0.88 (0.71, 1.09)	**Fluv**												
1.07 (0.80, 1.44)	1.48 (1.11, 1.98)	1.25 (0.92, 1.70)	1.03 (0.77, 1.38)	1.26 (0.90, 1.77)	1.05 (0.76, 1.45)	1.33 (1.01, 1.77)	1.23 (0.93, 1.63)	1.10 (0.84, 1.45)	1.25 (0.89, 1.74)	**Levo**											
0.93 (0.68, 1.29)	1.29 (0.95, 1.76)	1.08 (0.77, 1.53)	0.90 (0.65, 1.24)	1.10 (0.78, 1.55)	0.91 (0.64, 1.30)	1.16 (0.85, 1.59)	1.07 (0.78, 1.46)	0.95 (0.71, 1.29)	1.08 (0.76, 1.55)	0.87 (0.59, 1.29)	**Miln**										
0.86 (0.70, 1.06)	1.19 (0.98, 1.44)	1.00 (0.79, 1.26)	0.83 (0.68, 1.01)	1.01 (0.78, 1.32)	0.84 (0.66, 1.09)	1.07 (0.88, 1.30)	0.99 (0.82, 1.19)	0.88 (0.75, 1.04)	1.00 (0.79, 1.27)	0.80 (0.59, 1.09)	0.92 (0.66, 1.28)	**Mirt**									
0.82 (0.60, 1.11)	1.13 (0.84, 1.52)	0.95 (0.69, 1.32)	0.79 (0.58, 1.07)	0.96 (0.68, 1.36)	0.80 (0.57, 1.12)	1.02 (0.76, 1.37)	0.94 (0.70, 1.26)	0.84 (0.63, 1.11)	0.95 (0.67, 1.34)	0.76 (0.52, 1.11)	0.88 (0.59, 1.31)	0.95 (0.69, 1.30)	**Nefa**								
0.86 (0.74, 1.00)	1.18 (1.03, 1.36)	1.00 (0.83, 1.20)	0.82 (0.71, 0.95)	1.01 (0.81, 1.25)	0.84 (0.68, 1.03)	1.07 (0.94, 1.21)	0.98 (0.86, 1.12)	0.88 (0.79, 0.97)	0.99 (0.80, 1.24)	0.80 (0.61, 1.05)	0.92 (0.69, 1.23)	0.99 (0.84, 1.18)	1.05 (0.79, 1.40)	**Paro**							
1.16 (0.93, 1.45)	1.61 (1.30, 1.98)	1.35 (1.06, 1.72)	1.12 (0.91, 1.38)	1.37 (1.04, 1.80)	1.14 (0.88, 1.47)	1.45 (1.18, 1.77)	1.33 (1.09, 1.63)	1.19 (0.99, 1.43)	1.35 (1.03, 1.76)	1.08 (0.79, 1.48)	1.25 (0.89, 1.75)	1.35 (1.07, 1.70)	1.42 (1.03, 1.97)	1.36 (1.13, 1.63)	**Rebo**						
0.94 (0.78, 1.13)	1.30 (1.11, 1.52)	1.09 (0.89, 1.35)	0.90 (0.76, 1.07)	1.11 (0.87, 1.40)	0.92 (0.73, 1.16)	1.17 (0.99, 1.38)	1.08 (0.92, 1.26)	0.96 (0.84, 1.11)	1.09 (0.87, 1.38)	0.88 (0.66, 1.17)	1.01 (0.73, 1.38)	1.09 (0.90, 1.32)	1.15 (0.85, 1.55)	1.10 (0.95, 1.26)	0.81 (0.66, 1.00)	**Sert**					
1.05 (0.81, 1.35)	1.44 (1.14, 1.84)	1.22 (0.93, 1.60)	1.01 (0.78, 1.29)	1.23 (0.91, 1.67)	1.03 (0.77, 1.38)	1.30 (1.02, 1.66)	1.20 (0.94, 1.53)	1.07 (0.85, 1.35)	1.21 (0.90, 1.64)	0.98 (0.69, 1.37)	1.12 (0.78, 1.62)	1.21 (0.93, 1.59)	1.28 (0.90, 1.82)	1.22 (0.97, 1.53)	0.90 (0.68, 1.19)	1.11 (0.87, 1.42)	**Traz**				
0.84 (0.71, 0.99)	1.16 (0.99, 1.35)	0.98 (0.81, 1.18)	0.81 (0.69, 0.94)	0.99 (0.78, 1.25)	0.82 (0.66, 1.02)	1.04 (0.91, 1.20)	0.96 (0.84, 1.11)	0.86 (0.77, 0.96)	0.97 (0.78, 1.22)	0.78 (0.59, 1.03)	0.90 (0.66, 1.22)	0.97 (0.82, 1.16)	1.03 (0.77, 1.38)	0.98 (0.87, 1.10)	0.72 (0.59, 0.88)	0.89 (0.77, 1.04)	0.80 (0.63, 1.01)	**Venl**			
0.97 (0.75, 1.26)	1.34 (1.04, 1.73)	1.13 (0.86, 1.49)	0.93 (0.73, 1.19)	1.14 (0.84, 1.56)	0.95 (0.71, 1.27)	1.21 (0.94, 1.54)	1.11 (0.87, 1.42)	0.99 (0.79, 1.26)	1.13 (0.83, 1.53)	0.90 (0.64, 1.27)	1.04 (0.72, 1.50)	1.13 (0.86, 1.48)	1.19 (0.84, 1.69)	1.13 (0.90, 1.43)	0.83 (0.63, 1.10)	1.03 (0.80, 1.33)	0.93 (0.68, 1.26)	1.16 (0.91, 1.47)	**Vila**		
0.96 (0.79, 1.17)	1.32 (1.09, 1.61)	1.12 (0.89, 1.40)	0.92 (0.76, 1.12)	1.13 (0.87, 1.47)	0.94 (0.74, 1.19)	1.19 (1.01, 1.41)	1.10 (0.92, 1.31)	0.98 (0.83, 1.16)	1.11 (0.86, 1.44)	0.89 (0.66, 1.20)	1.03 (0.74, 1.43)	1.11 (0.90, 1.38)	1.17 (0.86, 1.61)	1.12 (0.95, 1.32)	0.82 (0.66, 1.03)	1.02 (0.84, 1.24)	0.92 (0.71, 1.19)	1.14 (0.97, 1.35)	0.99 (0.76, 1.28)	**Vort**	
1.43 (1.24, 1.65)	1.98 (1.73, 2.25)	1.66 (1.40, 1.97)	1.37 (1.20, 1.57)	1.68 (1.34, 2.10)	1.40 (1.16, 1.70)	1.78 (1.59, 1.99)	1.64 (1.47, 1.83)	1.46 (1.34, 1.60)	1.66 (1.35, 2.05)	1.33 (1.03, 1.73)	1.53 (1.14, 2.07)	1.66 (1.41, 1.95)	1.75 (1.33, 2.31)	1.67 (1.53, 1.82)	1.23 (1.03, 1.46)	1.52 (1.34, 1.73)	1.37 (1.10, 1.70)	1.70 (1.54, 1.89)	1.47 (1.18, 1.84)	1.49 (1.29, 1.72)	**Place bo**

OR>1 favours the treatment in the column

7.2.5 Dropouts due to adverse events

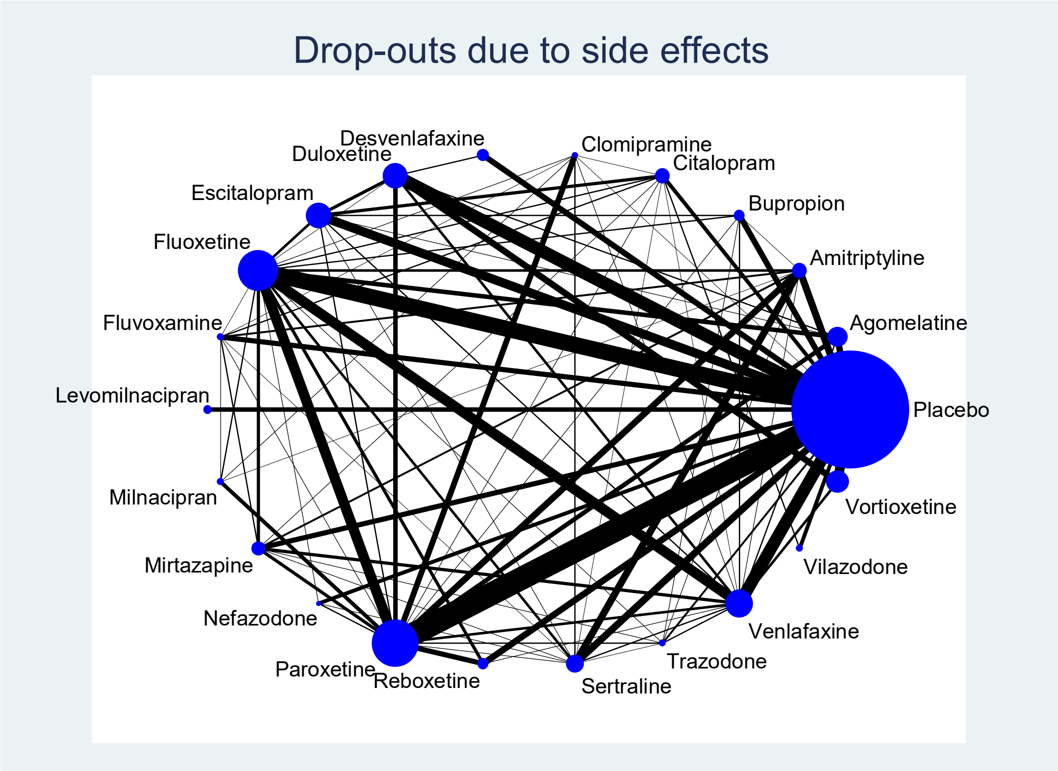

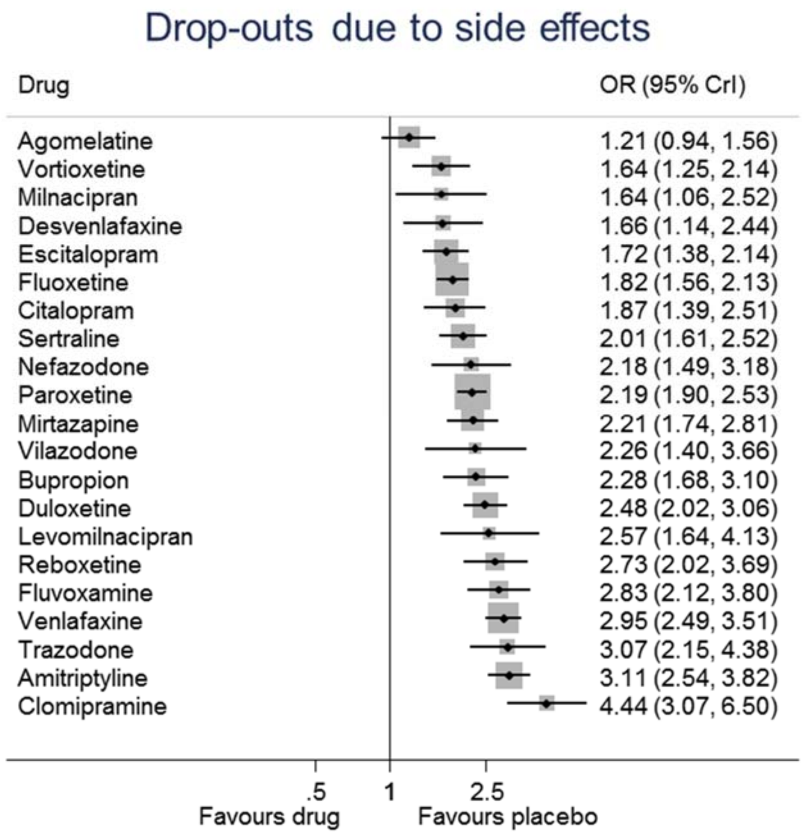

**Agom**																					
0.39 (0.28, 0.53)	**Amit**																				
0.53 (0.36, 0.78)	1.36 (0.95, 1.95)	**Bupr**																			
0.65 (0.44, 0.94)	1.66 (1.18, 2.36)	1.22 (0.80, 1.86)	**Cita**																		
0.27 (0.17, 0.42)	0.70 (0.46, 1.05)	0.51 (0.32, 0.83)	0.42 (0.26, 0.66)	**Clom**																	
0.73 (0.46, 1.14)	1.87 (1.22, 2.87)	1.37 (0.84, 2.23)	1.12 (0.69, 1.81)	2.67 (1.57, 4.54)	**Desv**																
0.49 (0.36, 0.66)	1.25 (0.95, 1.66)	0.92 (0.64, 1.33)	0.75 (0.53, 1.07)	1.79 (1.18, 2.74)	0.67 (0.44, 1.02)	**Dulo**															
0.70 (0.51, 0.97)	1.81 (1.35, 2.41)	1.33 (0.92, 1.90)	1.09 (0.78, 1.51)	2.58 (1.69, 3.96)	0.96 (0.63, 1.50)	1.44 (1.09, 1.91)	**Esci**														
0.66 (0.50, 0.87)	1.70 (1.36, 2.14)	1.25 (0.90, 1.74)	1.02 (0.75, 1.40)	2.43 (1.67, 3.58)	0.91 (0.61, 1.37)	1.36 (1.06, 1.74)	0.94 (0.74, 1.21)	**Fluo**													
0.43 (0.29, 0.62)	1.10 (0.79, 1.52)	0.81 (0.53, 1.22)	0.66 (0.45, 0.97)	1.57 (1.00, 2.49)	0.59 (0.37, 0.95)	0.88 (0.62, 1.25)	0.61 (0.43, 0.87)	0.65 (0.47, 0.89)	**Fluv**												
0.47 (0.27, 0.79)	1.21 (0.72, 1.99)	0.89 (0.50, 1.54)	0.73 (0.42, 1.25)	1.73 (0.95, 3.12)	0.65 (0.35, 1.17)	0.97 (0.58, 1.59)	0.67 (0.40, 1.11)	0.71 (0.43, 1.15)	1.10 (0.63, 1.89)	**Levo**											
0.74 (0.45, 1.21)	1.90 (1.20, 2.99)	1.39 (0.82, 2.36)	1.14 (0.68, 1.91)	2.71 (1.61, 4.63)	1.01 (0.57, 1.81)	1.51 (0.95, 2.43)	1.05 (0.65, 1.69)	1.11 (0.72, 1.73)	1.72 (1.06, 2.83)	1.57 (0.84, 2.98)	**Miln**										
0.55 (0.39, 0.76)	1.41 (1.06, 1.87)	1.03 (0.71, 1.51)	0.84 (0.59, 1.21)	2.01 (1.32, 3.09)	0.75 (0.48, 1.18)	1.12 (0.83, 1.53)	0.78 (0.57, 1.06)	0.82 (0.64, 1.07)	1.28 (0.91, 1.81)	1.16 (0.70, 1.98)	0.74 (0.46, 1.19)	**Mirt**									
0.55 (0.35, 0.87)	1.43 (0.94, 2.18)	1.05 (0.64, 1.70)	0.86 (0.53, 1.38)	2.04 (1.21, 3.46)	0.76 (0.45, 1.31)	1.14 (0.74, 1.76)	0.79 (0.51, 1.22)	0.84 (0.56, 1.25)	1.30 (0.81, 2.09)	1.18 (0.65, 2.16)	0.75 (0.43, 1.32)	1.01 (0.65, 1.58)	**Nefa**								
0.55 (0.42, 0.72)	1.42 (1.15, 1.76)	1.04 (0.75, 1.45)	0.85 (0.62, 1.17)	2.02 (1.41, 2.94)	0.76 (0.51, 1.14)	1.13 (0.90, 1.43)	0.78 (0.61, 1.00)	0.83 (0.70, 0.99)	1.29 (0.95, 1.75)	1.17 (0.73, 1.93)	0.75 (0.49, 1.14)	1.01 (0.78, 1.30)	0.99 (0.67, 1.47)	**Paro**							
0.44 (0.30, 0.65)	1.14 (0.80, 1.62)	0.84 (0.55, 1.28)	0.68 (0.45, 1.04)	1.63 (1.03, 2.61)	0.61 (0.38, 0.99)	0.91 (0.64, 1.30)	0.63 (0.44, 0.91)	0.67 (0.49, 0.92)	1.04 (0.69, 1.57)	0.94 (0.55, 1.65)	0.60 (0.36, 1.00)	0.81 (0.56, 1.18)	0.80 (0.49, 1.29)	0.80 (0.59, 1.10)	**Rebo**						
0.60 (0.43, 0.83)	1.54 (1.19, 2.01)	1.13 (0.78, 1.65)	0.93 (0.65, 1.33)	2.20 (1.47, 3.32)	0.83 (0.53, 1.29)	1.23 (0.91, 1.67)	0.85 (0.63, 1.15)	0.91 (0.71, 1.16)	1.40 (0.99, 2.00)	1.28 (0.77, 2.16)	0.81 (0.50, 1.30)	1.10 (0.81, 1.49)	1.08 (0.71, 1.65)	1.09 (0.85, 1.39)	1.36 (0.94, 1.96)	**Sert**					
0.39 (0.26, 0.60)	1.01 (0.69, 1.49)	0.74 (0.48, 1.16)	0.61 (0.39, 0.96)	1.45 (0.88, 2.41)	0.54 (0.32, 0.91)	0.81 (0.54, 1.22)	0.56 (0.37, 0.85)	0.59 (0.41, 0.86)	0.92 (0.59, 1.45)	0.84 (0.47, 1.52)	0.53 (0.31, 0.93)	0.72 (0.48, 1.08)	0.71 (0.42, 1.19)	0.72 (0.50, 1.03)	0.89 (0.56, 1.41)	0.66 (0.44, 0.98)	**Traz**				
0.41 (0.31, 0.54)	1.05 (0.82, 1.35)	0.77 (0.55, 1.08)	0.63 (0.46, 0.88)	1.50 (1.02, 2.24)	0.56 (0.37, 0.85)	0.84 (0.65, 1.08)	0.58 (0.45, 0.75)	0.62 (0.51, 0.75)	0.96 (0.69, 1.33)	0.87 (0.54, 1.44)	0.56 (0.35, 0.87)	0.75 (0.57, 0.98)	0.74 (0.49, 1.11)	0.74 (0.61, 0.91)	0.92 (0.66, 1.29)	0.68 (0.52, 0.89)	1.04 (0.71, 1.52)	**Venl**			
0.53 (0.31, 0.92)	1.38 (0.82, 2.31)	1.01 (0.57, 1.78)	0.83 (0.49, 1.40)	1.96 (1.07, 3.61)	0.74 (0.40, 1.35)	1.10 (0.65, 1.85)	0.76 (0.45, 1.28)	0.81 (0.49, 1.33)	1.25 (0.71, 2.18)	1.14 (0.59, 2.23)	0.73 (0.38, 1.38)	0.98 (0.57, 1.66)	0.96 (0.52, 1.77)	0.97 (0.59, 1.59)	1.21 (0.69, 2.11)	0.89 (0.52, 1.51)	1.36 (0.75, 2.46)	1.31 (0.79, 2.17)	**Vila**		
0.74 (0.51, 1.06)	1.90 (1.36, 2.67)	1.39 (0.93, 2.10)	1.14 (0.77, 1.70)	2.71 (1.72, 4.33)	1.02 (0.64, 1.62)	1.52 (1.13, 2.05)	1.05 (0.75, 1.48)	1.12 (0.82, 1.52)	1.73 (1.17, 2.58)	1.57 (0.93, 2.72)	1.00 (0.60, 1.66)	1.35 (0.94, 1.93)	1.33 (0.83, 2.12)	1.34 (1.00, 1.81)	1.67 (1.12, 2.50)	1.23 (0.87, 1.75)	1.88 (1.20, 2.94)	1.81 (1.33, 2.45)	1.38 (0.80, 2.40)	**Vort**	
1.21 (0.94, 1.56)	3.11 (2.54, 3.82)	2.28 (1.68, 3.10)	1.87 (1.39, 2.51)	4.44 (3.07, 6.50)	1.66 (1.14, 2.44)	2.48 (2.02, 3.06)	1.72 (1.38, 2.14)	1.82 (1.56, 2.13)	2.83 (2.12, 3.80)	2.57 (1.64, 4.13)	1.64 (1.06, 2.52)	2.21 (1.74, 2.81)	2.18 (1.49, 3.18)	2.19 (1.90, 2.53)	2.73 (2.02, 3.69)	2.01 (1.61, 2.52)	3.07 (2.15, 4.38)	2.95 (2.49, 3.51)	2.26 (1.40, 3.66)	1.64 (1.25, 2.14)	**Placebo**

Values<1 favour the treatment in the column

7.3 *Assessment of transitivity*

The following characteristics have been evaluated in all trials included in the network irrespectively of the outcome being reported.

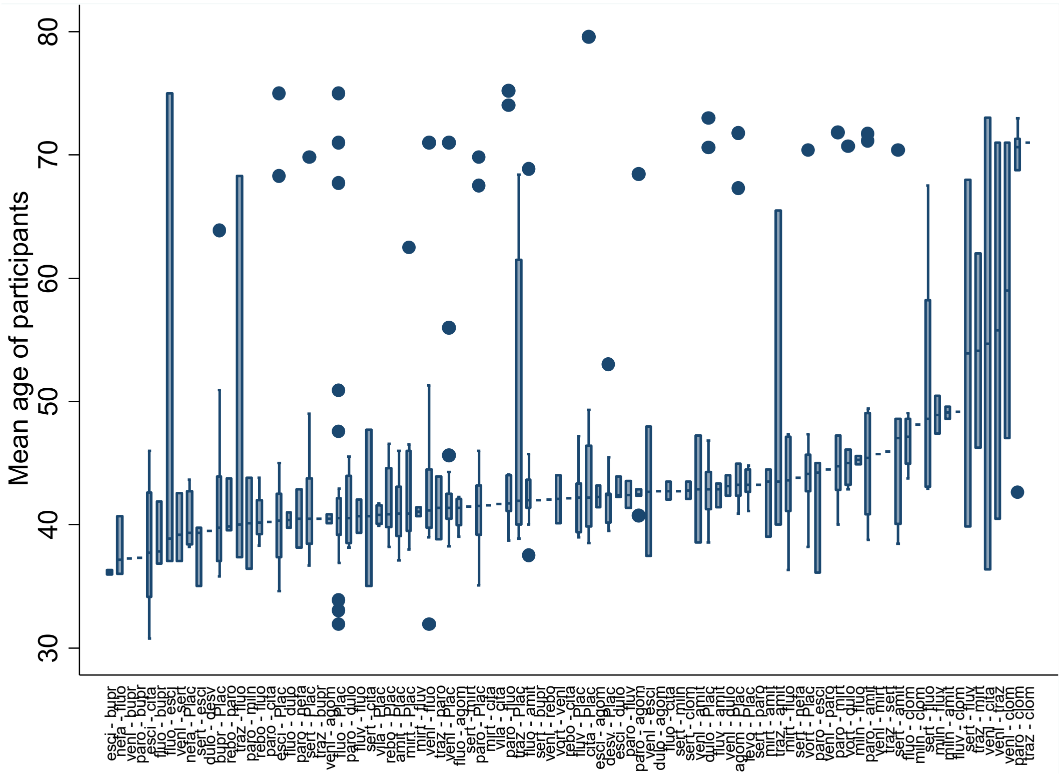

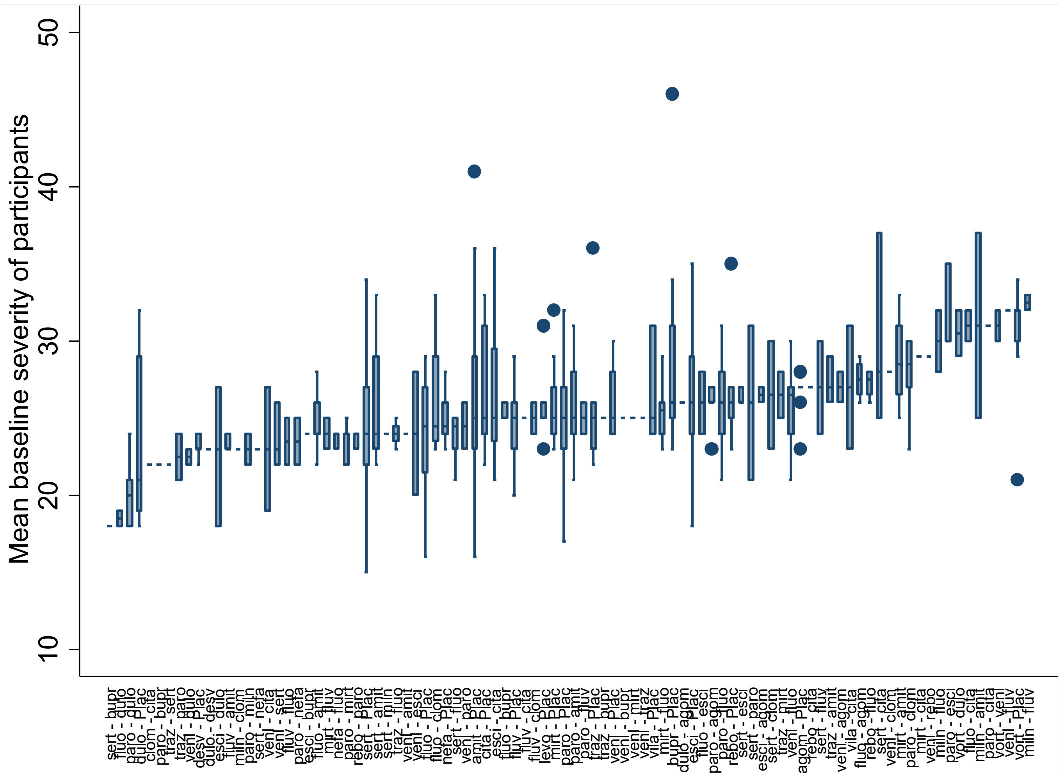

Most of the comparisons had similar mean age and baseline severity but there were a few comparisons which had relatively low or high age or baseline severity means. Meta-regressions of mean age and subgroup analyses by baseline severity, however, did not show that they impacted on the network estimates, although results from such analyses might suffer from ecological bias.

7.4 Evaluation of inconsistency

7.4.1 Response

7.4.1.1 Loop-specific approach

Loops With Evidence of Statistical Inconsistency:

Loop	IF	seIF	z_value	p_value	CI_95	Loop_Heterog_tau2
cita-fluv-venl	1.243	0.599	2.077	0.038	(0.07,2.42)	0.000
clom-fluo-traz	1.161	0.524	2.215	0.027	(0.13,2.19)	0.000
cita-clom-fluo	1.031	0.470	2.192	0.028	(0.11,1.95)	0.000
Plac-fluv-venl	0.992	0.457	2.168	0.030	(0.10,1.89)	0.010
agom-paro-venl	0.912	0.310	2.943	0.003	(0.30,1.52)	0.021
clom-fluo-sert	0.801	0.356	2.249	0.025	(0.10,1.50)	0.000
mirt-paro-venl	0.795	0.345	2.301	0.021	(0.12,1.47)	0.033
cita-fluo-venl	0.739	0.370	1.999	0.046	(0.01,1.46)	0.008
agom-dulo-venl	0.690	0.319	2.160	0.031	(0.06,1.32)	0.000
dulo-venl-vort	0.601	0.265	2.268	0.023	(0.08,1.12)	0.015
Plac-amit-fluo	0.561	0.213	2.628	0.009	(0.14,0.98)	0.075
bupr-fluo-venl	0.546	0.272	2.010	0.044	(0.01,1.08)	0.000
amit-fluo-sert	0.525	0.220	2.383	0.017	(0.09,0.96)	0.000
agom-fluo-venl	0.478	0.229	2.087	0.037	(0.03,0.93)	0.000
Plac-dulo-esci	0.463	0.193	2.405	0.016	(0.09,0.84)	0.035
Plac-amit-paro	0.388	0.155	2.498	0.012	(0.08,0.69)	0.021
Plac-cita-esci	0.357	0.142	2.520	0.012	(0.08,0.63)	0.010

Loops Without Evidence of Statistical Inconsistency:

Loop	IF	seIF	z_value	p_value	CI_95	Loop_Heterog_tau2
cita-clom-venl	1.891	1.255	1.507	0.132	(0.00,4.35)	0.492
bupr-sert-traz	1.385	1.151	1.203	0.229	(0.00,3.64)	0.000
fluo-miln-sert	1.242	0.964	1.288	0.198	(0.00,3.13)	0.019
clom-fluo-fluv	1.149	0.613	1.873	0.061	(0.00,2.35)	0.000
cita-sert-venl	1.118	0.892	1.253	0.210	(0.00,2.87)	0.284
clom-fluv-sert	1.102	0.650	1.695	0.090	(0.00,2.38)	0.000
bupr-fluo-traz	1.090	0.595	1.833	0.067	(0.00,2.26)	0.047
fluv-mirt-venl	1.037	0.749	1.385	0.166	(0.00,2.50)	0.126
cita-clom-paro	1.001	0.810	1.236	0.216	(0.00,2.59)	0.137
amit-fluv-venl	0.933	0.985	0.948	0.343	(0.00,2.86)	0.237
cita-paro-venl	0.916	0.923	0.992	0.321	(0.00,2.73)	0.206
Plac-bupr-traz	0.892	0.580	1.538	0.124	(0.00,2.03)	0.094
miln-paro-sert	0.872	0.939	0.928	0.353	(0.00,2.71)	0.000
cita-clom-mirt-traz	0.774	0.691	1.119	0.263	(0.00,2.13)	0.000
fluo-fluv-sert	0.755	0.407	1.856	0.063	(0.00,1.55)	0.000
fluo-sert-traz	0.750	0.525	1.430	0.153	(0.00,1.78)	0.014
clom-traz-venl	0.737	0.588	1.254	0.210	(0.00,1.89)	0.000
cita-fluv-sert	0.736	0.928	0.793	0.428	(0.00,2.55)	0.213
fluo-fluv-miln	0.719	0.432	1.665	0.096	(0.00,1.57)	0.010
cita-esci-venl	0.706	0.498	1.418	0.156	(0.00,1.68)	0.077
fluo-fluv-venl	0.701	0.491	1.429	0.153	(0.00,1.66)	0.000
Plac-cita-venl	0.700	0.359	1.950	0.051	(0.00,1.40)	0.026
paro-rebo-venl	0.671	0.367	1.829	0.067	(0.00,1.39)	0.000
fluv-paro-sert	0.670	0.467	1.435	0.151	(0.00,1.58)	0.000
agom-dulo-esci	0.646	0.406	1.591	0.112	(0.00,1.44)	0.031

esci-paro-venl	0.645	0.340	1.899	0.058	(0.00,1.31)	0.027
fluo-paro-traz	0.643	0.452	1.422	0.155	(0.00,1.53)	0.056
clom-miln-sert	0.641	1.026	0.624	0.532	(0.00,2.65)	0.000
Plac-paro-traz	0.620	0.320	1.936	0.053	(0.00,1.25)	0.023
amit-miln-sert	0.616	1.090	0.565	0.572	(0.00,2.75)	0.096
clom-fluv-paro	0.616	0.733	0.840	0.401	(0.00,2.05)	0.086
fluv-mirt-sert	0.615	0.799	0.769	0.442	(0.00,2.18)	0.225
bupr-paro-venl	0.606	0.502	1.207	0.227	(0.00,1.59)	0.023
cita-esci-paro	0.597	0.347	1.720	0.086	(0.00,1.28)	0.026
cita-fluo-mirt	0.594	0.509	1.167	0.243	(0.00,1.59)	0.040
amit-fluv-miln	0.587	0.881	0.666	0.505	(0.00,2.31)	0.308
amit-paro-venl	0.582	0.419	1.390	0.164	(0.00,1.40)	0.071
amit-sert-traz	0.568	0.529	1.073	0.283	(0.00,1.61)	0.043
nefa-paro-sert	0.546	0.566	0.964	0.335	(0.00,1.65)	0.039
amit-clom-miln-traz	0.517	0.928	0.557	0.578	(0.00,2.34)	0.143
Plac-cita-rebo	0.502	0.414	1.213	0.225	(0.00,1.31)	0.068
esci-sert-venl	0.496	0.399	1.243	0.214	(0.00,1.28)	0.032
Plac-sert-traz	0.488	0.523	0.931	0.352	(0.00,1.51)	0.054
cita-mirt-sert	0.487	1.388	0.351	0.726	(0.00,3.21)	0.463
bupr-fluo-sert	0.478	1.034	0.462	0.644	(0.00,2.51)	0.000
fluo-nefa-sert	0.470	0.464	1.013	0.311	(0.00,1.38)	0.000
clom-fluo-paro	0.457	0.333	1.371	0.170	(0.00,1.11)	0.055
fluo-rebo-venl	0.452	0.352	1.283	0.199	(0.00,1.14)	0.000
cita-clom-sert	0.448	0.886	0.506	0.613	(0.00,2.18)	0.159
fluv-miln-paro	0.446	0.429	1.041	0.298	(0.00,1.29)	0.000
Plac-agom-venl	0.441	0.265	1.666	0.096	(0.00,0.96)	0.038
fluo-traz-venl	0.438	0.379	1.155	0.248	(0.00,1.18)	0.000
Plac-traz-venl	0.436	0.362	1.202	0.229	(0.00,1.15)	0.045
Plac-cita-mirt	0.435	0.405	1.074	0.283	(0.00,1.23)	0.014
clom-fluv-mirt-traz	0.409	0.906	0.451	0.652	(0.00,2.18)	0.120
cita-fluo-paro	0.406	0.411	0.987	0.324	(0.00,1.21)	0.047
Plac-cita-fluv	0.403	0.331	1.217	0.224	(0.00,1.05)	0.000
Plac-esci-venl	0.401	0.238	1.685	0.092	(0.00,0.87)	0.016
bupr-sert-venl	0.391	1.057	0.370	0.712	(0.00,2.46)	0.000
Plac-cita-paro	0.388	0.280	1.389	0.165	(0.00,0.94)	0.010
amit-clom-miln-venl	0.383	0.841	0.455	0.649	(0.00,2.03)	0.122
amit-traz-venl	0.378	0.428	0.884	0.377	(0.00,1.22)	0.000
Plac-mirt-traz	0.374	0.423	0.882	0.378	(0.00,1.20)	0.087
dulo-paro-venl	0.372	0.297	1.250	0.211	(0.00,0.95)	0.029
Plac-bupr-venl	0.369	0.324	1.140	0.254	(0.00,1.00)	0.040
cita-fluo-rebo	0.367	0.307	1.193	0.233	(0.00,0.97)	0.000
amit-fluo-traz	0.363	0.380	0.957	0.339	(0.00,1.11)	0.000
agom-esci-paro	0.363	0.305	1.188	0.235	(0.00,0.96)	0.019
Plac-fluv-paro	0.362	0.335	1.079	0.281	(0.00,1.02)	0.004
Plac-paro-venl	0.361	0.189	1.912	0.056	(0.00,0.73)	0.010
mirt-traz-venl	0.353	0.388	0.909	0.363	(0.00,1.11)	0.000
amit-paro-traz	0.351	0.445	0.790	0.430	(0.00,1.22)	0.067
cita-fluv-paro	0.340	0.490	0.695	0.487	(0.00,1.30)	0.000
cita-paro-sert	0.340	0.797	0.427	0.669	(0.00,1.90)	0.144
Plac-amit-mirt	0.338	0.290	1.165	0.244	(0.00,0.91)	0.048
Plac-amit-sert	0.329	0.178	1.850	0.064	(0.00,0.68)	0.020
Plac-fluo-fluv	0.324	0.375	0.865	0.387	(0.00,1.06)	0.089
Plac-mirt-venl	0.324	0.246	1.319	0.187	(0.00,0.81)	0.020
fluo-sert-venl	0.323	0.239	1.347	0.178	(0.00,0.79)	0.000
cita-esci-sert	0.321	0.419	0.766	0.444	(0.00,1.14)	0.067
amit-paro-sert	0.319	0.336	0.951	0.342	(0.00,0.98)	0.071
agom-esci-fluo	0.313	0.284	1.102	0.270	(0.00,0.87)	0.000
Plac-nefa-paro	0.309	0.316	0.979	0.328	(0.00,0.93)	0.013
Plac-dulo-vort	0.308	0.176	1.747	0.081	(0.00,0.65)	0.052
cita-fluv-mirt	0.306	0.508	0.602	0.547	(0.00,1.30)	0.000
amit-fluo-venl	0.304	0.309	0.982	0.326	(0.00,0.91)	0.000
clom-sert-venl	0.293	0.429	0.684	0.494	(0.00,1.13)	0.000
fluo-mirt-traz	0.291	0.480	0.607	0.544	(0.00,1.23)	0.057
fluo-mirt-venl	0.289	0.231	1.250	0.211	(0.00,0.74)	0.000
Plac-clom-fluv-traz	0.287	0.768	0.374	0.708	(0.00,1.79)	0.050
amit-clom-fluv-traz	0.285	0.935	0.305	0.760	(0.00,2.12)	0.120
amit-fluv-sert	0.276	0.540	0.511	0.610	(0.00,1.34)	0.092
cita-fluo-sert	0.275	0.365	0.754	0.451	(0.00,0.99)	0.029
fluo-mirt-paro	0.269	0.278	0.967	0.333	(0.00,0.81)	0.059
Plac-fluv-mirt	0.268	0.309	0.867	0.386	(0.00,0.87)	0.033
Plac-rebo-venl	0.266	0.452	0.587	0.557	(0.00,1.15)	0.062
fluo-nefa-paro	0.264	0.482	0.548	0.584	(0.00,1.21)	0.048
bupr-esci-fluo	0.264	0.273	0.966	0.334	(0.00,0.80)	0.000
clom-fluv-venl	0.262	0.727	0.360	0.719	(0.00,1.69)	0.000
fluo-paro-venl	0.252	0.215	1.169	0.242	(0.00,0.67)	0.014
Plac-fluo-sert	0.249	0.207	1.200	0.230	(0.00,0.66)	0.074
esci-fluo-venl	0.246	0.261	0.939	0.348	(0.00,0.76)	0.000

fluv-miln-sert	0.243	1.002	0.243	0.808	(0.00,2.21)	0.000
amit-mirt-sert	0.241	0.452	0.533	0.594	(0.00,1.13)	0.065
bupr-fluo-paro	0.234	0.456	0.513	0.608	(0.00,1.13)	0.044
Plac-fluo-rebo	0.227	0.297	0.767	0.443	(0.00,0.81)	0.112
dulo-esci-fluo	0.225	0.369	0.609	0.542	(0.00,0.95)	0.011
clom-fluo-miln	0.219	0.492	0.444	0.657	(0.00,1.18)	0.000
clom-fluv-miln	0.218	0.707	0.308	0.758	(0.00,1.60)	0.000
amit-fluo-mirt	0.212	0.296	0.716	0.474	(0.00,0.79)	0.008
fluo-paro-sert	0.210	0.287	0.732	0.464	(0.00,0.77)	0.034
amit-mirt-traz	0.207	0.386	0.536	0.592	(0.00,0.96)	0.001
Plac-amit-venl	0.204	0.298	0.684	0.494	(0.00,0.79)	0.018
Plac-fluv-sert	0.200	0.346	0.579	0.563	(0.00,0.88)	0.008
Plac-dulo-paro	0.196	0.143	1.370	0.171	(0.00,0.48)	0.029
Plac-bupr-fluo	0.188	0.289	0.651	0.515	(0.00,0.75)	0.103
clom-fluo-venl	0.186	0.375	0.496	0.620	(0.00,0.92)	0.000
bupr-paro-traz	0.185	0.558	0.332	0.740	(0.00,1.28)	0.000
fluo-miln-paro	0.183	0.339	0.540	0.589	(0.00,0.85)	0.049
amit-mirt-venl	0.182	0.380	0.479	0.632	(0.00,0.93)	0.002
clom-paro-sert	0.180	0.483	0.373	0.709	(0.00,1.13)	0.069
Plac-dulo-venl	0.179	0.223	0.801	0.423	(0.00,0.62)	0.036
cita-fluo-fluv	0.169	0.417	0.405	0.685	(0.00,0.99)	0.000
dulo-fluo-paro	0.168	0.368	0.457	0.648	(0.00,0.89)	0.047
Plac-amit-fluv	0.167	0.295	0.564	0.572	(0.00,0.75)	0.021
fluo-fluv-mirt	0.161	0.507	0.319	0.750	(0.00,1.15)	0.098
Plac-amit-traz	0.161	0.333	0.482	0.630	(0.00,0.81)	0.057
Plac-venl-vort	0.155	0.259	0.597	0.551	(0.00,0.66)	0.057
Plac-fluo-nefa	0.153	0.415	0.369	0.712	(0.00,0.97)	0.095
bupr-esci-sert	0.152	1.158	0.132	0.895	(0.00,2.42)	0.044
dulo-esci-paro	0.152	0.270	0.562	0.574	(0.00,0.68)	0.041
Plac-bupr-paro	0.142	0.391	0.363	0.717	(0.00,0.91)	0.025
agom-dulo-paro	0.141	0.347	0.406	0.684	(0.00,0.82)	0.041
Plac-agom-esci	0.134	0.275	0.487	0.626	(0.00,0.67)	0.031
bupr-traz-venl	0.133	0.498	0.268	0.789	(0.00,1.11)	0.000
dulo-esci-venl	0.133	0.343	0.387	0.699	(0.00,0.81)	0.031
Plac-esci-fluo	0.130	0.231	0.564	0.573	(0.00,0.58)	0.051
Plac-mirt-sert	0.129	0.324	0.398	0.691	(0.00,0.76)	0.028
mirt-paro-sert	0.127	0.456	0.278	0.781	(0.00,1.02)	0.049
cita-esci-fluo	0.124	0.273	0.455	0.649	(0.00,0.66)	0.016
Plac-paro-sert	0.122	0.220	0.554	0.580	(0.00,0.55)	0.014
mirt-paro-traz	0.120	0.413	0.291	0.771	(0.00,0.93)	0.023
cita-mirt-venl	0.120	1.096	0.109	0.913	(0.00,2.27)	0.206
dulo-fluo-venl	0.119	0.334	0.358	0.721	(0.00,0.77)	0.000
Plac-esci-paro	0.119	0.159	0.748	0.455	(0.00,0.43)	0.011
agom-fluo-paro	0.118	0.221	0.533	0.594	(0.00,0.55)	0.040
cita-rebo-venl	0.107	0.485	0.221	0.825	(0.00,1.06)	0.000
Plac-fluo-paro	0.105	0.145	0.724	0.469	(0.00,0.39)	0.057
amit-fluv-mirt	0.102	0.707	0.144	0.885	(0.00,1.49)	0.276
fluo-fluv-paro	0.102	0.451	0.225	0.822	(0.00,0.99)	0.039
fluv-mirt-paro	0.099	0.566	0.175	0.861	(0.00,1.21)	0.121
Plac-cita-vila	0.099	0.230	0.429	0.668	(0.00,0.55)	0.023
Plac-agom-paro	0.097	0.163	0.595	0.552	(0.00,0.42)	0.031
Plac-fluo-mirt	0.097	0.267	0.362	0.717	(0.00,0.62)	0.099
clom-paro-venl	0.092	0.503	0.184	0.854	(0.00,1.08)	0.081
Plac-desv-dulo	0.092	0.291	0.317	0.752	(0.00,0.66)	0.031
esci-fluo-sert	0.090	0.266	0.338	0.735	(0.00,0.61)	0.000
agom-dulo-fluo	0.090	0.391	0.229	0.819	(0.00,0.86)	0.008
Plac-cita-fluo	0.090	0.297	0.302	0.763	(0.00,0.67)	0.077
Plac-dulo-fluo	0.088	0.386	0.229	0.819	(0.00,0.84)	0.087
Plac-mirt-paro	0.086	0.198	0.436	0.663	(0.00,0.47)	0.017
Plac-bupr-esci	0.084	0.252	0.332	0.740	(0.00,0.58)	0.035
amit-sert-venl	0.082	0.389	0.210	0.834	(0.00,0.84)	0.034
agom-esci-venl	0.080	0.353	0.226	0.821	(0.00,0.77)	0.000
clom-miln-paro	0.080	0.637	0.125	0.900	(0.00,1.33)	0.069
fluo-mirt-sert	0.080	0.331	0.240	0.810	(0.00,0.73)	0.016
cita-mirt-paro	0.079	0.646	0.123	0.903	(0.00,1.34)	0.074
clom-paro-traz	0.076	0.708	0.108	0.914	(0.00,1.46)	0.105
esci-fluo-paro	0.075	0.270	0.278	0.781	(0.00,0.60)	0.035
amit-mirt-paro	0.074	0.358	0.207	0.836	(0.00,0.78)	0.091
Plac-nefa-sert	0.073	0.393	0.185	0.853	(0.00,0.84)	0.024
Plac-agom-fluo	0.073	0.232	0.314	0.753	(0.00,0.53)	0.094
bupr-esci-paro	0.072	0.444	0.162	0.871	(0.00,0.94)	0.021
amit-fluv-paro	0.071	0.547	0.129	0.897	(0.00,1.14)	0.125
amit-fluo-miln	0.069	0.426	0.162	0.871	(0.00,0.90)	0.037
Plac-agom-dulo	0.069	0.345	0.200	0.842	(0.00,0.75)	0.059
Plac-cita-clom-traz	0.068	0.691	0.099	0.921	(0.00,1.42)	0.044
amit-fluo-paro	0.065	0.234	0.279	0.780	(0.00,0.52)	0.047
Plac-sert-venl	0.057	0.237	0.240	0.810	(0.00,0.52)	0.021

Plac-cita-sert	0.053	0.271	0.197	0.843	(0.00,0.58)	0.025
cita-clom-fluv	0.051	0.707	0.072	0.942	(0.00,1.44)	0.000
cita-paro-rebo	0.043	0.341	0.125	0.901	(0.00,0.71)	0.000
amit-miln-paro	0.039	0.474	0.082	0.935	(0.00,0.97)	0.091
bupr-esci-venl	0.039	0.486	0.079	0.937	(0.00,0.99)	0.059
amit-fluo-fluv	0.038	0.362	0.104	0.917	(0.00,0.75)	0.001
fluo-paro-rebo	0.034	0.248	0.135	0.892	(0.00,0.52)	0.030
esci-paro-sert	0.031	0.317	0.098	0.922	(0.00,0.65)	0.012
Plac-bupr-sert	0.030	1.078	0.028	0.978	(0.00,2.14)	0.046
Plac-esci-sert	0.029	0.232	0.126	0.900	(0.00,0.48)	0.020
bupr-paro-sert	0.026	1.086	0.024	0.981	(0.00,2.16)	0.000
Plac-fluo-venl	0.024	0.152	0.156	0.876	(0.00,0.32)	0.060
Plac-paro-rebo	0.020	0.200	0.099	0.921	(0.00,0.41)	0.034
Plac-fluo-traz	0.009	0.418	0.022	0.983	(0.00,0.83)	0.118

7.4.1.2 Side-spliting

Side	Direct Coef.	Std. Err.	Indirect Coef.	Std. Err.	Difference Coef.	Std. Err.	P>|z|
Plac agom	.4415803	.0859582	.552755	.0988431	-.1111747	.1311493	0.397
Plac amit	.9573629	.0946638	.5878816	.0787163	.3694813	.1234582	0.003
Plac bupr	.3654864	.0927772	.630745	.1532968	-.2652586	.1790409	0.138
Plac cita	.4110246	.095197	.4202149	.0913897	-.0091903	.1324423	0.945
Plac dulo	.6497865	.0689822	.5257675	.0962146	.124019	.1194443	0.299
Plac esci	.3632665	.0702393	.706371	.0821739	-.3431045	.1086326	0.002
Plac fluo	.3491209	.0597523	.4697402	.0571303	-.1206194	.082804	0.145
Plac fluv	.7099613	.1459749	.3942117	.1116645	.3157496	.183787	0.086
Plac mirt	.5714965	.1255684	.6470928	.0916331	-.0755964	.1567508	0.630
Plac nefa	.5510253	.1502111	.4301954	.1971884	.12083	.2478859	0.626
Plac paro	.4974848	.054954	.5967459	.0590759	-.0992611	.0806961	0.219
Plac rebo	.3352179	.1079945	.2476209	.1370544	.087597	.1744892	0.616
Plac sert	.4680976	.083469	.5270112	.0785816	-.0589136	.1147002	0.608
Plac traz	.5641814	.1486057	.263633	.1282519	.3005485	.1967293	0.127
Plac venl	.5815499	.0724536	.5545188	.0671739	.0270311	.0989629	0.785
Plac vort	.543535	.0765498	.2959051	.1789759	.2476298	.1967578	0.208
agom dulo	.2747858	.2866702	.1039066	.0854803	.1708792	.2991433	0.568
agom esci	-.2080054	.2595283	.0432264	.0844806	-.2512319	.2729249	0.357
agom fluo	-.0056701	.1392716	-.1025954	.0825508	.0969253	.1621819	0.550
agom paro	.1585275	.1334393	.0141641	.0826539	.1443634	.1569268	0.358
agom venl	-.3052271	.2372624	.1220368	.0808622	-.4272639	.2506652	0.088
amit fluo	-.0630969	.1615738	-.3806954	.0728447	.3175985	.1772401	0.073
amit fluv	-.0850522	.2616439	-.2533867	.1090556	.1683345	.2834592	0.553
amit miln	-.1562593	.3178515	-.207626	.1404851	.0513667	.3474751	0.882
amit mirt	-.0466865	.230338	-.1310849	.0959596	.0843985	.2495215	0.735
amit paro	-.0673421	.123842	-.2437107	.0758295	.1763686	.1451395	0.224
amit sert	-.251999	.133142	-.2344901	.0891939	-.0175088	.1602584	0.913
amit traz	-.2445385	.2509759	-.3707502	.1197354	.1262117	.2780513	0.650
amit venl	-.1688681	.2956097	-.1724617	.0759378	.0035936	.3051982	0.991
bupr esci	.0692227	.2203113	.0722108	.1023087	-.0029882	.2429165	0.990
bupr fluo	.1749594	.1888126	-.0766353	.0965806	.2515947	.2120367	0.235
bupr paro	-.0008168	.3902452	.1124624	.0892135	-.1132791	.4003128	0.777
bupr sert	.0645308	1.034954	.0625573	.0968483	.0019735	1.039476	0.998
bupr traz	-.7396667	.4164884	.0203062	.1272056	-.759973	.4354812	0.081
bupr venl	-.1588537	.2893555	.1613214	.0949315	-.3201751	.3045301	0.293
cita clom	.5667569	.4256849	-.0757	.1267543	.6424569	.4441557	0.148
cita esci	.2959492	.1130309	-.0570246	.0970747	.3529738	.1489159	0.018
cita fluo	.0373023	.2150543	-.0089074	.0777329	.0462097	.2286697	0.840
cita fluv	-.101741	.3558693	.1142703	.1115056	-.2160113	.3729295	0.562
cita mirt	-.2805179	.4062032	.2327475	.0975157	-.5132655	.4177443	0.219
cita paro	-.3038272	.309122	.1542744	.0757133	-.4581016	.3188031	0.151
cita rebo	-.5478924	.2878079	-.0521753	.1093527	-.4957171	.3078821	0.107
cita sert	.1134413	.2503919	.0798717	.0874143	.0335696	.2652213	0.899
cita venl	-.5482632	.3429272	.1899551	.0807923	-.7382183	.3524547	0.036
cita vila	.1041882	.2014791	.0255024	.1530279	.0786858	.2530667	0.756
clom fluo	.4934394	.2508941	-.0903415	.1209183	.5837809	.2785093	0.036
clom fluv	-.6174263	.5351887	.1673986	.1381831	-.784825	.55274	0.156
clom miln	.1397623	.4403739	.1486538	.1627557	-.0088915	.4694876	0.985
clom paro	.0973774	.1597419	.1944209	.1428646	-.0970435	.2142756	0.651
clom sert	.0462692	.2857686	.1187204	.1260841	-.0724512	.3123477	0.817
clom traz	-.4890301	.4360124	.0550663	.1471523	-.5440964	.4601168	0.237
clom venl	.49003	.3213696	.1290924	.1215146	.3609376	.343935	0.294
desv dulo	.2079944	.2756128	.2145641	.1128804	-.0065697	.2988051	0.982

dulo	esci	.1675942	.1671796	-.1601184	.0802877	.3277126	.1854881	0.077
dulo	fluo	-.179041	.316276	-.1959446	.0681761	.0169036	.3241009	0.958
dulo	paro	.0178401	.1182892	-.0969464	.0752425	.1147865	.1407065	0.415
dulo	venl	.1064391	.1996747	-.0608894	.0746071	.1673286	.2131564	0.432
dulo	vort	-.3047269	.1163128	.0755919	.1098213	-.3803188	.1606357	0.018
esci	fluo	-.1131038	.190763	-.0943116	.0674105	-.0187922	.2023511	0.926
esci	paro	.0054384	.1680778	.0399973	.0677852	-.0345589	.1813445	0.849
esci	sert	.1145127	.2239114	-.0247132	.0791709	.1392258	.2373798	0.558
esci	venl	-.1908574	.2416769	.0803873	.0719146	-.2712447	.2521516	0.282
fluo	fluv	.0307123	.2798662	.1070203	.0989596	-.0763079	.2968446	0.797
fluo	miln	-.1627381	.235568	.2348533	.1432085	-.3975914	.275769	0.149
fluo	mirt	.2751742	.1647164	.1893777	.0879108	.0857964	.1867878	0.646
fluo	nefa	.0369471	.3374653	.1034088	.133157	-.0664617	.3627892	0.855
fluo	paro	.0548377	.1036025	.1541233	.0564049	-.0992856	.1179038	0.400
fluo	rebo	-.1965862	.1790166	-.0819737	.10334	-.1146125	.206584	0.579
fluo	sert	.3546302	.1450259	.0232907	.0709925	.3313395	.1614537	0.040
fluo	traz	.1798791	.2929184	-.0475743	.1081022	.2274535	.312223	0.466
fluo	venl	.2199441	.0971524	.1225745	.0682736	.0973696	.1187474	0.412
fluv	miln	.5392491	.3087935	-.1019523	.1562422	.6412014	.3460707	0.064
fluv	mirt	.1315915	.2478076	.1049325	.1185017	.026659	.2746788	0.923
fluv	paro	.1277775	.3356916	.0249831	.097057	.1027945	.3494645	0.769
fluv	sert	-.4433568	.3346439	.0315004	.1055039	-.4748572	.3508828	0.176
fluv	venl	.8605743	.4627316	.0182338	.1003474	.8423405	.4734871	0.075
miln	paro	.0492335	.1892725	-.0275477	.1566732	.0767812	.2457054	0.755
miln	sert	-.7356996	.9342899	-.0267323	.1317377	-.7089674	.9435319	0.452
mirt	paro	.0318853	.1651668	-.1074174	.0874072	.1393026	.1868296	0.456
mirt	sert	.0262103	.3002919	-.1351149	.0918016	.1613252	.3140108	0.607
mirt	traz	-.4064169	.2740671	-.1910906	.1261943	-.2153263	.3016927	0.475
mirt	venl	-.3119721	.2216931	-.0121174	.0889142	-.2998547	.2388589	0.209
nefa	paro	.2805387	.3107846	-.0086226	.1345662	.2891613	.3385718	0.393
nefa	sert	-.1541509	.3719155	.0127929	.1376809	-.1669437	.3965819	0.674
paro	rebo	-.2133283	.1574656	-.2553783	.1073656	.04205	.1906199	0.825
paro	sert	.1066496	.2307413	-.0574073	.0677966	.1640569	.2405039	0.495
paro	traz	-.5413155	.295477	-.0999722	.1074826	-.4413434	.3144588	0.160
paro	venl	.4321144	.1987847	-.015208	.0609504	.4473223	.2080984	0.032
rebo	venl	-.0436753	.3674653	.2874033	.0979636	-.3310786	.3802993	0.384
sert	traz	.5806296	.4384827	-.1524673	.1121787	.7330969	.4526049	0.105
sert	venl	.1659343	.223033	.056731	.0743749	.1092033	.2351095	0.642
traz	venl	.4688603	.2919782	.1306436	.1129111	.3382167	.3135498	0.281
venl	vort	.2070379	.2081842	-.1126351	.0884713	.3196731	.2265198	0.158

7.4.1.3 Design-by-treatment test

chi2(141) = 151.12

Prob > chi2 = 0.0631

7.4.2 Dropouts for any reason

7.4.2.1 Loop-specific approach

Loops With Evidence of Statistical Inconsistency:

Loop	IF	seIF	z_value	p_value	CI_95	Loop_Heterog_tau2
cita-clom-venl	2.706	1.055	2.566	0.010	(0.64,4.77)	0.000
cita-fluv-venl	2.514	0.995	2.526	0.012	(0.56,4.46)	0.000
cita-paro-venl	2.271	0.897	2.532	0.011	(0.51,4.03)	0.000
Plac-cita-venl	2.202	0.890	2.474	0.013	(0.46,3.95)	0.038
cita-esci-venl	2.163	0.869	2.490	0.013	(0.46,3.87)	0.000
cita-fluo-venl	2.162	0.858	2.520	0.012	(0.48,3.84)	0.000
cita-clom-sert	1.534	0.652	2.354	0.019	(0.26,2.81)	0.000
agom-dulo-venl	1.312	0.392	3.349	0.001	(0.54,2.08)	0.000
fluv-mirt-venl	1.180	0.539	2.190	0.029	(0.12,2.24)	0.000
Plac-cita-mirt	0.989	0.467	2.119	0.034	(0.07,1.90)	0.000

agom-paro-venl	0.906	0.314	2.887	0.004	(0.29,1.52)	0.000
dulo-venl-vort	0.824	0.352	2.344	0.019	(0.13,1.51)	0.033
dulo-fluo-venl	0.796	0.363	2.196	0.028	(0.09,1.51)	0.000
cita-fluo-sert	0.757	0.368	2.054	0.040	(0.03,1.48)	0.008
Plac-amit-fluo	0.581	0.251	2.317	0.021	(0.09,1.07)	0.126
Plac-amit-sert	0.534	0.210	2.538	0.011	(0.12,0.95)	0.043

Loops Without Evidence of Statistical Inconsistency:

Loop	IF	seIF	z_value	p_value	CI_95	Loop_Heterog_tau2
Plac-clom-sert	1.440	0.906	1.589	0.112	(0.00,3.21)	0.020
cita-sert-venl	1.365	1.167	1.170	0.242	(0.00,3.65)	0.283
Plac-clom-paro	1.182	0.856	1.382	0.167	(0.00,2.86)	0.032
fluo-sert-traz	1.173	0.839	1.398	0.162	(0.00,2.82)	0.085
amit-fluv-sert	1.060	0.588	1.802	0.072	(0.00,2.21)	0.032
Plac-clom-fluo	1.051	0.986	1.065	0.287	(0.00,2.98)	0.128
cita-clom-paro	1.016	0.593	1.713	0.087	(0.00,2.18)	0.000
amit-bupr-fluo	0.998	0.566	1.763	0.078	(0.00,2.11)	0.000
cita-mirt-venl	0.981	0.960	1.022	0.307	(0.00,2.86)	0.000
amit-fluv-venl	0.981	0.676	1.450	0.147	(0.00,2.31)	0.042
cita-mirt-paro	0.931	0.554	1.679	0.093	(0.00,2.02)	0.000
cita-fluv-sert	0.924	1.314	0.703	0.482	(0.00,3.50)	0.484
clom-fluo-fluv	0.902	0.909	0.992	0.321	(0.00,2.68)	0.000
mirt-sert-traz	0.900	0.710	1.268	0.205	(0.00,2.29)	0.000
agom-dulo-esci	0.887	0.542	1.637	0.102	(0.00,1.95)	0.064
amit-bupr-paro	0.885	0.735	1.205	0.228	(0.00,2.32)	0.040
Plac-clom-venl	0.836	1.018	0.821	0.411	(0.00,2.83)	0.060
Plac-fluv-sert	0.825	0.515	1.600	0.110	(0.00,1.83)	0.027
cita-fluo-mirt	0.787	0.672	1.171	0.242	(0.00,2.10)	0.109
paro-sert-traz	0.779	0.890	0.876	0.381	(0.00,2.52)	0.071
clom-fluv-paro	0.776	0.881	0.881	0.379	(0.00,2.50)	0.000
cita-clom-fluo	0.774	0.608	1.273	0.203	(0.00,1.97)	0.000
amit-bupr-venl	0.735	0.637	1.153	0.249	(0.00,1.98)	0.000
fluo-nefa-sert	0.731	0.648	1.127	0.260	(0.00,2.00)	0.029
fluv-paro-sert	0.697	0.678	1.028	0.304	(0.00,2.03)	0.033
Plac-sert-traz	0.696	0.629	1.106	0.269	(0.00,1.93)	0.010
fluv-mirt-sert	0.696	0.851	0.818	0.413	(0.00,2.36)	0.095
fluo-fluv-venl	0.683	0.541	1.263	0.207	(0.00,1.74)	0.000
miln-paro-sert	0.675	0.614	1.100	0.271	(0.00,1.88)	0.000
cita-esci-sert	0.663	0.355	1.867	0.062	(0.00,1.36)	0.000
bupr-paro-traz	0.656	0.610	1.076	0.282	(0.00,1.85)	0.000
amit-bupr-esci-sert	0.644	0.607	1.061	0.289	(0.00,1.83)	0.000
Plac-amit-fluv	0.644	0.408	1.577	0.115	(0.00,1.44)	0.121
amit-sert-traz	0.634	0.669	0.948	0.343	(0.00,1.95)	0.000
Plac-fluv-venl	0.617	0.542	1.138	0.255	(0.00,1.68)	0.050
fluv-mirt-paro	0.614	0.431	1.423	0.155	(0.00,1.46)	0.000
dulo-esci-fluo	0.595	0.508	1.171	0.241	(0.00,1.59)	0.098
Plac-fluo-nefa	0.594	0.537	1.106	0.269	(0.00,1.65)	0.116
clom-fluv-sert	0.592	1.393	0.425	0.671	(0.00,3.32)	0.288
clom-paro-venl	0.580	0.465	1.247	0.212	(0.00,1.49)	0.000
mirt-paro-sert	0.573	0.376	1.525	0.127	(0.00,1.31)	0.000
Plac-agom-venl	0.569	0.323	1.764	0.078	(0.00,1.20)	0.053
agom-fluo-venl	0.563	0.331	1.700	0.089	(0.00,1.21)	0.033
amit-fluv-paro	0.549	0.469	1.170	0.242	(0.00,1.47)	0.038
fluo-fluv-sert	0.534	0.675	0.792	0.428	(0.00,1.86)	0.057
amit-fluv-miln	0.518	0.564	0.919	0.358	(0.00,1.62)	0.009
Plac-amit-traz	0.508	0.389	1.308	0.191	(0.00,1.27)	0.090
bupr-fluo-traz	0.505	0.572	0.882	0.378	(0.00,1.63)	0.027
amit-bupr-traz	0.504	0.688	0.733	0.464	(0.00,1.85)	0.000
amit-fluo-sert	0.495	0.257	1.928	0.054	(0.00,1.00)	0.000
bupr-esci-sert-traz	0.494	0.763	0.648	0.517	(0.00,1.99)	0.000
Plac-venl-vort	0.488	0.263	1.859	0.063	(0.00,1.00)	0.033
fluo-mirt-venl	0.485	0.307	1.578	0.114	(0.00,1.09)	0.032
amit-mirt-venl	0.482	0.418	1.153	0.249	(0.00,1.30)	0.000
fluo-nefa-paro	0.475	0.543	0.873	0.383	(0.00,1.54)	0.000
amit-miln-sert	0.468	0.675	0.693	0.488	(0.00,1.79)	0.000
fluo-mirt-paro	0.460	0.243	1.896	0.058	(0.00,0.94)	0.005
Plac-cita-sert	0.454	0.294	1.543	0.123	(0.00,1.03)	0.013
Plac-cita-rebo	0.453	0.473	0.958	0.338	(0.00,1.38)	0.080
Plac-dulo-fluo	0.431	0.389	1.108	0.268	(0.00,1.19)	0.081
Plac-mirt-sert	0.416	0.313	1.329	0.184	(0.00,1.03)	0.009
clom-sert-venl	0.410	0.870	0.472	0.637	(0.00,2.12)	0.221
Plac-dulo-esci	0.409	0.221	1.852	0.064	(0.00,0.84)	0.039
mirt-traz-venl	0.399	0.471	0.847	0.397	(0.00,1.32)	0.000

mirt-paro-traz	0.390	0.497	0.786	0.432	(0.00,1.36)	0.035
amit-fluo-paro	0.385	0.223	1.727	0.084	(0.00,0.82)	0.000
Plac-fluo-sert	0.384	0.242	1.587	0.113	(0.00,0.86)	0.088
Plac-dulo-venl	0.382	0.245	1.558	0.119	(0.00,0.86)	0.029
bupr-traz-venl	0.382	0.545	0.701	0.483	(0.00,1.45)	0.000
agom-esci-venl	0.370	0.430	0.861	0.389	(0.00,1.21)	0.000
agom-esci-paro	0.366	0.365	1.005	0.315	(0.00,1.08)	0.000
agom-esci-fluo	0.363	0.501	0.725	0.469	(0.00,1.34)	0.104
mirt-paro-venl	0.359	0.298	1.204	0.229	(0.00,0.94)	0.000
Plac-fluv-mirt	0.355	0.291	1.218	0.223	(0.00,0.93)	0.000
Plac-agom-dulo	0.354	0.318	1.115	0.265	(0.00,0.98)	0.020
cita-fluv-mirt	0.353	0.600	0.589	0.556	(0.00,1.53)	0.000
amit-fluo-miln	0.344	0.451	0.763	0.445	(0.00,1.23)	0.000
paro-traz-venl	0.343	0.468	0.733	0.464	(0.00,1.26)	0.000
Plac-cita-clom	0.342	0.964	0.355	0.723	(0.00,2.23)	0.000
fluo-paro-sert	0.341	0.275	1.239	0.215	(0.00,0.88)	0.000
cita-fluo-fluv	0.331	0.479	0.691	0.489	(0.00,1.27)	0.000
Plac-bupr-paro	0.325	0.453	0.716	0.474	(0.00,1.21)	0.029
dulo-esci-paro	0.324	0.287	1.130	0.258	(0.00,0.89)	0.015
esci-paro-sert	0.322	0.425	0.758	0.448	(0.00,1.16)	0.034
amit-mirt-paro	0.319	0.316	1.009	0.313	(0.00,0.94)	0.017
amit-paro-traz	0.314	0.451	0.695	0.487	(0.00,1.20)	0.031
fluo-miln-sert	0.311	0.620	0.501	0.616	(0.00,1.53)	0.006
clom-paro-sert	0.297	0.418	0.710	0.478	(0.00,1.12)	0.000
Plac-amit-bupr	0.295	0.612	0.482	0.630	(0.00,1.49)	0.078
Plac-esci-fluo	0.291	0.276	1.054	0.292	(0.00,0.83)	0.080
dulo-fluo-paro	0.290	0.331	0.874	0.382	(0.00,0.94)	0.000
fluo-paro-venl	0.285	0.211	1.349	0.177	(0.00,0.70)	0.000
amit-fluo-venl	0.285	0.359	0.794	0.427	(0.00,0.99)	0.000
Plac-amit-paro	0.282	0.192	1.475	0.140	(0.00,0.66)	0.065
Plac-cita-fluv	0.282	0.356	0.792	0.428	(0.00,0.98)	0.000
Plac-amit-venl	0.280	0.416	0.675	0.500	(0.00,1.09)	0.095
amit-fluo-mirt	0.279	0.343	0.813	0.416	(0.00,0.95)	0.026
amit-fluv-mirt	0.279	0.402	0.694	0.488	(0.00,1.07)	0.001
cita-fluo-rebo	0.277	0.365	0.759	0.448	(0.00,0.99)	0.000
Plac-bupr-traz	0.261	0.397	0.657	0.511	(0.00,1.04)	0.000
Plac-clom-fluv	0.255	1.177	0.217	0.828	(0.00,2.56)	0.008
Plac-sert-venl	0.244	0.301	0.811	0.417	(0.00,0.84)	0.051
bupr-fluo-paro	0.239	0.441	0.542	0.588	(0.00,1.10)	0.000
Plac-paro-venl	0.236	0.240	0.984	0.325	(0.00,0.71)	0.043
amit-mirt-traz	0.235	0.438	0.538	0.591	(0.00,1.09)	0.000
Plac-nefa-sert	0.234	0.426	0.548	0.584	(0.00,1.07)	0.019
cita-paro-sert	0.231	0.459	0.504	0.614	(0.00,1.13)	0.007
clom-fluo-venl	0.230	0.510	0.450	0.653	(0.00,1.23)	0.000
Plac-mirt-venl	0.227	0.273	0.833	0.405	(0.00,0.76)	0.033
amit-paro-sert	0.222	0.285	0.780	0.435	(0.00,0.78)	0.008
dulo-paro-venl	0.221	0.270	0.819	0.413	(0.00,0.75)	0.000
cita-clom-fluv	0.203	1.007	0.202	0.840	(0.00,2.18)	0.000
Plac-mirt-traz	0.201	0.318	0.631	0.528	(0.00,0.82)	0.000
Plac-nefa-paro	0.201	0.371	0.541	0.589	(0.00,0.93)	0.035
Plac-fluo-mirt	0.193	0.284	0.679	0.497	(0.00,0.75)	0.111
fluo-fluv-paro	0.191	0.443	0.431	0.666	(0.00,1.06)	0.000
bupr-esci-fluo	0.188	0.317	0.592	0.554	(0.00,0.81)	0.007
amit-paro-venl	0.185	0.368	0.504	0.614	(0.00,0.91)	0.000
agom-dulo-paro	0.185	0.305	0.606	0.545	(0.00,0.78)	0.000
Plac-bupr-fluo	0.177	0.278	0.637	0.524	(0.00,0.72)	0.086
cita-fluo-paro	0.176	0.361	0.489	0.625	(0.00,0.88)	0.000
Plac-fluv-paro	0.173	0.380	0.454	0.650	(0.00,0.92)	0.033
fluo-fluv-miln	0.171	0.491	0.349	0.727	(0.00,1.13)	0.000
Plac-cita-paro	0.170	0.363	0.467	0.640	(0.00,0.88)	0.031
esci-paro-venl	0.170	0.342	0.497	0.620	(0.00,0.84)	0.000
Plac-bupr-venl	0.161	0.318	0.506	0.613	(0.00,0.78)	0.036
Plac-fluo-rebo	0.158	0.313	0.504	0.614	(0.00,0.77)	0.125
Plac-desv-dulo	0.152	0.288	0.529	0.597	(0.00,0.72)	0.022
amit-traz-venl	0.152	0.528	0.287	0.774	(0.00,1.19)	0.000
fluo-mirt-traz	0.147	0.631	0.233	0.816	(0.00,1.38)	0.200
fluo-traz-venl	0.147	0.512	0.286	0.775	(0.00,1.15)	0.027
Plac-fluo-traz	0.146	0.421	0.347	0.729	(0.00,0.97)	0.116
cita-esci-fluo	0.140	0.300	0.467	0.640	(0.00,0.73)	0.004
Plac-esci-paro	0.139	0.255	0.546	0.585	(0.00,0.64)	0.045
cita-mirt-sert	0.137	0.572	0.239	0.811	(0.00,1.26)	0.000
cita-paro-rebo	0.136	0.642	0.212	0.832	(0.00,1.39)	0.089
esci-fluo-venl	0.135	0.334	0.402	0.687	(0.00,0.79)	0.023
fluo-mirt-sert	0.134	0.510	0.263	0.793	(0.00,1.13)	0.098
Plac-esci-sert	0.115	0.294	0.391	0.696	(0.00,0.69)	0.046
amit-sert-venl	0.113	0.436	0.259	0.795	(0.00,0.97)	0.027
Plac-fluo-paro	0.111	0.157	0.709	0.478	(0.00,0.42)	0.068

Plac-paro-traz	0.107	0.362	0.296	0.767	(0.00,0.82)	0.034
Plac-paro-rebo	0.107	0.245	0.437	0.662	(0.00,0.59)	0.062
Plac-agom-esci	0.107	0.370	0.289	0.773	(0.00,0.83)	0.051
Plac-agom-paro	0.104	0.189	0.551	0.582	(0.00,0.48)	0.037
fluo-sert-venl	0.103	0.337	0.307	0.758	(0.00,0.76)	0.033
nefa-paro-sert	0.103	0.517	0.199	0.842	(0.00,1.12)	0.000
Plac-esci-venl	0.100	0.317	0.314	0.753	(0.00,0.72)	0.057
Plac-dulo-paro	0.100	0.155	0.643	0.520	(0.00,0.40)	0.024
Plac-dulo-vort	0.099	0.161	0.617	0.537	(0.00,0.41)	0.010
Plac-mirt-paro	0.096	0.224	0.429	0.668	(0.00,0.54)	0.027
Plac-fluo-fluv	0.094	0.442	0.212	0.832	(0.00,0.96)	0.112
Plac-bupr-esci	0.090	0.271	0.331	0.741	(0.00,0.62)	0.034
agom-fluo-paro	0.087	0.192	0.454	0.650	(0.00,0.46)	0.000
dulo-esci-venl	0.079	0.400	0.199	0.842	(0.00,0.86)	0.037
Plac-cita-esci	0.079	0.203	0.389	0.698	(0.00,0.48)	0.028
bupr-esci-paro	0.078	0.495	0.157	0.875	(0.00,1.05)	0.003
fluo-paro-rebo	0.077	0.215	0.360	0.719	(0.00,0.50)	0.000
fluo-paro-traz	0.073	0.403	0.181	0.856	(0.00,0.86)	0.000
bupr-paro-venl	0.068	0.490	0.139	0.889	(0.00,1.03)	0.000
clom-fluo-paro	0.065	0.320	0.204	0.838	(0.00,0.69)	0.000
cita-esci-paro	0.062	0.373	0.167	0.867	(0.00,0.79)	0.000
fluv-miln-sert	0.056	1.119	0.050	0.960	(0.00,2.25)	0.183
agom-dulo-fluo	0.055	0.565	0.097	0.923	(0.00,1.16)	0.082
esci-sert-venl	0.054	0.582	0.092	0.926	(0.00,1.19)	0.125
Plac-cita-vila	0.053	0.230	0.232	0.817	(0.00,0.50)	0.000
fluo-fluv-mirt	0.051	0.544	0.094	0.925	(0.00,1.12)	0.095
Plac-paro-sert	0.047	0.270	0.174	0.862	(0.00,0.58)	0.033
cita-fluv-paro	0.036	0.540	0.067	0.946	(0.00,1.10)	0.000
Plac-cita-fluo	0.035	0.343	0.103	0.918	(0.00,0.71)	0.094
fluv-miln-paro	0.034	0.489	0.071	0.944	(0.00,0.99)	0.000
Plac-agom-fluo	0.032	0.263	0.123	0.902	(0.00,0.55)	0.106
amit-mirt-sert	0.031	0.365	0.084	0.933	(0.00,0.75)	0.000
Plac-fluo-venl	0.028	0.185	0.152	0.879	(0.00,0.39)	0.089
esci-fluo-paro	0.026	0.277	0.094	0.925	(0.00,0.57)	0.001
bupr-esci-venl	0.026	0.365	0.071	0.944	(0.00,0.74)	0.000
Plac-traz-venl	0.023	0.452	0.051	0.959	(0.00,0.91)	0.047
bupr-fluo-venl	0.022	0.281	0.078	0.938	(0.00,0.57)	0.000
clom-fluo-sert	0.018	0.465	0.039	0.969	(0.00,0.93)	0.000
esci-fluo-sert	0.017	0.411	0.042	0.967	(0.00,0.82)	0.070
Plac-amit-mirt	0.015	0.336	0.046	0.964	(0.00,0.67)	0.100
fluo-miln-paro	0.015	0.266	0.055	0.956	(0.00,0.54)	0.000
amit-miln-paro	0.010	0.445	0.022	0.983	(0.00,0.88)	0.019
amit-fluo-fluv	0.008	0.410	0.021	0.984	(0.00,0.81)	0.000
amit-fluo-traz	0.003	0.428	0.007	0.995	(0.00,0.84)	0.000

7.4.2.2 Side-splitting

<table>
<tr><th>Side</th><th>Direct</th><th></th><th>Indirect</th><th></th><th>Difference</th><th></th><th></th></tr>
<tr><th></th><th>Coef.</th><th>Std. Err.</th><th>Coef.</th><th>Std. Err.</th><th>Coef.</th><th>Std. Err.</th><th>P>|z|</th></tr>
<tr><td>Plac agom</td><td>-.1417504</td><td>.0990303</td><td>-.2047001</td><td>.1094086</td><td>.0629497</td><td>.1475627</td><td>0.670</td></tr>
<tr><td>Plac amit</td><td>-.259652</td><td>.0996284</td><td>.1001535</td><td>.0849819</td><td>-.3598056</td><td>.1309883</td><td>0.006</td></tr>
<tr><td>Plac bupr</td><td>.0092913</td><td>.0993403</td><td>-.1479865</td><td>.1537447</td><td>.1572779</td><td>.1832422</td><td>0.391</td></tr>
<tr><td>Plac cita</td><td>.0400299</td><td>.1128471</td><td>-.15235</td><td>.1105812</td><td>.1923799</td><td>.1585456</td><td>0.225</td></tr>
<tr><td>Plac clom</td><td>-.7790896</td><td>.8380909</td><td>.2953841</td><td>.1301104</td><td>-1.074474</td><td>.8481303</td><td>0.205</td></tr>
<tr><td>Plac dulo</td><td>.0681872</td><td>.0754057</td><td>.1107586</td><td>.105277</td><td>-.0425714</td><td>.1307833</td><td>0.745</td></tr>
<tr><td>Plac esci</td><td>-.0095213</td><td>.0779628</td><td>-.2347466</td><td>.0968923</td><td>.2252254</td><td>.1250739</td><td>0.072</td></tr>
<tr><td>Plac fluo</td><td>-.1553179</td><td>.0648497</td><td>-.0947885</td><td>.0626326</td><td>-.0605294</td><td>.0903839</td><td>0.503</td></tr>
<tr><td>Plac fluv</td><td>.0949152</td><td>.1433244</td><td>.102093</td><td>.1249923</td><td>-.0071777</td><td>.1901941</td><td>0.970</td></tr>
<tr><td>Plac mirt</td><td>-.0648253</td><td>.1242582</td><td>.0302744</td><td>.1000767</td><td>-.0950997</td><td>.160317</td><td>0.553</td></tr>
<tr><td>Plac nefa</td><td>-.1690423</td><td>.15114</td><td>.1023489</td><td>.2282917</td><td>-.2713912</td><td>.2737707</td><td>0.322</td></tr>
<tr><td>Plac paro</td><td>-.0266118</td><td>.0560642</td><td>-.0721487</td><td>.0641619</td><td>.0455369</td><td>.0852774</td><td>0.593</td></tr>
<tr><td>Plac rebo</td><td>.0844146</td><td>.1152656</td><td>.2898218</td><td>.1559599</td><td>-.2054072</td><td>.1945918</td><td>0.291</td></tr>
<tr><td>Plac sert</td><td>.063378</td><td>.0846077</td><td>-.1581981</td><td>.0900008</td><td>.2215762</td><td>.123491</td><td>0.073</td></tr>
<tr><td>Plac traz</td><td>.1844915</td><td>.1545033</td><td>.0697202</td><td>.1493147</td><td>.1147713</td><td>.2150916</td><td>0.594</td></tr>
<tr><td>Plac venl</td><td>-.0052645</td><td>.0769717</td><td>.0706177</td><td>.0754349</td><td>-.0758822</td><td>.1079615</td><td>0.482</td></tr>
<tr><td>Plac vort</td><td>.0741987</td><td>.0888104</td><td>-.2918756</td><td>.1956225</td><td>.3660744</td><td>.217939</td><td>0.093</td></tr>
<tr><td>agom dulo</td><td>-.1481214</td><td>.3168311</td><td>.2889453</td><td>.0952119</td><td>-.4370667</td><td>.3308282</td><td>0.186</td></tr>
<tr><td>agom esci</td><td>.2448182</td><td>.3183052</td><td>.0559886</td><td>.0954026</td><td>.1888295</td><td>.3322877</td><td>0.570</td></tr>
<tr><td>agom fluo</td><td>.0158028</td><td>.1586163</td><td>.0565966</td><td>.0928699</td><td>-.0407938</td><td>.1845338</td><td>0.825</td></tr>
<tr><td>agom paro</td><td>.0184064</td><td>.1460356</td><td>.1663836</td><td>.0929442</td><td>-.1479773</td><td>.1730588</td><td>0.393</td></tr>
<tr><td>agom venl</td><td>.7159061</td><td>.2661728</td><td>.1436906</td><td>.0906823</td><td>.5722155</td><td>.2811986</td><td>0.042</td></tr>
</table>

amit bupr	.5764223	.5488314	-.0058267	.1048726	.582249	.5587613	0.297
amit fluo	-.4284238	.178743	-.0037237	.0784072	-.4247001	.1951835	0.030
amit fluv	-.2747875	.2654016	.2332383	.1167896	-.5080258	.2899632	0.080
amit miln	-.0309054	.3935253	.0144379	.1538861	-.0453433	.4225395	0.915
amit mirt	.1472953	.2394118	.0255482	.1029574	.1217471	.2606315	0.640
amit paro	-.0155594	.1329109	.0132981	.0811668	-.0288574	.1556934	0.853
amit sert	-.074271	.1422772	.0507895	.0971334	-.1250605	.1723138	0.468
amit traz	-.0157647	.2885761	.2164643	.1308097	-.232229	.3166449	0.463
amit venl	-.0029246	.3316963	.0906345	.0821985	-.0935591	.3417317	0.784
bupr esci	.0683956	.2317756	-.0903197	.1095689	.1587154	.2562695	0.536
bupr fluo	-.0003371	.1904218	-.1121022	.1028219	.1117651	.2164123	0.606
bupr paro	.2883557	.4445731	-.0222188	.0931103	.3105745	.4542188	0.494
bupr traz	-.0800427	.4096088	.1900265	.1385847	-.2700692	.4324177	0.532
bupr venl	.1485547	.2840458	.0606486	.1013179	.0879061	.3015748	0.771
cita clom	-.4906225	.5329733	.3949748	.1519771	-.8855973	.554218	0.11
cita esci	-.1323977	.1465726	.0142948	.1130091	-.1466925	.1847094	0.427
cita fluo	-.1456367	.2363589	-.0535927	.0924288	-.092044	.2537923	0.717
cita fluv	.348574	.3711307	.1356334	.1245917	.2129406	.3914857	0.586
cita mirt	.8601203	.4797164	.008839	.1095908	.8512813	.4920751	0.084
cita paro	.0530123	.3429991	.0089729	.0891721	.0440393	.3552035	0.901
cita rebo	.4728524	.3238006	.1773268	.1242521	.2955256	.3468219	0.394
cita sert	.5067421	.2902116	-.0418243	.1010627	.5485664	.3072354	0.074
cita venl	2.154652	.8499842	.0667774	.0932234	2.087875	.855081	0.015
cita vila	.1953294	.2557092	.1892247	.1695264	.0061048	.3070547	0.984
clom fluo	-.4212824	.312232	-.3879587	.1443728	-.0333237	.3439786	0.923
clom fluv	.6359866	.8319994	-.200197	.1582428	.8361836	.8469142	0.323
clom paro	-.4282242	.1687926	-.1728656	.191041	-.2553585	.2549252	0.316
clom sert	-.601302	.3647615	-.2625946	.1484312	-.3387074	.3938777	0.390
clom venl	-.0606247	.4531709	-.2536835	.1419271	.1930589	.4748759	0.684
desv dulo	-.157439	.2792424	.0466251	.1300133	-.204064	.3097438	0.510
dulo esci	-.4784728	.1775344	-.1058253	.0894518	-.3726475	.1987863	0.061
dulo fluo	.2440621	.3230467	-.2315906	.0743652	.4756527	.3321355	0.152
dulo paro	.0111432	.1305052	-.1833732	.0808647	.1945164	.1542294	0.207
dulo venl	-.4539591	.2208525	.004951	.080955	-.4589101	.2352208	0.051
dulo vort	-.0347382	.131541	-.1109828	.1260087	.0762445	.1831531	0.677
esci fluo	.1288183	.2063248	-.0460838	.0754116	.174902	.2196087	0.426
esci paro	.1510066	.2209788	.0410271	.0738005	.1099795	.2332714	0.637
esci sert	-.0340373	.25033	.069022	.0876949	-.1030594	.2653119	0.698
esci venl	.1061694	.2578714	.1344531	.080692	-.0282837	.2702023	0.917
fluo fluv	.1432547	.3268883	.2312358	.1049457	-.0879811	.3433256	0.798
fluo miln	.0830921	.2431603	.0794899	.1643766	.0036022	.2935315	0.990
fluo mirt	.2943965	.1806695	.0697051	.0928864	.2246914	.2034191	0.269
fluo nefa	.5708648	.4630752	-.0094023	.137485	.580267	.4830494	0.230
fluo paro	.019244	.1056897	.0974142	.0614584	-.0781702	.1221767	0.522
fluo rebo	.3145562	.1837164	.2676995	.1153572	.0468567	.2173354	0.829
fluo sert	-.1597467	.1722636	.1317876	.0769326	-.2915343	.1886872	0.122
fluo traz	.4276314	.3093663	.222172	.1203377	.2054595	.3319793	0.536
fluo venl	.122035	.1074902	.1747721	.0752073	-.0527371	.1312373	0.688
fluv miln	-.2626376	.3621303	-.1153561	.1716433	-.1472814	.4007496	0.713
fluv mirt	.158668	.2744426	-.1613906	.1252845	.3200586	.3016875	0.289
fluv paro	-.2931033	.3477839	-.1322704	.1033132	-.1608329	.3628044	0.658
fluv sert	-.8551494	.489317	-.1027194	.1112877	-.75243	.5018057	0.134
fluv venl	-.7075923	.4799498	-.0332143	.1077533	-.6743781	.4918969	0.170
miln paro	.0156181	.2053644	-.0170514	.1785608	.0326695	.2721706	0.904
miln sert	-.5332985	.5840223	.0376363	.1493491	-.5709348	.6028161	0.344
mirt paro	.1593249	.1844662	-.0878227	.0914471	.2471475	.2058905	0.230
mirt sert	-.2698151	.3224156	-.0115051	.0984112	-.25831	.3371003	0.444
mirt traz	.0843296	.299112	.142679	.138093	-.0583493	.3297285	0.860
mirt venl	.3137912	.2260323	-.0083831	.0956875	.3221743	.2454522	0.189
nefa paro	-.0568818	.3328155	.0575481	.1419699	-.1144298	.3617328	0.752
nefa sert	.0016964	.4115949	.0510046	.1456874	-.0493083	.4366179	0.910
paro rebo	.2307754	.1646449	.1890859	.1185038	.0416895	.2029929	0.837
paro sert	.135232	.2510286	-.0054009	.0729908	.1406328	.2614221	0.591
paro traz	.3177554	.3235305	.1516761	.1189406	.1660793	.3447228	0.630
paro venl	-.2014348	.2064662	.1087371	.0663071	-.3101719	.2169694	0.153
sert traz	-.5618109	.628627	.1937958	.122931	-.7556067	.6405341	0.238
sert venl	.1802162	.2584007	.0637154	.0812185	.1165007	.2708656	0.667
traz venl	-.1514985	.3865997	-.0856581	.1225909	-.0658405	.4057546	0.871
venl vort	-.4214359	.2281205	.0515964	.1003368	-.4730323	.2495049	0.058

7.4.2.3 Design-by-treatment test

chi2(137) = 134.86

Prob > chi2 = 0.2190

7.4.3 Efficacy continuous

7.4.3.1 Loop-specific approach

Loops With Evidence of Statistical Inconsistency:

Loop	IF	seIF	z_value	p_value	CI_95	Loop_Heterog_tau2
clom-fluo-miln	1.015	0.284	3.570	0.000	(0.46,1.57)	0.000
amit-clom-miln-sert	0.862	0.292	2.957	0.003	(0.29,1.43)	0.000
clom-fluv-miln	0.724	0.337	2.151	0.031	(0.06,1.38)	0.000
cita-clom-fluo	0.590	0.257	2.293	0.022	(0.09,1.09)	0.000
agom-dulo-esci	0.433	0.195	2.216	0.027	(0.05,0.82)	0.008
agom-paro-venl	0.426	0.193	2.211	0.027	(0.05,0.80)	0.017
Plac-cita-fluv	0.424	0.162	2.611	0.009	(0.11,0.74)	0.000
agom-dulo-venl	0.357	0.147	2.424	0.015	(0.07,0.65)	0.000
dulo-venl-vort	0.341	0.114	3.002	0.003	(0.12,0.56)	0.000
Plac-dulo-esci	0.263	0.115	2.286	0.022	(0.04,0.49)	0.017
Plac-amit-paro	0.245	0.089	2.748	0.006	(0.07,0.42)	0.009
Plac-amit-sert	0.173	0.086	2.016	0.044	(0.00,0.34)	0.001
Plac-cita-esci	0.137	0.063	2.172	0.030	(0.01,0.26)	0.000

Loops Without Evidence of Statistical Inconsistency:

Loop	IF	seIF	z_value	p_value	CI_95	Loop_Heterog_tau2
amit-clom-miln-venl	0.876	0.545	1.608	0.108	(0.00,1.94)	0.053
clom-miln-paro	0.609	0.415	1.468	0.142	(0.00,1.42)	0.038
cita-clom-venl	0.579	0.329	1.757	0.079	(0.00,1.22)	0.000
cita-clom-sert	0.503	0.336	1.496	0.135	(0.00,1.16)	0.017
cita-clom-paro	0.488	0.521	0.936	0.349	(0.00,1.51)	0.070
clom-fluv-sert	0.436	0.296	1.473	0.141	(0.00,1.02)	0.000
clom-fluo-fluv	0.435	0.289	1.504	0.133	(0.00,1.00)	0.000
cita-clom-fluv	0.403	0.328	1.229	0.219	(0.00,1.05)	0.000
cita-mirt-sert-traz	0.400	0.548	0.729	0.466	(0.00,1.47)	0.037
cita-fluv-sert	0.338	0.330	1.025	0.305	(0.00,0.99)	0.023
bupr-sert-traz	0.321	0.569	0.564	0.573	(0.00,1.44)	0.000
Plac-mirt-traz	0.321	0.378	0.849	0.396	(0.00,1.06)	0.042
amit-mirt-traz	0.306	0.277	1.106	0.269	(0.00,0.85)	0.000
amit-fluv-venl	0.290	0.316	0.918	0.359	(0.00,0.91)	0.000
agom-esci-paro	0.287	0.172	1.665	0.096	(0.00,0.63)	0.013
amit-fluv-miln	0.284	0.379	0.749	0.454	(0.00,1.03)	0.040
Plac-fluv-venl	0.274	0.291	0.941	0.346	(0.00,0.84)	0.007
Plac-bupr-traz	0.272	0.332	0.820	0.412	(0.00,0.92)	0.031
esci-paro-venl	0.262	0.201	1.305	0.192	(0.00,0.66)	0.016
amit-traz-venl	0.259	0.263	0.985	0.325	(0.00,0.77)	0.000
paro-sert-traz	0.253	0.231	1.096	0.273	(0.00,0.70)	0.000
Plac-traz-venl	0.252	0.247	1.019	0.308	(0.00,0.74)	0.014
cita-fluo-fluv	0.247	0.200	1.238	0.216	(0.00,0.64)	0.000
fluv-miln-paro	0.245	0.222	1.102	0.270	(0.00,0.68)	0.000
amit-paro-venl	0.245	0.230	1.064	0.287	(0.00,0.70)	0.020
Plac-paro-traz	0.243	0.165	1.475	0.140	(0.00,0.57)	0.010
cita-esci-paro	0.238	0.169	1.408	0.159	(0.00,0.57)	0.005
Plac-amit-fluo	0.238	0.126	1.882	0.060	(0.00,0.48)	0.026
Plac-cita-rebo	0.228	0.236	0.968	0.333	(0.00,0.69)	0.021
mirt-paro-venl	0.226	0.326	0.695	0.487	(0.00,0.86)	0.057
clom-fluo-paro	0.217	0.197	1.102	0.271	(0.00,0.60)	0.017
dulo-esci-fluo	0.210	0.182	1.154	0.249	(0.00,0.57)	0.002

Plac-cita-paro	0.208	0.148	1.403	0.161	(0.00,0.50)	0.004
Plac-dulo-vort	0.206	0.107	1.923	0.054	(0.00,0.42)	0.024
fluo-miln-paro	0.205	0.164	1.254	0.210	(0.00,0.53)	0.003
bupr-paro-venl	0.201	0.224	0.900	0.368	(0.00,0.64)	0.000
fluo-paro-traz	0.195	0.237	0.825	0.409	(0.00,0.66)	0.010
Plac-amit-traz	0.192	0.244	0.784	0.433	(0.00,0.67)	0.021
amit-paro-traz	0.190	0.241	0.788	0.431	(0.00,0.66)	0.013
paro-rebo-venl	0.187	0.199	0.939	0.348	(0.00,0.58)	0.002
Plac-cita-venl	0.186	0.183	1.019	0.308	(0.00,0.54)	0.003
Plac-amit-fluv	0.186	0.202	0.922	0.357	(0.00,0.58)	0.007
Plac-esci-venl	0.182	0.118	1.547	0.122	(0.00,0.41)	0.006
fluo-fluv-venl	0.181	0.285	0.635	0.525	(0.00,0.74)	0.000
cita-fluo-mirt	0.175	0.225	0.778	0.437	(0.00,0.62)	0.012
fluv-mirt-sert-traz	0.173	0.387	0.446	0.656	(0.00,0.93)	0.014
Plac-cita-mirt	0.172	0.163	1.054	0.292	(0.00,0.49)	0.004
Plac-amit-mirt	0.168	0.176	0.956	0.339	(0.00,0.51)	0.023
agom-fluo-venl	0.168	0.113	1.488	0.137	(0.00,0.39)	0.003
amit-fluv-mirt	0.167	0.249	0.671	0.502	(0.00,0.66)	0.012
Plac-amit-venl	0.166	0.160	1.038	0.299	(0.00,0.48)	0.009
clom-paro-sert	0.166	0.310	0.535	0.593	(0.00,0.77)	0.040
dulo-esci-paro	0.165	0.130	1.277	0.202	(0.00,0.42)	0.009
Plac-paro-venl	0.165	0.111	1.485	0.138	(0.00,0.38)	0.006
Plac-fluv-paro	0.165	0.198	0.831	0.406	(0.00,0.55)	0.006
fluo-traz-venl	0.164	0.266	0.616	0.538	(0.00,0.68)	0.006
fluo-nefa-paro	0.164	0.214	0.765	0.444	(0.00,0.58)	0.002
Plac-fluo-traz	0.163	0.283	0.574	0.566	(0.00,0.72)	0.035
Plac-nefa-paro	0.161	0.170	0.947	0.344	(0.00,0.50)	0.007
fluo-fluv-sert	0.157	0.205	0.766	0.444	(0.00,0.56)	0.000
clom-fluo-sert	0.156	0.199	0.785	0.432	(0.00,0.55)	0.000
cita-esci-fluo	0.155	0.119	1.302	0.193	(0.00,0.39)	0.001
cita-paro-sert	0.150	0.286	0.524	0.601	(0.00,0.71)	0.018
fluo-mirt-paro	0.147	0.176	0.840	0.401	(0.00,0.49)	0.022
fluo-sert-traz	0.146	0.270	0.540	0.589	(0.00,0.67)	0.002
fluo-fluv-miln	0.144	0.221	0.652	0.514	(0.00,0.58)	0.000
Plac-agom-venl	0.144	0.131	1.101	0.271	(0.00,0.40)	0.013
cita-esci-sert	0.144	0.148	0.972	0.331	(0.00,0.43)	0.006
bupr-fluo-venl	0.144	0.162	0.889	0.374	(0.00,0.46)	0.004
fluo-paro-sert	0.141	0.121	1.162	0.245	(0.00,0.38)	0.001
clom-fluv-paro	0.138	0.394	0.350	0.727	(0.00,0.91)	0.049
mirt-paro-traz	0.137	0.442	0.311	0.756	(0.00,1.00)	0.072
cita-fluo-rebo	0.137	0.203	0.673	0.501	(0.00,0.54)	0.010
esci-fluo-venl	0.133	0.125	1.061	0.289	(0.00,0.38)	0.001
cita-esci-venl	0.132	0.214	0.619	0.536	(0.00,0.55)	0.005
Plac-fluv-mirt	0.132	0.201	0.658	0.511	(0.00,0.53)	0.022
fluv-paro-sert	0.130	0.238	0.547	0.585	(0.00,0.60)	0.000
cita-mirt-paro	0.130	0.524	0.248	0.804	(0.00,1.16)	0.099
Plac-desv-dulo	0.129	0.190	0.678	0.498	(0.00,0.50)	0.018
Plac-bupr-venl	0.129	0.187	0.691	0.489	(0.00,0.49)	0.014
cita-paro-venl	0.129	0.223	0.578	0.563	(0.00,0.57)	0.000
bupr-paro-sert	0.126	0.540	0.233	0.816	(0.00,1.18)	0.000
agom-fluo-paro	0.125	0.108	1.164	0.245	(0.00,0.34)	0.009
clom-fluo-venl	0.123	0.250	0.491	0.624	(0.00,0.61)	0.000
agom-esci-fluo	0.122	0.131	0.934	0.350	(0.00,0.38)	0.001
dulo-fluo-venl	0.119	0.167	0.714	0.475	(0.00,0.45)	0.000
amit-fluo-mirt	0.119	0.180	0.658	0.510	(0.00,0.47)	0.011
Plac-agom-paro	0.118	0.091	1.295	0.195	(0.00,0.30)	0.013
Plac-agom-dulo	0.116	0.208	0.560	0.575	(0.00,0.52)	0.028
cita-fluo-venl	0.115	0.190	0.606	0.545	(0.00,0.49)	0.001
Plac-fluo-fluv	0.113	0.221	0.510	0.610	(0.00,0.55)	0.028
agom-esci-venl	0.108	0.177	0.610	0.542	(0.00,0.46)	0.005
fluo-paro-venl	0.107	0.117	0.921	0.357	(0.00,0.34)	0.004
amit-fluv-paro	0.105	0.285	0.369	0.712	(0.00,0.66)	0.016
fluo-fluv-paro	0.103	0.216	0.475	0.635	(0.00,0.53)	0.003
cita-fluv-mirt	0.102	0.208	0.490	0.624	(0.00,0.51)	0.000
cita-fluo-paro	0.101	0.177	0.573	0.567	(0.00,0.45)	0.005
Plac-dulo-venl	0.098	0.139	0.701	0.483	(0.00,0.37)	0.019
Plac-venl-vort	0.097	0.152	0.640	0.522	(0.00,0.39)	0.025
amit-fluo-sert	0.096	0.116	0.821	0.411	(0.00,0.32)	0.000
amit-cita-mirt-sert	0.095	0.189	0.500	0.617	(0.00,0.47)	0.000
Plac-esci-paro	0.094	0.087	1.073	0.283	(0.00,0.26)	0.006
amit-sert-venl	0.092	0.176	0.523	0.601	(0.00,0.44)	0.000
bupr-fluo-traz	0.091	0.499	0.182	0.856	(0.00,1.07)	0.050
esci-paro-sert	0.084	0.139	0.601	0.548	(0.00,0.36)	0.002
Plac-fluo-mirt	0.083	0.170	0.487	0.626	(0.00,0.42)	0.031
amit-fluo-traz	0.082	0.309	0.265	0.791	(0.00,0.69)	0.022
Plac-cita-sert	0.081	0.096	0.847	0.397	(0.00,0.27)	0.000
agom-dulo-fluo	0.079	0.203	0.390	0.697	(0.00,0.48)	0.005

Plac-bupr-paro	0.079	0.221	0.358	0.720	(0.00,0.51)	0.012
amit-fluo-fluv	0.079	0.221	0.358	0.720	(0.00,0.51)	0.004
Plac-agom-esci	0.075	0.132	0.563	0.573	(0.00,0.33)	0.010
amit-fluo-miln	0.074	0.288	0.257	0.797	(0.00,0.64)	0.023
Plac-sert-venl	0.073	0.132	0.550	0.583	(0.00,0.33)	0.004
fluv-mirt-paro	0.072	0.357	0.202	0.840	(0.00,0.77)	0.065
fluo-mirt-venl	0.068	0.146	0.464	0.642	(0.00,0.35)	0.004
nefa-paro-sert	0.064	0.229	0.278	0.781	(0.00,0.51)	0.000
clom-paro-venl	0.063	0.439	0.143	0.886	(0.00,0.92)	0.057
esci-fluo-sert	0.062	0.127	0.485	0.628	(0.00,0.31)	0.000
Plac-sert-traz	0.061	0.219	0.277	0.781	(0.00,0.49)	0.007
clom-sert-venl	0.059	0.302	0.196	0.845	(0.00,0.65)	0.011
amit-fluo-paro	0.059	0.120	0.491	0.623	(0.00,0.29)	0.010
Plac-esci-sert	0.059	0.092	0.635	0.525	(0.00,0.24)	0.000
bupr-paro-traz	0.058	0.281	0.205	0.838	(0.00,0.61)	0.000
amit-miln-paro	0.058	0.236	0.244	0.807	(0.00,0.52)	0.017
cita-fluo-sert	0.057	0.133	0.428	0.669	(0.00,0.32)	0.000
Plac-cita-fluo	0.056	0.154	0.363	0.717	(0.00,0.36)	0.020
bupr-fluo-sert	0.054	0.514	0.105	0.916	(0.00,1.06)	0.000
bupr-sert-venl	0.052	0.528	0.099	0.921	(0.00,1.09)	0.000
Plac-paro-sert	0.051	0.108	0.473	0.636	(0.00,0.26)	0.004
fluo-paro-rebo	0.049	0.116	0.420	0.674	(0.00,0.28)	0.005
amit-fluo-venl	0.048	0.157	0.304	0.761	(0.00,0.36)	0.003
amit-paro-sert	0.047	0.129	0.366	0.714	(0.00,0.30)	0.003
Plac-fluo-rebo	0.047	0.170	0.278	0.781	(0.00,0.38)	0.036
fluo-fluv-mirt	0.047	0.238	0.197	0.844	(0.00,0.51)	0.018
cita-fluv-paro	0.046	0.246	0.188	0.851	(0.00,0.53)	0.000
Plac-nefa-sert	0.046	0.183	0.252	0.801	(0.00,0.40)	0.000
bupr-fluo-paro	0.045	0.225	0.201	0.840	(0.00,0.49)	0.009
dulo-paro-venl	0.045	0.152	0.298	0.766	(0.00,0.34)	0.006
cita-fluv-venl	0.045	0.332	0.136	0.892	(0.00,0.70)	0.000
Plac-fluo-paro	0.045	0.077	0.580	0.562	(0.00,0.20)	0.018
esci-sert-venl	0.043	0.193	0.221	0.825	(0.00,0.42)	0.007
Plac-fluo-nefa	0.042	0.220	0.193	0.847	(0.00,0.47)	0.028
Plac-agom-fluo	0.041	0.125	0.328	0.743	(0.00,0.29)	0.027
fluo-nefa-sert	0.040	0.240	0.169	0.866	(0.00,0.51)	0.000
cita-mirt-venl	0.040	0.224	0.180	0.857	(0.00,0.48)	0.000
dulo-esci-venl	0.039	0.171	0.228	0.819	(0.00,0.37)	0.009
cita-rebo-venl	0.039	0.252	0.153	0.878	(0.00,0.53)	0.000
dulo-fluo-paro	0.038	0.170	0.224	0.823	(0.00,0.37)	0.005
Plac-bupr-esci	0.037	0.137	0.268	0.788	(0.00,0.31)	0.012
fluo-rebo-venl	0.034	0.189	0.181	0.857	(0.00,0.41)	0.004
Plac-mirt-paro	0.032	0.132	0.243	0.808	(0.00,0.29)	0.014
Plac-fluo-venl	0.030	0.082	0.372	0.710	(0.00,0.19)	0.018
Plac-esci-fluo	0.030	0.125	0.240	0.810	(0.00,0.28)	0.018
fluo-sert-venl	0.029	0.136	0.213	0.831	(0.00,0.30)	0.000
Plac-mirt-venl	0.024	0.151	0.161	0.872	(0.00,0.32)	0.013
Plac-fluo-sert	0.024	0.111	0.217	0.828	(0.00,0.24)	0.018
bupr-esci-sert	0.023	0.518	0.044	0.965	(0.00,1.04)	0.000
bupr-esci-paro	0.022	0.234	0.093	0.926	(0.00,0.48)	0.007
amit-mirt-venl	0.021	0.208	0.102	0.919	(0.00,0.43)	0.007
Plac-dulo-paro	0.020	0.086	0.236	0.813	(0.00,0.19)	0.016
Plac-paro-rebo	0.020	0.125	0.161	0.872	(0.00,0.26)	0.016
esci-fluo-paro	0.020	0.119	0.165	0.869	(0.00,0.25)	0.005
amit-sert-traz	0.019	0.247	0.078	0.938	(0.00,0.50)	0.000
agom-dulo-paro	0.018	0.175	0.100	0.920	(0.00,0.36)	0.011
amit-mirt-paro	0.015	0.216	0.070	0.944	(0.00,0.44)	0.036
bupr-esci-venl	0.015	0.208	0.072	0.943	(0.00,0.42)	0.007
amit-fluv-sert	0.015	0.215	0.069	0.945	(0.00,0.44)	0.000
cita-paro-rebo	0.014	0.176	0.079	0.937	(0.00,0.36)	0.000
Plac-dulo-fluo	0.013	0.215	0.062	0.951	(0.00,0.43)	0.033
Plac-bupr-sert	0.011	0.525	0.021	0.984	(0.00,1.04)	0.010
Plac-cita-vila	0.008	0.135	0.058	0.954	(0.00,0.27)	0.013
Plac-bupr-fluo	0.006	0.163	0.040	0.968	(0.00,0.33)	0.032
fluo-mirt-traz	0.006	0.460	0.013	0.989	(0.00,0.91)	0.058
Plac-fluv-sert	0.005	0.175	0.031	0.975	(0.00,0.35)	0.000
bupr-esci-fluo	0.004	0.157	0.026	0.979	(0.00,0.31)	0.004
cita-sert-venl	0.004	0.384	0.010	0.992	(0.00,0.76)	0.038
Plac-rebo-venl	0.003	0.259	0.013	0.990	(0.00,0.51)	0.023

7.4.3.2 Side-splitting

Side	Direct Coef.	Std. Err.	Indirect Coef.	Std. Err.	Difference Coef.	Std. Err.	P>|z|
Plac agom	-.2512808	.0459306	-.2660282	.0514991	.0147474	.0690935	0.831
Plac amit	-.6103598	.0519063	-.3732179	.043578	-.2371419	.0677481	0.000
Plac bupr	-.2024932	.0497705	-.3410445	.0824924	.1385513	.0962711	0.150
Plac cita	-.2098557	.0507747	-.2640504	.0462435	.0541946	.0688042	0.431
Plac dulo	-.375099	.0383956	-.3658008	.0518621	-.0092981	.0650107	0.886
Plac esci	-.2150141	.0373355	-.3857045	.0418394	.1706904	.0562674	0.002
Plac fluo	-.2087802	.0315559	-.2427501	.0311074	.0339699	.0443257	0.443
Plac fluv	-.4378883	.098869	-.2742997	.0604566	-.1635886	.1159017	0.158
Plac mirt	-.3637403	.0683478	-.3603649	.0543432	-.0033754	.0873718	0.969
Plac nefa	-.3026198	.0781393	-.2264457	.1031057	-.076174	.1293746	0.556
Plac paro	-.2929862	.0291566	-.3493622	.032029	.056376	.0433307	0.193
Plac rebo	-.1714061	.0604265	-.1395756	.0733	-.0318305	.0949709	0.738
Plac sert	-.2721599	.045185	-.2708246	.0437179	-.0013353	.0628841	0.983
Plac traz	-.3486727	.0814432	-.2014595	.0825684	-.1472132	.115877	0.204
Plac venl	-.336268	.0371874	-.3245724	.0368772	-.0116956	.0523786	0.823
Plac vort	-.3014117	.0416346	-.1612856	.097015	-.1401262	.1065828	0.189
agom dulo	-.237261	.1513232	-.1026529	.0458873	-.1346081	.1581276	0.395
agom esci	.1119963	.1271535	-.0508843	.0445176	.1628806	.134717	0.227
agom fluo	-.0017993	.0752683	.043039	.0435064	-.0448383	.0870529	0.607
agom paro	-.160856	.071295	-.0234497	.0435325	-.1374064	.0835343	0.100
agom venl	.0602802	.1144701	-.0914764	.043244	.1517565	.1223642	0.215
amit fluo	.1704286	.0873593	.2619879	.040509	-.0915594	.0962961	0.342
amit fluv	-.021912	.1818389	.1729215	.0614824	-.1948335	.1919517	0.310
amit miln	.0846067	.1650324	.1978638	.082096	-.113257	.1843328	0.539
amit mirt	.0811044	.1187676	.1166636	.0562391	-.0355592	.1314063	0.787
amit paro	.0723168	.0685035	.1832981	.0417883	-.1109813	.0802468	0.167
amit sert	.1876655	.0732317	.2062233	.0497213	-.0185578	.0885043	0.834
amit traz	.0726626	.176609	.2146241	.0693656	-.1419615	.1897242	0.454
amit venl	.109774	.147427	.1440288	.0418692	-.0342549	.153257	0.823
bupr esci	-.0525734	.1188586	-.0509015	.0543524	-.0016719	.1306898	0.990
bupr fluo	-.0157645	.1023828	.021155	.051824	-.0369195	.1146863	0.748
bupr paro	-.0140928	.2082742	-.0824246	.0479194	.0683318	.2137157	0.749
bupr sert	-.0629887	.5168931	-.0315514	.0523925	-.0314373	.5195416	0.952
bupr traz	.1271639	.2220119	-.0545544	.0736182	.1817183	.2338994	0.437
bupr venl	-.0013175	.1594447	-.099801	.0508484	.0984835	.1673564	0.556
cita clom	-.37479	.2300268	-.0595383	.0705494	-.3152517	.2406025	0.190
cita esci	-.1302309	.0557588	.0155008	.051377	-.1457316	.0757714	0.054
cita fluo	.0536987	.1145703	.0084047	.040552	.045294	.1215348	0.709
cita fluv	.1855169	.1772178	-.112525	.0626899	.2980419	.1879792	0.113
cita mirt	.0202473	.167092	-.1375008	.0547901	.1577481	.1758457	0.370
cita paro	.1194218	.167554	-.0904116	.0395137	.2098333	.1723919	0.224
cita rebo	.2558837	.1601036	.0573587	.0589473	.198525	.1706105	0.245
cita sert	.0355478	.1142018	-.0434445	.047032	.0789923	.1235181	0.522
cita venl	.0658867	.2002698	-.0977906	.0421119	.1636774	.2046495	0.424
cita vila	-.034457	.1100089	-.0254496	.0752578	-.0090074	.133315	0.946
clom fluo	-.1596859	.1586681	.1471659	.0675902	-.3068518	.1724635	0.075
clom fluv	.1574758	.2355193	-.0100022	.0799784	.167478	.2487286	0.501
clom miln	.6887981	.2416763	-.0678511	.09298	.7566492	.2589454	0.003
clom paro	.006088	.0870943	.0090016	.083562	-.0029136	.1207017	0.981
clom sert	-.0885061	.1497974	.087926	.0721373	-.1764322	.1662697	0.289
clom venl	-.1378382	.2334831	.0068743	.0670299	-.1447125	.2429143	0.551
desv dulo	-.0278205	.1506692	-.1426767	.0614205	.1148563	.163147	0.481
dulo esci	-.091101	.0902523	.1197651	.0428972	-.2108661	.0999293	0.035
dulo fluo	.143706	.1638625	.1458712	.0372474	-.0021652	.16835	0.990
dulo paro	.0919989	.0628171	.0360679	.041534	.0559311	.0755905	0.459
dulo venl	-.0593003	.1089809	.0553195	.0404329	-.1146198	.1162357	0.324
dulo vort	.2003974	.0640412	-.0011567	.0599429	.2015541	.0880397	0.022
esci fluo	.0255431	.0987587	.069917	.0354663	-.0443739	.1049367	0.672
esci paro	.0125007	.0897985	-.0341039	.0355654	.0466046	.0966372	0.630
esci sert	.0147888	.1050175	.0201744	.0429075	-.0053856	.1134523	0.962
esci venl	.0700348	.1224274	-.0500007	.0379761	.1200355	.1281963	0.349
fluo fluv	-.1095842	.1478212	-.0903056	.0575448	-.0192786	.158625	0.903
fluo miln	-.1652954	.1686345	-.0499987	.0782099	-.1152966	.1858881	0.535
fluo mirt	-.2110616	.1090864	-.1201967	.0493536	-.0908649	.1197584	0.448
fluo nefa	-.0418493	.1723411	-.0499643	.0698117	.008115	.1859457	0.965
fluo paro	-.0440312	.054283	-.1080671	.0306567	.0640359	.0623394	0.304
fluo rebo	.0633928	.0938962	.0690271	.0573141	-.0056343	.1099872	0.959
fluo sert	-.0863324	.0846608	-.0368474	.0387958	-.049485	.0931402	0.595
fluo traz	-.0369502	.1933362	-.05157	.063679	.0146198	.2035894	0.943
fluo venl	-.1317808	.0524652	-.0909532	.0366298	-.0408276	.0639789	0.523

fluv miln	-.1917567	.1586241	.0944508	.0918993	-.2862075	.1833224	0.118
fluv mirt	-.0640723	.1282119	-.036494	.0698865	-.0275783	.1460215	0.850
fluv paro	-.0104914	.1826039	.0012551	.0559455	-.0117465	.190985	0.951
fluv sert	.1948878	.171668	.0287214	.0611204	.1661664	.1822251	0.362
fluv venl	-.1647263	.2789115	-.0049965	.0573374	-.1597299	.2847441	0.575
miln paro	-.0658516	.1052015	.0115019	.0924334	-.0773535	.1400403	0.581
mirt paro	.0394475	.1000604	.0439373	.0499147	-.0044898	.1118342	0.968
mirt traz	.3008748	.23366	.0643191	.073306	.2365557	.2449005	0.334
mirt venl	.0052397	.1218664	.035896	.051105	-.0306563	.1321482	0.817
nefa paro	-.1537458	.1612114	-.0228335	.0703625	-.1309123	.1758773	0.457
nefa sert	2.67e-08	.2026366	.0037946	.0722005	-.0037945	.2151151	0.986
paro rebo	.1506427	.0869293	.164429	.0587844	-.0137863	.1049623	0.896
paro sert	.0658566	.1194948	.0453614	.037182	.0204952	.1251588	0.870
paro traz	.1899164	.1398435	.0094342	.0661789	.1804823	.1547145	0.243
paro venl	-.1883823	.1180784	.0017336	.0325076	-.1901159	.1225212	0.121
rebo venl	-.1513165	.1927701	-.1734378	.0536946	.0221213	.2001085	0.912
sert traz	-.1307871	.2149651	.0075863	.0670861	-.1383735	.22519	0.539
sert venl	.0093448	.1387856	-.0646365	.0402715	.0739813	.1445103	0.609
traz venl	-.2272443	.2064317	-.0366811	.0653753	-.1905633	.2167323	0.379
venl vort	-.0864418	.1102267	.0779534	.0480877	-.1643952	.1204024	0.172

7.4.3.3 Design-by-treatment test

chi2(134) = 106.50

Prob > chi2 = 0.7872

7.4.4 Remission

7.4.4.1 Loop-specific approach

Loops With Evidence of Statistical Inconsistency:

Loop	IF	seIF	z_value	p_value	CI_95	Loop_Heterog_tau2
cita-clom-venl	2.334	0.710	3.288	0.001	(0.94,3.73)	0.000
cita-clom-fluo	1.707	0.493	3.464	0.001	(0.74,2.67)	0.000
cita-clom-sert	1.332	0.573	2.323	0.020	(0.21,2.46)	0.031
cita-esci-venl	1.230	0.596	2.063	0.039	(0.06,2.40)	0.140
clom-fluo-miln	1.108	0.543	2.041	0.041	(0.04,2.17)	0.000
Plac-cita-venl	1.039	0.350	2.970	0.003	(0.35,1.73)	0.000
agom-dulo-venl	1.006	0.326	3.090	0.002	(0.37,1.64)	0.000
agom-dulo-esci	0.862	0.404	2.132	0.033	(0.07,1.65)	0.028
cita-fluo-venl	0.823	0.387	2.126	0.034	(0.06,1.58)	0.007
Plac-bupr-venl	0.674	0.261	2.585	0.010	(0.16,1.19)	0.000
Plac-agom-venl	0.587	0.216	2.719	0.007	(0.16,1.01)	0.005
Plac-amit-fluo	0.518	0.229	2.260	0.024	(0.07,0.97)	0.066
Plac-amit-paro	0.450	0.162	2.787	0.005	(0.13,0.77)	0.000

Loops Without Evidence of Statistical Inconsistency:

Loop	IF	seIF	z_value	p_value	CI_95	Loop_Heterog_tau2
cita-clom-paro	1.534	0.947	1.620	0.105	(0.00,3.39)	0.206
cita-clom-fluv	1.444	0.783	1.846	0.065	(0.00,2.98)	0.000
bupr-sert-traz	1.428	1.218	1.173	0.241	(0.00,3.82)	0.000
cita-sert-venl	1.159	0.675	1.717	0.086	(0.00,2.48)	0.189
cita-paro-venl	1.101	0.840	1.312	0.190	(0.00,2.75)	0.185
agom-paro-venl	1.082	0.565	1.915	0.056	(0.00,2.19)	0.158
cita-fluv-sert	1.032	0.743	1.389	0.165	(0.00,2.49)	0.111
clom-fluo-fluv	0.959	0.604	1.588	0.112	(0.00,2.14)	0.000
bupr-paro-venl	0.943	0.735	1.283	0.199	(0.00,2.38)	0.107
clom-fluv-sert	0.906	0.649	1.397	0.162	(0.00,2.18)	0.000
bupr-sert-venl	0.889	1.288	0.691	0.490	(0.00,3.41)	0.157

fluo-traz-venl	0.855	0.570	1.500	0.134	(0.00,1.97)	0.000
fluv-mirt-venl	0.841	0.515	1.632	0.103	(0.00,1.85)	0.000
Plac-cita-fluv	0.834	0.492	1.694	0.090	(0.00,1.80)	0.000
mirt-sert-traz	0.831	0.754	1.101	0.271	(0.00,2.31)	0.000
cita-fluv-venl	0.797	0.700	1.138	0.255	(0.00,2.17)	0.000
Plac-bupr-traz	0.737	0.440	1.675	0.094	(0.00,1.60)	0.011
paro-sert-traz	0.720	0.478	1.507	0.132	(0.00,1.66)	0.000
cita-mirt-venl	0.706	0.733	0.963	0.335	(0.00,2.14)	0.102
bupr-fluo-traz	0.699	0.503	1.390	0.165	(0.00,1.68)	0.000
cita-fluo-fluv	0.696	0.536	1.298	0.194	(0.00,1.75)	0.000
paro-rebo-venl	0.695	0.520	1.338	0.181	(0.00,1.71)	0.042
fluo-fluv-venl	0.692	0.500	1.383	0.167	(0.00,1.67)	0.000
cita-fluv-mirt	0.674	0.551	1.222	0.222	(0.00,1.75)	0.000
Plac-traz-venl	0.668	0.514	1.299	0.194	(0.00,1.68)	0.000
cita-esci-paro	0.609	0.543	1.120	0.263	(0.00,1.67)	0.110
Plac-fluv-venl	0.591	0.471	1.255	0.209	(0.00,1.51)	0.000
agom-esci-paro	0.569	0.519	1.096	0.273	(0.00,1.59)	0.121
fluo-sert-traz	0.535	0.480	1.115	0.265	(0.00,1.47)	0.000
Plac-sert-traz	0.535	0.582	0.919	0.358	(0.00,1.68)	0.106
amit-traz-venl	0.528	0.613	0.862	0.389	(0.00,1.73)	0.000
amit-fluo-miln	0.526	0.499	1.055	0.292	(0.00,1.50)	0.000
bupr-esci-venl	0.514	0.427	1.203	0.229	(0.00,1.35)	0.029
cita-esci-sert	0.507	0.513	0.987	0.324	(0.00,1.51)	0.143
amit-fluv-venl	0.502	0.649	0.773	0.439	(0.00,1.77)	0.043
dulo-esci-fluo	0.484	0.456	1.063	0.288	(0.00,1.38)	0.062
amit-sert-traz	0.476	0.679	0.701	0.483	(0.00,1.81)	0.097
bupr-paro-traz	0.474	0.593	0.800	0.424	(0.00,1.64)	0.000
clom-fluo-paro	0.468	0.350	1.336	0.181	(0.00,1.15)	0.064
dulo-venl-vort	0.461	0.329	1.402	0.161	(0.00,1.11)	0.045
Plac-nefa-paro	0.461	0.383	1.205	0.228	(0.00,1.21)	0.000
dulo-fluo-venl	0.459	0.353	1.299	0.194	(0.00,1.15)	0.000
Plac-rebo-venl	0.459	0.385	1.191	0.234	(0.00,1.21)	0.005
cita-esci-fluo	0.453	0.434	1.043	0.297	(0.00,1.30)	0.127
fluv-paro-sert	0.445	0.522	0.852	0.394	(0.00,1.47)	0.000
amit-clom-miln-sert	0.443	0.951	0.465	0.642	(0.00,2.31)	0.107
Plac-cita-paro	0.439	0.300	1.464	0.143	(0.00,1.03)	0.000
bupr-fluo-venl	0.436	0.293	1.489	0.136	(0.00,1.01)	0.000
fluv-mirt-paro	0.433	0.446	0.971	0.332	(0.00,1.31)	0.000
clom-miln-paro	0.432	0.722	0.598	0.550	(0.00,1.85)	0.111
Plac-fluo-nefa	0.419	0.473	0.885	0.376	(0.00,1.35)	0.100
esci-sert-venl	0.418	0.401	1.043	0.297	(0.00,1.20)	0.025
Plac-amit-venl	0.409	0.301	1.360	0.174	(0.00,1.00)	0.000
clom-fluo-sert	0.400	0.373	1.072	0.284	(0.00,1.13)	0.000
agom-fluo-venl	0.385	0.221	1.743	0.081	(0.00,0.82)	0.000
amit-miln-paro	0.380	0.405	0.937	0.349	(0.00,1.17)	0.000
Plac-amit-mirt	0.373	0.313	1.192	0.233	(0.00,0.99)	0.000
dulo-fluo-paro	0.370	0.347	1.064	0.287	(0.00,1.05)	0.013
esci-paro-venl	0.362	0.335	1.082	0.279	(0.00,1.02)	0.023
amit-nefa-paro	0.362	0.754	0.480	0.631	(0.00,1.84)	0.000
Plac-amit-nefa	0.351	0.694	0.505	0.613	(0.00,1.71)	0.000
fluo-mirt-traz	0.347	0.698	0.497	0.619	(0.00,1.72)	0.000
fluo-fluv-sert	0.347	0.429	0.808	0.419	(0.00,1.19)	0.000
fluo-rebo-venl	0.341	0.395	0.864	0.387	(0.00,1.12)	0.000
Plac-amit-fluv	0.340	0.331	1.026	0.305	(0.00,0.99)	0.000
cita-paro-sert	0.340	0.497	0.684	0.494	(0.00,1.31)	0.048
Plac-cita-esci	0.338	0.179	1.882	0.060	(0.00,0.69)	0.042
Plac-cita-rebo	0.334	0.322	1.035	0.301	(0.00,0.97)	0.027
Plac-amit-sert	0.332	0.274	1.211	0.226	(0.00,0.87)	0.079
amit-paro-venl	0.331	0.365	0.908	0.364	(0.00,1.05)	0.017
Plac-agom-esci	0.328	0.285	1.151	0.250	(0.00,0.89)	0.034
amit-fluo-nefa	0.327	0.775	0.421	0.674	(0.00,1.85)	0.000
fluo-paro-venl	0.321	0.222	1.449	0.147	(0.00,0.76)	0.017
Plac-agom-dulo	0.318	0.362	0.880	0.379	(0.00,1.03)	0.057
mirt-paro-venl	0.315	0.351	0.898	0.369	(0.00,1.00)	0.024
amit-nefa-sert	0.314	1.146	0.274	0.784	(0.00,2.56)	0.138
clom-fluv-paro	0.309	0.801	0.386	0.700	(0.00,1.88)	0.145
amit-clom-miln-venl	0.307	0.769	0.400	0.689	(0.00,1.81)	0.000
esci-paro-sert	0.299	0.311	0.961	0.336	(0.00,0.91)	0.000
agom-dulo-paro	0.296	0.481	0.617	0.537	(0.00,1.24)	0.081
fluo-nefa-sert	0.291	0.541	0.538	0.591	(0.00,1.35)	0.000
Plac-fluv-sert	0.287	0.493	0.582	0.560	(0.00,1.25)	0.107
nefa-paro-sert	0.286	0.533	0.537	0.592	(0.00,1.33)	0.000
cita-fluo-paro	0.283	0.415	0.681	0.496	(0.00,1.10)	0.037
amit-clom-fluv-miln	0.280	0.877	0.320	0.749	(0.00,2.00)	0.056
Plac-amit-traz	0.276	0.307	0.902	0.367	(0.00,0.88)	0.000
Plac-paro-venl	0.275	0.180	1.529	0.126	(0.00,0.63)	0.000
fluo-paro-traz	0.269	0.424	0.633	0.527	(0.00,1.10)	0.030

Plac-venl-vort	0.265	0.215	1.232	0.218	(0.00,0.69)	0.024
Plac-cita-mirt	0.260	0.305	0.854	0.393	(0.00,0.86)	0.000
cita-rebo-venl	0.248	0.539	0.461	0.645	(0.00,1.30)	0.000
fluo-sert-venl	0.248	0.240	1.031	0.302	(0.00,0.72)	0.000
amit-fluo-sert	0.245	0.269	0.911	0.363	(0.00,0.77)	0.027
amit-mirt-paro	0.242	0.313	0.774	0.439	(0.00,0.86)	0.000
Plac-mirt-traz	0.242	0.684	0.353	0.724	(0.00,1.58)	0.022
agom-esci-fluo	0.237	0.356	0.666	0.505	(0.00,0.93)	0.041
bupr-paro-sert	0.235	1.170	0.200	0.841	(0.00,2.53)	0.000
dulo-paro-venl	0.232	0.253	0.917	0.359	(0.00,0.73)	0.005
Plac-esci-venl	0.228	0.215	1.058	0.290	(0.00,0.65)	0.000
amit-fluo-traz	0.226	0.404	0.560	0.575	(0.00,1.02)	0.000
fluo-miln-paro	0.225	0.394	0.571	0.568	(0.00,1.00)	0.025
Plac-dulo-esci	0.224	0.200	1.117	0.264	(0.00,0.62)	0.036
cita-fluv-paro	0.219	0.646	0.339	0.734	(0.00,1.49)	0.000
cita-fluo-rebo	0.219	0.370	0.593	0.553	(0.00,0.94)	0.023
amit-paro-sert	0.219	0.343	0.637	0.524	(0.00,0.89)	0.053
bupr-esci-fluo	0.214	0.386	0.553	0.580	(0.00,0.97)	0.061
Plac-nefa-sert	0.208	0.541	0.385	0.700	(0.00,1.27)	0.087
fluo-fluv-paro	0.205	0.488	0.420	0.675	(0.00,1.16)	0.025
amit-mirt-venl	0.197	0.429	0.458	0.647	(0.00,1.04)	0.000
Plac-bupr-sert	0.196	1.161	0.169	0.866	(0.00,2.47)	0.051
bupr-fluo-sert	0.195	1.114	0.175	0.861	(0.00,2.38)	0.000
Plac-dulo-fluo	0.194	0.389	0.500	0.617	(0.00,0.96)	0.084
clom-paro-venl	0.189	0.817	0.232	0.817	(0.00,1.79)	0.170
fluv-mirt-sert	0.188	0.547	0.343	0.731	(0.00,1.26)	0.051
Plac-fluv-paro	0.180	0.421	0.426	0.670	(0.00,1.01)	0.000
amit-mirt-traz	0.176	0.718	0.245	0.806	(0.00,1.58)	0.000
Plac-esci-paro	0.176	0.148	1.183	0.237	(0.00,0.47)	0.000
mirt-paro-sert	0.175	0.317	0.554	0.580	(0.00,0.80)	0.001
clom-fluo-venl	0.175	0.534	0.327	0.744	(0.00,1.22)	0.000
Plac-paro-traz	0.175	0.293	0.596	0.551	(0.00,0.75)	0.000
cita-mirt-sert	0.173	0.650	0.266	0.790	(0.00,1.45)	0.112
Plac-esci-sert	0.171	0.308	0.556	0.578	(0.00,0.78)	0.041
amit-fluv-sert	0.171	0.675	0.253	0.800	(0.00,1.49)	0.143
agom-fluo-paro	0.167	0.296	0.565	0.572	(0.00,0.75)	0.081
amit-mirt-sert	0.167	0.559	0.298	0.766	(0.00,1.26)	0.089
agom-dulo-fluo	0.163	0.423	0.386	0.699	(0.00,0.99)	0.012
Plac-mirt-paro	0.153	0.209	0.735	0.462	(0.00,0.56)	0.000
Plac-mirt-venl	0.149	0.264	0.565	0.572	(0.00,0.67)	0.000
Plac-paro-rebo	0.142	0.152	0.938	0.348	(0.00,0.44)	0.002
cita-fluo-mirt	0.130	0.333	0.389	0.697	(0.00,0.78)	0.000
amit-fluv-mirt	0.130	0.422	0.307	0.759	(0.00,0.96)	0.010
esci-fluo-paro	0.129	0.279	0.463	0.644	(0.00,0.68)	0.042
dulo-esci-venl	0.126	0.296	0.426	0.670	(0.00,0.71)	0.007
fluo-nefa-paro	0.125	0.538	0.233	0.816	(0.00,1.18)	0.027
Plac-fluo-paro	0.123	0.143	0.856	0.392	(0.00,0.40)	0.043
clom-paro-sert	0.121	0.563	0.215	0.830	(0.00,1.22)	0.114
fluo-paro-sert	0.116	0.272	0.426	0.670	(0.00,0.65)	0.023
Plac-fluo-venl	0.114	0.146	0.784	0.433	(0.00,0.40)	0.039
amit-sert-venl	0.114	0.562	0.203	0.839	(0.00,1.22)	0.120
Plac-dulo-venl	0.108	0.191	0.567	0.571	(0.00,0.48)	0.008
fluo-fluv-mirt	0.108	0.351	0.307	0.759	(0.00,0.80)	0.000
Plac-fluv-mirt	0.100	0.310	0.324	0.746	(0.00,0.71)	0.000
amit-fluo-venl	0.100	0.319	0.314	0.754	(0.00,0.73)	0.000
esci-fluo-venl	0.098	0.260	0.378	0.705	(0.00,0.61)	0.002
Plac-bupr-paro	0.096	0.384	0.249	0.803	(0.00,0.85)	0.000
Plac-dulo-vort	0.093	0.169	0.553	0.580	(0.00,0.42)	0.036
Plac-fluo-rebo	0.093	0.283	0.330	0.742	(0.00,0.65)	0.103
clom-fluv-venl	0.093	0.810	0.114	0.909	(0.00,1.68)	0.000
Plac-mirt-sert	0.088	0.410	0.215	0.830	(0.00,0.89)	0.071
amit-fluo-paro	0.086	0.214	0.402	0.688	(0.00,0.51)	0.013
bupr-fluo-paro	0.084	0.457	0.184	0.854	(0.00,0.98)	0.027
Plac-desv-dulo	0.081	0.305	0.266	0.790	(0.00,0.68)	0.030
Plac-bupr-esci	0.080	0.213	0.375	0.708	(0.00,0.50)	0.006
Plac-bupr-fluo	0.079	0.278	0.284	0.776	(0.00,0.62)	0.084
fluo-mirt-paro	0.075	0.244	0.306	0.759	(0.00,0.55)	0.018
Plac-fluo-sert	0.074	0.244	0.305	0.761	(0.00,0.55)	0.100
amit-fluo-fluv	0.069	0.384	0.181	0.857	(0.00,0.82)	0.000
esci-fluo-sert	0.068	0.316	0.216	0.829	(0.00,0.69)	0.009
Plac-dulo-paro	0.063	0.133	0.476	0.634	(0.00,0.32)	0.013
amit-fluv-paro	0.062	0.484	0.128	0.898	(0.00,1.01)	0.015
amit-fluo-mirt	0.055	0.327	0.168	0.867	(0.00,0.70)	0.000
Plac-agom-fluo	0.053	0.251	0.211	0.833	(0.00,0.55)	0.099
mirt-paro-traz	0.053	0.700	0.075	0.940	(0.00,1.43)	0.009
fluo-mirt-sert	0.051	0.290	0.177	0.860	(0.00,0.62)	0.000
Plac-paro-sert	0.051	0.223	0.230	0.818	(0.00,0.49)	0.012

cita-fluo-sert	0.050	0.291	0.171	0.864	(0.00,0.62)	0.010
bupr-esci-sert	0.049	1.160	0.042	0.966	(0.00,2.32)	0.032
Plac-agom-paro	0.049	0.195	0.249	0.803	(0.00,0.43)	0.037
Plac-cita-fluo	0.048	0.287	0.168	0.867	(0.00,0.61)	0.074
fluo-mirt-venl	0.042	0.267	0.157	0.876	(0.00,0.56)	0.000
agom-esci-venl	0.039	0.330	0.118	0.906	(0.00,0.69)	0.000
Plac-sert-venl	0.038	0.245	0.155	0.877	(0.00,0.52)	0.022
fluo-paro-rebo	0.036	0.247	0.147	0.883	(0.00,0.52)	0.029
Plac-fluo-mirt	0.035	0.278	0.127	0.899	(0.00,0.58)	0.087
Plac-esci-fluo	0.027	0.229	0.119	0.905	(0.00,0.48)	0.053
cita-mirt-paro	0.027	0.427	0.063	0.950	(0.00,0.86)	0.006
Plac-cita-sert	0.026	0.286	0.091	0.927	(0.00,0.59)	0.045
Plac-fluo-traz	0.026	0.414	0.062	0.951	(0.00,0.84)	0.106
dulo-esci-paro	0.023	0.221	0.106	0.915	(0.00,0.46)	0.010
clom-sert-venl	0.023	0.694	0.033	0.974	(0.00,1.38)	0.080
Plac-fluo-fluv	0.013	0.426	0.030	0.976	(0.00,0.85)	0.106
cita-paro-rebo	0.013	0.423	0.030	0.976	(0.00,0.84)	0.016
amit-paro-traz	0.001	0.371	0.002	0.998	(0.00,0.73)	0.000
Plac-cita-vila	0.001	0.204	0.004	0.997	(0.00,0.40)	0.000
bupr-esci-paro	0.001	0.502	0.001	0.999	(0.00,0.98)	0.037

7.4.4.2 Side-splitting

Side	Direct Coef.	Std. Err.	Indirect Coef.	Std. Err.	Difference Coef.	Std. Err.	P>|z|
Plac agom	.2742141	.1008079	.4267388	.0950954	-.1525247	.1387232	0.272
Plac amit	.9241333	.116775	.4925027	.0810043	.4316306	.1433067	0.003
Plac bupr	.3560292	.0993803	.7296492	.1536516	-.37362	.1835915	0.042
Plac cita	.3075801	.0990608	.3136082	.08528	-.0060281	.131428	0.963
Plac dulo	.5653745	.0679892	.5554197	.0941565	.0099547	.1180411	0.933
Plac esci	.3555811	.0745968	.6003433	.0773862	-.2447622	.1082139	0.024
Plac fluo	.30149	.0635611	.4145661	.0578933	-.1130761	.0862272	0.190
Plac fluv	.5162983	.2057231	.4702389	.122176	.0460593	.2392422	0.847
Plac mirt	.4799069	.1499081	.4787367	.0942249	.0011701	.1795984	0.995
Plac nefa	.6705887	.1813463	.3516411	.2129321	.3189476	.2796895	0.254
Plac paro	.4791893	.0571388	.4939838	.0605267	-.0147944	.0834337	0.859
Plac rebo	.18131	.1081985	.2000003	.1320203	-.0186904	.170965	0.913
Plac sert	.4028782	.1059181	.4142359	.0781377	-.0113578	.1317119	0.931
Plac traz	.3373667	.1678135	.274706	.1463917	.0626606	.2233274	0.779
Plac venl	.5789591	.0755828	.4645588	.0660139	.1144003	.1008816	0.257
Plac vort	.3780048	.0772756	.4183703	.1697759	-.0403655	.1900658	0.832
agom dulo	.6234499	.2769449	.165558	.0871584	.4578919	.2903361	0.115
agom esci	-.2207809	.2387446	.1645752	.0875731	-.3853561	.2542993	0.130
agom fluo	.0247353	.1396562	.0017863	.0873788	.022949	.1651037	0.889
agom paro	.2484744	.1396965	.086656	.0863003	.1618184	.1635326	0.322
agom venl	-.2598312	.2092811	.2274581	.0846972	-.4872893	.2257737	0.031
amit fluo	-.1423882	.1603549	-.3023517	.0785859	.1599636	.1786469	0.371
amit fluv	-.0613769	.2735419	-.1722257	.1275542	.1108488	.3017491	0.713
amit miln	-.4157315	.4037919	-.2018824	.1609912	-.2138491	.4347024	0.623
amit mirt	-.0923577	.2673307	-.1646894	.1013209	.0723316	.285852	0.800
amit nefa	.0953095	.6835875	-.1079172	.1538382	.2032267	.700684	0.772
amit paro	-.0105921	.1234971	-.2094176	.0819697	.1988256	.1482359	0.180
amit sert	-.2276593	.1419361	-.2230223	.0964473	-.004637	.1716713	0.978
amit traz	-.3044849	.2668247	-.340145	.1350799	.03566	.2988839	0.905
amit venl	.0625363	.2956076	-.1337461	.0798471	.1962824	.3062014	0.522
bupr esci	.0708213	.2064996	-.0104458	.1070143	.0812671	.2325033	0.727
bupr fluo	-.1290124	.1848312	-.0962969	.102387	-.0327156	.2112596	0.877
bupr paro	.0285873	.4056227	.0184147	.092918	.0101726	.4161292	0.980
bupr sert	.2231338	1.1074	-.0593396	.1026126	.2824734	1.112144	0.800
bupr traz	-.7463907	.419044	-.0997854	.1406402	-.6466053	.4420153	0.144
bupr venl	-.4392145	.2859877	.1044274	.0982077	-.5436419	.3023801	0.072
cita clom	1.298032	.4368018	.0872732	.1266529	1.210759	.4547932	0.008
cita esci	.3931275	.1016305	-.0418589	.0957442	.4349864	.1395716	0.002
cita fluo	-.0582283	.1956727	.0690615	.076364	-.1272898	.2100509	0.545
cita fluv	-.5833954	.4738403	.2219321	.1225709	-.8053275	.4894367	0.100
cita mirt	-.0870115	.3040366	.1961548	.1009481	-.2831662	.3203572	0.377
cita paro	-.2543858	.3228698	.1980203	.0736482	-.452406	.3317471	0.173
cita rebo	-.4534165	.2751395	-.0722515	.1070669	-.381165	.2952373	0.197
cita sert	.0538369	.209193	.1076963	.0905164	-.0538594	.2278978	0.813
cita venl	-.7713011	.3545617	.2508198	.078555	-1.022121	.3632405	0.005
cita vila	.0571497	.1986198	.0863117	.1444772	-.0291621	.2457155	0.906
clom fluo	.3409582	.2460238	-.2479651	.1240904	.5889233	.2754941	0.033

clom fluv	-.436202	.5125198	.0271245	.1510976	-.4633264	.5343301	0.386
clom miln	-.4986637	.4232228	-.0131933	.1816694	-.4854703	.4605663	0.292
clom paro	-.031953	.1511654	.0201977	.1526492	-.0521506	.2148302	0.808
clom sert	.0908138	.2889452	-.1169353	.129823	.2077491	.316767	0.512
clom venl	.2876811	.503945	.0071756	.1190794	.2805054	.5178228	0.588
desv dulo	.3329104	.2732742	.2125814	.1130657	.1203291	.2967423	0.685
dulo esci	.0173482	.1597234	-.1137475	.0790624	.1310957	.1782316	0.462
dulo fluo	-.4234202	.3206089	-.1884475	.0668632	-.2349727	.3280352	0.474
dulo paro	-.0262073	.1111627	-.0981952	.0745963	.0719879	.1343478	0.592
dulo venl	.1274991	.1929787	-.072681	.0730183	.2001801	.2063243	0.332
dulo vort	-.2546992	.1130444	-.1041826	.1087407	-.1505166	.1574449	0.339
esci fluo	.0060652	.1769062	-.127646	.0673566	.1337111	.1893701	0.480
esci paro	-.0445955	.156975	.0227883	.0676492	-.0673838	.1710477	0.694
esci sert	.1973457	.254803	-.0903131	.0813813	.2876588	.2674512	0.282
esci venl	.0029825	.2270103	.0440628	.0714351	-.0410803	.2379822	0.863
fluo fluv	.1684262	.2790038	.1103187	.1167112	.0581075	.3024221	0.848
fluo miln	.2608335	.3406348	-.007133	.1577959	.2679666	.3754087	0.475
fluo mirt	.1228264	.1642476	.1135786	.0935013	.0092477	.1889685	0.961
fluo nefa	-.0731966	.3840808	.2121007	.1525065	-.2852974	.4132447	0.490
fluo paro	.0729963	.099284	.1394884	.0574559	-.066492	.1145319	0.562
fluo rebo	-.1733843	.1694422	-.1748456	.1028733	.0014613	.1980348	0.994
fluo sert	.1517345	.1482942	.0194696	.0759256	.1322649	.1665881	0.427
fluo traz	.0595788	.2979704	-.0820012	.1227041	.14158	.3222506	0.660
fluo venl	.1498138	.0915963	.1518161	.0692024	-.0020023	.1147595	0.986
fluv mirt	-.1766771	.2293632	.0597968	.1382579	-.2364739	.2678081	0.377
fluv paro	.1074053	.3896344	-.0048645	.1126463	.1122698	.4056007	0.782
fluv sert	-.3743977	.341117	-.0330137	.1226846	-.341384	.3625052	0.346
fluv venl	.6321052	.4547529	-.0068444	.1154137	.6389496	.4691699	0.173
miln paro	.036084	.175547	.1609197	.2273029	-.1248358	.287178	0.664
mirt paro	-.1255477	.1670261	.0479214	.0927999	-.173469	.1910706	0.364
mirt sert	-.0098146	.2717728	-.076703	.0989077	.0668883	.2892113	0.817
mirt traz	-.3653329	.6422027	-.1691677	.1339661	-.1961651	.655639	0.765
mirt venl	-.0296934	.243448	.0446196	.0926351	-.074313	.2604758	0.775
nefa paro	.2716517	.3627116	-.1081791	.1535092	.3798308	.3938298	0.335
nefa sert	-.0655973	.4056699	-.1355531	.1583121	.0699558	.4354662	0.872
paro rebo	-.2142652	.1444555	-.3443312	.10864	.1300659	.1807835	0.472
paro sert	-.0270348	.2115334	-.0816764	.0724652	.0546416	.2236256	0.807
paro traz	-.2891909	.2787074	-.1636174	.123715	-.1255736	.3049765	0.681
paro venl	.3891754	.1904294	-.0092893	.061129	.3984647	.2002183	0.047
rebo venl	-.0465201	.394682	.3475787	.0960614	-.3940988	.406204	0.332
sert traz	.4587335	.3998632	-.1658906	.127368	.6246241	.4196584	0.137
sert venl	.2199969	.209497	.0877627	.0785648	.1322342	.2237383	0.555
traz venl	.9022304	.510545	.1760041	.1203618	.7262263	.5230417	0.165
venl vort	.0995657	.1878571	-.1800679	.0886338	.2796335	.2080229	0.179

7.4.4.3 Design-by-treatment test

chi2(133) = 120.68

Prob > chi2 = 0.4655

7.4.5 Dropouts due to adverse events

7.4.5.1 Loop-specific approach

Loops With Evidence of Statistical Inconsistency:

Loop	IF	seIF	z_value	p_value	CI_95	Loop_Heterog_tau2
mirt-sert-traz	3.232	1.092	2.960	0.003	(1.09,5.37)	0.000
paro-sert-traz	2.189	1.058	2.069	0.039	(0.12,4.26)	0.000
Plac-mirt-sert	1.997	0.690	2.892	0.004	(0.64,3.35)	0.126
agom-dulo-venl	1.909	0.587	3.252	0.001	(0.76,3.06)	0.000
agom-dulo-esci	1.904	0.792	2.405	0.016	(0.35,3.46)	0.087
cita-fluo-venl	1.876	0.933	2.011	0.044	(0.05,3.70)	0.020

amit-mirt-sert	1.673	0.602	2.780	0.005	(0.49,2.85)	0.000
dulo-venl-vort	1.577	0.427	3.694	0.000	(0.74,2.41)	0.017
esci-paro-venl	1.297	0.579	2.240	0.025	(0.16,2.43)	0.000
agom-paro-venl	1.278	0.489	2.612	0.009	(0.32,2.24)	0.000
amit-fluo-sert	1.270	0.439	2.896	0.004	(0.41,2.13)	0.029
Plac-fluv-mirt	1.199	0.428	2.803	0.005	(0.36,2.04)	0.000
Plac-dulo-venl	1.025	0.338	3.032	0.002	(0.36,1.69)	0.037
amit-fluo-paro	0.795	0.340	2.338	0.019	(0.13,1.46)	0.000
Plac-dulo-esci	0.675	0.300	2.252	0.024	(0.09,1.26)	0.000
Plac-paro-venl	0.583	0.297	1.962	0.050	(0.00,1.17)	0.009

Loops Without Evidence of Statistical Inconsistency:

Loop	IF	seIF	z_value	p_value	CI_95	Loop_Heterog_tau2
Plac-cita-clom	3.244	1.849	1.755	0.079	(0.00,6.87)	0.000
cita-clom-fluv	2.428	1.998	1.216	0.224	(0.00,6.34)	0.000
clom-fluv-mirt-venl	2.094	1.436	1.458	0.145	(0.00,4.91)	0.000
clom-fluo-fluv	1.985	1.700	1.167	0.243	(0.00,5.32)	0.000
bupr-esci-sert-traz	1.957	1.789	1.094	0.274	(0.00,5.46)	0.658
Plac-clom-sert	1.904	1.254	1.517	0.129	(0.00,4.36)	0.141
Plac-clom-fluo	1.879	1.209	1.555	0.120	(0.00,4.25)	0.058
amit-clom-fluv-venl	1.798	1.489	1.207	0.227	(0.00,4.72)	0.000
Plac-cita-venl	1.677	0.908	1.847	0.065	(0.00,3.46)	0.057
Plac-clom-venl	1.622	1.279	1.269	0.205	(0.00,4.13)	0.104
fluo-sert-traz	1.604	0.990	1.620	0.105	(0.00,3.54)	0.000
cita-fluo-sert	1.584	0.977	1.622	0.105	(0.00,3.50)	0.000
cita-mirt-sert	1.570	1.196	1.313	0.189	(0.00,3.91)	0.000
cita-clom-fluo	1.544	1.614	0.957	0.339	(0.00,4.71)	0.000
agom-dulo-fluo	1.533	1.172	1.307	0.191	(0.00,3.83)	0.000
Plac-sert-traz	1.501	1.002	1.497	0.134	(0.00,3.47)	0.132
cita-paro-venl	1.472	0.994	1.481	0.139	(0.00,3.42)	0.000
bupr-paro-venl	1.417	0.756	1.875	0.061	(0.00,2.90)	0.000
Plac-clom-paro	1.375	1.075	1.279	0.201	(0.00,3.48)	0.000
amit-sert-traz	1.295	0.961	1.347	0.178	(0.00,3.18)	0.006
Plac-amit-clom-miln	1.263	1.688	0.748	0.454	(0.00,4.57)	0.267
amit-fluv-sert	1.252	0.948	1.320	0.187	(0.00,3.11)	0.073
amit-bupr-fluo	1.232	1.410	0.873	0.382	(0.00,4.00)	0.223
mirt-paro-sert	1.221	0.772	1.581	0.114	(0.00,2.73)	0.000
fluv-mirt-paro	1.202	0.843	1.427	0.154	(0.00,2.85)	0.165
cita-esci-venl	1.170	0.971	1.204	0.228	(0.00,3.07)	0.043
Plac-dulo-fluo	1.154	1.121	1.030	0.303	(0.00,3.35)	0.038
agom-esci-fluo	1.142	0.589	1.940	0.052	(0.00,2.30)	0.000
Plac-cita-mirt	1.130	0.708	1.595	0.111	(0.00,2.52)	0.025
cita-esci-sert	1.130	1.064	1.062	0.288	(0.00,3.21)	0.055
cita-fluo-fluv	1.100	1.128	0.976	0.329	(0.00,3.31)	0.000
amit-bupr-venl	1.095	1.323	0.828	0.408	(0.00,3.69)	0.000
cita-clom-paro	1.054	1.660	0.635	0.525	(0.00,4.31)	0.041
mirt-sert-venl	1.049	0.696	1.507	0.132	(0.00,2.41)	0.000
clom-fluv-paro	1.038	1.687	0.616	0.538	(0.00,4.34)	0.231
cita-fluv-sert	1.024	1.238	0.827	0.408	(0.00,3.45)	0.000
cita-esci-paro	1.020	0.667	1.529	0.126	(0.00,2.33)	0.000
clom-fluo-miln	1.001	0.813	1.231	0.218	(0.00,2.59)	0.000
amit-fluv-paro	0.991	0.640	1.548	0.122	(0.00,2.25)	0.000
clom-fluv-sert	0.986	2.541	0.388	0.698	(0.00,5.97)	1.291
dulo-esci-paro	0.960	0.504	1.904	0.057	(0.00,1.95)	0.000
Plac-nefa-sert	0.950	0.600	1.581	0.114	(0.00,2.13)	0.074
dulo-fluo-paro	0.943	1.115	0.846	0.398	(0.00,3.13)	0.000
amit-fluo-miln	0.935	0.960	0.975	0.330	(0.00,2.82)	0.070
Plac-paro-sert	0.933	0.561	1.663	0.096	(0.00,2.03)	0.003
amit-bupr-traz	0.885	1.323	0.669	0.503	(0.00,3.48)	0.000
Plac-amit-bupr	0.884	1.259	0.702	0.483	(0.00,3.35)	0.132
amit-fluo-traz	0.862	0.777	1.109	0.267	(0.00,2.39)	0.101
esci-fluo-paro	0.837	0.503	1.663	0.096	(0.00,1.82)	0.000
cita-fluo-mirt	0.830	0.743	1.116	0.264	(0.00,2.29)	0.000
Plac-cita-paro	0.824	0.526	1.567	0.117	(0.00,1.86)	0.000
fluo-mirt-sert	0.815	0.620	1.315	0.189	(0.00,2.03)	0.000
fluo-mirt-traz	0.812	0.704	1.153	0.249	(0.00,2.19)	0.000
cita-fluo-paro	0.806	0.591	1.363	0.173	(0.00,1.96)	0.000
dulo-esci-fluo	0.799	1.186	0.674	0.500	(0.00,3.12)	0.037
amit-fluv-miln	0.794	0.994	0.799	0.425	(0.00,2.74)	0.000
cita-paro-sert	0.794	1.134	0.700	0.484	(0.00,3.02)	0.000
cita-mirt-venl	0.788	1.067	0.738	0.460	(0.00,2.88)	0.000
fluo-traz-venl	0.773	0.671	1.152	0.249	(0.00,2.09)	0.030
clom-fluo-sert	0.762	0.684	1.115	0.265	(0.00,2.10)	0.000

dulo-esci-venl	0.745	0.671	1.110	0.267	(0.00,2.06)	0.114
Plac-fluo-sert	0.736	0.377	1.951	0.051	(0.00,1.48)	0.072
Plac-sert-venl	0.724	0.515	1.406	0.160	(0.00,1.73)	0.095
cita-clom-sert	0.722	1.788	0.404	0.686	(0.00,4.23)	0.000
amit-cita-fluv-venl	0.690	1.069	0.645	0.519	(0.00,2.78)	0.000
agom-esci-venl	0.690	0.694	0.994	0.320	(0.00,2.05)	0.000
bupr-traz-venl	0.681	0.714	0.954	0.340	(0.00,2.08)	0.000
Plac-agom-esci	0.680	0.526	1.292	0.196	(0.00,1.71)	0.000
fluo-fluv-paro	0.672	1.193	0.563	0.573	(0.00,3.01)	0.044
agom-esci-paro	0.671	0.659	1.019	0.308	(0.00,1.96)	0.000
Plac-cita-sert	0.671	0.960	0.699	0.485	(0.00,2.55)	0.053
Plac-amit-fluv	0.671	0.446	1.505	0.132	(0.00,1.54)	0.062
paro-rebo-venl	0.666	1.011	0.659	0.510	(0.00,2.65)	0.336
fluo-miln-paro	0.665	0.425	1.566	0.117	(0.00,1.50)	0.000
Plac-esci-sert	0.649	0.520	1.248	0.212	(0.00,1.67)	0.046
amit-sert-venl	0.648	0.652	0.994	0.320	(0.00,1.93)	0.000
esci-paro-sert	0.644	0.801	0.805	0.421	(0.00,2.21)	0.000
Plac-desv-dulo	0.637	0.396	1.609	0.108	(0.00,1.41)	0.000
Plac-fluv-sert	0.636	0.867	0.734	0.463	(0.00,2.34)	0.068
amit-fluo-venl	0.634	0.603	1.050	0.294	(0.00,1.82)	0.050
cita-esci-fluo	0.623	0.488	1.276	0.202	(0.00,1.58)	0.000
Plac-fluo-fluv	0.605	1.053	0.574	0.566	(0.00,2.67)	0.019
amit-clom-miln-sert	0.595	1.119	0.531	0.595	(0.00,2.79)	0.052
clom-fluv-miln	0.588	1.490	0.395	0.693	(0.00,3.51)	0.000
Plac-amit-fluo	0.578	0.374	1.544	0.123	(0.00,1.31)	0.131
Plac-bupr-paro	0.574	0.627	0.915	0.360	(0.00,1.80)	0.000
fluo-paro-traz	0.570	0.603	0.944	0.345	(0.00,1.75)	0.000
Plac-agom-dulo	0.567	0.429	1.323	0.186	(0.00,1.41)	0.000
Plac-fluo-traz	0.563	0.602	0.935	0.350	(0.00,1.74)	0.079
bupr-paro-traz	0.553	0.828	0.668	0.504	(0.00,2.18)	0.000
amit-fluo-fluv	0.547	1.125	0.486	0.627	(0.00,2.75)	0.026
bupr-fluo-paro	0.540	0.720	0.750	0.453	(0.00,1.95)	0.009
Plac-fluo-rebo	0.518	0.344	1.508	0.132	(0.00,1.19)	0.050
clom-sert-venl	0.514	0.790	0.651	0.515	(0.00,2.06)	0.000
amit-miln-paro	0.511	0.809	0.632	0.528	(0.00,2.10)	0.000
Plac-cita-vila	0.493	0.452	1.092	0.275	(0.00,1.38)	0.000
agom-fluo-venl	0.492	0.441	1.116	0.265	(0.00,1.36)	0.000
fluo-fluv-sert	0.482	1.669	0.289	0.773	(0.00,3.75)	0.194
amit-paro-sert	0.480	0.575	0.835	0.404	(0.00,1.61)	0.000
Plac-clom-fluv	0.479	1.661	0.288	0.773	(0.00,3.73)	0.000
amit-traz-venl	0.471	0.759	0.621	0.535	(0.00,1.96)	0.000
bupr-fluo-venl	0.466	0.629	0.742	0.458	(0.00,1.70)	0.048
cita-fluv-paro	0.452	0.800	0.565	0.572	(0.00,2.02)	0.000
bupr-fluo-traz	0.446	1.059	0.421	0.674	(0.00,2.52)	0.301
cita-mirt-paro	0.445	0.820	0.543	0.587	(0.00,2.05)	0.000
Plac-nefa-paro	0.445	0.448	0.993	0.321	(0.00,1.32)	0.000
Plac-fluo-mirt	0.439	0.362	1.214	0.225	(0.00,1.15)	0.073
Plac-agom-paro	0.433	0.274	1.580	0.114	(0.00,0.97)	0.000
fluo-mirt-paro	0.421	0.358	1.176	0.240	(0.00,1.12)	0.000
fluo-paro-venl	0.420	0.308	1.363	0.173	(0.00,1.02)	0.000
clom-paro-sert	0.420	0.820	0.513	0.608	(0.00,2.03)	0.077
amit-clom-miln-venl	0.416	1.184	0.351	0.725	(0.00,2.74)	0.000
Plac-amit-mirt	0.415	0.431	0.963	0.335	(0.00,1.26)	0.091
amit-paro-traz	0.411	0.591	0.695	0.487	(0.00,1.57)	0.000
dulo-paro-venl	0.408	0.401	1.015	0.310	(0.00,1.19)	0.000
amit-fluo-mirt	0.402	0.455	0.885	0.376	(0.00,1.29)	0.000
nefa-paro-sert	0.401	0.791	0.507	0.612	(0.00,1.95)	0.000
fluo-fluv-miln	0.395	1.212	0.326	0.744	(0.00,2.77)	0.000
cita-fluv-mirt	0.395	0.794	0.497	0.619	(0.00,1.95)	0.000
fluo-nefa-paro	0.390	0.723	0.540	0.589	(0.00,1.81)	0.000
fluv-miln-paro	0.387	1.059	0.366	0.715	(0.00,2.46)	0.133
Plac-esci-paro	0.384	0.433	0.887	0.375	(0.00,1.23)	0.000
agom-fluo-paro	0.366	0.311	1.175	0.240	(0.00,0.98)	0.000
Plac-bupr-esci	0.358	0.470	0.763	0.445	(0.00,1.28)	0.000
Plac-esci-venl	0.357	0.419	0.850	0.395	(0.00,1.18)	0.047
clom-paro-venl	0.356	0.618	0.576	0.565	(0.00,1.57)	0.000
Plac-amit-paro	0.343	0.246	1.398	0.162	(0.00,0.82)	0.012
Plac-venl-vort	0.342	0.366	0.935	0.350	(0.00,1.06)	0.048
Plac-cita-fluv	0.337	0.446	0.756	0.449	(0.00,1.21)	0.000
Plac-esci-fluo	0.328	0.323	1.014	0.311	(0.00,0.96)	0.000
Plac-agom-venl	0.323	0.426	0.757	0.449	(0.00,1.16)	0.005
clom-fluo-paro	0.318	0.536	0.592	0.554	(0.00,1.37)	0.000
amit-fluv-mirt	0.273	0.537	0.509	0.611	(0.00,1.32)	0.000
bupr-esci-fluo	0.271	0.830	0.327	0.744	(0.00,1.90)	0.227
cita-sert-venl	0.267	1.286	0.208	0.835	(0.00,2.79)	0.000
Plac-amit-traz	0.260	0.703	0.370	0.711	(0.00,1.64)	0.214
amit-mirt-traz	0.260	0.703	0.371	0.711	(0.00,1.64)	0.000

clom-fluo-venl	0.256	0.745	0.344	0.731	(0.00,1.72)	0.004
Plac-bupr-traz	0.252	0.559	0.450	0.653	(0.00,1.35)	0.010
esci-sert-venl	0.251	0.727	0.346	0.729	(0.00,1.68)	0.030
Plac-bupr-venl	0.247	0.517	0.478	0.632	(0.00,1.26)	0.061
amit-paro-venl	0.243	0.558	0.435	0.663	(0.00,1.34)	0.000
fluo-mirt-venl	0.239	0.350	0.684	0.494	(0.00,0.92)	0.000
Plac-dulo-vort	0.239	0.275	0.870	0.385	(0.00,0.78)	0.032
fluo-rebo-venl	0.239	0.685	0.349	0.727	(0.00,1.58)	0.035
mirt-paro-venl	0.238	0.408	0.584	0.559	(0.00,1.04)	0.000
mirt-traz-venl	0.234	0.667	0.352	0.725	(0.00,1.54)	0.000
Plac-paro-traz	0.232	0.472	0.490	0.624	(0.00,1.16)	0.000
Plac-mirt-venl	0.230	0.396	0.581	0.561	(0.00,1.00)	0.088
Plac-fluo-nefa	0.229	0.663	0.345	0.730	(0.00,1.53)	0.044
agom-dulo-paro	0.223	0.471	0.474	0.635	(0.00,1.15)	0.000
Plac-bupr-fluo	0.219	0.467	0.469	0.639	(0.00,1.13)	0.072
Plac-cita-esci	0.217	0.347	0.624	0.533	(0.00,0.90)	0.000
Plac-fluo-paro	0.215	0.187	1.149	0.251	(0.00,0.58)	0.000
Plac-paro-rebo	0.209	0.321	0.650	0.516	(0.00,0.84)	0.068
esci-fluo-sert	0.208	0.585	0.355	0.722	(0.00,1.36)	0.000
Plac-rebo-venl	0.202	0.727	0.277	0.782	(0.00,1.63)	0.129
Plac-mirt-traz	0.201	0.683	0.294	0.769	(0.00,1.54)	0.125
Plac-cita-fluo	0.198	0.432	0.457	0.648	(0.00,1.04)	0.037
Plac-agom-fluo	0.189	0.278	0.680	0.496	(0.00,0.73)	0.000
paro-traz-venl	0.183	0.604	0.303	0.762	(0.00,1.37)	0.000
mirt-paro-traz	0.179	0.651	0.275	0.784	(0.00,1.45)	0.000
Plac-traz-venl	0.176	0.638	0.275	0.783	(0.00,1.43)	0.114
fluv-mirt-sert	0.145	1.870	0.078	0.938	(0.00,3.81)	0.623
Plac-amit-sert	0.142	0.345	0.413	0.680	(0.00,0.82)	0.091
fluo-fluv-mirt	0.124	1.102	0.113	0.910	(0.00,2.28)	0.000
dulo-fluo-venl	0.118	1.135	0.104	0.917	(0.00,2.34)	0.006
Plac-mirt-paro	0.117	0.313	0.375	0.708	(0.00,0.73)	0.000
amit-bupr-paro	0.079	1.324	0.060	0.952	(0.00,2.67)	0.000
cita-clom-venl	0.060	1.802	0.033	0.974	(0.00,3.59)	0.000
esci-fluo-venl	0.059	0.476	0.125	0.901	(0.00,0.99)	0.018
amit-bupr-esci-sert	0.055	1.492	0.037	0.970	(0.00,2.98)	0.130
Plac-dulo-paro	0.045	0.241	0.188	0.851	(0.00,0.52)	0.000
fluo-paro-rebo	0.045	0.454	0.100	0.921	(0.00,0.94)	0.130
Plac-fluo-venl	0.033	0.247	0.135	0.893	(0.00,0.52)	0.078
amit-mirt-paro	0.028	0.398	0.071	0.944	(0.00,0.81)	0.000
Plac-amit-venl	0.027	0.602	0.045	0.964	(0.00,1.21)	0.158
Plac-fluv-paro	0.024	0.574	0.042	0.967	(0.00,1.15)	0.000
amit-mirt-venl	0.024	0.592	0.040	0.968	(0.00,1.18)	0.000
clom-miln-paro	0.018	0.615	0.029	0.977	(0.00,1.22)	0.000
bupr-esci-paro	0.017	1.202	0.014	0.989	(0.00,2.37)	0.364
fluo-paro-sert	0.016	0.606	0.026	0.979	(0.00,1.20)	0.000
bupr-esci-venl	0.015	1.355	0.011	0.991	(0.00,2.67)	0.654
fluo-sert-venl	0.006	0.511	0.011	0.991	(0.00,1.01)	0.000
fluo-nefa-sert	0.005	0.790	0.006	0.995	(0.00,1.55)	0.000

7.4.5.2 Side-splitting

```
Side    Direct                 Indirect               Difference
        Coef.      Std. Err.   Coef.      Std. Err.   Coef.      Std. Err.  P>|z|
Plac agom .0368342  .1771775   .2384122   .1646743   -.201578   .2422192  0.405
Plac amit .9994015  .1673345   .9948111   .1255082   .0045904   .2118561  0.983
Plac bupr .6739476  .1851941   .8414251   .2652018  -.1674776   .3268148  0.608
Plac cita .7878851  .2203316   .3777655   .2003561   .4101196   .3078968  0.183
Plac clom -.117783  1.083012   1.371263   .1805781  -1.489046   1.097963  0.175
Plac dulo .6789495  .1268085   1.111076   .1664644  -.4321269   .2152391  0.045
Plac esci .5580632  .1458396     .35059    .169294   .2074732   .2285179  0.364
Plac fluo .5315609  .1137867   .5142279   .1023526    .017333   .1550585  0.911
Plac fluv 1.135195  .2107863   .7251524   .1940882   .4100428   .2863476  0.152
Plac mirt .4381218  .1953816   .8470265   .1474625  -.4089047   .2490433  0.101
Plac nefa  .521457  .2310991   1.019478   .2963012  -.4980207   .3757358  0.185
Plac paro .7043564   .093708   .7566027   .1020084  -.0522462   .1404567  0.710
Plac rebo  .742254  .1801749   1.268105   .2396442  -.5258515   .3083804  0.088
Plac sert .9433967  .1637246   .3413535   .1514809   .6020432   .2265209  0.008
Plac traz 1.251293  .2990593    .907548   .2174225   .3437446   .3780128  0.363
Plac venl .9973621  .1292609   .9760276   .1116891   .0213345   .1731546  0.902
Plac vort .3991385  .1493948   .5495124   .2864206  -.1503739   .3346712  0.653
agom dulo .0716558   .444745   .7724352   .1558532  -.7007793   .4712625  0.137
agom esci 1.183167  .5117796   .2388736   .1607456   .9442938   .5364406  0.078
```

agom	fluo	.3361908	.2437035	.3933197	.1522884	-.0571289	.2876512	0.843
agom	paro	.2391442	.2292214	.7309795	.1503746	-.4918353	.273475	0.072
agom	venl	1.300315	.4093664	.7821962	.1449299	.5181192	.4342336	0.233
amit	bupr	.483416	1.194528	-.2833206	.1770446	.7667366	1.207577	0.525
amit	fluo	-1.068725	.282084	-.3690595	.1186784	-.6996653	.3059999	0.022
amit	fluv	-.5674969	.3451656	.0486081	.1794009	-.616105	.3890209	0.113
amit	miln	-.5868074	.7995766	-.5642671	.2204212	-.0225404	.8294022	0.978
amit	mirt	-.3316328	.3015131	-.2907855	.1537206	-.0408473	.3381541	0.904
amit	paro	-.0189716	.1770262	-.3923627	.1261221	.3733912	.2167599	0.085
amit	sert	-.1673621	.1908589	-.5316114	.1649528	.3642493	.2524355	0.149
amit	traz	-.0861581	.4721936	.0542414	.2034784	-.1403995	.5141011	0.785
amit	venl	.005513	.502289	-.0123089	.1227268	.0178219	.5170664	0.973
bupr	esci	-.4031209	.4387582	-.2319817	.1949775	-.1711391	.4807404	0.722
bupr	fluo	-.298959	.3965199	-.1891581	.1765325	-.1098009	.4351711	0.801
bupr	paro	-.5487822	.6414033	.0353194	.1651753	-.5841016	.6623301	0.378
bupr	traz	.3263267	.4971493	.2930618	.2374161	.0332649	.55093	0.952
bupr	venl	.6167635	.4566186	.2020895	.1758405	.414674	.489306	0.397
cita	clom	2.269275	1.520907	.7334024	.2250649	1.535872	1.53747	0.318
cita	esci	-.1014954	.2597697	-.0920975	.204482	-.0093979	.3303741	0.977
cita	fluo	-.4884944	.3685151	.0487214	.1672701	-.5372158	.4047845	0.184
cita	fluv	.5884544	.4234505	.289647	.2105816	.2988074	.4729217	0.527
cita	mirt	.6931453	.6674609	.0906886	.1821399	.6024567	.6918663	0.384
cita	paro	.6260824	.5309617	.1219115	.1591277	.5041709	.5545114	0.363
cita	sert	.8541692	.9063854	.024764	.1785865	.8294051	.9235949	0.369
cita	venl	1.854524	.8678957	.3701911	.1619565	1.484333	.8828773	0.093
cita	vila	.4294403	.4015273	.0851854	.3100165	.3442548	.5093124	0.499
clom	fluo	-1.195078	.5199162	-.7552473	.1946524	-.4398303	.5551573	0.428
clom	fluv	.747194	1.289608	-.4527268	.224205	1.199921	1.308952	0.359
clom	miln	-.650583	.5981236	-.9517271	.2702543	.3011441	.6563453	0.646
clom	paro	-.5826601	.2344708	-.6274839	.2581198	.0448238	.348652	0.898
clom	sert	-.8180359	.4392723	-.6842149	.2186526	-.133821	.490727	0.785
clom	venl	-.4744547	.5879405	-.3314267	.198156	-.1430281	.6204352	0.818
desv	dulo	.808185	.4091193	.2462568	.2268308	.5619282	.4679017	0.230
dulo	esci	-.7638434	.2780351	-.2515276	.1540117	-.5123159	.3179542	0.107
dulo	fluo	-1.012427	.9848392	-.3081431	.1186403	-.7042837	.9933554	0.478
dulo	paro	.0082135	.2191539	-.1547067	.1287655	.1629202	.2549911	0.523
dulo	venl	-.6927157	.2944473	.2946754	.125998	-.9873911	.3202784	0.002
dulo	vort	-.0756807	.1842195	-.8069424	.2033783	.7312617	.2754692	0.008
esci	fluo	.3071848	.3123055	.0075518	.1317678	.299633	.3388543	0.377
esci	paro	-.2521645	.431949	.3033944	.1254927	-.5555589	.4504239	0.217
esci	sert	-.2017458	.4653987	.1913036	.1559822	-.3930495	.4912991	0.424
esci	venl	.8300667	.3773687	.4757893	.1350119	.3542774	.4007747	0.377
fluo	fluv	-.0416727	1.047248	.401427	.1562435	-.4430997	1.058839	0.676
fluo	miln	-.4567047	.3878018	.0423891	.2340129	-.4990938	.452928	0.270
fluo	mirt	.2968483	.2652595	.1417448	.1392922	.1551035	.299853	0.605
fluo	nefa	.1851867	.6161665	.188382	.2024422	-.0031953	.6485709	0.996
fluo	paro	.2421431	.1606138	.1928264	.0982577	.0493167	.187703	0.793
fluo	rebo	.5618311	.2677049	.3462549	.1804253	.2155762	.3257239	0.508
fluo	sert	-.3571537	.3020124	.1865023	.1326979	-.543656	.3294498	0.099
fluo	traz	.0812856	.4868728	.5729973	.1914961	-.4917117	.5233253	0.347
fluo	venl	.4369159	.1529482	.4787403	.1176804	-.0418244	.1932453	0.829
fluv	miln	-.823075	.5863681	-.4201645	.252222	-.4029105	.6382351	0.528
fluv	mirt	.5123113	.3788996	-.3957739	.1881258	.9080852	.4230644	0.032
fluv	paro	-.3826246	.564582	-.1700294	.1565494	-.2125952	.5860024	0.717
fluv	sert	-.8415077	.8107806	-.2671259	.176853	-.5743818	.8298501	0.489
miln	paro	.0943881	.2713632	.5114988	.2786245	-.4171108	.3889656	0.284
mirt	paro	.3548524	.2756223	-.0460458	.1350286	.4008982	.3068146	0.191
mirt	sert	-1.4976	.5570794	.0318482	.153778	-1.529448	.5779145	0.008
mirt	traz	.5106705	.5105894	.3000682	.2129035	.2106023	.5539976	0.704
mirt	venl	.3734287	.2780177	.2646563	.1449682	.1087723	.3135438	0.729
nefa	paro	-.3145918	.4380565	.0948154	.2097582	-.4094072	.4855787	0.399
nefa	sert	-.5389951	.501241	-.0002533	.223432	-.5387418	.5487845	0.326
paro	rebo	.3118461	.2325733	.142371	.1846674	.1694751	.2966311	0.568
paro	sert	-.6357388	.5746065	-.0848512	.1228977	-.5508876	.5865767	0.348
paro	traz	.3552921	.431924	.2894249	.1936733	.0658672	.4733207	0.889
paro	venl	-.233858	.2943236	.3131965	.1008622	-.5470545	.3111609	0.079
rebo	venl	.0761883	.625335	.0470955	.1618875	.0290928	.64595	0.964
sert	traz	-1.203963	.8694493	.4951407	.2016529	-1.699104	.8925278	0.057
sert	venl	.8204079	.4450608	.322584	.1351633	.497824	.4651352	0.284
traz	venl	-.410494	.4693935	.0219081	.1978134	-.432402	.5099875	0.397
venl	vort	-.9806834	.3097823	-.4368283	.1592761	-.5438551	.3479028	0.118

7.4.5.3 Design-by-treatment test

chi2(130) = 171.38

Prob > chi2 = 0.0008

7.5 Comparison-adjusted funnel plots (all drugs vs placebo)

The funnel plots pertain to all trials comparing at least one antidepressant versus placebo. In the case of multi-arm trials which compared, for example, placebo vs drug A vs drug B, we plotted placebo vs A as well as placebo vs B.

The comparison-adjusted funnel plots against placebo suggest that there might be small-study effects for “efficacy continuous” but not for the other outcomes. The asymmetry in the funnel plot of response is primarily driven by one study.

7.5.1 Response

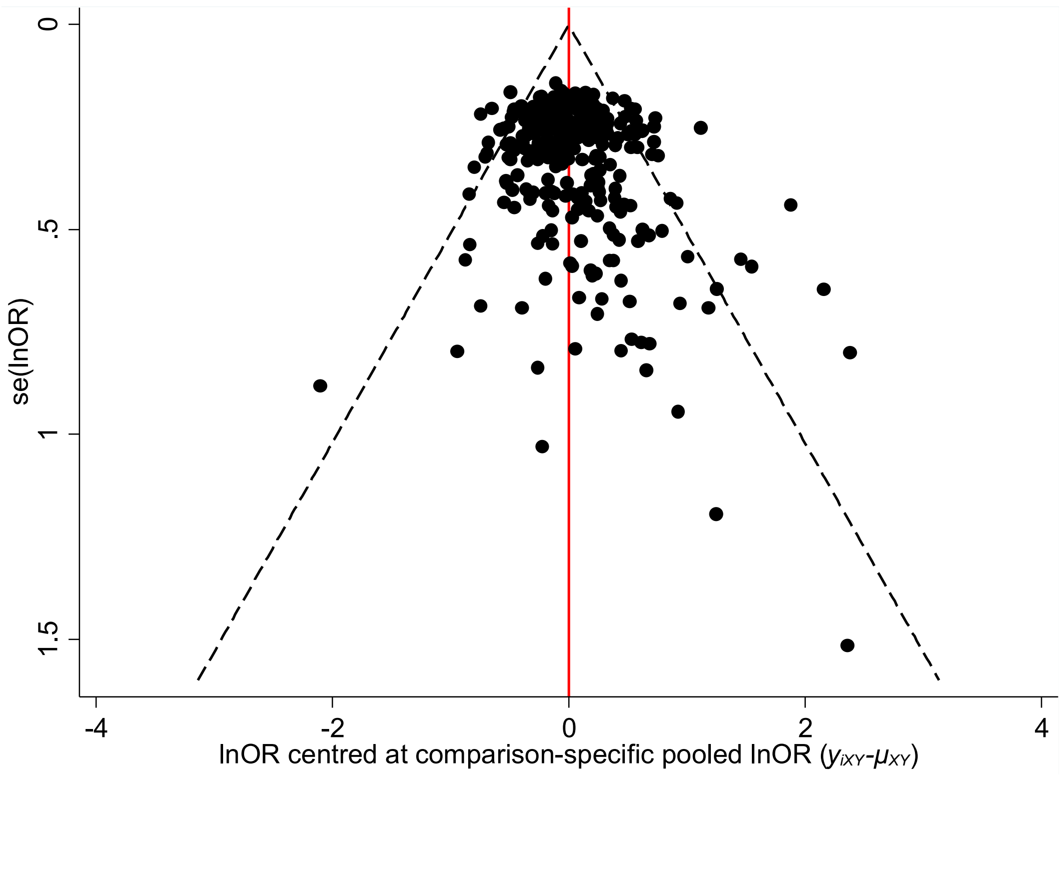

7.5.2 Dropouts for any reason

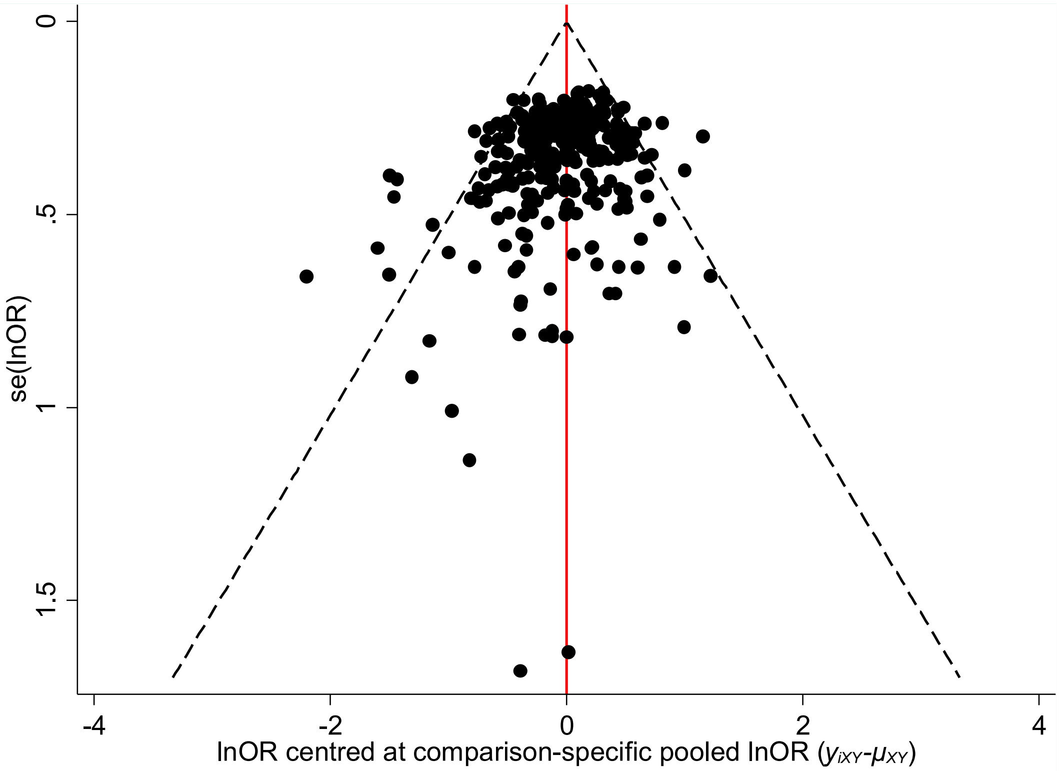

7.5.3 Efficacy continuous

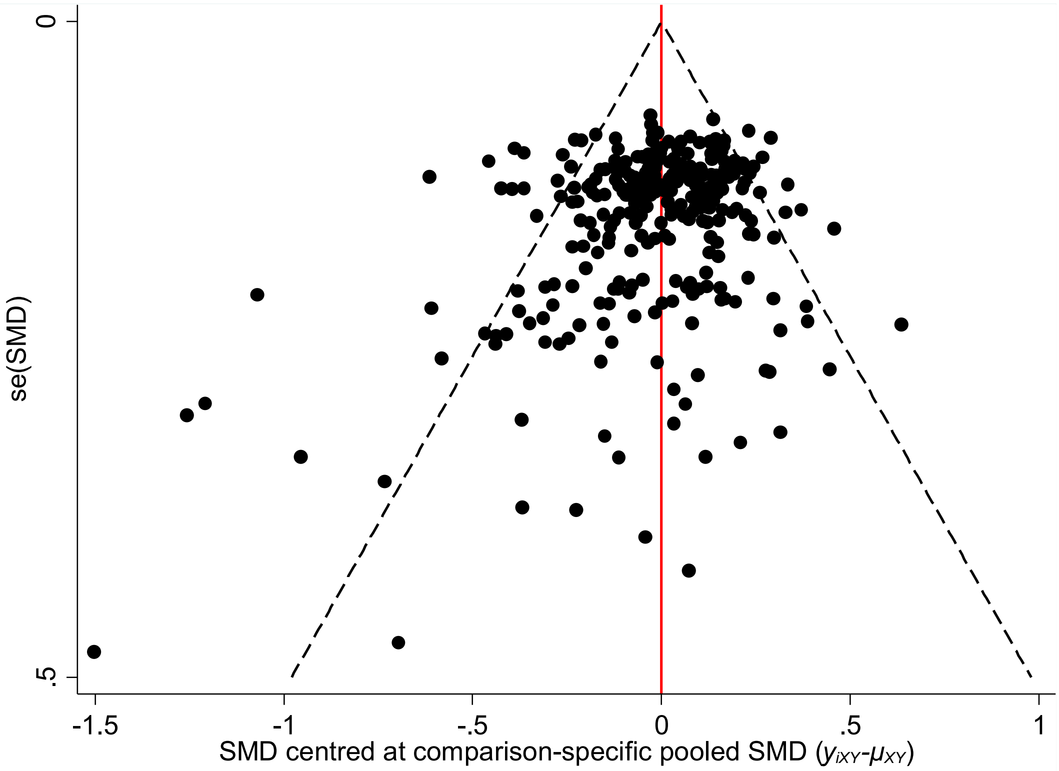

7.5.4 Remission

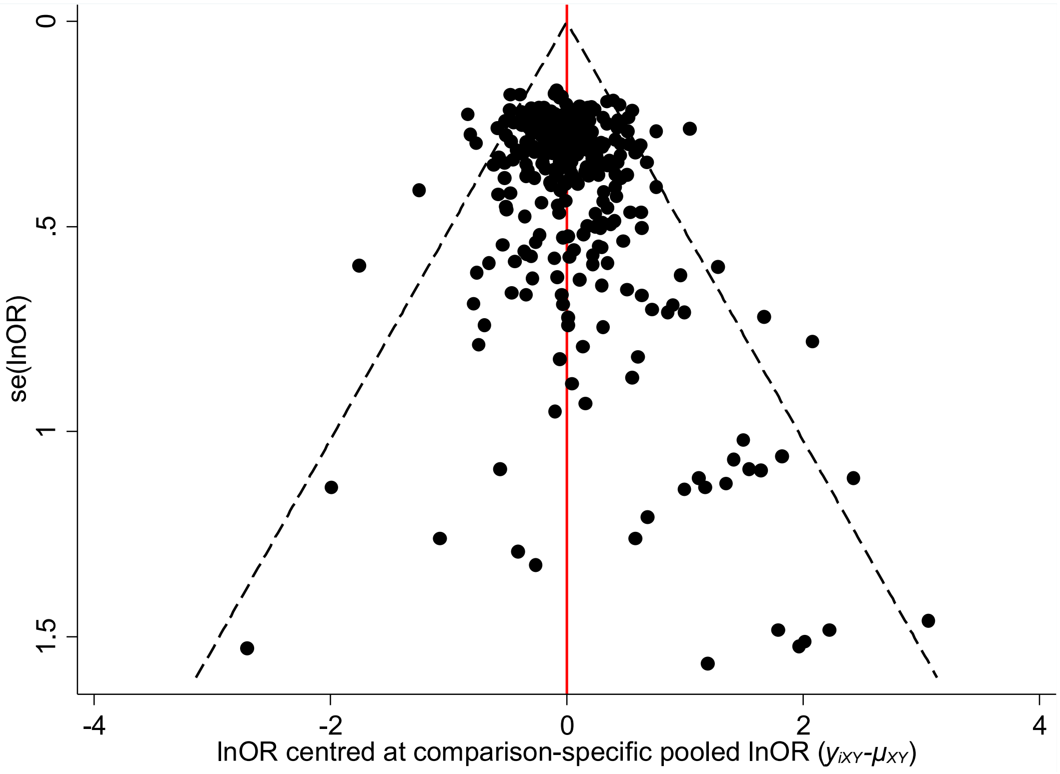

7.5.5 Dropouts due to adverse events

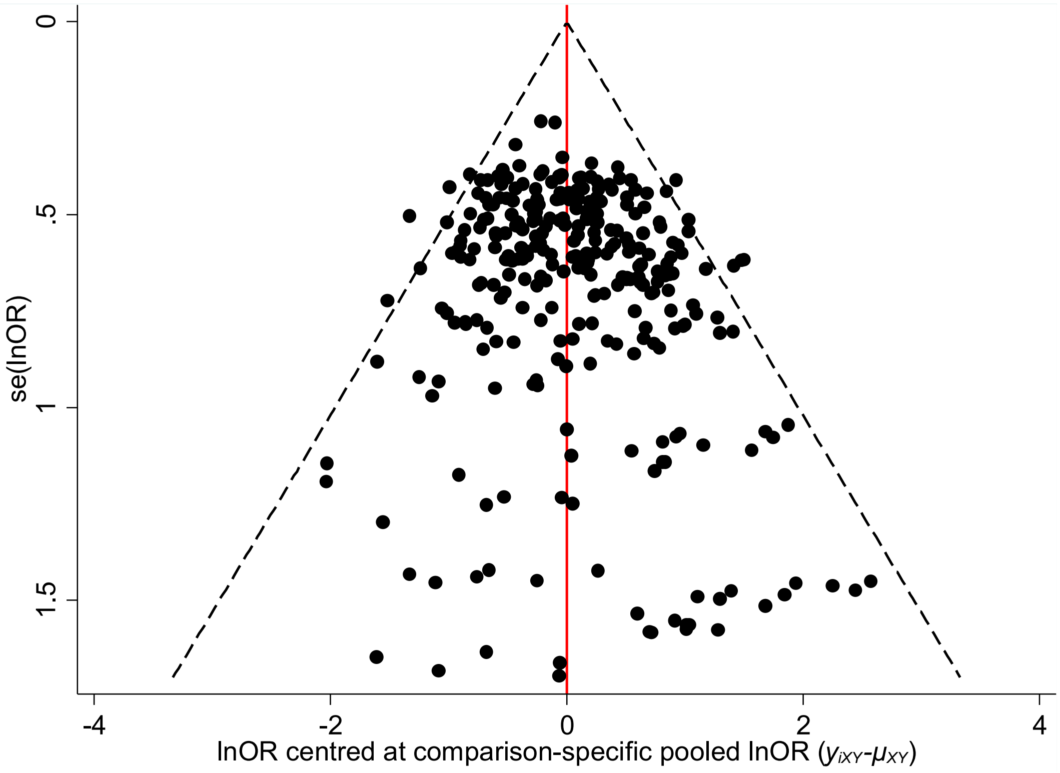

7.6 Meta-regressions and sensitivity analyses

To decide upon the impact of variables we consider the meta-regression results and subgroup analysis results and we judged

(a) whether heterogeneity is explained compared to the unadjusted analysis as reflected on the relative % change in the variance presented in section 7.6.2

(b) the changes in the estimated median ORs of active versus placebo

(c) the magnitude and significance of the regression coefficients when estimated/relevant (section 7.6.2).

Preplanned analyses including studies at low risk of bias only were not performed because very few studies would have been included compared to the total sample (39 studies).

7.6.1 Statistical details for network meta-analysis and meta-regression models

All models presented below (apart from the model accounting for the effect of novelty) pertain to meta-regressions with consistent coefficients. The basic coefficients (for each drug vs placebo) were assumed independent for comparisons with sufficient data and excangeable for comparisons with few data for which coefficients were not estimable. Specifically, we first ran all models assuming all coefficients bwing independent. Subsequently, we ran again the meta-regression models and we assumed exchangeable those coefficients ($\log(\beta)$) with lower or upper credible interval value exceeding the value of -20 or 20 respectively. For the novelty effect model we used exchangeable coefficients for the effect of novel/experimental versus older/control intervention.

For every study $\mathrm{i} = 1, \dots, \mathrm{N}$ we denote with $r_{i,k}$ the number of events in arm $\mathrm{k} = 1, \dots, K$ and $n_{i,k}$ is the sample size in arm k.

<u>Binomial Likelihood:</u> $\mathrm{r_{i,k}} \sim \mathrm{Binom}(\pi_{\mathrm{i,k}}, \mathrm{n_{i,k}})$

<u>Parameterization:</u>

$$\mathrm{logit}(\pi_{\mathrm{i,1}}) = u_i$$

$$\mathrm{logit}(\pi_{\mathrm{i,k}}) = \mathrm{u_i} + \theta^*_{i,k} \qquad \text{for } k \geq 2,$$

<u>Random effects distribution</u>:

For the model without covariates we set $\theta^*_{i,k} = \theta_{i,k}$

$$\theta_{i,2}, \dots, \theta_{i,K} \sim MVN(\mu_2, \dots, \mu_K, \boldsymbol{T})$$

The matrix T involves the heterogeneity standard deviation τ

<u>Consistency Equations:</u> Each $\mu_2, \dots, \mu_K$ corresponds into a treatment comparison, e.g. $\mu_k \equiv \mu_{AB}$ then we assume consistency by putting $\mu_{AB} = d_A - d_B$, with $d_A = 0$ for an arbitrarily selected reference treatment.

<u>Model for study-level moderators:</u> $\theta^*_{i,k} = \theta_{i,k} + \log(\beta_{1,\kappa}) x_{i,k}$

if treatment 1 is placebo, $\log(\beta_{1,\kappa}) = \log(\beta_{\kappa})$

if treatment 1 ≠placebo, $log(\beta_{1,\kappa}) \equiv \log(\beta_{AB}) = \log(\beta_B) - \log(\beta_A)$ for any active treatments A, B (assumption of consistent coefficients)

The dependant variable $x_{i,k}$ is defined as:

for small-study effects: $x_{i,k} = \frac{1}{r_{i,k}} + \frac{1}{r_{i,1}} + \frac{1}{n_{i,k}-r_{i,k}} + \frac{1}{n_{i,1}-r_{i,1}}$

for other continuous characteristics: $x_{i,k} \equiv x_i = value_i - (value\ of\ centralization)$ (the value of centralization is given before thre results)

for sponsorship: $x_{i,k} = \frac{I_{i,1}-I_{i,k}}{2}$, with $I_{i,k} = \begin{cases} 1, if\ treatment\ k\ is\ sponsored\ in\ study\ i \\ -1, if\ treatment\ k\ is\ non-sponsored\ in\ i \end{cases}$

for single/multi-centre trials: $x_{i,k} \equiv x_i = \begin{cases} 1, if\ \ study\ i\ single - centre \\ 0, if\ \ study\ i\ multi - centre \end{cases}$

There are cases where there are not enough data to estimate separate β_A, β_B, etc. We assumed to be exchangeable any coefficients to improve power and estimation (relevant information is presented before the results). For example, we assumed that β_A, β_B are exchangeable $log\beta_A \sim N(logB, \tau_B^2)$, $log\beta_B \sim N(logB, \tau_B^2)$

Model for novel agent effects: $\theta_{i,k}^{*} = \theta_{i,k} + \log(\beta_{i,1,\kappa})x_{i,k}$

if treatment 1 is placebo, $\log(\beta_{i,1,\kappa}) = 0$

if treatment 1 ≠placebo,$\log(\beta_{i,1,\kappa}) \sim N(\log(\beta_{1,\kappa}), \tau_{log\beta_{1,\kappa}}^2)$

for any active treatments A, B $\log(\beta_{1,\kappa}) \equiv \log(\beta_{AB}) = \log(B)$, $\tau_{log\beta_{1,\kappa}}^2 \equiv \tau_{log\beta_{AB}}^2 = \tau_B^2$

priors: $\log(B) \sim N(0,10^4), \tau_B^2 \sim U(0,5)$

$x_{i,k} = \frac{I_{i,1}-I_{i,k}}{2}$, with $I_{i,k} = \begin{cases} 1, if\ treatment\ k\ is\ novel/experimental\ in\ study\ i \\ -1, if\ treatment\ k\ is\ old/comparator\ in\ study\ i \end{cases}$

Priors:

We assume vague normal priors $N(0,10^4)$ for the paraemters u_i and d_B, d_C, d_D , ...

if β_A is assumed independent $log(\beta_A) \sim N(0,10^4)$

if β_A, β_B are assumed exchangeable $log\beta_A \sim N(logB, \tau_B^2)$, $log\beta_B \sim N(logB, \tau_B^2)$

$logB \sim N(0,10^4), \tau_B^2 \sim U(0,5)$

$\tau \sim U(0,5)$

7.6.2 Changes in heterogeneity after accounting for potential effect modifiers

Covariate	Heterogeneity standard deviation (median and 95% CrI)	Relative change in the variance

	Response	
**None**	0.21 (0.17,0.25)	-
**Study variance**	0.19 (0.15,0.24)	10%
**Year**	**0.18 (0.13,0.22)**	**14%**
**Sponsorship**	0.21 (0.17,0.26)	0%
**Probability of receiving placebo**	0.20 (0.16,0.24)	5%
**Dosing Schedule**	0.21 (0.16,0.25)	0%
**Accounting for novel/experimental agent effects**	0.20 (0.16,0.24)	5%
**Excluding studies with no unpublished data**	0.21 (0.16,0.26)	0%
**Excluding studies with imputed number of responders**	0.21 (0.17,0.26)	0%
**Excluding placebo-controlled trials**	**0.16 (0.04,0.25)**	**24%**
**Including only studies with low to moderate average severity at baseline (HAMD<=24)**	0.25 (0.19,0.32)	0%
**Including only studies with high average severity at baseline (HAMD>24)**	0.24 (0.19,0.30)	0%
**Multi-center vs single center trials**	0.20 (0.16,0.24)	5%
**Including all studies with accepted dose range**	0.20 (0.17,0.24)	5%
	Dropout	
**None**	0.20 (0.15,0.25)	-
**Study variance**	0.19 (0.14,0.24)	5%
**Year**	0.19 (0.14,0.24)	5%
**Sponsorship**	0.20 (0.14,0.25)	0%
**Probability of receiving placebo**	0.19 (0.14,0.24)	5%
**Dosing Schedule**	0.19 (0.14,0.24)	5%
**Excluding studies with no unpublished data**	0.18 (0.11,0.24)	10%
**Excluding placebo-controlled trials**	**0.11 (0.01,0.23)**	**45%**
**Including only studies with low to moderate average severity at baseline (HAMD<=24)**	0.26 (0.18,0.34)	0%
**Including only studies with high average severity at baseline (HAMD>24)**	0.20 (0.13,0.27)	0%
**Multi-center vs single center trials**	0.18 (0.13,0.24)	10%
**Including all studies with accepted dose range**	0.20 (0.15,0.25)	0%

7.6.3 Sensitivity analyses

7.6.3.1 Excluding studies with no unpublished data

	Response							Dropouts for any reason						
	OR drug vs PLA			beta of comparison				OR drug vs PLA			beta of comparison			
	2.5%	median	97.5%	2.5%	median	97.5%		2.5%	median	97.5%	2.5%	median	97.5%	

Agomelatine	1.33	1.53	1.76		0.76	0.89	1.03	
Amitriptyline	1.65	2.05	2.56		0.69	0.87	1.08	
Bupropion	1.20	1.43	1.71		0.80	0.96	1.15	
Citalopram	1.27	1.49	1.74		0.84	1.01	1.19	
Clomipramine	0.90	1.31	1.88		1.02	1.46	2.11	
Desvenlafaxine	1.16	1.49	1.90		1.07	1.39	1.81	
Duloxetine	1.61	1.81	2.04		1.00	1.13	1.29	
Escitalopram	1.43	1.62	1.85		0.86	0.99	1.13	
Fluoxetine	1.27	1.43	1.61		0.80	0.91	1.02	
Fluvoxamine	0.94	1.36	1.97		0.83	1.20	1.75	
Levomilnacipran	1.24	1.59	2.05		0.94	1.19	1.52	
Milnacipran	-	-	-		-	-	-	
Mirtazapine	1.46	1.74	2.08		0.86	1.02	1.21	
Nefazodone	1.23	1.69	2.34		0.62	0.85	1.17	
Paroxetine	1.51	1.66	1.84		0.90	0.99	1.09	
Reboxetine	1.11	1.32	1.58		0.99	1.19	1.42	
Sertraline	1.29	1.60	1.97		0.82	1.01	1.23	
Trazodone	0.94	1.38	2.02		0.95	1.40	2.05	
Venlafaxine	1.49	1.70	1.94		0.93	1.06	1.21	
Vilazodone	1.09	1.44	1.90		0.92	1.26	1.71	
Vortioxetine	1.43	1.65	1.91		0.88	1.03	1.20	

(230 studies included for response)

(264 studies included for dropout)

7.6.3.2 Excluding studies with imputed response

	Response						
	OR drug vs PLA			beta of comparison			
	2.5%	median	97.5%	2.5%	median	97.5%	
Agomelatine	1.48	1.69	1.94				
Amitriptyline	1.87	2.16	2.49				
Bupropion	1.32	1.57	1.87				
Citalopram	1.35	1.60	1.88				
Clomipramine	1.18	1.53	1.99				
Desvenlafaxine	1.21	1.46	1.77				
Duloxetine	1.68	1.88	2.11				
Escitalopram	1.55	1.76	2.01				
Fluoxetine	1.42	1.56	1.71				
Fluvoxamine	1.40	1.76	2.20				
Levomilnacipran	1.24	1.59	2.05				
Milnacipran	1.46	1.95	2.62				

Mirtazapine	1.73	2.04	2.41	
Nefazodone	1.39	1.87	2.52	
Paroxetine	1.66	1.82	2.00	
Reboxetine	1.18	1.40	1.67	
Sertraline	1.52	1.73	1.96	
Trazodone	1.25	1.55	1.92	
Venlafaxine	1.67	1.85	2.06	
Vilazodone	1.33	1.84	2.56	
Vortioxetine	1.45	1.68	1.93	

(321 studies included for response)

7.6.3.3 *Adjusted for effect of novel agents*

	Response						
	OR drug vs PLA			beta of comparison			
	2.5%	median	97.5%	2.5%	median	97.5%	
Agomelatine	1.37	1.56	1.78	1.09	1.18	1.27	
Amitriptyline	2.07	2.36	2.68				
Bupropion	1.31	1.54	1.80				
Citalopram	1.33	1.52	1.74				
Clomipramine	1.40	1.76	2.22				
Desvenlafaxine	1.24	1.48	1.78				
Duloxetine	1.64	1.84	2.05				
Escitalopram	1.43	1.59	1.78				
Fluoxetine	1.47	1.60	1.75				
Fluvoxamine	1.43	1.71	2.05				
Levomilnacipran	1.25	1.59	2.03				
Milnacipran	1.20	1.55	1.99				
Mirtazapine	1.50	1.74	2.03				
Nefazodone	1.25	1.59	2.02				
Paroxetine	1.63	1.76	1.91				
Reboxetine	1.11	1.32	1.57				
Sertraline	1.46	1.64	1.84				
Trazodone	1.27	1.54	1.87				
Venlafaxine	1.65	1.83	2.03				
Vilazodone	1.28	1.60	1.99				
Vortioxetine	1.43	1.65	1.89				

Adjustment took place only in head-to-head trials. To increase power we did not account for differences between the interventions but assumed a single coefficient B for the effect of novel/experimental over older/control trial interventions which was estimated at B= 1.18 (1.09,1.28)

All coefficients were assumed exchangeable.

7.6.3.4 *Inncluding all the studies that used the drugs within the accepted doses*

	Response							Dropouts for any reason						
	OR drug vs PLA			beta of comparison				OR drug vs PLA			beta of comparison			
	2.5%	median	97.5%	2.5%	median	97.5%		2.5%	median	97.5%	2.5%	median	97.5%	
Agomelatine	1.44	1.64	1.87					0.72	0.84	0.97				
Amitriptyline	1.92	2.14	2.38					0.88	0.99	1.11				
Bupropion	1.40	1.58	1.80					0.85	0.97	1.11				
Citalopram	1.39	1.57	1.77					0.81	0.94	1.08				
Clomipramine	1.27	1.56	1.91					0.94	1.21	1.55				
Desvenlafaxine	1.24	1.49	1.78					0.88	1.08	1.33				
Duloxetine	1.65	1.85	2.07					0.96	1.08	1.22				
Escitalopram	1.51	1.68	1.87					0.81	0.91	1.03				
Fluoxetine	1.41	1.52	1.65					0.80	0.88	0.96				
Fluvoxamine	1.41	1.68	2.01					0.93	1.12	1.35				
Levomilnacipran	1.24	1.59	2.04					0.93	1.19	1.53				
Milnacipran	1.37	1.74	2.22					0.73	0.96	1.26				
Mirtazapine	1.60	1.84	2.12					0.85	0.98	1.14				
Nefazodone	1.23	1.53	1.90					0.76	0.94	1.17				
Paroxetine	1.62	1.75	1.89					0.87	0.94	1.03				
Reboxetine	1.15	1.36	1.60					0.97	1.16	1.39				
Sertraline	1.46	1.62	1.80					0.88	0.99	1.11				
Trazodone	1.29	1.55	1.86					0.90	1.10	1.36				
Venlafaxine	1.59	1.74	1.91					0.92	1.01	1.12				
Vilazodone	1.21	1.46	1.77					0.89	1.12	1.42				
Vortioxetine	1.44	1.66	1.91					0.86	1.01	1.19				

(474 studies included for response)

(464 studies included for dropout)

7.6.4 Summary of significance in the regression coefficients from meta-regressions

The following table has been constructed using the convention that statistically significant effects are identified by a 95% CrI that excludes the value of 1. Because of multiple-testing issues significance levels should be higher than nominal and hence this table is for presentation purposes only. Bold corresponds to coefficients that exclude a 10% relative increase or decrease in the odds (OR 1.1 or 0.9).

	Response	***Dropout***

	Small study effects	Year	Sponsorship	Probability of receiving placebo	Multi-center studies	Baseline severity	Small study effects	Year	Sponsorship	Prob of receiving placebo	Multicenter studies	Baseline severity
**Agomelatine**									X			
**Amitriptyline**	**X**	X		X	**X**	X	**X**	X		X	**X**	
**Bupropion**	**X**	X				**X**						
**Citalopram**					X						X	
**Clomipramine**												
**Desvenlafaxine**										X		
**Duloxetine**		X					**X**					X
**Escitalopram**												
**Fluoxetine**	**X**	**X**			**X**			X			**X**	X
**Fluvoxamine**	X			X		**X**						
**Levomilnacipran**												
**Milnacipran**												
**Mirtazapine**												X
**Nefazodone**												
**Paroxetine**		X			**X**			X			**X**	
**Reboxetine**		**X**					**X**	**X**				**X**
**Sertraline**		X						X				X
**Trazodone**	**X**			X	**X**							
**Venlafaxine**		**X**						**X**				
**Vilazodone**												
**Vortioxetine**												

7.6.5 Odds-ratios for response and dropout for any reason adjusted for covariates.

Percentage (%) change between adjusted and unadjusted ORs were estimated when the adjusted ORs could be considered as more 'trustworthy', e.g. when extrapolated at studies with very large sample size or estimated from multicenter studies

7.6.5.1 Unadjusted

	Response						Dropouts for any reason					
	OR drug vs PLA			beta of comparison			OR drug vs PLA			beta of comparison		
	2.5%	median	97.5%	2.5%	median	97.5%	2.5%	median	97.5%	2.5%	median	97.5%
Agomelatine	1.44	1.65	1.88				0.72	0.84	0.97			

Amitriptyline	1.89	2.13	2.41				0.83	0.95	1.08			
Bupropion	1.35	1.58	1.86				0.81	0.96	1.14			
Citalopram	1.33	1.52	1.74				0.80	0.94	1.09			
Clomipramine	1.21	1.49	1.85				1.01	1.30	1.68			
Desvenlafaxine	1.24	1.49	1.79				0.88	1.08	1.33			
Duloxetine	1.66	1.85	2.07				0.96	1.09	1.23			
Escitalopram	1.50	1.68	1.87				0.80	0.90	1.02			
Fluoxetine	1.40	1.52	1.66				0.80	0.88	0.96			
Fluvoxamine	1.41	1.69	2.02				0.91	1.10	1.33			
Levomilnacipran	1.24	1.59	2.05				0.93	1.19	1.53			
Milnacipran	1.37	1.74	2.23				0.73	0.95	1.26			
Mirtazapine	1.64	1.89	2.20				0.85	0.99	1.15			
Nefazodone	1.32	1.67	2.12				0.72	0.93	1.19			
Paroxetine	1.61	1.75	1.90				0.87	0.95	1.03			
Reboxetine	1.16	1.37	1.63				0.96	1.16	1.40			
Sertraline	1.49	1.67	1.87				0.85	0.96	1.08			
Trazodone	1.25	1.51	1.83				0.93	1.15	1.42			
Venlafaxine	1.61	1.78	1.96				0.93	1.04	1.15			
Vilazodone	1.28	1.60	2.00				0.88	1.14	1.47			
Vortioxetine	1.45	1.66	1.92				0.86	1.01	1.19			

7.6.5.2 *Adjusted for small-study effects*

	Response						Dropouts for any reason					
	OR drug vs PLA			beta of comparison			OR drug vs PLA			beta of comparison		
	2.5%	median	97.5%	2.5%	median	97.5%	2.5%	median	97.5%	2.5%	median	97.5%
Agomelatine	1.24	1.53	1.88	0.03	2.19	157.91	0.74	0.92	1.14	0.03	0.51	7.78
Amitriptyline	1.51	1.75	2.04	2.40	7.10	21.12	0.99	1.15	1.35	0.05	0.13	0.37
Bupropion	1.11	1.34	1.62	1.21	4.56	19.18	0.87	1.07	1.32	0.11	0.46	1.91
Citalopram	1.19	1.45	1.76	0.02	1.27	64.39	0.80	0.99	1.22	0.08	0.95	11.54
Clomipramine	1.08	1.34	1.67	-	-	-	1.12	1.46	1.91	0.01	0.15	1.75
Desvenlafaxine	0.62	1.14	2.04	-	-	-	0.64	1.18	2.18	-	-	-
Duloxetine	1.26	1.56	1.93	-	-	-	1.09	1.36	1.70	0.00	0.05	0.70
Escitalopram	1.51	1.80	2.13	0.00	0.05	1.07	0.78	0.94	1.15	0.10	1.09	11.95
Fluoxetine	1.21	1.34	1.50	1.43	3.77	10.03	0.86	0.97	1.10	0.15	0.46	1.41
Fluvoxamine	1.16	1.42	1.75	1.04	3.71	13.79	1.00	1.24	1.54	0.13	0.50	1.88
Levomilnacipran	1.00	3.52	14.08	-	-	-	0.93	1.37	2.07	0.00	0.10	20.76
Milnacipran	1.19	1.52	1.95	-	-	-	0.81	1.06	1.40	-	-	-
Mirtazapine	1.38	1.66	2.00	0.62	3.32	17.94	0.91	1.12	1.38	0.05	0.29	1.88
Nefazodone	0.97	1.40	2.02	0.21	4.66	102.82	0.73	1.09	1.66	0.01	0.24	5.69

Paroxetine	1.42	1.57	1.75	0.84	2.01	4.79	0.94	1.05	1.16	0.31	0.61	1.19
Reboxetine	0.86	1.06	1.31	-	-	-	1.22	1.55	1.97	0.00	0.03	0.22
Sertraline	1.33	1.54	1.78	0.21	1.24	7.32	0.82	0.97	1.13	0.60	2.48	10.72
Trazodone	1.08	1.32	1.62	1.13	2.45	6.48	1.01	1.30	1.67	0.05	0.32	1.86
Venlafaxine	1.35	1.56	1.81	0.64	6.38	66.02	1.01	1.19	1.40	0.02	0.16	1.26
Vilazodone	1.44	2.89	5.74	0.00	0.00	4.74	0.45	1.13	2.77	-	-	-
Vortioxetine	1.06	1.62	2.48	-	-	-	0.63	0.93	1.36	-	-	-

*Parameters with estimated intervals from 0 to >100 have been substituted by NA

The ORs presented are extrapolated for a study with infinite sample size (zero variance).

Then the OR of each active intervention j is multiplied by beta for one unit increase in the variance of the logOR.

Coefficients assumed exchangeable in response and dropout: beta[1,6], beta[1,12], beta[1,13]

7.6.5.3 *Adjusted for year of studies*

	Response						Dropouts for any reason					
	OR drug vs PLA			beta of comparison			OR drug vs PLA			beta of comparison		
	2.5%	median	97.5%	2.5%	median	97.5%	2.5%	median	97.5%	2.5%	median	97.5%
Agomelatine	0.94	1.27	1.72	0.53	0.76	1.09	0.72	1.05	1.50	0.80	1.24	1.91
Amitriptyline	1.64	1.96	2.35	1.02	1.26	1.55	0.87	1.07	1.31	0.62	0.77	0.96
Bupropion	1.34	1.56	1.82	1.06	1.25	1.48	0.83	0.99	1.17	0.76	0.92	1.11
Citalopram	1.31	1.54	1.80	0.81	1.01	1.26	0.79	0.95	1.14	0.73	0.94	1.22
Clomipramine	1.18	1.64	2.28	0.65	1.05	1.70	0.89	1.40	2.21	0.38	0.72	1.39
Desvenlafaxine	1.11	2.04	3.69	0.80	1.29	2.04	0.76	1.68	3.64	0.77	1.43	2.60
Duloxetine	1.11	1.44	1.88	0.58	0.76	1.01	0.89	1.17	1.54	0.79	1.05	1.41
Escitalopram	1.44	1.85	2.39	0.86	1.19	1.64	0.82	1.10	1.48	0.86	1.26	1.83
Fluoxetine	1.47	1.60	1.74	1.22	1.38	1.57	0.78	0.85	0.94	0.68	0.79	0.91
Fluvoxamine	1.35	1.66	2.05	0.82	1.07	1.40	0.85	1.06	1.33	0.78	1.10	1.54
Levomilnacipran	0.59	2.60	11.57	0.45	1.50	5.03	0.06	0.33	1.71	0.09	0.35	1.33
Milnacipran	1.43	1.82	2.33	0.82	1.08	1.43	0.69	0.93	1.25	0.61	0.85	1.19
Mirtazapine	1.64	1.93	2.27	0.85	1.17	1.61	0.85	1.01	1.21	0.68	0.96	1.35
Nefazodone	1.25	1.80	2.61	0.33	1.01	3.12	0.65	0.98	1.48	0.23	0.75	2.42
Paroxetine	1.66	1.80	1.95	0.95	1.06	1.18	0.85	0.93	1.01	0.78	0.87	0.98
Reboxetine	1.24	1.46	1.72	1.21	1.87	2.92	0.92	1.11	1.33	0.28	0.46	0.77
Sertraline	1.58	1.78	2.00	1.01	1.27	1.60	0.80	0.91	1.04	0.55	0.72	0.93
Trazodone	1.36	1.64	1.98	0.83	1.00	1.21	0.89	1.10	1.37	0.69	0.85	1.05
Venlafaxine	1.86	2.12	2.42	1.30	1.59	1.95	0.77	0.89	1.03	0.51	0.64	0.81
Vilazodone	0.31	1.13	4.11	0.26	0.69	1.86	0.20	0.87	3.51	0.26	0.82	2.39
Vortioxetine	0.72	1.56	3.30	0.53	0.96	1.72	0.30	0.79	2.00	0.38	0.79	1.63

The ORs are estimated/extrapolated for a study published in 1999 (centralisation value). Then, for every ten years increase, the OR is multiplied by beta.

No coefficients were assumed exchangeable.

7.6.5.4 *Adjusted for sponsorship*

	Response						Dropouts for any reason					
	OR drug vs PLA			beta of comparison			OR drug vs PLA			beta of comparison		
	2.5%	median	97.5%	2.5%	median	97.5%	2.5%	median	97.5%	2.5%	median	97.5%
Agomelatine	1.44	1.78	2.19	0.60	0.85	1.18	0.62	0.75	0.92	1.03	1.40	1.90
Amitriptyline	1.97	2.32	2.72	0.47	0.73	1.14	0.79	0.94	1.12	0.66	1.00	1.52
Bupropion	1.20	1.61	2.16	0.65	0.97	1.46	0.74	0.96	1.24	0.73	1.04	1.46
Citalopram	1.23	1.48	1.79	0.79	1.04	1.38	0.67	0.84	1.06	0.85	1.24	1.81
Clomipramine	1.10	1.50	2.05	0.35	0.77	1.33	0.90	1.35	1.98	0.47	0.96	2.17
Desvenlafaxine	1.04	1.62	2.51	0.52	0.89	1.54	0.78	1.20	2.01	0.43	0.86	1.46
Duloxetine	1.52	1.81	2.16	0.81	1.04	1.35	1.06	1.27	1.52	0.47	0.66	0.91
Escitalopram	1.47	1.73	2.03	0.73	0.93	1.20	0.74	0.87	1.03	0.80	1.06	1.42
Fluoxetine	1.31	1.48	1.67	0.85	1.06	1.33	0.76	0.87	1.00	0.81	1.02	1.29
Fluvoxamine	1.23	1.56	1.96	0.99	1.49	2.27	0.84	1.15	1.58	0.45	0.80	1.40
Levomilnacipran	1.10	1.98	4.25	0.35	0.79	1.42	0.84	1.44	3.15	0.32	0.80	1.40
Milnacipran	1.21	1.65	2.23	0.43	0.86	1.70	0.70	0.96	1.32	0.54	1.07	2.10
Mirtazapine	1.59	1.91	2.29	0.65	0.88	1.21	0.78	0.94	1.14	0.87	1.17	1.56
Nefazodone	1.05	1.56	2.30	0.66	1.14	2.00	0.62	0.94	1.44	0.53	0.95	1.69
Paroxetine	1.59	1.80	2.03	0.73	0.90	1.11	0.83	0.94	1.06	0.85	1.03	1.26
Reboxetine	0.94	1.30	1.76	0.68	0.99	1.52	0.84	1.22	1.76	0.57	0.93	1.53
Sertraline	1.44	1.69	1.97	0.71	0.96	1.31	0.74	0.87	1.03	0.85	1.15	1.57
Trazodone	1.17	1.48	1.87	0.69	1.26	2.34	0.90	1.15	1.47	0.54	0.95	1.67
Venlafaxine	1.52	1.75	2.02	0.84	1.08	1.40	0.91	1.06	1.23	0.68	0.88	1.14
Vilazodone	1.24	1.90	3.23	0.36	0.77	1.32	0.70	1.33	3.41	0.32	0.86	1.60
Vortioxetine	1.43	1.97	2.93	0.41	0.76	1.21	0.66	0.97	1.34	0.71	1.10	1.95

The ORs are extrapolated for a study without sponsoring. When the study is sponsored then the OR is on average multiplied by beta. Hence, beta>1 means that sponsoring magnifies the efficacy of the active intervention. It was not possible to estimate effects for interventions that are sponsored in all included studies (or not sponsored in all included studies) such as vortioxetine.

7.6.5.5 *Adjusted for probability of receiving placebo*

	Response		Dropouts for any reason	
	OR drug vs PLA	beta of comparison	OR drug vs PLA	beta of comparison

	2.5%	median	97.5%	2.5%	median	97.5%	2.5%	median	97.5%	2.5%	median	97.5%
Agomelatine	1.25	1.57	1.98	0.47	1.04	2.28	0.67	0.87	1.14	0.39	0.93	2.22
Amitriptyline	2.40	3.16	4.17	1.75	4.02	9.37	0.48	0.66	0.89	0.11	0.29	0.72
Bupropion	1.23	1.60	2.08	0.44	1.29	3.70	0.65	0.86	1.14	0.17	0.50	1.45
Citalopram	1.02	1.42	1.97	0.38	0.99	2.67	0.77	1.15	1.69	0.48	1.52	4.57
Clomipramine	0.97	1.28	1.69	-	-	-	0.01	0.19	1.79	-	-	-
Desvenlafaxine	1.10	1.58	2.30	0.19	1.54	12.96	0.50	0.75	1.13	0.01	0.09	0.92
Duloxetine	1.69	2.09	2.59	0.82	1.80	4.02	0.74	0.94	1.19	0.21	0.49	1.16
Escitalopram	1.13	1.39	1.72	0.31	0.63	1.35	0.86	1.09	1.37	0.74	1.62	3.63
Fluoxetine	1.45	1.73	2.06	0.97	1.85	3.57	0.66	0.80	0.96	0.31	0.62	1.19
Fluvoxamine	1.61	2.60	4.10	1.16	4.00	13.17	0.53	0.90	1.56	0.13	0.51	1.90
Levomilnacipran	1.19	1.63	2.24	0.13	1.29	12.97	0.75	1.05	1.46	0.02	0.26	2.72
Milnacipran	1.06	1.42	1.91	-	-	-	0.00	0.08	2.68	-	-	-
Mirtazapine	1.40	1.96	2.72	0.55	1.41	3.57	0.63	0.88	1.24	0.23	0.61	1.60
Nefazodone	1.29	2.07	3.31	0.58	2.49	10.70	0.45	0.72	1.17	0.08	0.36	1.71
Paroxetine	1.47	1.76	2.13	0.69	1.34	2.68	0.77	0.92	1.11	0.36	0.73	1.43
Reboxetine	1.25	1.69	2.30	0.92	2.69	8.12	0.66	0.91	1.25	0.10	0.32	1.07
Sertraline	1.30	1.65	2.08	0.59	1.27	2.63	0.89	1.14	1.46	0.64	1.41	3.10
Trazodone	1.41	1.97	2.76	1.17	3.13	8.54	0.84	1.19	1.69	0.31	0.90	2.60
Venlafaxine	1.66	2.10	2.66	0.91	1.92	4.06	0.66	0.85	1.08	0.21	0.48	1.01
Vilazodone	1.34	1.85	2.55	0.55	3.30	19.91	0.72	1.01	1.41	0.04	0.29	2.18
Vortioxetine	0.91	1.26	1.76	0.08	0.30	1.22	0.74	1.06	1.54	0.26	1.13	5.31

The ORs estimated are for a study with 0% probability of receiving placebo (head-to-head studies). Then, the ORs are multiplied by beta for every 20% increase in the probability; beta[1,j]<1 means that the higher the probability of receiving placebo, the smalled the effectiveness of the intervention.

Betas were not estimable for clomipramine and milnacipran because they feature only in head-to-head studies (so the covariate is always 0%).

7.6.5.6 Multicenter trials

	Response						Dropouts for any reason					
	OR drug vs PLA			beta of comparison			OR drug vs PLA			beta of comparison		
	2.5%	median	97.5%	2.5%	median	97.5%	2.5%	median	97.5%	2.5%	median	97.5%
Agomelatine	1.42	1.61	1.84	-	-	-	0.75	0.86	0.99	-	-	-
Amitriptyline	1.67	1.92	2.21	1.24	1.74	2.44	0.90	1.05	1.22	0.44	0.63	0.88
Bupropion	1.29	1.51	1.77	0.16	0.91	6.49	0.84	0.99	1.17	-	-	-
Citalopram	1.27	1.45	1.66	1.10	3.18	9.50	0.84	0.98	1.14	0.03	0.19	0.95
Clomipramine	1.15	1.43	1.77	0.41	1.63	8.92	1.09	1.41	1.82	-	-	-
Desvenlafaxine	1.24	1.49	1.78	-	-	-	0.88	1.08	1.32	-	-	-

Duloxetine	1.64	1.83	2.04	-	-	-	0.98	1.10	1.24	-	-	-
Escitalopram	1.47	1.64	1.82	0.93	3.50	13.41	0.82	0.92	1.04	0.01	0.13	1.00
Fluoxetine	1.33	1.45	1.58	1.28	1.84	2.64	0.84	0.93	1.02	0.40	0.58	0.84
Fluvoxamine	1.28	1.55	1.87	0.83	1.37	2.29	0.95	1.16	1.42	0.55	0.94	1.60
Levomilnacipran	1.25	1.59	2.03	-	-	-	0.94	1.19	1.52	-	-	-
Milnacipran	1.28	1.64	2.09	-	-	-	0.77	1.01	1.32	-	-	-
Mirtazapine	1.48	1.75	2.07	0.98	1.62	2.72	0.91	1.09	1.30	0.35	0.61	1.04
Nefazodone	1.24	1.62	2.10	0.65	1.15	2.05	0.70	0.93	1.23	0.55	1.01	1.82
Paroxetine	1.53	1.67	1.81	1.14	1.51	2.00	0.92	1.00	1.09	0.53	0.69	0.90
Reboxetine	1.14	1.34	1.59	-	-	-	0.99	1.19	1.42	-	-	-
Sertraline	1.43	1.60	1.81	0.54	1.06	2.09	0.88	1.00	1.13	0.88	2.32	6.53
Trazodone	1.13	1.39	1.72	1.14	2.17	4.12	0.96	1.21	1.53	0.36	0.73	1.46
Venlafaxine	1.61	1.78	1.97	0.24	0.49	0.99	0.94	1.05	1.17	0.74	1.56	3.27
Vilazodone	1.28	1.59	1.98	-	-	-	0.90	1.15	1.47	-	-	-
Vortioxetine	1.45	1.66	1.90	-	-	-	0.87	1.02	1.19	-	-	-

The ORs estimated are estimated from studies in multi-center trials. Then, the ORs are multiplied by beta for single center trials

7.6.5.7 Adjusted for baseline severity (continuous)

	Response						Dropouts for any reason					
	OR drug vs PLA			beta of comparison			OR drug vs PLA			beta of comparison		
	2.5%	median	97.5%	2.5%	median	97.5%	2.5%	median	97.5%	2.5%	median	97.5%
Agomelatine	1.33	1.57	1.84	0.76	1.36	2.43	0.75	0.89	1.06	0.39	0.71	1.30
Amitriptyline	1.77	2.03	2.32	1.09	1.58	2.31	0.83	0.96	1.11	0.38	0.63	1.05
Bupropion	1.27	1.51	1.81	1.12	2.17	4.22	0.76	0.94	1.15	0.34	0.99	2.90
Citalopram	1.33	1.60	1.92	0.84	1.28	1.93	0.77	0.95	1.16	0.54	0.86	1.36
Clomipramine	1.19	1.49	1.87	0.59	0.98	1.65	0.98	1.27	1.65	0.47	0.80	1.37
Desvenlafaxine	0.92	1.44	2.27	0.04	0.79	16.78	1.17	1.99	3.40	-	-	-
Duloxetine	1.54	1.80	2.10	0.64	0.94	1.36	1.09	1.29	1.53	1.07	1.59	2.35
Escitalopram	1.42	1.62	1.85	0.64	0.88	1.22	0.81	0.93	1.07	0.77	1.10	1.58
Fluoxetine	1.38	1.50	1.64	0.95	1.27	1.68	0.81	0.89	0.97	0.54	0.72	0.96
Fluvoxamine	1.36	1.63	1.96	1.24	2.58	5.38	0.94	1.14	1.39	0.25	0.55	1.24
Levomilnacipran	1.22	1.60	2.10	0.40	1.03	2.64	0.90	1.19	1.56	0.24	0.63	1.66
Milnacipran	1.38	1.77	2.28	0.46	1.57	5.39	0.73	0.96	1.26	0.16	0.59	2.14
Mirtazapine	1.58	1.85	2.16	0.97	1.72	3.06	0.86	1.01	1.19	0.28	0.51	0.94
Nefazodone	1.31	1.67	2.11	0.42	2.54	15.56	0.73	0.94	1.20	0.06	0.35	2.10
Paroxetine	1.60	1.74	1.89	0.78	1.01	1.30	0.89	0.97	1.05	0.63	0.82	1.07
Reboxetine	0.76	1.08	1.51	0.89	3.29	12.33	1.30	1.87	2.67	0.02	0.10	0.44
Sertraline	1.46	1.66	1.89	0.83	1.22	1.78	0.78	0.90	1.03	0.33	0.51	0.80
Trazodone	1.31	1.61	1.97	0.40	0.88	1.97	0.95	1.18	1.47	0.21	0.49	1.14

Venlafaxine	1.57	1.74	1.92	0.98	1.35	1.86	0.93	1.03	1.15	0.51	0.72	1.00
Vilazodone	1.04	1.40	1.89	0.28	0.62	1.32	0.81	1.13	1.58	0.42	0.92	2.04
Vortioxetine	1.39	1.65	1.96	0.71	1.03	1.50	0.83	1.01	1.22	0.58	0.87	1.31

The ORs are estimated/extrapolated for a study with average baseline severity of 24.5 on HAMD17 (centralisation value). Then, for 10 units of increase, the OR is multiplied by beta. However, we observed that the association might not be linear and hence we perforemed a subgroup analysis of studies with average socre of 24 or below (moderate to low severity) versus studies with average severity above 24 (severe).
No coefficients were assumed exchangeable.

7.6.5.8 *Adjusted for baseline severity (dichotomised)*

	Response						Dropouts for any reason					
	<=24			>24			<=24			>24		
	2.5%	median	97.5%	2.5%	median	97.5%	2.5%	median	97.5%	2.5%	median	97.5%
Agomelatine	-	-	-	1.46	1.70	1.98	-	-	-	0.70	0.83	0.97
Amitriptyline	1.52	1.93	2.45	1.89	2.20	2.57	0.79	1.02	1.31	0.74	0.87	1.03
Bupropion	0.89	1.27	1.80	1.36	1.69	2.11	0.63	0.99	1.54	0.74	0.91	1.12
Citalopram	1.20	1.43	1.70	1.30	1.71	2.24	0.76	0.95	1.18	0.70	0.92	1.20
Clomipramine	1.13	1.57	2.20	0.90	1.27	1.79	0.88	1.30	1.94	0.84	1.23	1.80
Desvenlafaxine	1.17	1.45	1.81	0.97	1.80	3.34	0.81	1.04	1.33	0.70	1.50	3.37
Duloxetine	1.54	1.78	2.05	1.49	1.94	2.52	0.85	1.00	1.17	1.07	1.40	1.83
Escitalopram	1.46	1.73	2.04	1.35	1.60	1.91	0.68	0.83	1.01	0.79	0.95	1.13
Fluoxetine	1.15	1.36	1.61	1.40	1.55	1.73	0.76	0.92	1.11	0.76	0.85	0.94
Fluvoxamine	0.96	1.32	1.82	1.52	1.92	2.42	0.75	1.12	1.69	0.85	1.06	1.33
Levomilnacipran	1.07	1.66	2.60	1.09	1.54	2.19	0.84	1.36	2.20	0.84	1.17	1.64
Milnacipran	1.06	1.62	2.46	1.36	1.91	2.71	0.56	0.94	1.56	0.64	0.91	1.31
Mirtazapine	1.27	1.70	2.26	1.62	1.97	2.40	0.89	1.22	1.68	0.70	0.85	1.03
Nefazodone	1.05	1.52	2.20	1.25	1.75	2.46	0.66	0.98	1.46	0.64	0.90	1.27
Paroxetine	1.45	1.65	1.89	1.58	1.78	2.01	0.83	0.96	1.11	0.81	0.91	1.02
Reboxetine	-	-	-	1.17	1.41	1.70	-	-	-	0.94	1.14	1.37
Sertraline	1.23	1.47	1.75	1.49	1.81	2.20	0.88	1.07	1.30	0.65	0.80	0.98
Trazodone	1.27	1.78	2.49	1.07	1.41	1.85	0.85	1.24	1.81	0.79	1.05	1.39
Venlafaxine	1.25	1.51	1.82	1.57	1.79	2.04	0.78	0.97	1.20	0.87	1.00	1.14
Vilazodone	1.15	1.55	2.11	1.04	1.61	2.50	0.83	1.21	1.75	0.68	1.04	1.59
Vortioxetine	1.31	1.61	1.97	1.34	1.72	2.20	0.84	1.06	1.35	0.76	0.99	1.28

The percentage change for this variable is between subgroups and shows the % decrease in the ORs for response or dropout between moderate/severe depression vs severe depression (=ORlowmoderate/ORsevere - 1).

7.6.5.9 *Adjusted for dosing schedule*

	Response							Dropouts for any reason						
	Fixed dose			Flexible dose				Fixed dose			Flexible dose			
	2.5%	median	97.5%	2.5%	median	97.5%		2.5%	median	97.5%	2.5%	median	97.5%	
Agomelatine	1.13	1.38	1.67	0.99	1.31	1.74		0.82	1.01	1.25	0.51	0.69	0.94	
Amitriptyline	1.29	1.85	2.69	0.83	1.24	1.82		0.82	1.16	1.68	0.51	0.77	1.12	
Bupropion	1.07	1.40	1.83	0.87	1.21	1.68		0.71	0.96	1.29	0.71	1.02	1.46	
Citalopram	1.19	1.42	1.69	0.84	1.11	1.46		0.87	1.06	1.29	0.55	0.77	1.07	
Clomipramine	1.06	1.58	2.35	0.61	0.99	1.63		0.79	1.20	1.84	0.66	1.15	1.98	
Desvenlafaxine	1.24	1.49	1.79	-	-	-		0.88	1.08	1.33	-	-	-	
Duloxetine	1.63	1.85	2.11	0.44	0.92	1.92		0.96	1.10	1.26	0.39	0.82	1.71	
Escitalopram	1.54	1.82	2.13	0.67	0.84	1.06		0.66	0.79	0.95	1.05	1.36	1.74	
Fluoxetine	1.20	1.38	1.60	0.96	1.15	1.38		0.84	0.99	1.17	0.67	0.82	1.00	
Fluvoxamine	0.75	1.13	1.73	1.03	1.64	2.59		0.94	1.34	1.93	0.48	0.74	1.13	
Levomilnacipran	1.05	1.56	2.33	0.62	1.04	1.73		1.02	1.53	2.28	0.40	0.67	1.11	
Milnacipran	1.06	1.46	2.03	0.59	1.42	3.47		0.73	1.03	1.46	0.45	1.53	5.33	
Mirtazapine	1.35	1.78	2.35	0.81	1.12	1.56		0.88	1.16	1.54	0.55	0.77	1.07	
Nefazodone	0.38	1.01	2.58	0.66	1.75	4.72		0.51	1.36	3.51	0.24	0.65	1.79	
Paroxetine	1.40	1.59	1.81	0.97	1.15	1.37		0.88	1.00	1.14	0.76	0.91	1.08	
Reboxetine	1.35	2.09	3.27	0.39	0.63	1.01		0.45	0.74	1.23	0.93	1.60	2.77	
Sertraline	0.86	1.27	1.91	0.90	1.37	2.07		0.87	1.26	1.83	0.48	0.72	1.07	
Trazodone	0.62	1.34	2.82	0.54	1.16	2.56		0.62	1.35	3.13	0.35	0.83	1.87	
Venlafaxine	1.47	1.74	2.06	0.85	1.04	1.29		0.89	1.06	1.27	0.75	0.94	1.19	
Vilazodone	1.26	1.58	1.98	-	-	-		0.90	1.16	1.50	-	-	-	
Vortioxetine	1.44	1.67	1.94	-	-	-		0.87	1.02	1.21	-	-	-	

The percentage change for this variable is between subgroups and shows the % decrease in the ORs for response or dropout betweem fixed vs flexible dose (=ORfixed/ORflexible - 1).

7.6.6 Summary of the findings from meta-regressions and sugroup analyses

The effect of amitriptyline (response and dropoput) was moderated by all but one variable. The other drugs had variable associations, depending on outcome and analysis.

Variables that impact on the relative response and dropout of interventions over placebo. The use of placebo in trials is the strongest explanation of heterogeneity among those evaluated (section 7.6.2). Smaller studies and older studies presented larger effects of the active interventions versus placebo (in particular for amitriptyline, buproprion, fluoxetine and reboxetine) (see sections 7.6.2, 7.6.5). These three variables were considered the most important effect modifiers to consider further.

Variables with questionable impact on the effectiveness of interventions over placebo. Multi-center trials tended to show smaller relative responses and larger dropout rates for active versus placebo. However, the relative change in the odds ratios between multi-center studies and all data was very small and the drop in heterogeneity negligible. Studies with patients with more severe depression showed larger effectiveness and less dropout than studies with moderate/low depression for most drugs and for most active interventions. The impact of baseline severity appears to be important for the effectiveness of fluvoxamine and the dropout rates of desvenlafaxine, reboxetine and sertraline. However the heterogeneity remains unchanged once accounting for differences in baseline severity and given the risk of ecological bias we did not consider this evidence conclusive. Analysis of studies with fixed dosing schedule showed smaller relative response and larger dropout rates compared to flexible dose. However, none of these differences was significant and the change in heterogeneity was negligeable.

Variables that do not impact on the effectiveness of interventions over placebo. Excluding studies for which we had no access to unpublished data or for which we had imputed the response did not impact on the estimates or the heterogeneity. Likewise, including all studies with accepted dose range did not have any material impact on the estimates or the heterogeneity. We could not detect any important impact of sponsorship. Accounting for novel agent effects (wish bias) yields a statistically significant coefficient indicating that the effectiveness of an intervention is magnified by on average 10% (OR 1.1) when it is the experimental intervention in a comparison rather than the reference. However, the novel/experimental-agent adjusted ORs and the heterogeneity were not materially different to those from the unadjusted model. Note that this model is the only one that uses exchangeable coefficients for all interventions and hence the power to estimate the impact of this variable is very large.

8 *Analyses of the head-to-head trials network*

8.1 Results from network meta-analyses

The results below show the relative efficacy and acceptability of all drugs versus reboxetine.

8.1.1 Response

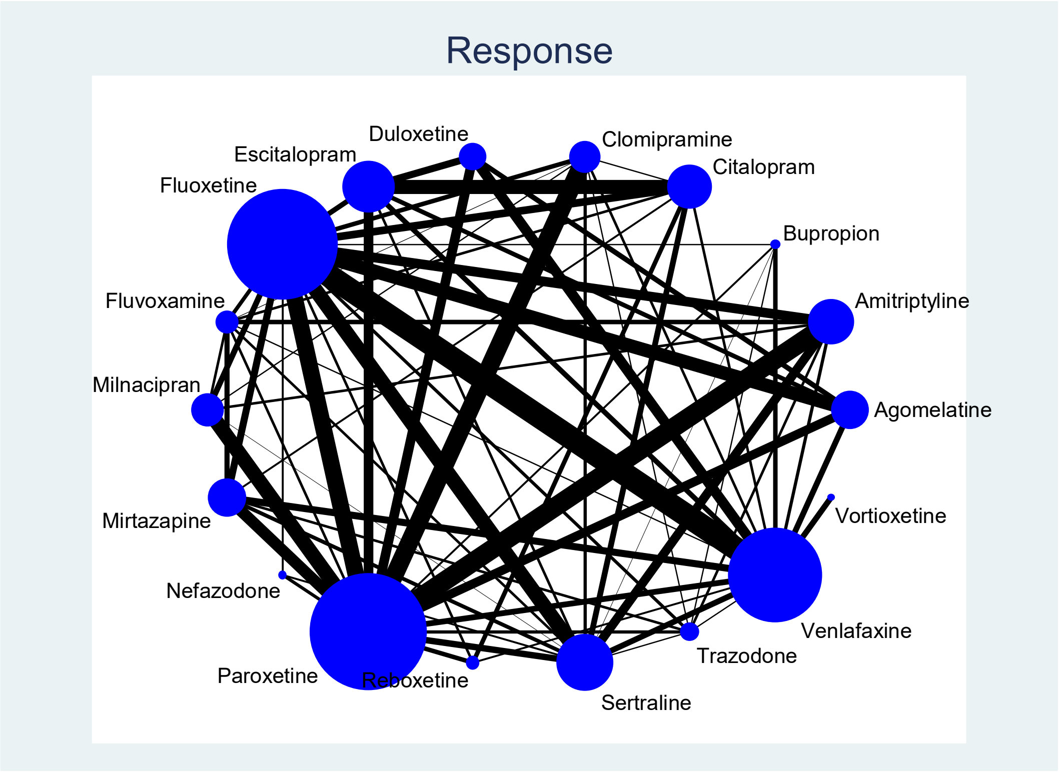

Response

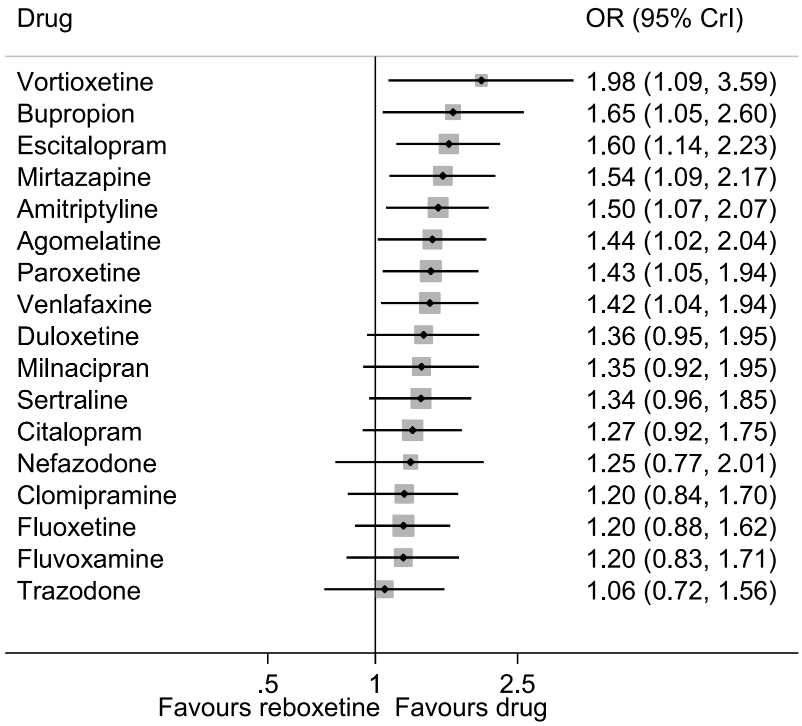

**Agom**																	
0.96 (0.76,1.24)	**Amit**																
0.87 (0.59,1.30)	0.91 (0.62,1.31)	**Bupr**															
1.13 (0.88,1.47)	1.18 (0.93,1.49)	1.30 (0.88,1.93)	**Cita**														
1.20 (0.91,1.59)	1.24 (0.98,1.58)	1.37 (0.93,2.04)	1.06 (0.82,1.38)	**Clom**													
1.06 (0.82,1.37)	1.10 (0.84,1.42)	1.21 (0.81,1.81)	0.93 (0.71,1.22)	0.88 (0.66,1.18)	**Dulo**												
0.90 (0.71,1.14)	0.93 (0.74,1.17)	1.03 (0.70,1.51)	0.79 (0.65,0.97)	0.75 (0.58,0.97)	0.85 (0.67,1.08)	**Esci**											
1.20 (0.99,1.48)	1.25 (1.06,1.48)	1.38 (0.97,1.97)	1.06 (0.87,1.29)	1.00 (0.81,1.24)	1.14 (0.91,1.44)	1.34 (1.11,1.61)	**Fluo**										
1.20 (0.91,1.61)	1.25 (0.99,1.59)	1.38 (0.93,2.07)	1.06 (0.82,1.39)	1.01 (0.76,1.32)	1.14 (0.85,1.54)	1.34 (1.03,1.75)	1.00 (0.80,1.25)	**Fluvo**									
1.07 (0.80,1.44)	1.11 (0.86,1.43)	1.23 (0.81,1.85)	0.94 (0.71,1.26)	0.89 (0.67,1.19)	1.01 (0.74,1.38)	1.19 (0.90,1.58)	0.89 (0.70,1.13)	0.89 (0.67,1.17)	**Miln**								
0.93 (0.72,1.21)	0.97 (0.77,1.21)	1.07 (0.73,1.57)	0.82 (0.65,1.05)	0.78 (0.60,1.01)	0.88 (0.67,1.16)	1.04 (0.82,1.32)	0.78 (0.64,0.94)	0.78 (0.60,0.99)	0.87 (0.66,1.15)	**Mirt**							
1.15 (0.76,1.76)	1.19 (0.80,1.78)	1.32 (0.80,2.20)	1.01 (0.67,1.54)	0.96 (0.63,1.45)	1.09 (0.71,1.68)	1.28 (0.86,1.94)	0.96 (0.66,1.40)	0.95 (0.63,1.46)	1.07 (0.70,1.67)	1.23 (0.82,1.86)	**Nefa**						
1.01 (0.82,1.24)	1.05 (0.89,1.23)	1.16 (0.81,1.64)	0.89 (0.72,1.09)	0.84 (0.68,1.03)	0.95 (0.76,1.19)	1.12 (0.93,1.35)	0.84 (0.73,0.95)	0.84 (0.67,1.04)	0.94 (0.75,1.18)	1.08 (0.89,1.30)	0.88 (0.60,1.27)	**Paro**					

1.44 (1.02,2.04)	1.50 (1.07,2.07)	1.65 (1.05,2.60)	1.27 (0.92,1.75)	1.20 (0.84,1.70)	1.36 (0.95,1.95)	1.60 (1.14,2.23)	1.20 (0.88,1.62)	1.20 (0.83,1.71)	1.35 (0.92,1.95)	1.54 (1.09,2.17)	1.25 (0.77,2.01)	1.43 (1.05,1.94)	**Rebo**					
1.07 (0.85,1.37)	1.11 (0.92,1.35)	1.23 (0.85,1.79)	0.95 (0.76,1.18)	0.90 (0.71,1.13)	1.02 (0.79,1.32)	1.20 (0.97,1.48)	0.89 (0.76,1.05)	0.89 (0.70,1.13)	1.00 (0.77,1.30)	1.15 (0.93,1.43)	0.93 (0.63,1.37)	1.07 (0.90,1.26)	0.75 (0.54,1.04)	**Sert**				
1.36 (0.99,1.87)	1.41 (1.06,1.86)	1.56 (1.04,2.31)	1.20 (0.88,1.63)	1.13 (0.83,1.54)	1.28 (0.92,1.79)	1.51 (1.12,2.04)	1.13 (0.87,1.46)	1.13 (0.82,1.55)	1.27 (0.91,1.76)	1.45 (1.09,1.94)	1.18 (0.75,1.84)	1.35 (1.04,1.75)	0.94 (0.64,1.39)	1.26 (0.95,1.67)	**Traz**			
1.01 (0.82,1.26)	1.05 (0.87,1.27)	1.16 (0.82,1.65)	0.90 (0.72,1.10)	0.85 (0.67,1.06)	0.96 (0.77,1.21)	1.13 (0.93,1.37)	0.84 (0.73,0.97)	0.84 (0.66,1.07)	0.95 (0.73,1.23)	1.09 (0.89,1.33)	0.88 (0.59,1.30)	1.01 (0.86,1.17)	0.70 (0.51,0.97)	0.94 (0.78,1.13)	0.75 (0.57,0.98)	**Venl**		
0.73 (0.42,1.26)	0.76 (0.44,1.29)	0.83 (0.45,1.54)	0.64 (0.37,1.11)	0.61 (0.35,1.05)	0.69 (0.40,1.20)	0.81 (0.47,1.39)	0.60 (0.36,1.02)	0.60 (0.34,1.05)	0.68 (0.39,1.20)	0.78 (0.45,1.34)	0.63 (0.33,1.19)	0.72 (0.43,1.22)	0.51 (0.28,0.92)	0.68 (0.39,1.16)	0.54 (0.30,0.95)	0.72 (0.43,1.19)	**Vort**

OR>1 favour the treatment in the column

8.1.2 Dropouts for any reason

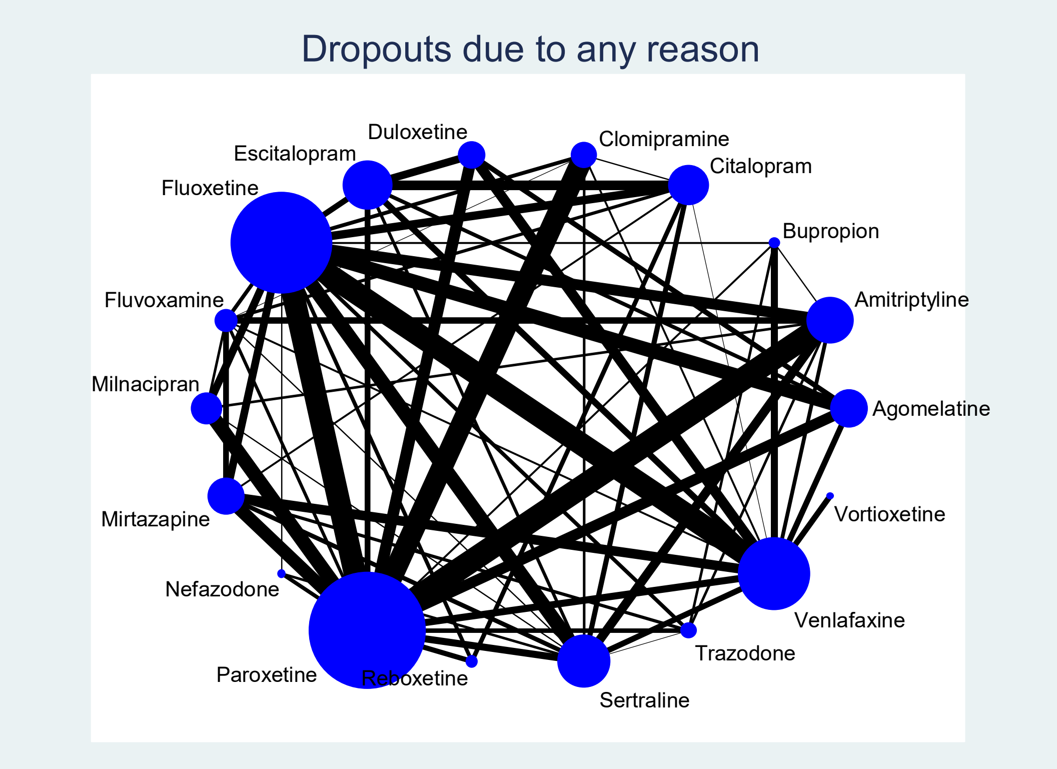

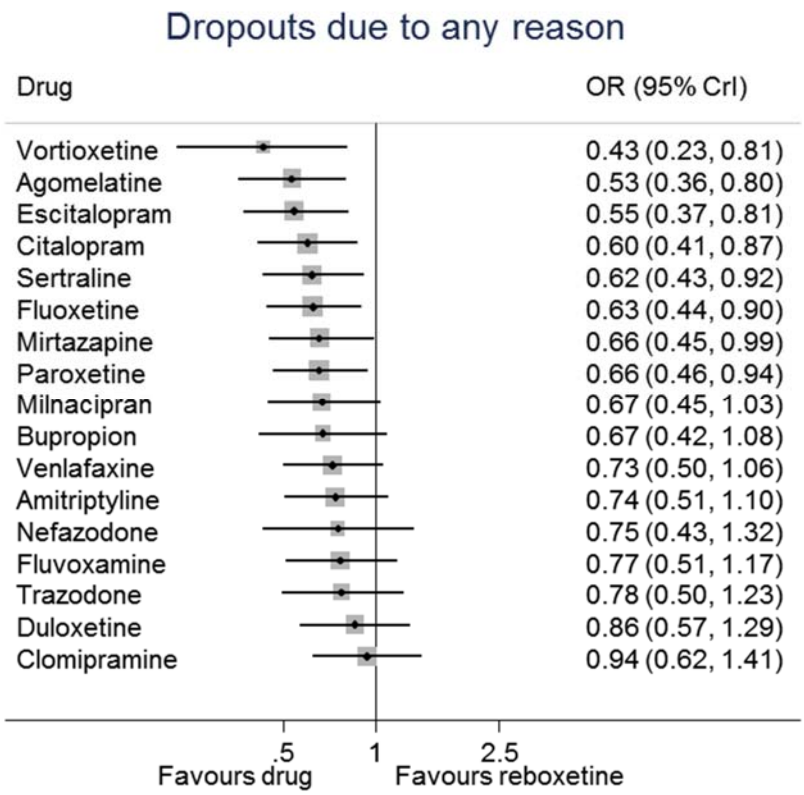
Dropouts due to any reason
Drug
OR (95% CrI)
Vortioxetine 0.43 (0.23, 0.81)
Agomelatine 0.53 (0.36, 0.80)
Escitalopram 0.55 (0.37, 0.81)
Citalopram 0.60 (0.41, 0.87)
Sertraline 0.62 (0.43, 0.92)
Fluoxetine 0.63 (0.44, 0.90)
Mirtazapine 0.66 (0.45, 0.99)
Paroxetine 0.66 (0.46, 0.94)
Milnacipran 0.67 (0.45, 1.03)
Bupropion 0.67 (0.42, 1.08)
Venlafaxine 0.73 (0.50, 1.06)
Amitriptyline 0.74 (0.51, 1.10)
Nefazodone 0.75 (0.43, 1.32)
Fluvoxamine 0.77 (0.51, 1.17)
Trazodone 0.78 (0.50, 1.23)
Duloxetine 0.86 (0.57, 1.29)
Clomipramine 0.94 (0.62, 1.41)
.5
1
2.5
Favours drug
Favours reboxetine

**Agom**																	
0.72 (0.55,0.92)	**Amit**																
0.80 (0.54,1.15)	1.10 (0.78,1.58)	**Bupr**															
0.89 (0.66,1.19)	1.23 (0.94,1.64)	1.11 (0.76,1.67)	**Cita**														
0.57 (0.42,0.77)	0.79 (0.60,1.05)	0.71 (0.49,1.07)	0.64 (0.47,0.87)	**Clom**													
0.62 (0.47,0.82)	0.87 (0.66,1.15)	0.78 (0.53,1.18)	0.70 (0.51,0.95)	1.10 (0.80,1.51)	**Dulo**												
0.97 (0.74,1.27)	1.35 (1.05,1.74)	1.23 (0.84,1.80)	1.09 (0.85,1.42)	1.71 (1.27,2.29)	1.56 (1.19,2.01)	**Esci**											
0.85 (0.68,1.05)	1.18 (0.99,1.42)	1.07 (0.76,1.50)	0.96 (0.76,1.21)	1.49 (1.16,1.90)	1.37 (1.06,1.73)	0.87 (0.70,1.09)	**Fluo**										
0.69 (0.51,0.97)	0.97 (0.74,1.24)	0.87 (0.59,1.30)	0.78 (0.57,1.06)	1.22 (0.88,1.67)	1.12 (0.80,1.53)	0.71 (0.53,0.96)	0.82 (0.64,1.04)	**Fluv**									
0.79 (0.58,1.09)	1.10 (0.84,1.45)	1.00 (0.66,1.49)	0.89 (0.64,1.23)	1.40 (1.00,1.92)	1.28 (0.91,1.75)	0.81 (0.60,1.11)	0.94 (0.72,1.20)	1.14 (0.84,1.56)	**Miln**								
0.81 (0.61,1.05)	1.12 (0.89,1.42)	1.01 (0.70,1.47)	0.91 (0.68,1.21)	1.41 (1.05,1.91)	1.30 (0.96,1.72)	0.83 (0.63,1.08)	0.95 (0.77,1.16)	1.16 (0.89,1.52)	1.02 (0.75,1.37)	**Mirt**							
0.70 (0.44,1.14)	0.98 (0.62,1.55)	0.89 (0.51,1.54)	0.79 (0.49,1.32)	1.24 (0.76,2.00)	1.13 (0.69,1.83)	0.72 (0.45,1.18)	0.83 (0.54,1.30)	1.01 (0.62,1.71)	0.88 (0.54,1.44)	0.87 (0.55,1.41)	**Nefa**						
0.81 (0.65,1.00)	1.12 (0.95,1.34)	1.02 (0.73,1.43)	0.91 (0.71,1.17)	1.42 (1.12,1.79)	1.30 (1.02,1.63)	0.83 (0.67,1.03)	0.95 (0.83,1.09)	1.16 (0.90,1.49)	1.02 (0.80,1.31)	1.00 (0.82,1.23)	1.15 (0.74,1.78)	**Paro**					
0.53 (0.36,0.80)	0.74 (0.51,1.10)	0.67 (0.42,1.08)	0.60 (0.41,0.87)	0.94 (0.62,1.41)	0.86 (0.57,1.29)	0.55 (0.37,0.81)	0.63 (0.44,0.90)	0.77 (0.51,1.17)	0.67 (0.45,1.03)	0.66 (0.45,0.99)	0.75 (0.43,1.32)	0.66 (0.46,0.94)	**Rebo**				
0.86 (0.66,1.13)	1.20 (0.97,1.47)	1.08 (0.75,1.56)	0.97 (0.74,1.25)	1.51 (1.15,1.96)	1.38 (1.04,1.80)	0.88 (0.69,1.12)	1.01 (0.84,1.21)	1.23 (0.94,1.63)	1.08 (0.82,1.44)	1.06 (0.84,1.35)	1.23 (0.77,1.90)	1.06 (0.88,1.28)	1.61 (1.09,2.34)	**Sert**			
0.69 (0.48,0.98)	0.96 (0.70,1.31)	0.87 (0.57,1.30)	0.77 (0.53,1.13)	1.21 (0.83,1.73)	1.10 (0.76,1.59)	0.70 (0.49,1.00)	0.81 (0.60,1.09)	0.99 (0.69,1.42)	0.86 (0.60,1.25)	0.85 (0.62,1.18)	0.98 (0.57,1.64)	0.85 (0.63,1.15)	1.29 (0.81,2.01)	0.80 (0.58,1.11)	**Traz**		
0.74 (0.58,0.92)	1.02 (0.83,1.26)	0.92 (0.66,1.30)	0.83 (0.64,1.07)	1.29 (0.99,1.67)	1.18 (0.92,1.49)	0.75 (0.60,0.94)	0.87 (0.74,1.01)	1.06 (0.80,1.38)	0.93 (0.71,1.22)	0.91 (0.73,1.13)	1.04 (0.66,1.65)	0.91 (0.77,1.07)	1.38 (0.94,1.99)	0.86 (0.70,1.05)	1.07 (0.77,1.47)	**Venl**	
1.24 (0.71,2.19)	1.72 (1.00,3.05)	1.55 (0.85,2.94)	1.40 (0.78,2.48)	2.20 (1.22,3.90)	1.99 (1.13,3.52)	1.27 (0.73,2.25)	1.46 (0.85,2.53)	1.78 (1.00,3.24)	1.56 (0.89,2.84)	1.53 (0.89,2.72)	1.76 (0.90,3.56)	1.53 (0.90,2.66)	2.32 (1.24,4.41)	1.45 (0.84,2.54)	1.80 (0.98,3.38)	1.69 (1.01,2.86)	**Vort**

OR<1 favour the treatment in the column

8.1.3 Efficacy continuous

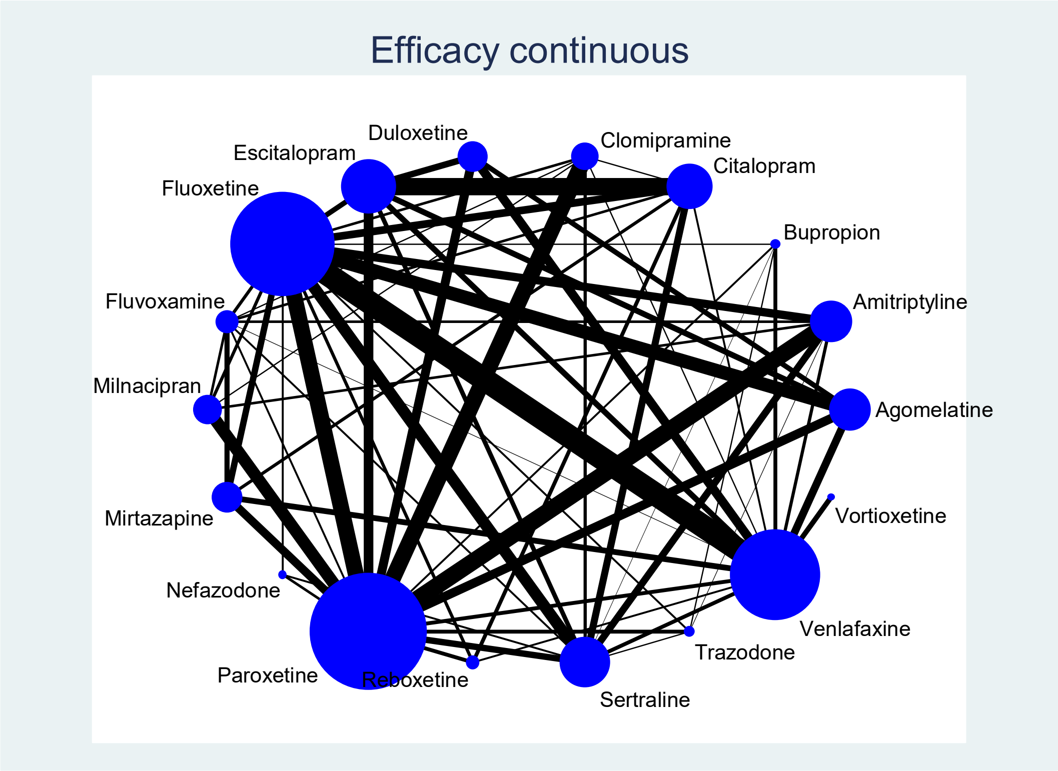

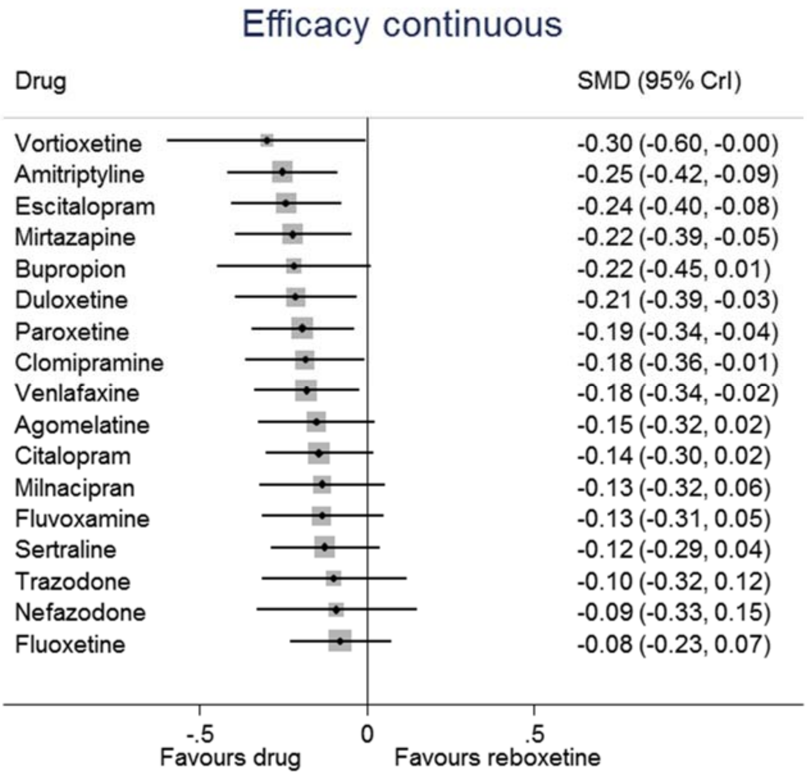

**Agom**																	
0.10 (-0.02,0.22)	**Amit**																
0.07 (-0.13,0.26)	-0.04 (-0.22,0.16)	**Bupr**															
-0.01 (-0.13,0.11)	-0.11 (-0.23,0.01)	-0.08 (-0.27,0.12)	**Cita**														
0.03 (-0.11,0.17)	-0.07 (-0.19,0.06)	-0.03 (-0.24,0.17)	0.04 (-0.09,0.18)	**Clom**													
0.06 (-0.07,0.19)	-0.04 (-0.17,0.09)	-0.00 (-0.21,0.20)	0.07 (-0.06,0.21)	0.03 (-0.12,0.18)	**Dulo**												
0.09 (-0.02,0.20)	-0.01 (-0.12,0.10)	0.02 (-0.17,0.21)	0.10 (0.01,0.19)	0.06 (-0.08,0.19)	0.03 (-0.09,0.15)	**Esci**											
-0.07 (-0.17,0.03)	-0.17 (-0.26,-0.09)	-0.14 (-0.32,0.04)	-0.06 (-0.16,0.03)	-0.10 (-0.22,0.01)	-0.13 (-0.25,-0.02)	-0.16 (-0.25,-0.07)	**Fluo**										
-0.02 (-0.16,0.12)	-0.12 (-0.25,0.01)	-0.08 (-0.29,0.12)	-0.01 (-0.14,0.12)	-0.05 (-0.20,0.09)	-0.08 (-0.23,0.07)	-0.11 (-0.24,0.02)	0.05 (-0.06,0.17)	**Fluv**									
-0.02 (-0.17,0.13)	-0.12 (-0.25,0.02)	-0.08 (-0.30,0.13)	-0.01 (-0.16,0.14)	-0.05 (-0.20,0.10)	-0.08 (-0.24,0.08)	-0.11 (-0.25,0.04)	0.05 (-0.07,0.18)	0.00 (-0.15,0.15)	**Miln**								
0.07 (-0.06,0.20)	-0.03 (-0.16,0.09)	0.00 (-0.20,0.20)	0.08 (-0.04,0.20)	0.04 (-0.11,0.18)	0.01 (-0.14,0.15)	-0.02 (-0.14,0.10)	0.14 (0.04,0.25)	0.09 (-0.04,0.22)	0.09 (-0.06,0.24)	**Mirt**							
-0.06 (-0.27,0.15)	-0.16 (-0.36,0.04)	-0.13 (-0.38,0.13)	-0.05 (-0.26,0.16)	-0.09 (-0.31,0.12)	-0.12 (-0.34,0.09)	-0.15 (-0.36,0.05)	0.01 (-0.18,0.20)	-0.04 (-0.26,0.17)	-0.04 (-0.26,0.18)	-0.13 (-0.34,0.08)	**Nefa**						
0.04 (-0.06,0.14)	-0.06 (-0.14,0.02)	-0.02 (-0.20,0.15)	0.05 (-0.05,0.15)	0.01 (-0.10,0.12)	-0.02 (-0.13,0.09)	-0.05 (-0.14,0.04)	0.11 (0.05,0.18)	0.06 (-0.06,0.17)	0.06 (-0.06,0.18)	-0.03 (-0.13,0.08)	0.10 (-0.09,0.29)	**Paro**					
-0.15 (-0.32,0.02)	-0.25 (-0.42,-0.09)	-0.22 (-0.45,0.01)	-0.14 (-0.30,0.02)	-0.18 (-0.36,-0.01)	-0.21 (-0.39,-0.03)	-0.24 (-0.40,-0.08)	-0.08 (-0.23,0.07)	-0.13 (-0.31,0.05)	-0.13 (-0.32,0.06)	-0.22 (-0.39,-0.05)	-0.09 (-0.33,0.15)	-0.19 (-0.34,-0.04)	**Rebo**				
-0.03 (-0.15,0.09)	-0.13 (-0.23,-0.03)	-0.09 (-0.28,0.10)	-0.02 (-0.12,0.09)	-0.06 (-0.18,0.06)	-0.09 (-0.22,0.04)	-0.12 (-0.22,-0.01)	0.05 (-0.04,0.13)	-0.01 (-0.13,0.12)	-0.01 (-0.15,0.13)	-0.10 (-0.22,0.03)	0.03 (-0.16,0.23)	-0.07 (-0.15,0.02)	0.12 (-0.04,0.29)	**Sert**			
-0.05 (-0.24,0.13)	-0.15 (-0.33,0.02)	-0.12 (-0.33,0.10)	-0.04 (-0.22,0.14)	-0.08 (-0.28,0.10)	-0.11 (-0.31,0.08)	-0.14 (-0.32,0.04)	0.02 (-0.15,0.19)	-0.03 (-0.22,0.16)	-0.03 (-0.23,0.16)	-0.12 (-0.31,0.07)	0.01 (-0.24,0.25)	-0.09 (-0.26,0.07)	0.10 (-0.12,0.32)	-0.03 (-0.20,0.15)	**Traz**		
0.03 (-0.08,0.13)	-0.07 (-0.17,0.03)	-0.04 (-0.21,0.14)	0.04 (-0.06,0.14)	-0.00 (-0.13,0.12)	-0.03 (-0.15,0.08)	-0.06 (-0.15,0.04)	0.10 (0.03,0.18)	0.05 (-0.07,0.17)	0.05 (-0.09,0.18)	-0.04 (-0.15,0.07)	0.09 (-0.11,0.29)	-0.01 (-0.09,0.07)	0.18 (0.02,0.34)	0.06 (-0.04,0.15)	0.08 (-0.09,0.25)	**Venl**	
0.15 (-0.13,0.43)	0.05 (-0.23,0.32)	0.08 (-0.23,0.40)	0.16 (-0.12,0.44)	0.12 (-0.17,0.40)	0.08 (-0.19,0.37)	0.06 (-0.21,0.33)	0.22 (-0.04,0.49)	0.17 (-0.12,0.45)	0.17 (-0.12,0.46)	0.08 (-0.20,0.36)	0.21 (-0.11,0.54)	0.11 (-0.16,0.38)	0.30 (0.00,0.60)	0.17 (-0.10,0.45)	0.20 (-0.11,0.51)	0.12 (-0.14,0.38)	**Vort**

SMD<0 favours the treatment in the column

8.1.4 Remission

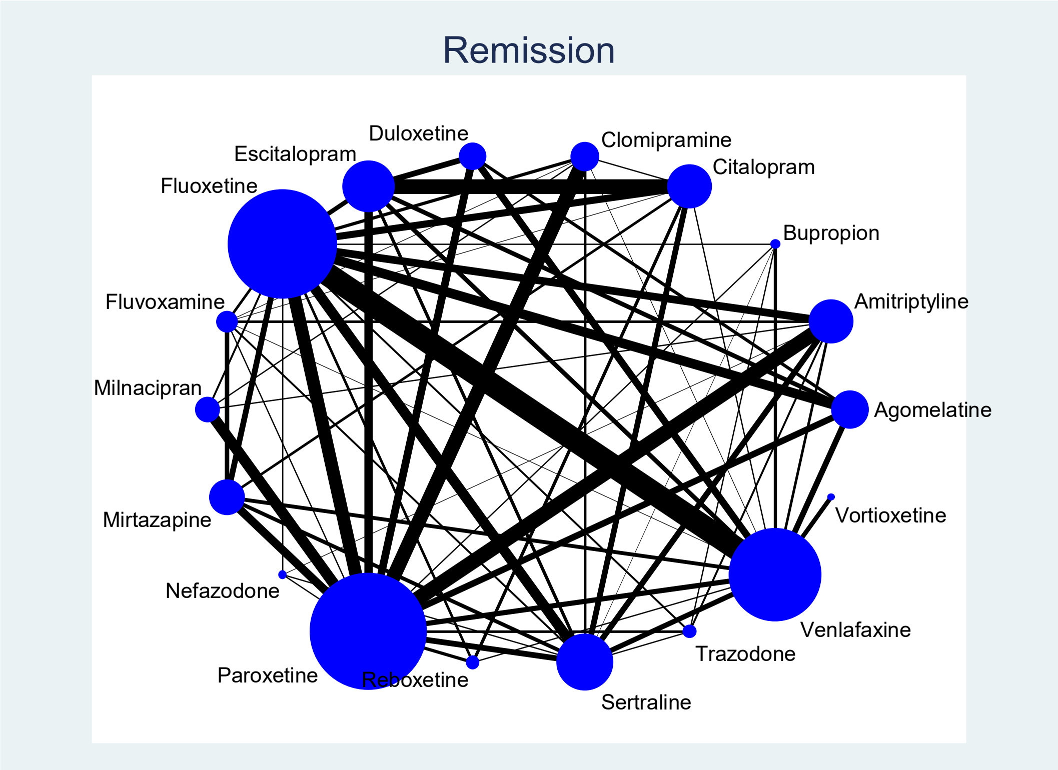

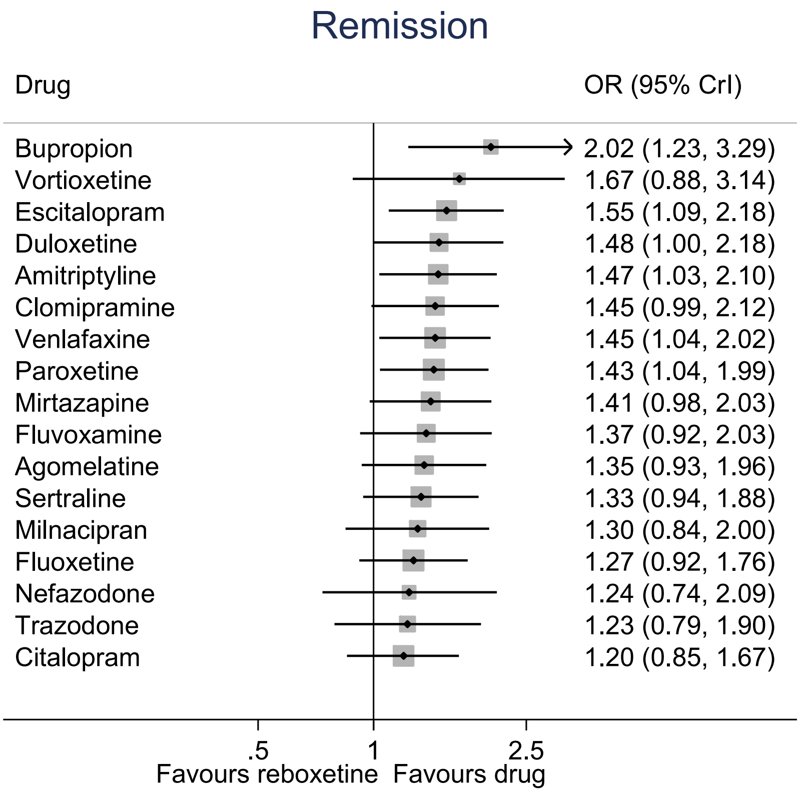

**Agom**																	
0.92 (0.71,1.19)	**Amit**																
0.67 (0.44,1.03)	0.73 (0.48,1.10)	**Bupr**															
1.13 (0.87,1.48)	1.23 (0.97,1.57)	1.69 (1.11,2.57)	**Cita**														
0.94 (0.70,1.26)	1.02 (0.78,1.32)	1.40 (0.90,2.17)	0.83 (0.63,1.09)	**Clom**													
0.91 (0.69,1.21)	0.99 (0.75,1.32)	1.36 (0.87,2.12)	0.81 (0.61,1.07)	0.98 (0.71,1.34)	**Dulo**												
0.87 (0.69,1.12)	0.95 (0.76,1.20)	1.30 (0.86,1.97)	0.77 (0.64,0.94)	0.93 (0.71,1.23)	0.96 (0.74,1.24)	**Esci**											
1.06 (0.86,1.33)	1.16 (0.97,1.38)	1.59 (1.08,2.35)	0.94 (0.77,1.15)	1.14 (0.90,1.44)	1.16 (0.91,1.50)	1.22 (1.01,1.47)	**Fluo**										
0.99 (0.72,1.36)	1.08 (0.82,1.41)	1.47 (0.94,2.31)	0.87 (0.65,1.17)	1.06 (0.77,1.44)	1.08 (0.77,1.51)	1.13 (0.84,1.51)	0.93 (0.72,1.19)	**Fluv**									
1.04 (0.73,1.49)	1.13 (0.83,1.56)	1.55 (0.96,2.51)	0.92 (0.65,1.30)	1.11 (0.79,1.57)	1.14 (0.79,1.65)	1.19 (0.85,1.67)	0.98 (0.72,1.32)	1.05 (0.73,1.54)	**Miln**								
0.96 (0.73,1.27)	1.05 (0.82,1.33)	1.43 (0.94,2.19)	0.85 (0.66,1.09)	1.03 (0.77,1.36)	1.05 (0.78,1.42)	1.10 (0.86,1.41)	0.90 (0.73,1.11)	0.97 (0.74,1.28)	0.92 (0.65,1.31)	**Mirt**							
1.09 (0.68,1.74)	1.19 (0.76,1.85)	1.63 (0.92,2.87)	0.97 (0.61,1.53)	1.17 (0.72,1.88)	1.20 (0.74,1.94)	1.25 (0.79,1.97)	1.03 (0.67,1.57)	1.11 (0.68,1.78)	1.05 (0.63,1.74)	1.14 (0.72,1.81)	**Nefa**						
0.94 (0.76,1.18)	1.03 (0.86,1.22)	1.41 (0.95,2.08)	0.83 (0.68,1.03)	1.01 (0.81,1.26)	1.03 (0.81,1.31)	1.08 (0.89,1.31)	0.89 (0.77,1.02)	0.95 (0.74,1.23)	0.91 (0.68,1.21)	0.98 (0.80,1.21)	0.86 (0.56,1.33)	**Paro**					
1.35 (0.93,1.96)	1.47 (1.03,2.10)	2.02 (1.23,3.29)	1.20 (0.85,1.67)	1.45 (0.99,2.12)	1.48 (1.00,2.18)	1.55 (1.09,2.18)	1.27 (0.92,1.76)	1.37 (0.92,2.03)	1.30 (0.84,2.00)	1.41 (0.98,2.03)	1.24 (0.74,2.09)	1.43 (1.04,1.99)	**Rebo**				
1.02 (0.79,1.32)	1.11 (0.91,1.36)	1.52 (1.01,2.28)	0.90 (0.72,1.12)	1.09 (0.84,1.40)	1.11 (0.85,1.47)	1.17 (0.94,1.44)	0.96 (0.81,1.13)	1.03 (0.79,1.35)	0.98 (0.70,1.36)	1.06 (0.84,1.34)	0.93 (0.61,1.44)	1.08 (0.90,1.29)	0.75 (0.53,1.07)	**Sert**			
1.10 (0.77,1.59)	1.20 (0.87,1.65)	1.64 (1.05,2.58)	0.98 (0.68,1.39)	1.18 (0.81,1.71)	1.21 (0.82,1.77)	1.26 (0.89,1.79)	1.04 (0.76,1.41)	1.12 (0.76,1.64)	1.06 (0.70,1.60)	1.15 (0.80,1.64)	1.01 (0.61,1.70)	1.17 (0.86,1.60)	0.82 (0.53,1.26)	1.08 (0.78,1.50)	**Traz**		
0.94 (0.75,1.17)	1.02 (0.83,1.25)	1.40 (0.95,2.06)	0.83 (0.67,1.03)	1.00 (0.78,1.29)	1.03 (0.80,1.31)	1.07 (0.88,1.31)	0.88 (0.75,1.02)	0.95 (0.72,1.24)	0.90 (0.65,1.24)	0.98 (0.78,1.22)	0.86 (0.55,1.33)	0.99 (0.84,1.17)	0.69 (0.50,0.97)	0.92 (0.76,1.11)	0.85 (0.61,1.18)	**Venl**	
0.81 (0.45,1.45)	0.88 (0.50,1.56)	1.21 (0.62,2.33)	0.72 (0.40,1.28)	0.87 (0.48,1.56)	0.89 (0.49,1.59)	0.93 (0.52,1.64)	0.76 (0.43,1.32)	0.82 (0.45,1.48)	0.78 (0.41,1.44)	0.84 (0.47,1.50)	0.74 (0.37,1.48)	0.86 (0.49,1.50)	0.60 (0.32,1.13)	0.80 (0.45,1.40)	0.73 (0.39,1.37)	0.87 (0.51,1.47)	**Vort**

OR>1 favours the treatment in the column

8.1.5 Dropouts due to adverse events

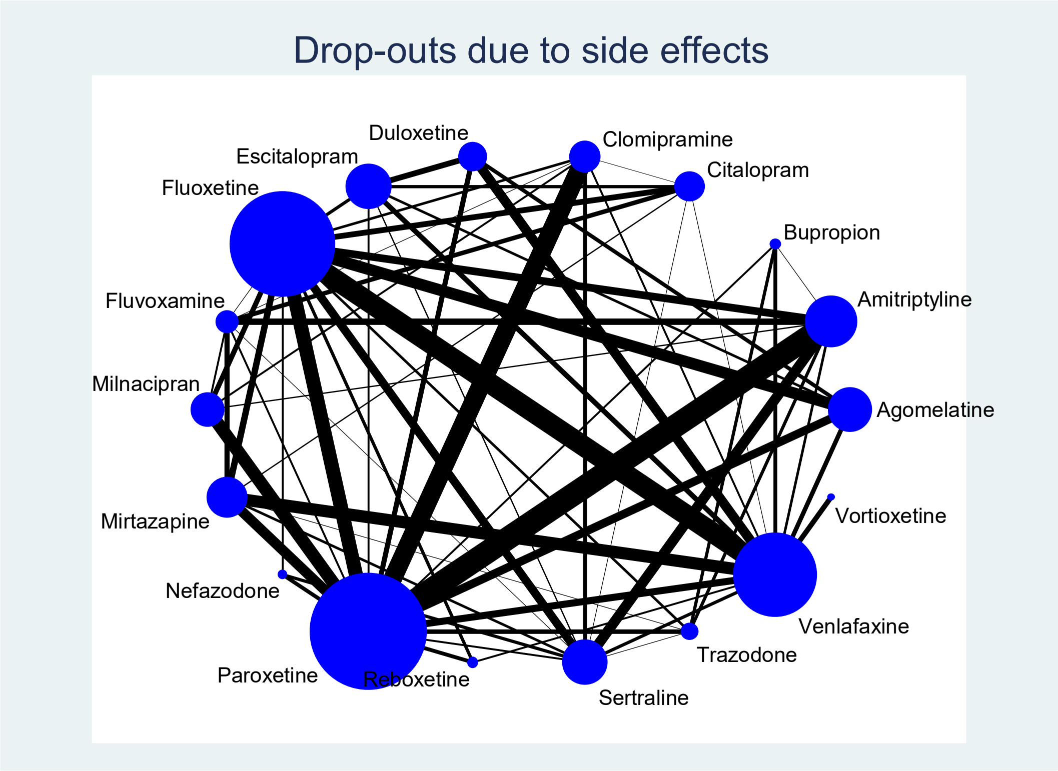

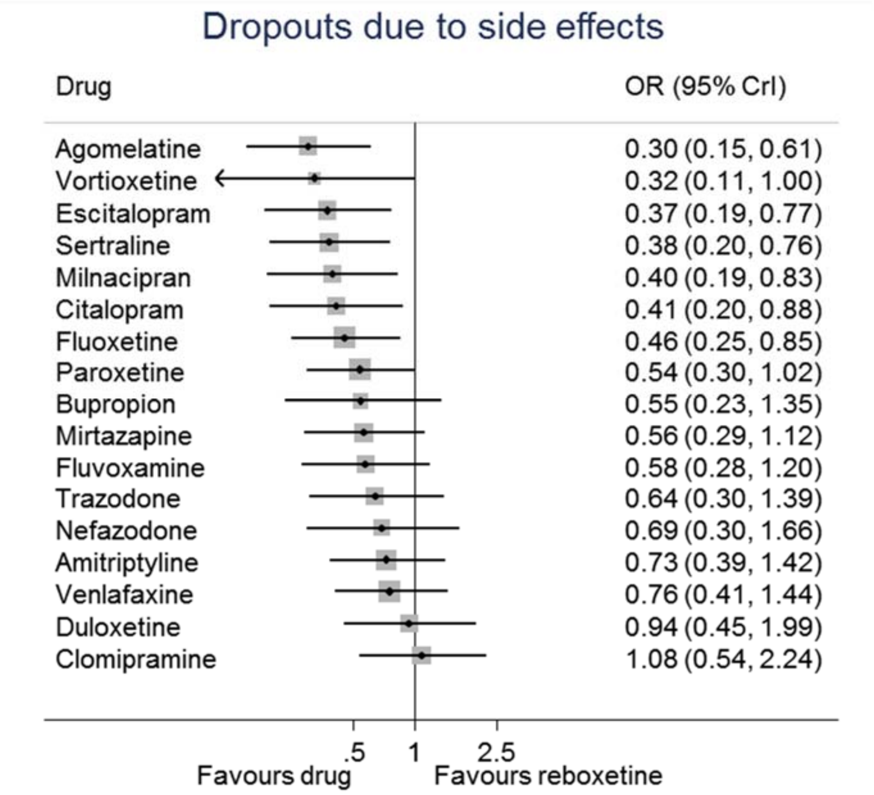

**Agom**																	
0.41 (0.26,0.64)	**Amit**																
0.55 (0.26,1.13)	1.33 (0.68,2.61)	**Bupr**															
0.73 (0.41,1.28)	1.76 (1.06,2.96)	1.32 (0.61,2.89)	**Cita**														
0.28 (0.16,0.47)	0.67 (0.44,1.04)	0.51 (0.24,1.05)	0.38 (0.21,0.68)	**Clom**													
0.32 (0.19,0.53)	0.78 (0.47,1.29)	0.58 (0.27,1.26)	0.44 (0.24,0.80)	1.15 (0.66,2.04)	**Dulo**												
0.81 (0.50,1.30)	1.95 (1.23,3.13)	1.46 (0.70,3.10)	1.11 (0.66,1.88)	2.89 (1.70,5.00)	2.51 (1.55,4.10)	**Esci**											
0.66 (0.44,0.97)	1.60 (1.19,2.16)	1.20 (0.62,2.34)	0.91 (0.57,1.44)	2.38 (1.58,3.59)	2.06 (1.30,3.28)	0.82 (0.54,1.24)	**Fluo**										
0.53 (0.30,0.89)	1.27 (0.83,1.94)	0.95 (0.45,2.00)	0.72 (0.42,1.23)	1.89 (1.11,3.20)	1.63 (0.91,2.94)	0.65 (0.38,1.12)	0.79 (0.52,1.21)	**Fluv**									
0.76 (0.44,1.34)	1.84 (1.16,3.02)	1.38 (0.65,3.02)	1.05 (0.57,1.94)	2.73 (1.63,4.73)	2.37 (1.30,4.41)	0.94 (0.53,1.68)	1.15 (0.74,1.83)	1.45 (0.85,2.53)	**Miln**								
0.54 (0.33,0.86)	1.29 (0.88,1.92)	0.97 (0.48,1.97)	0.73 (0.43,1.23)	1.92 (1.20,3.10)	1.66 (0.98,2.83)	0.66 (0.40,1.08)	0.81 (0.57,1.14)	1.02 (0.65,1.60)	0.70 (0.41,1.17)	**Mirt**							
0.44 (0.21,0.89)	1.05 (0.55,2.04)	0.79 (0.33,1.91)	0.60 (0.28,1.26)	1.57 (0.77,3.17)	1.36 (0.64,2.88)	0.54 (0.26,1.12)	0.66 (0.35,1.23)	0.83 (0.40,1.71)	0.57 (0.27,1.19)	0.82 (0.41,1.61)	**Nefa**						
0.56 (0.37,0.83)	1.35 (1.03,1.78)	1.01 (0.53,1.95)	0.76 (0.47,1.24)	1.99 (1.39,2.94)	1.73 (1.10,2.76)	0.69 (0.45,1.05)	0.84 (0.65,1.09)	1.06 (0.70,1.62)	0.73 (0.47,1.11)	1.04 (0.74,1.48)	1.28 (0.69,2.39)	**Paro**					
0.30 (0.15,0.61)	0.73 (0.39,1.42)	0.55 (0.23,1.35)	0.41 (0.20,0.88)	1.08 (0.54,2.24)	0.94 (0.45,1.99)	0.37 (0.19,0.77)	0.46 (0.25,0.85)	0.58 (0.28,1.20)	0.40 (0.19,0.83)	0.56 (0.29,1.12)	0.69 (0.30,1.66)	0.54 (0.30,1.02)	**Rebo**				
0.79 (0.48,1.27)	1.91 (1.33,2.76)	1.43 (0.71,2.90)	1.08 (0.64,1.83)	2.83 (1.81,4.50)	2.46 (1.45,4.17)	0.98 (0.60,1.59)	1.19 (0.84,1.69)	1.50 (0.94,2.43)	1.04 (0.61,1.74)	1.48 (0.97,2.25)	1.81 (0.95,3.46)	1.42 (1.00,2.01)	2.62 (1.32,5.10)	**Sert**			
0.47 (0.26,0.85)	1.14 (0.69,1.89)	0.85 (0.43,1.71)	0.65 (0.34,1.23)	1.69 (0.93,3.06)	1.46 (0.77,2.77)	0.58 (0.32,1.06)	0.71 (0.43,1.16)	0.89 (0.49,1.63)	0.62 (0.33,1.15)	0.88 (0.50,1.52)	1.08 (0.50,2.33)	0.85 (0.52,1.37)	1.56 (0.72,3.28)	0.60 (0.34,1.03)	**Traz**		
0.40 (0.26,0.59)	0.97 (0.69,1.36)	0.72 (0.38,1.39)	0.55 (0.34,0.89)	1.43 (0.93,2.22)	1.24 (0.80,1.93)	0.50 (0.33,0.74)	0.60 (0.46,0.79)	0.76 (0.48,1.20)	0.52 (0.32,0.85)	0.75 (0.52,1.07)	0.91 (0.48,1.77)	0.72 (0.53,0.96)	1.32 (0.69,2.46)	0.51 (0.35,0.73)	0.85 (0.50,1.43)	**Ven**	
0.93 (0.34,2.56)	2.25 (0.86,6.11)	1.69 (0.56,5.24)	1.28 (0.45,3.61)	3.34 (1.22,9.36)	2.90 (1.06,8.13)	1.15 (0.42,3.22)	1.40 (0.55,3.71)	1.77 (0.64,4.99)	1.22 (0.43,3.47)	1.74 (0.66,4.72)	2.13 (0.70,6.67)	1.67 (0.64,4.43)	3.09 (1.00,9.37)	1.18 (0.44,3.20)	1.98 (0.69,5.74)	2.32 (0.94,5.94)	**Vort**

Values<1 favour the treatment in the column

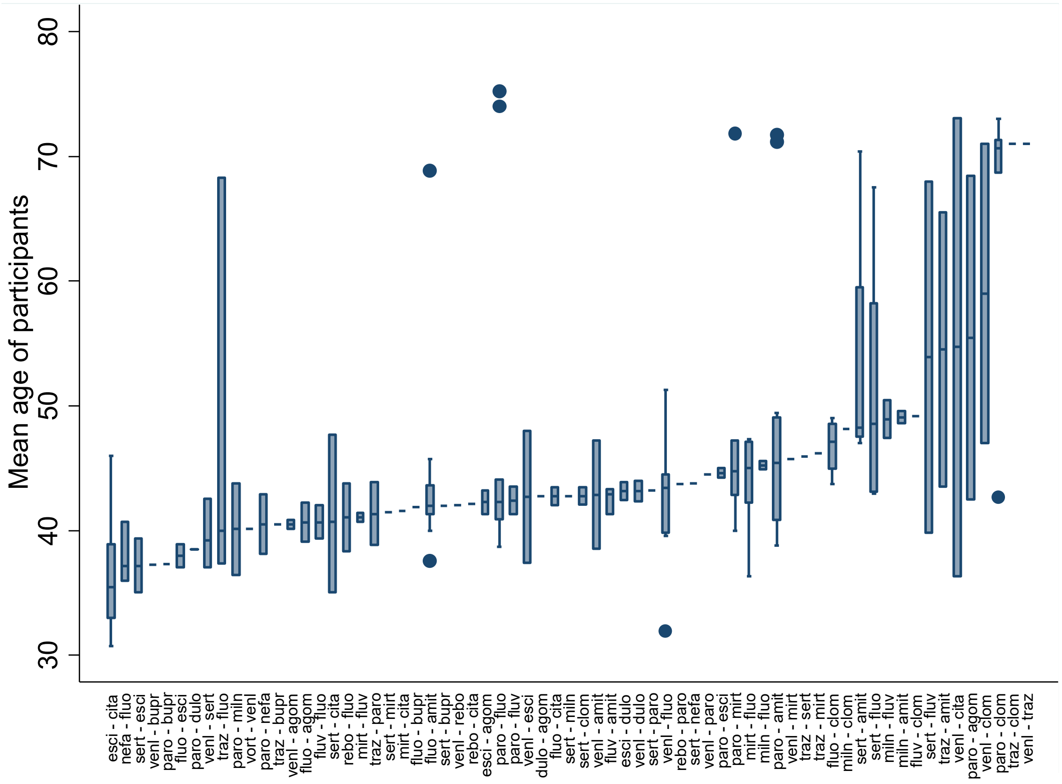
Mean age of participants

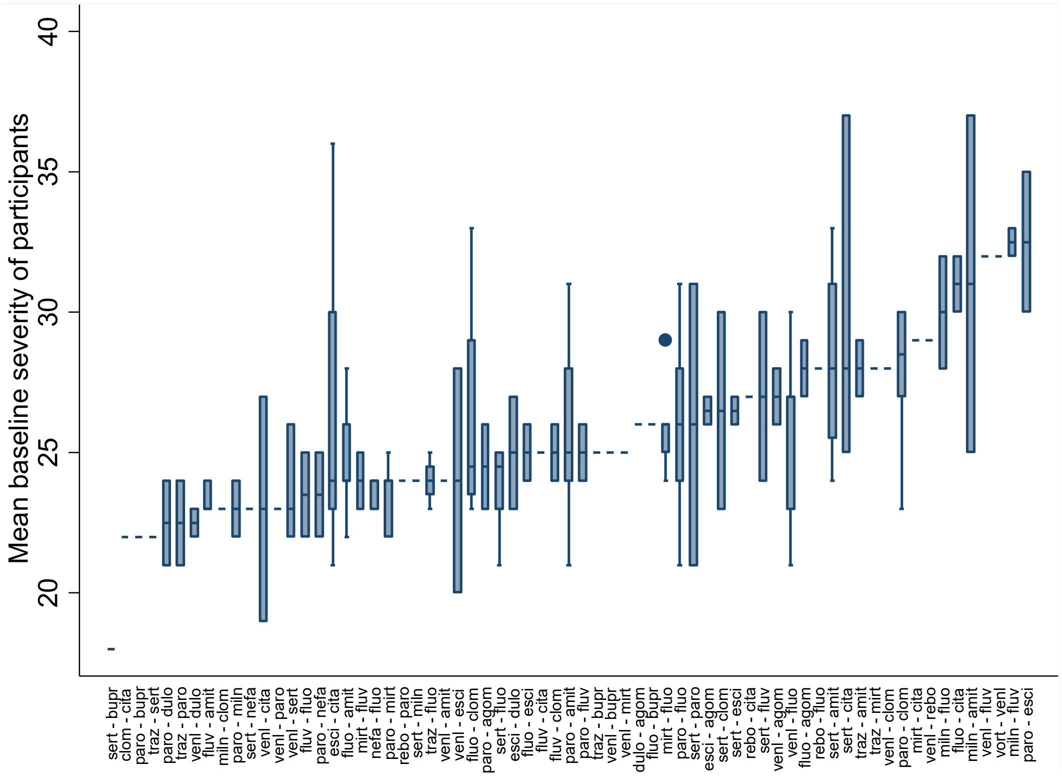
Mean baseline severity of participants

Examination of possible effect modifiers indicated that age was evenly distributed across comparisons but not baseline severity. However, we could not detenct any important impact of the baseline severity in the previous meta-regressions.

8.3 Inconsistency

8.3.1 Response

8.3.1.1 Loop-specific approach

Loops With Evidence of Statistical Inconsistency:

Loop	IF	seIF	z_value	p_value	CI_95	Loop_Heterog_tau2
cita-fluv-venl	1.243	0.599	2.077	0.038	(0.07,2.42)	0.000
clom-fluo-traz	1.161	0.524	2.215	0.027	(0.13,2.19)	0.000
fluv-sert-venl	1.133	0.554	2.046	0.041	(0.05,2.22)	0.000
cita-clom-fluo	1.031	0.470	2.192	0.028	(0.11,1.95)	0.000
agom-paro-venl	0.927	0.387	2.396	0.017	(0.17,1.69)	0.028
agom-dulo-esci	0.844	0.348	2.424	0.015	(0.16,1.53)	0.000
mirt-paro-venl	0.843	0.420	2.007	0.045	(0.02,1.67)	0.054
clom-fluo-sert	0.801	0.356	2.249	0.025	(0.10,1.50)	0.000
agom-dulo-venl	0.690	0.319	2.160	0.031	(0.06,1.32)	0.000
clom-fluo-paro	0.645	0.307	2.100	0.036	(0.04,1.25)	0.019
amit-fluo-sert	0.533	0.246	2.164	0.030	(0.05,1.02)	0.000

Loops Without Evidence of Statistical Inconsistency:

Loop	IF	seIF	z_value	p_value	CI_95	Loop_Heterog_tau2
cita-clom-venl	1.891	1.255	1.507	0.132	(0.00,4.35)	0.492
bupr-sert-traz	1.385	1.151	1.203	0.229	(0.00,3.64)	0.000
fluo-miln-sert	1.242	0.964	1.288	0.198	(0.00,3.13)	0.019
clom-fluo-fluv	1.149	0.613	1.873	0.061	(0.00,2.35)	0.000
cita-sert-venl	1.118	0.892	1.253	0.210	(0.00,2.87)	0.284
clom-fluv-sert	1.102	0.650	1.695	0.090	(0.00,2.38)	0.000
fluv-mirt-venl	1.037	0.749	1.385	0.166	(0.00,2.50)	0.126
amit-fluv-venl	0.933	0.985	0.948	0.343	(0.00,2.86)	0.237
cita-clom-mirt-paro	0.899	0.826	1.088	0.277	(0.00,2.52)	0.105
miln-paro-sert	0.872	0.939	0.928	0.353	(0.00,2.71)	0.000
cita-clom-paro-rebo	0.842	0.934	0.901	0.367	(0.00,2.67)	0.137
cita-esci-venl	0.834	0.548	1.522	0.128	(0.00,1.91)	0.119
cita-fluo-venl	0.806	0.430	1.876	0.061	(0.00,1.65)	0.039
cita-clom-mirt-traz	0.775	0.711	1.089	0.276	(0.00,2.17)	0.000
fluo-fluv-sert	0.755	0.407	1.856	0.063	(0.00,1.55)	0.000
fluo-sert-traz	0.750	0.525	1.430	0.153	(0.00,1.78)	0.014
fluo-traz-venl	0.743	0.517	1.438	0.150	(0.00,1.76)	0.011
cita-fluv-sert	0.736	0.928	0.793	0.428	(0.00,2.55)	0.213
cita-fluo-mirt	0.735	0.462	1.591	0.112	(0.00,1.64)	0.014
bupr-fluo-traz	0.734	0.996	0.737	0.461	(0.00,2.69)	0.215
fluo-fluv-miln	0.719	0.432	1.665	0.096	(0.00,1.57)	0.010
fluo-fluv-venl	0.718	0.494	1.453	0.146	(0.00,1.69)	0.000
fluv-paro-sert	0.670	0.467	1.435	0.151	(0.00,1.58)	0.000
amit-sert-traz	0.666	0.554	1.201	0.230	(0.00,1.75)	0.024
amit-miln-sert	0.642	1.096	0.585	0.558	(0.00,2.79)	0.110
clom-miln-sert	0.641	1.026	0.624	0.532	(0.00,2.65)	0.000
amit-paro-venl	0.621	0.478	1.299	0.194	(0.00,1.56)	0.093
clom-fluv-paro	0.616	0.733	0.840	0.401	(0.00,2.05)	0.086
fluv-mirt-sert	0.615	0.799	0.769	0.442	(0.00,2.18)	0.225
esci-paro-venl	0.608	0.469	1.297	0.195	(0.00,1.53)	0.066
amit-traz-venl	0.594	0.566	1.050	0.294	(0.00,1.70)	0.000
amit-fluv-miln	0.587	0.881	0.666	0.505	(0.00,2.31)	0.308
bupr-paro-venl	0.582	0.447	1.303	0.193	(0.00,1.46)	0.000

cita-fluo-rebo	0.575	0.383	1.499	0.134	(0.00,1.33)	0.000
nefa-paro-sert	0.546	0.566	0.964	0.335	(0.00,1.65)	0.039
cita-esci-mirt-paro	0.538	0.523	1.029	0.304	(0.00,1.56)	0.047
esci-sert-venl	0.537	0.543	0.990	0.322	(0.00,1.60)	0.074
fluo-paro-traz	0.502	0.389	1.289	0.197	(0.00,1.26)	0.004
cita-mirt-sert	0.487	1.388	0.351	0.726	(0.00,3.21)	0.463
fluo-paro-venl	0.481	0.248	1.936	0.053	(0.00,0.97)	0.000
fluo-nefa-sert	0.470	0.464	1.013	0.311	(0.00,1.38)	0.000
cita-esci-sert	0.465	0.597	0.779	0.436	(0.00,1.64)	0.139
amit-fluo-traz	0.456	0.424	1.076	0.282	(0.00,1.29)	0.000
agom-esci-paro	0.450	0.307	1.467	0.142	(0.00,1.05)	0.000
cita-clom-sert	0.448	0.886	0.506	0.613	(0.00,2.18)	0.159
fluv-miln-paro	0.446	0.429	1.041	0.298	(0.00,1.29)	0.000
amit-clom-miln-traz	0.445	1.176	0.378	0.705	(0.00,2.75)	0.301
cita-esci-paro-rebo	0.435	0.466	0.932	0.351	(0.00,1.35)	0.031
clom-fluv-mirt-traz	0.407	1.157	0.352	0.725	(0.00,2.67)	0.265
bupr-sert-venl	0.391	1.057	0.370	0.712	(0.00,2.46)	0.000
esci-fluo-venl	0.386	0.303	1.272	0.203	(0.00,0.98)	0.000
amit-clom-miln-venl	0.383	0.841	0.455	0.649	(0.00,2.03)	0.122
cita-clom-esci-paro	0.377	0.584	0.645	0.519	(0.00,1.52)	0.069
agom-fluo-venl	0.361	0.251	1.440	0.150	(0.00,0.85)	0.001
cita-esci-fluo	0.358	0.316	1.134	0.257	(0.00,0.98)	0.012
esci-fluo-paro	0.356	0.280	1.272	0.203	(0.00,0.90)	0.000
amit-clom-fluv-traz	0.356	1.096	0.325	0.745	(0.00,2.50)	0.220
fluo-sert-venl	0.340	0.247	1.378	0.168	(0.00,0.82)	0.000
dulo-paro-venl	0.337	0.436	0.774	0.439	(0.00,1.19)	0.058
fluo-miln-paro	0.332	0.266	1.249	0.212	(0.00,0.85)	0.005
dulo-esci-paro	0.321	0.364	0.883	0.377	(0.00,1.03)	0.038
fluo-fluv-mirt	0.314	0.512	0.614	0.539	(0.00,1.32)	0.098
cita-fluv-mirt	0.306	0.508	0.602	0.547	(0.00,1.30)	0.000
amit-fluv-sert	0.303	0.572	0.529	0.597	(0.00,1.42)	0.098
amit-paro-sert	0.296	0.359	0.827	0.408	(0.00,1.00)	0.067
clom-sert-venl	0.293	0.429	0.684	0.494	(0.00,1.13)	0.000
amit-fluo-venl	0.287	0.315	0.911	0.363	(0.00,0.90)	0.000
esci-fluo-sert	0.278	0.368	0.757	0.449	(0.00,1.00)	0.000
cita-fluo-sert	0.275	0.365	0.754	0.451	(0.00,0.99)	0.029
amit-paro-traz	0.269	0.512	0.525	0.600	(0.00,1.27)	0.081
clom-fluv-venl	0.262	0.727	0.360	0.719	(0.00,1.69)	0.000
cita-esci-fluv-paro	0.255	0.517	0.493	0.622	(0.00,1.27)	0.022
amit-fluo-paro	0.247	0.231	1.068	0.286	(0.00,0.70)	0.020
fluv-miln-sert	0.243	1.002	0.243	0.808	(0.00,2.21)	0.000
fluo-rebo-venl	0.227	0.425	0.533	0.594	(0.00,1.06)	0.000
clom-fluo-miln	0.219	0.492	0.444	0.657	(0.00,1.18)	0.000
clom-fluv-miln	0.218	0.707	0.308	0.758	(0.00,1.60)	0.000
fluo-mirt-paro	0.210	0.279	0.751	0.453	(0.00,0.76)	0.023
bupr-paro-traz	0.185	0.558	0.332	0.740	(0.00,1.28)	0.000
cita-fluv-paro-rebo	0.181	0.526	0.345	0.730	(0.00,1.21)	0.000
cita-paro-rebo-sert	0.181	0.949	0.191	0.849	(0.00,2.04)	0.144
clom-paro-sert	0.180	0.483	0.373	0.709	(0.00,1.13)	0.069
bupr-traz-venl	0.175	0.592	0.296	0.767	(0.00,1.34)	0.000
cita-fluo-fluv	0.169	0.417	0.405	0.685	(0.00,0.99)	0.000
clom-fluo-venl	0.169	0.380	0.445	0.657	(0.00,0.91)	0.000
fluo-paro-rebo	0.163	0.371	0.438	0.661	(0.00,0.89)	0.000
bupr-fluo-venl	0.161	0.436	0.371	0.711	(0.00,1.02)	0.000
clom-paro-venl	0.149	0.585	0.255	0.799	(0.00,1.30)	0.119
mirt-paro-sert	0.127	0.456	0.278	0.781	(0.00,1.02)	0.049
cita-mirt-venl	0.120	1.096	0.109	0.913	(0.00,2.27)	0.206
esci-paro-sert	0.119	0.494	0.241	0.809	(0.00,1.09)	0.043
fluo-mirt-traz	0.117	0.594	0.197	0.844	(0.00,1.28)	0.079
bupr-fluo-sert	0.110	1.087	0.102	0.919	(0.00,2.24)	0.000
cita-rebo-venl	0.107	0.485	0.221	0.825	(0.00,1.06)	0.000
fluv-mirt-paro	0.099	0.566	0.175	0.861	(0.00,1.21)	0.121
amit-sert-venl	0.094	0.358	0.261	0.794	(0.00,0.80)	0.000
mirt-paro-traz	0.091	0.516	0.177	0.859	(0.00,1.10)	0.049
fluo-mirt-venl	0.084	0.263	0.317	0.751	(0.00,0.60)	0.000
fluo-mirt-sert	0.083	0.310	0.266	0.790	(0.00,0.69)	0.000
cita-mirt-paro-rebo	0.080	0.736	0.109	0.913	(0.00,1.52)	0.074
agom-esci-venl	0.080	0.353	0.226	0.821	(0.00,0.77)	0.000
clom-miln-paro	0.080	0.637	0.125	0.900	(0.00,1.33)	0.069
dulo-esci-venl	0.075	0.299	0.249	0.803	(0.00,0.66)	0.000
amit-fluv-paro	0.071	0.547	0.129	0.897	(0.00,1.14)	0.125
amit-fluo-miln	0.069	0.426	0.162	0.871	(0.00,0.90)	0.037
fluo-fluv-paro	0.062	0.404	0.153	0.878	(0.00,0.85)	0.000
agom-dulo-paro	0.061	0.484	0.127	0.899	(0.00,1.01)	0.071
bupr-fluo-paro	0.061	0.511	0.118	0.906	(0.00,1.06)	0.000
agom-esci-fluo	0.054	0.327	0.166	0.868	(0.00,0.69)	0.000
cita-clom-fluv	0.051	0.707	0.072	0.942	(0.00,1.44)	0.000

agom-fluo-paro	0.049	0.256	0.192	0.847	(0.00,0.55)	0.012
amit-fluv-mirt-traz	0.045	1.124	0.040	0.968	(0.00,2.25)	0.343
amit-miln-paro	0.039	0.474	0.082	0.935	(0.00,0.97)	0.091
amit-fluo-fluv	0.038	0.362	0.104	0.917	(0.00,0.75)	0.001
fluo-nefa-paro	0.035	0.426	0.081	0.935	(0.00,0.87)	0.000
bupr-paro-sert	0.026	1.086	0.024	0.981	(0.00,2.16)	0.000
fluo-paro-sert	0.019	0.249	0.077	0.939	(0.00,0.51)	0.000

8.3.1.2 Side-splitting

Side	Direct Coef.	Std. Err.	Indirect Coef.	Std. Err.	Difference Coef.	Std. Err.	P>|z|
agom dulo	.2747857	.2521974	-.1595808	.1448596	.4343666	.2908399	0.135
agom esci	-.2054692	.2408502	.2012154	.1296101	-.4066846	.2734618	0.137
agom fluo	-.1357624	.1648437	-.2062446	.1277267	.0704822	.2083064	0.735
agom paro	.1551196	.1852912	-.0805797	.1215433	.2356993	.221775	0.288
agom venl	-.3075187	.2167293	.0777467	.1179195	-.3852654	.2467538	0.118
amit fluo	-.0651015	.1565131	-.2752606	.0972169	.2101591	.1842438	0.254
amit fluv	-.0808348	.2454107	-.2664355	.1358457	.1856008	.2805513	0.508
amit miln	-.1623724	.304638	-.0946298	.1371863	-.0677427	.3339434	0.839
amit paro	-.0742893	.116934	-.0240856	.1085608	-.0502037	.1590613	0.752
amit sert	-.2467633	.1722422	-.0570727	.1111317	-.1896906	.2049945	0.355
amit traz	-.3321745	.3105124	-.3472934	.1565595	.0151189	.3475844	0.965
amit venl	-.1725341	.2808635	-.0327869	.1010942	-.1397471	.2983635	0.640
bupr fluo	-.1733529	.3950105	-.3428694	.1940512	.1695165	.4401013	0.700
bupr paro	-.0008167	.3688016	-.1811793	.198299	.1803626	.4187328	0.667
bupr sert	.0645379	1.026878	-.2147268	.1856432	.2792648	1.043524	0.789
bupr traz	-.7396673	.3970234	-.338129	.2279451	-.4015383	.4578063	0.380
bupr venl	-.1588537	.2613484	-.1287387	.2311477	-.030115	.3489015	0.931
cita clom	.566757	.405286	-.1276823	.1379348	.6944393	.4281153	0.105
cita esci	.3783393	.138393	.0653837	.1462349	.3129556	.2014659	0.120
cita fluo	.0363134	.1954565	-.0889415	.1131575	.1252548	.2258606	0.579
cita fluv	-.1017411	.3325821	-.0566913	.1447811	-.0450497	.3627292	0.901
cita mirt	-.2805174	.3845445	.2336776	.1270722	-.514195	.4049961	0.204
cita rebo	-.5478923	.2551439	-.061945	.1954906	-.4859473	.3214265	0.131
cita sert	.0976222	.2306307	.0313836	.1237106	.0662387	.2617504	0.800
cita venl	-.5232255	.328357	.1839263	.1121416	-.7071518	.3478198	0.042
clom fluo	.4955394	.237831	-.1203524	.1159442	.6158918	.2645773	0.020
clom fluv	-.6174436	.5273701	.0385519	.1443414	-.6559955	.5467663	0.230
clom miln	.1397618	.4214485	.1060928	.1501441	.033669	.4473947	0.940
clom paro	.0880156	.1431286	.247075	.1418997	-.1590594	.2014555	0.430
clom sert	.0457217	.2709229	.1160337	.1283141	-.0703121	.2997657	0.815
clom traz	-.4884265	.4170583	-.070085	.1682902	-.4183415	.4499332	0.352
clom venl	.4928066	.3075177	.1142274	.1226623	.3785792	.3313251	0.253
dulo esci	.3669248	.199267	.0520941	.1457318	.3148307	.2468565	0.202
dulo paro	-.0309282	.1846976	.0836151	.1375155	-.1145433	.2303862	0.619
dulo venl	.1057734	.1788444	.0010995	.1416467	.1046739	.2281406	0.646
esci fluo	.0205539	.2311082	-.3449254	.0999251	.3654793	.2517818	0.147
esci paro	-.0895839	.1839402	-.1280329	.1076797	.0384491	.2130404	0.857
esci sert	.0701931	.3045527	-.2187811	.1131823	.2889742	.3250565	0.374
esci venl	-.190284	.2248258	-.1034011	.1078458	-.0868829	.2493693	0.728
fluo fluv	.0316354	.2641408	-.0144651	.1221473	.0461005	.291005	0.874
fluo miln	-.1548268	.2169856	.215453	.1369002	-.3702797	.2569164	0.150
fluo mirt	.4269079	.1913256	.1808898	.1079203	.2460181	.2199582	0.263
fluo nefa	.0352549	.3287834	.0511978	.2311843	-.0159429	.4019553	0.968
fluo paro	.2370718	.124605	.144114	.0756787	.0929579	.1456865	0.523
fluo rebo	-.0121576	.2920174	-.2437954	.1743634	.2316377	.34024	0.496
fluo sert	.3510676	.1330612	-.0244881	.096269	.3755557	.1642331	0.022
fluo traz	.181445	.2821071	-.2135178	.1481638	.3949628	.3186684	0.215
fluo venl	.2068751	.116299	.1438903	.0917313	.0629848	.1478048	0.670
fluv miln	.5392037	.2944311	-.0063849	.1583282	.5455886	.3343015	0.103
fluv mirt	.1301778	.2307367	.2934699	.1462627	-.1632921	.2731689	0.550
fluv paro	.1317394	.3224493	.1814294	.1190114	-.04969	.3438289	0.885
fluv sert	-.4419129	.3211516	.1988713	.1289356	-.6407842	.3460867	0.064
fluv venl	.8605795	.4432719	.1201468	.1230326	.7404327	.4600292	0.107
miln paro	.0481103	.1658708	.0678289	.1531688	-.0197186	.2257712	0.930
miln sert	-.735707	.9252808	.0078936	.129881	-.7436006	.934352	0.426
mirt paro	.0185567	.1538993	-.1228408	.1166973	.1413975	.1931442	0.464
mirt sert	.0262103	.2719722	-.1659296	.1154465	.1921398	.2954603	0.515
mirt traz	-.4073069	.3225476	-.3578452	.1625971	-.0494616	.3612129	0.891
mirt venl	-.3120889	.2014071	.0042085	.1145826	-.3162974	.2317168	0.172
nefa paro	.2623066	.2916347	.0227264	.2478449	.2395802	.3822987	0.531
nefa sert	-.1541506	.3489625	.1505375	.2306913	-.3046881	.418322	0.466

paro rebo	-.4029367	.2696105	-.3275587	.1830298	-.0753779	.3258677	0.817
paro sert	.1010077	.2117524	-.09617	.0910794	.1971777	.230704	0.393
paro traz	-.5463717	.2755833	-.2243151	.1486195	-.3220565	.3131467	0.304
paro venl	.4529244	.2261595	-.0595068	.0805538	.5124312	.2401329	0.033
rebo venl	-.0436749	.3426637	.4494012	.1709662	-.4930762	.3829462	0.198
sert traz	.5806286	.4163348	-.335826	.1479145	.9164546	.4418295	0.038
sert venl	.1664371	.2101924	.0407784	.1001232	.1256587	.2328428	0.589
traz venl	.7681215	.4332836	.2406392	.1459659	.5274823	.4584009	0.250

8.3.1.3 Design-by-treatment test

chi2(60) = 61.50

Prob > chi2 = 0.3182

8.3.2 Dropouts for any reason

8.3.2.1 Loop-specific approach

Loops With Evidence of Statistical Inconsistency:

Loop	IF	seIF	z_value	p_value	CI_95	Loop_Heterog_tau2
cita-clom-venl	2.706	1.055	2.566	0.010	(0.64,4.77)	0.000
cita-esci-venl	2.327	0.879	2.647	0.008	(0.60,4.05)	0.000
cita-fluo-venl	2.167	0.864	2.508	0.012	(0.47,3.86)	0.001
cita-clom-mirt-paro	1.946	0.699	2.786	0.005	(0.58,3.32)	0.000
cita-clom-sert	1.534	0.652	2.354	0.019	(0.26,2.81)	0.000
agom-dulo-venl	1.312	0.392	3.349	0.001	(0.54,2.08)	0.000
cita-mirt-paro-rebo	1.251	0.599	2.087	0.037	(0.08,2.43)	0.000
fluv-mirt-venl	1.180	0.539	2.190	0.029	(0.12,2.24)	0.000
agom-paro-venl	0.851	0.361	2.359	0.018	(0.14,1.56)	0.000
cita-fluo-sert	0.757	0.368	2.054	0.040	(0.03,1.48)	0.008
amit-fluo-paro	0.490	0.240	2.042	0.041	(0.02,0.96)	0.000

Loops Without Evidence of Statistical Inconsistency:

Loop	IF	seIF	z_value	p_value	CI_95	Loop_Heterog_tau2
cita-sert-venl	1.365	1.167	1.170	0.242	(0.00,3.65)	0.283
fluo-sert-traz	1.173	0.839	1.398	0.162	(0.00,2.82)	0.085
amit-bupr-fluo	1.145	0.678	1.689	0.091	(0.00,2.47)	0.000
amit-fluv-sert	1.105	0.612	1.806	0.071	(0.00,2.30)	0.029
cita-fluo-mirt	1.020	0.528	1.931	0.054	(0.00,2.05)	0.000
agom-dulo-esci	1.002	0.724	1.384	0.166	(0.00,2.42)	0.161
cita-esci-mirt-paro	0.981	0.557	1.763	0.078	(0.00,2.07)	0.000
cita-mirt-venl	0.981	0.960	1.022	0.307	(0.00,2.86)	0.000
amit-fluv-venl	0.981	0.676	1.450	0.147	(0.00,2.31)	0.042
cita-clom-esci-paro	0.965	0.595	1.622	0.105	(0.00,2.13)	0.000
cita-fluv-sert	0.924	1.314	0.703	0.482	(0.00,3.50)	0.484
clom-fluo-fluv	0.902	0.909	0.992	0.321	(0.00,2.68)	0.000
amit-bupr-paro	0.885	0.735	1.205	0.228	(0.00,2.32)	0.040
cita-esci-sert	0.789	0.519	1.520	0.129	(0.00,1.81)	0.043
clom-fluv-paro	0.776	0.881	0.881	0.379	(0.00,2.50)	0.000
cita-clom-fluo	0.774	0.608	1.273	0.203	(0.00,1.97)	0.000
amit-sert-traz	0.741	0.728	1.018	0.309	(0.00,2.17)	0.000
amit-bupr-venl	0.735	0.637	1.153	0.249	(0.00,1.98)	0.000
fluo-nefa-sert	0.731	0.648	1.127	0.260	(0.00,2.00)	0.029
fluv-paro-sert	0.697	0.678	1.028	0.304	(0.00,2.03)	0.033
cita-clom-paro-rebo	0.696	0.635	1.095	0.273	(0.00,1.94)	0.000
fluv-mirt-sert	0.696	0.851	0.818	0.413	(0.00,2.36)	0.095
bupr-fluo-traz	0.692	1.269	0.545	0.586	(0.00,3.18)	0.379
fluo-fluv-venl	0.679	0.564	1.203	0.229	(0.00,1.79)	0.009

miln-paro-sert	0.675	0.614	1.100	0.271	(0.00,1.88)	0.000
mirt-sert-traz	0.669	0.735	0.910	0.363	(0.00,2.11)	0.000
bupr-paro-traz	0.656	0.610	1.076	0.282	(0.00,1.85)	0.000
fluo-mirt-traz	0.647	0.634	1.020	0.308	(0.00,1.89)	0.092
amit-mirt-traz-venl	0.634	0.606	1.047	0.295	(0.00,1.82)	0.000
mirt-paro-traz	0.634	0.535	1.186	0.236	(0.00,1.68)	0.027
fluv-mirt-paro	0.614	0.431	1.423	0.155	(0.00,1.46)	0.000
clom-fluv-sert	0.592	1.393	0.425	0.671	(0.00,3.32)	0.288
fluo-nefa-paro	0.580	0.551	1.053	0.292	(0.00,1.66)	0.000
mirt-paro-sert	0.573	0.376	1.525	0.127	(0.00,1.31)	0.000
cita-paro-rebo-sert	0.551	0.520	1.060	0.289	(0.00,1.57)	0.007
amit-fluv-paro	0.549	0.469	1.170	0.242	(0.00,1.47)	0.038
esci-fluo-paro	0.544	0.352	1.545	0.122	(0.00,1.24)	0.000
amit-fluo-sert	0.544	0.296	1.837	0.066	(0.00,1.12)	0.000
agom-esci-paro	0.542	0.407	1.333	0.183	(0.00,1.34)	0.000
fluo-fluv-sert	0.534	0.675	0.792	0.428	(0.00,1.86)	0.057
clom-paro-venl	0.525	0.482	1.088	0.277	(0.00,1.47)	0.000
amit-fluv-miln	0.518	0.564	0.919	0.358	(0.00,1.62)	0.009
amit-miln-sert	0.517	0.691	0.748	0.454	(0.00,1.87)	0.000
bupr-sert-traz-venl	0.497	1.304	0.382	0.703	(0.00,3.05)	0.330
fluo-paro-rebo	0.487	0.403	1.210	0.226	(0.00,1.28)	0.000
cita-fluo-rebo	0.449	0.437	1.028	0.304	(0.00,1.30)	0.000
amit-fluv-mirt-traz	0.431	0.593	0.727	0.467	(0.00,1.59)	0.000
mirt-sert-venl	0.411	0.657	0.626	0.532	(0.00,1.70)	0.112
clom-sert-venl	0.410	0.870	0.472	0.637	(0.00,2.12)	0.221
agom-esci-venl	0.370	0.430	0.861	0.389	(0.00,1.21)	0.000
esci-paro-sert	0.367	0.572	0.641	0.521	(0.00,1.49)	0.060
esci-fluo-sert	0.363	0.523	0.693	0.488	(0.00,1.39)	0.063
cita-fluv-mirt	0.353	0.600	0.589	0.556	(0.00,1.53)	0.000
amit-bupr-traz	0.348	0.731	0.476	0.634	(0.00,1.78)	0.000
amit-fluo-miln	0.344	0.451	0.763	0.445	(0.00,1.23)	0.000
fluo-fluv-mirt	0.335	0.435	0.771	0.441	(0.00,1.19)	0.000
cita-fluo-fluv	0.331	0.479	0.691	0.489	(0.00,1.27)	0.000
fluo-paro-venl	0.331	0.273	1.212	0.226	(0.00,0.87)	0.000
agom-fluo-venl	0.330	0.348	0.951	0.342	(0.00,1.01)	0.022
agom-dulo-paro	0.329	0.354	0.929	0.353	(0.00,1.02)	0.000
cita-esci-fluo	0.314	0.356	0.880	0.379	(0.00,1.01)	0.000
fluo-miln-sert	0.311	0.620	0.501	0.616	(0.00,1.53)	0.006
mirt-paro-venl	0.303	0.324	0.937	0.349	(0.00,0.94)	0.000
clom-paro-sert	0.297	0.418	0.710	0.478	(0.00,1.12)	0.000
amit-fluo-venl	0.289	0.367	0.787	0.431	(0.00,1.01)	0.000
cita-fluv-paro-rebo	0.284	0.587	0.483	0.629	(0.00,1.43)	0.000
cita-esci-paro-rebo	0.269	0.475	0.568	0.570	(0.00,1.20)	0.000
fluv-sert-venl	0.249	1.253	0.199	0.842	(0.00,2.71)	0.444
bupr-mirt-traz-venl	0.248	0.569	0.435	0.664	(0.00,1.36)	0.000
clom-fluo-venl	0.235	0.523	0.450	0.653	(0.00,1.26)	0.003
fluo-paro-sert	0.215	0.292	0.736	0.461	(0.00,0.79)	0.000
cita-clom-fluv	0.203	1.007	0.202	0.840	(0.00,2.18)	0.000
bupr-fluo-paro	0.197	0.585	0.336	0.737	(0.00,1.34)	0.000
dulo-esci-paro	0.195	0.567	0.344	0.731	(0.00,1.31)	0.128
esci-sert-venl	0.195	0.726	0.269	0.788	(0.00,1.62)	0.197
fluo-mirt-paro	0.193	0.289	0.667	0.505	(0.00,0.76)	0.000
amit-paro-sert	0.185	0.310	0.597	0.551	(0.00,0.79)	0.000
agom-fluo-paro	0.181	0.260	0.696	0.487	(0.00,0.69)	0.000
agom-esci-fluo	0.180	0.439	0.410	0.682	(0.00,1.04)	0.021
amit-paro-traz	0.178	0.537	0.332	0.740	(0.00,1.23)	0.042
fluo-paro-traz	0.178	0.423	0.419	0.675	(0.00,1.01)	0.005
fluo-fluv-miln	0.171	0.491	0.349	0.727	(0.00,1.13)	0.000
fluo-mirt-venl	0.165	0.304	0.543	0.587	(0.00,0.76)	0.000
amit-fluo-traz	0.159	0.494	0.323	0.747	(0.00,1.13)	0.000
amit-sert-venl	0.158	0.455	0.348	0.728	(0.00,1.05)	0.020
esci-fluo-venl	0.145	0.376	0.386	0.700	(0.00,0.88)	0.017
fluo-mirt-sert	0.141	0.380	0.371	0.711	(0.00,0.89)	0.005
amit-paro-venl	0.139	0.408	0.340	0.734	(0.00,0.94)	0.011
cita-mirt-sert	0.137	0.572	0.239	0.811	(0.00,1.26)	0.000
dulo-paro-venl	0.132	0.324	0.409	0.683	(0.00,0.77)	0.000
fluo-miln-paro	0.120	0.280	0.427	0.669	(0.00,0.67)	0.000
bupr-fluo-venl	0.113	0.519	0.217	0.828	(0.00,1.13)	0.017
nefa-paro-sert	0.103	0.517	0.199	0.842	(0.00,1.12)	0.000
fluo-sert-venl	0.092	0.377	0.245	0.807	(0.00,0.83)	0.056
fluo-fluv-paro	0.086	0.452	0.190	0.849	(0.00,0.97)	0.000
esci-paro-venl	0.061	0.386	0.159	0.874	(0.00,0.82)	0.000
fluv-miln-sert	0.056	1.119	0.050	0.960	(0.00,2.25)	0.183
clom-fluo-paro	0.040	0.332	0.119	0.905	(0.00,0.69)	0.000
fluv-miln-paro	0.034	0.489	0.071	0.944	(0.00,0.99)	0.000
dulo-esci-venl	0.025	0.477	0.053	0.958	(0.00,0.96)	0.068
clom-fluo-sert	0.018	0.465	0.039	0.969	(0.00,0.93)	0.000

cita-esci-fluv-paro	0.014	0.543	0.026	0.979	(0.00,1.08)	0.000
bupr-paro-venl	0.012	0.506	0.025	0.980	(0.00,1.00)	0.000
clom-fluv-venl	0.011	1.014	0.011	0.991	(0.00,2.00)	0.000
amit-miln-paro	0.010	0.445	0.022	0.983	(0.00,0.88)	0.019
amit-fluo-fluv	0.008	0.410	0.021	0.984	(0.00,0.81)	0.000

8.3.2.2 Side-splitting

Side	Direct Coef.	Std. Err.	Indirect Coef.	Std. Err.	Difference Coef.	Std. Err.	P>|z|
agom dulo	-.1481216	.2591986	.6396765	.1443544	-.7877981	.2966852	0.008
agom esci	.2397861	.2919887	-.0200878	.1419339	.2598739	.324622	0.423
agom fluo	.2563222	.1640613	.0867768	.1358545	.1695453	.2117746	0.423
agom paro	.0186653	.1840965	.2930637	.1290091	-.2743984	.2248431	0.222
agom venl	.718039	.2363567	.1717818	.1222956	.5462572	.266145	0.040
amit bupr	.5764195	.5237737	-.1900441	.1851085	.7664636	.5555215	0.168
amit fluo	-.4283316	.171572	-.053039	.1028903	-.3752926	.2000564	0.061
amit fluv	-.2776619	.2342912	.1743344	.1498418	-.4519962	.2781556	0.104
amit miln	-.0183109	.3718603	-.0952345	.1431375	.0769236	.3983168	0.847
amit paro	.0086852	.1215845	-.2061398	.115156	.214825	.1671171	0.199
amit sert	-.0302221	.1971315	-.2149731	.1233489	.1847511	.232623	0.427
amit traz	.1442308	.3686544	-.0081396	.1747364	.1523704	.4078954	0.709
amit venl	-.0079104	.30938	-.0192096	.1084152	.0112993	.3279154	0.973
bupr fluo	.1402558	.4130355	-.0847723	.1817897	.2250281	.4512713	0.618
bupr paro	.2883554	.4142058	-.0559859	.1827296	.3443413	.4527212	0.447
bupr traz	-.0800426	.3760925	.2038381	.2334629	-.2838807	.442663	0.521
bupr venl	.1485547	.2342833	.0276953	.229131	.1208594	.3277036	0.712
cita clom	-.4906219	.5060083	.5239905	.1571142	-1.014612	.5298389	0.05
cita esci	-.2911876	.1881383	.0617449	.1674529	-.3529325	.2519925	0.161
cita fluo	-.1420769	.2047063	.1079371	.1357768	-.250014	.2456676	0.309
cita fluv	.3485738	.3337713	.1992701	.1665542	.1493037	.3730196	0.689
cita mirt	.8601197	.4494768	.0046426	.1424429	.8554771	.4715075	0.070
cita rebo	.4728521	.2815487	.5138439	.2320413	-.0409918	.3648463	0.911
cita sert	.4715263	.2515499	-.1245576	.1430265	.5960839	.2891466	0.039
cita venl	2.154643	.8337837	.1213496	.1259603	2.033294	.8432443	0.016
clom fluo	-.4233596	.2904297	-.395739	.1344692	-.0276206	.3199491	0.931
clom fluv	.6359839	.8155056	-.237897	.160877	.8738809	.8312225	0.293
clom paro	-.4316628	.1363726	-.2031864	.1891448	-.2284764	.233158	0.327
clom sert	-.5923985	.3437739	-.3776454	.146842	-.2147531	.3742026	0.566
clom venl	-.0606247	.4228216	-.2869998	.1366108	.2263751	.4443429	0.610
dulo esci	-.5764256	.2120457	-.3435293	.1584891	-.2328963	.2647957	0.379
dulo paro	-.153148	.1797998	-.320009	.1468818	.1668609	.2322052	0.472
dulo venl	-.4471851	.1833211	.0027329	.1427816	-.449918	.2324948	0.053
esci fluo	-.1682946	.2406889	.1919826	.114824	-.3602772	.2664812	0.176
esci paro	.318897	.2410639	.1394872	.1171341	.1794098	.2682203	0.504
esci sert	.0617732	.3284748	.1245345	.129822	-.0627614	.3531738	0.859
esci venl	.1086348	.2310139	.3058658	.1250286	-.1972309	.2627265	0.453
fluo fluv	.1544566	.3028321	.2055013	.1327658	-.0510447	.3309703	0.877
fluo miln	.0739673	.2128905	.0652938	.1498162	.0086735	.2603505	0.973
fluo mirt	-.0214108	.2146199	.0732734	.1138881	-.0946842	.2431691	0.697
fluo nefa	.5678896	.453223	.0419022	.2435346	.5259874	.5144989	0.307
fluo paro	-.0520726	.1252834	.0901345	.0810986	-.1422071	.149332	0.341
fluo rebo	.1648873	.2907283	.619195	.2061443	-.4543077	.3563983	0.202
fluo sert	-.1503814	.1554658	.0598886	.1091688	-.2102701	.1900234	0.268
fluo traz	.419724	.2922967	.0855953	.174006	.3341287	.3401738	0.326
fluo venl	.1256649	.1262349	.1417501	.1008705	-.0160852	.1618434	0.921
fluv miln	-.2603573	.3419958	-.096468	.1703102	-.1638893	.3820285	0.668
fluv mirt	.1584327	.2483018	-.2661683	.1576238	.424601	.294111	0.149
fluv paro	-.2929141	.3263337	-.1260715	.1300402	-.1668426	.3512849	0.635
fluv sert	-.879688	.468195	-.1455606	.1392814	-.7341274	.488769	0.133
fluv venl	-.7075936	.4501581	-.0024729	.1350686	-.7051207	.4699849	0.134
miln paro	.0063752	.1684073	-.0469917	.1689909	.0533669	.2389022	0.823
miln sert	-.5332983	.560766	-.0481639	.1426691	-.4851344	.5786303	0.402
mirt paro	.1618315	.1636529	-.0966993	.1226802	.2585307	.2044442	0.206
mirt sert	-.269815	.2775062	-.0184461	.1272447	-.2513688	.3052882	0.410
mirt traz	-.1623425	.3394146	.2038089	.1838224	-.3661514	.3859961	0.343
mirt venl	.3138311	.1931975	-.0128804	.1251598	.3267116	.2301958	0.156
nefa paro	-.0409628	.3020986	-.1822794	.2994612	.1413166	.425062	0.740
nefa sert	.0016963	.3786828	-.2552128	.2669577	.256909	.4633218	0.579
paro rebo	.7006942	.2684461	.2355098	.2167271	.4651845	.345013	0.178
paro sert	.1390516	.2221847	-.0980123	.1004749	.2370639	.2438279	0.331
paro traz	.2945897	.2838392	.060267	.1755716	.2343226	.3333859	0.482

paro	venl	-.1506307	.2223719	.124587	.0881634	-.2752177	.239216	0.250
sert	traz	-.5618102	.6071661	.2399672	.1688958	-.8017774	.6302194	0.203
sert	venl	.1844182	.2408344	.1367055	.1118982	.0477128	.2655927	0.857

8.3.2.3 Design-by-treatment test

chi2(57) = 70.47

Prob > chi2 = 0.0544

8.3.3 Efficacy continuous

8.3.3.1 Loop-specific approach

Loops With Evidence of Statistical Inconsistency:

Loop	IF	seIF	z_value	p_value	CI_95	Loop_Heterog_tau2
clom-fluo-miln	1.015	0.284	3.570	0.000	(0.46,1.57)	0.000
amit-clom-miln-sert	0.837	0.298	2.807	0.005	(0.25,1.42)	0.000
clom-fluv-miln	0.724	0.337	2.151	0.031	(0.06,1.38)	0.000
cita-clom-fluo	0.590	0.257	2.293	0.022	(0.09,1.09)	0.000
agom-dulo-esci	0.530	0.161	3.282	0.001	(0.21,0.85)	0.000
agom-dulo-venl	0.357	0.147	2.424	0.015	(0.07,0.65)	0.000

Loops Without Evidence of Statistical Inconsistency:

Loop	IF	seIF	z_value	p_value	CI_95	Loop_Heterog_tau2
amit-clom-miln-venl	0.876	0.545	1.608	0.108	(0.00,1.94)	0.053
clom-miln-paro	0.609	0.415	1.468	0.142	(0.00,1.42)	0.038
cita-clom-venl	0.579	0.329	1.757	0.079	(0.00,1.22)	0.000
cita-clom-sert	0.503	0.336	1.496	0.135	(0.00,1.16)	0.017
clom-fluv-sert	0.436	0.296	1.473	0.141	(0.00,1.02)	0.000
clom-fluo-fluv	0.435	0.289	1.504	0.133	(0.00,1.00)	0.000
cita-clom-fluv	0.403	0.328	1.229	0.219	(0.00,1.05)	0.000
cita-clom-paro-rebo	0.380	0.611	0.622	0.534	(0.00,1.58)	0.070
fluv-sert-venl	0.369	0.370	0.999	0.318	(0.00,1.09)	0.018
cita-clom-mirt-paro	0.365	0.562	0.649	0.516	(0.00,1.47)	0.082
cita-fluv-sert	0.338	0.330	1.025	0.305	(0.00,0.99)	0.023
bupr-sert-traz	0.321	0.569	0.564	0.573	(0.00,1.44)	0.000
dulo-esci-paro	0.315	0.173	1.824	0.068	(0.00,0.65)	0.008
agom-esci-paro	0.305	0.202	1.513	0.130	(0.00,0.70)	0.013
cita-esci-fluv-paro	0.303	0.257	1.180	0.238	(0.00,0.81)	0.006
agom-paro-venl	0.297	0.197	1.512	0.131	(0.00,0.68)	0.007
amit-fluv-venl	0.290	0.316	0.918	0.359	(0.00,0.91)	0.000
amit-fluv-miln	0.284	0.379	0.749	0.454	(0.00,1.03)	0.040
clom-fluo-paro	0.274	0.204	1.344	0.179	(0.00,0.67)	0.017
cita-fluo-rebo	0.273	0.189	1.442	0.149	(0.00,0.64)	0.000
bupr-fluo-sert	0.260	0.541	0.481	0.631	(0.00,1.32)	0.000
amit-paro-traz	0.260	0.338	0.768	0.442	(0.00,0.92)	0.016
cita-fluo-fluv	0.247	0.200	1.238	0.216	(0.00,0.64)	0.000
fluv-miln-paro	0.245	0.222	1.102	0.270	(0.00,0.68)	0.000
cita-clom-esci-paro	0.231	0.338	0.685	0.493	(0.00,0.89)	0.026
amit-bupr-traz-venl	0.230	0.355	0.647	0.518	(0.00,0.93)	0.000
cita-esci-fluo	0.224	0.137	1.630	0.103	(0.00,0.49)	0.000
fluo-paro-rebo	0.205	0.180	1.134	0.257	(0.00,0.56)	0.000
fluo-fluv-venl	0.200	0.298	0.672	0.502	(0.00,0.78)	0.004
bupr-fluo-venl	0.194	0.249	0.780	0.435	(0.00,0.68)	0.006
bupr-fluo-paro	0.186	0.259	0.715	0.475	(0.00,0.69)	0.000
cita-fluo-mirt	0.175	0.225	0.778	0.437	(0.00,0.62)	0.012
cita-esci-venl	0.171	0.214	0.796	0.426	(0.00,0.59)	0.005

amit-fluo-traz	0.167	0.409	0.407	0.684	(0.00,0.97)	0.030
cita-esci-mirt-paro	0.166	0.297	0.560	0.576	(0.00,0.75)	0.029
clom-paro-sert	0.166	0.310	0.535	0.593	(0.00,0.77)	0.040
fluo-fluv-sert	0.157	0.205	0.766	0.444	(0.00,0.56)	0.000
clom-fluo-sert	0.156	0.199	0.785	0.432	(0.00,0.55)	0.000
cita-fluv-paro-rebo	0.154	0.265	0.581	0.561	(0.00,0.67)	0.000
esci-fluo-venl	0.151	0.166	0.913	0.361	(0.00,0.48)	0.006
cita-esci-paro-rebo	0.148	0.235	0.630	0.529	(0.00,0.61)	0.008
esci-fluo-paro	0.148	0.143	1.030	0.303	(0.00,0.43)	0.003
fluo-paro-traz	0.147	0.226	0.648	0.517	(0.00,0.59)	0.005
fluo-sert-traz	0.146	0.270	0.540	0.589	(0.00,0.67)	0.002
fluo-fluv-miln	0.144	0.221	0.652	0.514	(0.00,0.58)	0.000
esci-fluo-sert	0.141	0.159	0.890	0.374	(0.00,0.45)	0.000
cita-esci-sert	0.138	0.184	0.753	0.452	(0.00,0.50)	0.009
clom-fluv-paro	0.138	0.394	0.350	0.727	(0.00,0.91)	0.049
fluo-rebo-venl	0.137	0.227	0.605	0.545	(0.00,0.58)	0.005
clom-fluv-venl	0.131	0.394	0.332	0.740	(0.00,0.90)	0.000
fluv-paro-sert	0.130	0.238	0.547	0.585	(0.00,0.60)	0.000
dulo-paro-venl	0.126	0.151	0.838	0.402	(0.00,0.42)	0.000
bupr-paro-sert	0.126	0.540	0.233	0.816	(0.00,1.18)	0.000
fluo-miln-paro	0.125	0.150	0.833	0.405	(0.00,0.42)	0.000
amit-paro-venl	0.116	0.260	0.448	0.654	(0.00,0.63)	0.019
fluo-paro-venl	0.116	0.138	0.834	0.404	(0.00,0.39)	0.002
agom-fluo-paro	0.112	0.123	0.911	0.362	(0.00,0.35)	0.003
agom-esci-venl	0.108	0.177	0.610	0.542	(0.00,0.46)	0.005
fluo-mirt-venl	0.106	0.168	0.632	0.527	(0.00,0.44)	0.010
fluv-mirt-venl	0.106	0.351	0.301	0.763	(0.00,0.79)	0.019
amit-fluv-paro	0.105	0.285	0.369	0.712	(0.00,0.66)	0.016
fluo-mirt-paro	0.103	0.188	0.547	0.584	(0.00,0.47)	0.025
cita-fluv-mirt	0.102	0.208	0.490	0.624	(0.00,0.51)	0.000
mirt-paro-venl	0.097	0.405	0.240	0.811	(0.00,0.89)	0.068
clom-fluo-venl	0.092	0.257	0.359	0.720	(0.00,0.60)	0.002
cita-fluo-venl	0.092	0.209	0.441	0.659	(0.00,0.50)	0.005
bupr-paro-venl	0.092	0.234	0.392	0.695	(0.00,0.55)	0.000
agom-fluo-venl	0.084	0.144	0.581	0.561	(0.00,0.37)	0.007
amit-fluo-fluv	0.079	0.221	0.358	0.720	(0.00,0.51)	0.004
agom-dulo-paro	0.078	0.186	0.420	0.675	(0.00,0.44)	0.007
dulo-esci-venl	0.078	0.145	0.540	0.589	(0.00,0.36)	0.000
bupr-fluo-traz	0.077	0.314	0.247	0.805	(0.00,0.69)	0.000
fluo-nefa-paro	0.074	0.213	0.350	0.726	(0.00,0.49)	0.000
amit-fluo-miln	0.074	0.288	0.257	0.797	(0.00,0.64)	0.023
fluv-mirt-paro	0.072	0.357	0.202	0.840	(0.00,0.77)	0.065
clom-paro-venl	0.071	0.519	0.137	0.891	(0.00,1.09)	0.070
amit-fluo-sert	0.070	0.132	0.533	0.594	(0.00,0.33)	0.000
amit-sert-venl	0.066	0.190	0.349	0.727	(0.00,0.44)	0.001
nefa-paro-sert	0.064	0.229	0.278	0.781	(0.00,0.51)	0.000
bupr-paro-traz	0.058	0.281	0.205	0.838	(0.00,0.61)	0.000
amit-miln-paro	0.058	0.236	0.244	0.807	(0.00,0.52)	0.017
cita-fluo-sert	0.057	0.133	0.428	0.669	(0.00,0.32)	0.000
bupr-sert-venl	0.052	0.528	0.099	0.921	(0.00,1.09)	0.000
fluo-fluv-mirt	0.047	0.238	0.197	0.844	(0.00,0.51)	0.018
esci-paro-sert	0.046	0.193	0.238	0.812	(0.00,0.43)	0.009
cita-fluv-venl	0.045	0.332	0.136	0.892	(0.00,0.70)	0.000
fluo-paro-sert	0.043	0.124	0.351	0.725	(0.00,0.29)	0.000
cita-paro-rebo-sert	0.042	0.343	0.122	0.903	(0.00,0.71)	0.018
agom-esci-fluo	0.041	0.162	0.254	0.799	(0.00,0.36)	0.003
fluo-nefa-sert	0.040	0.240	0.169	0.866	(0.00,0.51)	0.000
cita-mirt-venl	0.040	0.224	0.180	0.857	(0.00,0.48)	0.000
cita-rebo-venl	0.039	0.252	0.153	0.878	(0.00,0.53)	0.000
amit-sert-traz	0.028	0.321	0.088	0.930	(0.00,0.66)	0.000
amit-fluo-venl	0.025	0.173	0.144	0.885	(0.00,0.37)	0.007
fluo-fluv-paro	0.024	0.211	0.116	0.908	(0.00,0.44)	0.000
cita-mirt-paro-rebo	0.022	0.624	0.036	0.972	(0.00,1.24)	0.099
amit-paro-sert	0.021	0.162	0.129	0.897	(0.00,0.34)	0.008
amit-fluv-sert	0.010	0.224	0.047	0.963	(0.00,0.45)	0.000
cita-mirt-paro-sert	0.009	0.434	0.020	0.984	(0.00,0.86)	0.056
amit-fluo-paro	0.004	0.124	0.036	0.972	(0.00,0.25)	0.008
fluo-sert-venl	0.004	0.143	0.029	0.977	(0.00,0.28)	0.001
esci-sert-venl	0.003	0.231	0.012	0.990	(0.00,0.45)	0.014

8.3.3.2 Side-splitting

Side	Direct Coef.	Std. Err.	Indirect Coef.	Std. Err.	Difference Coef.	Std. Err.	P>|z|
agom dulo	-.2372621	.1246922	-.004516	.0723209	-.2327461	.1441474	0.106
agom esci	.1064611	.1096172	-.1554867	.0623718	.2619478	.1260936	0.038
agom fluo	.0646071	.0831887	.0763364	.0619722	-.0117294	.103677	0.910
agom paro	-.1564327	.0925606	.0060971	.0591666	-.1625298	.1099062	0.139
agom venl	.0617733	.0982373	-.0648789	.0603852	.1266522	.115314	0.272
amit fluo	.1707998	.08211	.1722394	.0530301	-.0014395	.0977522	0.988
amit fluv	-.0219119	.1619977	.1455029	.0700747	-.1674148	.1765041	0.343
amit miln	.0902417	.1542771	.1168112	.0765152	-.0265695	.1722542	0.877
amit paro	.0692971	.0621417	.0503619	.0583015	.0189352	.0852328	0.824
amit sert	.1601147	.0944628	.1115442	.0603864	.0485704	.1121158	0.665
amit traz	.0020891	.2622183	.1659	.0938482	-.1638109	.2785065	0.556
amit venl	.1070927	.1354572	.0637587	.0545633	.0433339	.1460585	0.767
bupr fluo	.2774453	.2004499	.1032407	.1014366	.1742046	.2246542	0.438
bupr paro	-.0140928	.1915195	.0380372	.1034224	-.05213	.2176601	0.811
bupr sert	-.0629924	.5102624	.0982792	.0973715	-.1612716	.5194698	0.756
bupr traz	.1271642	.2064127	.1091969	.1304603	.0179673	.2441845	0.941
bupr venl	-.0013175	.1368964	.0663124	.1202997	-.0676299	.1822433	0.711
cita clom	-.3747901	.2140697	-.0049093	.0708785	-.3698808	.2254985	0.101
cita esci	-.1688673	.0615004	-.0087766	.0715224	-.1600908	.0943031	0.090
cita fluo	.0540477	.0990789	.06396	.0545127	-.0099124	.113088	0.930
cita fluv	.1855169	.1567446	-.0290573	.0727418	.2145742	.1728012	0.214
cita mirt	.0202473	.1455782	-.1002115	.0687101	.1204587	.1609785	0.454
cita rebo	.2558838	.1372913	.085245	.0978406	.1706387	.1685873	0.311
cita sert	.0320441	.1003025	.0087544	.0633933	.0232897	.1186685	0.844
cita venl	.0658868	.1827417	-.0498076	.0544945	.1156944	.1906939	0.544
clom fluo	-.1599586	.1496697	.1510045	.0643846	-.3109631	.1629168	0.056
clom fluv	.1574928	.2281729	.0383311	.0783087	.1191617	.2412369	0.621
clom miln	.688798	.2257066	-.0406472	.0805896	.7294452	.2396626	0.002
clom paro	-.0048725	.0757833	-.0132244	.0809899	.0083519	.1108997	0.940
clom sert	-.086769	.1376204	.0958134	.0712522	-.1825824	.1550196	0.239
clom venl	-.1378385	.2185344	.0142365	.0656801	-.152075	.228191	0.505
dulo esci	-.1926235	.1003116	.0546759	.0711967	-.2472994	.1230103	0.044
dulo paro	.1576163	.0918776	-.0540704	.0687091	.2116868	.1147619	0.065
dulo venl	-.0580184	.0909714	.0879704	.07111	-.1459888	.1154285	0.206
esci fluo	-.0005546	.1113246	.1931755	.0485245	-.1937301	.1214485	0.111
esci paro	.0383115	.0922882	.0533117	.0522797	-.0150001	.1060485	0.888
esci sert	.0591822	.1211814	.1291358	.0579021	-.0699536	.1343053	0.602
esci venl	.065861	.1078968	.0588171	.0536147	.0070439	.120589	0.953
fluo fluv	-.1126686	.1351202	-.0390349	.0643987	-.0736337	.1497068	0.623
fluo miln	-.1652961	.1471023	-.0352478	.0717488	-.1300483	.1636673	0.427
fluo mirt	-.1964439	.0988048	-.117288	.0623759	-.0791559	.1170072	0.499
fluo nefa	-.040939	.1653198	.0047798	.1190907	-.0457188	.2037492	0.822
fluo paro	-.1048271	.0619164	-.1159429	.0404309	.0111158	.0739577	0.881
fluo rebo	-.066511	.1413458	.1407458	.0894675	-.2072568	.1673351	0.216
fluo sert	-.0839804	.0757402	-.0282443	.0521699	-.0557361	.0919646	0.544
fluo traz	-.0545399	.1809548	-.0162014	.0950267	-.0383385	.204651	0.851
fluo venl	-.0853557	.0597703	-.1130421	.0482949	.0276864	.0769361	0.719
fluv miln	-.1922666	.1474932	.0570737	.0873882	-.2493403	.1714301	0.146
fluv mirt	-.0637746	.1144129	-.0988758	.0807128	.0351013	.1400172	0.802
fluv paro	-.0120494	.1727242	-.066089	.0620355	.0540395	.1835298	0.768
fluv sert	.1942694	.1613438	-.0281696	.0693959	.222439	.1756396	0.205
fluv venl	-.1647312	.2664656	-.0424986	.0645521	-.1222327	.2741731	0.656
miln paro	-.0658244	.0873911	-.0389923	.0870669	-.0268321	.1233602	0.828
mirt paro	.0551035	.0895213	.0124208	.0652775	.0426828	.1112353	0.701
mirt venl	.0052368	.1072257	.0499178	.0652481	-.044681	.1255176	0.722
nefa paro	-.1476319	.1467949	-.0670285	.1276729	-.0806034	.194544	0.679
nefa sert	-4.77e-09	.1852943	-.0494861	.1172212	.0494861	.2192596	0.821
paro rebo	.238503	.1386985	.1733964	.0926324	.0651066	.1667873	0.696
paro sert	.0699296	.1041508	.0656133	.0490683	.0043163	.1151462	0.970
paro traz	.1938981	.1260637	.0092994	.1088734	.1845987	.1666091	0.268
paro venl	-.0787694	.1351125	.0194973	.042685	-.0982667	.1416948	0.488
rebo venl	-.1513167	.1745292	-.1910701	.0883765	.0397534	.1956293	0.839
sert traz	-.1307873	.1984509	.0583241	.0973059	-.1891114	.2210231	0.392
sert venl	.0093521	.1260996	-.0673504	.0530686	.0767025	.1368114	0.575

8.3.3.3 *Design-by-treatment test*

chi2(55) = 42.81

Prob > chi2 = 0.8145

8.3.4 Remission

8.3.4.1 *Loop-specific approach*

Loops With Evidence of Statistical Inconsistency:

Loop	IF	seIF	z_value	p_value	CI_95	Loop_Heterog_tau2
cita-clom-venl	2.334	0.710	3.288	0.001	(0.94,3.73)	0.000
cita-clom-fluo	1.707	0.493	3.464	0.001	(0.74,2.67)	0.000
cita-esci-venl	1.345	0.639	2.103	0.035	(0.09,2.60)	0.184
cita-clom-sert	1.332	0.573	2.323	0.020	(0.21,2.46)	0.031
clom-fluo-miln	1.108	0.543	2.041	0.041	(0.04,2.17)	0.000
agom-dulo-esci	1.088	0.350	3.105	0.002	(0.40,1.77)	0.000
agom-dulo-venl	1.006	0.326	3.090	0.002	(0.37,1.64)	0.000
cita-fluo-venl	0.782	0.395	1.977	0.048	(0.01,1.56)	0.009
fluo-paro-venl	0.533	0.258	2.063	0.039	(0.03,1.04)	0.009

Loops Without Evidence of Statistical Inconsistency:

Loop	IF	seIF	z_value	p_value	CI_95	Loop_Heterog_tau2
cita-clom-mirt-paro	1.447	0.797	1.814	0.070	(0.00,3.01)	0.111
cita-clom-fluv	1.444	0.783	1.846	0.065	(0.00,2.98)	0.000
bupr-sert-traz	1.428	1.218	1.173	0.241	(0.00,3.82)	0.000
cita-clom-paro-rebo	1.322	1.085	1.219	0.223	(0.00,3.45)	0.206
cita-sert-venl	1.159	0.675	1.717	0.086	(0.00,2.48)	0.189
cita-fluv-sert	1.032	0.743	1.389	0.165	(0.00,2.49)	0.111
clom-fluo-fluv	0.959	0.604	1.588	0.112	(0.00,2.14)	0.000
agom-paro-venl	0.938	0.486	1.931	0.053	(0.00,1.89)	0.070
cita-esci-fluv-paro	0.919	0.861	1.067	0.286	(0.00,2.61)	0.157
clom-fluv-sert	0.906	0.649	1.397	0.162	(0.00,2.18)	0.000
bupr-sert-venl	0.889	1.288	0.691	0.490	(0.00,3.41)	0.157
cita-clom-esci-paro	0.843	0.792	1.064	0.287	(0.00,2.39)	0.180
fluv-mirt-venl	0.841	0.515	1.632	0.103	(0.00,1.85)	0.000
bupr-paro-venl	0.828	0.480	1.726	0.084	(0.00,1.77)	0.000
cita-fluv-venl	0.797	0.700	1.138	0.255	(0.00,2.17)	0.000
cita-esci-fluo	0.790	0.507	1.558	0.119	(0.00,1.78)	0.143
fluo-fluv-venl	0.738	0.502	1.469	0.142	(0.00,1.72)	0.000
paro-sert-traz	0.720	0.478	1.507	0.132	(0.00,1.66)	0.000
cita-mirt-venl	0.706	0.733	0.963	0.335	(0.00,2.14)	0.102
cita-fluo-fluv	0.696	0.536	1.298	0.194	(0.00,1.75)	0.000
cita-fluv-mirt	0.674	0.551	1.222	0.222	(0.00,1.75)	0.000
amit-sert-traz	0.666	0.502	1.328	0.184	(0.00,1.65)	0.000
cita-esci-sert	0.615	0.559	1.101	0.271	(0.00,1.71)	0.189
cita-esci-mirt-paro	0.607	0.615	0.988	0.323	(0.00,1.81)	0.126
clom-fluo-paro	0.604	0.363	1.663	0.096	(0.00,1.32)	0.063
esci-fluo-paro	0.563	0.291	1.938	0.053	(0.00,1.13)	0.010
fluo-sert-traz	0.535	0.480	1.115	0.265	(0.00,1.47)	0.000
bupr-fluo-sert	0.534	1.180	0.453	0.651	(0.00,2.85)	0.000
amit-fluo-miln	0.526	0.499	1.055	0.292	(0.00,1.50)	0.000
amit-fluv-venl	0.502	0.649	0.773	0.439	(0.00,1.77)	0.043
agom-esci-paro	0.483	0.342	1.413	0.158	(0.00,1.15)	0.018
cita-esci-paro-rebo	0.479	0.792	0.605	0.545	(0.00,2.03)	0.169
bupr-paro-traz	0.474	0.593	0.800	0.424	(0.00,1.64)	0.000
fluv-paro-sert	0.445	0.522	0.852	0.394	(0.00,1.47)	0.000
fluv-mirt-paro	0.433	0.446	0.971	0.332	(0.00,1.31)	0.000
clom-miln-paro	0.432	0.722	0.598	0.550	(0.00,1.85)	0.111

cita-fluv-paro-rebo	0.431	0.661	0.652	0.514	(0.00,1.73)	0.000
esci-paro-venl	0.427	0.503	0.849	0.396	(0.00,1.41)	0.082
esci-sert-venl	0.418	0.401	1.043	0.297	(0.00,1.20)	0.025
clom-fluo-sert	0.400	0.373	1.072	0.284	(0.00,1.13)	0.000
amit-miln-paro	0.380	0.405	0.937	0.349	(0.00,1.17)	0.000
agom-fluo-venl	0.368	0.237	1.550	0.121	(0.00,0.83)	0.000
mirt-paro-venl	0.366	0.416	0.880	0.379	(0.00,1.18)	0.048
amit-nefa-paro	0.362	0.754	0.480	0.631	(0.00,1.84)	0.000
bupr-fluo-traz	0.359	0.636	0.565	0.572	(0.00,1.61)	0.000
amit-paro-venl	0.352	0.409	0.861	0.389	(0.00,1.15)	0.031
fluo-fluv-sert	0.347	0.429	0.808	0.419	(0.00,1.19)	0.000
agom-dulo-paro	0.343	0.381	0.900	0.368	(0.00,1.09)	0.022
amit-fluo-nefa	0.327	0.775	0.421	0.674	(0.00,1.85)	0.000
esci-fluo-venl	0.323	0.289	1.120	0.263	(0.00,0.89)	0.000
esci-paro-sert	0.319	0.324	0.987	0.324	(0.00,0.95)	0.002
paro-sert-venl	0.312	0.573	0.544	0.586	(0.00,1.43)	0.143
amit-fluv-sert	0.310	0.442	0.702	0.483	(0.00,1.18)	0.000
clom-fluv-paro	0.309	0.801	0.386	0.700	(0.00,1.88)	0.145
amit-clom-miln-venl	0.307	0.769	0.400	0.689	(0.00,1.81)	0.000
fluo-sert-venl	0.294	0.244	1.202	0.229	(0.00,0.77)	0.000
cita-fluo-rebo	0.293	0.380	0.772	0.440	(0.00,1.04)	0.000
fluo-nefa-sert	0.291	0.541	0.538	0.591	(0.00,1.35)	0.000
amit-clom-miln-sert	0.289	0.624	0.463	0.643	(0.00,1.51)	0.000
nefa-paro-sert	0.286	0.533	0.537	0.592	(0.00,1.33)	0.000
amit-clom-fluv-miln	0.280	0.877	0.320	0.749	(0.00,2.00)	0.056
fluo-mirt-paro	0.268	0.256	1.046	0.296	(0.00,0.77)	0.000
clom-paro-venl	0.262	0.917	0.286	0.775	(0.00,2.06)	0.238
bupr-fluo-paro	0.251	0.583	0.430	0.667	(0.00,1.39)	0.005
cita-rebo-venl	0.248	0.539	0.461	0.645	(0.00,1.30)	0.000
dulo-esci-paro	0.247	0.299	0.827	0.408	(0.00,0.83)	0.010
mirt-sert-venl	0.242	0.507	0.477	0.633	(0.00,1.23)	0.069
amit-fluo-traz	0.238	0.434	0.547	0.584	(0.00,1.09)	0.000
bupr-paro-sert	0.235	1.170	0.200	0.841	(0.00,2.53)	0.000
dulo-paro-venl	0.232	0.455	0.509	0.611	(0.00,1.12)	0.055
fluo-rebo-venl	0.214	0.454	0.470	0.638	(0.00,1.10)	0.000
esci-fluo-sert	0.201	0.325	0.618	0.537	(0.00,0.84)	0.000
fluv-mirt-sert	0.188	0.547	0.343	0.731	(0.00,1.26)	0.051
cita-mirt-paro-rebo	0.185	0.456	0.405	0.685	(0.00,1.08)	0.006
mirt-paro-sert	0.175	0.317	0.554	0.580	(0.00,0.80)	0.001
cita-mirt-sert	0.173	0.650	0.266	0.790	(0.00,1.45)	0.112
amit-fluo-venl	0.146	0.323	0.454	0.650	(0.00,0.78)	0.000
cita-fluo-mirt	0.143	0.360	0.398	0.690	(0.00,0.85)	0.004
amit-nefa-sert	0.142	0.781	0.182	0.856	(0.00,1.67)	0.000
fluo-paro-traz	0.137	0.396	0.347	0.729	(0.00,0.91)	0.000
clom-fluo-venl	0.129	0.536	0.240	0.811	(0.00,1.18)	0.000
cita-paro-rebo-sert	0.128	0.562	0.228	0.820	(0.00,1.23)	0.048
clom-paro-sert	0.121	0.563	0.215	0.830	(0.00,1.22)	0.114
dulo-esci-venl	0.120	0.305	0.394	0.694	(0.00,0.72)	0.000
fluo-fluv-mirt	0.115	0.366	0.316	0.752	(0.00,0.83)	0.000
amit-fluo-sert	0.106	0.265	0.401	0.688	(0.00,0.63)	0.000
clom-fluv-venl	0.093	0.810	0.114	0.909	(0.00,1.68)	0.000
amit-fluo-paro	0.088	0.220	0.402	0.688	(0.00,0.52)	0.000
agom-fluo-paro	0.080	0.285	0.282	0.778	(0.00,0.64)	0.021
fluo-paro-rebo	0.078	0.365	0.215	0.830	(0.00,0.79)	0.000
amit-bupr-traz-venl	0.076	0.606	0.125	0.901	(0.00,1.26)	0.000
amit-fluo-fluv	0.069	0.384	0.181	0.857	(0.00,0.82)	0.000
fluo-paro-sert	0.069	0.254	0.272	0.785	(0.00,0.57)	0.000
amit-paro-sert	0.066	0.271	0.244	0.808	(0.00,0.60)	0.000
amit-fluv-paro	0.062	0.484	0.128	0.898	(0.00,1.01)	0.015
fluo-miln-paro	0.058	0.345	0.168	0.867	(0.00,0.73)	0.000
fluo-nefa-paro	0.053	0.517	0.103	0.918	(0.00,1.07)	0.000
bupr-fluo-venl	0.051	0.490	0.104	0.917	(0.00,1.01)	0.000
fluo-fluv-paro	0.050	0.465	0.108	0.914	(0.00,0.96)	0.000
cita-fluo-sert	0.050	0.291	0.171	0.864	(0.00,0.62)	0.010
fluo-mirt-sert	0.044	0.308	0.142	0.887	(0.00,0.65)	0.000
amit-sert-venl	0.041	0.375	0.110	0.913	(0.00,0.78)	0.001
agom-esci-venl	0.039	0.330	0.118	0.906	(0.00,0.69)	0.000
clom-sert-venl	0.023	0.694	0.033	0.974	(0.00,1.38)	0.080
fluo-mirt-venl	0.012	0.289	0.042	0.966	(0.00,0.58)	0.000
amit-paro-traz	0.012	0.409	0.029	0.977	(0.00,0.81)	0.003
agom-esci-fluo	0.005	0.310	0.017	0.986	(0.00,0.61)	0.000

8.3.4.2 *Side-splitting*

Side	Direct Coef.	Std. Err.	Indirect Coef.	Std. Err.	Difference Coef.	Std. Err.	P>|z|
agom dulo	.6234503	.2592641	-.0729466	.1430137	.6963969	.2960926	0.019
agom esci	-.2207425	.2303707	.2382185	.1287231	-.458961	.2638932	0.082
agom fluo	.0370261	.1725256	-.1067063	.1276805	.1437324	.2145508	0.503
agom paro	.19437	.1950423	.0059383	.1237384	.1884317	.2310855	0.415
agom venl	-.2595933	.2023589	.182695	.1211126	-.4422883	.2358413	0.061
amit fluo	-.1433022	.159218	-.1483395	.1042078	.0050373	.1904468	0.979
amit fluv	-.0600327	.2709023	-.0711222	.1542565	.0110895	.3115862	0.972
amit miln	-.4157296	.399406	-.0749736	.164075	-.340756	.4317937	0.430
amit nefa	.0953102	.6811125	-.2002606	.2327999	.2955708	.7197986	0.681
amit paro	-.0102981	.122258	-.0495495	.1146336	.0392513	.1676749	0.815
amit sert	-.1118952	.1957148	-.1034456	.1150287	-.0084496	.2270131	0.970
amit traz	-.3104324	.3156806	-.1409792	.1849787	-.1694532	.3658706	0.643
amit venl	.0626154	.2929606	-.0366823	.1049696	.0992978	.311206	0.750
bupr fluo	-.4560423	.4467389	-.4547051	.2084543	-.0013371	.4929797	0.998
bupr paro	.0285874	.4007722	-.4448935	.2142873	.4734809	.4544638	0.297
bupr sert	.2231389	1.10581	-.4347678	.200193	.6579067	1.123785	0.558
bupr traz	-.7463913	.4157567	-.3945967	.2581918	-.3517946	.4894044	0.472
bupr venl	-.4392146	.2829709	-.2495941	.2517055	-.1896205	.3787192	0.617
cita clom	1.298033	.4297473	.0577907	.138904	1.240243	.4516382	0.006
cita esci	.4928984	.1173185	-.1213499	.1382455	.6142483	.18149	0.001
cita fluo	-.0584436	.1914976	.0917294	.110443	-.1501731	.221089	0.497
cita fluv	-.5833963	.4693608	.2066134	.150065	-.7900097	.4927667	0.109
cita mirt	-.0870114	.2986062	.2002014	.1327638	-.2872127	.3267903	0.379
cita rebo	-.4534166	.2685338	-.0432042	.1990596	-.4102124	.3342681	0.220
cita sert	.0501854	.206055	.1119497	.123842	-.0617643	.2404041	0.797
cita venl	-.7682205	.3517416	.2646464	.1096716	-1.032867	.3690367	0.005
clom fluo	.3421894	.2431069	-.2460549	.1275312	.5882444	.2743276	0.032
clom fluv	-.4363221	.5106611	-.0021753	.162141	-.4341468	.5357873	0.418
clom miln	-.498664	.418871	-.0256673	.1777178	-.4729968	.4550126	0.29
clom paro	-.0319974	.148244	.0253304	.1569356	-.0573279	.2158805	0.791
clom sert	.0911531	.2859228	-.1182206	.1362064	.2093737	.3167069	0.509
clom venl	.2876819	.5005675	-.0176248	.1262816	.3053067	.5162507	0.554
dulo esci	.2420548	.2069254	-.063441	.147557	.3054958	.2541282	0.229
dulo paro	-.0878558	.1875057	.0062097	.1441552	-.0940655	.23652	0.691
dulo venl	.1272084	.1882259	-.1108109	.1441451	.2380193	.2370676	0.315
esci fluo	.2536616	.2184839	-.2663022	.0964278	.5199638	.2387526	0.029
esci paro	-.0669473	.1828704	-.0680956	.1061064	.0011484	.2114458	0.996
esci sert	.1976214	.2499216	-.2102412	.1119537	.4078626	.2738098	0.136
esci venl	.0028297	.2238301	-.0764378	.106041	.0792675	.2476641	0.749
fluo fluv	.1689046	.2761632	.0552206	.1400451	.1136839	.3096127	0.713
fluo miln	.2608349	.3363229	-.0319458	.160884	.2927807	.3728228	0.432
fluo mirt	.1366917	.1966925	.0854065	.116125	.0512852	.2283761	0.822
fluo nefa	-.0740596	.3825706	.000529	.256416	-.0745886	.460532	0.871
fluo paro	.2364097	.1287154	.0695953	.0790252	.1668144	.1509597	0.269
fluo rebo	-.1039729	.2865555	-.298179	.1822568	.1942061	.3397262	0.568
fluo sert	.1509848	.146175	-.0092996	.098973	.1602844	.1764611	0.364
fluo traz	.0607136	.2959498	-.0739122	.1800549	.1346259	.346533	0.698
fluo venl	.1026671	.11057	.136408	.0965059	-.0337408	.1464736	0.818
fluv mirt	-.1767502	.2254925	.1278427	.1672736	-.3045929	.2807534	0.278
fluv paro	.107648	.3875086	.0285051	.1341185	.0791429	.4100911	0.847
fluv sert	-.3744894	.3387209	.0240879	.1451651	-.3985773	.3685026	0.279
fluv venl	.6321095	.4499498	-.0136255	.138065	.6457349	.4706555	0.170
miln paro	.0356936	.1713673	.1959236	.2279966	-.16023	.2851266	0.574
mirt paro	-.1288186	.1637282	.100516	.1238685	-.2293346	.2054311	0.264
mirt sert	-.0098147	.2672884	-.0674565	.1230049	.0576418	.2942334	0.845
mirt venl	-.0298415	.2405777	.0361494	.1200942	-.0659909	.2688693	0.806
nefa paro	.2704112	.3601378	.0669878	.264014	.2034234	.4465036	0.649
nefa sert	-.0655973	.4017572	.1164611	.2565356	-.1820584	.4766753	0.703
paro rebo	-.4105341	.272158	-.3315163	.1896937	-.0790179	.3317434	0.812
paro sert	-.0279577	.2085221	-.0841285	.0948523	.0561709	.2291453	0.806
paro traz	-.2905221	.2754681	-.0915147	.1842626	-.1990073	.3315104	0.548
paro venl	.4098544	.2264543	-.0517998	.0846832	.4616542	.2416861	0.056
rebo venl	-.04652	.3897696	.4449159	.1732643	-.4914359	.4265453	0.249
sert traz	.4587335	.3943884	-.1857429	.1759788	.6444764	.4318689	0.136
sert venl	.219623	.206775	.0457009	.1028339	.1739221	.2309029	0.451

8.3.4.3 *Design-by-treatment test*

chi2(56) = 69.35

Prob > chi2 = 0.0652

8.3.5 Dropouts due to adverse events

8.3.5.1 *Loop-specific approach*

Loops With Evidence of Statistical Inconsistency:

Loop	IF	seIF	z_value	p_value	CI_95	Loop_Heterog_tau2
mirt-sert-traz	3.232	1.092	2.960	0.003	(1.09,5.37)	0.000
paro-sert-traz	2.189	1.058	2.069	0.039	(0.12,4.26)	0.000
Plac-mirt-sert	1.997	0.690	2.892	0.004	(0.64,3.35)	0.126
agom-dulo-venl	1.909	0.587	3.252	0.001	(0.76,3.06)	0.000
agom-dulo-esci	1.904	0.792	2.405	0.016	(0.35,3.46)	0.087
cita-fluo-venl	1.876	0.933	2.011	0.044	(0.05,3.70)	0.020
amit-mirt-sert	1.673	0.602	2.780	0.005	(0.49,2.85)	0.000
dulo-venl-vort	1.577	0.427	3.694	0.000	(0.74,2.41)	0.017
esci-paro-venl	1.297	0.579	2.240	0.025	(0.16,2.43)	0.000
agom-paro-venl	1.278	0.489	2.612	0.009	(0.32,2.24)	0.000
amit-fluo-sert	1.270	0.439	2.896	0.004	(0.41,2.13)	0.029
Plac-fluv-mirt	1.199	0.428	2.803	0.005	(0.36,2.04)	0.000
Plac-dulo-venl	1.025	0.338	3.032	0.002	(0.36,1.69)	0.037
amit-fluo-paro	0.795	0.340	2.338	0.019	(0.13,1.46)	0.000
Plac-dulo-esci	0.675	0.300	2.252	0.024	(0.09,1.26)	0.000
Plac-paro-venl	0.583	0.297	1.962	0.050	(0.00,1.17)	0.009

Loops Without Evidence of Statistical Inconsistency:

Loop	IF	seIF	z_value	p_value	CI_95	Loop_Heterog_tau2
Plac-cita-clom	3.244	1.849	1.755	0.079	(0.00,6.87)	0.000
cita-clom-fluv	2.428	1.998	1.216	0.224	(0.00,6.34)	0.000
clom-fluv-mirt-venl	2.094	1.436	1.458	0.145	(0.00,4.91)	0.000
clom-fluo-fluv	1.985	1.700	1.167	0.243	(0.00,5.32)	0.000
bupr-esci-sert-traz	1.957	1.789	1.094	0.274	(0.00,5.46)	0.658
Plac-clom-sert	1.904	1.254	1.517	0.129	(0.00,4.36)	0.141
Plac-clom-fluo	1.879	1.209	1.555	0.120	(0.00,4.25)	0.058
amit-clom-fluv-venl	1.798	1.489	1.207	0.227	(0.00,4.72)	0.000
Plac-cita-venl	1.677	0.908	1.847	0.065	(0.00,3.46)	0.057
Plac-clom-venl	1.622	1.279	1.269	0.205	(0.00,4.13)	0.104
fluo-sert-traz	1.604	0.990	1.620	0.105	(0.00,3.54)	0.000
cita-fluo-sert	1.584	0.977	1.622	0.105	(0.00,3.50)	0.000
cita-mirt-sert	1.570	1.196	1.313	0.189	(0.00,3.91)	0.000
cita-clom-fluo	1.544	1.614	0.957	0.339	(0.00,4.71)	0.000
agom-dulo-fluo	1.533	1.172	1.307	0.191	(0.00,3.83)	0.000
Plac-sert-traz	1.501	1.002	1.497	0.134	(0.00,3.47)	0.132
cita-paro-venl	1.472	0.994	1.481	0.139	(0.00,3.42)	0.000
bupr-paro-venl	1.417	0.756	1.875	0.061	(0.00,2.90)	0.000
Plac-clom-paro	1.375	1.075	1.279	0.201	(0.00,3.48)	0.000
amit-sert-traz	1.295	0.961	1.347	0.178	(0.00,3.18)	0.006
Plac-amit-clom-miln	1.263	1.688	0.748	0.454	(0.00,4.57)	0.267
amit-fluv-sert	1.252	0.948	1.320	0.187	(0.00,3.11)	0.073
amit-bupr-fluo	1.232	1.410	0.873	0.382	(0.00,4.00)	0.223
mirt-paro-sert	1.221	0.772	1.581	0.114	(0.00,2.73)	0.000
fluv-mirt-paro	1.202	0.843	1.427	0.154	(0.00,2.85)	0.165
cita-esci-venl	1.170	0.971	1.204	0.228	(0.00,3.07)	0.043
Plac-dulo-fluo	1.154	1.121	1.030	0.303	(0.00,3.35)	0.038
agom-esci-fluo	1.142	0.589	1.940	0.052	(0.00,2.30)	0.000
Plac-cita-mirt	1.130	0.708	1.595	0.111	(0.00,2.52)	0.025
cita-esci-sert	1.130	1.064	1.062	0.288	(0.00,3.21)	0.055

cita-fluo-fluv	1.100	1.128	0.976	0.329	(0.00,3.31)	0.000
amit-bupr-venl	1.095	1.323	0.828	0.408	(0.00,3.69)	0.000
cita-clom-paro	1.054	1.660	0.635	0.525	(0.00,4.31)	0.041
mirt-sert-venl	1.049	0.696	1.507	0.132	(0.00,2.41)	0.000
clom-fluv-paro	1.038	1.687	0.616	0.538	(0.00,4.34)	0.231
cita-fluv-sert	1.024	1.238	0.827	0.408	(0.00,3.45)	0.000
cita-esci-paro	1.020	0.667	1.529	0.126	(0.00,2.33)	0.000
clom-fluo-miln	1.001	0.813	1.231	0.218	(0.00,2.59)	0.000
amit-fluv-paro	0.991	0.640	1.548	0.122	(0.00,2.25)	0.000
clom-fluv-sert	0.986	2.541	0.388	0.698	(0.00,5.97)	1.291
dulo-esci-paro	0.960	0.504	1.904	0.057	(0.00,1.95)	0.000
Plac-nefa-sert	0.950	0.600	1.581	0.114	(0.00,2.13)	0.074
dulo-fluo-paro	0.943	1.115	0.846	0.398	(0.00,3.13)	0.000
amit-fluo-miln	0.935	0.960	0.975	0.330	(0.00,2.82)	0.070
Plac-paro-sert	0.933	0.561	1.663	0.096	(0.00,2.03)	0.003
amit-bupr-traz	0.885	1.323	0.669	0.503	(0.00,3.48)	0.000
Plac-amit-bupr	0.884	1.259	0.702	0.483	(0.00,3.35)	0.132
amit-fluo-traz	0.862	0.777	1.109	0.267	(0.00,2.39)	0.101
esci-fluo-paro	0.837	0.503	1.663	0.096	(0.00,1.82)	0.000
cita-fluo-mirt	0.830	0.743	1.116	0.264	(0.00,2.29)	0.000
Plac-cita-paro	0.824	0.526	1.567	0.117	(0.00,1.86)	0.000
fluo-mirt-sert	0.815	0.620	1.315	0.189	(0.00,2.03)	0.000
fluo-mirt-traz	0.812	0.704	1.153	0.249	(0.00,2.19)	0.000
cita-fluo-paro	0.806	0.591	1.363	0.173	(0.00,1.96)	0.000
dulo-esci-fluo	0.799	1.186	0.674	0.500	(0.00,3.12)	0.037
amit-fluv-miln	0.794	0.994	0.799	0.425	(0.00,2.74)	0.000
cita-paro-sert	0.794	1.134	0.700	0.484	(0.00,3.02)	0.000
cita-mirt-venl	0.788	1.067	0.738	0.460	(0.00,2.88)	0.000
fluo-traz-venl	0.773	0.671	1.152	0.249	(0.00,2.09)	0.030
clom-fluo-sert	0.762	0.684	1.115	0.265	(0.00,2.10)	0.000
dulo-esci-venl	0.745	0.671	1.110	0.267	(0.00,2.06)	0.114
Plac-fluo-sert	0.736	0.377	1.951	0.051	(0.00,1.48)	0.072
Plac-sert-venl	0.724	0.515	1.406	0.160	(0.00,1.73)	0.095
cita-clom-sert	0.722	1.788	0.404	0.686	(0.00,4.23)	0.000
amit-cita-fluv-venl	0.690	1.069	0.645	0.519	(0.00,2.78)	0.000
agom-esci-venl	0.690	0.694	0.994	0.320	(0.00,2.05)	0.000
bupr-traz-venl	0.681	0.714	0.954	0.340	(0.00,2.08)	0.000
Plac-agom-esci	0.680	0.526	1.292	0.196	(0.00,1.71)	0.000
fluo-fluv-paro	0.672	1.193	0.563	0.573	(0.00,3.01)	0.044
agom-esci-paro	0.671	0.659	1.019	0.308	(0.00,1.96)	0.000
Plac-cita-sert	0.671	0.960	0.699	0.485	(0.00,2.55)	0.053
Plac-amit-fluv	0.671	0.446	1.505	0.132	(0.00,1.54)	0.062
paro-rebo-venl	0.666	1.011	0.659	0.510	(0.00,2.65)	0.336
fluo-miln-paro	0.665	0.425	1.566	0.117	(0.00,1.50)	0.000
Plac-esci-sert	0.649	0.520	1.248	0.212	(0.00,1.67)	0.046
amit-sert-venl	0.648	0.652	0.994	0.320	(0.00,1.93)	0.000
esci-paro-sert	0.644	0.801	0.805	0.421	(0.00,2.21)	0.000
Plac-desv-dulo	0.637	0.396	1.609	0.108	(0.00,1.41)	0.000
Plac-fluv-sert	0.636	0.867	0.734	0.463	(0.00,2.34)	0.068
amit-fluo-venl	0.634	0.603	1.050	0.294	(0.00,1.82)	0.050
cita-esci-fluo	0.623	0.488	1.276	0.202	(0.00,1.58)	0.000
Plac-fluo-fluv	0.605	1.053	0.574	0.566	(0.00,2.67)	0.019
amit-clom-miln-sert	0.595	1.119	0.531	0.595	(0.00,2.79)	0.052
clom-fluv-miln	0.588	1.490	0.395	0.693	(0.00,3.51)	0.000
Plac-amit-fluo	0.578	0.374	1.544	0.123	(0.00,1.31)	0.131
Plac-bupr-paro	0.574	0.627	0.915	0.360	(0.00,1.80)	0.000
fluo-paro-traz	0.570	0.603	0.944	0.345	(0.00,1.75)	0.000
Plac-agom-dulo	0.567	0.429	1.323	0.186	(0.00,1.41)	0.000
Plac-fluo-traz	0.563	0.602	0.935	0.350	(0.00,1.74)	0.079
bupr-paro-traz	0.553	0.828	0.668	0.504	(0.00,2.18)	0.000
amit-fluo-fluv	0.547	1.125	0.486	0.627	(0.00,2.75)	0.026
bupr-fluo-paro	0.540	0.720	0.750	0.453	(0.00,1.95)	0.009
Plac-fluo-rebo	0.518	0.344	1.508	0.132	(0.00,1.19)	0.050
clom-sert-venl	0.514	0.790	0.651	0.515	(0.00,2.06)	0.000
amit-miln-paro	0.511	0.809	0.632	0.528	(0.00,2.10)	0.000
Plac-cita-vila	0.493	0.452	1.092	0.275	(0.00,1.38)	0.000
agom-fluo-venl	0.492	0.441	1.116	0.265	(0.00,1.36)	0.000
fluo-fluv-sert	0.482	1.669	0.289	0.773	(0.00,3.75)	0.194
amit-paro-sert	0.480	0.575	0.835	0.404	(0.00,1.61)	0.000
Plac-clom-fluv	0.479	1.661	0.288	0.773	(0.00,3.73)	0.000
amit-traz-venl	0.471	0.759	0.621	0.535	(0.00,1.96)	0.000
bupr-fluo-venl	0.466	0.629	0.742	0.458	(0.00,1.70)	0.048
cita-fluv-paro	0.452	0.800	0.565	0.572	(0.00,2.02)	0.000
bupr-fluo-traz	0.446	1.059	0.421	0.674	(0.00,2.52)	0.301
cita-mirt-paro	0.445	0.820	0.543	0.587	(0.00,2.05)	0.000
Plac-nefa-paro	0.445	0.448	0.993	0.321	(0.00,1.32)	0.000
Plac-fluo-mirt	0.439	0.362	1.214	0.225	(0.00,1.15)	0.073

Plac-agom-paro	0.433	0.274	1.580	0.114	(0.00,0.97)	0.000
fluo-mirt-paro	0.421	0.358	1.176	0.240	(0.00,1.12)	0.000
fluo-paro-venl	0.420	0.308	1.363	0.173	(0.00,1.02)	0.000
clom-paro-sert	0.420	0.820	0.513	0.608	(0.00,2.03)	0.077
amit-clom-miln-venl	0.416	1.184	0.351	0.725	(0.00,2.74)	0.000
Plac-amit-mirt	0.415	0.431	0.963	0.335	(0.00,1.26)	0.091
amit-paro-traz	0.411	0.591	0.695	0.487	(0.00,1.57)	0.000
dulo-paro-venl	0.408	0.401	1.015	0.310	(0.00,1.19)	0.000
amit-fluo-mirt	0.402	0.455	0.885	0.376	(0.00,1.29)	0.000
nefa-paro-sert	0.401	0.791	0.507	0.612	(0.00,1.95)	0.000
fluo-fluv-miln	0.395	1.212	0.326	0.744	(0.00,2.77)	0.000
cita-fluv-mirt	0.395	0.794	0.497	0.619	(0.00,1.95)	0.000
fluo-nefa-paro	0.390	0.723	0.540	0.589	(0.00,1.81)	0.000
fluv-miln-paro	0.387	1.059	0.366	0.715	(0.00,2.46)	0.133
Plac-esci-paro	0.384	0.433	0.887	0.375	(0.00,1.23)	0.000
agom-fluo-paro	0.366	0.311	1.175	0.240	(0.00,0.98)	0.000
Plac-bupr-esci	0.358	0.470	0.763	0.445	(0.00,1.28)	0.000
Plac-esci-venl	0.357	0.419	0.850	0.395	(0.00,1.18)	0.047
clom-paro-venl	0.356	0.618	0.576	0.565	(0.00,1.57)	0.000
Plac-amit-paro	0.343	0.246	1.398	0.162	(0.00,0.82)	0.012
Plac-venl-vort	0.342	0.366	0.935	0.350	(0.00,1.06)	0.048
Plac-cita-fluv	0.337	0.446	0.756	0.449	(0.00,1.21)	0.000
Plac-esci-fluo	0.328	0.323	1.014	0.311	(0.00,0.96)	0.000
Plac-agom-venl	0.323	0.426	0.757	0.449	(0.00,1.16)	0.005
clom-fluo-paro	0.318	0.536	0.592	0.554	(0.00,1.37)	0.000
amit-fluv-mirt	0.273	0.537	0.509	0.611	(0.00,1.32)	0.000
bupr-esci-fluo	0.271	0.830	0.327	0.744	(0.00,1.90)	0.227
cita-sert-venl	0.267	1.286	0.208	0.835	(0.00,2.79)	0.000
Plac-amit-traz	0.260	0.703	0.370	0.711	(0.00,1.64)	0.214
amit-mirt-traz	0.260	0.703	0.371	0.711	(0.00,1.64)	0.000
clom-fluo-venl	0.256	0.745	0.344	0.731	(0.00,1.72)	0.004
Plac-bupr-traz	0.252	0.559	0.450	0.653	(0.00,1.35)	0.010
esci-sert-venl	0.251	0.727	0.346	0.729	(0.00,1.68)	0.030
Plac-bupr-venl	0.247	0.517	0.478	0.632	(0.00,1.26)	0.061
amit-paro-venl	0.243	0.558	0.435	0.663	(0.00,1.34)	0.000
fluo-mirt-venl	0.239	0.350	0.684	0.494	(0.00,0.92)	0.000
Plac-dulo-vort	0.239	0.275	0.870	0.385	(0.00,0.78)	0.032
fluo-rebo-venl	0.239	0.685	0.349	0.727	(0.00,1.58)	0.035
mirt-paro-venl	0.238	0.408	0.584	0.559	(0.00,1.04)	0.000
mirt-traz-venl	0.234	0.667	0.352	0.725	(0.00,1.54)	0.000
Plac-paro-traz	0.232	0.472	0.490	0.624	(0.00,1.16)	0.000
Plac-mirt-venl	0.230	0.396	0.581	0.561	(0.00,1.00)	0.088
Plac-fluo-nefa	0.229	0.663	0.345	0.730	(0.00,1.53)	0.044
agom-dulo-paro	0.223	0.471	0.474	0.635	(0.00,1.15)	0.000
Plac-bupr-fluo	0.219	0.467	0.469	0.639	(0.00,1.13)	0.072
Plac-cita-esci	0.217	0.347	0.624	0.533	(0.00,0.90)	0.000
Plac-fluo-paro	0.215	0.187	1.149	0.251	(0.00,0.58)	0.000
Plac-paro-rebo	0.209	0.321	0.650	0.516	(0.00,0.84)	0.068
esci-fluo-sert	0.208	0.585	0.355	0.722	(0.00,1.36)	0.000
Plac-rebo-venl	0.202	0.727	0.277	0.782	(0.00,1.63)	0.129
Plac-mirt-traz	0.201	0.683	0.294	0.769	(0.00,1.54)	0.125
Plac-cita-fluo	0.198	0.432	0.457	0.648	(0.00,1.04)	0.037
Plac-agom-fluo	0.189	0.278	0.680	0.496	(0.00,0.73)	0.000
paro-traz-venl	0.183	0.604	0.303	0.762	(0.00,1.37)	0.000
mirt-paro-traz	0.179	0.651	0.275	0.784	(0.00,1.45)	0.000
Plac-traz-venl	0.176	0.638	0.275	0.783	(0.00,1.43)	0.114
fluv-mirt-sert	0.145	1.870	0.078	0.938	(0.00,3.81)	0.623
Plac-amit-sert	0.142	0.345	0.413	0.680	(0.00,0.82)	0.091
fluo-fluv-mirt	0.124	1.102	0.113	0.910	(0.00,2.28)	0.000
dulo-fluo-venl	0.118	1.135	0.104	0.917	(0.00,2.34)	0.006
Plac-mirt-paro	0.117	0.313	0.375	0.708	(0.00,0.73)	0.000
amit-bupr-paro	0.079	1.324	0.060	0.952	(0.00,2.67)	0.000
cita-clom-venl	0.060	1.802	0.033	0.974	(0.00,3.59)	0.000
esci-fluo-venl	0.059	0.476	0.125	0.901	(0.00,0.99)	0.018
amit-bupr-esci-sert	0.055	1.492	0.037	0.970	(0.00,2.98)	0.130
Plac-dulo-paro	0.045	0.241	0.188	0.851	(0.00,0.52)	0.000
fluo-paro-rebo	0.045	0.454	0.100	0.921	(0.00,0.94)	0.130
Plac-fluo-venl	0.033	0.247	0.135	0.893	(0.00,0.52)	0.078
amit-mirt-paro	0.028	0.398	0.071	0.944	(0.00,0.81)	0.000
Plac-amit-venl	0.027	0.602	0.045	0.964	(0.00,1.21)	0.158
Plac-fluv-paro	0.024	0.574	0.042	0.967	(0.00,1.15)	0.000
amit-mirt-venl	0.024	0.592	0.040	0.968	(0.00,1.18)	0.000
clom-miln-paro	0.018	0.615	0.029	0.977	(0.00,1.22)	0.000
bupr-esci-paro	0.017	1.202	0.014	0.989	(0.00,2.37)	0.364
fluo-paro-sert	0.016	0.606	0.026	0.979	(0.00,1.20)	0.000
bupr-esci-venl	0.015	1.355	0.011	0.991	(0.00,2.67)	0.654
fluo-sert-venl	0.006	0.511	0.011	0.991	(0.00,1.01)	0.000

fluo-nefa-sert	0.005	0.790	0.006	0.995	(0.00,1.55)	0.000

8.3.5.2 *Side-splitting*

Side	Direct Coef.	Std. Err.	Indirect Coef.	Std. Err.	Difference Coef.	Std. Err.	P>|z|
agom dulo	.071656	.4231495	1.405661	.2628502	-1.334005	.4981423	0.007
agom esci	1.183817	.5066484	-.0648548	.254886	1.248672	.5671449	0.028
agom fluo	.3706843	.286011	.3627269	.2337901	.0079574	.36879	0.983
agom paro	.383754	.3136438	.6267815	.2259473	-.2430274	.3866153	0.530
agom venl	1.299743	.4048458	.715701	.2098939	.5840418	.4558364	0.200
amit bupr	.4834188	1.193323	-.3274338	.3292175	.8108526	1.237904	0.512
amit fluo	-1.07203	.2791787	-.1672649	.1624183	-.9047652	.3230952	0.005
amit fluv	-.5677627	.3420054	-.0500238	.2602087	-.5177389	.4298427	0.228
amit miln	-.5868086	.7981444	-.5337424	.2341838	-.0530663	.8317911	0.949
amit paro	-.0168418	.1767244	-.4596895	.1920549	.4428477	.2584835	0.087
amit sert	-.2152856	.2842755	-.7730865	.2176022	.5578009	.3579508	0.119
amit traz	-.0859177	.471326	-.1143251	.285858	.0284074	.551027	0.959
amit venl	.0054928	.5012419	.0829282	.1720514	-.0774353	.5299573	0.884
bupr paro	-.5487831	.6374914	.2260854	.3483917	-.7748685	.7264795	0.286
bupr traz	.326327	.4962342	.0360934	.4380122	.2902337	.6618935	0.661
bupr venl	.6167637	.4548642	.1193331	.414267	.4974305	.6152384	0.419
cita clom	2.269283	1.519873	.8128087	.2834529	1.456474	1.546078	0.346
cita esci	-.4432333	.4348477	.1032593	.3250728	-.5464926	.5437309	0.315
cita fluo	-.4855696	.3610326	.4249282	.2820119	-.9104978	.4580013	0.047
cita fluv	.588455	.4144032	.0296754	.3266222	.5587796	.5276476	0.290
cita mirt	.6931459	.6654509	.193572	.2700347	.4995739	.7181529	0.487
cita sert	.8615676	.9047157	-.170473	.2701657	1.032041	.9435401	0.274
cita venl	1.85453	.8654943	.452154	.2465058	1.402376	.8999133	0.119
clom fluo	-1.195107	.5183874	-.7146526	.2088795	-.4804544	.558886	0.390
clom fluv	.747199	1.288331	-.6752484	.2608237	1.422447	1.314468	0.279
clom miln	-.6505839	.5973387	-.973166	.2742317	.322582	.6572796	0.624
clom paro	-.5825751	.2341298	-.6320714	.2759904	.0494963	.3616185	0.891
clom sert	-.8167654	.438163	-.9920341	.2510942	.1752688	.5049326	0.729
clom venl	-.4744552	.5866441	-.2800555	.2196129	-.1943997	.6264034	0.756
dulo esci	-1.061139	.3658429	-.7236774	.3006593	-.3374615	.4737016	0.476
dulo paro	-.1851954	.4138518	-.6200026	.2452327	.4348072	.4810534	0.366
dulo venl	-.6915066	.2857055	.1876045	.2597468	-.879111	.3860944	0.023
esci fluo	.3611136	.4651178	.1302325	.2230887	.2308811	.5158207	0.654
esci paro	-.3045203	.6379104	.4287341	.2169662	-.7332544	.6737983	0.276
esci sert	.1814836	.6962149	-.0163941	.2522738	.1978777	.7407068	0.789
esci venl	.8293866	.3767247	.5811644	.2369215	.2482222	.4445195	0.577
fluo fluv	-.0416727	1.046043	.1711334	.2135561	-.2128061	1.067619	0.842
fluo miln	-.4566506	.3845329	-.001843	.2460835	-.4548076	.4565038	0.319
fluo mirt	.0564188	.3332664	.2263635	.1907598	-.1699447	.3846514	0.659
fluo nefa	.1851866	.6155243	.4474093	.3483146	-.2622226	.7072437	0.711
fluo paro	-.0722141	.2249153	.2756809	.1383221	-.347895	.2641764	0.188
fluo rebo	.2278386	.446757	1.168849	.3729275	-.9410108	.5816663	0.106
fluo sert	-.3597736	.3026713	-.0827884	.1999158	-.2769852	.3620075	0.444
fluo traz	.0803763	.4862792	.3661402	.275688	-.2857639	.5589255	0.609
fluo venl	.5364349	.2017689	.4366562	.1697112	.0997787	.2658142	0.707
fluv miln	-.8220612	.5841142	-.1684841	.2884123	-.6535772	.6510852	0.315
fluv mirt	.5114504	.3760972	-.2259915	.2670842	.7374419	.4612917	0.110
fluv paro	-.3844132	.5626446	.0781584	.2191207	-.4625715	.604305	0.444
fluv sert	-.8418464	.8098143	-.2818538	.2460689	-.5599927	.8464093	0.508
miln paro	.0940082	.2654121	.5627967	.2845691	-.4687885	.3892262	0.228
mirt paro	.3585103	.2705784	-.1929263	.1978386	.5514365	.3342919	0.099
mirt sert	-1.497602	.5478545	-.1739024	.2124937	-1.323699	.5876208	0.024
mirt traz	.9473778	.8792269	.0284872	.2793004	.9188906	.9225231	0.319
mirt venl	.3734289	.277695	.2460855	.2132989	.1273434	.3501583	0.716
nefa paro	-.3140989	.4379402	-.1091499	.4100725	-.204949	.599888	0.733
nefa sert	-.5389955	.5011198	-.5579279	.392667	.0189325	.6366385	0.976
paro rebo	1.329225	.4299984	.0590205	.3747589	1.270205	.5703887	0.026
paro sert	-.6578174	.5735042	-.3172846	.1765423	-.3405328	.5982339	0.569
paro traz	.354115	.4303295	.0159896	.2822347	.3381254	.5138797	0.511
paro venl	-.3671985	.3387162	.4119245	.1442397	-.779123	.3682331	0.034
rebo venl	.0761882	.6217788	-.4126537	.3415725	.4888419	.7094227	0.491
sert traz	-1.203967	.8669622	.6348165	.2792131	-1.838783	.9108145	0.044
sert venl	.8204454	.4443081	.6094643	.1985195	.2109812	.4866533	0.665

8.3.5.3 Design-by-treatment test

chi2(54) = 80.05

Prob > chi2 = 0.0034

8.4 Comparison-adjusted funnel plots (active vs fluoxetine)

We drew comparison-adjusted funnel plots for all drugs against fluoxetine, because this was the most often studied drugs and has been considered standard SSRI. The comparison-adjusted funnel plots against fluoxetine suggested no funnel plot asymmetry.

8.4.1 Response

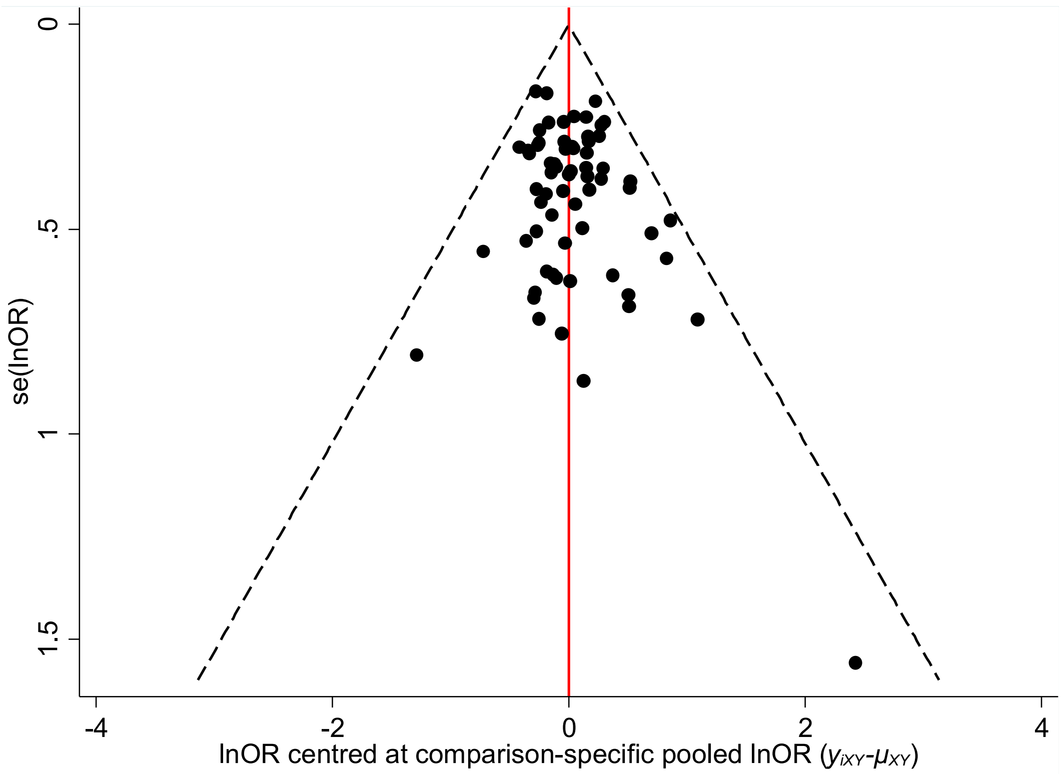

8.4.2 Dropouts for any reason

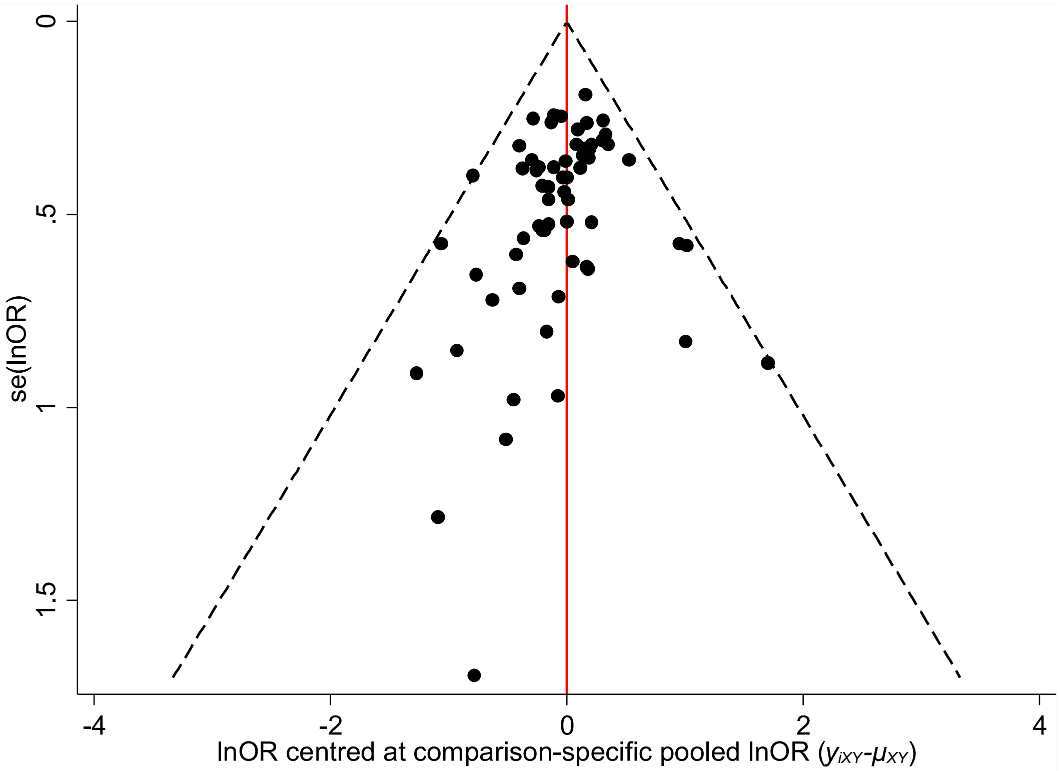

8.4.3 Efficacy continuous

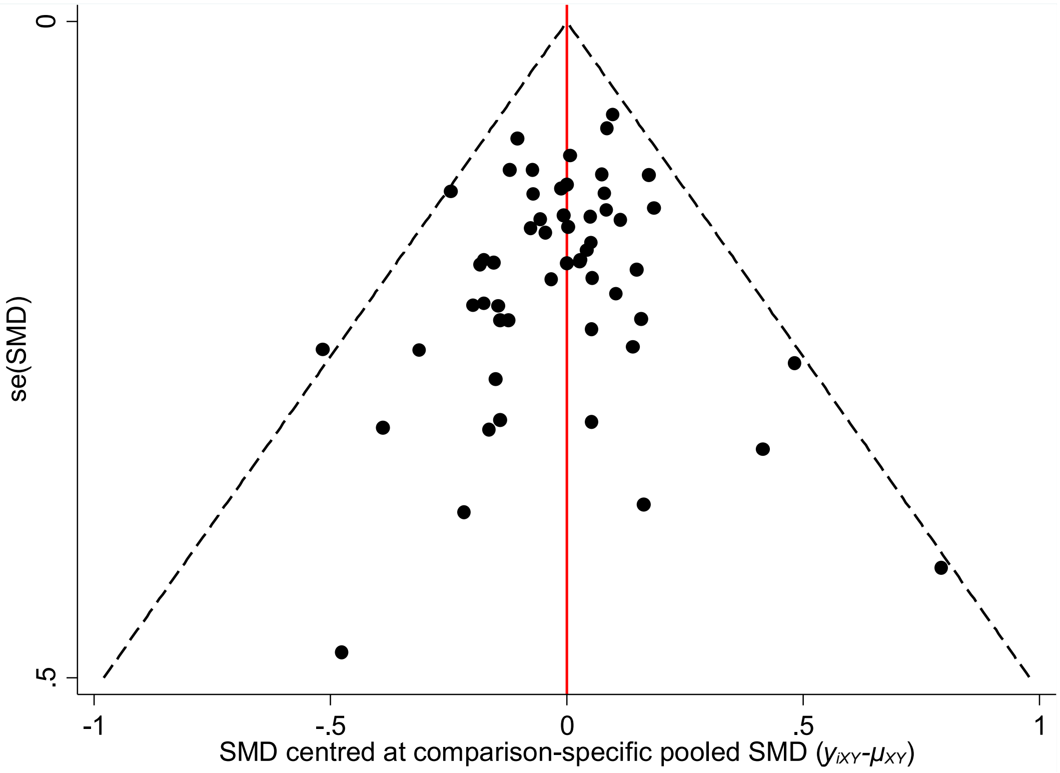

8.4.4 Remission

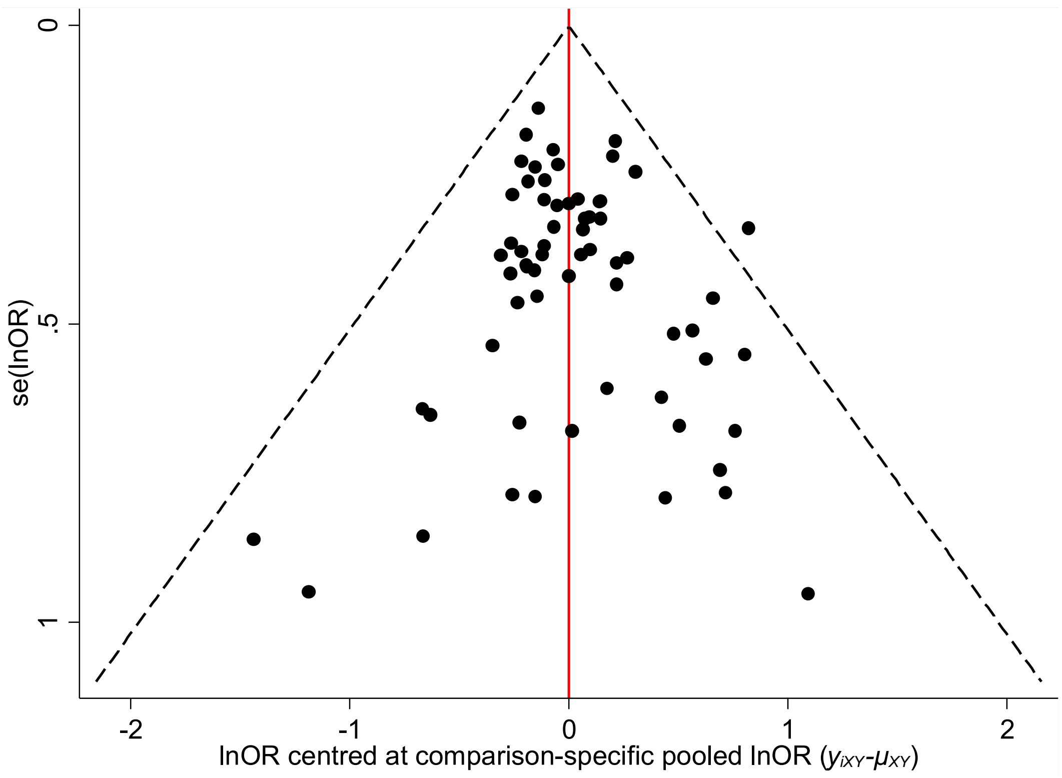

8.4.5 Dropouts due to adverse events

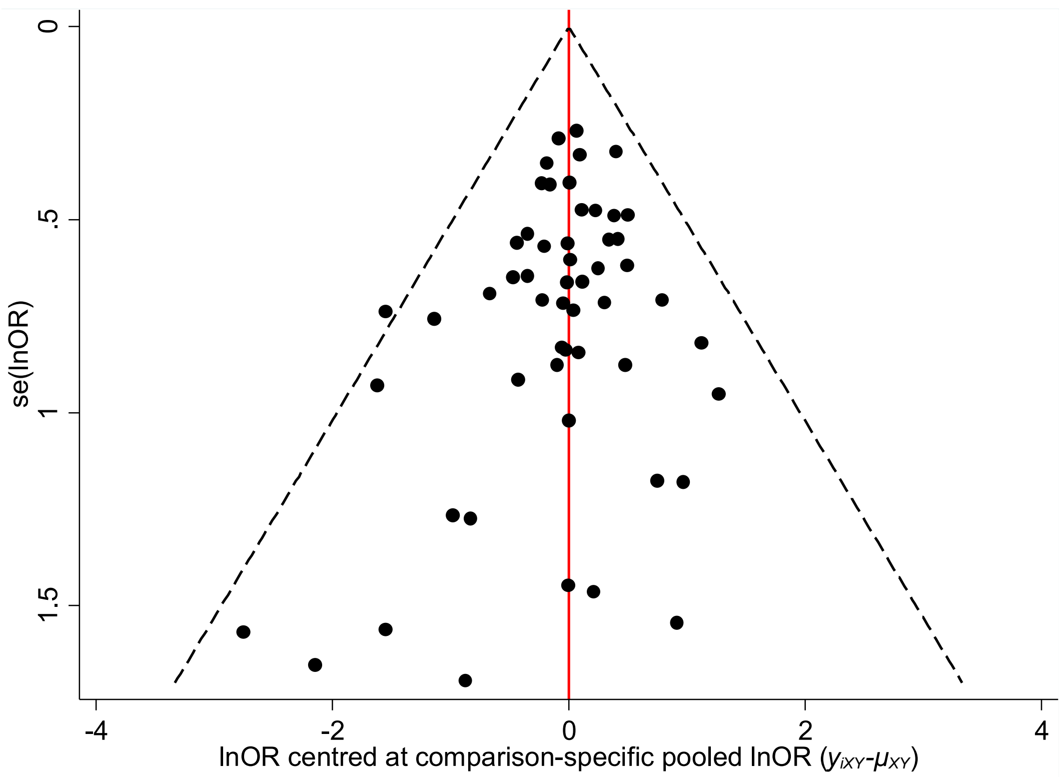

8.5 *Meta-regressions and sensitivity analyses*

8.5.1 Changes in heterogeneity after accounting for potential effect modifiers

The following table shows the evidence about the impact of various potential effect modifiers in the relative efficacy of interventions according to the findings of the meta-regressions. We examined only variance and year as these were the most important potential effect modifiers in the entire network (see section 7.6.2). All basic coefficients (i.e. for every comparison between fluoxetine and any other drug) were assumed exchangeable in the models. None of the mean coefficients were significant: small study-effects: B= 2,99 (1.41, 6.81) for response and B= 0.69 (0.33,1.34) for dropouts year: B= 1.00 (0.98,1.02) for response and B=1.00 (0.98,1.03) for dropouts

Changes in heterogeneity

**Variable**	**Heterogeneity standard deviation median and 95% CrI**	**Relative change in the variance**
**Response**		
None	0.16 (0.04,0.25)	-
Study variance	0.13 (0.01,0.23)	19%
Study year	0.12 (0.01,0.23)	25%
Including all studies with accepted dose range	0.14 (0.03,0.23)	13%
**Dropout**		
None	0.11 (0.01,0.23)	-
Study variance	0.10 (0.00,0.23)	9%
Study year	0.10 (0.00,0.23)	9%
Including all studies with accepted dose range	0.14 (0.02,0.25)	0%

8.5.2 Odds-ratios for response and dropout for any reason adjusted for covariates

8.5.2.1 *Unadjusted*

	Response						Dropouts for any reason					
	OR drug vs PLA			beta of comparison			OR drug vs PLA			beta of comparison		
	2.5%	median	97.5%	2.5%	median	97.5%	2.5%	median	97.5%	2.5 %	median	97.5 %
Agomelatine	1.02	1.44	2.04				0.36	0.53	0.80			
Amitriptyline	1.07	1.50	2.07				0.51	0.74	1.10			
Bupropion	1.05	1.65	2.60				0.42	0.67	1.08			
Citalopram	0.92	1.27	1.75				0.41	0.60	0.87			
Clomipramine	0.84	1.20	1.70				0.62	0.94	1.41			
Duloxetine	0.95	1.36	1.95				0.57	0.86	1.29			
Escitalopram	1.14	1.60	2.23				0.37	0.55	0.81			
Fluoxetine	0.88	1.20	1.62				0.44	0.63	0.90			

Fluvoxamine	0.83	1.20	1.71				0.51	0.77	1.17			
Milnacipran	0.92	1.35	1.95				0.45	0.67	1.03			
Mirtazapine	1.09	1.54	2.17				0.45	0.66	0.99			
Nefazodone	0.77	1.25	2.01				0.43	0.75	1.32			
Paroxetine	1.05	1.43	1.94				0.46	0.66	0.94			
Sertraline	0.96	1.34	1.85				0.43	0.62	0.92			
Trazodone	0.72	1.06	1.56				0.50	0.78	1.23			
Venlafaxine	1.04	1.42	1.94				0.50	0.73	1.06			
Vortioxetine	1.09	1.98	3.59				0.23	0.43	0.81			

8.5.2.2 *Adjusted for small-study effects*

	Response						Dropouts for any reason					
	OR drug vs PLA			beta of comparison			OR drug vs PLA			beta of comparison		
	2.5%	median	97.5%	2.5%	median	97.5%	2.5%	median	97.5%	2.5%	median	97.5%
Agomelatine	1.09	1.53	2.11	0.53	2.91	16.25	0.35	0.52	0.77	0.18	0.75	5.54
Amitriptyline	1.17	1.62	2.23	0.85	2.41	5.35	0.49	0.71	1.02	0.29	0.64	1.28
Bupropion	1.12	1.73	2.65	0.59	2.93	15.27	0.41	0.65	1.05	0.18	0.74	4.49
Citalopram	0.98	1.34	1.82	0.60	2.97	16.68	0.40	0.58	0.83	0.10	0.65	2.61
Clomipramine	0.92	1.30	1.80	1.10	3.08	10.46	0.61	0.91	1.35	0.17	0.72	3.35
Duloxetine	1.00	1.42	2.00	0.55	2.96	18.63	0.56	0.84	1.25	0.11	0.70	3.72
Escitalopram	1.23	1.70	2.33	0.85	3.18	22.33	0.35	0.53	0.78	0.34	0.82	2.70
Fluoxetine	0.97	1.30	1.75	-	-	-	0.42	0.60	0.85	-	-	-
Fluvoxamine	0.86	1.23	1.74	0.62	2.90	13.72	0.51	0.76	1.14	0.13	0.66	2.42
Milnacipran	0.97	1.39	1.97	0.27	2.58	9.48	0.44	0.66	0.99	0.14	0.71	3.59
Mirtazapine	1.09	1.52	2.10	1.66	3.77	19.26	0.45	0.66	1.00	0.16	0.60	1.33
Nefazodone	0.74	1.21	1.91	0.62	2.67	8.58	0.41	0.73	1.29	0.42	1.00	4.46
Paroxetine	1.09	1.47	1.96	0.95	3.00	11.36	0.45	0.65	0.92	0.13	0.59	1.53
Sertraline	0.99	1.37	1.86	1.38	3.86	45.74	0.43	0.62	0.91	0.07	0.57	1.63
Trazodone	0.71	1.04	1.52	0.91	2.75	7.66	0.48	0.76	1.22	0.41	0.93	3.38
Venlafaxine	1.07	1.45	1.96	0.86	2.73	7.85	0.50	0.72	1.04	0.14	0.57	1.37
Vortioxetine	1.15	2.01	3.50	0.56	2.96	18.32	0.23	0.42	0.79	0.11	0.70	3.72

8.5.2.3 *Adjusted for year of studies*

	Response						Dropouts for any reason					
	OR drug vs PLA			beta of comparison			OR drug vs PLA			beta of comparison		
	2.5%	median	97.5%	2.5%	median	97.5%	2.5%	median	97.5%	2.5%	median	97.5%
Agomelatine	0.82	1.35	2.18	0.63	0.98	1.42	0.32	0.54	0.86	0.65	0.98	1.41
Amitriptyline	1.00	1.44	2.06	0.75	1.00	1.29	0.54	0.79	1.18	0.67	0.91	1.20
Bupropion	0.95	1.51	2.36	0.73	1.05	1.53	0.44	0.70	1.18	0.78	1.05	1.52

Citalopram	0.79	1.14	1.61	0.64	0.87	1.16	0.43	0.62	0.92	0.80	1.08	1.58
Clomipramine	0.79	1.18	1.74	0.65	0.97	1.33	0.61	0.95	1.50	0.68	0.99	1.43
Duloxetine	0.56	1.08	1.77	0.42	0.82	1.21	0.62	1.03	2.34	0.82	1.17	2.60
Escitalopram	1.01	1.58	2.40	0.67	1.02	1.49	0.39	0.60	1.07	0.78	1.09	1.97
Fluoxetine	0.83	1.15	1.58	-	-	-	0.46	0.64	0.92	-	-	-
Fluvoxamine	0.79	1.15	1.65	0.58	0.88	1.22	0.53	0.79	1.21	0.79	1.08	1.74
Milnacipran	0.87	1.28	1.85	0.71	0.95	1.23	0.46	0.69	1.06	0.75	0.99	1.33
Mirtazapine	0.99	1.43	2.05	0.63	0.95	1.31	0.47	0.70	1.07	0.78	1.08	1.71
Nefazodone	0.75	1.20	1.93	0.66	1.05	1.84	0.45	0.76	1.35	0.61	0.99	1.59
Paroxetine	1.00	1.38	1.88	0.79	0.98	1.21	0.48	0.66	0.98	0.78	0.98	1.23
Sertraline	0.93	1.31	1.82	0.82	1.10	1.49	0.44	0.63	0.93	0.66	0.94	1.27
Trazodone	0.67	1.01	1.50	0.69	0.99	1.35	0.51	0.79	1.27	0.74	1.02	1.47
Venlafaxine	1.10	1.55	2.14	1.01	1.38	1.91	0.49	0.70	1.03	0.59	0.90	1.21
Vortioxetine	0.45	1.34	3.27	0.57	1.00	1.63	0.22	0.49	1.60	0.65	1.01	1.66

9 GRADE for the primary outcomes

9.1 Response

9.1.1 Summary of study limitations of the included studies

The colours in the circles indicate the percentage of low RoB studies [green], moderate RoB studies [yellow] and high RoB studies [red] involving each antidepressant.

The colours of the line then indicate the summative RoB assessment of each comaparison based on the above information – low RoB comparison [green] and moderate RoB comparison [yellow]. There was no comparison which was judged to be at high RoB.

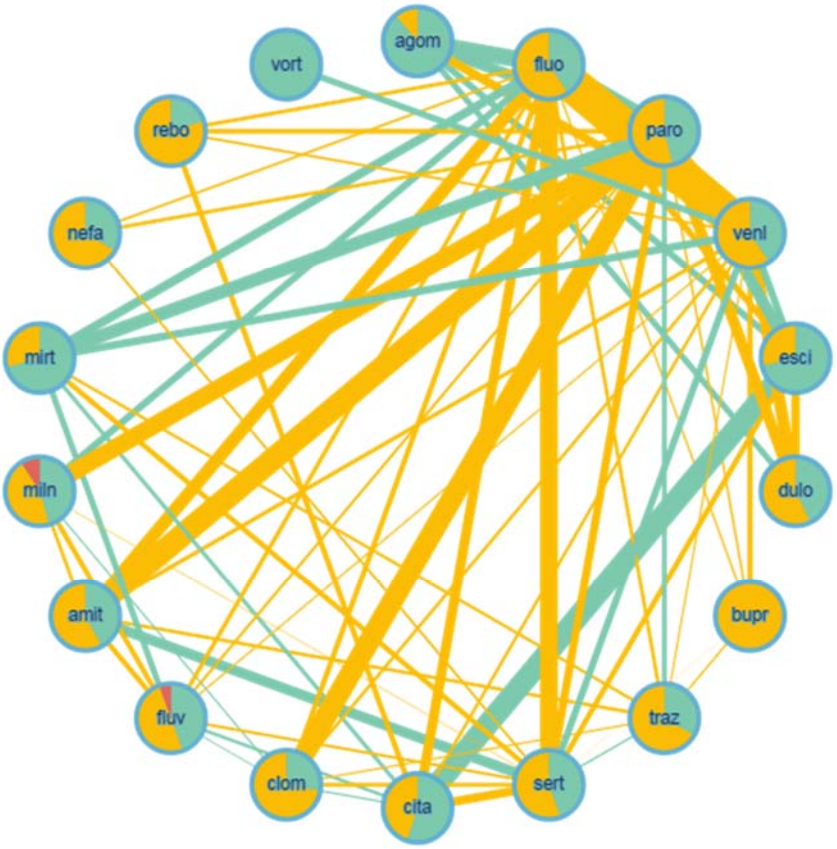

9.1.2 Contribution of low or moderate RoB comparisons to each network estimate

Based on the above assessment of RoB for each comparison and the contribution matrix detailing contribution of each direct comparison to all network estimates, the following bar graphs show the percentage of low or moderate RoB contributions for each network estimate.

The judgements about study limitations in each direct comparison is shown at the beginning of the graph. Each bar corresponds to a NMA relative treatment effect and shows how much information comes from comparisons at low risk of bias [green] or moderate risk of bias [yellow].

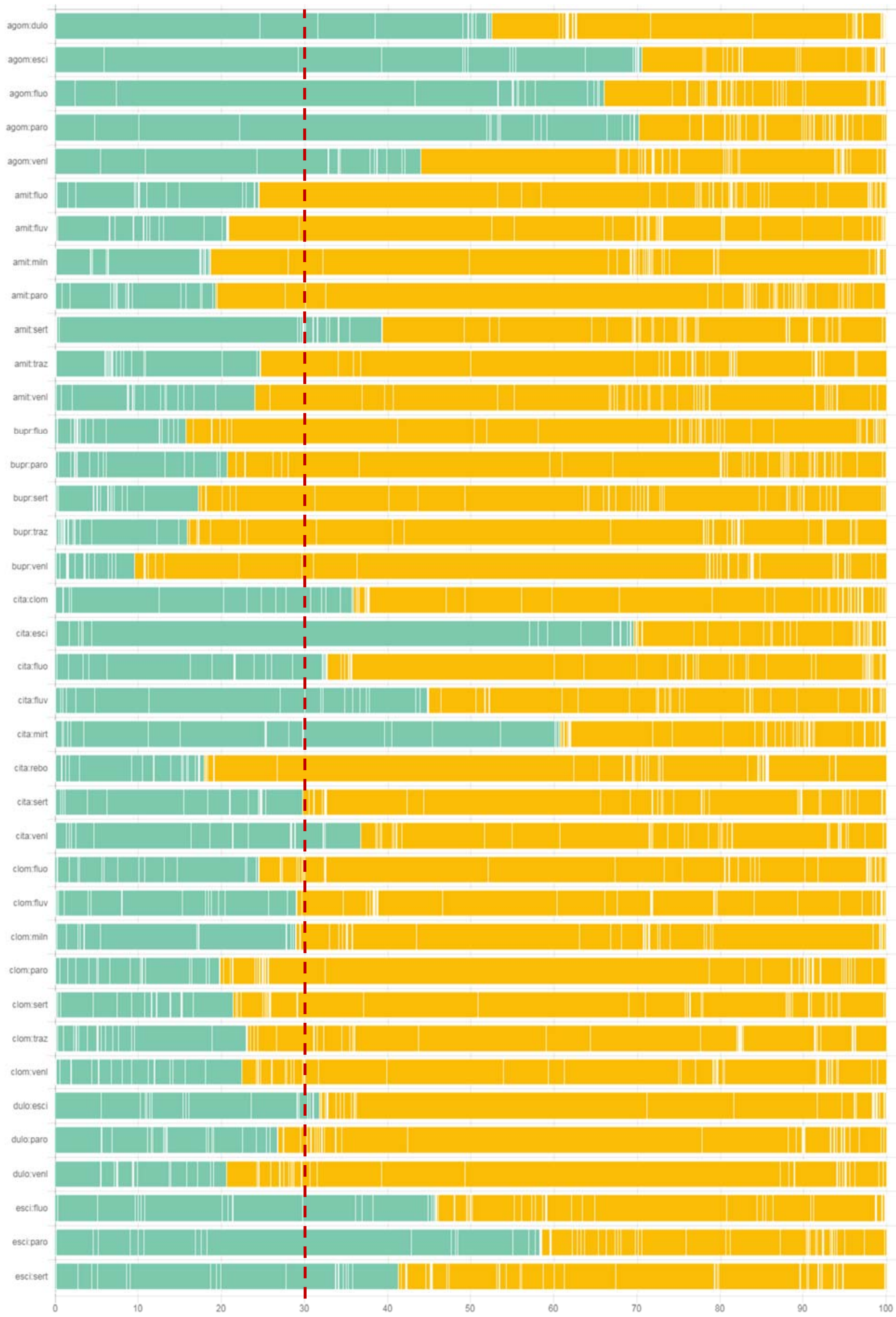
agom:dulo
agom:esci
agom:fluo
agom:paro
agom:venl
amit:fluo
amit:fluv
amit:miln
amit:paro
amit:sert
amit:traz
amit:venl
bupr:fluo
bupr:paro
bupr:sert
bupr:traz
bupr:venl
cita:clom
cita:esci
cita:fluo
cita:fluv
cita:mirt
cita:rebo
cita:sert
cita:venl
clom:fluo
clom:fluv
clom:miln
clom:paro
clom:sert
clom:traz
clom:venl
dulo:esci
dulo:paro
dulo:venl
esci:fluo
esci:paro
esci:sert
0
10
20
30
40
50
60
70
80
90
100

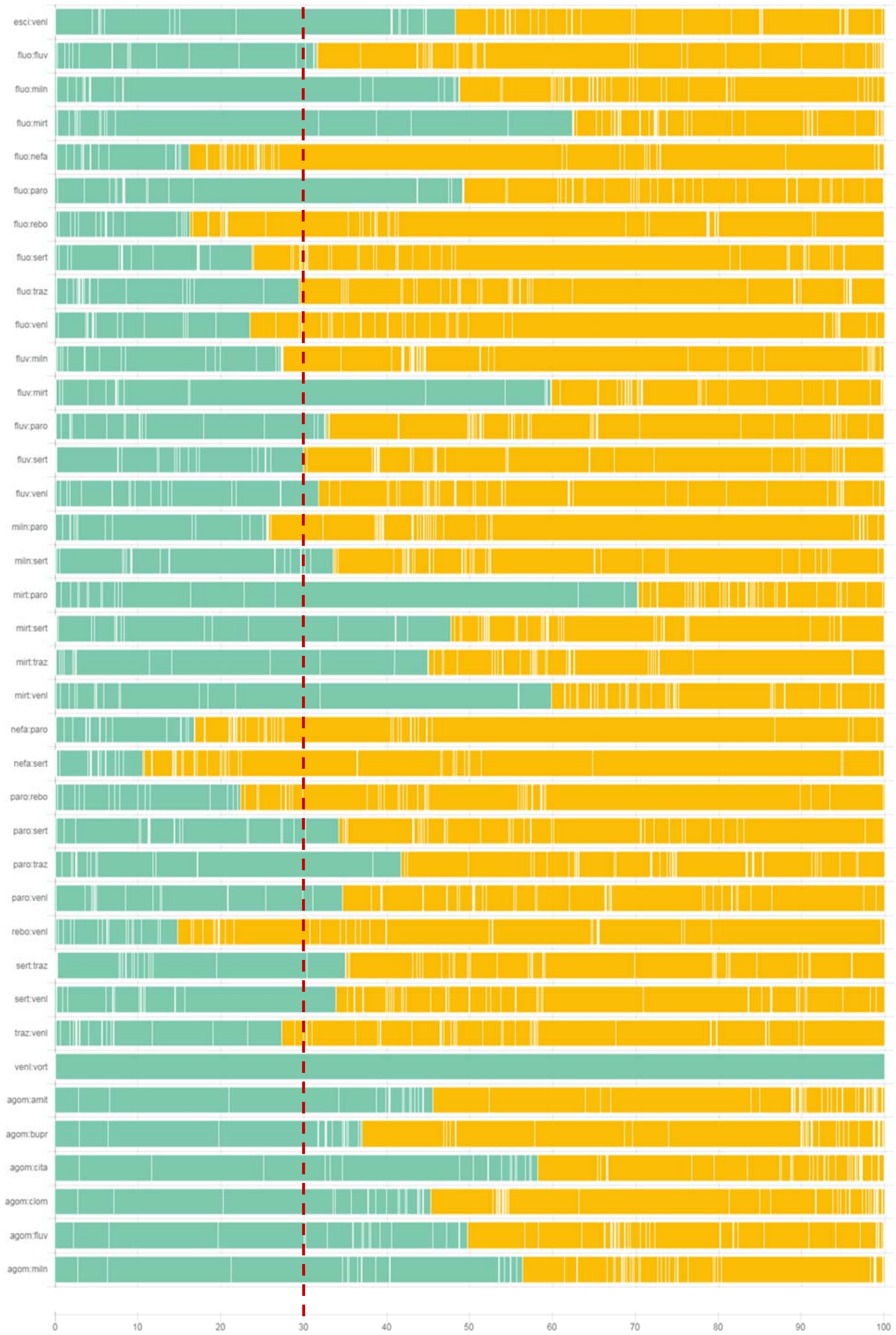
esci:venl
fluo:fluv
fluo:miln
fluo:mirt
fluo:nefa
fluo:paro
fluo:rebo
fluo:sert
fluo:traz
fluo:venl
fluv:miln
fluv:mirt
fluv:paro
fluv:sert
fluv:venl
miln:paro
miln:sert
mirt:paro
mirt:sert
mirt:traz
mirt:venl
nefa:paro
nefa:sert
paro:rebo
paro:sert
paro:traz
paro:venl
rebo:venl
sert:traz
sert:venl
traz:venl
venl:vort
agom:amit
agom:bupr
agom:cita
agom:clom
agom:fluv
agom:miln
0
10
20
30
40
50
60
70
80
90
100

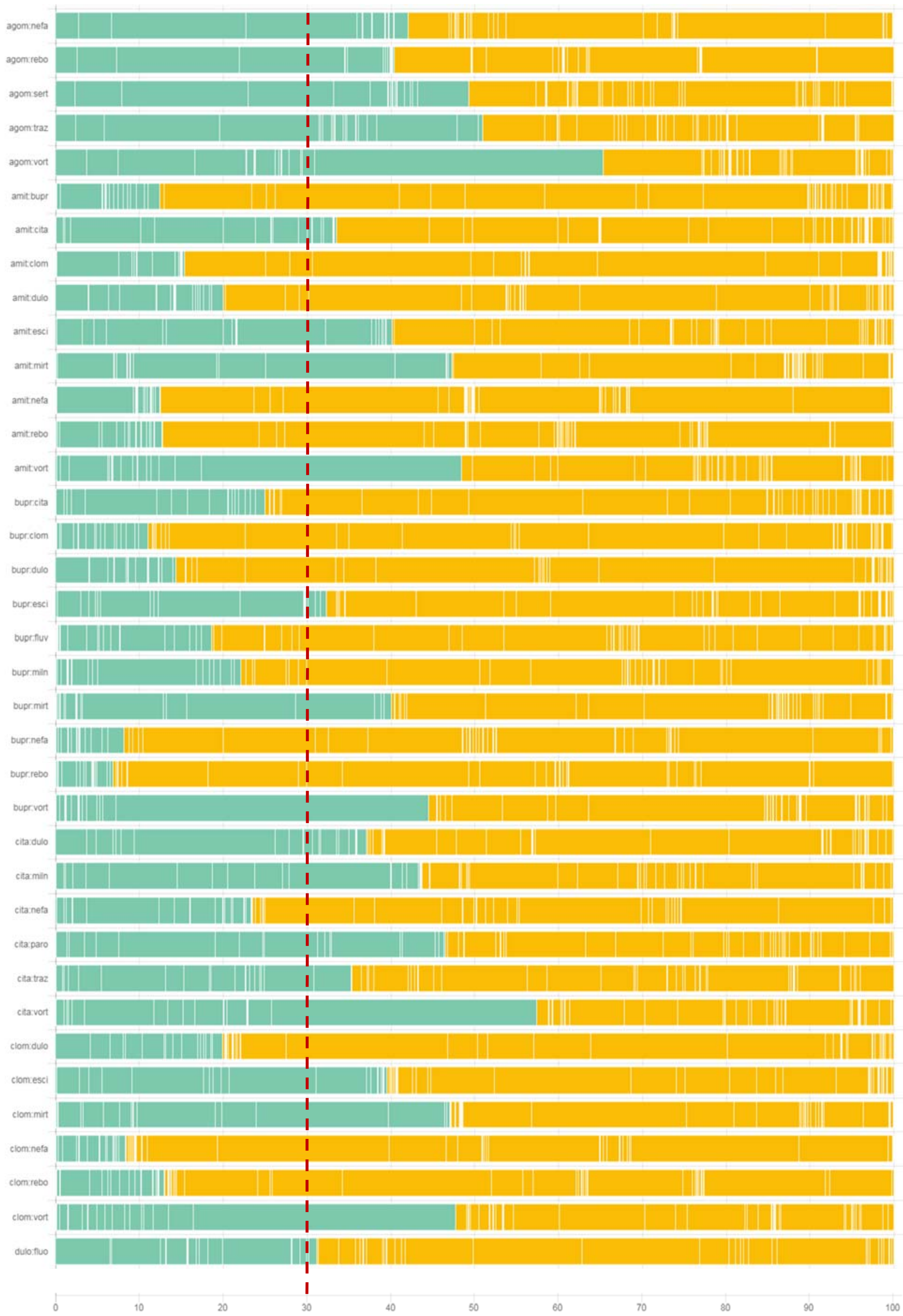
agom:nefa
agom:rebo
agom:sert
agom:traz
agom:vort
amit:bupr
amit:cita
amit:clom
amit:dulo
amit:esci
amit:mirt
amit:nefa
amit:rebo
amit:vort
bupr:cita
bupr:clom
bupr:dulo
bupr:esci
bupr:fluv
bupr:miln
bupr:mirt
bupr:nefa
bupr:rebo
bupr:vort
cita:dulo
cita:miln
cita:nefa
cita:paro
cita:traz
cita:vort
clom:dulo
clom:esci
clom:mirt
clom:nefa
clom:rebo
clom:vort
dulo:fluo
0
10
20
30
40
50
60
70
80
90
100

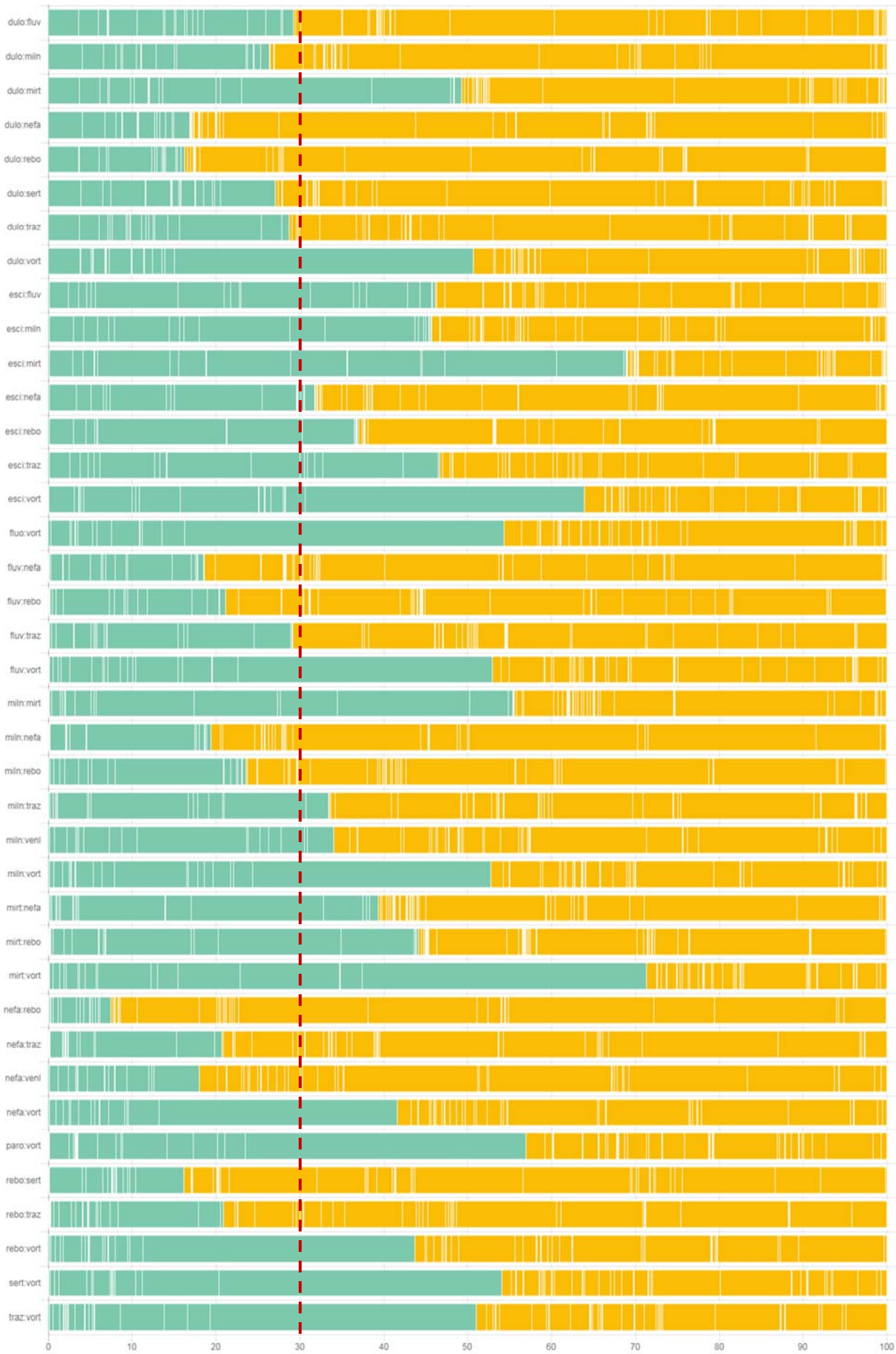
dulo:fluv
dulo:miln
dulo:mirt
dulo:nefa
dulo:rebo
dulo:sert
dulo:traz
dulo:vort
esci:fluv
esci:miln
esci:mirt
esci:nefa
esci:rebo
esci:traz
esci:vort
fluo:vort
fluv:nefa
fluv:rebo
fluv:traz
fluv:vort
miln:mirt
miln:nefa
miln:rebo
miln:traz
miln:venl
miln:vort
mirt:nefa
mirt:rebo
mirt:vort
nefa:rebo
nefa:traz
nefa:venl
nefa:vort
paro:vort
rebo:sert
rebo:traz
rebo:vort
sert:vort
traz:vort
0
10
20
30
40
50
60
70
80
90
100

9.1.3 Table of reasons for downgrading

Based on all the above information, we GRADEd each network estimate according to the following criteria.

(1) **Study limitations**: We downgraded by one level when the contributions from low RoB comparisons were less than 30% and contributions from moderate RoB comparisons were 70% or greater.

(2) **Imprecision**: We considered a clinically meaningful threshold for OR to be 0.80 or 1.25 and downgraded the estimate if the OR point estimate is 1 or more and the lower limit of its CrI is below 0.80; or if the OR point estimate is less than 1 and the upper limit of its CrI is above 1.25.

(3) **Inconsistency**: We rated two concepts, heterogeneity and incoherence (inconsistency), in this domain. For heterogeneity, we looked at the common tau and found that it is low compared to the expected value as reported in the literature (Turner RM et al (2012) Int J Epidemiol, 41, 818-827) , so we did not downgrade any network estimate for heterogeneity. For inconsistency, we looked at the results of side splitting (8.3.1.2) and we downgraded the comparisons with important inconsistency ($p<0.10$), where we have not downgraded for imprecision (we did not downgrade the same network estimate for both imprecision and inconsistency).

(4) **Indirectness**: We have assured transitivity in our network by limiting the included studies to non-psychotic non-refractory unipolar major depression, because bipolarity, psychotic features, subthreshold depression and treatment refractoriness are established treatment modifiers for antidepressants. We further ran various meta-regressions depression severity, and except for placebo-controlled trials, assured that they did not violate transitivity of the network. However, we downgraded singly-connected nodes for indirectness because evaluation of transitivity for such nodes is unclear.

(5) **Publication bias**: The comparison-adjusted funnel plot (8.4.1) did not suggest presence of overall publication bias in comparison with the old, standard drug fluoxetine. Further, there was no network estimate for which the meta-regression for small-study effects suggested the statistically significant influence of small-study effects (8.5.2.2). We managed to retrieve supplementary and unpublished information included in the available systematic reviews and network meta-analyses, and we are confident that we have all available information that is possible to capture from clinical trial registries, regulatory agencies' repositories and drug companies' websites (particularly for the newest and most recently marketed antidepressants). However, we cannot completely rule out the possibility that some studies are still missing. Considering that the field of antidepressant trials in the past has been prone to publication bias, the review team decided by default to downgrade all the included studies for potential publication bias by one level.

	Study limitations	Imprecision	Inconsistency	Indirectness	Publication bias	GRADE
**Agom vs Amit**	No downgrade	No downgrade	No downgrade	No downgrade	Downgrade	MODERATE
**Agom vs Bupr**	No downgrade	Downgrade because point estimate <1.0 but upper limit >1.25	No downgrade	No downgrade	Downgrade	LOW
**Agom vs Cita**	No downgrade	No downgrade	No downgrade	No downgrade	Downgrade	MODERATE
**Agom vs Clom**	No downgrade	No downgrade	No downgrade	No downgrade	Downgrade	MODERATE
**Agom vs Dulo**	No downgrade	No downgrade	No downgrade	No downgrade	Downgrade	MODERATE
**Agom vs Esci**	No downgrade	No downgrade	No downgrade	No downgrade	Downgrade	MODERATE
**Agom vs Fluo**	No downgrade	No downgrade	No downgrade	No downgrade	Downgrade	MODERATE
**Agom vs Fluv**	No downgrade	No downgrade	No downgrade	No downgrade	Downgrade	MODERATE
**Agom vs Miln**	No downgrade	No downgrade	No downgrade	No downgrade	Downgrade	MODERATE
**Agom vs Mirt**	No downgrade	No downgrade	No downgrade	No downgrade	Downgrade	MODERATE
**Agom vs Nefa**	No downgrade	Downgrade because point estimate >1.0 but lower limit <0.80	No downgrade	No downgrade	Downgrade	LOW
**Agom vs Paro**	No downgrade	No downgrade	No downgrade	No downgrade	Downgrade	MODERATE
**Agom vs Rebo**	No downgrade	No downgrade	No downgrade	No downgrade	Downgrade	MODERATE
**Agom vs Sert**	No downgrade	No downgrade	No downgrade	No downgrade	Downgrade	MODERATE
**Agom vs Traz**	No downgrade	No downgrade	No downgrade	No downgrade	Downgrade	MODERATE
**Agom vs Venl**	No downgrade	No downgrade	No downgrade	No downgrade	Downgrade	MODERATE
**Agom vs Vort**	No downgrade	Downgrade because point estimate <1.0 but upper limit >1.25	No downgrade	Downgrade because of concerns about transitivity. Vort is a singly connected node and hence evaluation of transitivity is unclear.	Downgrade	VERY LOW
**Amit vs Bupr**	Downgrade because >70% contribution from moderate RoB comparisons	Downgrade because point estimate <1.0 but upper limit >1.25	No downgrade	No downgrade	Downgrade	VERY LOW
**Amit vs Cita**	No downgrade	No downgrade	No downgrade	No downgrade	Downgrade	MODERATE
**Amit vs Clom**	Downgrade because >70% contribution from moderate RoB comparisons	No downgrade	No downgrade	No downgrade	Downgrade	LOW
**Amit vs Dulo**	Downgrade because >70% contribution from moderate RoB comparisons	No downgrade	No downgrade	No downgrade	Downgrade	LOW

**Amit vs Esci**	No downgrade	No downgrade	No downgrade	No downgrade	Downgrade	MODERATE
**Amit vs Fluo**	Downgrade because >70% contribution from moderate RoB comparisons	No downgrade	No downgrade	No downgrade	Downgrade	LOW
**Amit vs Fluv**	Downgrade because >70% contribution from moderate RoB comparisons	No downgrade	No downgrade	No downgrade	Downgrade	LOW
**Amit vs Miln**	Downgrade because >70% contribution from moderate RoB comparisons	No downgrade	No downgrade	No downgrade	Downgrade	LOW
**Amit vs Mirt**	No downgrade	No downgrade	No downgrade	No downgrade	Downgrade	MODERATE
**Amit vs Nefa**	Downgrade because >70% contribution from moderate RoB comparisons	No downgrade	No downgrade	No downgrade	Downgrade	LOW
**Amit vs Paro**	Downgrade because >70% contribution from moderate RoB comparisons	No downgrade	No downgrade	No downgrade	Downgrade	LOW
**Amit vs Rebo**	Downgrade because >70% contribution from moderate RoB comparisons	No downgrade	No downgrade	No downgrade	Downgrade	LOW
**Amit vs Sert**	No downgrade	No downgrade	No downgrade	No downgrade	Downgrade	MODERATE
**Amit vs Traz**	Downgrade because >70% contribution from moderate RoB comparisons	No downgrade	No downgrade	No downgrade	Downgrade	LOW
**Amit vs Venl**	Downgrade because >70% contribution from moderate RoB comparisons	No downgrade	No downgrade	No downgrade	Downgrade	LOW
**Amit vs Vort**	No downgrade	Downgrade because point estimate <1.0 but upper limit >1.25	No downgrade	Downgrade because of concerns about transitivity. Vort is a singly connected node and hence evaluation of transitivity is unclear.	Downgrade	VERY LOW

**Bupr vs Cita**	Downgrade because >70% contribution from moderate RoB comparisons	No downgrade	No downgrade	No downgrade	Downgrade	LOW
**Bupr vs Clom**	Downgrade because >70% contribution from moderate RoB comparisons	No downgrade	No downgrade	No downgrade	Downgrade	LOW
**Bupr vs Dulo**	Downgrade because >70% contribution from moderate RoB comparisons	No downgrade	No downgrade	No downgrade	Downgrade	LOW
**Bupr vs Esci**	No downgrade	Downgrade because point estimate >1.0 but lower limit <0.80	No downgrade	No downgrade	Downgrade	LOW
**Bupr vs Fluo**	Downgrade because >70% contribution from moderate RoB comparisons	No downgrade	No downgrade	No downgrade	Downgrade	LOW
**Bupr vs Fluv**	Downgrade because >70% contribution from moderate RoB comparisons	No downgrade	No downgrade	No downgrade	Downgrade	LOW
**Bupr vs Miln**	Downgrade because >70% contribution from moderate RoB comparisons	No downgrade	No downgrade	No downgrade	Downgrade	LOW
**Bupr vs Mirt**	No downgrade	Downgrade because point estimate >1.0 but lower limit <0.80	No downgrade	No downgrade	Downgrade	LOW
**Bupr vs Nefa**	Downgrade because >70% contribution from moderate RoB comparisons	Downgrade because point estimate >1.0 but lower limit <0.80	No downgrade	No downgrade	Downgrade	VERY LOW
**Bupr vs Paro**	Downgrade because >70% contribution from moderate RoB comparisons	No downgrade	No downgrade	No downgrade	Downgrade	LOW
**Bupr vs Rebo**	Downgrade because >70% contribution from moderate RoB comparisons	No downgrade	No downgrade	No downgrade	Downgrade	LOW

**Bupr vs Sert**	Downgrade because >70% contribution from moderate RoB comparisons	No downgrade	No downgrade	No downgrade	Downgrade	LOW
**Bupr vs Traz**	Downgrade because >70% contribution from moderate RoB comparisons	No downgrade	No downgrade	No downgrade	Downgrade	LOW
**Bupr vs Venl**	Downgrade because >70% contribution from moderate RoB comparisons	No downgrade	No downgrade	No downgrade	Downgrade	LOW
**Bupr vs Vort**	No downgrade	Downgrade because point estimate <1.0 but upper limit >1.25	No downgrade	Downgrade because of concerns about transitivity. Vort is a singly connected node and hence evaluation of transitivity is unclear.	Downgrade	VERY LOW
**Cita vs Clom**	No downgrade	No downgrade	No downgrade	No downgrade	Downgrade	MODERATE
**Cita vs Dulo**	No downgrade	No downgrade	No downgrade	No downgrade	Downgrade	MODERATE
**Cita vs Esci**	No downgrade	No downgrade	No downgrade	No downgrade	Downgrade	MODERATE
**Cita vs Fluo**	No downgrade	No downgrade	No downgrade	No downgrade	Downgrade	MODERATE
**Cita vs Fluv**	No downgrade	No downgrade	No downgrade	No downgrade	Downgrade	MODERATE
**Cita vs Miln**	No downgrade	Downgrade because point estimate <1.0 but upper limit >1.25	No downgrade	No downgrade	Downgrade	LOW
**Cita vs Mirt**	No downgrade	No downgrade	No downgrade	No downgrade	Downgrade	MODERATE
**Cita vs Nefa**	Downgrade because >70% contribution from moderate RoB comparisons	Downgrade because point estimate >1.0 but lower limit <0.80	No downgrade	No downgrade	Downgrade	VERY LOW
**Cita vs Paro**	No downgrade	No downgrade	No downgrade	No downgrade	Downgrade	MODERATE
**Cita vs Rebo**	Downgrade because >70% contribution from moderate RoB comparisons	No downgrade	No downgrade	No downgrade	Downgrade	LOW
**Cita vs Sert**	Downgrade because >70% contribution from moderate RoB comparisons	No downgrade	No downgrade	No downgrade	Downgrade	LOW
**Cita vs Traz**	No downgrade	No downgrade	No downgrade	No downgrade	Downgrade	MODERATE

**Cita vs Venl**	No downgrade	No downgrade	Downgrade for side-splitting p=0.042	No downgrade	Downgrade	LOW
**Cita vs Vort**	No downgrade	No downgrade	No downgrade	Downgrade because of concerns about transitivity. Vort is a singly connected node and hence evaluation of transitivity is unclear.	Downgrade	LOW
**Clom vs Dulo**	Downgrade because >70% contribution from moderate RoB comparisons	No downgrade	No downgrade	No downgrade	Downgrade	LOW
**Clom vs Esci**	No downgrade	No downgrade	No downgrade	No downgrade	Downgrade	MODERATE
**Clom vs Fluo**	Downgrade because >70% contribution from moderate RoB comparisons	No downgrade	Downgrade for side-splitting p=0.020	No downgrade	Downgrade	VERY LOW
**Clom vs Fluv**	Downgrade because >70% contribution from moderate RoB comparisons	Downgrade because point estimate >1.0 but lower limit <0.80	No downgrade	No downgrade	Downgrade	VERY LOW
**Clom vs Miln**	Downgrade because >70% contribution from moderate RoB comparisons	No downgrade	No downgrade	No downgrade	Downgrade	LOW
**Clom vs Mirt**	No downgrade	No downgrade	No downgrade	No downgrade	Downgrade	MODERATE
**Clom vs Nefa**	Downgrade because >70% contribution from moderate RoB comparisons	Downgrade because point estimate <1.0 but upper limit >1.25	No downgrade	No downgrade	Downgrade	VERY LOW
**Clom vs Paro**	Downgrade because >70% contribution from moderate RoB comparisons	No downgrade	No downgrade	No downgrade	Downgrade	LOW
**Clom vs Rebo**	Downgrade because >70% contribution from moderate RoB comparisons	No downgrade	No downgrade	No downgrade	Downgrade	LOW
**Clom vs Sert**	Downgrade because >70% contribution from moderate RoB comparisons	No downgrade	No downgrade	No downgrade	Downgrade	LOW

**Clom vs Traz**	Downgrade because >70% contribution from moderate RoB comparisons	No downgrade	No downgrade	No downgrade	Downgrade	LOW
**Clom vs Venl**	Downgrade because >70% contribution from moderate RoB comparisons	No downgrade	No downgrade	No downgrade	Downgrade	LOW
**Clom vs Vort**	No downgrade	No downgrade	No downgrade	Downgrade because of concerns about transitivity. Vort is a singly connected node and hence evaluation of transitivity is unclear.	Downgrade	LOW
**Dulo vs Esci**	No downgrade	No downgrade	No downgrade	No downgrade	Downgrade	MODERATE
**Dulo vs Fluo**	No downgrade	No downgrade	No downgrade	No downgrade	Downgrade	MODERATE
**Dulo vs Fluv**	Downgrade because >70% contribution from moderate RoB comparisons	No downgrade	No downgrade	No downgrade	Downgrade	LOW
**Dulo vs Miln**	Downgrade because >70% contribution from moderate RoB comparisons	Downgrade because point estimate >1.0 but lower limit <0.80	No downgrade	No downgrade	Downgrade	VERY LOW
**Dulo vs Mirt**	No downgrade	No downgrade	No downgrade	No downgrade	Downgrade	MODERATE
**Dulo vs Nefa**	Downgrade because >70% contribution from moderate RoB comparisons	Downgrade because point estimate >1.0 but lower limit <0.80	No downgrade	No downgrade	Downgrade	VERY LOW
**Dulo vs Paro**	Downgrade because >70% contribution from moderate RoB comparisons	No downgrade	No downgrade	No downgrade	Downgrade	LOW
**Dulo vs Rebo**	Downgrade because >70% contribution from moderate RoB comparisons	No downgrade	No downgrade	No downgrade	Downgrade	LOW
**Dulo vs Sert**	Downgrade because >70% contribution from moderate RoB comparisons	Downgrade because point estimate >1.0 but lower limit <0.80	No downgrade	No downgrade	Downgrade	VERY LOW

**Dulo vs Traz**	Downgrade because >70% contribution from moderate RoB comparisons	No downgrade	No downgrade	No downgrade	Downgrade	LOW
**Dulo vs Venl**	Downgrade because >70% contribution from moderate RoB comparisons	No downgrade	No downgrade	No downgrade	Downgrade	LOW
**Dulo vs Vort**	No downgrade	No downgrade	No downgrade	Downgrade because of concerns about transitivity. Vort is a singly connected node and hence evaluation of transitivity is unclear.	Downgrade	LOW
**Esci vs Fluo**	No downgrade	No downgrade	No downgrade	No downgrade	Downgrade	MODERATE
**Esci vs Fluv**	No downgrade	No downgrade	No downgrade	No downgrade	Downgrade	MODERATE
**Esci vs Miln**	No downgrade	No downgrade	No downgrade	No downgrade	Downgrade	MODERATE
**Esci vs Mirt**	No downgrade	No downgrade	No downgrade	No downgrade	Downgrade	MODERATE
**Esci vs Nefa**	No downgrade	No downgrade	No downgrade	No downgrade	Downgrade	MODERATE
**Esci vs Paro**	No downgrade	No downgrade	No downgrade	No downgrade	Downgrade	MODERATE
**Esci vs Rebo**	No downgrade	No downgrade	No downgrade	No downgrade	Downgrade	MODERATE
**Esci vs Sert**	No downgrade	No downgrade	No downgrade	No downgrade	Downgrade	MODERATE
**Esci vs Traz**	No downgrade	No downgrade	No downgrade	No downgrade	Downgrade	MODERATE
**Esci vs Venl**	No downgrade	No downgrade	No downgrade	No downgrade	Downgrade	MODERATE
**Esci vs Vort**	No downgrade	Downgrade because point estimate <1.0 but upper limit >1.25	No downgrade	Downgrade because of concerns about transitivity. Vort is a singly connected node and hence evaluation of transitivity is unclear.	Downgrade	VERY LOW
**Fluo vs Fluv**	No downgrade	No downgrade	No downgrade	No downgrade	Downgrade	MODERATE
**Fluo vs Miln**	No downgrade	No downgrade	No downgrade	No downgrade	Downgrade	MODERATE
**Fluo vs Mirt**	No downgrade	No downgrade	No downgrade	No downgrade	Downgrade	MODERATE
**Fluo vs Nefa**	Downgrade because >70% contribution from moderate RoB comparisons	Downgrade because point estimate <1.0 but upper limit >1.25	No downgrade	No downgrade	Downgrade	VERY LOW
**Fluo vs Paro**	No downgrade	No downgrade	No downgrade	No downgrade	Downgrade	MODERATE
**Fluo vs Rebo**	Downgrade because >70% contribution from moderate RoB comparisons	No downgrade	No downgrade	No downgrade	Downgrade	LOW

**Fluo vs Sert**	Downgrade because >70% contribution from moderate RoB comparisons	No downgrade	Downgrade because side-splitting p=0.022	No downgrade	Downgrade	VERY LOW
**Fluo vs Traz**	Downgrade because >70% contribution from moderate RoB comparisons	No downgrade	No downgrade	No downgrade	Downgrade	LOW
**Fluo vs Venl**	Downgrade because >70% contribution from moderate RoB comparisons	No downgrade	No downgrade	No downgrade	Downgrade	LOW
**Fluo vs Vort**	No downgrade	No downgrade	No downgrade	Downgrade because of concerns about transitivity. Vort is a singly connected node and hence evaluation of transitivity is unclear.	Downgrade	LOW
**Fluv vs Miln**	Downgrade because >70% contribution from moderate RoB comparisons	No downgrade	No downgrade	No downgrade	Downgrade	LOW
**Fluv vs Mirt**	No downgrade	No downgrade	No downgrade	No downgrade	Downgrade	MODERATE
**Fluv vs Nefa**	Downgrade because >70% contribution from moderate RoB comparisons	Downgrade because point estimate <1.0 but upper limit >1.25	No downgrade	No downgrade	Downgrade	VERY LOW
**Fluv vs Paro**	No downgrade	No downgrade	No downgrade	No downgrade	Downgrade	MODERATE
**Fluv vs Rebo**	Downgrade because >70% contribution from moderate RoB comparisons	No downgrade	No downgrade	No downgrade	Downgrade	LOW
**Fluv vs Sert**	No downgrade	No downgrade	Downgrade because side-splitting p=0.069	No downgrade	Downgrade	LOW
**Fluv vs Traz**	Downgrade because >70% contribution from moderate RoB comparisons	No downgrade	No downgrade	No downgrade	Downgrade	LOW
**Fluv vs Venl**	No downgrade	No downgrade	No downgrade	No downgrade	Downgrade	MODERATE

**Fluv vs Vort**	No downgrade	No downgrade	No downgrade	Downgrade because of concerns about transitivity. Vort is a singly connected node and hence evaluation of transitivity is unclear.	Downgrade	LOW
**Miln vs Mirt**	No downgrade	No downgrade	No downgrade	No downgrade	Downgrade	MODERATE
**Miln vs Nefa**	Downgrade because >70% contribution from moderate RoB comparisons	Downgrade because point estimate >1.0 but lower limit <0.80	No downgrade	No downgrade	Downgrade	VERY LOW
**Miln vs Paro**	Downgrade because >70% contribution from moderate RoB comparisons	No downgrade	No downgrade	No downgrade	Downgrade	LOW
**Miln vs Rebo**	Downgrade because >70% contribution from moderate RoB comparisons	No downgrade	No downgrade	No downgrade	Downgrade	LOW
**Miln vs Sert**	No downgrade	Downgrade because point estimate <1.0 but upper limit >1.25	No downgrade	No downgrade	Downgrade	LOW
**Miln vs Traz**	No downgrade	No downgrade	No downgrade	No downgrade	Downgrade	MODERATE
**Miln vs Venl**	No downgrade	No downgrade	No downgrade	No downgrade	Downgrade	MODERATE
**Miln vs Vort**	No downgrade	No downgrade	No downgrade	Downgrade because of concerns about transitivity. Vort is a singly connected node and hence evaluation of transitivity is unclear.	Downgrade	LOW
**Mirt vs Nefa**	No downgrade	No downgrade	No downgrade	No downgrade	Downgrade	MODERATE
**Mirt vs Paro**	No downgrade	No downgrade	No downgrade	No downgrade	Downgrade	MODERATE
**Mirt vs Rebo**	No downgrade	No downgrade	No downgrade	No downgrade	Downgrade	MODERATE
**Mirt vs Sert**	No downgrade	No downgrade	No downgrade	No downgrade	Downgrade	MODERATE
**Mirt vs Traz**	No downgrade	No downgrade	No downgrade	No downgrade	Downgrade	MODERATE
**Mirt vs Venl**	No downgrade	No downgrade	No downgrade	No downgrade	Downgrade	MODERATE
**Mirt vs Vort**	No downgrade	Downgrade because point estimate <1.0 but upper limit >1.25	No downgrade	Downgrade because of concerns about transitivity. Vort is a singly connected node and hence evaluation of transitivity is unclear.	Downgrade	VERY LOW

**Nefa vs Paro**	Downgrade because >70% contribution from moderate RoB comparisons	Downgrade because point estimate <1.0 but upper limit >1.25	No downgrade	No downgrade	Downgrade	VERY LOW
**Nefa vs Rebo**	Downgrade because >70% contribution from moderate RoB comparisons	Downgrade because point estimate >1.0 but lower limit <0.80	No downgrade	No downgrade	Downgrade	VERY LOW
**Nefa vs Sert**	Downgrade because >70% contribution from moderate RoB comparisons	Downgrade because point estimate <1.0 but upper limit >1.25	No downgrade	No downgrade	Downgrade	VERY LOW
**Nefa vs Traz**	Downgrade because >70% contribution from moderate RoB comparisons	Downgrade because point estimate >1.0 but lower limit <0.80	No downgrade	No downgrade	Downgrade	VERY LOW
**Nefa vs Venl**	Downgrade because >70% contribution from moderate RoB comparisons	Downgrade because point estimate <1.0 but upper limit >1.25	No downgrade	No downgrade	Downgrade	VERY LOW
**Nefa vs Vort**	No downgrade	No downgrade	No downgrade	Downgrade because of concerns about transitivity. Vort is a singly connected node and hence evaluation of transitivity is unclear.	Downgrade	LOW
**Paro vs Rebo**	Downgrade because >70% contribution from moderate RoB comparisons	No downgrade	No downgrade	No downgrade	Downgrade	LOW
**Paro vs Sert**	No downgrade	No downgrade	No downgrade	No downgrade	Downgrade	MODERATE
**Paro vs Traz**	No downgrade	No downgrade	No downgrade	No downgrade	Downgrade	MODERATE
**Paro vs Venl**	No downgrade	No downgrade	Downgrade for side-splitting p=0.033	No downgrade	Downgrade	LOW
**Paro vs Vort**	No downgrade	No downgrade	No downgrade	Downgrade because of concerns about transitivity. Vort is a singly connected node and hence evaluation of transitivity is unclear.	Downgrade	LOW

**Rebo vs Sert**	Downgrade because >70% contribution from moderate RoB comparisons	No downgrade	No downgrade	No downgrade	Downgrade	LOW
**Rebo vs Traz**	Downgrade because >70% contribution from moderate RoB comparisons	Downgrade because point estimate <1.0 but upper limit >1.25	No downgrade	No downgrade	Downgrade	VERY LOW
**Rebo vs Venl**	Downgrade because >70% contribution from moderate RoB comparisons	No downgrade	No downgrade	No downgrade	Downgrade	LOW
**Rebo vs Vort**	No downgrade	No downgrade	No downgrade	Downgrade because of concerns about transitivity. Vort is a singly connected node and hence evaluation of transitivity is unclear.	Downgrade	LOW
**Sert vs Traz**	No downgrade	No downgrade	Downgrade for side-splitting p=0.038	No downgrade	Downgrade	LOW
**Sert vs Venl**	No downgrade	No downgrade	No downgrade	No downgrade	Downgrade	MODERATE
**Sert vs Vort**	No downgrade	No downgrade	No downgrade	Downgrade because of concerns about transitivity. Vort is a singly connected node and hence evaluation of transitivity is unclear.	Downgrade	LOW
**Traz vs Venl**	Downgrade because >70% contribution from moderate RoB comparisons	No downgrade	No downgrade		Downgrade	LOW
**Traz vs Vort**	No downgrade	No downgrade	No downgrade	Downgrade because of concerns about transitivity. Vort is a singly connected node and hence evaluation of transitivity is unclear.	Downgrade	LOW

**Venl vs Vort**	No downgrade	No downgrade	No downgrade	Downgrade because of concerns about transitivity. Vort is a singly connected node and hence evaluation of transitivity is unclear.	Downgrade	LOW

9.2 *Dropouts for any reason*

9.2.1 Summary of study limitations of the included studies

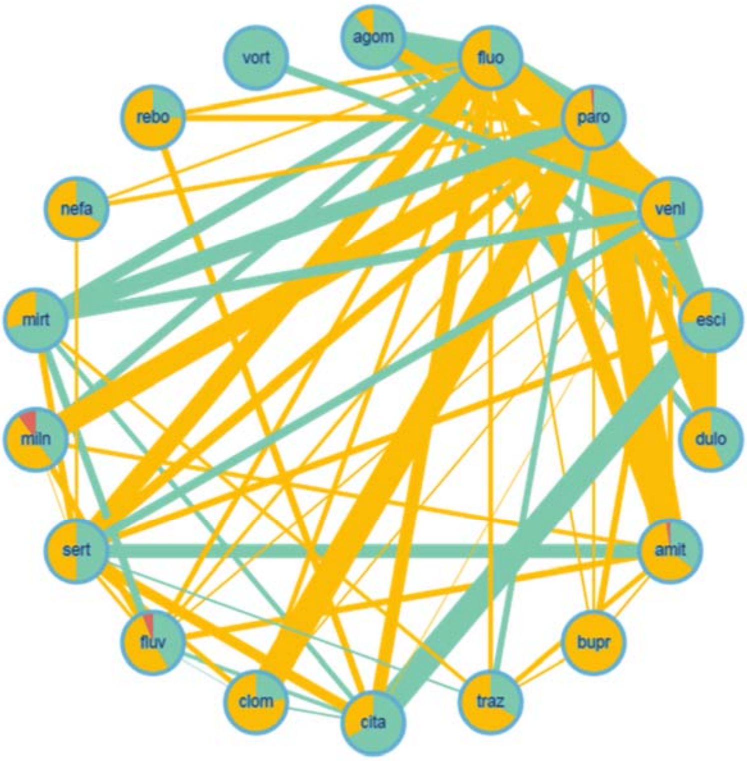

9.2.2 Contribution of low or moderate RoB comparisons to each network estimate

Based on the above assessment of RoB for each comparison and the contribution matrix detailing contribution of each direct comparison to all network estimates, the following bar graphs show the percentage of low or moderate RoB contributions for each network estimate.

The judgements about study limitations in each direct comparison is shown at the beginning of the graph. Each bar corresponds to a NMA relative treatment effect and shows how much information comes from comparisons at low risk of bias [green] or moderate risk of bias [yellow].

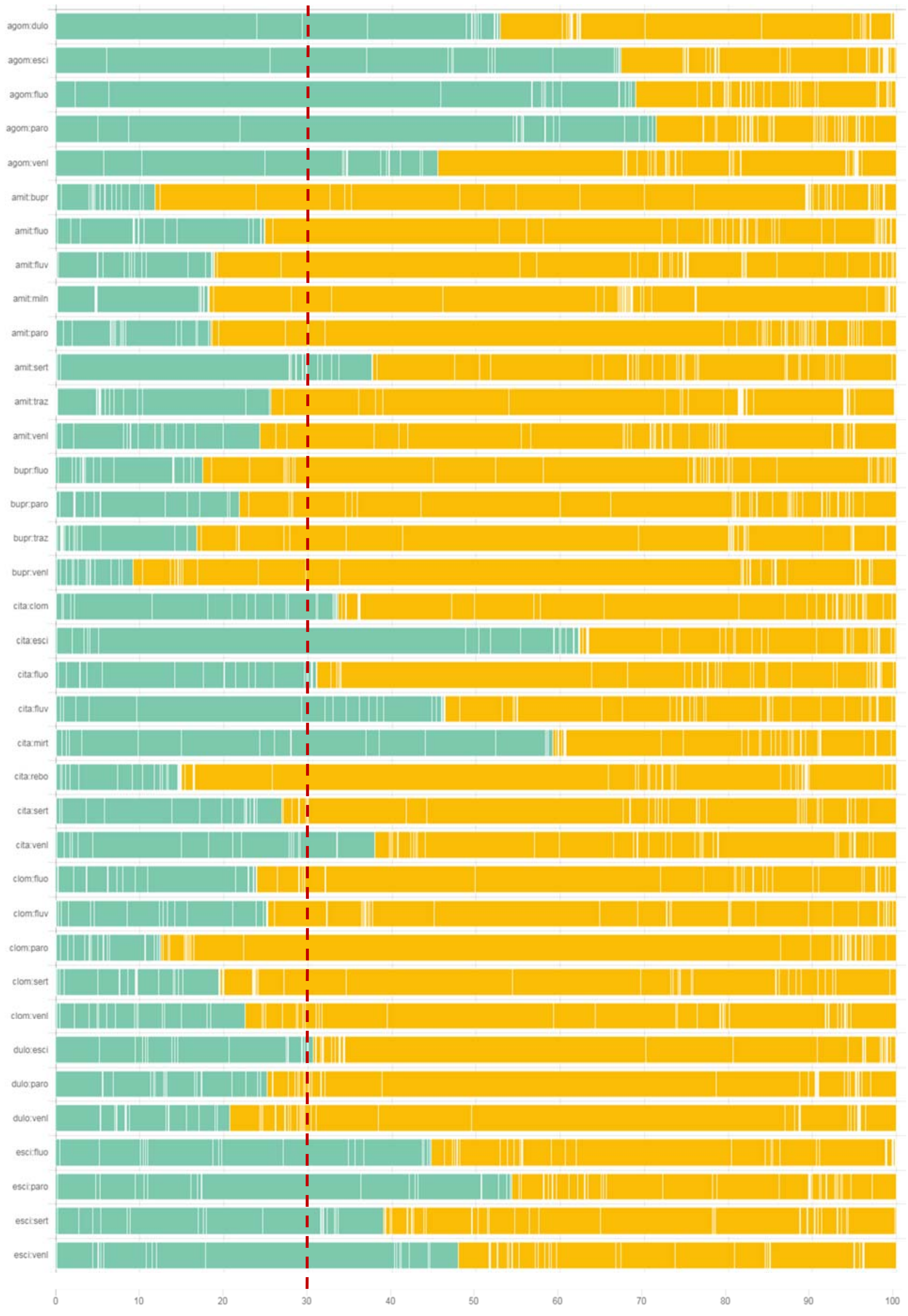
agom:dulo
agom:esci
agom:fluo
agom:paro
agom:venl
amit:bupr
amit:fluo
amit:fluv
amit:miln
amit:paro
amit:sert
amit:traz
amit:venl
bupr:fluo
bupr:paro
bupr:traz
bupr:venl
cita:clom
cita:esci
cita:fluo
cita:fluv
cita:mirt
cita:rebo
cita:sert
cita:venl
clom:fluo
clom:fluv
clom:paro
clom:sert
clom:venl
dulo:esci
dulo:paro
dulo:venl
esci:fluo
esci:paro
esci:sert
esci:venl
0
10
20
30
40
50
60
70
80
90
100

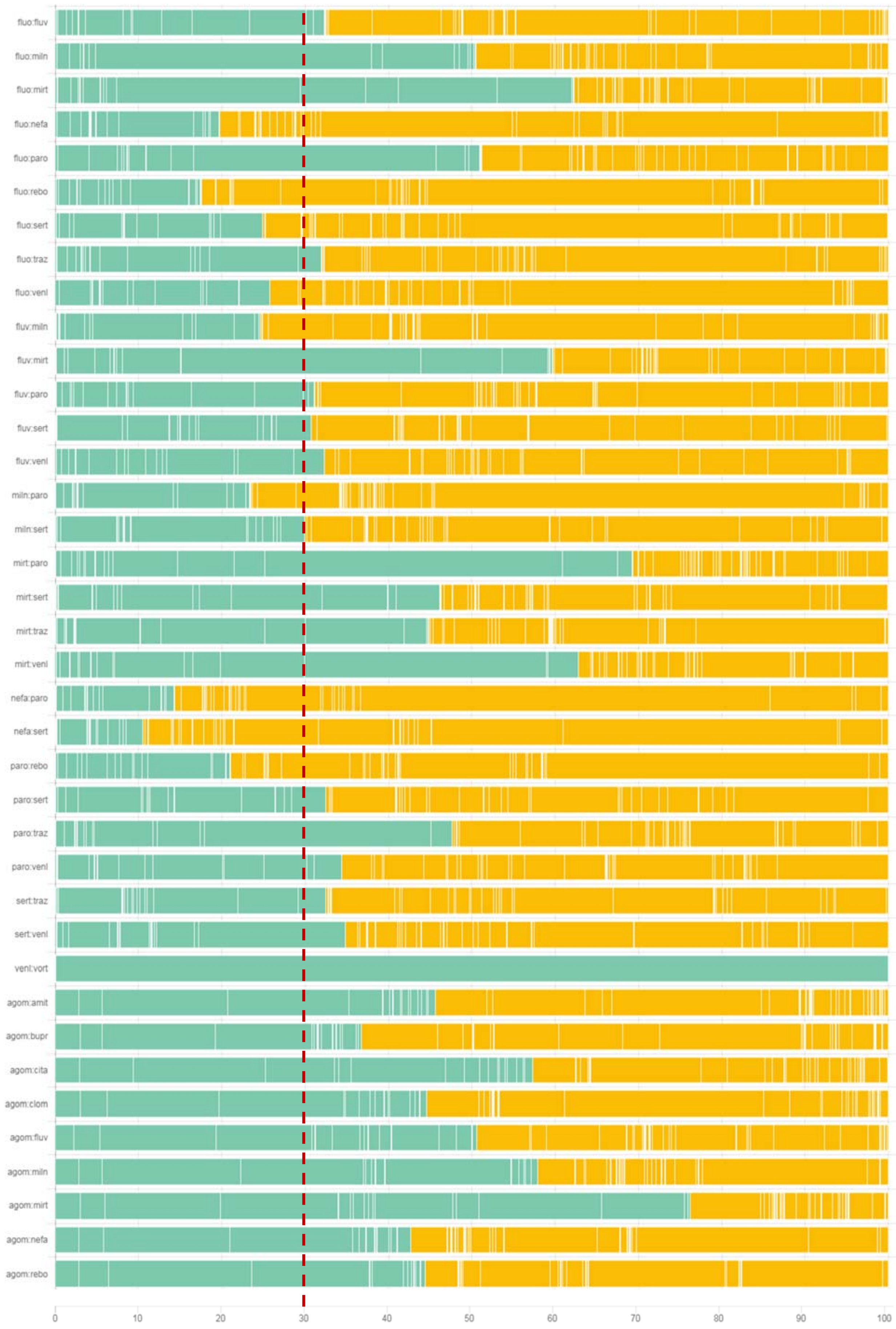
fluo:fluv
fluo:miln
fluo:mirt
fluo:nefa
fluo:paro
fluo:rebo
fluo:sert
fluo:traz
fluo:venl
fluv:miln
fluv:mirt
fluv:paro
fluv:sert
fluv:venl
miln:paro
miln:sert
mirt:paro
mirt:sert
mirt:traz
mirt:venl
nefa:paro
nefa:sert
paro:rebo
paro:sert
paro:traz
paro:venl
sert:traz
sert:venl
venl:vort
agom:amit
agom:bupr
agom:cita
agom:clom
agom:fluv
agom:miln
agom:mirt
agom:nefa
agom:rebo
0
10
20
30
40
50
60
70
80
90
100

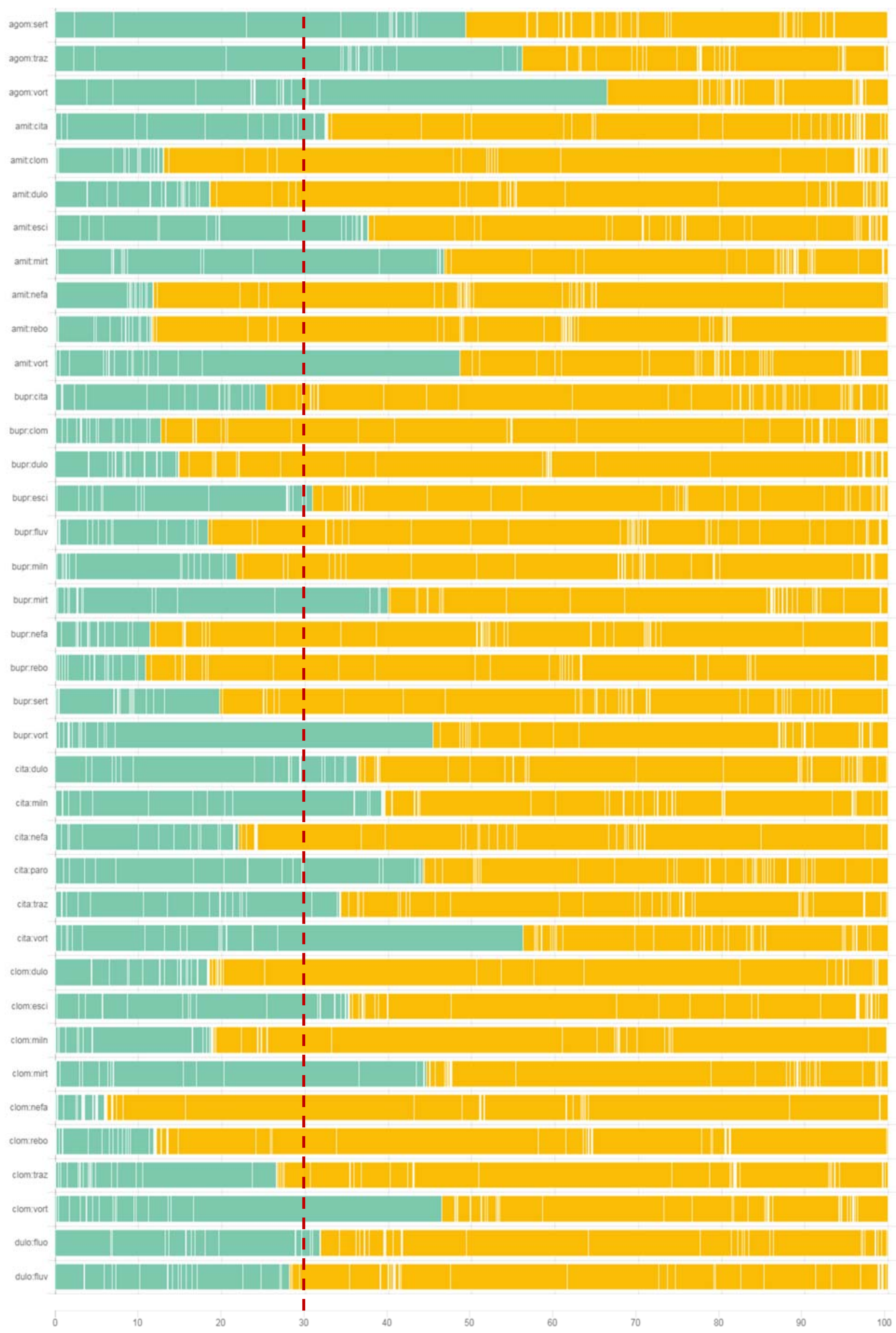
agom:sert
agom:traz
agom:vort
amit:cita
amit:clom
amit:dulo
amit:esci
amit:mirt
amit:nefa
amit:rebo
amit:vort
bupr:cita
bupr:clom
bupr:dulo
bupr:esci
bupr:fluv
bupr:miln
bupr:mirt
bupr:nefa
bupr:rebo
bupr:sert
bupr:vort
cita:dulo
cita:miln
cita:nefa
cita:paro
cita:traz
cita:vort
clom:dulo
clom:esci
clom:miln
clom:mirt
clom:nefa
clom:rebo
clom:traz
clom:vort
dulo:fluo
dulo:fluv
0
10
20
30
40
50
60
70
80
90
100

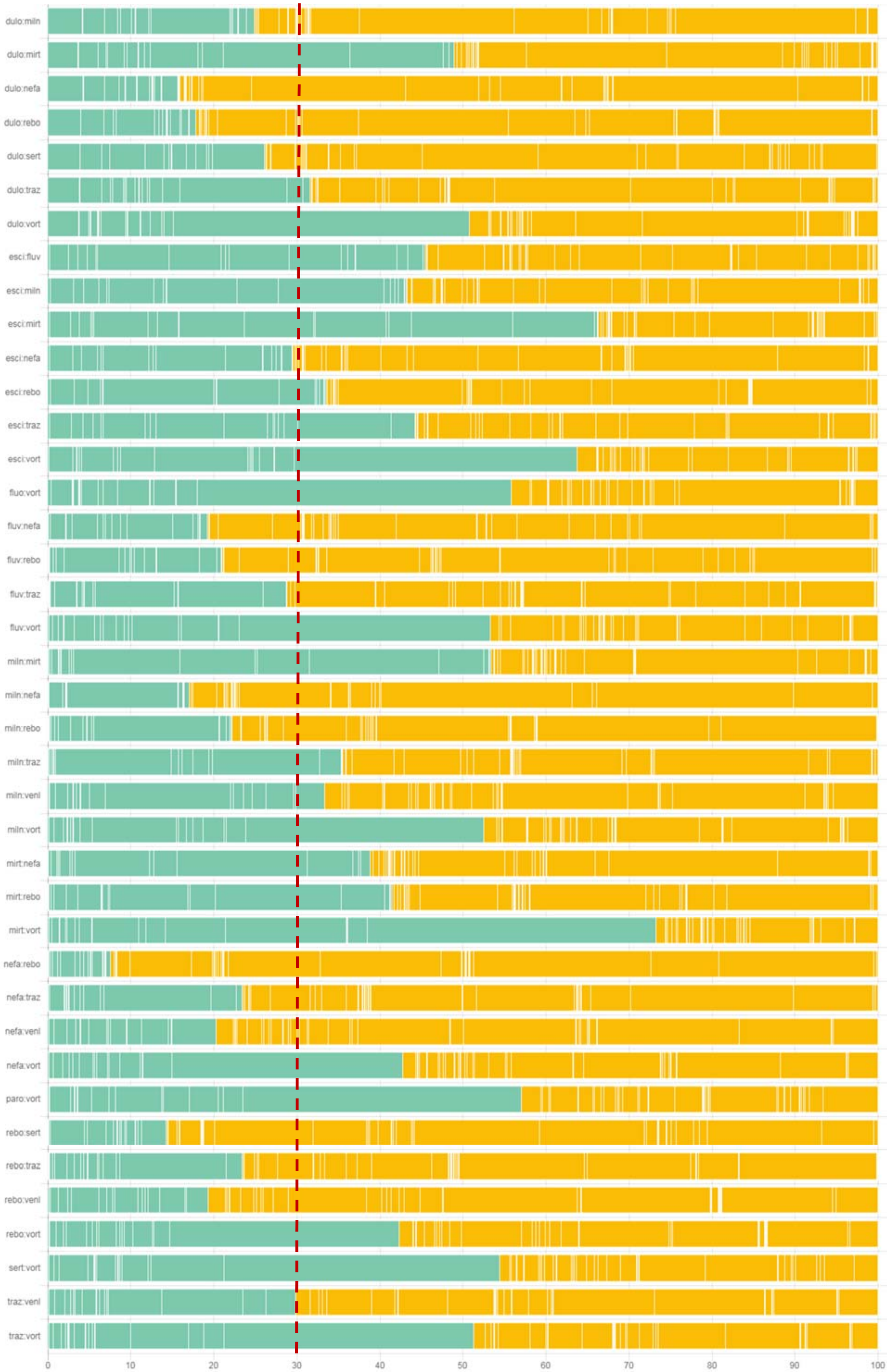

dulo:miln
dulo:mirt
dulo:nefa
dulo:rebo
dulo:sert
dulo:traz
dulo:vort
esci:fluv
esci:miln
esci:mirt
esci:nefa
esci:rebo
esci:traz
esci:vort
fluo:vort
fluv:nefa
fluv:rebo
fluv:traz
fluv:vort
miln:mirt
miln:nefa
miln:rebo
miln:traz
miln:venl
miln:vort
mirt:nefa
mirt:rebo
mirt:vort
nefa:rebo
nefa:traz
nefa:venl
nefa:vort
paro:vort
rebo:sert
rebo:traz
rebo:venl
rebo:vort
sert:vort
traz:venl
traz:vort
0
10
20
30
40
50
60
70
80
90
100

9.2.3 Table of reasons for downgrading

(1) **Study limitations**: We downgraded by one level when the contributions from low RoB comparisons were less than 30% and contributiions from moderate RoB comparisons were greater than 70%.

(2) **Imprecision**: We considered a clinically meaningful threshold for OR to be 0.80 or 1.25 and downgraded the estimate if the OR point estimate is 1 or more and the lower limit of its CrI is below 0.80; or if the OR point estimate is less than 1 and the upper limit of its CrI is above 1.25.

(3) **Inconsistency**: We rated two concepts, heterogeneity and incoherence (inconsistency), in this domain. For heterogeneity, we looked at the common tau and found that it is low compared to the expected value as reported in the literature (Turner RM et al (2012) Int J Epidemiol, 41, 818-827) , so we did not downgrade any network estimate for heterogeneity. For inconsistency, we looked at the results of side splitting (8.3.2.2) and we downgraded the comparisons with important inconsistency ($p<0.10$), where we have not downgraded for imprecision (we did not downgrade the same network estimate for both imprecision and inconsistency). The design-by-treatment test was also suggestive of inconsistency in the network (8.3.2.3).

(4) **Indirectness**: We have assured transitivity in our network by limiting the included studies to non-psychotic non-refractory unipolar major depression, because bipolarity, psychotic features, subthreshold depression and treatment refractoriness are established treatment modifiers for antidepressants. We further ran various meta-regressions, and assured that they did not violate transitivity of the network. However, we downgraded singly-connected nodes for indirectness because evaluation of transitivity for such nodes is unclear.

(5) **Publication bias**: The comparison-adjusted funnel plot (8.4.2) was not suggestive of funnel plot asymmetry in comparison with the old, standard drug fluoxetine. There was no network estimate for which the meta-regression for small-study effects suggested the statistically significant influence of small-study effects (8.5.2.2). We managed to retrieve supplementary and unpublished information included in the available systematic reviews and network meta-analyses, and we are confident that we have all available information that is possible to capture from clinical trial registries, regulatory agencies' repositories and drug companies' websites (particularly for the newest and most recently marketed antidepressants). However, we cannot completely rule out the possibility that some studies are still missing. Considering that the field of antidepressant trials in the past has been prone to publication bias, the review team decided by default to downgrade all the included studies for potential publication bias by one level.

	Study limitations	Imprecision	Inconsistency	Indirectness	Publication bias	GRADE
**Agom vs Amit**	No downgrade	No downgrade	No downgrade	No downgrade	Downgrade	MODERATE
**Agom vs Bupr**	No downgrade	No downgrade	No downgrade	No downgrade	Downgrade	MODERATE
**Agom vs Cita**	No downgrade	No downgrade	No downgrade	No downgrade	Downgrade	MODERATE
**Agom vs Clom**	No downgrade	No downgrade	No downgrade	No downgrade	Downgrade	MODERATE
**Agom vs Dulo**	No downgrade	No downgrade	Downgrade for side-splitting p=0.008	No downgrade	Downgrade	LOW
**Agom vs Esci**	No downgrade	No downgrade	No downgrade	No downgrade	Downgrade	MODERATE
**Agom vs Fluo**	No downgrade	No downgrade	Downgrade for side-splitting p=0.061	No downgrade	Downgrade	LOW
**Agom vs Fluv**	No downgrade	No downgrade	Downgrade for side-splitting p=0.091	No downgrade	Downgrade	LOW
**Agom vs Miln**	No downgrade	No downgrade	No downgrade	No downgrade	Downgrade	MODERATE
**Agom vs Mirt**	No downgrade	No downgrade	No downgrade	No downgrade	Downgrade	MODERATE
**Agom vs Nefa**	No downgrade	No downgrade	No downgrade	No downgrade	Downgrade	MODERATE
**Agom vs Paro**	No downgrade	No downgrade	No downgrade	No downgrade	Downgrade	MODERATE
**Agom vs Rebo**	No downgrade	No downgrade	No downgrade	No downgrade	Downgrade	MODERATE
**Agom vs Sert**	No downgrade	No downgrade	No downgrade	No downgrade	Downgrade	MODERATE
**Agom vs Traz**	No downgrade	No downgrade	No downgrade	No downgrade	Downgrade	MODERATE
**Agom vs Venl**	No downgrade	No downgrade	Downgrade for side-splitting p=0.040	No downgrade	Downgrade	LOW
**Agom vs Vort**	No downgrade	No downgrade	No downgrade	Downgrade because of concerns about transitivity.	Downgrade	LOW
**Amit vs Bupr**	Downgrade because >70% contribution from moderate RoB comparisons	Downgrade because point estimate >1.0 but lower limit <0.80	No downgrade	No downgrade	Downgrade	VERY LOW
**Amit vs Cita**	No downgrade	No downgrade	No downgrade	No downgrade	Downgrade	MODERATE
**Amit vs Clom**	Downgrade because >70% contribution from moderate RoB comparisons	No downgrade	No downgrade	No downgrade	Downgrade	LOW
**Amit vs Dulo**	Downgrade because >70% contribution from moderate RoB comparisons	No downgrade	No downgrade	No downgrade	Downgrade	LOW
**Amit vs Esci**	No downgrade	No downgrade	No downgrade	No downgrade	Downgrade	MODERATE
**Amit vs Fluo**	Downgrade because >70% contribution from moderate RoB comparisons	No downgrade	No downgrade	No downgrade	Downgrade	LOW

**Amit vs Fluv**	Downgrade because >70% contribution from moderate RoB comparisons	No downgrade	No downgrade	No downgrade	Downgrade	LOW
**Amit vs Miln**	Downgrade because >70% contribution from moderate RoB comparisons	No downgrade	No downgrade	No downgrade	Downgrade	LOW
**Amit vs Mirt**	No downgrade	No downgrade	No downgrade	No downgrade	Downgrade	MODERATE
**Amit vs Nefa**	Downgrade because >70% contribution from moderate RoB comparisons	Downgrade because point estimate <1.0 but upper limit >1.25	No downgrade	No downgrade	Downgrade	VERY LOW
**Amit vs Paro**	Downgrade because >70% contribution from moderate RoB comparisons	No downgrade	No downgrade	No downgrade	Downgrade	LOW
**Amit vs Rebo**	Downgrade because >70% contribution from moderate RoB comparisons	No downgrade	No downgrade	No downgrade	Downgrade	LOW
**Amit vs Sert**	No downgrade	No downgrade	No downgrade	No downgrade	Downgrade	MODERATE
**Amit vs Traz**	Downgrade because >70% contribution from moderate RoB comparisons	Downgrade because point estimate <1.0 but upper limit >1.25	No downgrade	No downgrade	Downgrade	VERY LOW
**Amit vs Venl**	Downgrade because >70% contribution from moderate RoB comparisons	No downgrade	No downgrade	No downgrade	Downgrade	LOW
**Amit vs Vort**	No downgrade	No downgrade	No downgrade	Downgrade because of concerns about transitivity.	Downgrade	LOW
**Bupr vs Cita**	Downgrade because >70% contribution from moderate RoB comparisons	Downgrade because point estimate >1.0 but lower limit <0.80	No downgrade	No downgrade	Downgrade	VERY LOW
**Bupr vs Clom**	Downgrade because >70% contribution from moderate RoB comparisons	No downgrade	No downgrade	No downgrade	Downgrade	LOW

**Bupr vs Dulo**	Downgrade because >70% contribution from moderate RoB comparisons	No downgrade	No downgrade	No downgrade	Downgrade	LOW
**Bupr vs Esci**	No downgrade	No downgrade	No downgrade	No downgrade	Downgrade	MODERATE
**Bupr vs Fluo**	Downgrade because >70% contribution from moderate RoB comparisons	Downgrade because point estimate >1.0 but lower limit <0.80	No downgrade	No downgrade	Downgrade	VERY LOW
**Bupr vs Fluv**	Downgrade because >70% contribution from moderate RoB comparisons	Downgrade because point estimate <1.0 but upper limit >1.25	No downgrade	No downgrade	Downgrade	VERY LOW
**Bupr vs Miln**	Downgrade because >70% contribution from moderate RoB comparisons	Downgrade because point estimate >1.0 but lower limit <0.80	No downgrade	No downgrade	Downgrade	VERY LOW
**Bupr vs Mirt**	No downgrade	Downgrade because point estimate >1.0 but lower limit <0.80	No downgrade	No downgrade	Downgrade	LOW
**Bupr vs Nefa**	Downgrade because >70% contribution from moderate RoB comparisons	Downgrade because point estimate <1.0 but upper limit >1.25	No downgrade	No downgrade	Downgrade	VERY LOW
**Bupr vs Paro**	Downgrade because >70% contribution from moderate RoB comparisons	Downgrade because point estimate >1.0 but lower limit <0.80	No downgrade	No downgrade	Downgrade	VERY LOW
**Bupr vs Rebo**	Downgrade because >70% contribution from moderate RoB comparisons	No downgrade	No downgrade	No downgrade	Downgrade	LOW
**Bupr vs Sert**	Downgrade because >70% contribution from moderate RoB comparisons	Downgrade because point estimate >1.0 but lower limit <0.80	No downgrade	No downgrade	Downgrade	VERY LOW
**Bupr vs Traz**	Downgrade because >70% contribution from moderate RoB comparisons	Downgrade because point estimate <1.0 but upper limit >1.25	No downgrade	No downgrade	Downgrade	VERY LOW

**Bupr vs Venl**	Downgrade because >70% contribution from moderate RoB comparisons	Downgrade because point estimate <1.0 but upper limit >1.25	No downgrade	No downgrade	Downgrade	VERY LOW
**Bupr vs Vort**	No downgrade	No downgrade	No downgrade	Downgrade because of concerns about transitivity.	Downgrade	LOW
**Cita vs Clom**	No downgrade	No downgrade	Downgrade for side-splitting p=0.056	No downgrade	Downgrade	LOW
**Cita vs Dulo**	No downgrade	No downgrade	No downgrade	No downgrade	Downgrade	MODERATE
**Cita vs Esci**	No downgrade	No downgrade	No downgrade	No downgrade	Downgrade	MODERATE
**Cita vs Fluo**	No downgrade	No downgrade	No downgrade	No downgrade	Downgrade	MODERATE
**Cita vs Fluv**	No downgrade	No downgrade	No downgrade	No downgrade	Downgrade	MODERATE
**Cita vs Miln**	No downgrade	No downgrade	No downgrade	No downgrade	Downgrade	MODERATE
**Cita vs Mirt**	No downgrade	No downgrade	Downgrade for side-splitting p=0.069	No downgrade	Downgrade	LOW
**Cita vs Nefa**	Downgrade because >70% contribution from moderate RoB comparisons	Downgrade because point estimate <1.0 but upper limit >1.25	No downgrade	No downgrade	Downgrade	VERY LOW
**Cita vs Paro**	No downgrade	No downgrade	No downgrade	No downgrade	Downgrade	MODERATE
**Cita vs Rebo**	Downgrade because >70% contribution from moderate RoB comparisons	No downgrade	No downgrade	No downgrade	Downgrade	LOW
**Cita vs Sert**	Downgrade because >70% contribution from moderate RoB comparisons	No downgrade	Downgrade for side-splitting p=0.039	No downgrade	Downgrade	VERY LOW
**Cita vs Traz**	No downgrade	No downgrade	No downgrade	No downgrade	Downgrade	MODERATE
**Cita vs Venl**	No downgrade	No downgrade	Downgrade for side-splitting p=0.016	No downgrade	Downgrade	LOW
**Cita vs Vort**	No downgrade	No downgrade	No downgrade	Downgrade because of concerns about transitivity.	Downgrade	LOW
**Clom vs Dulo**	Downgrade because >70% contribution from moderate RoB comparisons	No downgrade	No downgrade	No downgrade	Downgrade	LOW
**Clom vs Esci**	No downgrade	No downgrade	No downgrade	No downgrade	Downgrade	MODERATE
**Clom vs Fluo**	Downgrade because >70% contribution from moderate RoB comparisons	No downgrade	No downgrade	No downgrade	Downgrade	LOW

**Clom vs Fluv**	Downgrade because >70% contribution from moderate RoB comparisons	No downgrade	No downgrade	No downgrade	Downgrade	LOW
**Clom vs Miln**	Downgrade because >70% contribution from moderate RoB comparisons	No downgrade	No downgrade	No downgrade	Downgrade	LOW
**Clom vs Mirt**	No downgrade	No downgrade	No downgrade	No downgrade	Downgrade	MODERATE
**Clom vs Nefa**	Downgrade because >70% contribution from moderate RoB comparisons	Downgrade because point estimate >1.0 but lower limit <0.80	No downgrade	No downgrade	Downgrade	VERY LOW
**Clom vs Paro**	Downgrade because >70% contribution from moderate RoB comparisons	No downgrade	No downgrade	No downgrade	Downgrade	LOW
**Clom vs Rebo**	Downgrade because >70% contribution from moderate RoB comparisons	Downgrade because point estimate <1.0 but upper limit >1.25	No downgrade	No downgrade	Downgrade	VERY LOW
**Clom vs Sert**	Downgrade because >70% contribution from moderate RoB comparisons	No downgrade	No downgrade	No downgrade	Downgrade	LOW
**Clom vs Traz**	Downgrade because >70% contribution from moderate RoB comparisons	No downgrade	No downgrade	No downgrade	Downgrade	LOW
**Clom vs Venl**	Downgrade because >70% contribution from moderate RoB comparisons	No downgrade	No downgrade	No downgrade	Downgrade	LOW
**Clom vs Vort**	No downgrade	No downgrade	No downgrade	Downgrade because of concerns about transitivity.	Downgrade	LOW
**Dulo vs Esci**	No downgrade	No downgrade	No downgrade	No downgrade	Downgrade	MODERATE
**Dulo vs Fluo**	No downgrade	No downgrade	No downgrade	No downgrade	Downgrade	MODERATE
**Dulo vs Fluv**	Downgrade because >70% contribution from moderate RoB comparisons	No downgrade	No downgrade	No downgrade	Downgrade	LOW

**Dulo vs Miln**	Downgrade because >70% contribution from moderate RoB comparisons	No downgrade	No downgrade	No downgrade	Downgrade	LOW
**Dulo vs Mirt**	No downgrade	No downgrade	No downgrade	No downgrade	Downgrade	MODERATE
**Dulo vs Nefa**	Downgrade because >70% contribution from moderate RoB comparisons	Downgrade because point estimate >1.0 but lower limit <0.80	No downgrade	No downgrade	Downgrade	VERY LOW
**Dulo vs Paro**	Downgrade because >70% contribution from moderate RoB comparisons	No downgrade	No downgrade	No downgrade	Downgrade	LOW
**Dulo vs Rebo**	Downgrade because >70% contribution from moderate RoB comparisons	Downgrade because point estimate <1.0 but upper limit >1.25	No downgrade	No downgrade	Downgrade	VERY LOW
**Dulo vs Sert**	Downgrade because >70% contribution from moderate RoB comparisons	No downgrade	No downgrade	No downgrade	Downgrade	LOW
**Dulo vs Traz**	No downgrade	Downgrade because point estimate >1.0 but lower limit <0.80	No downgrade	No downgrade	Downgrade	LOW
**Dulo vs Venl**	Downgrade because >70% contribution from moderate RoB comparisons	No downgrade	Downgrade for side-splitting p=0.052	No downgrade	Downgrade	VERY LOW
**Dulo vs Vort**	No downgrade	No downgrade	No downgrade	Downgrade because of concerns about transitivity.	Downgrade	LOW
**Esci vs Fluo**	No downgrade	No downgrade	No downgrade	No downgrade	Downgrade	MODERATE
**Esci vs Fluv**	No downgrade	No downgrade	No downgrade	No downgrade	Downgrade	MODERATE
**Esci vs Miln**	No downgrade	No downgrade	No downgrade	No downgrade	Downgrade	MODERATE
**Esci vs Mirt**	No downgrade	No downgrade	No downgrade	No downgrade	Downgrade	MODERATE
**Esci vs Nefa**	Downgrade because >70% contribution from moderate RoB comparisons	No downgrade	No downgrade	No downgrade	Downgrade	LOW
**Esci vs Paro**	No downgrade	No downgrade	No downgrade	No downgrade	Downgrade	MODERATE
**Esci vs Rebo**	No downgrade	No downgrade	No downgrade	No downgrade	Downgrade	MODERATE
**Esci vs Sert**	No downgrade	No downgrade	No downgrade	No downgrade	Downgrade	MODERATE
**Esci vs Traz**	No downgrade	No downgrade	No downgrade	No downgrade	Downgrade	MODERATE
**Esci vs Venl**	No downgrade	No downgrade	No downgrade	No downgrade	Downgrade	MODERATE

**Esci vs Vort**	No downgrade	Downgrade because point estimate >1.0 but lower limit <0.80	No downgrade	Downgrade because of concerns about transitivity.	Downgrade	VERY LOW
**Fluo vs Fluv**	No downgrade	No downgrade	No downgrade	No downgrade	Downgrade	MODERATE
**Fluo vs Miln**	No downgrade	No downgrade	No downgrade	No downgrade	Downgrade	MODERATE
**Fluo vs Mirt**	No downgrade	No downgrade	No downgrade	No downgrade	Downgrade	MODERATE
**Fluo vs Nefa**	Downgrade because >70% contribution from moderate RoB comparisons	No downgrade	No downgrade	No downgrade	Downgrade	LOW
**Fluo vs Paro**	No downgrade	No downgrade	No downgrade	No downgrade	Downgrade	MODERATE
**Fluo vs Rebo**	Downgrade because >70% contribution from moderate RoB comparisons	No downgrade	No downgrade	No downgrade	Downgrade	LOW
**Fluo vs Sert**	Downgrade because >70% contribution from moderate RoB comparisons	No downgrade	No downgrade	No downgrade	Downgrade	LOW
**Fluo vs Traz**	No downgrade	No downgrade	No downgrade	No downgrade	Downgrade	MODERATE
**Fluo vs Venl**	Downgrade because >70% contribution from moderate RoB comparisons	No downgrade	No downgrade	No downgrade	Downgrade	LOW
**Fluo vs Vort**	No downgrade	No downgrade	No downgrade	Downgrade because of concerns about transitivity.	Downgrade	LOW
**Fluv vs Miln**	Downgrade because >70% contribution from moderate RoB comparisons	No downgrade	No downgrade	No downgrade	Downgrade	LOW
**Fluv vs Mirt**	No downgrade	No downgrade	No downgrade	No downgrade	Downgrade	MODERATE
**Fluv vs Nefa**	Downgrade because >70% contribution from moderate RoB comparisons	Downgrade because point estimate >1.0 but lower limit <0.80	No downgrade	No downgrade	Downgrade	VERY LOW
**Fluv vs Paro**	No downgrade	No downgrade	No downgrade	No downgrade	Downgrade	MODERATE
**Fluv vs Rebo**	Downgrade because >70% contribution from moderate RoB comparisons	No downgrade	No downgrade	No downgrade	Downgrade	LOW
**Fluv vs Sert**	No downgrade	No downgrade	No downgrade	No downgrade	Downgrade	MODERATE

**Fluv vs Traz**	Downgrade because >70% contribution from moderate RoB comparisons	Downgrade because point estimate >1.0 but lower limit <0.80	No downgrade	No downgrade	Downgrade	VERY LOW
**Fluv vs Venl**	No downgrade	No downgrade	No downgrade	No downgrade	Downgrade	MODERATE
**Fluv vs Vort**	No downgrade	No downgrade	No downgrade	Downgrade because of concerns about transitivity.	Downgrade	LOW
**Miln vs Mirt**	No downgrade	Downgrade because point estimate >1.0 but lower limit <0.80	No downgrade	No downgrade	Downgrade	LOW
**Miln vs Nefa**	Downgrade because >70% contribution from moderate RoB comparisons	Downgrade because point estimate <1.0 but upper limit >1.25	No downgrade	No downgrade	Downgrade	VERY LOW
**Miln vs Paro**	Downgrade because >70% contribution from moderate RoB comparisons	Downgrade because point estimate <1.0 but upper limit >1.25	No downgrade	No downgrade	Downgrade	VERY LOW
**Miln vs Rebo**	Downgrade because >70% contribution from moderate RoB comparisons	No downgrade	No downgrade	No downgrade	Downgrade	LOW
**Miln vs Sert**	No downgrade	No downgrade	No downgrade	No downgrade	Downgrade	MODERATE
**Miln vs Traz**	No downgrade	No downgrade	No downgrade	No downgrade	Downgrade	MODERATE
**Miln vs Venl**	No downgrade	No downgrade	No downgrade	No downgrade	Downgrade	MODERATE
**Miln vs Vort**	No downgrade	No downgrade	No downgrade	Downgrade because of concerns about transitivity.	Downgrade	LOW
**Mirt vs Nefa**	No downgrade	Downgrade because point estimate <1.0 but upper limit >1.25	No downgrade	No downgrade	Downgrade	LOW
**Mirt vs Paro**	No downgrade	No downgrade	No downgrade	No downgrade	Downgrade	MODERATE
**Mirt vs Rebo**	No downgrade	No downgrade	No downgrade	No downgrade	Downgrade	MODERATE
**Mirt vs Sert**	No downgrade	No downgrade	No downgrade	No downgrade	Downgrade	MODERATE
**Mirt vs Traz**	No downgrade	No downgrade	No downgrade	No downgrade	Downgrade	MODERATE
**Mirt vs Venl**	No downgrade	No downgrade	No downgrade	No downgrade	Downgrade	MODERATE
**Mirt vs Vort**	No downgrade	No downgrade	No downgrade	Downgrade because of concerns about transitivity.	Downgrade	LOW

**Nefa vs Paro**	Downgrade because >70% contribution from moderate RoB comparisons	Downgrade because point estimate >1.0 but lower limit <0.80	No downgrade	No downgrade	Downgrade	VERY LOW
**Nefa vs Rebo**	Downgrade because >70% contribution from moderate RoB comparisons	Downgrade because point estimate <1.0 but upper limit >1.25	No downgrade	No downgrade	Downgrade	VERY LOW
**Nefa vs Sert**	Downgrade because >70% contribution from moderate RoB comparisons	No downgrade	No downgrade	No downgrade	Downgrade	LOW
**Nefa vs Traz**	Downgrade because >70% contribution from moderate RoB comparisons	Downgrade because point estimate <1.0 but upper limit >1.25	No downgrade	No downgrade	Downgrade	VERY LOW
**Nefa vs Venl**	Downgrade because >70% contribution from moderate RoB comparisons	Downgrade because point estimate <1.0 but upper limit >1.25	No downgrade	No downgrade	Downgrade	VERY LOW
**Nefa vs Vort**	No downgrade	No downgrade	No downgrade	Downgrade because of concerns about transitivity.	Downgrade	LOW
**Paro vs Rebo**	Downgrade because >70% contribution from moderate RoB comparisons	No downgrade	No downgrade	No downgrade	Downgrade	LOW
**Paro vs Sert**	No downgrade	No downgrade	No downgrade	No downgrade	Downgrade	MODERATE
**Paro vs Traz**	No downgrade	No downgrade	No downgrade	No downgrade	Downgrade	MODERATE
**Paro vs Venl**	No downgrade	No downgrade	No downgrade	No downgrade	Downgrade	MODERATE
**Paro vs Vort**	No downgrade	No downgrade	No downgrade	Downgrade because of concerns about transitivity.	Downgrade	LOW
**Rebo vs Sert**	Downgrade because >70% contribution from moderate RoB comparisons	No downgrade	No downgrade	No downgrade	Downgrade	LOW
**Rebo vs Traz**	Downgrade because >70% contribution from moderate RoB comparisons	No downgrade	No downgrade	No downgrade	Downgrade	LOW
**Rebo vs Venl**	Downgrade because >70% contribution from moderate RoB comparisons	No downgrade	No downgrade	No downgrade	Downgrade	LOW

**Rebo vs Vort**	No downgrade	No downgrade	No downgrade	Downgrade because of concerns about transitivity.	Downgrade	LOW
**Sert vs Traz**	No downgrade	No downgrade	No downgrade	No downgrade	Downgrade	MODERATE
**Sert vs Venl**	No downgrade	No downgrade	No downgrade	No downgrade	Downgrade	MODERATE
**Sert vs Vort**	No downgrade	No downgrade	No downgrade	Downgrade because of concerns about transitivity.	Downgrade	LOW
**Traz vs Venl**	Downgrade because >70% contribution from moderate RoB comparisons	Downgrade because point estimate >1.0 but lower limit <0.80	No downgrade	No downgrade	Downgrade	VERY LOW
**Traz vs Vort**	No downgrade	No downgrade	No downgrade	Downgrade because of concerns about transitivity.	Downgrade	LOW
**Venl vs Vort**	No downgrade	No downgrade	No downgrade	Downgrade because of concerns about transitivity.	Downgrade	LOW

10 SUCRA and cumulative probability plots

10.1 All trials

474 studies (with at least two arms at licensed dose), 106 966 participants

10.1.1 Response

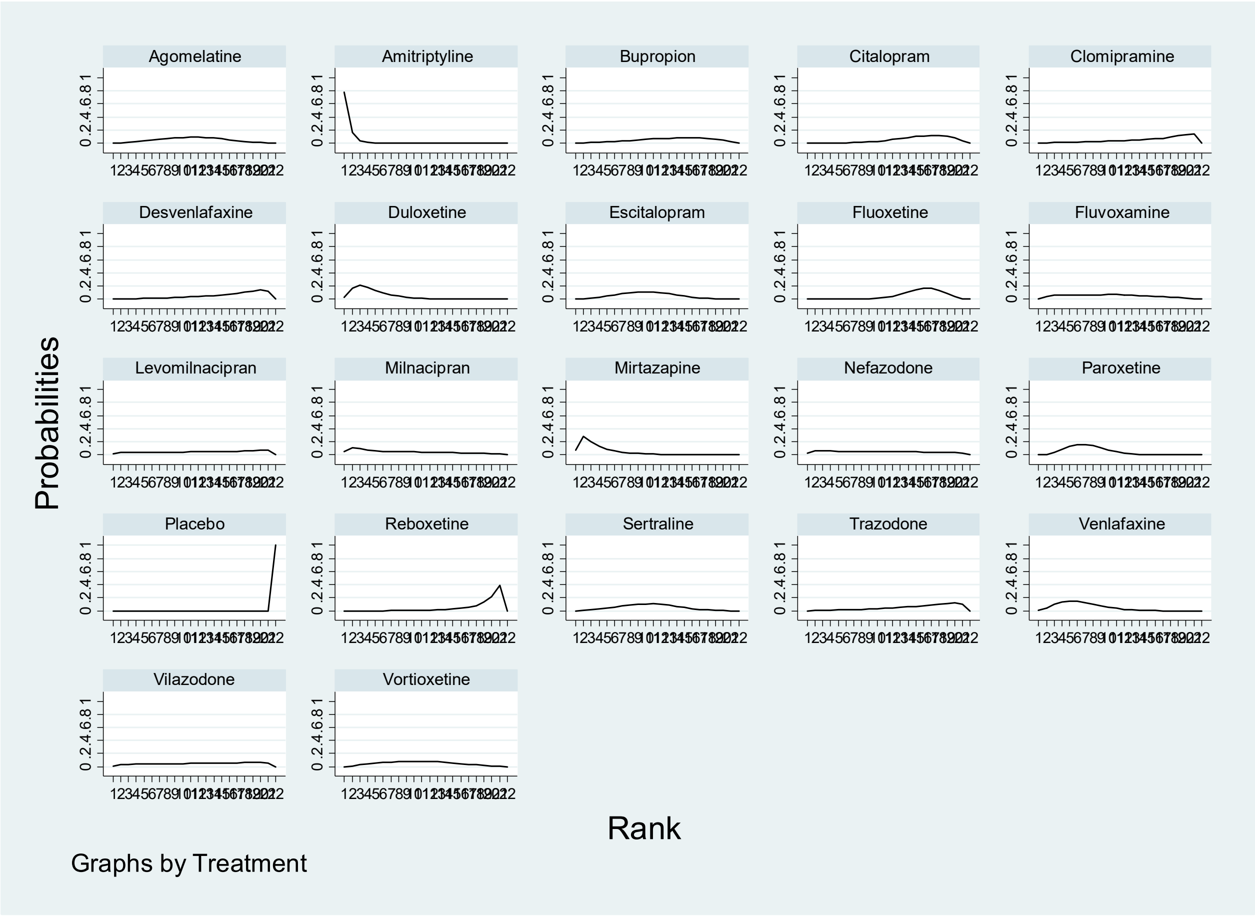

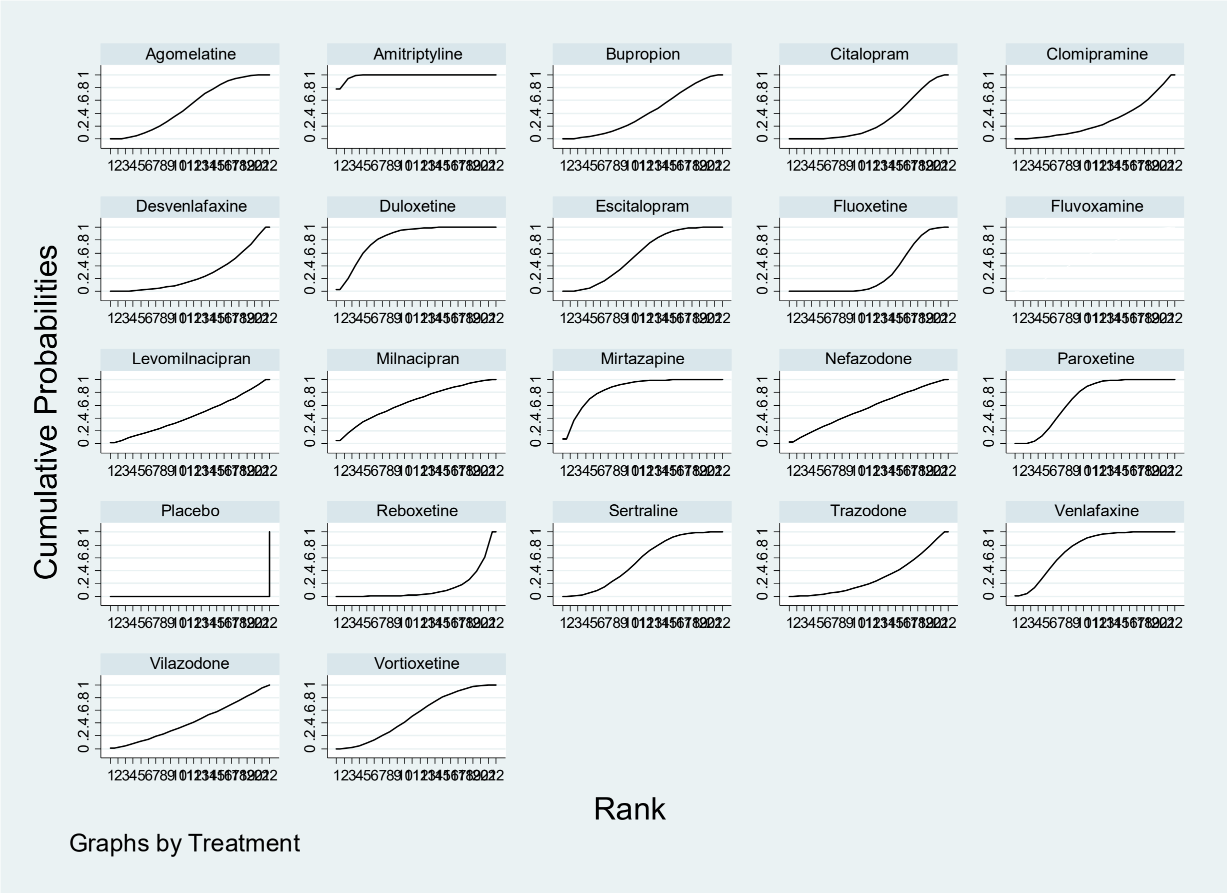

Treatment	SUCRA	PrBest	MeanRank
Placebo	0.0	0.0	22.0
Agomelatine	51.6	0.0	11.2
Amitriptyline	98.6	78.1	1.3
Bupropion	41.4	0.1	13.3
Citalopram	30.4	0.0	15.6
Clomipramine	29.4	0.1	15.8
Desvenlafaxine	27.7	0.0	16.2
Duloxetine	83.0	2.6	4.6
Escitalopram	56.9	0.0	10.0
Fluoxetine	29.2	0.0	15.9
Fluvoxamine	58.1	0.7	9.8
Levomilnacipran	44.5	1.6	12.7
Milnacipran	64.6	5.2	8.4
Mirtazapine	85.7	7.6	4.0
Nefazodone	55.3	2.7	10.4
Paroxetine	70.1	0.0	7.3
Reboxetine	13.6	0.0	19.1
Sertraline	55.2	0.0	10.4
Trazodone	30.8	0.0	15.5
Venlafaxine	74.3	0.2	6.4
Vilazodone	45.2	0.9	12.5
Vortioxetine	54.6	0.2	10.5

10.1.2 Dropouts for any reason

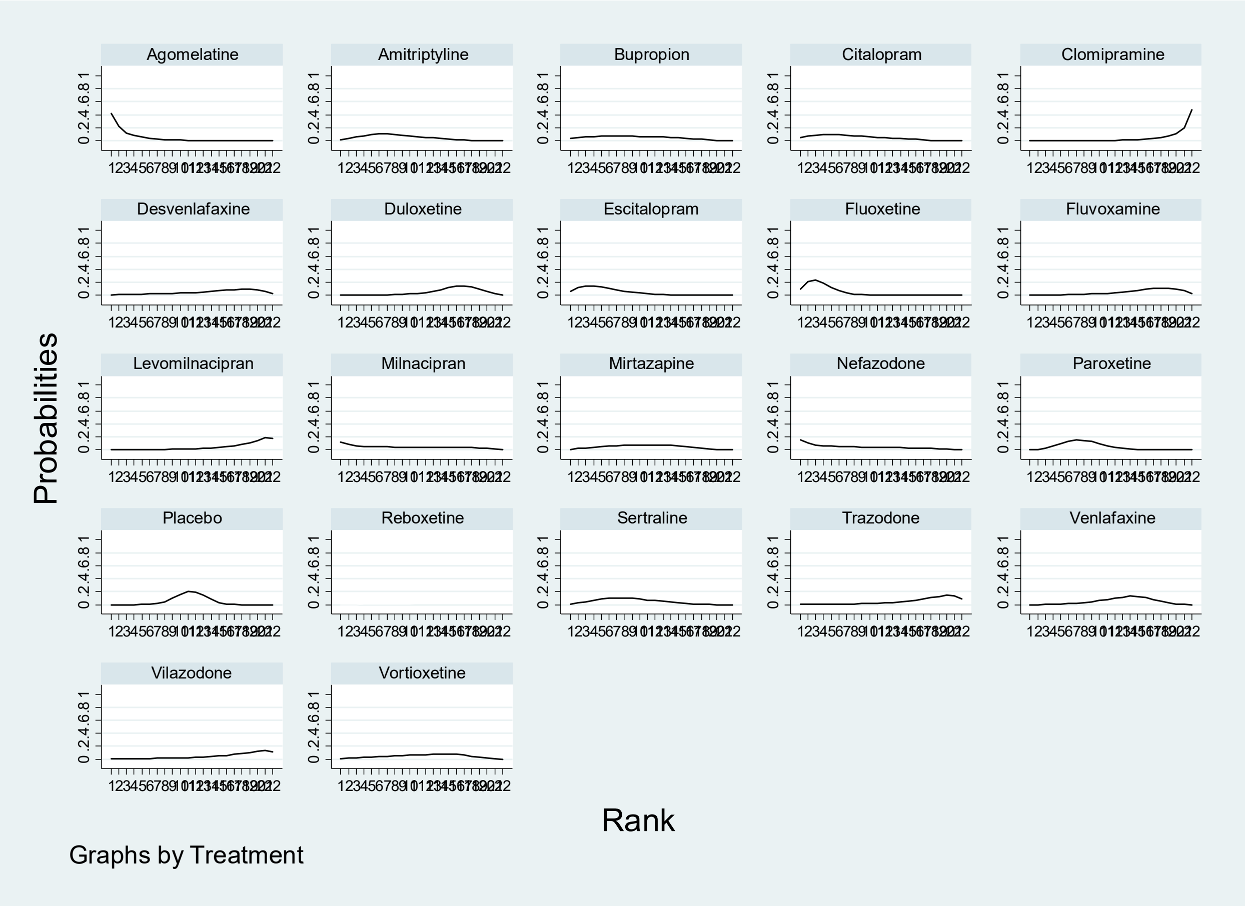

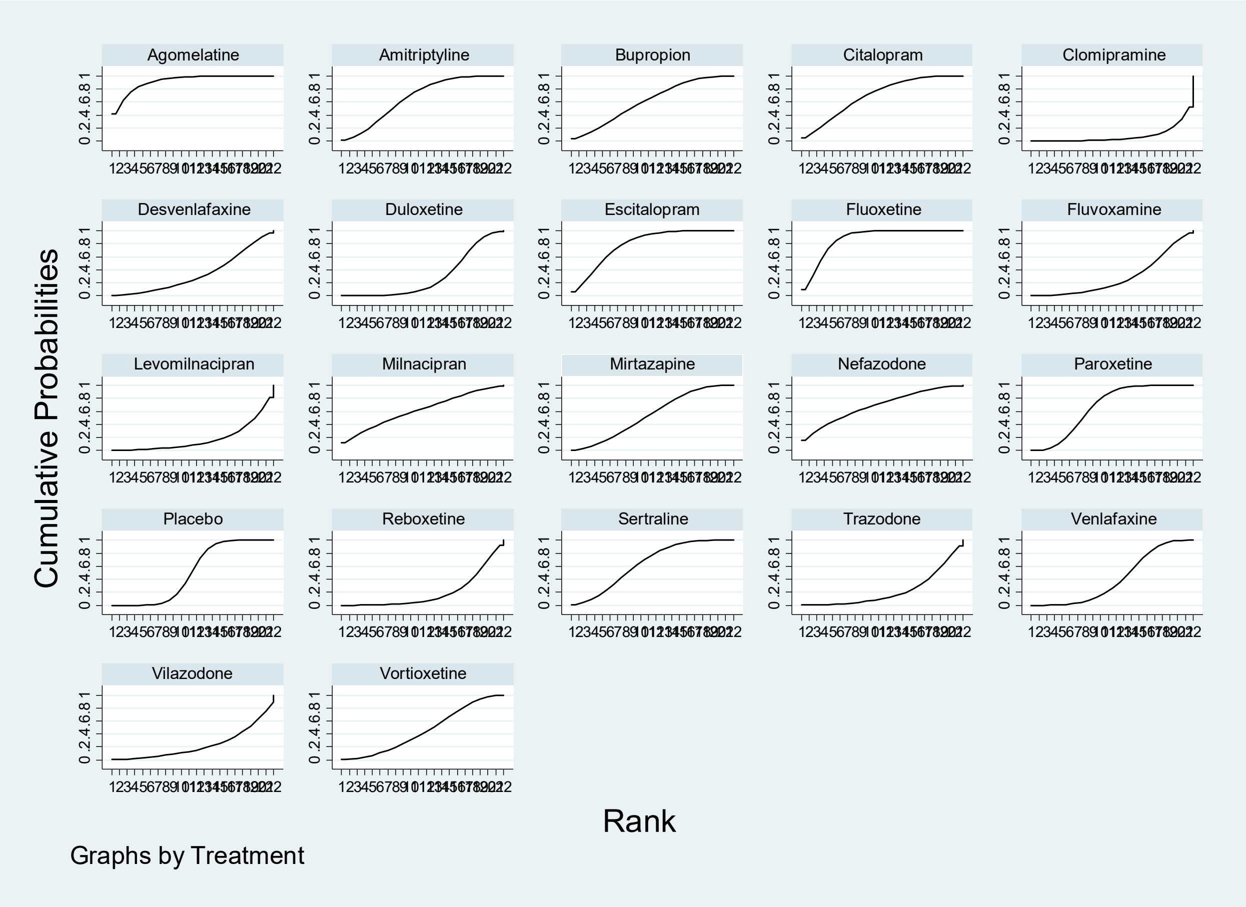

Treatment	SUCRA	PrBest	MeanRank
Placebo	50.7	0.0	11.3
Agomelatine	91.9	41.3	2.7
Amitriptyline	66.9	1.7	8.0
Bupropion	61.6	3.1	9.1
Citalopram	69.7	5.2	7.4
Clomipramine	7.9	0.0	20.3
Desvenlafaxine	34.1	0.6	14.8
Duloxetine	29.5	0.0	15.8
Escitalopram	79.5	6.5	5.3
Fluoxetine	87.4	9.1	3.6
Fluvoxamine	28.9	0.1	15.9
Levomilnacipran	18.0	0.2	18.2
Milnacipran	62.3	12.6	8.9
Mirtazapine	55.1	1.0	10.4
Nefazodone	69.0	15.9	7.5
Paroxetine	67.4	0.1	7.8
Reboxetine	19.7	0.0	17.9
Sertraline	64.2	1.1	8.5
Trazodone	22.2	0.1	17.3
Venlafaxine	40.8	0.0	13.4
Vilazodone	25.1	0.6	16.7
Vortioxetine	47.9	0.7	11.9

10.1.3 Efficacy as continuous outcome

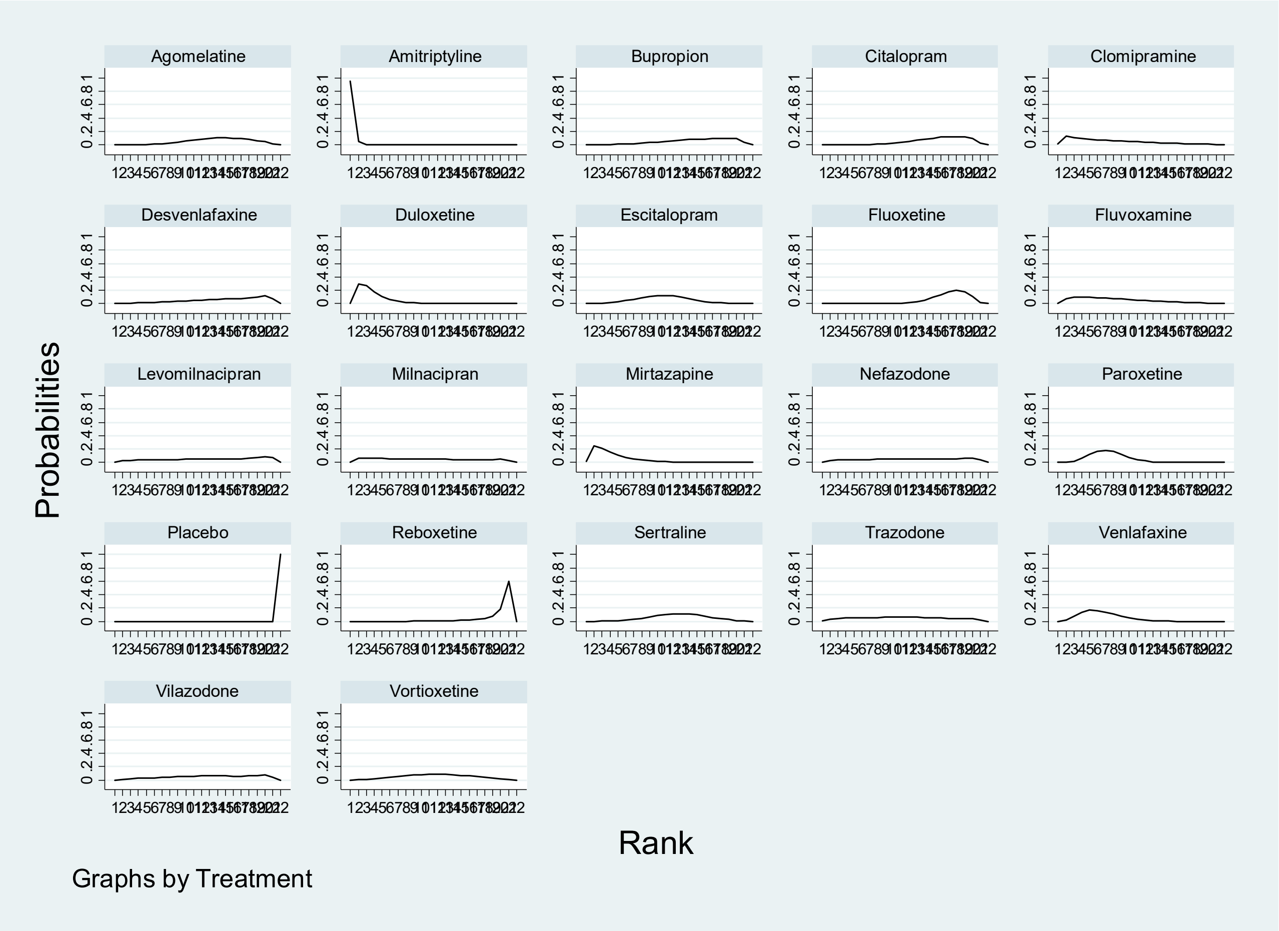

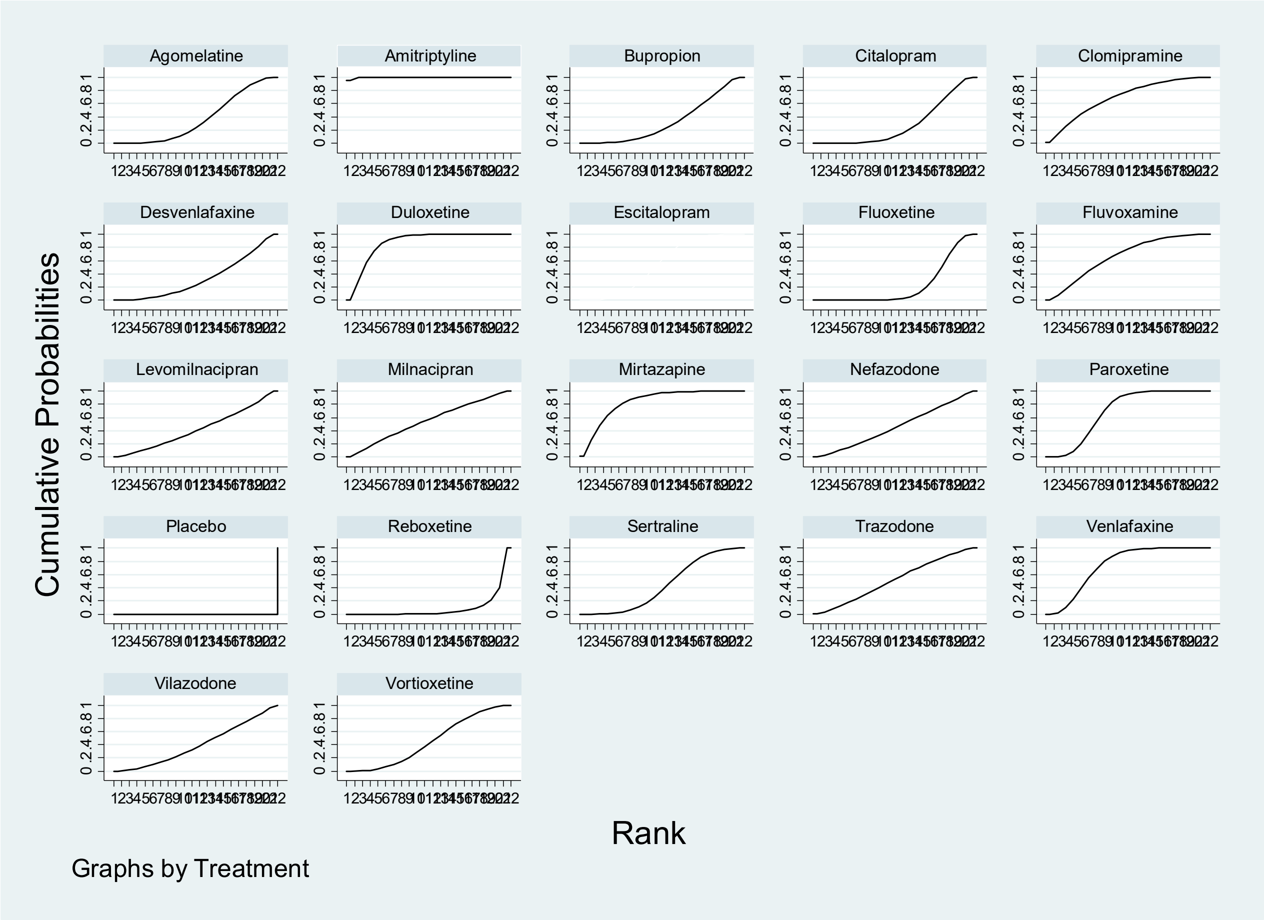

Treatment	SUCRA	PrBest	MeanRank
Placebo	0.0	0.0	22.0
Agomelatine	37.5	0.0	14.1
Amitriptyline	99.7	94.7	1.1
Bupropion	33.0	0.0	15.1
Citalopram	29.0	0.0	15.9
Clomipramine	69.3	1.5	7.4
Desvenlafaxine	33.1	0.0	15.0
Duloxetine	87.2	0.9	3.7
Escitalopram	54.1	0.0	10.6
Fluoxetine	22.9	0.0	17.2
Fluvoxamine	66.7	0.4	8.0
Levomilnacipran	42.9	0.3	13.0
Milnacipran	55.0	0.7	10.5
Mirtazapine	83.6	1.2	4.4
Nefazodone	46.4	0.2	12.3
Paroxetine	69.7	0.0	7.4
Reboxetine	9.7	0.0	20.0
Sertraline	44.1	0.0	12.7
Trazodone	51.7	0.2	11.1
Venlafaxine	74.0	0.0	6.5
Vilazodone	42.8	0.1	13.0
Vortioxetine	47.6	0.0	12.0

10.1.4 Remission

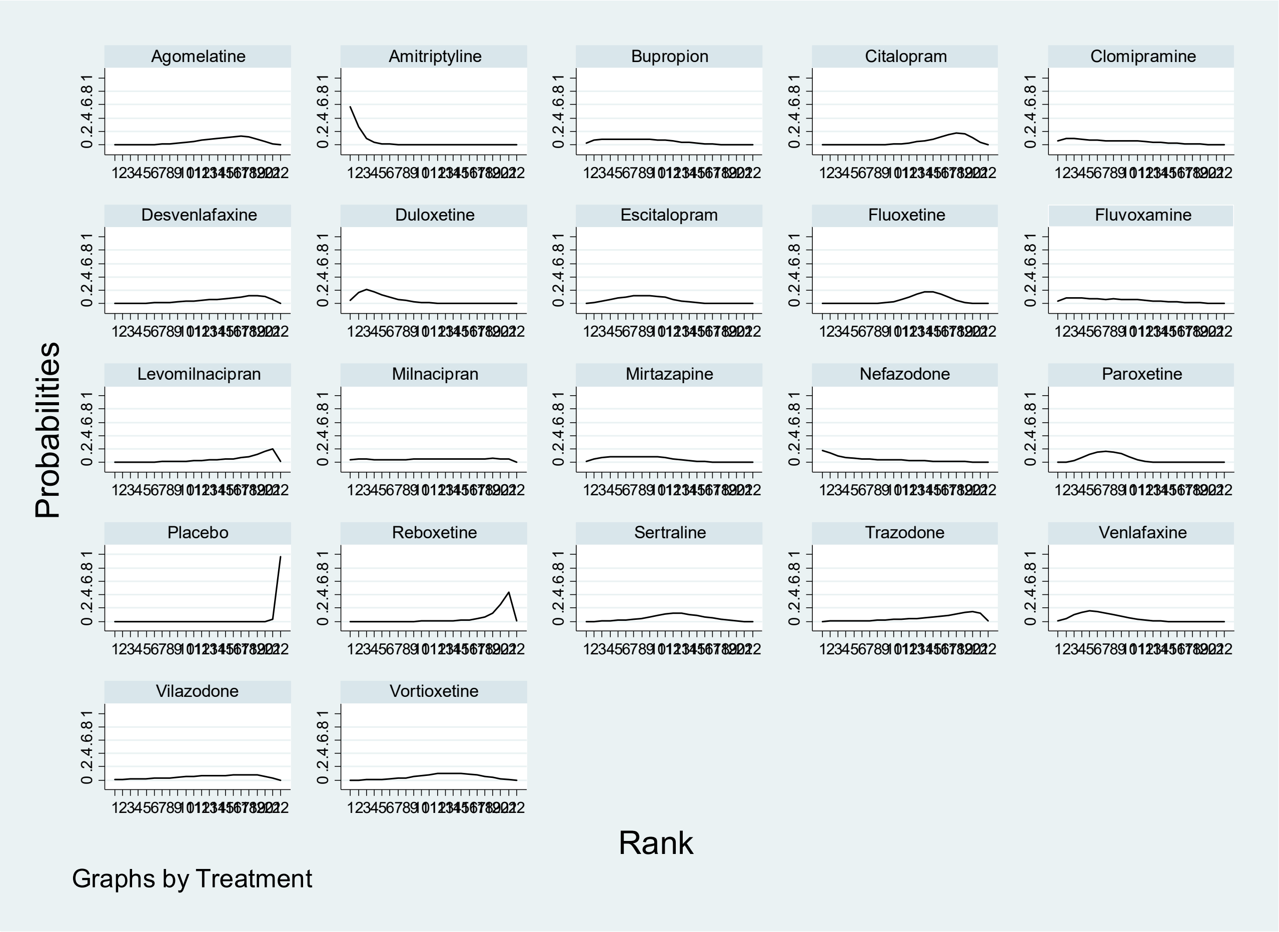

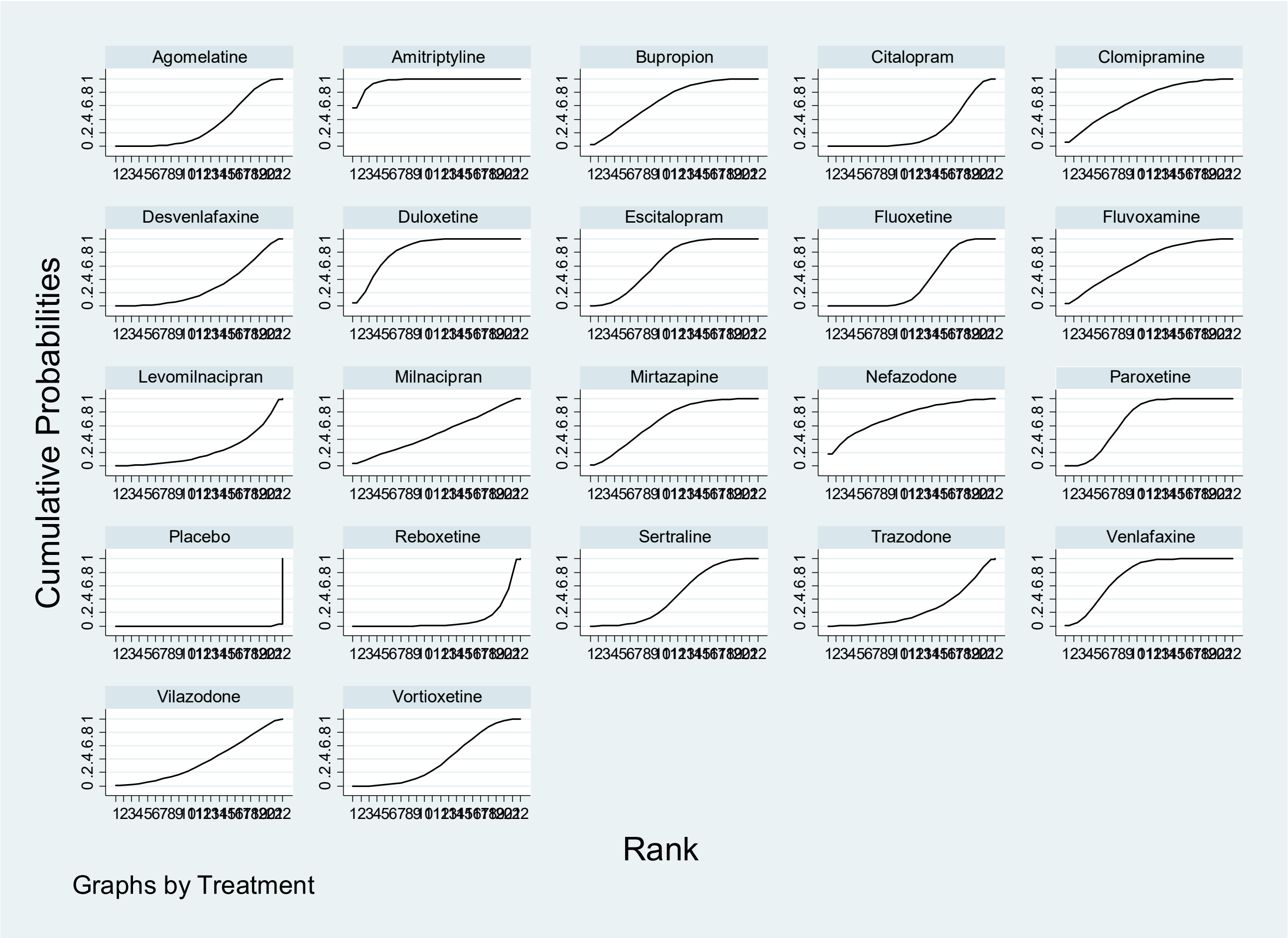

Treatment	SUCRA	PrBest	MeanRank
Placebo	0.1	0.0	22.0
Agomelatine	32.4	0.0	15.2
Amitriptyline	96.6	57.3	1.7
Bupropion	68.0	2.7	7.7
Citalopram	24.0	0.0	17.0
Clomipramine	69.2	6.3	7.5
Desvenlafaxine	29.9	0.0	15.7
Duloxetine	83.9	5.1	4.4
Escitalopram	65.3	0.2	8.3
Fluoxetine	36.5	0.0	14.3
Fluvoxamine	66.7	4.1	8.0
Levomilnacipran	24.0	0.2	17.0
Milnacipran	49.6	3.6	11.6
Mirtazapine	67.7	1.6	7.8
Nefazodone	74.6	17.7	6.3
Paroxetine	70.3	0.0	7.2
Reboxetine	10.9	0.0	19.7
Sertraline	46.3	0.0	12.3
Trazodone	26.2	0.0	16.5
Venlafaxine	75.3	0.6	6.2
Vilazodone	40.7	0.5	13.5
Vortioxetine	41.8	0.0	13.2

10.1.5 Dropouts due to adverse events

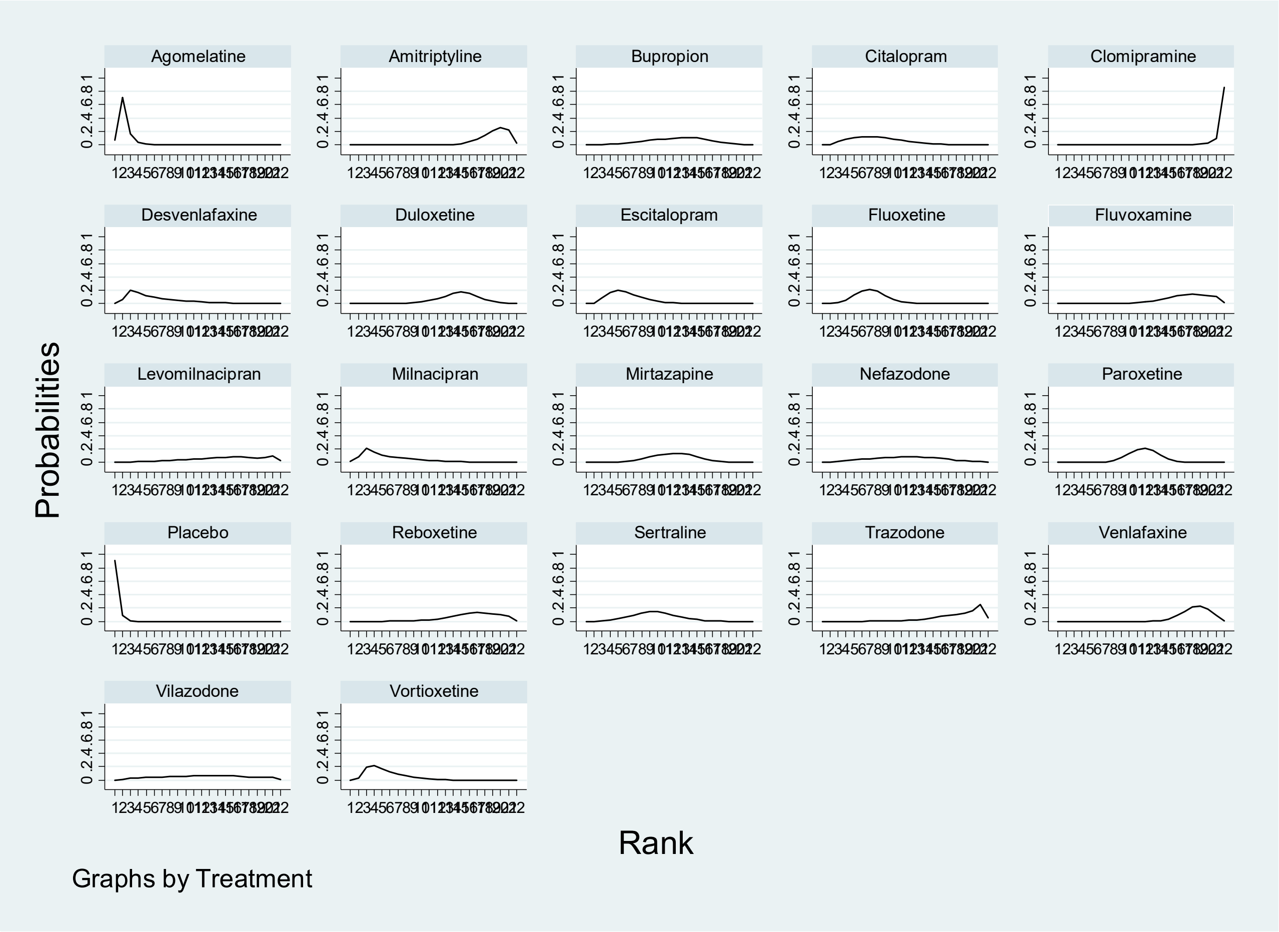

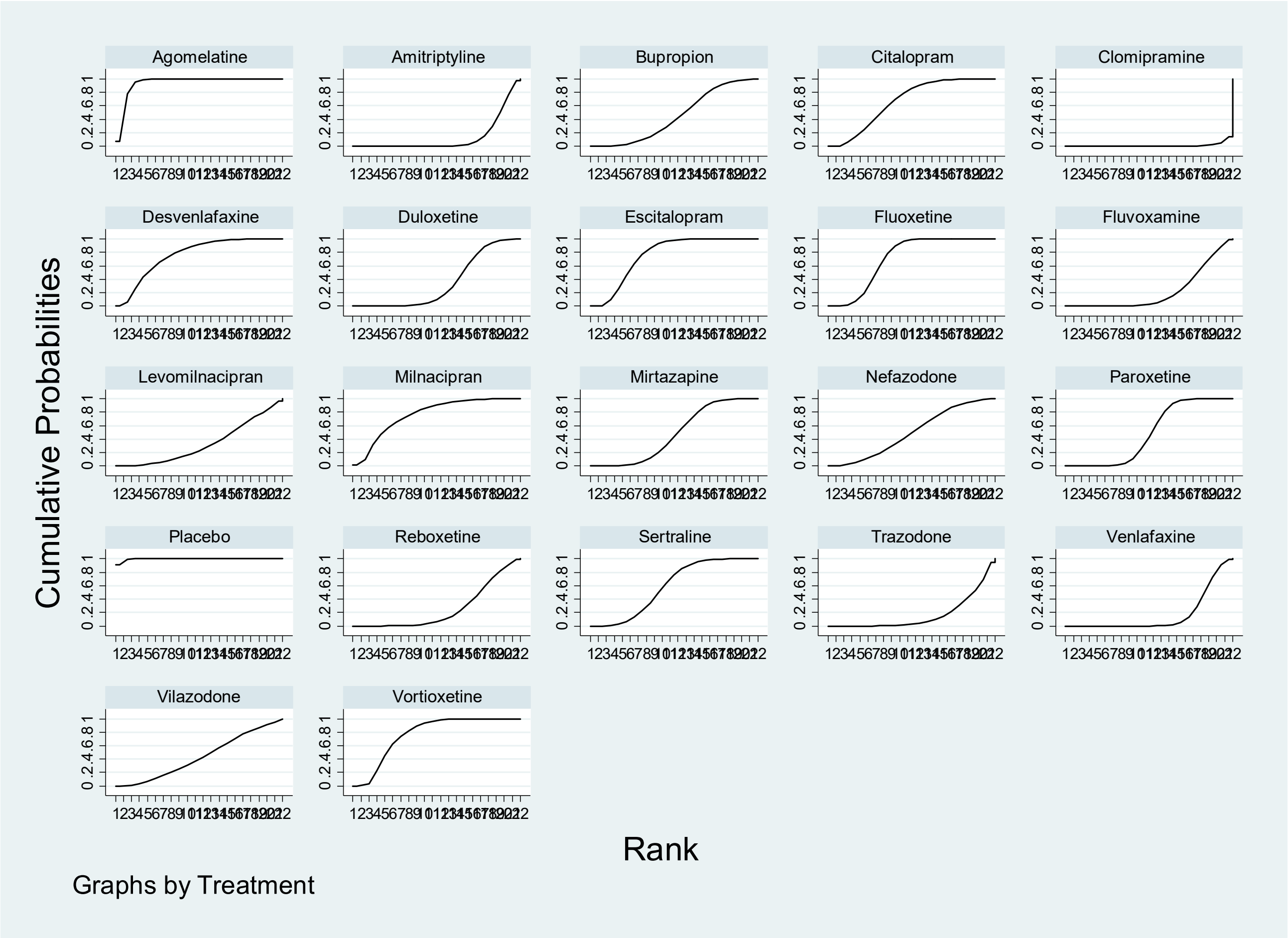

Treatment	SUCRA	PrBest	MeanRank
Placebo	99.6	91.0	1.1
Agomelatine	94.2	7.3	2.2
Amitriptyline	13.4	0.0	19.2
Bupropion	44.6	0.0	12.6
Citalopram	66.8	0.0	8.0
Clomipramine	1.2	0.0	21.8
Desvenlafaxine	76.1	0.4	6.0
Duloxetine	34.7	0.0	14.7
Escitalopram	75.9	0.0	6.1
Fluoxetine	70.8	0.0	7.1
Fluvoxamine	22.4	0.0	17.3
Levomilnacipran	33.3	0.0	15.0
Milnacipran	76.6	1.2	5.9
Mirtazapine	48.0	0.0	11.9
Nefazodone	49.8	0.0	11.5
Paroxetine	48.6	0.0	11.8
Reboxetine	25.8	0.0	16.6
Sertraline	59.0	0.0	9.6
Trazodone	16.6	0.0	18.5
Venlafaxine	17.3	0.0	18.4
Vilazodone	46.0	0.0	12.3
Vortioxetine	79.3	0.0	5.3

10.2 Head-to-head trials

194 studies (with at least two arms at licensed dose), 34 196 participants

10.2.1 Response

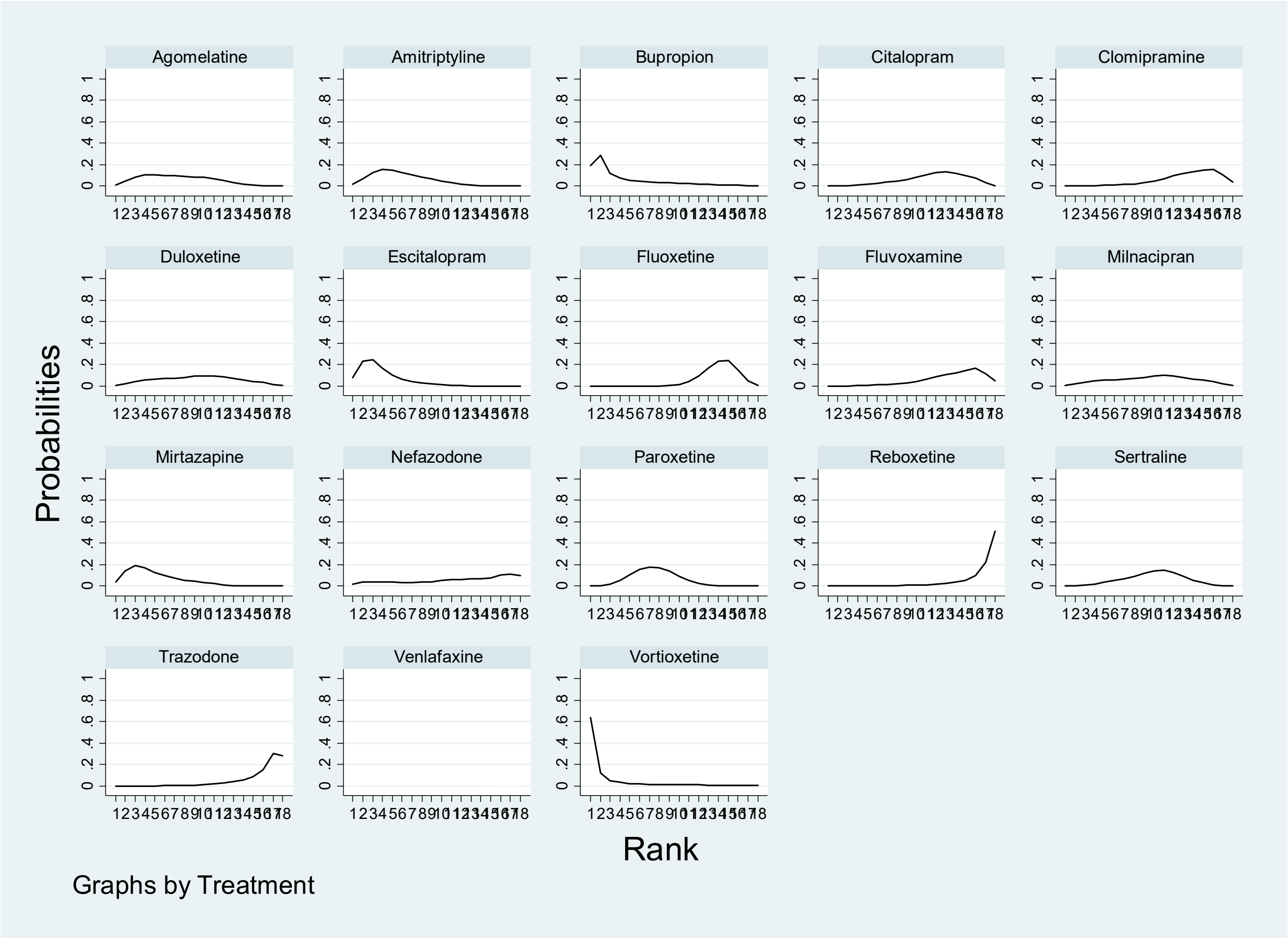

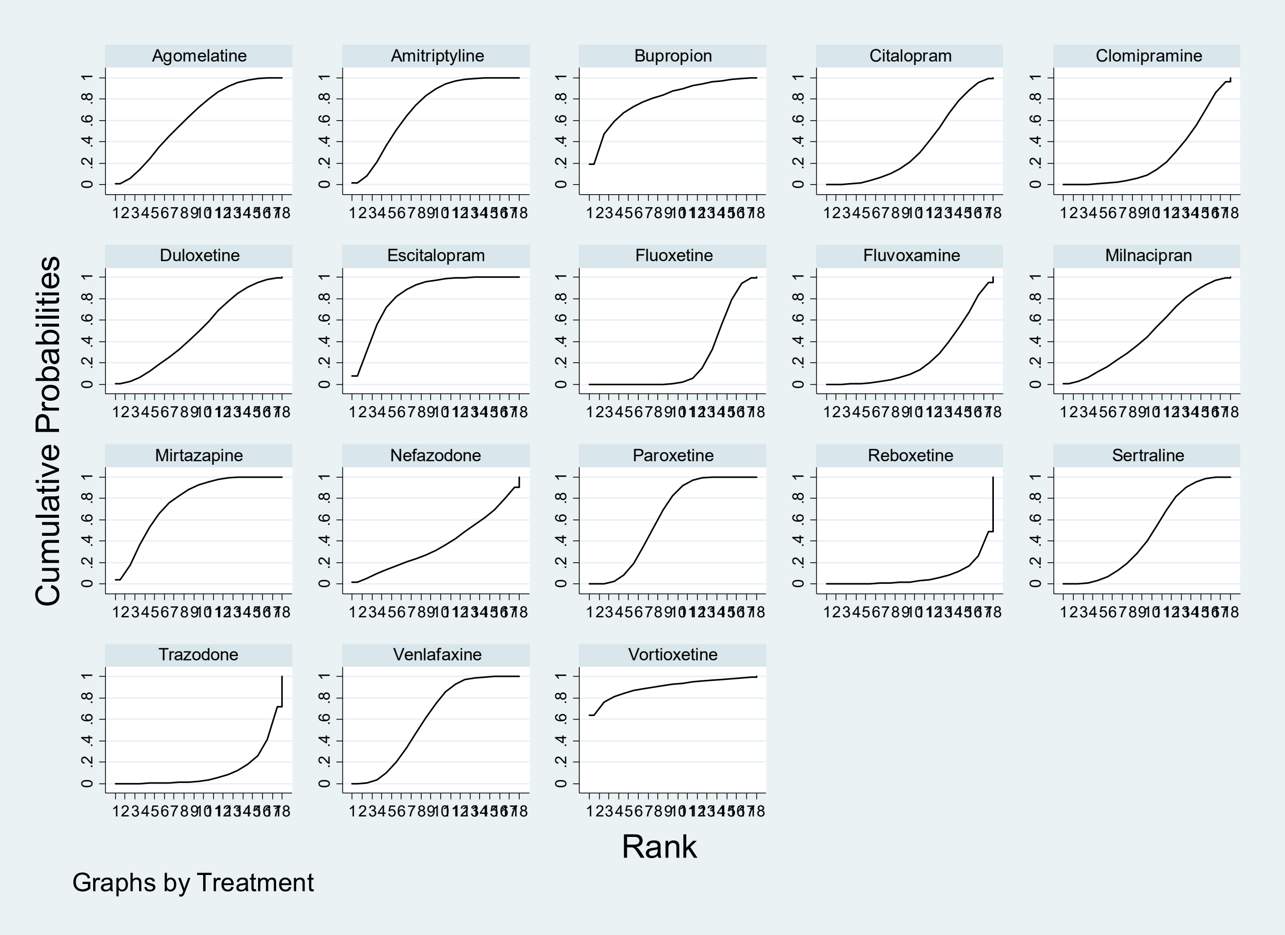

Treatment	SUCRA	PrBest	MeanRank
Agomelatine	62.9	1.2	7.3
Amitriptyline	71.7	1.5	5.8
Bupropion	80.3	19.5	4.4
Citalopram	36.1	0.0	11.9
Clomipramine	25.9	0.0	13.6
Duloxetine	50.7	0.5	9.4
Escitalopram	83.6	7.5	3.8
Fluoxetine	22.7	0.0	14.1
Fluvoxamine	25.1	0.0	13.7
Milnacipran	48.2	0.7	9.8
Mirtazapine	76.9	3.8	4.9
Nefazodone	37.3	1.6	11.7
Paroxetine	62.1	0.1	7.4
Reboxetine	7.7	0.0	16.7
Sertraline	47.1	0.0	10.0
Trazodone	11.3	0.0	16.1
Venlafaxine	60.3	0.0	7.7
Vortioxetine	90.1	63.6	2.7

10.2.2 Dropouts for any reason

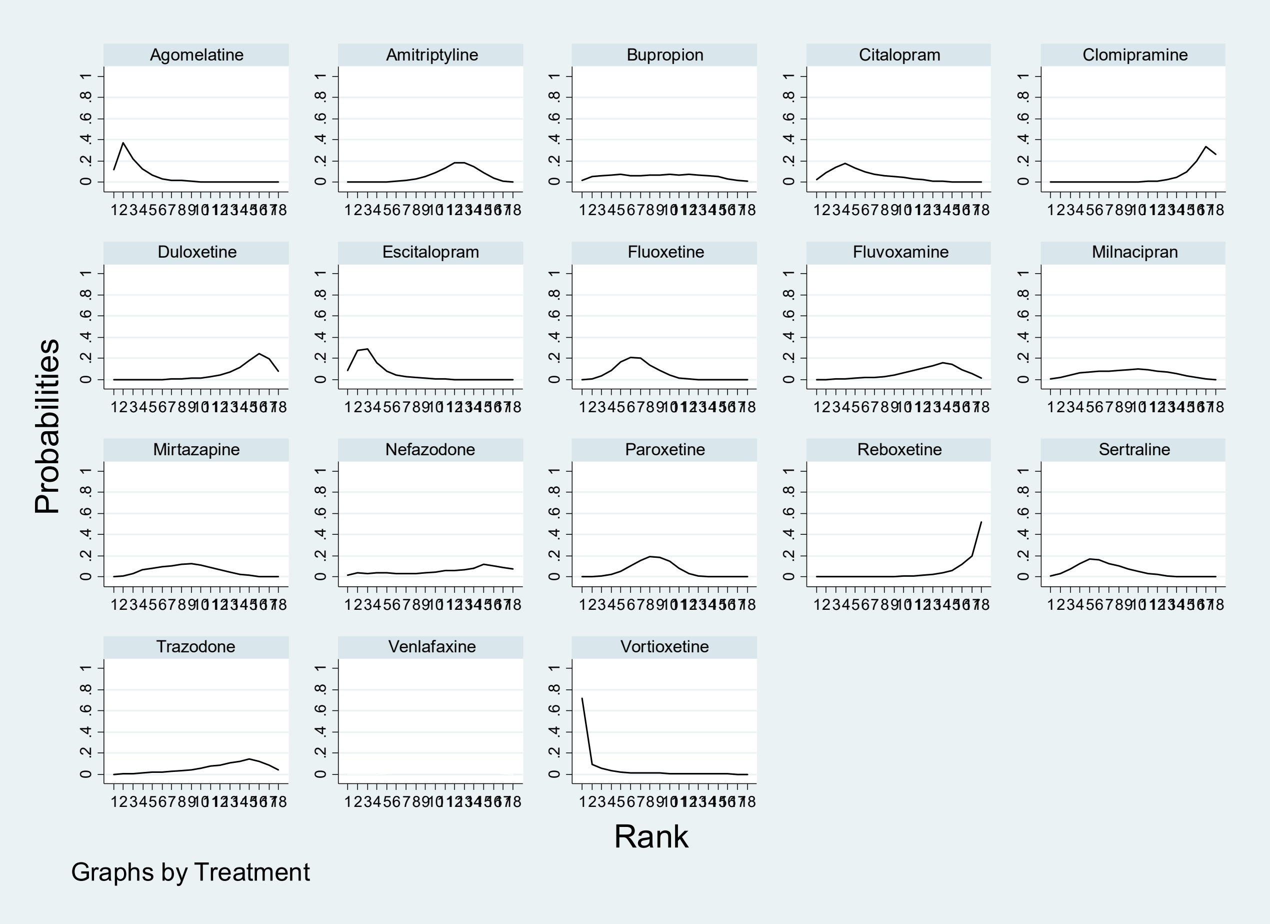

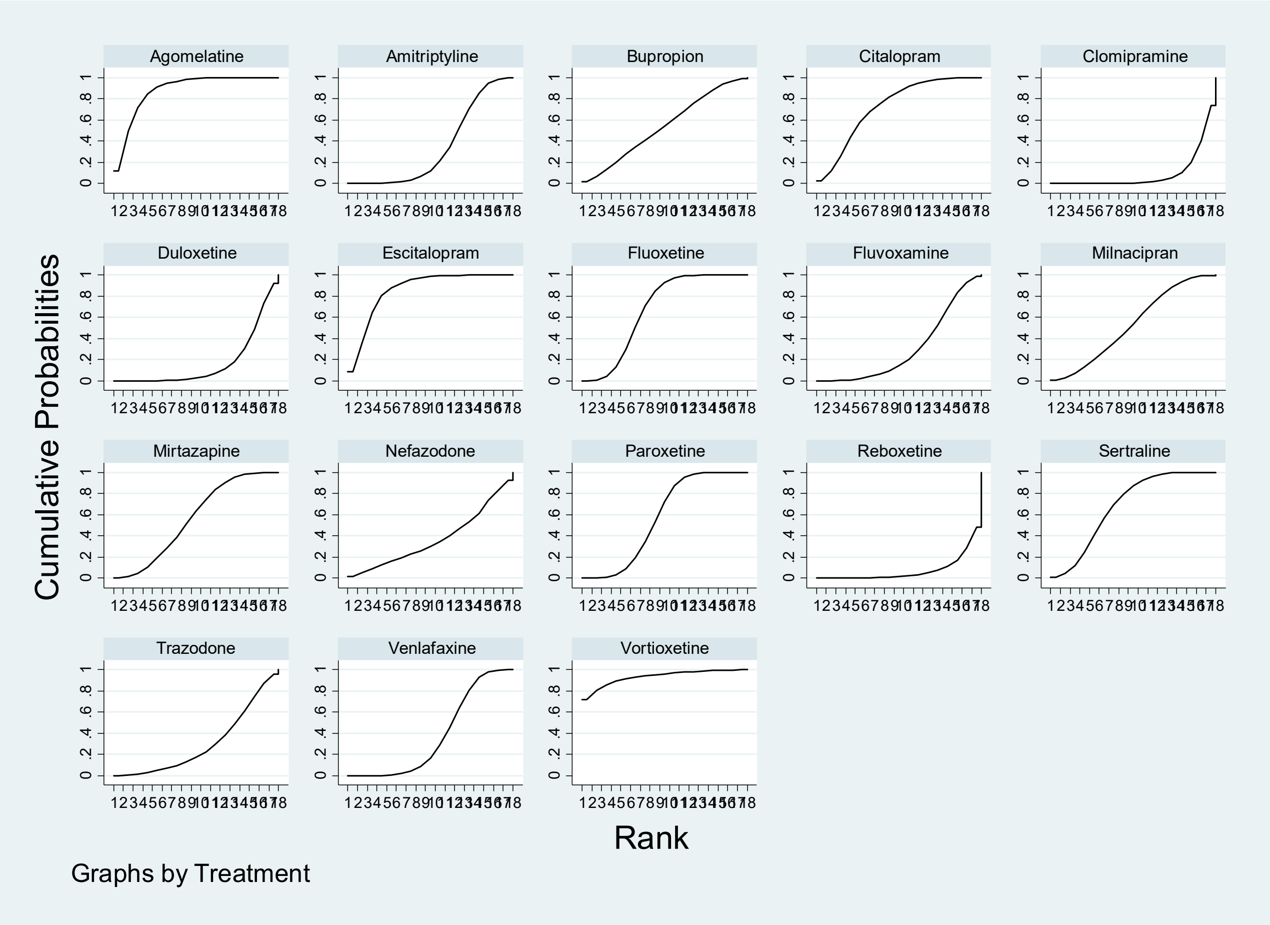

Treatment	SUCRA	PrBest	MeanRank
Agomelatine	88.1	12.3	3.0
Amitriptyline	34.3	0.0	12.2
Bupropion	53.7	1.6	8.9
Citalopram	72.7	2.5	5.6
Clomipramine	9.2	0.0	16.4
Duloxetine	17.1	0.0	15.1
Escitalopram	85.9	8.7	3.4
Fluoxetine	67.4	0.0	6.5
Fluvoxamine	30.8	0.0	12.8
Milnacipran	53.2	0.5	9.0
Mirtazapine	56.4	0.2	8.4
Nefazodone	36.8	1.4	11.7
Paroxetine	57.2	0.0	8.3
Reboxetine	7.5	0.0	16.7
Sertraline	68.4	1.1	6.4
Trazodone	30.3	0.1	12.9
Venlafaxine	37.8	0.0	11.6
Vortioxetine	93.3	71.5	2.1

10.2.3 Efficacy as continuous outcome

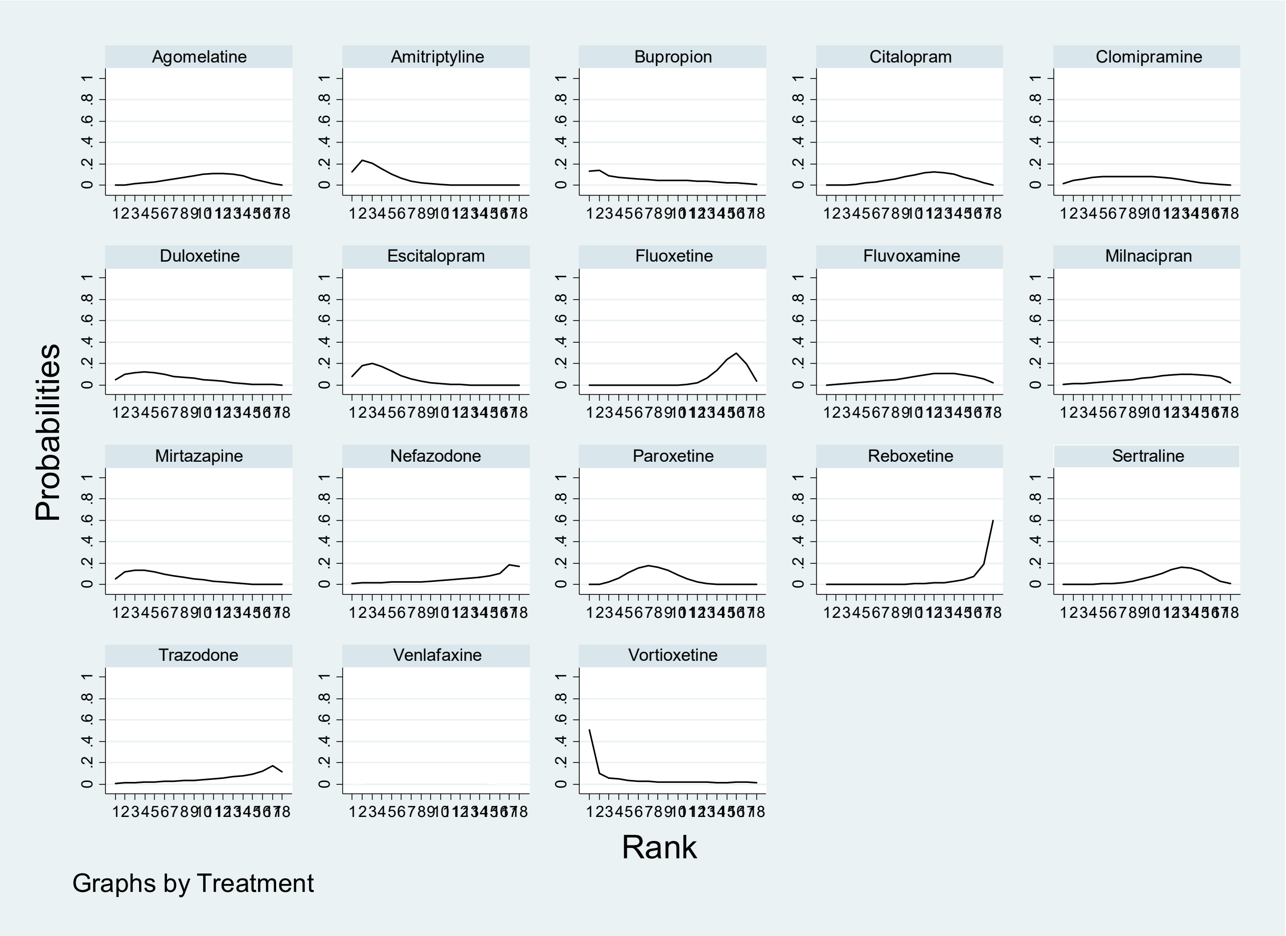

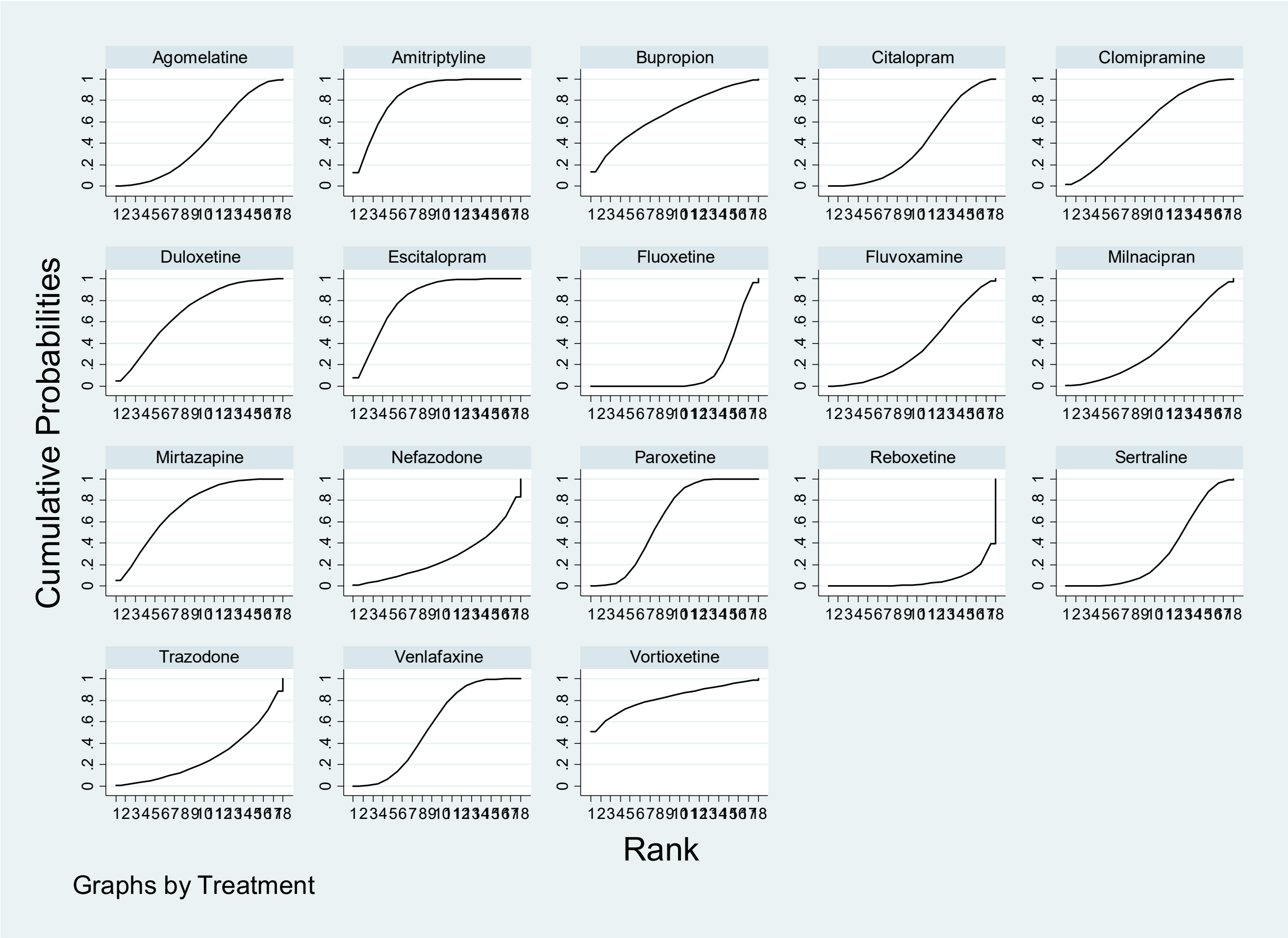

Treatment	SUCRA	PrBest	MeanRank
Agomelatine	43.3	0.2	10.6
Amitriptyline	84.8	12.9	3.6
Bupropion	67.5	13.4	6.5
Citalopram	39.3	0.0	11.3
Clomipramine	58.0	1.9	8.1
Duloxetine	69.7	4.7	6.1
Escitalopram	81.6	8.2	4.1
Fluoxetine	15.1	0.0	15.4
Fluvoxamine	36.6	0.1	11.8
Milnacipran	37.4	0.4	11.6
Mirtazapine	73.3	5.6	5.5
Nefazodone	27.2	1.1	13.4
Paroxetine	62.3	0.1	7.4
Reboxetine	5.9	0.0	17.0
Sertraline	32.0	0.0	12.6
Trazodone	27.8	0.6	13.3
Venlafaxine	56.2	0.1	8.4
Vortioxetine	82.1	50.7	4.0

10.2.4 Remission

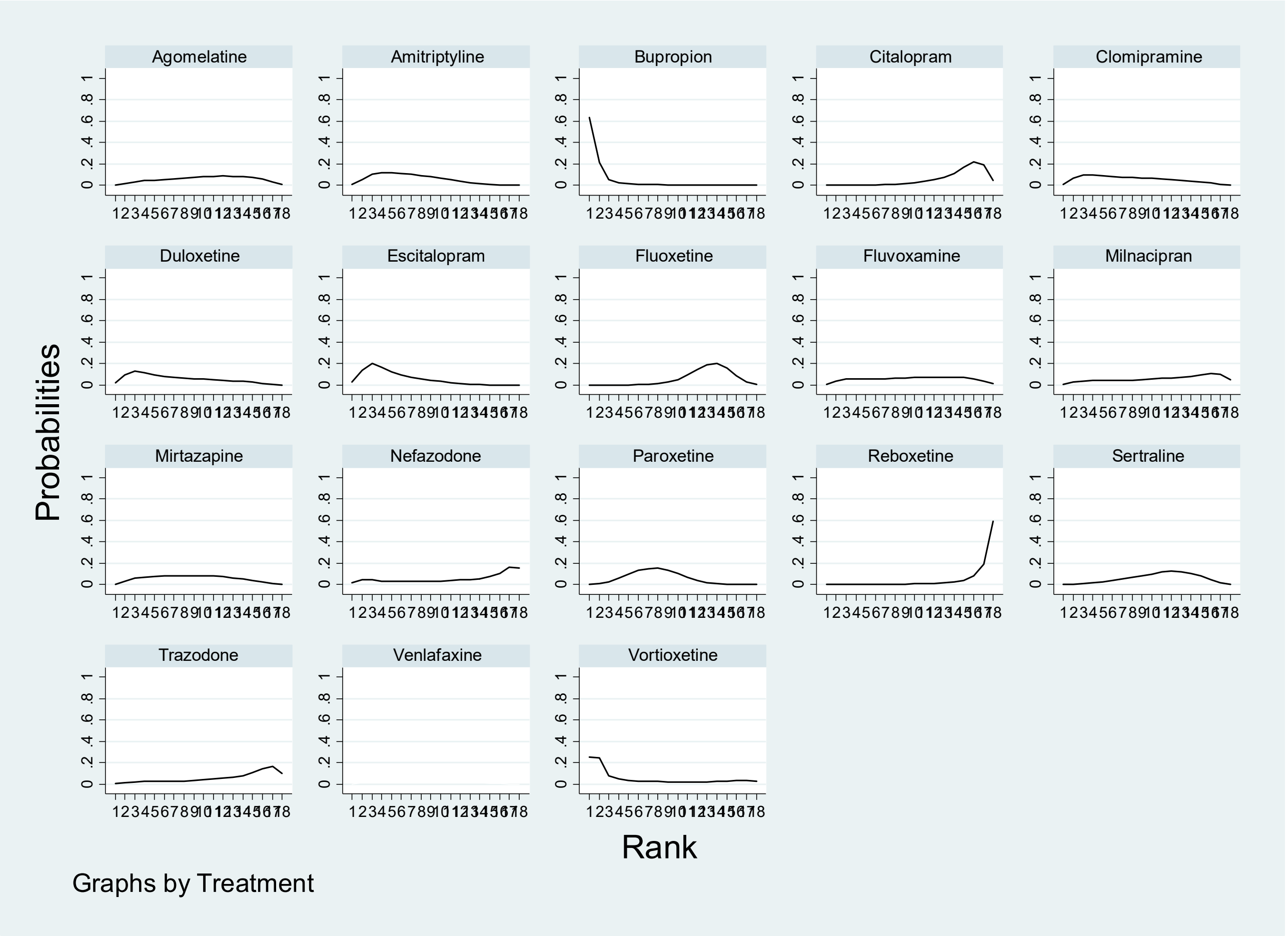

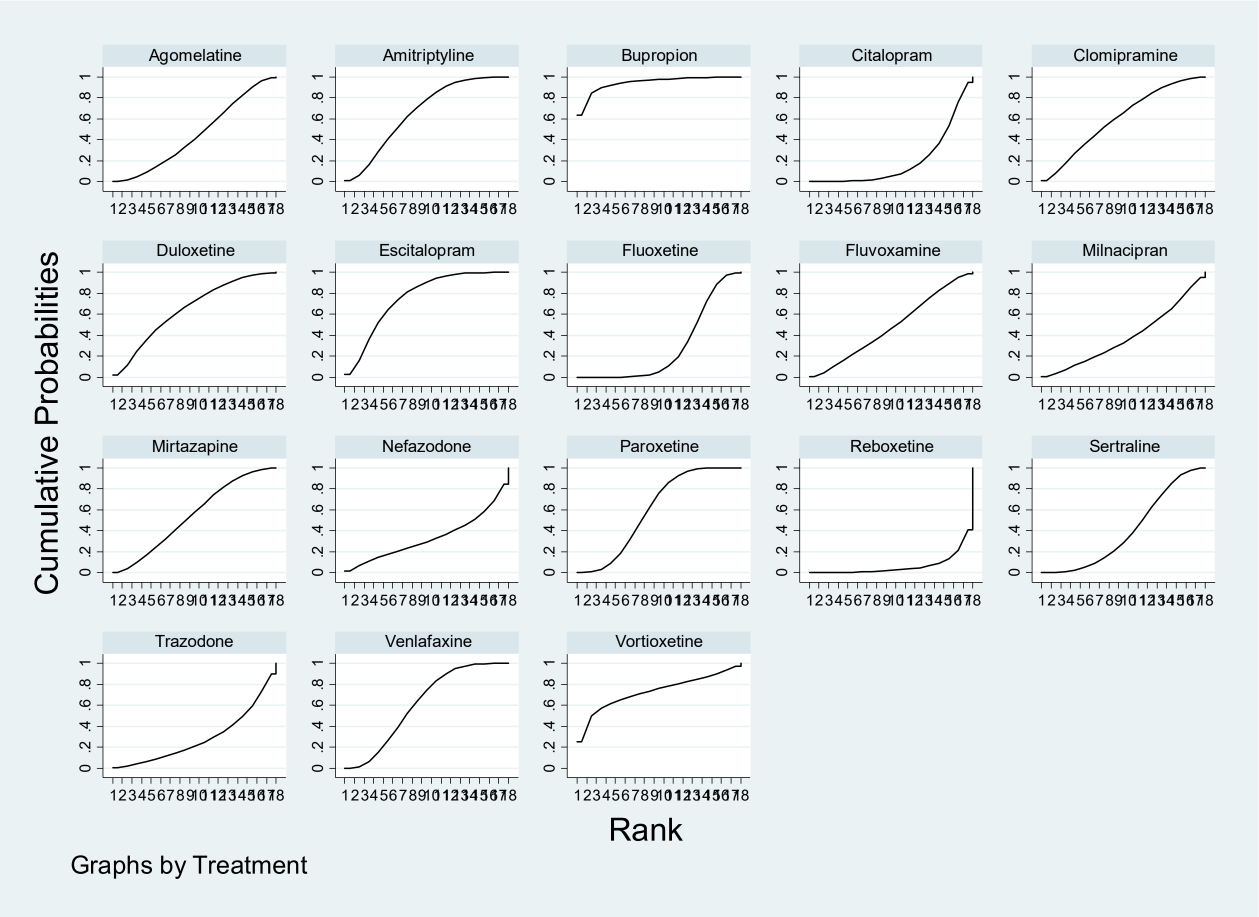

Treatment	SUCRA	PrBest	MeanRank
Agomelatine	45.0	0.2	10.4
Amitriptyline	66.1	0.8	6.8
Bupropion	94.4	63.5	2.0
Citalopram	19.8	0.0	14.6
Clomipramine	60.4	1.3	7.7
Duloxetine	64.9	2.1	7.0
Escitalopram	76.0	2.6	5.1
Fluoxetine	28.5	0.0	13.2
Fluvoxamine	48.3	0.7	9.8
Milnacipran	38.5	0.7	11.5
Mirtazapine	54.8	0.5	8.7
Nefazodone	33.6	1.9	12.3
Paroxetine	60.1	0.1	7.8
Reboxetine	6.4	0.0	16.9
Sertraline	40.2	0.0	11.2
Trazodone	28.6	0.2	13.1
Venlafaxine	61.5	0.1	7.6
Vortioxetine	73.1	25.4	5.6

10.2.5 Dropouts due to adverse eventss

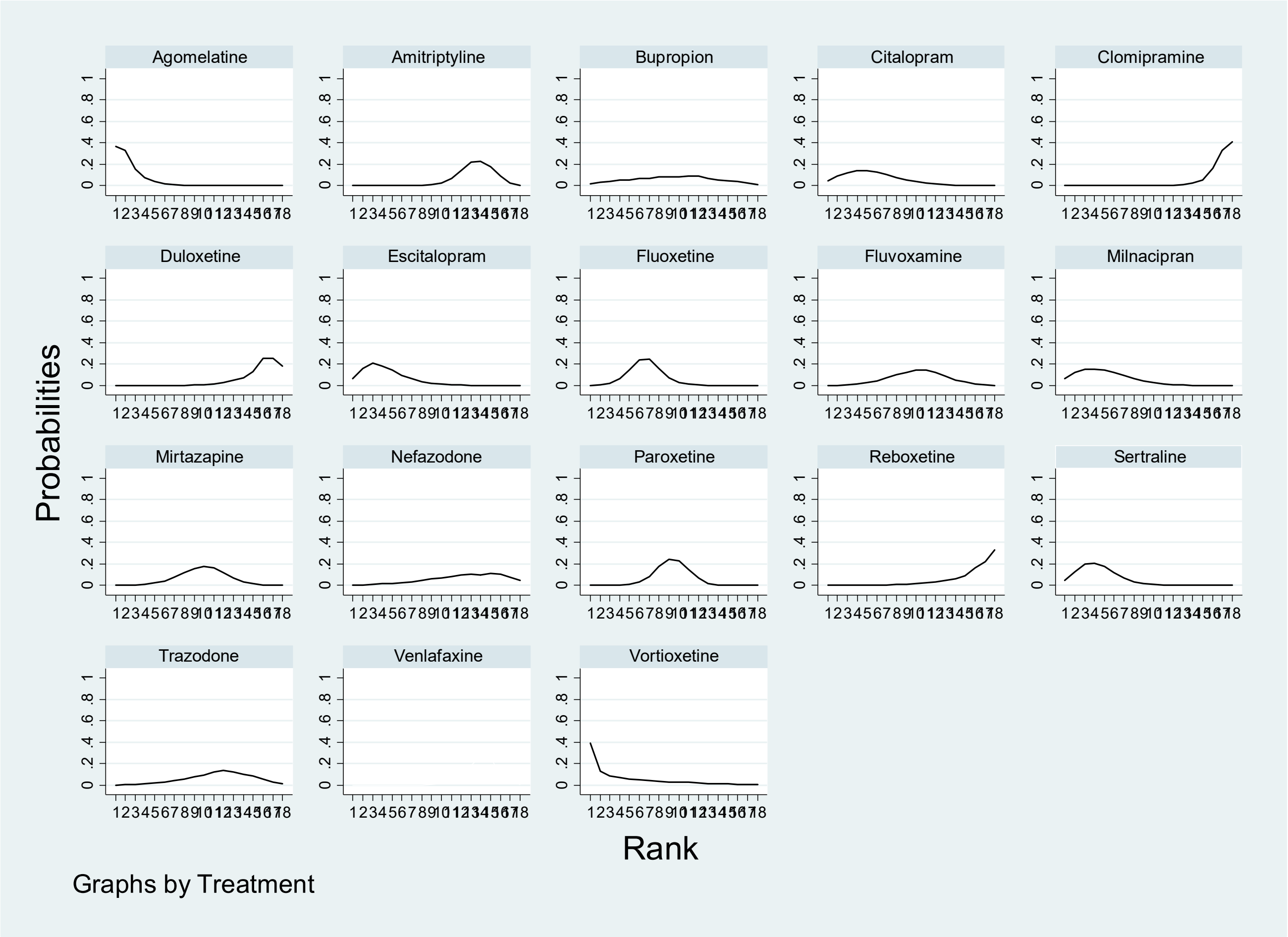

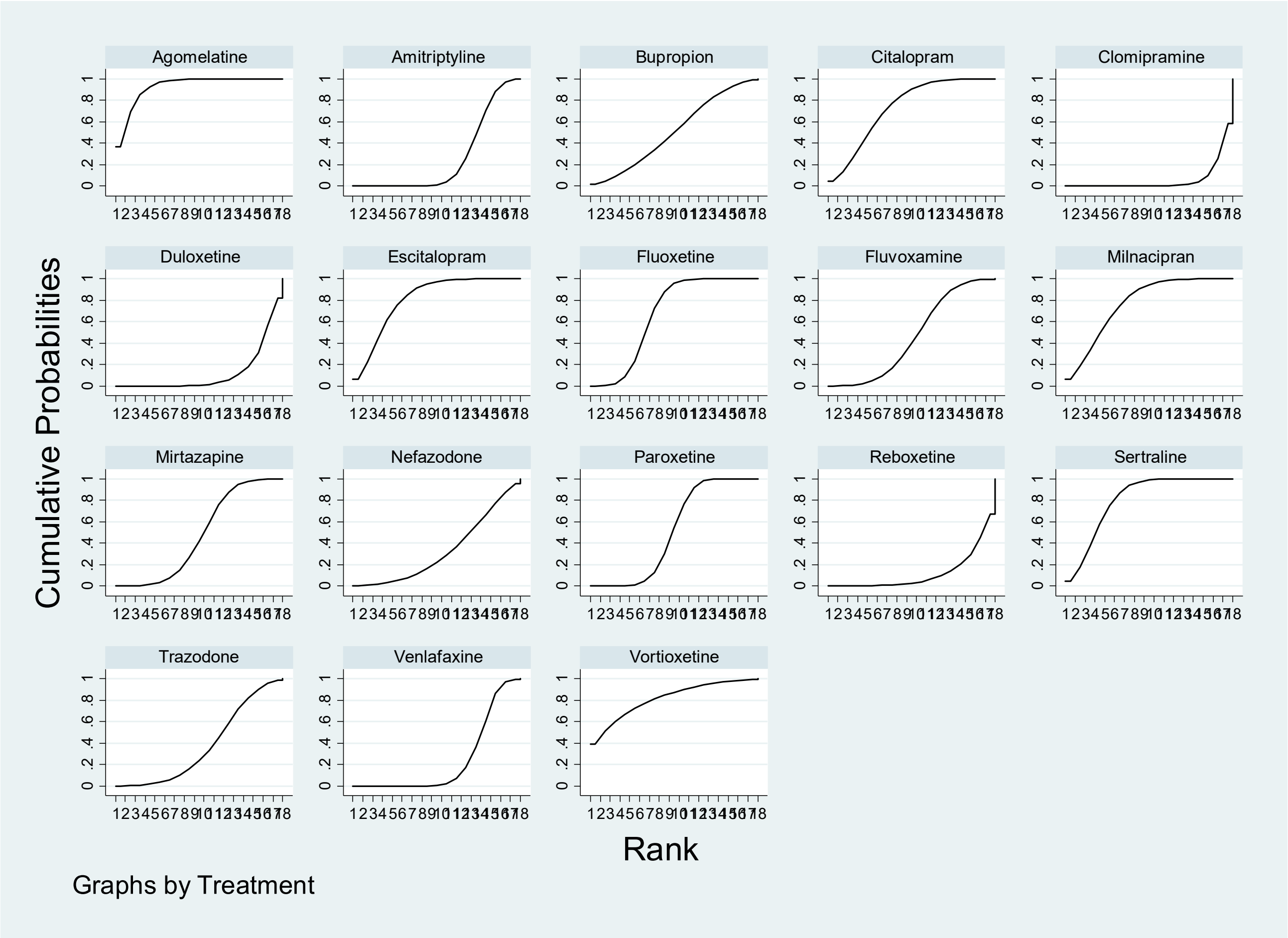

Treatment	SUCRA	PrBest	MeanRank
Agomelatine	92.9	36.9	2.2
Amitriptyline	26.2	0.0	13.5
Bupropion	50.9	1.8	9.4
Citalopram	73.4	4.5	5.5
Clomipramine	5.9	0.0	17.0
Duloxetine	12.4	0.0	15.9
Escitalopram	81.0	6.5	4.2
Fluoxetine	66.9	0.0	6.6
Fluvoxamine	46.2	0.0	10.1
Milnacipran	77.0	6.4	4.9
Mirtazapine	47.7	0.0	9.9
Nefazodone	33.0	0.2	12.4
Paroxetine	51.1	0.0	9.3
Reboxetine	11.9	0.0	16.0
Sertraline	80.5	4.7	4.3
Trazodone	37.5	0.1	11.6
Venlafaxine	23.9	0.0	13.9
Vortioxetine	81.6	38.8	4.1

11 Post hoc sensitivity analyses and meta-regression analyses (as requested by peer reviewers)

11.1 Meta-regression using the mean age of participants as covariate

	Response						Dropouts for any reason					
	OR drug vs PLA			beta of comparison			OR drug vs PLA			beta of comparison		
	2.5%	median	97.5%	2.5%	median	97.5%	2.5%	median	97.5%	2.5%	median	97.5%
Agomelatine	1.44	1.64	1.88	0.84	0.96	1.09	0.73	0.85	0.98	0.90	1.04	1.20
Amitriptyline	1.92	2.18	2.48	1.00	1.18	1.38	0.82	0.94	1.08	0.73	0.87	1.03
Bupropion	1.53	2.00	2.65	0.41	0.65	1.00	0.64	0.84	1.11	0.84	1.29	1.98
Citalopram	1.36	1.57	1.82	0.92	1.07	1.25	0.80	0.95	1.13	0.63	0.79	0.98
Clomipramine	1.03	1.36	1.78	0.86	1.06	1.30	1.00	1.37	1.85	0.81	1.05	1.36
Desvenlafaxine	1.22	1.48	1.80	0.62	0.99	1.56	0.82	1.03	1.30	0.80	1.35	2.29
Duloxetine	1.66	1.86	2.08	0.73	0.85	0.99	0.97	1.10	1.24	0.92	1.06	1.21
Escitalopram	1.47	1.65	1.84	0.97	1.09	1.23	0.81	0.92	1.04	0.82	0.95	1.09
Fluoxetine	1.40	1.53	1.66	1.04	1.14	1.25	0.81	0.89	0.98	0.81	0.90	0.99
Fluvoxamine	1.50	1.82	2.21	0.71	1.02	1.46	0.76	0.98	1.25	0.60	1.12	2.13
Levomilnacipran	1.24	1.69	2.30	0.09	0.55	3.45	0.74	1.04	1.46	0.46	2.99	19.38
Milnacipran	1.34	1.72	2.20	0.49	0.83	1.39	0.70	0.94	1.25	0.64	1.17	2.16
Mirtazapine	1.59	1.85	2.16	0.75	0.92	1.13	0.86	1.01	1.19	0.96	1.19	1.49
Nefazodone	1.15	1.84	2.97	0.34	0.93	2.55	0.45	0.76	1.28	0.46	1.36	3.99
Paroxetine	1.60	1.74	1.89	0.88	0.98	1.08	0.87	0.96	1.04	0.90	1.00	1.12
Reboxetine	1.22	1.59	2.08	0.30	0.62	1.28	0.79	1.09	1.51	0.52	1.19	2.75
Sertraline	1.46	1.64	1.85	0.94	1.07	1.22	0.84	0.96	1.09	0.81	0.92	1.06
Trazodone	1.21	1.47	1.80	1.03	1.26	1.54	0.96	1.19	1.48	0.67	0.88	1.15
Venlafaxine	1.56	1.73	1.91	0.90	1.03	1.19	0.92	1.04	1.16	0.87	1.05	1.26
Vilazodone	0.52	1.48	4.06	0.10	1.91	42.35	0.49	1.45	4.31	0.02	0.42	9.92
Vortioxetine	1.45	1.67	1.92	0.74	0.91	1.11	0.86	1.01	1.19	0.76	0.97	1.26

11.2 Meta-regression using the sex distribution (percentage of females in each study) as covariate

	Response						Dropouts for any reason					
	OR drug vs PLA			beta of comparison			OR drug vs PLA			beta of comparison		
	2.5%	median	97.5%	2.5%	median	97.5%	2.5%	median	97.5%	2.5%	median	97.5%
Agomelatine	1.10	1.47	1.95	0.55	0.80	1.14	0.60	0.85	1.21	0.75	3.88	260.34
Amitriptyline	1.47	1.89	2.43	0.77	0.97	1.23	0.72	0.93	1.20	0.87	3.05	36.23
Bupropion	0.84	1.27	1.91	0.80	1.06	1.40	0.87	1.37	2.15	0.61	0.16	3.49
Citalopram	1.28	1.65	2.12	0.42	0.63	0.92	0.69	0.93	1.25	-	-	-
Clomipramine	1.01	1.53	2.31	0.78	1.11	1.59	0.79	1.30	2.13	0.66	1.00	69.76
Desvenlafaxine	1.20	1.50	1.89	0.84	0.99	1.16	0.84	1.09	1.42	0.91	2.50	15.61

Duloxetine	1.74	2.20	2.80	0.77	1.17	1.80	0.79	1.01	1.29	0.45	0.03	2.00
Escitalopram	1.23	1.54	1.93	0.70	0.86	1.06	0.70	0.88	1.12	0.92	3.84	35.37
Fluoxetine	1.34	1.55	1.81	0.77	0.92	1.10	0.71	0.83	0.97	0.91	3.15	26.39
Fluvoxamine	0.93	1.46	2.29	0.56	0.90	1.48	0.69	1.14	1.86	0.49	0.09	8.36
Levomilnacipran	1.09	1.48	2.01	0.16	0.52	1.65	0.95	1.27	1.68	-	-	-
Milnacipran	0.58	1.16	2.32	-	-	-	0.49	1.01	2.13	-	-	-
Mirtazapine	1.23	1.60	2.08	0.76	1.09	1.56	0.94	1.25	1.64	0.45	0.02	0.93
Nefazodone	1.42	1.86	2.45	0.61	0.87	1.26	0.63	0.84	1.12	0.64	0.74	44.61
Paroxetine	1.57	1.81	2.10	0.99	1.12	1.26	0.76	0.88	1.01	0.82	0.44	1.38
Reboxetine	1.21	1.48	1.82	0.94	1.24	1.65	0.86	1.07	1.32	0.62	0.18	3.13
Sertraline	1.17	1.46	1.82	0.92	1.23	1.64	0.74	0.94	1.19	0.78	1.68	34.88
Trazodone	1.24	1.62	2.14	-	-	-	0.89	1.20	1.60	-	-	-
Venlafaxine	1.54	1.79	2.07	0.89	1.07	1.29	0.92	1.08	1.26	0.85	1.23	8.04
Vilazodone	0.19	0.97	4.70	-	-	-	0.57	2.58	12.01	-	-	-
Vortioxetine	1.47	1.73	2.06	0.84	1.09	1.42	0.77	0.94	1.14	0.50	0.02	0.41

11.3 Sensitivity analysis excluding studies including bipolar patients

	Response						Dropouts for any reason					
	OR drug vs PLA			beta of comparison			OR drug vs PLA			beta of comparison		
	2.5%	median	97.5%	2.5%	median	97.5%	2.5%	median	97.5%	2.5%	median	97.5%
Agomelatine	1.42	1.62	1.86				0.72	0.83	0.97			
Amitriptyline	1.88	2.14	2.44				0.83	0.95	1.10			
Bupropion	1.35	1.58	1.86				0.80	0.95	1.12			
Citalopram	1.32	1.52	1.75				0.81	0.97	1.15			
Clomipramine	1.18	1.49	1.88				0.98	1.27	1.66			
Desvenlafaxine	1.24	1.49	1.79				0.88	1.08	1.33			
Duloxetine	1.65	1.85	2.07				0.96	1.09	1.23			
Escitalopram	1.50	1.67	1.87				0.80	0.91	1.02			
Fluoxetine	1.39	1.51	1.65				0.80	0.88	0.96			
Fluvoxamine	1.41	1.71	2.07				0.92	1.13	1.38			
Levomilnacipran	1.24	1.59	2.05				0.93	1.19	1.53			
Milnacipran	1.21	1.62	2.17				0.68	0.95	1.32			
Mirtazapine	1.63	1.89	2.20				0.85	0.99	1.15			
Nefazodone	1.35	1.86	2.57				0.54	0.75	1.05			
Paroxetine	1.61	1.74	1.90				0.87	0.95	1.03			
Reboxetine	1.06	1.28	1.54				1.00	1.22	1.48			
Sertraline	1.44	1.65	1.90				0.84	0.98	1.14			
Trazodone	1.21	1.49	1.84				0.87	1.09	1.37			
Venlafaxine	1.61	1.78	1.97				0.92	1.02	1.14			
Vilazodone	1.27	1.60	2.01				0.89	1.15	1.48			

Vortioxetine	1.44	1.66	1.92		0.86	1.01	1.19

11.4 Sensitivity analysis for study duration (8 weeks or less versus more than 8 weeks)

	Response							Dropouts for any reason						
	<=8			>8				<=8			>8			
	2.5%	median	97.5%	2.5%	median	97.5%		2.5%	median	97.5%	2.5%	median	97.5%	
Agomelatine	1.41	1.73	2.13	1.26	1.49	1.76		0.68	0.85	1.06	0.65	0.81	1.01	
Amitriptyline	1.90	2.16	2.46	1.30	2.14	3.57		0.84	0.96	1.10	0.46	0.74	1.21	
Bupropion	1.32	1.57	1.87	1.16	1.95	3.28		0.80	0.96	1.15	0.55	0.96	1.71	
Citalopram	1.30	1.51	1.76	1.03	1.47	2.11		0.80	0.96	1.15	0.64	1.00	1.58	
Clomipramine	1.15	1.48	1.90	0.97	1.41	2.06		0.93	1.25	1.69	0.90	1.56	2.73	
Desvenlafaxine	1.18	1.45	1.79	1.13	1.86	3.03		0.92	1.15	1.44	0.38	0.71	1.34	
Duloxetine	1.67	1.90	2.16	1.31	1.69	2.17		1.01	1.15	1.31	0.50	0.72	1.02	
Escitalopram	1.52	1.71	1.94	1.02	1.37	1.82		0.79	0.90	1.02	0.72	1.04	1.51	
Fluoxetine	1.37	1.51	1.67	1.32	1.54	1.77		0.81	0.90	1.00	0.68	0.82	0.98	
Fluvoxamine	1.42	1.70	2.05	-	-	-		0.92	1.11	1.34	-	-	-	
Levomilnacipran	1.11	1.49	2.02	1.27	1.99	3.12		1.01	1.33	1.76	0.42	0.76	1.37	
Milnacipran	1.29	1.80	2.52	1.13	1.56	2.19		0.68	0.97	1.40	0.60	0.95	1.48	
Mirtazapine	1.66	1.94	2.27	0.61	1.20	2.29		0.85	0.99	1.17	0.49	1.04	2.16	
Nefazodone	1.36	1.74	2.23	0.09	0.44	1.77		0.70	0.91	1.17	0.37	1.98	13.40	
Paroxetine	1.66	1.83	2.01	1.30	1.50	1.74		0.85	0.94	1.04	0.85	1.01	1.20	
Reboxetine	1.21	1.45	1.75	0.46	0.85	1.58		0.93	1.14	1.38	0.72	1.62	3.70	
Sertraline	1.48	1.68	1.91	1.16	1.61	2.24		0.82	0.94	1.07	0.77	1.13	1.66	
Trazodone	1.25	1.52	1.86	-	-	-		0.93	1.15	1.43	-	-	-	
Venlafaxine	1.62	1.83	2.06	1.41	1.68	2.00		0.92	1.05	1.19	0.81	1.01	1.26	
Vilazodone	1.18	1.55	2.03	1.13	1.72	2.62		0.80	1.09	1.48	0.77	1.32	2.24	
Vortioxetine	1.45	1.68	1.95	0.86	1.43	2.44		0.88	1.03	1.21	0.55	0.92	1.53	

11.5 Sensitivity analysis for country (North America only vs other)

	Response							Dropouts for any reason						
	North America			Other				North America			Other			
	2.5%	median	97.5%	2.5%	median	97.5%		2.5%	median	97.5%	2.5%	median	97.5%	
Agomelatine	-	-	-	1.23	1.65	2.23		-	-	-	0.53	0.73	1.01	
Amitriptyline	1.94	2.32	2.77	1.49	1.83	2.25		0.78	0.95	1.15	0.71	0.89	1.12	
Bupropion	1.41	1.65	1.95	0.46	0.87	1.66		0.81	0.97	1.16	-	-	-	
Citalopram	1.29	1.59	1.96	1.11	1.41	1.78		0.84	1.10	1.43	0.59	0.79	1.04	
Clomipramine	-	-	-	0.98	1.30	1.73		-	-	-	0.79	1.13	1.61	

Desvenlafaxine	1.16	1.42	1.73	-	-	-		0.84	1.08	1.39	-	-	-	
Duloxetine	1.48	1.72	2.00	1.40	1.93	2.67		0.97	1.16	1.39	0.58	0.85	1.24	
Escitalopram	1.36	1.58	1.83	1.35	1.68	2.10		0.85	1.02	1.22	0.52	0.67	0.87	
Fluoxetine	1.45	1.62	1.82	1.11	1.32	1.58		0.73	0.83	0.95	0.73	0.89	1.08	
Fluvoxamine	1.65	2.27	3.16	0.93	1.23	1.63		0.81	1.10	1.50	0.74	1.02	1.40	
Levomilnacipran	1.14	1.50	1.96	-	-	-		0.98	1.33	1.80	-	-	-	
Milnacipran	-	-	-	1.13	1.52	2.05		-	-	-	0.65	0.89	1.24	
Mirtazapine	1.57	1.97	2.47	1.34	1.71	2.19		0.74	0.95	1.22	0.73	0.95	1.24	
Nefazodone	1.38	1.79	2.33	0.67	1.19	2.13		0.65	0.87	1.16	0.62	1.19	2.31	
Paroxetine	1.47	1.65	1.85	1.39	1.64	1.94		0.94	1.07	1.21	0.70	0.85	1.02	
Reboxetine	0.92	1.19	1.53	0.68	1.03	1.56		1.00	1.34	1.80	0.83	1.31	2.06	
Sertraline	1.48	1.72	2.01	1.18	1.47	1.83		0.85	1.01	1.20	0.68	0.88	1.14	
Trazodone	1.20	1.56	2.02	1.00	1.37	1.88		0.87	1.17	1.56	0.72	1.04	1.50	
Venlafaxine	1.46	1.70	1.99	1.41	1.72	2.09		0.84	1.02	1.24	0.79	0.98	1.21	
Vilazodone	1.30	1.62	2.01	-	-	-		0.88	1.17	1.55	-	-	-	
Vortioxetine	0.95	1.20	1.51	1.18	1.88	3.01		0.83	1.11	1.48	0.37	0.63	1.10	

12 *Changes to the protocol*

Of the seven pre-planned network meta-regressions, we did not conduct one by ‘response to placebo’ because this was logically inappropriate to conduct. That is, the two primary outcomes of interest, OR for response already contains this information and it would not make sense to regress OR for response (i.e. response to drug over response to placebo) on response to placebo.
