# Supplementary material for: Effects of different training on lower limb explosive power in youth soccer players: a systematic review and network meta-analysis
Source: Front Physiol. 2026 Mar 19;17:1769079. doi: 10.3389/fphys.2026.1769079 (PMC13043373; doi:10.3389/fphys.2026.1769079)
Supplement: Supplementary file 2 [file Presentation1.zip › 附件/Risk of bias and GRADE assessment/A6.docx]

|  | A | B | C | D | E | F |
| --- | --- | --- | --- | --- | --- | --- |
| Best | 0 | 27.3 | 2.5 | 2.2 | 2.3 | 65.7 |
| 2nd | 0 | 40.8 | 13.3 | 1.5 | 15.9 | 28.5 |
| 3rd | 0 | 21.4 | 25.3 | 2 | 46 | 5.3 |
| 4th | 3.1 | 9.6 | 50.6 | 3.3 | 32.9 | 0.5 |
| 5th | 80.1 | 0.8 | 7.6 | 8.5 | 2.9 | 0 |
| Worst | 16.8 | 0 | 0.7 | 82.4 | 0 | 0 |
