# Supplementary material for: Effects of different training on lower limb explosive power in youth soccer players: a systematic review and network meta-analysis
Source: Front Physiol. 2026 Mar 19;17:1769079. doi: 10.3389/fphys.2026.1769079 (PMC13043373; doi:10.3389/fphys.2026.1769079)
Supplement: Supplementary file 2 [file Presentation1.zip › 附件/Risk of bias and GRADE assessment/A7.docx]

| Side | Direct |  | Indirect |  | Difference |  |  |
| --- | --- | --- | --- | --- | --- | --- | --- |
|  | Coef. | Std. Err. | Coef. | Std. Err. | Coef. | Std. Err. | P>z |
| A B | 2.797158 | 0.998808 | 2.52199 | 1.986267 | 0.2751688 | 2.215979 | 0.901 |
| A C | 1.683148 | 1.098524 | 1.524632 | 1.633557 | 0.1585152 | 1.967246 | 0.936 |
| A D | . | . | . | . | . | . | . |
| A E | 2.426929 | 0.8375178 | 0.7121947 | 1.350166 | 1.714734 | 1.561834 | 0.272 |
| A F | 3.380992 | 0.703642 | 3.16751 | 1.339445 | 0.2134823 | 1.511566 | 0.888 |
| B E | -1.712815 | 1.422661 | 0.1429704 | 1.410799 | -1.855786 | 2.008551 | 0.356 |
| C F | 1.791806 | 1.494013 | 1.634171 | 1.280336 | 0.1576348 | 1.967685 | 0.936 |
| E F | 1.487605 | 1.355051 | 1.283945 | 1.134905 | 0.2036604 | 1.761125 | 0.908 |
