# Supplementary material for: Effects of different training on lower limb explosive power in youth soccer players: a systematic review and network meta-analysis
Source: Front Physiol. 2026 Mar 19;17:1769079. doi: 10.3389/fphys.2026.1769079 (PMC13043373; doi:10.3389/fphys.2026.1769079)
Supplement: Supplementary file 2 [file Presentation1.zip › 附件/Tables/Table 2.docx]

|  | **Study  Limitation** | **Imprecision** | **Inconsistency** | **Indirectness** | **Publication bias** | **Grade** |
| --- | --- | --- | --- | --- | --- | --- |
| **CON vs OPL** | downgrade | downgrade | No downgrade | No downgrade | No downgrade | low |
| **CON vs RT** | No downgrade | downgrade | No downgrade | No downgrade | No downgrade | Moderate |
| **CON vs HIIT** | No downgrade | No downgrade | downgrade | No downgrade | No downgrade | Moderate |
| **CON vs JT** | No downgrade | No downgrade | No downgrade | downgrade | No downgrade | Moderate |
| **CON vs CT** | No downgrade | No downgrade | No downgrade | downgrade | No downgrade | Moderate |
| **OPL vs JT** | No downgrad | No downgrade | No downgrade | downgrade | No downgrade | Moderate |
| **RT vs CT** | No downgrade | No downgrade | No downgrade | downgrade | No downgrade | Moderate |
| **JT vs CT** | No downgrade | downgrade | No downgrade | No downgrade | No downgrade | Moderate |
| **OPL vs RT** | downgrade | downgrade | downgrade | downgrade | No downgrade | very low |
| **OPL vs HIIT** | downgrade | downgrade | downgrade | downgrade | No downgrade | very low |
| **OPL vs CT** | downgrade | downgrade | downgrade | downgrade | No downgrade | very low |
| **RT vs HIIT** | downgrade | downgrade | downgrade | downgrade | No downgrade | very low |
| **RT vs JT** | downgrade | downgrade | downgrade | downgrade | No downgrade | very low |
| **HIIT vs JT** | downgrade | downgrade | downgrade | downgrade | No downgrade | very low |
| **HIIT vs CT** | downgrade | downgrade | downgrade | downgrade | No downgrade | very low |
