# Supplementary figures and images for: Effects of different training on lower limb explosive power in youth soccer players: a systematic review and network meta-analysis
Source: Front Physiol. 2026 Mar 19;17:1769079. doi: 10.3389/fphys.2026.1769079 (PMC13043373; doi:10.3389/fphys.2026.1769079)

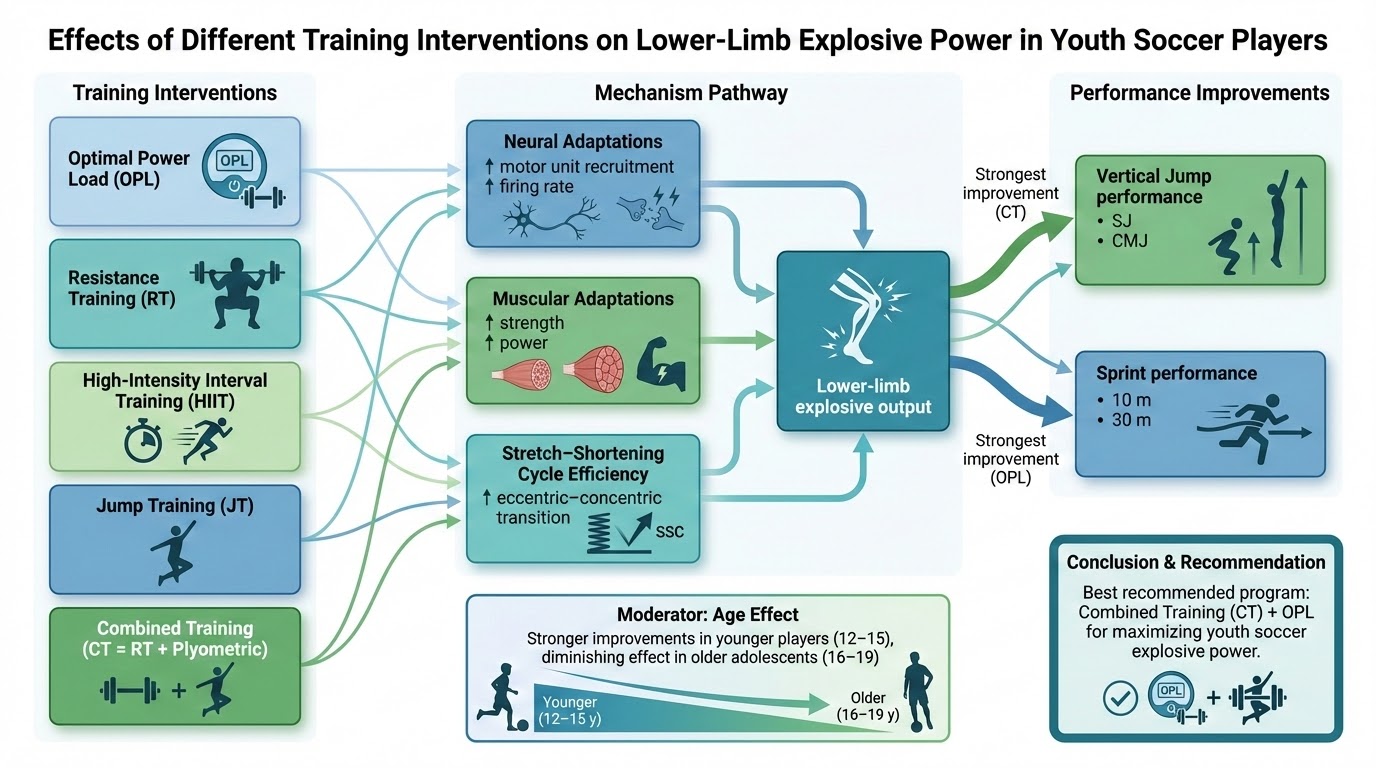

Supplement: Supplementary file 2 [file Presentation1.zip › 附件/Figs/Fig 4 .jpg]

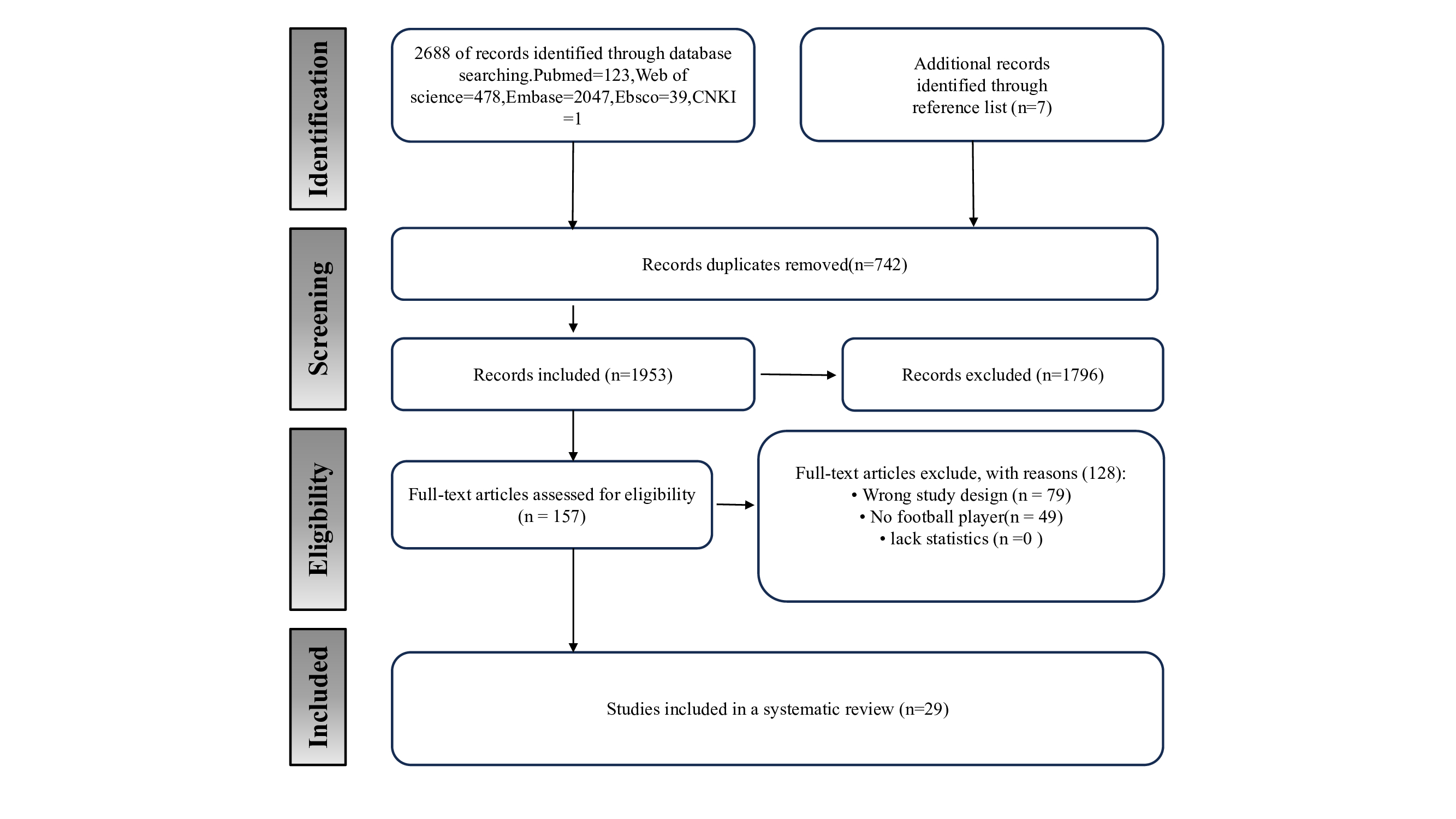

Supplement: Supplementary file 2 [file Presentation1.zip › 附件/Figs/Fig-1.jpeg]

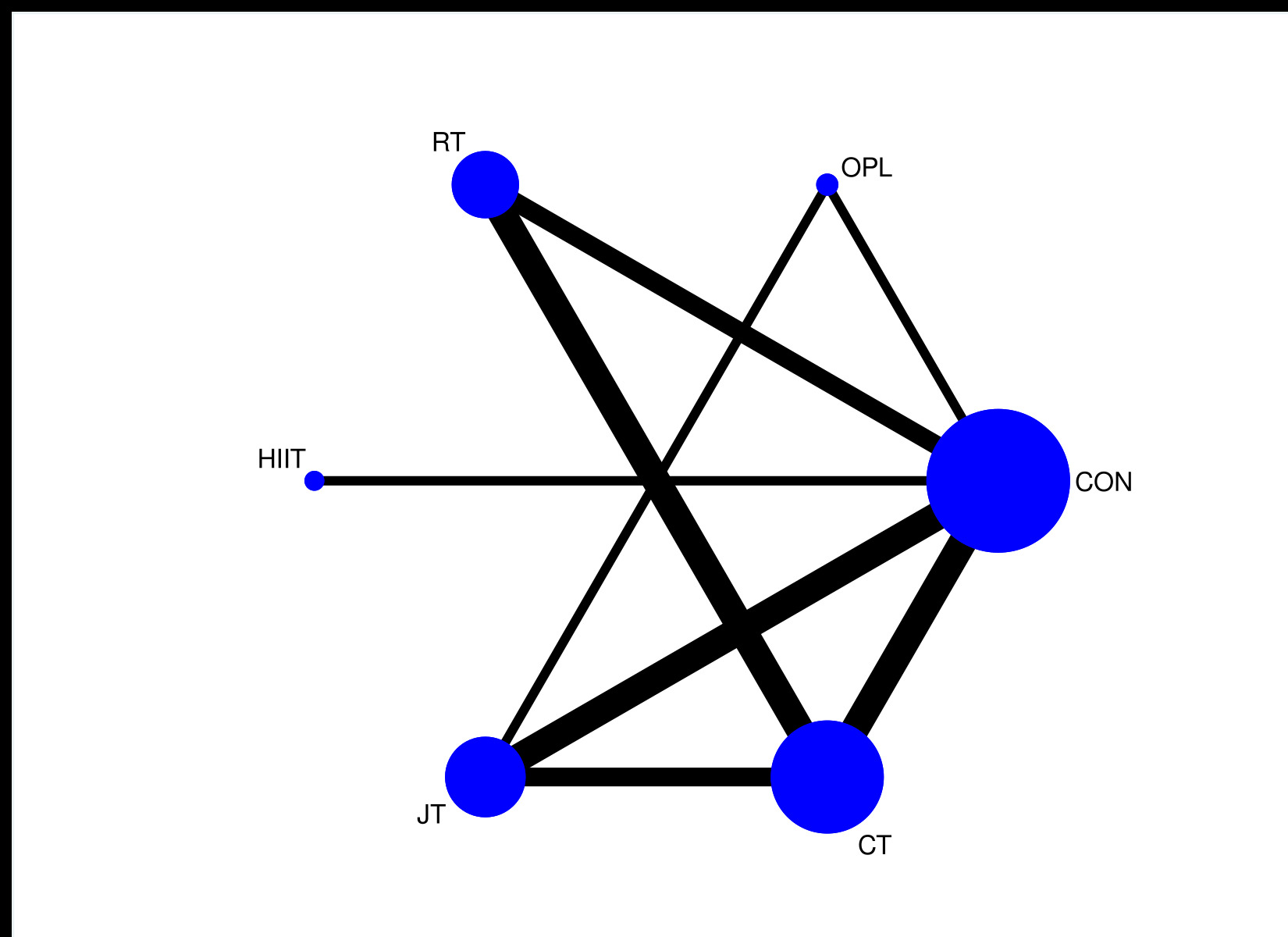

Supplement: Supplementary file 2 [file Presentation1.zip › 附件/Figs/Fig-13.jpeg]

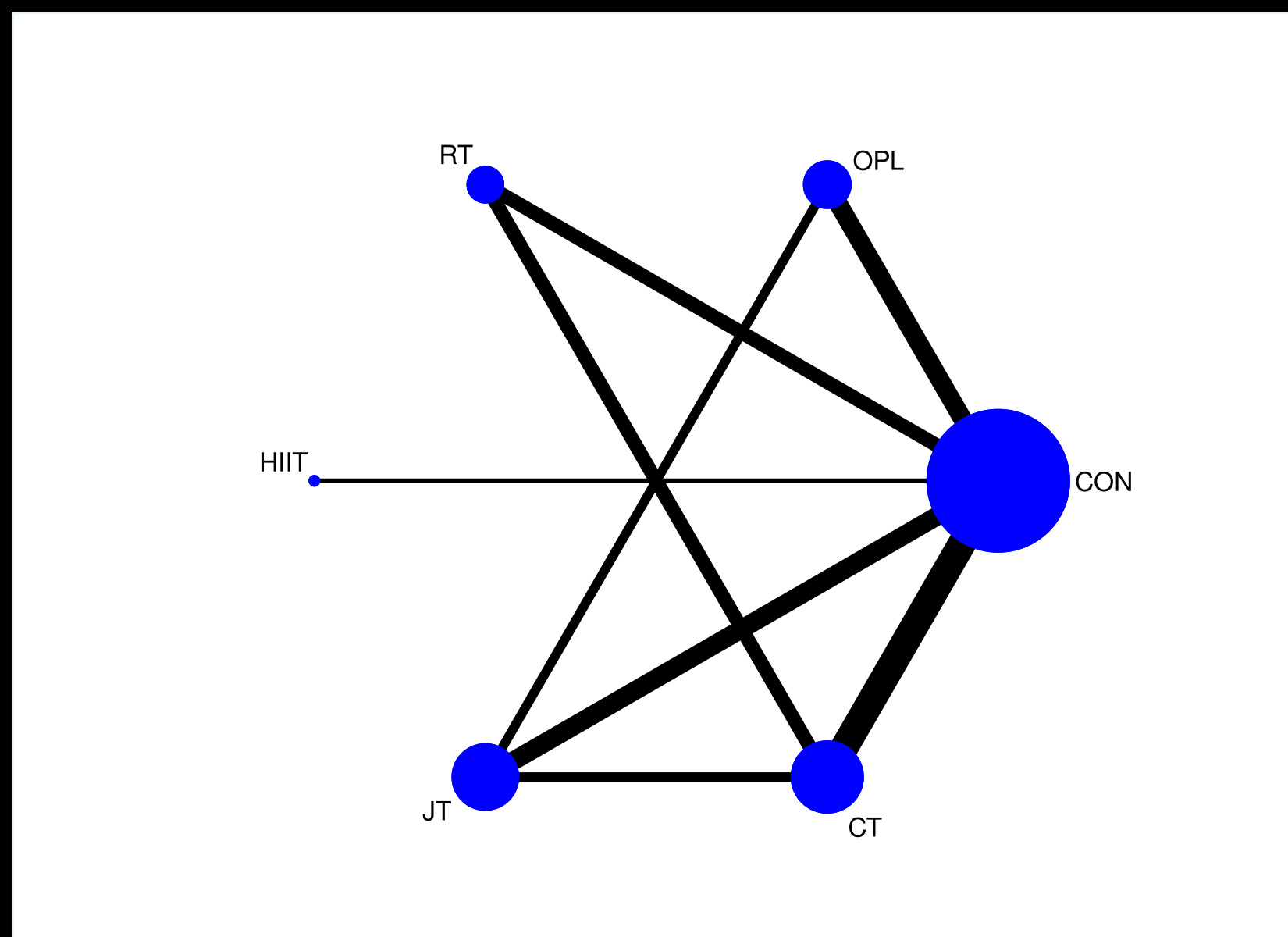

Supplement: Supplementary file 2 [file Presentation1.zip › 附件/Figs/Fig-14.jpeg]

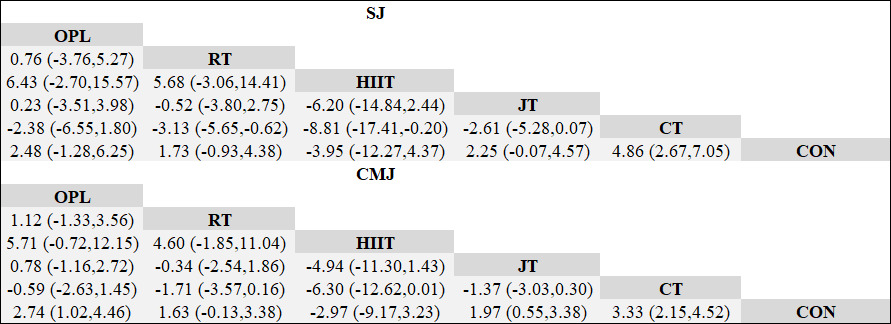

Supplement: Supplementary file 2 [file Presentation1.zip › 附件/Figs/Fig-15.jpeg]

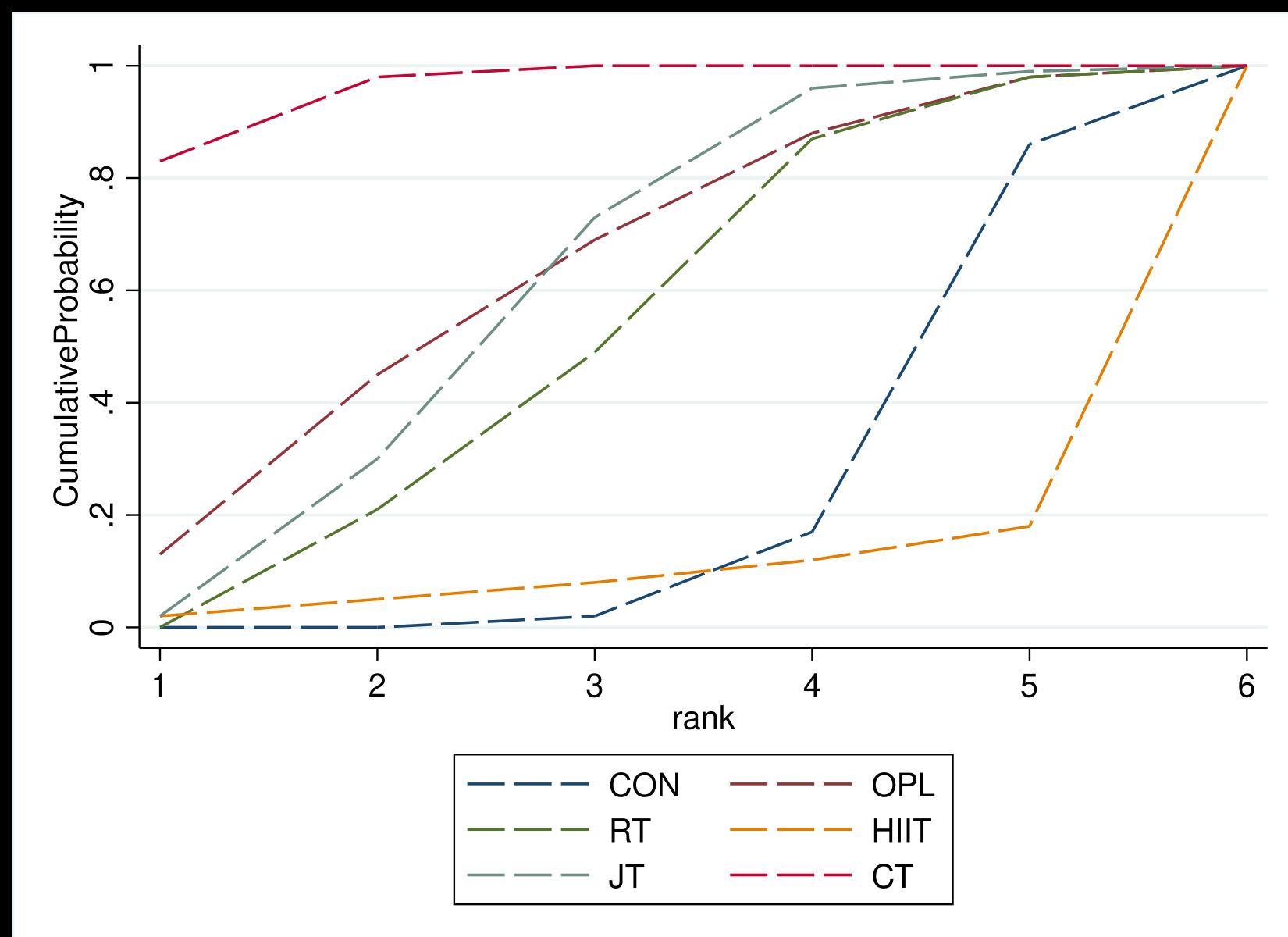

Supplement: Supplementary file 2 [file Presentation1.zip › 附件/Figs/Fig-16.jpeg]

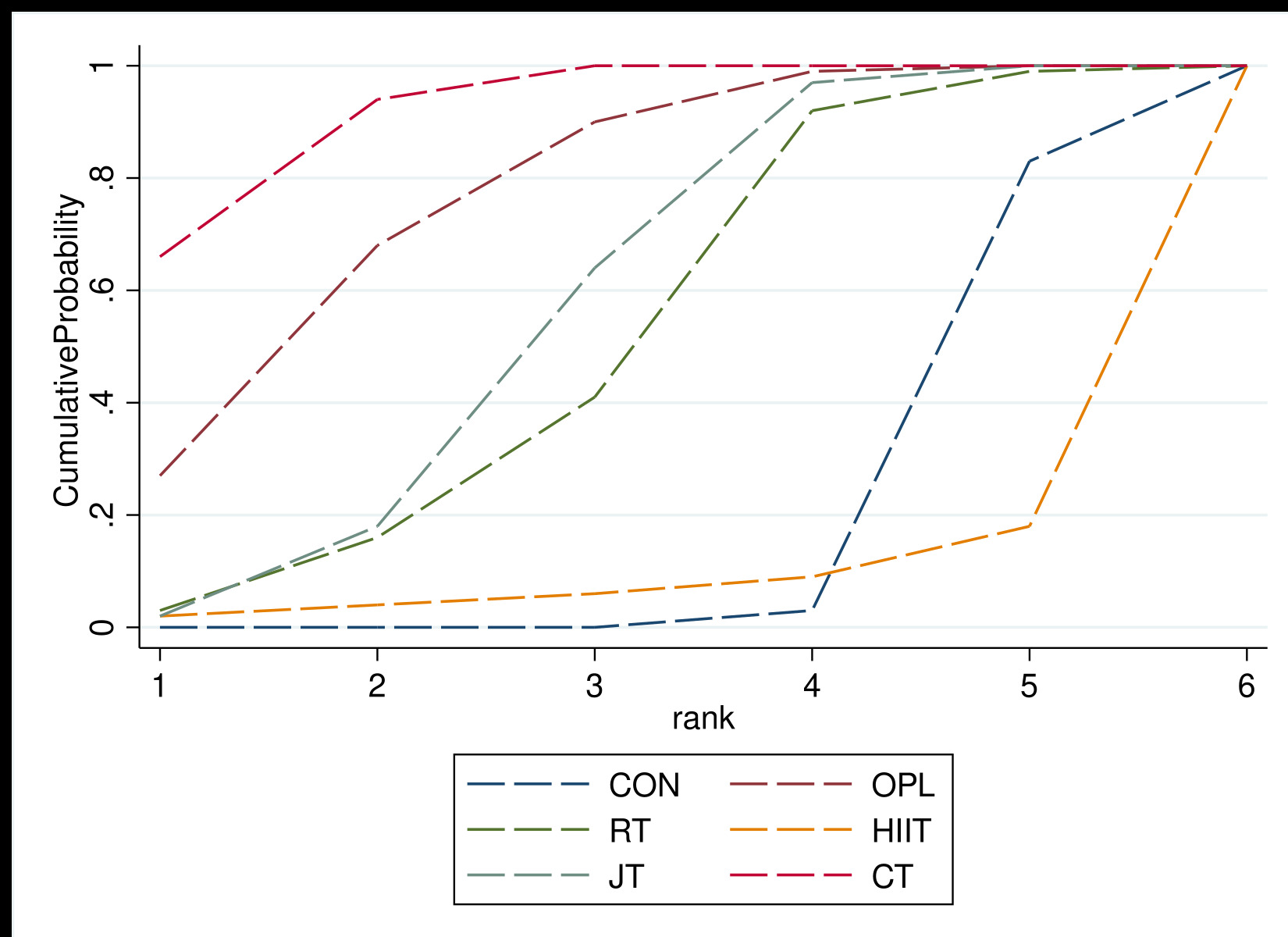

Supplement: Supplementary file 2 [file Presentation1.zip › 附件/Figs/Fig-17.jpeg]

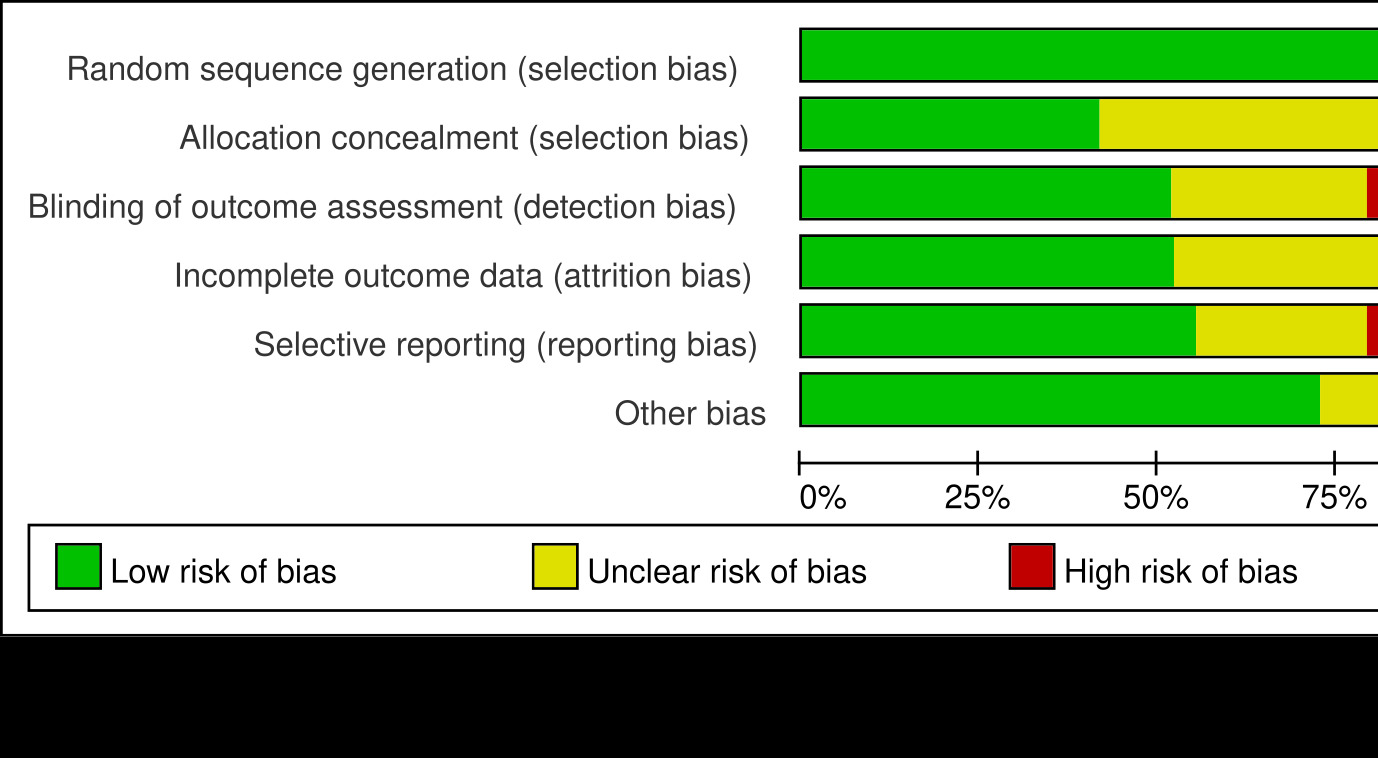

Supplement: Supplementary file 2 [file Presentation1.zip › 附件/Figs/Fig-2.jpeg]
